# An evaluation of computational methods for reconstruction of human viral DNA genomes

**DOI:** 10.1093/gigascience/giaf159

**Published:** 2025-12-26

**Authors:** Maria J P Sousa, Mari Toppinen, Lari Pyöriä, Klaus Hedman, Antti Sajantila, Maria F Perdomo, Diogo Pratas

**Affiliations:** Institute of Electronics and Informatics Engineering of Aveiro and Intelligent Systems Associate Laboratory, University of Aveiro, Campus Universitario de Santiago, 3810-193 Aveiro, Portugal; Department of Electronics, Telecommunications and Informatics, University of Aveiro, Campus Universitario de Santiago, 3810-193 Aveiro, Portugal; Department of Forensic Medicine, University of Helsinki, Kytösuontie 11, 00300 Helsinki, Finland; Department of Virology and Helsinki University Hospital, University of Helsinki, Helsinki 00290, Finland; Department of Virology and Helsinki University Hospital, University of Helsinki, Helsinki 00290, Finland; Department of Forensic Medicine, University of Helsinki, Kytösuontie 11, 00300 Helsinki, Finland; Forensic Medicine Unit, Finnish Institute for Health and Welfare, PO Box 30, FI-00271 Helsinki, Finland; Department of Virology and Helsinki University Hospital, University of Helsinki, Helsinki 00290, Finland; Institute of Electronics and Informatics Engineering of Aveiro and Intelligent Systems Associate Laboratory, University of Aveiro, Campus Universitario de Santiago, 3810-193 Aveiro, Portugal; Department of Electronics, Telecommunications and Informatics, University of Aveiro, Campus Universitario de Santiago, 3810-193 Aveiro, Portugal; Department of Virology and Helsinki University Hospital, University of Helsinki, Helsinki 00290, Finland

**Keywords:** human viral genomes, viral reconstruction, sequence assembly, survey, reproducibility

## Abstract

**Background:**

The increasing availability of viral sequencing data has led to the emergence of many optimized viral genome reconstruction tools. Given that the number of new tools is steadily increasing, it is complex to identify functional and optimized tools that offer an equilibrium between accuracy and computational resources, as well as the features that each tool provides.

**Results:**

In this article, we surveyed open-source computational tools (including pipelines) used for human viral genome reconstruction, identifying specific characteristics, features, similarities, and dissimilarities between these tools. For quantitative comparison, we created an open-source reconstruction benchmark based on viral data. The benchmark was executed using both synthetic and real datasets. With the former, we evaluated the effects on the reconstruction process of using different human DNA viruses with simulated mutation rates, contamination and mitochondrial DNA inclusion, and various coverage depths. Each reconstruction program was also evaluated using real datasets, demonstrating their performance in real-life scenarios. The evaluation measures include the identity, a normalized compression semi-distance, and the normalized relative compression between the genomes before and after reconstruction, as well as metrics regarding the length of the genomes reconstructed, computational time, and resources spent by each tool.

**Conclusions:**

We provide a fully reproducible benchmark capable of evaluating currently available reconstruction programs. The benchmark is open-source and freely available at https://github.com/viromelab/HVRS. Additionally, based on the knowledge obtained from the systematic review and the benchmark, we provide some program recommendations for different reconstruction scenarios.

## Introduction

With the advancement of high-throughput sequencing technologies, it has become easier to study the genetic makeup of viral genomes [1]. However, the reconstruction of human viral genomes from short sequence reads remains a challenging and complex process at both the laboratory and computational levels.

In particular, human samples can be characterized by complex mixtures of viral and host cells, as well as bacteria, fungi, protozoa, and other organisms. This composition can make it difficult to accurately identify and reconstruct viral genomes from metagenomic data.

At a laboratory level, additional challenges related to tissues and samples need special protocols, such as bone, teeth, and bone marrow [2, 3] or are suboptimally preserved [4].

Additionally, viruses can exhibit a high degree of genetic diversity, with different strains and variants that may be present within a single infection. This diversity can complicate the assembly and annotation of viral genomes, particularly when reference genomes are not available [5].

The reconstruction of viral sequences is crucial because they can be responsible for diseases such as cancer [6] or pandemics such as COVID-19 [7]. Additionally, their lifelong persistence may be associated with human evolution [8, 9].

Human viral genomes can range from a few thousand to over a hundred thousand base pairs in length and contain regions with average high complexity interspaced with alternated low complexity [10]. These characteristics can make it challenging to generate long and contiguous sequences from short-read sequencing data. Moreover, viral sequences are mostly present in low abundance within metagenomic data, hindering the recovery of viral sequences from noise and other nonviral sequences [11].

At a technical level, the accuracy and completeness of viral genome reconstruction can be affected by sequencing errors, PCR bias, cross-similarity, the quality and quantity of the input data, the existence of local low-complexity regions, and ambiguous read mapping.

Despite these challenges, many computer programs have been created using 1 of 3 main types of methodologies: reference-free (RF), reference-based (RB), and hybrid (HB).

The RF (or *de novo*) reconstruction has no prior knowledge of the DNA sequence to be reconstructed [12]. This methodology is ideal for reconstructing novel viral genomes, especially with high coverage but computationally intensive for reconstructing enriched samples. A well-known tool, SPAdes [13], has been extended to metaSPAdes [14], which is used in metaviralSPAdes [15] and, for the reconstruction of coronaviruses, in coronaSPAdes [16].

The RB (or targeted) reconstruction uses a reference or a database of reference sequences upon which the DNA sequence is aligned while spending on average lower computational resources than reference-free approaches, especially when the reconstruction aims to assemble already known targets. However, the database containing the references is critical for accurate reconstruction because, without the proper reference genome, the reconstructed sequence will be inaccurately predicted or unreconstructed. Therefore, choosing a diverse and high-quality database is a critical part of the methodology to accomplish accurate results.

Finally, the HB approaches combine both previous methods. For example, TRACESPipe [17] is a hybrid pipeline that provides the reconstruction using metaSPAdes [14], Bowtie [18], and BWA [19], combined with iterative refinement. These methodologies allow better adaptation to different sequences but require much more computational resources.

In this article, we provide a survey on computational tools for human viral genome reconstruction, comparing the features and characteristics of these tools. For quantitative comparison purposes, we created a fully reproducible benchmark for human viral reconstruction assessment. This benchmark provides the installation of all programs necessary and the reconstruction of human viral genomes using the selected open-source tools or pipelines.

The benchmark was tested using both generated and real datasets. The generated datasets contain several human DNA viral genomes mutated at different rates, with contamination and human DNA included, followed by the sequencing simulation using different coverage depths. The contamination sequences inserted in the datasets were generated using a pseudo-random algorithm, and the human DNA sequences were retrieved from a database. Although the human viral genomes are not synthetic, the mutation rates are generated *in silico*. This provides a controlled environment to benchmark-test following recommendation practices [20]. The mutated viral genomes are compared with the genomes reconstructed by each program using the identity, the normalized compression semi-distance (NCSD) and the normalized relative compression (NRC). Additionally, the lengths of the genome reconstructed and of the scaffolds generated, as well as the computational time and the resources spent by each tool, are evaluated.

Using real datasets, the genomes are classified so that suitable references, necessary for the execution of some RB and HB programs, can be retrieved from a database. For these datasets, the evaluation process is done using metrics that assess the length of the genome reconstructed and of the scaffolds generated, the computational time, and the resources spent by each tool.

## Methodology

This article contains 2 main frameworks: a systematic review with feature and qualitative comparison and the benchmark of the tools with quantitative measures.

### Systematic review methodology

The search strategy targeted studies that focused on viral genome reconstruction tools. The databases of articles searched were PubMed [21], IEEE Xplore Digital Library [22], and Google Scholar [23].

The search strategies used Boolean logic with MeSH terminology, including terms of reconstruction and general computational terms. The term “assembly or reconstruction” was used to search IEEE Xplore Digital Library, and the top results from Google Scholar, including related references and studies/reviews, were screened.

More specifically, when searching for articles in the PubMed database, 2 main MeSH searches were made. A more general one ((“Genome, Viral”[MAJR]) AND (“Software”[MAJR])) yielded 98 results, and a more specific search ((“Genome, Viral”[MAJR]) AND (“Software”[MAJR]) AND assembly) yielded 16 results. Moreover, IEEE Xplore Digital Library was searched, using the term “viral reconstruction,” generating 33 results. Lastly, additional articles were considered when mentioned in one or more of the articles selected or if they were found through Google Scholar. All results obtained were filtered using the criteria previously stipulated. This review exclusively included studies that provided an open-source computational tool capable of reconstructing human viral genomes. This review excluded any tools that were not able to be installed locally, tools that were unable to be installed or executed without a registration, tools only accessible through a graphical user interface (GUI), and tools that required aligned reads, contigs, or the result of other tools as input. Only articles with full texts (abstracts were excluded) and in the English language were included. The publication dates were specified as January 2000 to March 2023.

To compare the reconstruction programs found in the systematic review, several aspects were analyzed, namely, their programming language, license, operating system, and reconstruction methodology. Additionally, the connections that the reconstruction tools and pipelines shared with each other, as well as the alignment tools used by the programs, were taken into consideration.

### Reconstruction benchmark methodology

The benchmark is open-source and fully reproducible, including the installation of each selected tool from the survey. The benchmark contains a total of 73 different synthetic datasets, as well as 6 *real* datasets. All datasets analyzed were metagenomic, and our focus was on reconstructing the DNA viruses present in these samples.

Out of the 73 generated datasets, 70 consist of 4 *real* viral sequences (B19V, human parvovirus B19; HPV, human papillomavirus; VZV, varicella zoster virus; and MCPyV, Merkel cell polyomavirus), followed by different mutations rates. Additionally, 3 more generated datasets composed of *real* viral sequences explore different viral compositions, containing the viruses B19V, HPV, VZV, MCPyV, human polyomavirus 7 (HPyV7), human herpesvirus 6B (HHV6B), Epstein–Barr virus (EBV), and human cytomegalovirus (HCMV).

Each of the viral genomes was mutated with GTO [24]. In many of the datasets generated, viral contamination generated using AlcoR [25] was added, along with human mitochondrial DNA sequences retrieved from the NCBI database (reference sequence NC_012920.1). Most datasets (DS1 to DS65) then followed a read simulation process using ART [26]. The simulated sequencing process was conducted using the quality profile HiSeq 2500 for all datasets except for DS61 and DS62. The DS61 was sequenced using the Genome Analyzer II quality profile, while DS62 was sequenced with the MiSeq v1 quality profile. These quality profiles were automatically selected by ART, allowing us to observe the differences in the sequencing quality produced based on the chosen read length. The remaining synthetic datasets generated (DS66 to DS73) had their read simulation process simulated using wgsim [27], simulating error rates of 0.0, 0.01, 0.025, and 0.05, based on the error rates used in [28].

The characteristics of each generated dataset are available in Supplementary Tables S1 and S2, and the viral sequences in each generated dataset can be seen in Supplementary Table S3.

To test the performance of the reconstruction programs under real conditions, 6 real datasets were considered. The datasets are available in SRA [29] under the codes PRJNA644600 and PRJNA924035 and contain the human viral metagenome, both from single and multiple tissues. The samples of tissues contained in these datasets were retrieved from the brain, blood, kidney, bone marrow, and pulled hair and underwent targeted-enrichment sequencing to analyze the human eukaryotic DNA viruses.

The complete benchmark and scripts to replicate the results are publicly available at the repository [30]. The benchmark is flexible to add or remove more viral sequences, datasets, and tools.

**Figure 1:**
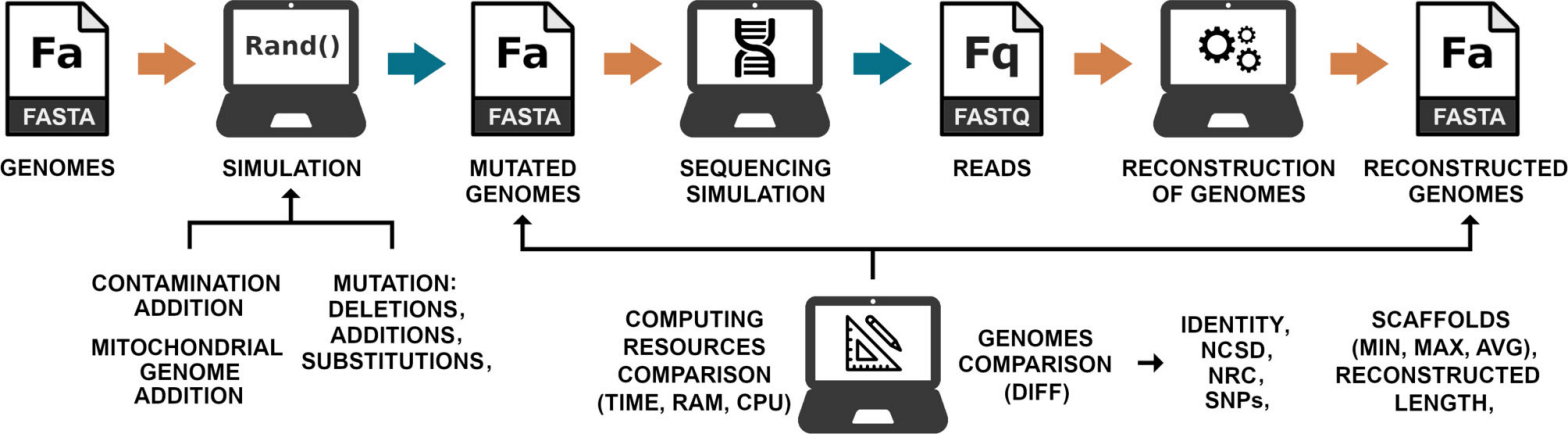
Benchmark methodology depicting the different phases. In the reconstruction phase, the genomes are used as references specifically for reference-based and hybrid reconstruction approaches that require them, while the mutated genomes are used only for evaluation in the genomes comparison phase. The blue arrow indicates that the step is optional in the execution of HVRS.

As depicted in Fig. 1, after the reconstruction process for each of the benchmarked tools, the reconstructed sequences were compared with the mutated sequences using different approaches: identity, NCSD, NRC, length of the genome reconstructed, and metrics regarding the length of the scaffolds.

The identity, also referred to as the average identity, was computed with dnadiff, from MUMmer 4 [31]. This method has been widely adopted to compare genomes, especially to highlight and provide statistics on the differences between 2 sequences. Although very practical and fast, because it is not based on lossless data compression approximations but based on alignments, it may underestimate or perform ambiguous analyses in the presence of regions of low complexity.

The NCSD is a particular case of the normalized compression distance (NCD) [32]. The NCD is an approximation of the normalized information distance (NID) [32–34], a normalized distance derived from the information distance [33] that contains the other known distances, and is defined as


(1) \begin{eqnarray*}
NCD(x,y) = \frac{\max \lbrace C(x|y), C(y|x) \rbrace }{\max \lbrace C(x), C(y) \rbrace },
\end{eqnarray*}

where *x* and *y* are 2 strings, and $C(x)$ and $C(y)$ represent the number of bits from the lossless compression on the input *x* and *y*, respectively. $C(x|y)$ and $C(y|x)$ represent the number of bits from the lossless compression on the input *x* given *y* and *y* given *x*, respectively.

However, in our case, we are required to have a relative comparison, assuming that if some reconstruction tools provide the reconstructed sequence of the contamination or mitochondrial DNA in *y*, then *y* contains more information than *x* in a perfect assembly. To avoid this constraint, we use the minimum through a semi-distance [35], defined as


(2) \begin{eqnarray*}
NCSD(x,y) = \frac{C(x|y)}{C(x)},
\end{eqnarray*}

where *x* is the sequence with the conjoint sequences of the mutated viruses before the next-generation sequencing (NGS) simulation process, and *y* is the sequence with the conjoint reconstructed sequences from a given reconstruction tool. Using the NCSD, the theoretical range of values is between slightly above 0 and close to 1, with 0 indicating that the reference and the file being evaluated are similar, and therefore, the genome is correctly reconstructed, with values close to 1 indicating that the reference and the file being evaluated are completely different, according to the compressor used.

The NRC [36–38] is also a semi-distance and is described as


(3) \begin{eqnarray*}
NRC(x || y) = \frac{ C(x || y)}{ |x| \log _{2} |\mathcal {A}|},
\end{eqnarray*}

where *x* is the sequence with the conjoint sequences of the mutated viruses before the NGS simulation process, and *y* is the sequence with the conjoint reconstructed sequences from a given reconstruction tool.

In this study, we considered the alphabet ($\mathcal {A}$) to have 4 symbols, $\mathcal {A}=\lbrace A, C, G, T\rbrace$, corresponding to the bases that compose the genomes in this study. We make sure that symbols outside this alphabet (which rarely appear in full assembled genomes considered references) are substituted by symbols from the $\mathcal {A}$ using a pseudo-random generation provided by GTO [24]. Using the NRC, the theoretical range of values is between slightly above 0 and close to 1, with 0 indicating that the reference and the file being evaluated are similar, and therefore, the genome is correctly reconstructed, with values close to 1 indicating that the reference and the file being evaluated are completely different, according to the compressor used.

The NCSD and NRC contain conditional and relative compression modes that are not supported by many data compression tools, especially for genomic sequences [39, 40]. Fortunately, the GeCo3 [41] compression tool has been shown to provide state-of-the-art compression results, at the expense of more computational resources, and it can support, besides the usual compression as $C(x)$ and $C(y)$, both relative $C(x||y)$ and conditional $C(x|y)$ compression modes. Therefore, we use GeCo3 to compute these measures.

Additionally, metrics regarding the length of the scaffolds and the number of bases reconstructed were retrieved using SeqKit [42]. These metrics can provide us with an upper bound of the amount of information reconstructed by the programs and, when combined with the number of scaffolds generated, can indicate the degree of fragmentation of the reconstruction process. These metrics are especially important when evaluating real datasets, as they can be used even when the true composition of a dataset is unknown. In the context of this review, it was considered that each scaffold is a sequence of nucleotide bases present in the FASTA file output by a reconstruction program that comes after a header.

Each of the tools was also evaluated, taking into consideration the number of SNPs and ratio of SNPs in relation to the number of bases reconstructed. The number of SNPs is a metric retrieved using dnadiff [31], and when combined with the number of bases reconstructed, retrieved by SeqKit [42], it can indicate if an RB or HB program is relying heavily on the reference genome used in the reconstruction process.

Lastly, the computational resources used were quantified through the computational time needed to reconstruct the genomes, the maximum amount of RAM used, and the CPU usage. The CPU usage is a percentage calculated as the user time plus system time divided by the total running time.

In order to obtain the statistics, each of the tools was executed 3 times for each synthetic dataset and 2 times for each real dataset. Specifically, for obtaining the time of execution of each reconstruction tool for each dataset, if 3 results were obtained, the 2 closest measures were averaged. If only 2 results were obtained, the 2 values were averaged, and if only 1 result was retrieved, it was considered as such. This process is aimed at eliminating possible outliers that may have occurred in cases where the tools successfully reconstructed the datasets in every execution cycle. For the remaining measures, the results obtained were averaged.

In the context of this review, it was considered that a tool performed a metagenomic analysis, as the identification of the sequence organisms contained in a FASTQ sample, if the genomes were reconstructed without the contents of the sample being explicitly given. This can be achieved by reference-free tools that reconstruct sequences of more than 1 species and by reference-based/hybrid tools that use a database of references from which they measure and select the most suitable ones automatically. It was also considered that metagenomic classification indicates that the scaffolds generated by the tools are classified by their provenance, without relying on previous annotations.

## Reconstruction Tools Review

In this section, we describe the human viral genome reconstruction tools found using the search methodology previously defined. Table 1 includes a summary of the characteristics of each tool, including the website, programming language, license, operating system (OS), reconstruction methodology, and whether or not the tool was reproducible. In the context of this review, it was considered that a tool is reproducible if it was successfully installed and executed, partially reproducible if it was only able to be partially installed or if it was only able to perform some of the tasks desired but did not output results, and not reproducible if was not able to be installed or executed. It should be noted that the inability to install or execute some of the tools considered may be due to version conflicts between dependencies required in the execution process, conflicts with other programs installed, conflicts with the version of the operating system used, or the dependencies being no longer available.

**Table 1:** Computational tools used for viral genome reconstruction and their characteristics. Fields OS, RM, and Re stand for operating system, reconstruction methodology and reproducible, respectively. W, L, and U (OS) stand for Windows, Linux, and Unix, respectively. RF, RB, and HB (RM) stand for reference-free, reference-based, and hybrid. NS and Lic stand for not specified and license, respectively.

Name	Website	Language	Lic	OS	RM	Re	Reference
ASPIRE	https://github.com/kevingroup/aspire	Perl	GPL	L, U	RB	No	[43]
coronaSPAdes	https://github.com/ablab/spades	C++, Python	GPL	L, U	RF	Yes	[16]
drVM	https://sourceforge.net/projects/sb2nhri/files/drVM/	Python	GPL	W, L, U	HB	Partial	[44]
EnsembleAssembler	https://github.com/xutaodeng/EnsembleAssembler	Python	NS	L	RF	No	[45]
Haploflow	https://github.com/hzi-bifo/Haploflow	C++	GPL	U	RF	Yes	[46]
IRMA	https://wonder.cdc.gov/amd/flu/irma/	Bash, Perl	GPL	L, U	RB	Yes	[47]
IVA	http://sanger-pathogens.github.io/iva	Python, Perl, Java	GPL	L, U	RF	Partial	[48]
LAZYPIPE	https://www.helsinki.fi/en/projects/lazypipe	C++, Perl, R	MIT	U	HB	Yes	[49]
metaSPAdes	https://github.com/ablab/spades	C++, Python	GPL	L, U	RF	Yes	[14]
metaviralSPAdes	https://github.com/ablab/spades	C++, Python	GPL	L, U	RF	Yes	[15]
MLEHaplo	https://github.com/raunaq-m/MLEHaplo	Perl	BSD	NS	RF	Partial	[50]
PEHaplo	https://github.com/chjiao/PEHaplo	Python	GPL	L	RF	Yes	[51]
PRICE	https://sourceforge.net/projects/pricedenovo/	C++	NS	L, U	RF	Partial	[52]
QuRe	https://sourceforge.net/projects/qure	Java	GPL	W, L, U	RB	Yes	[53]
QVG	https://github.com/laczkol/QVG	Bash	NS	L, U	RB	Yes	[54]
SAVAGE	https://github.com/haploconduct/haploconduct	C++, Python	GPL	L	RF	Yes	[55]
SPAdes	https://github.com/ablab/spades	C++, Python	GPL	L, U	RF	Yes	[13]
SSAKE	http://www.bcgsc.ca/bioinfo/software/ssake	Perl	GPL	L	RF	Yes	[56]
Strainline	https://github.com/HaploKit/Strainline	Python, Bash	GPL	L	RF	Partial	[57]
TAR-VIR	https://github.com/chjiao/TAR-VIR	Python	NS	L	HB	Partial	[58]
TRACESPipe	https://github.com/viromelab/TRACESPipe	Bash	GPL	L, U	HB	Yes	[17]
TRACESPipeLite	https://github.com/viromelab/TRACESPipeLite	Bash	GPL	L, U	RB	Yes	[17]
viaDBG	https://bitbucket.org/bfreirec1/viadbg/	C++	NS	NS	RF	No	[59]
VIP	https://github.com/keylabivdc/VIP	Perl, Bash, Python	GPL	L	HB	Partial	[60]
VGEA	https://github.com/pauloluniyi/VGEA/	Bash, Python	GPL	L, U	HB	No	[61]
ViQUF	https://github.com/borjaf696/ViQUF	Python, C++	NS	NS	RF	No	[62]
ViralFlow	https://github.com/WallauBioinfo/ViralFlow	Python, Bash	NS	W, L, U	RB	Partial	[63]
VirGenA	https://github.com/gFedonin/VirGenA	Java	NS	W, L, U	HB	Yes	[64]
ViSpA	https://alan.cs.gsu.edu/NGS/?q=content/vispa	Java, Python	NS	U	RB	Yes	[65]
V-pipe	https://github.com/cbg-ethz/V-pipe	Python, Bash	Apache	L, U	HB	Yes	[66]

According to the type of assembly strategy, the computational tools listed in Table 1 are divided into 3 categories: RB, RF, and HB.

### RB reconstruction tools

The RB category includes several computational tools such as QuRe [53], QVG [54], TRACESPipeLite [17], ViSpA [65], ViralFlow [63], and IRMA [47].

QVG [54] is a pipeline prepared to deal with single-end and pair-end reads. QVG checks the quality and adapter content of the input reads and filters them using fastp [67]. Afterward, the reads are aligned to the reference with BWA [68], and duplications are marked with sambamba [69]. The variant call is performed by freebayes [70] through parallel [71] for calling variant positions of multiple samples simultaneously. QVG includes multiple statistic outputs; for example, breadth and depth coverage values are provided along with R plots.

TRACESPipeLite [17] is a variation of TRACESPipe for single-end and pair-end sequencing. TRACESPipeLite includes a high-quality curated human viral database. TRACESPipeLite uses AdapterRemoval [72] for trimming, followed by FALCON-meta [73] for classification, specifically to identify the genomes with the highest similarity (best reference) in the database according to the reads. Then, the reads are aligned to the best references using BWA [68], while the consensus sequences are generated using SAMtools [74] and bcftools [75].

QuRe [53] reconstructs viral quasispecies and corrects errors, using the Poisson distribution, while providing support for reads longer than 100 bp. QuRe is platform-independent as it has been implemented in Java. QuRe can align sequence fragments with a reference genome and partition the genome into sliding windows based on coverage and diversity. Using a heuristic algorithm, QuRe reconstructs the individual sequences of the viral quasispecies while including their prevalence. This feature is achieved by matching multinomial distributions of distinct viral variants that overlap across the genome partition. Additionally, QuRe has a built-in Poisson error correction method and a post reconstruction probabilistic clustering, both parameterized on given error rates in homopolymeric and non-homopolymeric regions.

ViSpA [65] focuses on reconstructing quasispecies from 454 pyrosequencing reads. ViSpA uses MOSAIK [76] as an alternative to SEGEMEHL [77] for aligning the reads to the reference and extending the reference. Afterward, it creates a consensus sequence, constructs the read graph, assembles the contigs, and estimates the candidate quasispecies sequence frequencies. ViSpA also uses an error correction algorithm, assembles viral variants based on maximum-bandwidth paths in weighted read graphs, and does frequency estimation via expectation maximization.

ViralFlow [63] is a pipeline created to analyze and assemble the SARS-CoV-2 virus from Illumina paired-end amplicon sequencing data. This pipeline trims the reads using fastp [67] and aligns them against a reference genome with BWA [19]. The aligned reads are sorted and indexed with SAMtools [74], and the minor variants are analyzed using both SAMtools and iVar [78]. ViralFlow is also capable of identifying intrahost variants, evaluating the quality of the consensus and the set of mutations retrieved using nextclade [79], and retrieving the assembly metrics with bamdst [80].

IRMA [47] is a pipeline designed to assemble highly variable viral RNA genomes, detect indels, and perform variant calling and phasing. The pipeline begins with the filtering of the input reads based on their length or quality. The filtered reads are then aligned to a reference genome, using SAM [81] or BLAT [82], creating a new consensus sequence, which allows more reads to be aligned. After this process, the pipeline enhances the consensus sequence generated using the implementation of the striped Smith–Waterman provided by [83].

### RF reconstruction tools

SPAdes [13], a tool for *de novo* assembly that is capable of single-cell and multicell assembly, can be applied to single, paired-end, and mate-pairs reads. SPAdes uses *k*-mers for building the initial de Bruijn graph [84, 85], and in the following stages, it performs graph-theoretical operations that are based on graph structure, coverage, and sequence lengths. The errors are minimized iteratively. Four main phases are used in SPAdes. The first phase is the assembly graph construction, where SPAdes employs different *k*-mer de Bruijn graphs, which detect and remove bubble and chimeric reads. In the second phase, the pairs of *k*-mers are adjusted and exact distances between *k*-mers in the genome are estimated. The third phase is the paired assembly graph construction. The last phase is the contig construction. Here, SPAdes constructs the contigs and maps the reads back to their positions in the assembly graph after graph simplification. SPAdes serves as a base for a myriad of other assembling pipelines, including the following pipelines that come with the SPAdes package: metaSPAdes [14], metaviralSPAdes [15], and coronaSPAdes [16].

The metaSPAdes [14] is a pipeline developed to assemble genomes from metagenomic datasets. To reconstruct the genomes, metaSPAdes uses a de Bruijn graph generated by SPAdes [13] and, from it, creates the assembly graph. Afterward, it uses a modified version of exSPAnder [86] to resolve repeats and scaffolding in the graph.

The metaviralSPAdes [15] is a pipeline made to identify and reconstruct viral genomes in metagenomic samples. It starts by using metaSPAdes [14] to construct the assembly graph and modifies the graph with viralAssembly. The provenance of the contigs assembled is then assessed by viralVerify [87]. Finally, viralComplete [88] determines whether the viral contigs correspond to the entirety of the viral genome by comparing them to a database, using a naive Bayesian classifier.

The coronaSPAdes [16] is another pipeline variation of SPAdes that focuses on the recovery of coronaviruses but is also capable of making RNA species recovery. It is a pipeline that uses rnaviralSPAdes [89] to assemble the input data and afterward uses HMMPathExtension to align hidden Markov models (HMMs) to the assembly graph, which is then used to create the assembly graph paths. Although HMMs based on Pfam SARS-CoV-2 [90] are included in coronaSPAdes, there is a possibility to create a custom set, providing additional flexibility.

Another RF reconstruction program is SAVAGE [55], specifically for quasispecies—the ensemble of viral strains populating an infected person. SAVAGE is based on overlap graphs and relies on deep coverage datasets ($\ge 20,000$x). It has 2 main modes of operation: RF reconstruction, which uses FM-index–based techniques, and RB, which aligns the reads to the reference provided. In detail, SAVAGE performs overlap graph construction using pairwise overlaps with FM-index or read-to-reference alignment followed by BLAST [91], then checks for the quality of the overlap using an overlap score and a mismatch rate. This process provides an undirected overlap graph that, through read orientations, enables a directed overlap graph. Finally, the processes of transitive edge removal and read clustering are made recursively until convergence is achieved, and the final contigs are output.

As SAVAGE, viaDBG [59] is a tool that focuses on reconstructing viral quasispecies, but it uses a de Bruijn graph–based approach. The viaDBG has 2 main phases: error correction and haplotype inference. The error correction phase identifies solid *k*-mers via the LoRDEC algorithm [92]. The haplotype inference phase builds a de Bruijn graph and obtains the unary paths of the graph. The paired-end information is then added to the graph, and some heuristics are used to polish the paired-end information. Finally, the haplotypes are obtained by splitting the graph nodes based on the paired-end information and obtaining the unary paths from the modified graph.

ViQUF [62] is an assembler designed specifically for reconstructing quasispecies, and it is able to provide frequency estimations for the contigs. The ViQUF methodology involves several steps. First, it selects *k*-mers above a predefined frequency threshold and utilizes them to construct a de Bruijn graph. Subsequently, it solves a min-cost flow problem on a flow network created for each pair of adjacent vertices, using paired-end information. This process generates an approximate paired assembly graph, where the suggested frequency values serve as edge labels. Finally, the original haplotypes are obtained through a greedy path reconstruction, guided by a min-cost flow solution within the approximate paired assembly graph.

SSAKE [56] is an assembler that uses an overlap-based strategy specialized for short reads. The SSAKE methodology involves loading the sequence reads in a hash table keyed by uniqueness, along with values representing the number of occurrences of each sequence in the set. Afterward, the sequences are organized using a prefix tree, including their reverse complement. Next, the sequences are sorted by decreasing occurrence. Then, the most frequent sequences are progressively extended by the longest sequences that can be aligned to them. When this process is no longer possible, the extended sequence is complemented and the extension process is repeated.

PRICE [52], a specialized *de novo* tool designed for paired reads, employs a combination of overlap and de Bruijn graph–based strategies to extend contigs. Initially, it identifies and merges identical or closely similar reads to form contigs through overlap graphs. Subsequently, contigs that fall below a user-defined threshold are further extended using a de Bruijn graph approach. Finally, the sequences generated from both assemblies are combined, and redundant information is removed.

Haploflow [46] is a tool for strain-resolved assembly of viral genomes that uses information on differential coverage between strains to deconvolute the assembly graph into strain-resolved genome assemblies. The Haploflow methodology involves creating a de Bruijn graph, finding the connected components, and turning them into unitig graphs. From the unitig graphs, a set of contigs is generated, based on the flows of the graphs.

Another RF pipeline is Strainline [57], which assembles viral haplotypes from noisy long-read data. To mitigate the drawbacks of noisy long reads, the errors are corrected using a local de Bruijn graph strategy, utilizing both Daccord [93] and Daligner [94]. Then, the reads are organized in clusters, determined by Minimap2 [95], where the reads of each cluster are ordered by Spoa [96] and are iteratively extended using an overlap-based strategy. Lastly, the resulting contigs are filtered to remove low-divergence and low-abundance haplotypes.

MLEHaplo [50] is a pipeline designed to reconstruct viral haplotypes from paired-end data. It corrects errors with BLESS [97] and represents the reads in a de Bruijn graph. The de Bruijn graph is then used by ViPRA [50] to compute a path cover of the graph, retaining the paths that could be haplotypes, from which the haplotypes are chosen.

EnsembleAssembler [45] is a pipeline designed to analyze metagenomic reads and assemble small viral, bacterial, and eukaryotic mitochondrial genomes. The tool assembles the reads using de Bruijn graph–based assemblers, particularly SOAPdenovo2 [98], ABySS [99], and MetaVelvet [100]. This assembly is performed by splitting the input data into chunks with 100K reads, followed by the assembly of each chunk. All contigs generated are combined, and short contigs are filtered. Finally, an overlap graph–based assembler, either CAP3 [101] or Minimo from the AMOS package [102], is used to generate the final contigs.

PEHaplo [51] is a pipeline that assembles viral haplotypes from deep sequencing data. It starts by trimming and correcting the input reads using Karect [103], and afterward, it constructs an overlap graph. Then, using a path finding algorithm, the contigs are retrieved.

IVA [48] is an assembler developed to reconstruct RNA viruses from short-read pairs with highly variable depths. The input reads can be trimmed using Trimmomatic [104]. Then, the most abundant *k*-mer is found using kmc [105], and it is extended with reads that have a perfect match to it, which generates the contigs. The contigs are then extended using SMALT [106] and SAMtools [74]. Lastly, the contig ends are trimmed, and overlapping contigs are merged.

### HB reconstruction tools

The hybrid approaches are rare (not to be confused with short- and long-read hybrid assembly) and usually provide a unique architecture that goes beyond the diversity of internal tools chosen.

V-pipe [66] is a pipeline that supports quality control, read mapping and alignment, low-frequency mutation calling, and inference of viral haplotypes. Reads are aligned employing a reference-guided approach using ngshmmalign [66]. This approach is based on profile hidden Markov models that are tailored to small and highly diverse viral genomes. For the read alignment, the reference sequence can be provided or built *de novo* from the read data using VICUNA [107]. Alternatively, reads can be aligned using BWA [68] or Bowtie2 [18]. Intermediate results are provided in the form of a consensus sequence per sample, a multiple sequence alignment of all consensus sequences. Finally, variants are called by different tools, namely, LoFreq [108] and ShoRAH [109].

TRACESPipe [17] is a pipeline for the reconstruction of viral genomes from single- and multiple-organ samples. It includes modules for quality control, filtering, assembly, and annotation of viral genomes. TRACESPipe uses a hybrid approach that combines both *de novo* and reference-based assembly methods, which can increase the accuracy and completeness of the reconstructed viral genomes. This pipeline can handle sensitive data, and the genome assembly uses the reference with the highest similarity to provide RB assembly with Bowtie2 [18], in addition to RF assembly with metaSPAdes [14], followed by iterative refinement using BWA alignment [110].

ASPIRE [43] is a pipeline that uses an RF assembler, either SPAdes [13] or SGA [111], for generating scaffolds and, afterward, aligns the scaffolds obtained to a reference using MUMmer 3 [31], while filtering the viral unlikely contigs. Then, the gaps present in the genome are filled using GapFiller [112]. ASPIRE uses an iterative refinement procedure involving repeated alignments of scaffolds to the latest version of the reconstructed viral genome, followed by gap filling and a correction step (using Bowtie2 [18], SAMtools [74], and bcftools [75]), based on allele frequencies derived from read alignments.

TAR-VIR [58] is a pipeline that was designed to reconstruct and classify RNA viral reads. TAR-VIR starts by eliminating reads that do not have viral origin, using BMTagger [113], Bowtie2 [18], and Karrect [103]. Then, it maps the reads to a reference using BWA [19] or Bowtie2 and obtains a seed set. Reads that have a significant overlap with the seed set are iteratively added to the seed set, which is used by PEHaplo [51] to do the strain-level assembly.

The drVM [44] is a pipeline that identifies, reconstructs, and annotates known viral genomes from NGS reads. The drVM aligns the reads to a viral database using SNAP [114], partitions the aligned reads into genus groups, and reconstructs the reads of each genus group using SPAdes [13]. Lastly, the annotation of the reconstructed genomes is made using BLAST [115].

VIP [60] is a pipeline that can identify and discover viruses from metagenomic NGS data. VIP includes quality controls and is able to filter reads with similarity to the host using Bowtie2 [18]. The input reads are aligned against the Virus Pathogen Resource [116] and Influenza Research Database [117] or against the NCBI Refseq [118] and neighbor genomes, which allows the taxonomic classification of each read. Afterward, an RF reconstruction is done using Velvet-Oases [119, 120], and the resulting contig is added to the phylogenetic tree.

LAZYPIPE (version 2) [49] is a pipeline designed to discover and reconstruct viruses from metagenomic NGS data obtained from clinical, animal, and environmental samples. LAZYPIPE includes the filtering of the input reads based on their quality as well as filtering the reads that contain the genome of the host, using Trimmomatic [104], fastp [67], BWA-MEM [68], SAMtools [74], and SeqKit [42]. Afterward, it assembles the filtered reads using MEGAHIT [121] or SPAdes [13], which are scanned to check for genelike regions with MetaGeneAnnotator [122] and subsequently translated to amino acid sequences with SeqKit [42] and used to assign NCBI taxonomy IDs to the contigs. Later, the reads remaining after the filtering process are aligned to the contigs using BWA-MEM. In addition to assembling the genomes, LAZYPIPE also annotates, estimates the abundance, and generates statistics.

VirGenA [64] is a software capable of separating strains into genetic groups, creating a consensus sequence for each group, and detecting evidence of cross-contamination. VirGenA removes the adapter and PCR primer sequences, using Trimmomatic [104], then aligns the reads to one or more reference sequences and clusters adjacent reads using Usearch [123], which are then joined, generating contigs. The contigs are then used to generate an overlap graph, which, along with the reference, is used to merge the contigs.

VGEA [61] is a pipeline that focuses on the reconstruction of RNA viruses. It starts by trimming and filtering the reads using fastp [67], and using BWA [19], it aligns the reads to a reference human genome. Afterward, using SAMtools [74], the unmapped reads are extracted and split into FASTQ files, which are reconstructed using IVA [48]. The reads are then processed in terms of quality and contamination and mapped to a reference, using shiver [124]. Lastly, VGEA cleans up the reconstruction made using SeqKit [42]. In addition to reconstructing the genomes, VGEA is also capable of evaluating its performance with QUAST [125].

### Uncategorized reconstruction tools

In this subsection, we include several tools that were not in accordance with the criteria defined in the methodology or that could not be categorized entirely into one of the reconstruction methodologies.

HAPHPIPE [126] is a modular pipeline that assembles genomes using an RF or RB strategy. It is designed to assemble viral consensus sequences and haplotypes. It includes quality controls, such as trimming the reads with Trimmomatic [104] and correcting them using SPAdes [13]. Then, if the RF strategy is used, the contigs are generated with SPAdes [13] and then joined into scaffolds using MUMmer 3 (or after) [31]. Otherwise, if the RB strategy is chosen, the reads are aligned to the reference sequence using Bowtie2 [18] and then are realigned with Picard [127].

There are many tools to reconstruct viral genomes that require the output of other tools or prealigned reads in order to perform the reconstruction process. Examples of these tools are Virus-VG [128], VG-Flow [129], QSdpR [130], PredictHaplo [131], aBayesQR [132], HaploClique [133], QuasiRecomb [134], ViQuaS [135], RegressHaplo [136], CliqueSNV [137], TenSQR [138], ShoRaH [109], viralFlye [139], Arapan-S [140], and ContigExtender [141].

Moreover, there are tools that can reconstruct viral genomes but do not comply with other criteria stipulated, such as VirAmp [142], Vipie [143], EDGE COVID-19 [144], and VICUNA [107]. VirAmp can only be installed via Amazon Web Services, Vipie and VICUNA require a registration to be used, and EDGE COVID-19 is only accessible through a GUI. We acknowledge viral-ngs [145], which, unfortunately, does not have a complete article describing the methodology. We also acknowledge viralrecon [146], which is a pipeline capable of performing variant calling for viral samples for both Illumina and Nanopore sequencing data, but it only supports the assembly of Illumina sequencing data. Additionally, both Genome Detective Virus tool [147] and EzCOVID19 [148] do not meet the criteria stipulated as they are only available online and require a registration.

Furthermore, there are other tools that, while not being specifically designed to reconstruct viral genomes, can be used for that purpose. Examples include Falcon [149], HiCanu [150], hifiasm [151], and IPA [152], which are tools that reconstruct HiFi reads. Currently, long reads are starting to be included also in viral reconstruction pipelines, and the integration of these assemblers is of significant importance. Additionally, there are assembly tools that are included directly or by option in some of the described pipelines, such as MEGAHIT [121], Velvet [119], MetaVelvet [100], SOAPdenovo2 [98], ABySS [99], CAP3 [101], Minimo [102], and SGA [111].

### Considerations between tools and pipelines

In the realm of human viral genome reconstruction, there are 2 primary categories of programs: individual tools and pipelines. Tools are standalone programs that are meticulously optimized using programming languages known for their efficiency, such as C, C++, Rust, and others. However, their flexibility is constrained to specific functions, such as trimming, assembly, or classification. On the other hand, pipelines are comprehensive programs that facilitate the integration of multiple instructions and tools in a unique and customized manner. Typically, pipelines employ programming languages like Bash, Python, or Perl. The flexibility of pipelines surpasses that of individual tools due to the wider array of available subprograms and parameters, thereby offering a greater number of sequential options. Additionally, pipelines often prioritize experimentation, allowing for optional usage and combination of specific tools. However, it is important to note that this heightened flexibility often comes at a higher computational cost.

**Figure 2:**
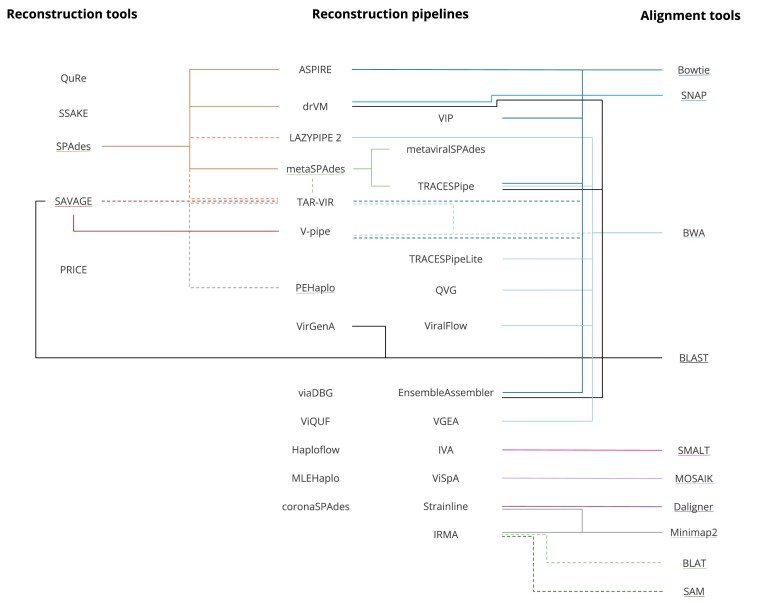
Classification of programs that perform genome reconstruction in three groups: reconstruction tools, reconstruction pipelines and alignment tools. The reconstruction pipelines contain the two columns in the middle. Programs that have their names underlined are the basis tool. Connections made with a dashed line indicate that the tool is optional. The tools or pipelines include all versions in the name as proxies. The order in which the tools appear follows no particular criteria.

Fig. 2 provides an illustration of the connections between the reconstruction tools and pipelines found in the present systematic review, as well as the alignment tools used by these programs.

Accordingly, there are two types of tools, reconstruction (*de novo*) tools, which may contain small alignment options, and exclusively alignment tools. From the included pipelines, the 2 most used reconstruction tools are SPAdes and SAVAGE, while the most commonly used alignment tools are BWA and Bowtie.

The alignment tools can be used for several purposes depending on the program that includes them. The following paragraphs outline the purposes each alignment tool has in each reconstruction pipeline considered.

Bowtie is used by ASPIRE to correct the reconstruction made but can also be used to filter out the host genome, as seen in the reconstruction pipelines VIP and EnsembleAssembler. Additionally, it can be used to align reads to a reference, as is the case in TRACESPipe and V-pipe. Furthermore, this tool can be used by TAR-VIR to obtain seed reads.

SNAP is included in only one of the pipelines considered in this review, drVM, and it is used to generate a viral database and to align the input reads to it.

BWA is used by LAZYPIPE and VGEA to filter out the host genome and by TRACESPipe to combine scaffolds. BWA is also used to obtain seed reads, as is the case of the TAR-VIR pipeline, and to align the reads to a reference, as shown in TRACESPipeLite, V-pipe, QVG, and ViralFlow.

BLAST is used for several different purposes: to identify to what species a scaffold belongs to, as observed in TRACESPipe; to construct overlap graphs, as seen in SAVAGE; and to perform contig annotation, as shown in drVM. BLAST is also used by VirGenA, which uses this alignment tool to identify chimeras, and by EnsembleAssembler to improve the quality of the reads and remove the adaptors present.

SMALT is included in only one of the pipelines considered, IVA, to extend the contigs previously obtained, with reads that do not have a perfect match to them.

MOSAIK is used by the pipeline ViSpA to align the input reads to a reference sequence provided by the user.

Minimap2 is utilized by Strainline to determine pairwise alignments between seed reads and the corrected reads and by IRMA in the final assembly step. Strainline also uses another alignment tool, Daligner, in the error correction process to compute read overlaps.

BLAT and SAM are included in the IRMA pipeline to align the input reads to a reference genome.

## Benchmark Results

The results reported in this section follow the methodology described in the “Methodology” section, specifically in the subsection “Reconstruction benchmark methodology.” The results obtained regarding the synthetic datasets generated and accompanying figures are freely available either in the present section or in the Supplementary Material and are fully reproducible using the tool available at [30]. The benchmark was executed on a computer running Linux Ubuntu 22.04.4 LTS with an Intel Core i7-6700 CPU $@$ 3.40 GHz $\times$ 8 processor and with 64 GB of RAM, and the computational resources were limited, when possible, to 48 GB of RAM and 6 CPU threads. For each program, the execution time was capped at 6 hours, either overall or per reference genome provided.

The analysis focused on 7 characteristics of the datasets (existence of mitochondrial DNA and contamination, variation in the percentage of SNP and depth coverage, read length, viral composition, and error rates) and takes into consideration several metrics, focusing especially on the identity, NCSD, NRC, number of nucleotide bases reconstructed, the number of scaffolds generated, the average length of the scaffolds, and the ratio between the number of SNPs and number of bases reconstructed. When comparing length-based metrics, the gaps (“N” bases) present in the reconstructed genome were removed for the comparison of the results to be fairer. When comparing sets of datasets, the average performance in a given metric was the average between the values obtained in the datasets considered.

Additionally, a comparison of the overall performance obtained by the reconstruction programs across all synthetic datasets considered will be provided.

### Mitochondrial DNA and contamination

In order to assess the impact of the inclusion of mitochondrial DNA and contamination on the performance of the reconstruction programs considered, datasets with a low percentage of single-nucleotide polymorphisms (SNPs), specifically 1%, and a depth coverage varying between 2× and 40× were generated and evaluated. A low percentage of SNPs was considered as the data obtained from real-life scenarios often contain diversity and as the viral genomes contained in the samples may have mutations, making them different from reference genomes.

**Figure 3:**
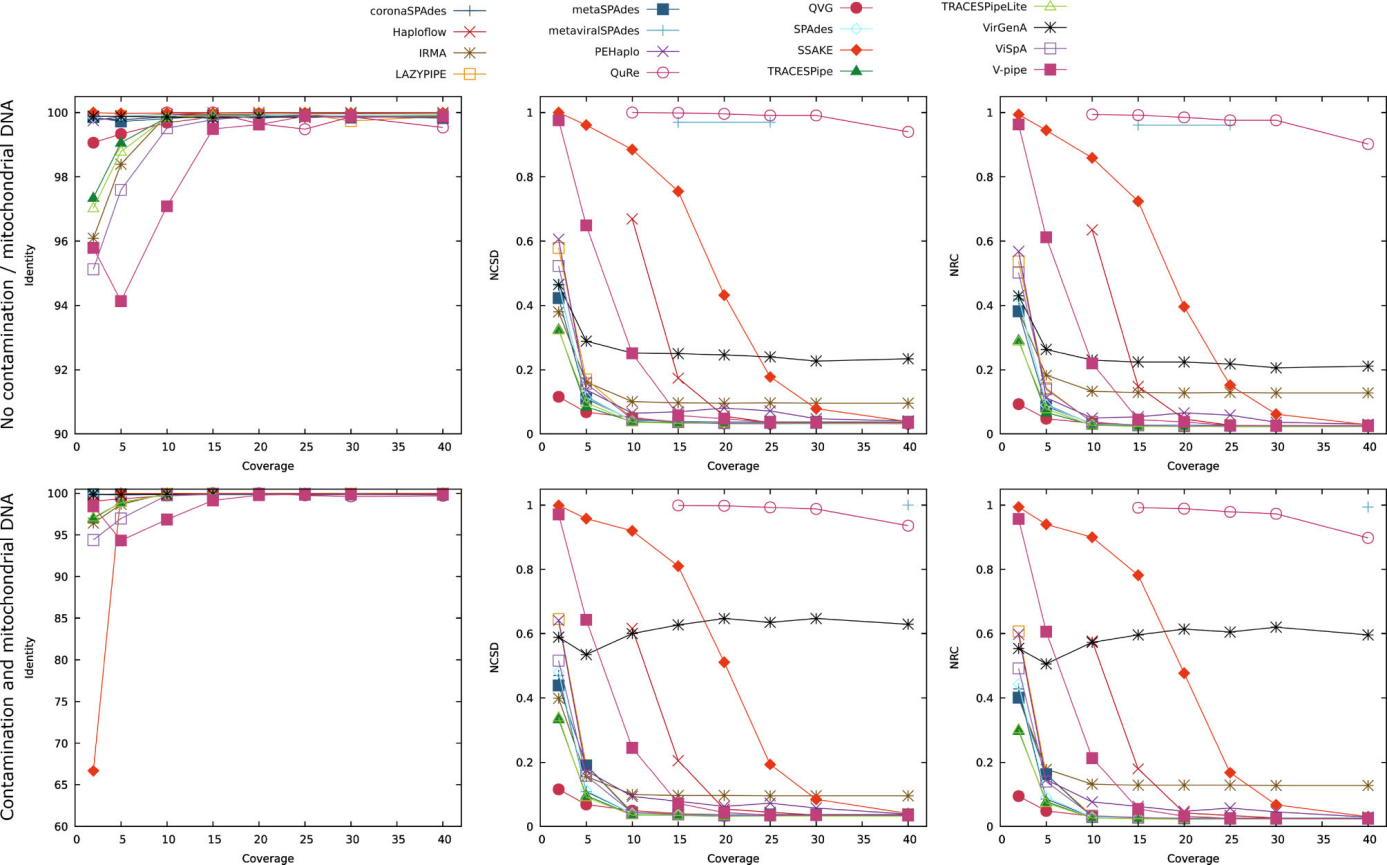
Comparison of the performance of the reconstruction programs according to the identity, NCSD, and NRC for datasets without contamination and mitochondrial DNA (DS1 to DS8) and datasets with contamination and mitochondrial DNA (DS9 to DS16), with depth coverage ranging between 2× and 40×. Optimal identity values are close to 100, while lower NCSD and NRC values indicate better results.

This analysis focused on datasets 1 to 16, and the performance obtained in terms of the identity, NCSD, and NRC in these datasets is represented in Fig. 3. The top 3 plots of Fig. 3 represent the results obtained in datasets 1 through 8, which contain no contamination or mitochondrial DNA, whereas the bottom plots represent the results obtained in datasets 9 to 16, which contain both contamination and mitochondrial DNA.

Not all reconstruction programs were able to reconstruct datasets 1 through 8, with Haploflow and QuRe unable to reconstruct datasets with a depth coverage equal to or lower than 5× and metaviralSPAdes only reconstructing 2 of the datasets considered (DS4 and DS6).

The identity tended to improve at higher depth coverage, often stabilizing at values close to 100. It should be noted that the identity is an alignment-based metric, and as such, it may provide ambiguous analyses of the data.

The results obtained regarding the NCSD and NCD are similar to each other, as they are both compression-based evaluation metrics that assess the reconstructed genomes as a whole. Similar to the identity, the tools tend to improve their performance as the depth coverage increases. Exceptions to this trend are QuRe and metaviralSPAdes, whose performance remained close to 1, and VirGenA, the performance of which stabilized at around 0.25 for depth coverages of 5× and over. QuRe and metaviralSPAdes are incapable of reconstructing many bases, and therefore, the results obtained using them are more limited than the remaining tools. VirGenA does not significantly alter its performance, but according to the data obtained, it is only capable of reconstructing scaffolds corresponding to VZV in these datasets. The limitations in the results obtained using QuRe, metaviralSPAdes, and VirGenA may be due to these programs being designed to work in specific datasets or the capping of the computational resources used to 48 GB of the RAM and 6 threads of CPU, when possible.

In terms of the number of reconstructed bases (excluding gaps), most of the programs reconstructed on average between 75,000 and 210,000 bp, and the performance of each tool has maintained itself stable throughout the datasets considered. However, both QuRe and metaviralSPAdes reconstructed considerably fewer bases per dataset than the remaining programs, reconstructing fewer than 6,000 bp on average. On the other hand, ViSpA outputs significantly more bases on average than the remaining programs (over 880,000 bp per dataset), as it reconstructed the quasispecies, generating several different scaffolds for a single viral genome. This phenomenon is particularly noticeable in datasets with depth coverage ranging between 2× and 10×.

Regarding the average length of the scaffolds and fragmentation of the genomes, IRMA, QVG, TRACESPipe, TRACESPipeLite, and V-pipe were able to reconstruct scaffolds with an average length of over 20,000 bp (excluding gaps), while producing the expected number of scaffolds (between 4 and 5), considering there are 4 viral genomes contained in the datasets, indicating that these tools were able to identify the individual genomes and reconstruct them whole.

The ratio between the number of SNPs and the number of bases reconstructed was stable throughout the datasets. However, there are some outliers, namely, PEHaplo, QVG, and QuRe. PEHaplo and QVG improved their performances as the depth coverage of the datasets increased, both due to the reduction in the number of SNPs contained in the reconstructed genome and the increase in the number of bases reconstructed. Conversely, the performance of QuRe decreased at depth coverages of 20× and over due to a significant increase in the number of SNPs in relation to the number of nucleotide bases reconstructed.

The bottom 3 plots of Fig. 3 represent the results obtained in terms of the identity, NCSD, and NRC for datasets 9 through 16, which contain both contamination and mitochondrial DNA. Again, not all datasets were able to be reconstructed by all of the reconstruction tools, with Haploflow not reconstructing datasets with a depth coverage below 10×, QuRe requiring a depth coverage of at least 15×, and metaviralSPAdes only reconstructing the dataset with 40× depth coverage. This means that the addition of contamination and mitochondrial DNA to the datasets can affect the reconstruction ability of the programs.

With the inclusion of contamination and mitochondrial DNA, the results obtained followed the same trend as the ones obtained in datasets 1 to 8. However, the average performance of the tools was inferior, with a decrease in performance of 1.1% in terms of the identity, 6.7% in terms of the NCSD, and 6.9% in terms of the NRC. It should be noted that the results obtained using VirGenA were not as good in terms of the NCSD and NRC in relation to the ones obtained in datasets without the addition of contamination and mitochondrial DNA, indicating that this tool may be sensitive to these additions.

The number of reconstructed bases followed the same trend observed in datasets 1 to 8, and most programs reconstructed between 50,000 and 250,000 bp per dataset, on average. However, the number of reconstructed bases increased by 20.7% in relation to the previous group of datasets, which may indicate that some tools reconstructed part of the mitochondrial DNA and contamination present in the samples.

In terms of the average scaffold length, PEHaplo, QuRe, SSAKE, and VirGenA produced scaffolds with an average length of less than 1,000 bp. In contrast, IRMA, QVG, TRACESPipe, and V-pipe were able to reconstruct the genomes using the expected number of scaffolds and output scaffolds with over 25,000 bp. Overall, the average length of the scaffolds increased 2.0% in relation to the previous set of datasets.

The ratio of SNPs in relation to the number of bases reconstructed decreased by 11.7% but followed the same trend observed in the previous group of datasets.

**Figure 4:**
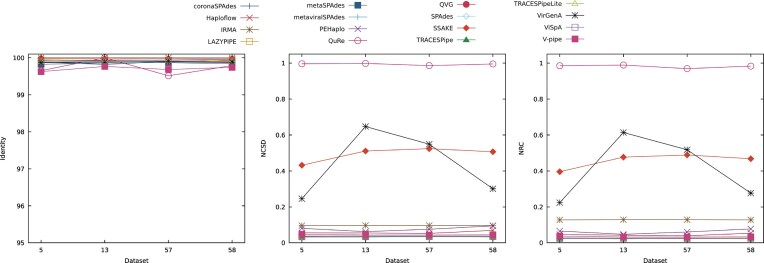
Comparison of the performance of the reconstruction programs according to the identity, NCSD, and NRC for datasets DS5, DS13, DS57, and DS58, which show the effects that contamination and mitochondrial DNA have on the performance of the reconstruction programs. Optimal identity values are close to 100, while lower NCSD and NRC values indicate better results.

To assess the effect of the addition of contamination and mitochondrial DNA on the results obtained, datasets 5, 13, 57, and 58 were considered. The datasets have a read length of 150 bp, a depth coverage of 20×, and the same viral composition (B19V, HPV, MCPyV, and VZV), differing only on whether or not contamination and/or mitochondrial DNA were added. Dataset 5 contained no contamination or mitochondrial DNA, dataset 57 contained contamination, dataset 58 contained mitochondrial DNA, and dataset 13 contained both contamination and mitochondrial DNA. Fig. 4 shows the effects that contamination and mitochondrial DNA have on the performance of the reconstruction programs.

All reconstruction programs were able to reconstruct the 4 datasets considered, except for metaviralSPAdes, which output no results.

Regarding the identity, the tools displayed a stable performance throughout all datasets considered. The values of the identity ranged between 99.5 and 100, and the most variance in performance was observed in the tool QuRe. In this metric, the best results overall were obtained for dataset 13, followed by datasets 58, 5, and 57.

In terms of the NCSD and NRC, the performance was best overall for dataset 5, which did not contain contamination or mitochondrial DNA. The next best results were obtained for dataset 58, which contained just mitochondrial DNA, followed by dataset 57, with just contamination, and the worst performance, on average, was obtained in dataset 13, containing both contamination and mitochondrial DNA. These plots also show that VirGenA has the most variation in its performance, indicating susceptibility to these additions to the datasets. Additionally, QuRe obtained the least favorable performances across all datasets considered.

In contrast to the results obtained using the NCSD and NRC, the tools overall reconstructed the most bases from dataset 13, indicating that some tools may have reconstructed the contamination and mitochondrial DNA added. This can also be observed in datasets 57 and 58, in which most tools also increased the number of reconstructed bases in relation to dataset 5, which had no contamination or mitochondrial DNA added. Exceptions to this behavior can be observed in the performances of QVG, QuRe, and VirGenA. QVG had a stable performance in datasets 5, 13, and 58, decreasing its performance in dataset 57. QuRe maintained a stable performance throughout all datasets considered, but it did not reconstruct many bases in any of the datasets considered. VirGenA is susceptible to the additions present, as previously discussed, and its performance declines especially when contamination or contamination and mitochondrial DNA are added to the datasets.

In terms of the average length of the scaffolds, it was observed that the scaffolds reconstructed in dataset 13 had the greatest length on average, followed by dataset 58, dataset 5, and, lastly, dataset 57. The best results were obtained in dataset 13, which may be explained by some tools reconstructing contamination and mitochondrial DNA, as previously indicated.

The ratio of SNPs in relation to the number of bases reconstructed is generally low, and the programs had little variance in their performance throughout the datasets considered.

It is important to note that when human samples are sequenced, it is unrealistic for only viral genomes to be present in a sample. Therefore, analyzing datasets that contain only viral genomes does not accurately represent real-life scenarios. Taking this aspect into consideration, the following analyses were done using datasets containing both mitochondrial DNA and contamination.

### Percentage of SNPs and depth coverage

To analyze the effects that the percentage of SNPs and depth coverage can have on the performance of the reconstruction programs considered, several datasets containing varying percentages of SNPs, ranging between 0% and 15%, were considered. The depth coverage of the datasets varied between 2× and 40×, and all datasets contained both mitochondrial DNA and contamination.

SNPs are single nucleotide bases that are not equal to the correspondent base in another genome. In the reconstruction process, SNPs correspond to ambiguities in the genome, which make it more difficult for a dataset to be reconstructed accurately.

**Figure 5:**
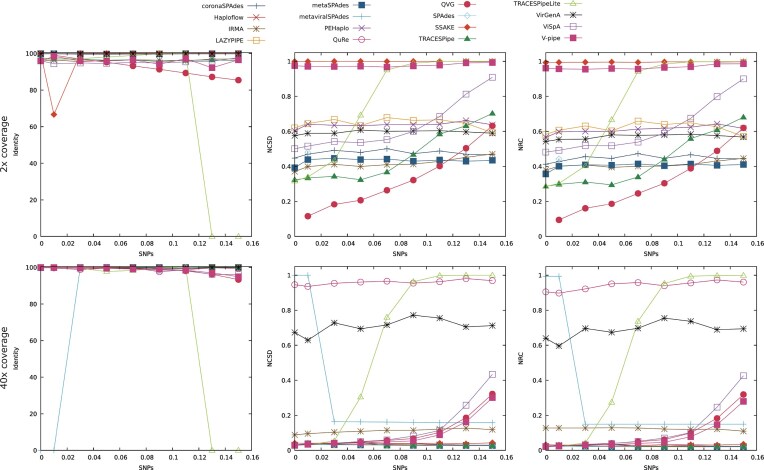
Comparison of the performance of the reconstruction programs according to the identity, NCSD, and NRC for datasets DS17 to DS24 plus DS9 (2× coverage) and datasets DS49 to DS56 plus DS16 (40× coverage). The x-axis represents the ratio of SNPs added to the viruses in the datasets. Optimal identity values are close to 100, while lower NCSD and NRC values indicate better results.

The datasets considered for this analysis were datasets 17 through 56, plus datasets 9, 10, 11, 13, and 16. Fig. 5 represents the results obtained by the reconstruction programs when analyzing datasets with depth coverage of 2× and 40×.

The top 3 plots of Fig. 5 represent the results obtained in terms of the identity, NCSD, and NRC with a depth coverage of 2×. At this depth coverage, each part of the genome is present on a dataset, on average, only 2 times, which makes the reconstruction process difficult.

It should be noted that metaviralSPAdes and QuRe were not able to reconstruct data from any of the datasets considered with depth coverage 2×. Haploflow reconstructed only the datasets with 13% or 15% of SNPs (datasets 23 and 24), QVG reconstructed all datasets except dataset 17, and SPAdes was only able to recover dataset 9.

The identity correlated inversely with the percentage of SNPs present in the datasets. This can be explained as the identity is calculated based on the genome fragments that can be aligned, which declines as the quality of the reconstruction made decreases. The drop in performance suffered by SSAKE to 66.7% in dataset 9 can be explained as in one of the reconstructed files, dnadiff was not able to align any fragment of the reconstructed genomes to the viral genomes. This shows that SSAKE does not have a deterministic behavior. The drop observed in TRACESPipeLite’s performance when the percentage of SNPs contained in the datasets was 13% or over was due to the pipeline only reconstructing mitochondrial DNA in those datasets.

The NCSD and NRC showed 2 trends in the performance of the reconstruction tools: either it was relatively stable or it decreased as the percentage of SNPs increased. The NCSD and NRC confirmed the drop in performance of TRACESPipeLite, as in the datasets where the identity dropped to 0, the NCSD and NRC had values close to 1. Haploflow, SSAKE, and V-pipe had a lower performance across all datasets reconstructed based on the NCSD and NRC as they did not reconstruct many bases from the datasets (on average less than 10,000 bp per dataset). QVG obtained better results in relation to the remaining programs until the percentage of SNPs reached 11%, from which point forward, the best performance was obtained by metaSPAdes. It should be noted that the ratio of SNPs in relation to the number of bases reconstructed obtained by QVG followed an almost linear increase as the percentage of SNPs increased, indicating that this tool may be overreliant on the reference genome when the depth coverage is low, making TRACESPipe, IRMA, or metaSPAdes more reliable in this scenario. For the remaining tools, the ratio of SNPs in relation to the number of bases reconstructed was low.

Regarding the number of bases reconstructed by each of the programs, most programs had a stable performance. However, ViSpA reconstructed a significantly greater amount of bases than the other tools throughout most datasets, providing multiple genome sequences for a single virus. The drop in the number of bases reconstructed by ViSpA coincides with the decrease in its performance in terms of the NCSD and NRC. As previously mentioned, the tools Haploflow, SSAKE, and V-pipe reconstructed fewer bases than the remaining tools, outputting, on average, less than 10,000 bp per dataset. When taking into consideration the number of bases reconstructed and the number of scaffolds produced, IRMA, TRACESPipe, and QVG performed the best as they output the most bases, using the expected number of scaffolds, considering there were 4 viral genomes in the datasets considered.

The average number of bases per scaffold reconstructed was low or tended to be lower as the percentage of SNPs contained in the datasets increased, with all programs except for QVG, TRACESPipe, ViSpA, and IRMA outputting less than 10,000 bp per scaffold, on average.

The bottom 3 plots of Fig. 5 show the results obtained in terms of the identity, NCSD, and NRC with depth coverage of 40×, which is much higher than the one shown in the plots with 2× coverage. Thus, the tools have to handle a greater amount of data, but they have less difficulty in discovering a consensus sequence as there are, on average, 40 copies of every part of the genome. It is worth noting that with a depth coverage of 40×, all programs were able to reconstruct all datasets considered, except for QVG, which could not reconstruct dataset 49. This shows a significant improvement in the tools’ capacity to reconstruct datasets in relation to when the depth coverage considered was 2×.

The identity decreased slightly with the increase in the percentage of SNPs present in the sample, confirming the previously obtained results. Significant changes to the values of the identity are observed in both metaviralSPAdes and TRACESPipeLite, caused by the reconstruction of exclusively contamination and/or mitochondrial DNA, which are not considered in this metric. Overall, the identity increased by 1.0% in relation to the average performance obtained using datasets with a depth coverage of 2×.

Regarding the NCSD and NRC, most tools maintained their performance throughout the datasets considered. However, some tools decreased their performance as the percentage of SNPs increased, namely, TRACESPipeLite, ViSpA, QVG, and V-pipe. With a depth coverage of 40×, coronaSPAdes, Haploflow, LAZYPIPE, metaSPAdes, PEHaplo, SPAdes, and TRACESPipe obtained a consistently good performance, while QuRe and VirGenA had the least favorable performances. The performance of the reconstruction programs in these datasets improved by 66.9% in terms of the NCSD and by 67.3% in terms of the NRC, in comparison to the average performance obtained with a depth coverage of 2×.

In relation to the number of bases reconstructed, most programs had a stable performance throughout the datasets considered. ViSpA, however, reconstructed a considerably greater amount of bases than the other tools, especially in the datasets with a percentage of SNPs of 7% or greater. This may be attributed to higher ambiguity in the genome, interpreted by ViSpA as different variants. The increase in the depth coverage has led to a rise in the number of bases reconstructed by 38.5% on average, in relation to the previous group of datasets.

The average length of the scaffolds was overall stable. However, it was lower for VirGenA, SSAKE, PEHaplo, and QuRe, which have output scaffolds with an average length under 2,500 bp. With the increase in depth coverage from 2× to 40×, the average length of the scaffolds increased by 178.8%. This improvement is especially significant, as it was much greater than the increase in the number of reconstructed bases, indicating that the reconstructed genomes were less fragmented.

The ratio of SNPs in relation to the number of bases reconstructed was low and stable for most programs, with the exception of QVG and V-pipe, the performance of which declined in datasets with over 9% of SNPs added. The ratio of SNPs in relation to the number of bases reconstructed decreased by about 81.6% in relation to the datasets with 2× depth coverage.

Overall, as the percentage of SNPs increased, the performance of the reconstruction tools tended to decline, suggesting that higher levels of ambiguity make the reconstruction process more difficult. Additionally, the performance of the programs considered improved at higher depth coverages. These findings can be explained as lower depth coverages mean that every part of the genome is present on the sequenced files, on average, fewer times, which makes the reconstruction process harder due to the reduced amount of data available. In addition to lower performance, low-depth coverages can affect the reconstruction programs’ ability to reconstruct genomic sequences.

SPAdes, coronaSPAdes, LAZYPIPE, and TRACESPipe maintained constant performances at increased SNPs and depths higher than 5×. In scenarios of low-depth coverage and low percentage of SNPs, good performances were obtained with TRACESPipe and QVG, although QVG may be overreliant on the reference genome provided. In scenarios with low-depth coverage and a high percentage of SNPs, better performance was obtained with metaSPAdes.

### Read length

**Figure 6:**
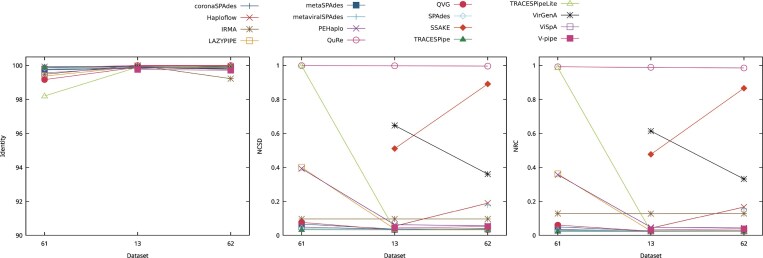
Comparison of the performance of the reconstruction programs according to the identity, NCSD, and NRC for datasets 13, 61, and 62, which show the effects that different read lengths have on the performance of the reconstruction programs. Optimal identity values are close to 100, while lower NCSD and NRC values indicate better results.

To assess how different read lengths affect the reconstruction process, datasets 13, 61, and 62 were considered. These datasets have a depth coverage of 20×, have 1% of SNPs, and contain both mitochondrial DNA and contamination. The reads included have a length of 75 bp in dataset 61, a length of 150 bp in dataset 13, and a length of 250 bp in dataset 62, all of which are considered short reads. Fig. 6 represents the results obtained in terms of the identity, NCSD, and NRC in these datasets.

The effect of the read length on the performance of the programs was evidenced by the fact that at 75 bp (dataset 61), only 11 of the 16 tools were able to reconstruct the genomes. In contrast, at 150 bp, all reconstruction programs except metaviralSPAdes were able to reconstruct the dataset, and at 250 bp, the dataset was reconstructed by all tools.

In terms of the identity, the performance was generally constant throughout the datasets considered, varying between 98% and 100%. The best overall performance was obtained in dataset 13, with a read length of 150 bp; the second-best average performance was obtained in dataset 62, with reads of 250 bp; and the least favorable overall performance was obtained in dataset 61, with reads with 75 bp of length.

Regarding the NCSD and NRC, the reconstruction programs either improved or maintained their performance from datasets 61 to 13 and, except for VirGenA, maintained or decreased their performance between datasets 13 and 62. Regarding these metrics, the best overall performance was obtained for dataset 13, followed by dataset 62 and, lastly, dataset 61.

The number of reconstructed bases increased or leveled from dataset 61 to dataset 13, with the exception of IRMA and ViSpA, which reconstructed considerably more bases in dataset 61. From datasets 13 to 62, except for IRMA and VirGenA, the tools maintained or decreased the number of bases reconstructed. Regarding this metric, the best performance was obtained when using the dataset with 75 bp, followed by the dataset with reads of 250 bp and, lastly, the dataset with reads of 75 bp.

The average scaffold length also increased or stabilized between datasets 61 and 13, with the exception of ViSpA and IRMA, whose performance decreased. Between datasets 13 and 62, most tools maintained their performance, with Haploflow, metaSPAdes, and LAZYPIPE having the most significant decreases and IRMA and VirGenA increasing the average scaffold length the most. Overall, the best results were obtained with dataset 61, followed by dataset 62 and dataset 13.

The ratio between the number of SNPs and the number of bases reconstructed tended to be even or decreased between datasets 61 and 13, as well as leveled or increased between datasets 13 and 62. The best overall performances for this metric were observed for dataset 13, followed by datasets 62 and 61.

Although the performance was best overall for the dataset with reads of length 150 bp based on the identity, NCSD, NRC, and ratio of SNPs in relation to the number of bases reconstructed, the best results regarding the number of reconstructed bases and average length of the scaffolds were obtained in the dataset with a read length of 75 bp. This indicates that there may not be a read length that is best for every metric considered and that the results obtained may vary based on the tools considered when calculating the average of each metric. It should be noted that these tests were made on simulated datasets with a constant percentage of SNPs and that the reads considered in these tests were all considered short reads and contained the same genomes and characteristics, except for the read length. This comparison may have different results if different read lengths, viral genomes, or read characteristics are tested.

### Viral composition

**Figure 7:**
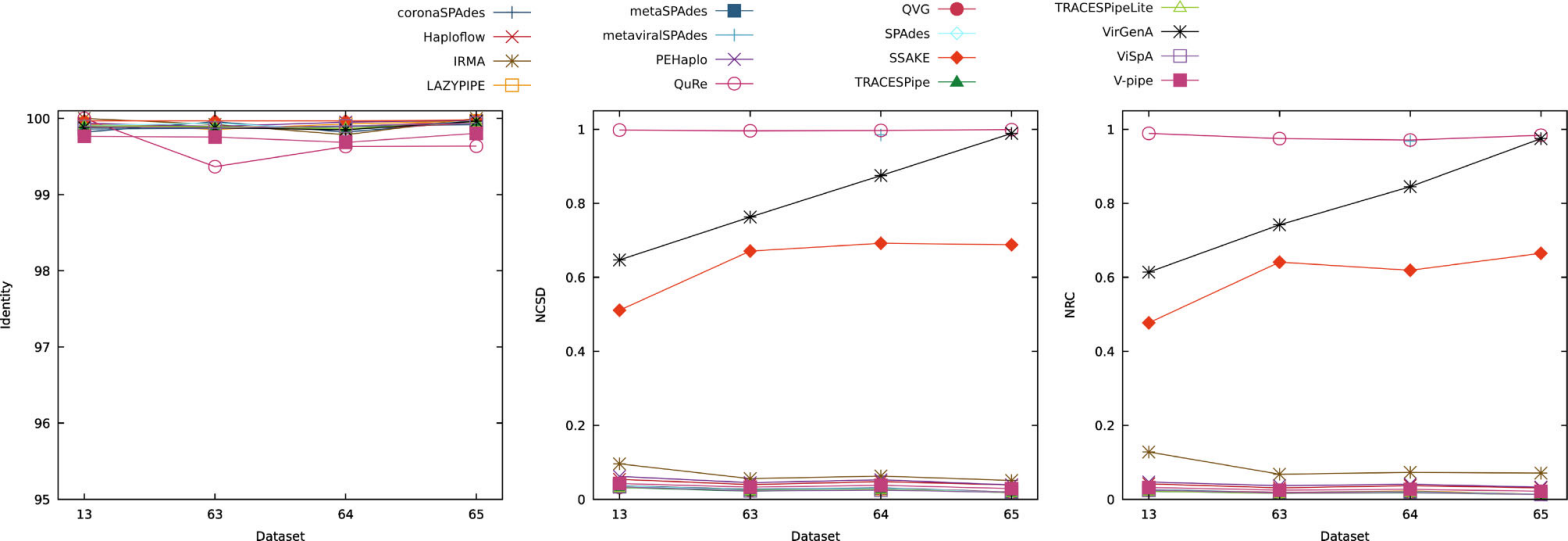
Comparison of the performance of the reconstruction programs according to the identity, NCSD, and NRC for datasets 13, 63, 64, and 65, which show the effects that different viral compositions have on the performance of the reconstruction programs. Optimal identity values are close to 100, while lower NCSD and NRC values indicate better results.

To study the effect that different viral compositions can have on the performance of the reconstruction programs, 4 different combinations of viruses were considered. The datasets 13, 63, 64, and 65 were considered for this analysis, and the viruses contained in each dataset can be found in Supplementary Table S3. All of these datasets have a depth coverage of 20×, 1% of SNPs, and a read length of 150 bp. Fig. 7 illustrates the performances obtained based on the identity, NCSD, and NRC for each dataset considered.

All reconstruction programs considered reconstructed these datasets, except for metaviralSPAdes, which only output results for dataset 64.

Overall, the identity had little variation, with values ranging between 99% and 100%.

Regarding the NCSD and NRC, the performance was overall stable, with the most variation belonging to SSAKE and VirGenA. The best overall results were observed for TRACESPipe, TRACESPipeLite, ViSpA, coronaSPAdes, QVG, and SPAdes. Although QuRe had a stable performance throughout all datasets, that performance was low, as it reconstructed fewer bases than the remaining programs considered.

The number of reconstructed bases was greater for ViSpA, QVG, and PEHaplo, reconstructing over 400,000 bp per dataset, on average. On the other hand, metaviralSPAdes and QuRe had a lower performance as they reconstructed, on average, less than 6,000 bp per dataset.

The average length of the scaffolds was greatest for QVG, TRACESPipe, ViSpA, and V-pipe, all outputting scaffolds with, on average, over 50,000 bp. The reconstruction programs VirGenA, PEHaplo, SSAKE, and QuRe obtained the least favorable performances in this metric, averaging under 1,000 bp per scaffold.

Most reconstruction programs had little variance on the ratio of SNPs in relation to the number of bases reconstructed, which may indicate that under these scenarios, there was generally little reliance on the reference genomes provided.

It should be taken into consideration that the datasets considered still have in common at least 3 viruses (B19V, HPV, and VZV) and that different viral compositions and characteristics of the datasets may affect the results.

### Error rates

**Figure 8:**
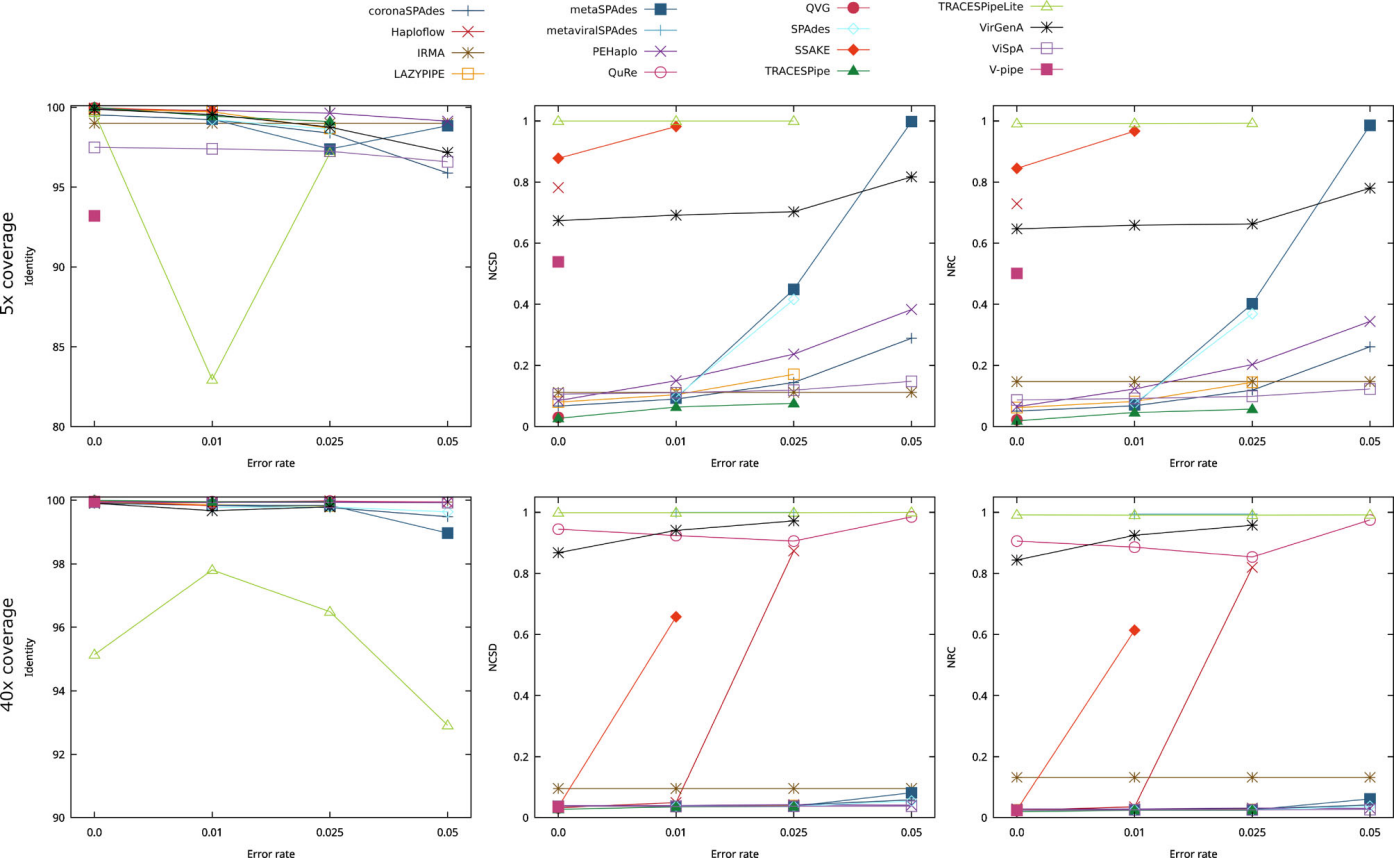
Comparison of the performance of the reconstruction programs according to the identity, NCSD, and NRC for datasets DS66 to DS69 (5× coverage) and datasets DS70 to DS73 (40× coverage). The x-axis represents the error rates considered in the datasets. Optimal identity values are close to 100, while lower NCSD and NRC values indicate better results.

To evaluate the impact of the inclusion of different error rates on the performance of the reconstruction programs considered, datasets with error rates ranging between 0.0 and 0.05 and depth coverages of 5× and 40× were generated. This analysis focused on datasets 66 to 73, and the performance obtained in terms of the identity, NCSD, and NRC in these datasets is represented in Fig. 8. The top 3 plots in Fig. 8 represent the results obtained in datasets 66 through 69, which were simulated with 5× depth coverage, while the bottom 3 plots represent the results obtained in datasets 70 to 73, simulated with 40× depth coverage.

Although every tool was able to reconstruct at least 1 dataset when the depth coverage was 40×, with a depth coverage of 5×, both metaviralSPAdes and QuRe were unable to reconstruct any of the datasets considered. Additionally, the number of tools that failed to reconstruct the datasets tended to rise as the percentage of error rates added increased, as expected.

With a depth coverage of 5×, the identity obtained by the tools tended to be stable and over 95%. The V-pipe and TRACESPipeLite were an exception to this, as they obtained particularly low performances in datasets 66 and 67, respectively.

The performance of the tools in terms of the NCSD and NRC in this scenario tended to decline as the error rates increased. The most variation in results is observed in both SPAdes and metaSPAdes, indicating that although they can reconstruct most of the datasets considered, they may be particularly susceptible to these changes. Additionally, both IRMA and ViSpA were able to get a good performance in these metrics while reconstructing all of the datasets considered. It should be noted that some of the results obtained in DS66 contrast with those obtained in DS25, which has the same characteristics in terms of composition and read length, differing only in the read simulation tool considered. This is particularly noticeable in the tools metaSPAdes, QVG, SPAdes, and TRACESPipeLite, whose performance or ability to reconstruct the datasets was affected depending on the read simulation tool considered.

The number of reconstructed bases tended to be stable, with IRMA and ViSpA reconstructing considerably more bases than the remaining tools, outputting over 700,000 bp per dataset on average, which may explain their good performance in these datasets.

The average length of the scaffolds also tended to be stable, except for ViSpA, which output significantly more bases in the dataset with an error rate of 0.05 compared to the remaining.

The ratio of SNPs in relation to the number of bases reconstructed tended to increase as the percentage of error rates rose. However, there were exceptions, such as ViSpA and IRMA, where this ratio remained low across all the datasets examined.

When considering datasets with a greater depth coverage (40×), the identity remained stable and above 98.5% for all tools except for TRACESPipeLite, which varied between 92.5% and 98% and metaviralSPAdes, which obtained an identity value of 0% in the 2 datasets it reconstructed. Overall, the identity decreased by 3% in relation to the previous scenario.

With the increase in depth coverage, the NCSD and NRC improved by at least 8% on average. The most variance in NCSD and NRC was observed in the results obtained with SSAKE and Haploflow, while TRACESPipeLite, QuRe, and VirGenA obtained the least favorable performances in these metrics. Conversely, the tools ViSpA, PEHaplo, and coronaSPAdes obtained the best performances in these metrics while reconstructing all datasets considered.

The number of bases reconstructed by each of the tools tended to be stable, with the exception of SSAKE and Haploflow, whose number of reconstructed bases declined significantly as the error rates considered increased. Although the NCSD and NRC have overall improved in relation to the previous scenario, on average, the number of reconstructed bases decreased by 46.6%.

The average length of the reads tended to be stable or to decrease as the error rates increased. In these datasets, ViSpA, IRMA, and TRACESPipe output the longest scaffolds, having on average over 30,000 bp of length. Overall, the average length of the scaffolds output decreased by 22.6% when compared to datasets 66 to 69.

The ratio of SNPs in relation to the number of bases reconstructed tended to be low while the error rates of the datasets were between 0.0 and 0.025, with TRACESPipeLite, metaSPAdes, coronaSPAdes, and SPAdes decreasing their performance when reconstructing the dataset, with an error rate of 0.05. On average, the ratio of SNPs in relation to the number of bases reconstructed decreased by 83.7% in relation to the previous group of datasets.

The increase in error rates within the datasets typically led to a decline in the performance of the reconstruction tools evaluated. However, this negative impact on tool performance was lessened when reconstructing datasets with a higher depth coverage. Additionally, reconstructing datasets with greater depth coverage not only improved performance but also allowed more programs to successfully assemble the data. Moreover, we found that the results obtained in these scenarios may not be directly comparable to the ones obtained using datasets simulated using ART, as the performance and ability to reconstruct the datasets have differed for some of the tools considered, potentially because they are optimized for certain characteristics of the reads that are differently simulated by wgsim and ART. In this context, it is also worth noting that there are other tools capable of simulating sequencing data with different features, as highlighted in [153].

### General comparisons

**Figure 9:**
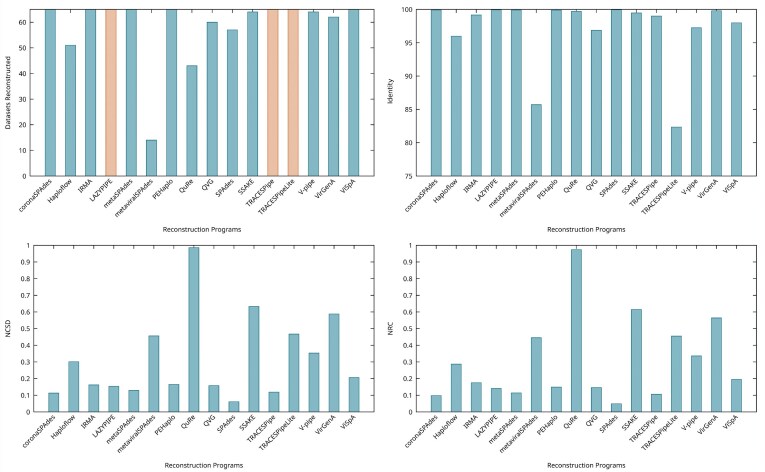
Comparison of the average performance of the reconstruction programs according to the number of datasets reconstructed, identity, NCSD, and NRC. In the “Datasets Reconstructed” plot, the bars that appear in orange indicate that the corresponding pipeline does metagenomic classification. For the number of datasets reconstructed, the results are best the closest they are to 65 (number of synthetic datasets considered). Optimal identity values are close to 100, while lower NCSD and NRC values indicate better results.

To evaluate the performance of each of the programs, the number of datasets reconstructed by each program was calculated. To ensure fairness, only the 65 datasets simulated with ART (DS1 to DS65) were considered in this comparison, as the tools’ performance varied based on the read simulation tool used. In the context of this review, the number of datasets reconstructed is the count of datasets reconstructed by a tool, regardless of whether or not the results were obtained in a single execution cycle. This aspect is important as some tools—namely, metaSPAdes, QuRe, SSAKE, and VirGenA—sometimes did not output results for a dataset in at least one of the execution cycles. Based on the number of datasets reconstructed by each program, we averaged the performance obtained in terms of the identity, NCSD, NRC, number of bases reconstructed, average scaffold length, ratio of SNPs in relation to the number of bases reconstructed, number of scaffolds generated, execution time, and computational resources. Some of the results obtained for each reconstruction program are illustrated in Fig. 9, whereas the remaining are available in the Supplementary Material.

The coronaSPAdes, IRMA, LAZYPIPE, metaSPAdes, PEHaplo, TRACESPipe, TRACESPipeLite, and ViSpA were able to reconstruct all datasets. In contrast, Haploflow, metaviralSPAdes, QuRe, and SPAdes often failed at reconstructing genomes in low-depth coverage scenarios, and QVG did not output a result if no SNPs were added to the dataset.

The identity measures the correctness of the parts of the genome that could be aligned to the viral genomes contained in the datasets. Low identity values indicate that the parts of the genomes that could be aligned did not correspond to the reference, and a value of zero means that no part of the genome could be aligned. In this metric, all reconstruction tools except for metaviralSPAdes and TRACESPipelite obtained an average performance above 90%. TRACESPipeLite is negatively impacted in this metric because although it is able to reconstruct parts of every dataset, for some, it is only able to reconstruct mitochondrial DNA, which is not considered in this study, obtaining an identity of zero. The same phenomenon happened to metaviralSPAdes, although the number of datasets reconstructed by this pipeline is lower.

The NCSD evaluates the dissimilarity of the reconstructed genomes against a reference. The programs that obtained the best results overall were SPAdes, coronaSPAdes, TRACESPipe, and metaSPAdes, all of which obtained an average value of NCSD below 0.15. Conversely, QuRe obtained the least favorable performance, with an NCSD value nearing 1, which indicates that it did not reconstruct much data from the datasets or that the data were reconstructed incorrectly. It should be noted that the average was made by dividing the sum of all NCSD values obtained by the number of datasets reconstructed by each tool, which penalizes tools that can reconstruct more datasets, albeit incorrectly, or that only reconstructed parts of the genome are not being taken into consideration, namely, mitochondrial DNA or contamination.

The NRC corroborates the findings from the NCSD, as the best performances were obtained by SPAdes, coronaSPAdes, TRACESPipe, and metaSPAdes. Similar to the NCSD, QuRe obtained the lowest performance in this metric, with an average performance close to 1.

The overall number of bases reconstructed (excluding gaps) is a metric that should not be considered by itself, as some programs may reconstruct bases that do not belong to viral genomes or may reconstruct the quasispecies spectrum, outputting several different genomic sequences for a single virus. Hence, the number of scaffolds generated should also be taken into consideration, as tools that reconstruct many bases and output an appropriate number of scaffolds (taking into consideration the number of viruses in the sample) correctly identified the genomes and were able to reconstruct them in 1 piece. Although ViSpA, PEHaplo, QVG, SPAdes, coronaSPAdes, metaSPAdes, LAZYPIPE, Haploflow, TRACESPipe, and IRMA reconstructed on average over 140,000 bp per dataset, out of those, only IRMA, QVG, and TRACESPipe produced a suitable number of scaffolds (about 4 to 6), considering the number of viral genomes contained in the datasets. We observed that QVG, TRACESPipe, TRACESPipeLite, and V-pipe had the most discrepancies between the number of bases considering and disregarding gaps, which indicates that these tools output scaffolds with the most gaps.

In terms of the average length of each scaffold (excluding gaps), VirGenA, PEHaplo, QuRe, and SSAKE obtained a lower performance, each reconstructing scaffolds with, on average, less than 2,000 bp. On the other hand, the programs metaviralSPAdes, QVG, TRACESPipe, and ViSpA were capable of outputting scaffolds with over 35,000 bp, on average.

Regarding the ratio of SNPs in relation to the number of bases reconstructed, QVG obtained the least favorable performances out of the programs considered, obtaining results over 0.02. Although this metric may perform ambiguous analyses, as it is based on alignments, it can be used as an indicator of the degree of reliance on the reference genomes for reconstruction programs that follow RB or HB methodologies.

The time needed for a program to reconstruct a dataset was, on average, less than 180 seconds. On average, metaviralSPAdes, SPAdes, SSAKE, LAZYPIPE, Haploflow, and coronaSPAdes required under 10 seconds to reconstruct a dataset, whereas QuRe and VirGenA required over 700 seconds.

We analyzed the CPU usage and found that VirGenA and QuRe were the most resource-intensive tools, each requiring over 5 execution cores on average to reconstruct a dataset. On the other hand, SSAKE, Haploflow, and ViSpA required the least amount of resources, requiring, on average, less than 1 execution core.

Regarding the maximum RAM used by the tools, QuRe, TRACESPipe, and ViSpA utilized the most resources, each requiring, on average, over 6 GB to reconstruct a dataset, while coronaSPAdes, V-pipe, Haploflow, and SSAKE required the least resources, each using under 0.15 GB of RAM.

To analyze the computational resources utilized by each reconstruction program taking into consideration the reconstruction performance, the metric *P* was introduced. The metric *P* is defined as


(4) \begin{eqnarray*}
P = \bar{m} * \bar{n},
\end{eqnarray*}

where $\bar{m}$ represents the average value obtained by the reconstruction program in the metric chosen (the execution time, RAM usage, or CPU usage), and $\bar{n}$ represents the average value of the NCSD for the reconstruction program considered. In this case, the NCSD acts as an attenuating factor to the metric chosen. The values of the *P* range from slightly above 0 to infinity, with lower values indicating a better performance.

Haploflow, LAZYPIPE, SPAdes, and coronaSPAdes performed the best in terms of the weighted performance of the time, achieving a value under 2. On the other hand, the tools QuRe and VirGenA had a lower performance, both obtaining values over 400.

Regarding the weighted performance of the CPU, Haploflow, QVG, SPAdes, ViSpA, and PEHaplo achieved the best performances, obtaining values under 30. Conversely, QuRe and VirGenA had less-than-ideal performances, obtaining values over 400.

Lastly, in terms of the weighted performance of the RAM, SSAKE, V-pipe, Haploflow, metaSPAdes, coronaSPAdes, and SPAdes demonstrated the best performance, with values under 0.1, while QuRe had the least satisfactory performance in this metric, obtaining a value over 20.

Comparing the amount of computational resources utilized by reconstruction programs belonging to a reconstruction methodology (RF, RB, or HB), we observed that the programs that followed the RF methodology were overall the most efficient. The programs following the HB methodology were the second most efficient in terms of the execution time and RAM usage, while programs using the RB methodology were the second most efficient with regard to CPU usage. These findings seem to contradict the description of the methodologies provided in the “Introduction” section, but it must be taken into consideration that some programs—namely, the QuRe and VirGenA—tend to consume considerably more resources than the remaining and that some tools provide additional outputs, which may have affected the results obtained.

## Performance in Real Datasets

In order to measure the performance of each reconstruction program in real-life scenarios, 6 real datasets were considered. In these scenarios, there is no detailed information on the contents of the sample, and therefore, in addition to the reconstruction process, it was necessary to classify the datasets using FALCON-meta [73]. FALCON-meta provides information on the viruses present in the sample and predicts which are the most suitable references to be used in the reconstruction process. The references were extracted from a viral database, included in the benchmark, and are used by programs that follow the RB and HB methodologies and require references to be provided by the user. When analyzing these datasets with a top-similarity value of 8,000, FALCON-meta found a minimum of 26 and a maximum of 36 suitable references per dataset, averaging 30.8. The viruses found in each dataset can be found in Supplementary Table S151.

To assess the performance of the reconstruction programs in these datasets, the metrics identity, NCSD, NRC, and the ratio of SNPs in relation to the number of bases reconstructed could not be used, as these metrics require the true composition of the datasets to be known (requires a gold standard). Hence, the evaluation was based on other metrics available—namely, the number of datasets reconstructed; the number of bases reconstructed; the number of scaffolds generated; the minimum, maximum, and average length of the scaffolds; and the computational resources used by each of the programs.

**Figure 10:**
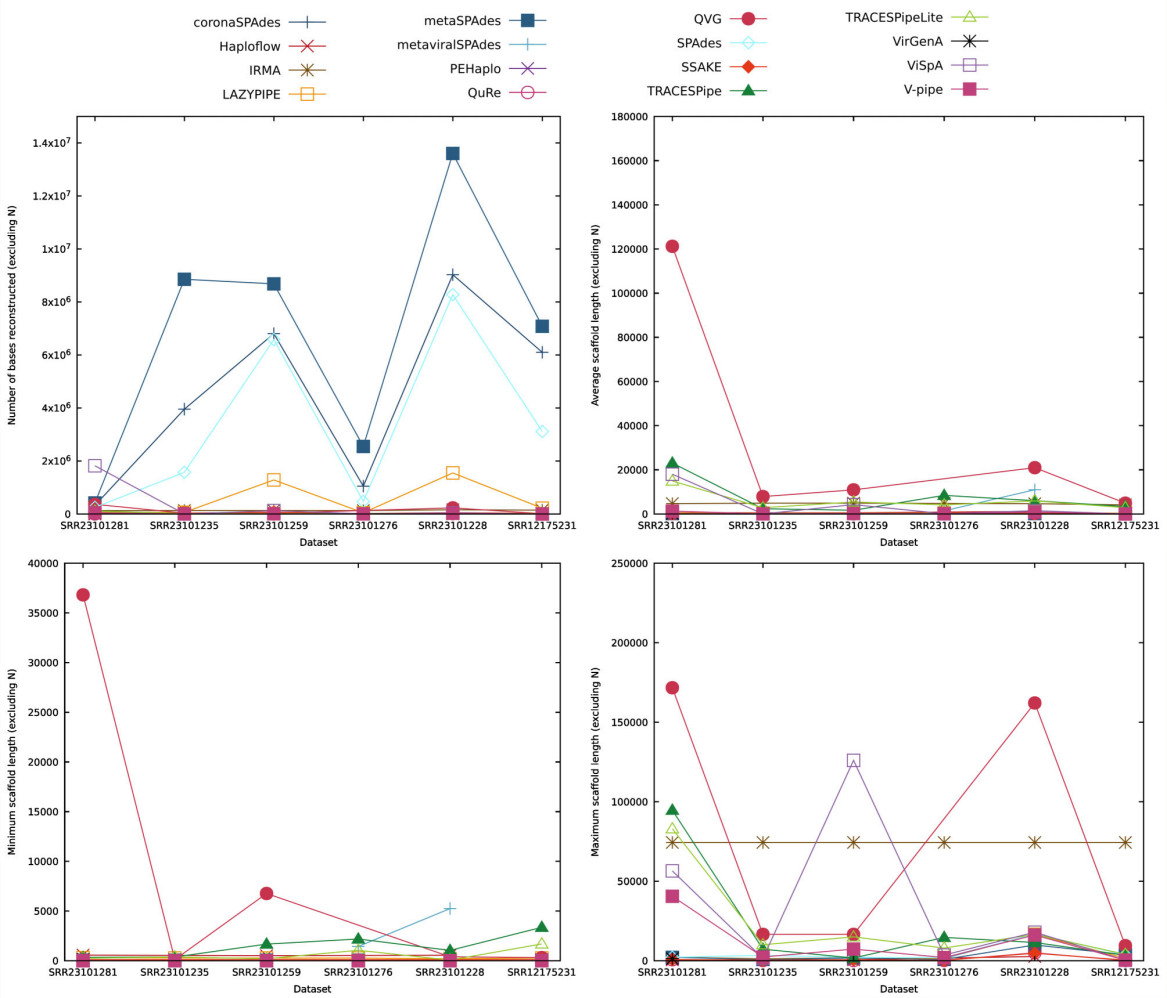
Comparison of the performance of the reconstruction programs according to the number of reconstructed bases and the average, minimum, and maximum number of bases per scaffold generated, excluding non-reconstructed bases (N) in real datasets. For these metrics, higher values indicate a better performance.

Some of the results obtained using these datasets are available in Fig. 10, while the remaining are in the Supplementary Material.

All of the reconstruction programs, except for PEHaplo, were able to reconstruct at least 1 of the real datasets provided. Additionally, coronaSPAdes, IRMA, LAZYPIPE, metaSPAdes, QuRe, SPAdes, SSAKE, TRACESPipe, TRACESPipeLite, V-pipe, and ViSpA were able to output genome sequences for all datasets provided.

Regarding the number of bases reconstructed (excluding gaps), metaSPAdes output the most bases in every dataset considered, except SRR23101281, where the best performance was obtained by ViSpA. In these datasets, metaSPAdes, coronaSPAdes, and SPAdes reconstructed the most bases, outputting, on average, over 3,000,000 bp per dataset.

The average length of the scaffolds was especially great for the tool QVG, which obtained a better performance in datasets SRR23101281, SRR23101235, SRR23101259, SRR23101228, and SRR12175231. The dataset SRR23101276 was not reconstructed by QVG, and the best performance was obtained by TRACESPipe. TRACESPipe, metaviralSPAdes, TRACESPipeLite, and QVG had the best performances in this metric and output on average over 5,000 bp per scaffold. Conversely, V-pipe, LAZYPIPE, VirGenA, SPAdes, coronaSPAdes, SSAKE, metaSPAdes, and QuRe obtained a lower performance in this metric, outputting scaffolds with an average length under 500 bp.

In terms of the minimum length of the scaffolds, QVG performed better than the remaining programs in datasets SRR23101281 and SRR23101259. In dataset SRR23101235, the best performance was obtained by both TRACESPipe and TRACESPipeLite. TRACESPipe also obtained the best performances in datasets SRR23101276 and SRR12175231. Additionally, metaviralSPAdes obtained the best results in this metric in dataset SRR23101228. Overall, the best reconstruction programs regarding this metric were QVG, TRACESPipe, and metaviralSPAdes, with a minimum scaffold length above 1,000 bp, on average.

Regarding the maximum length of the scaffolds, QVG obtained the best results in datasets SRR23101281 and SRR23101228; IRMA had the best performance in datasets SRR23101235, SRR23101276, and SRR12175231; and ViSpA had the best performance in dataset SRR23101259. Overall, the programs that obtained the best performances in this metric were QVG, IRMA, and ViSpA, whose maximum length scaffolds had, on average, over 30,000 bp.

Using real datasets without a gold standard, it is complex to accurately determine how many different viral genomes are contained in the dataset, and as such, the exact number of scaffolds that should have been generated by each program is unknown. On average, the reconstruction programs output about 4,870 scaffolds per dataset, with coronaSPAdes, LAZYPIPE, metaSPAdes, and SPAdes outputting an average of over 10,000 scaffolds per dataset, while the remaining programs generated, on average, fewer than 1,500 scaffolds per dataset.

Regarding the reconstruction time, the most efficient programs were coronaSPAdes, Haploflow, TRACESPipeLite, and LAZYPIPE, which reconstructed the datasets in under 2 minutes, on average, and the least efficient programs were QuRe and VirGenA, which required, on average, over 4.5 hours to reconstruct a dataset.

In terms of the CPU usage, Haploflow and SSAKE required, on average, less than a core to execute, while VirGenA, QuRe, and IRMA spent the most resources, using on average over 6 execution cores.

Regarding the maximum RAM used, SSAKE, IRMA, and V-pipe were the most efficient tools, using less than 1 GB of RAM, whereas the most resource-intensive tool in this metric was QuRe, requiring, on average, over 34 GB of maximum RAM.

## Discussion

Every day, the number of genomic samples sequenced is rising, which makes the reconstruction of the sequenced genomes and the evaluation of the programs that reconstruct them increasingly important.

Throughout the synthetic datasets analyzed, we observed that the addition of contamination and mitochondrial DNA can have a negative effect on the performance of the reconstruction programs. We also observed that the performance of the reconstruction programs generally deteriorated at low coverage and at higher percentages of SNPs in the sample. Furthermore, we showed that different values of read length and different viral compositions can affect the performance of the reconstruction programs.

Regarding the metrics considered, the identity was a good indicator of whether or not a dataset was reconstructed. However, the NCSD and NRC were better at describing the performance of the reconstruction programs. We found that the NCSD and NRC were inherently linked, as their performance is highly consistent with each other, and both rely on compression. Additionally, we found that the number of reconstructed bases is more informative when combined with the number of scaffolds reconstructed, as that provides information about not only the quantity of genome that was reconstructed but also the degree of fragmentation of the reconstructed genome.

In terms of the computational resources, it was estimated that RB methods were the most efficient, followed by RF methods and, lastly, HB methods. In reality, on average, tools that followed the RF methodology were the most efficient in terms of execution time, maximum RAM usage, and CPU usage. Pipelines based on the HB methodology achieved the second-best results in terms of execution time and maximum RAM usage, while the programs using the RB methodology achieved the second-best results for CPU usage and the least favorable results in the remaining 2 metrics. This may be due to other tasks that the programs that follow the RB and HB methodologies perform, namely, analyzing and plotting the results obtained.

Employing authentic datasets facilitated the comparison of the performance of the reconstruction programs within real-life scenarios. However, it is crucial to note the absence of a definitive gold standard in this context, rendering the findings merely indicative. A potential method to benchmark such datasets involves synthesizing a virus, integrating it into a sample, and subsequently subjecting it to sequencing and computational analysis. This approach would yield the complete viral sequence, yet it is essential to acknowledge that sequencing processes may introduce errors. Analysis pipelines might interpret these errors as variants unless guided by explicit instructions to handle low-quality sequencing data.

While it can be argued that high coverage depth sequencing minimizes uncertainties, challenges persist at smaller depth coverages. Even with the proposed methodology, complete certainty in the benchmarks for real datasets remains elusive.

Existing pipelines and tools rely on rigid or adaptable parameters, which can significantly impact their accuracy. For instance, employing an aligner with high sensitivity differs markedly from one with lower sensitivity, thereby influencing the quality of the reconstructed sequence. Throughout this review, default parameters or settings conducive to reasonable processing times were predominantly employed. Regrettably, benchmarking these pipelines under various parameter sets becomes impractical without a substantial increase in computational resources. Such an expansion could easily reach an order of magnitude when exploring diverse parameter combinations. It is imperative to comprehend that altering the parameters of these tools and pipelines can markedly influence the accuracy and, consequently, the outcomes presented in this article.

Considering the reconstruction programs and the parameters used, there is no reconstruction program that is better than the remaining for all different scenarios and across all metrics.

When the datasets had low coverage and a low percentage of SNPs, the programs TRACESPipe and QVG obtained better performances in terms of the correctness of the reconstruction (NCSD and NRC) and the length of the scaffolds produced, although QVG may be overrelying on the reference genomes provided. With a low-depth coverage and a high percentage of SNPs, metaSPAdes had better performance according to the NCSD and the NRC, but it output significantly shorter scaffolds.

Using datasets with a higher depth coverage (at least 5×), the reconstruction programs SPAdes, coronaSPAdes, LAZYPIPE, and TRACESPipe should be considered, as they had better performances, considering the correctness of the genome. In terms of the fragmentation of the genome output, TRACESPipe reconstructed the expected number of scaffolds (4), whereas the remaining tools output considerably more scaffolds, especially with depth coverages of 5× and 10×.

If there is a preference for tools that output long scaffolds, metaviralSPAdes, QVG, and TRACESPipe should be considered, as they reconstructed few scaffolds with significant average length. It should be noted that although metaviralSPAdes output long scaffolds, the tool reconstructed only some of the datasets considered.

If the execution time is a priority, the tools coronaSPAdes and LAZYPIPE should be considered, as they have reconstructed at least part of each dataset, had a low average reconstruction time in both real and synthetic datasets, and obtained some of the best values in terms of the the weighted time performance.

In cases where computational resources are limited, Haploflow, SSAKE, and V-pipe should be considered as they had the lowest CPU and RAM requirements.

We have successfully surveyed reconstruction programs that focused on the reconstruction of viral genomes, summarized their methodology, and highlighted their characteristics. Additionally, we created a publicly available benchmark capable of automatically installing all programs necessary for its execution, as well as reconstructing viral genomes and evaluating the performance of each tool for datasets with and without a gold standard. Furthermore, we provide scripts capable of generating synthetic datasets and retrieving real datasets from the NCBI. Using the benchmark, the tools were executed using both real and synthetic datasets, and the results were provided and described according to each characteristics tested. Lastly, we compared the performances of the reconstruction programs as a whole and provided recommendations about what tools should be used in given scenarios.

## Conclusions

Although the fast and accurate reconstruction of human viral genomes is fundamental for biological, medical, and forensic applications, identifying the best assembly tool is challenging.

In this article, we surveyed some of the existent viral genome reconstruction methods and identified some features, similarities, and dissimilarities between these tools. Moreover, we provided a reconstruction benchmark and evaluated the reconstruction process in 73 synthetic datasets and 6 real datasets.

The reconstruction programs were evaluated based on the correctness of the reconstruction process, using the metrics identity, NCSD, NRC, number of SNPs, and the ratio of SNPs in relation to the number of bases reconstructed. Also, some metrics were used to evaluate the genomes and scaffolds reconstructed—namely, the number of bases reconstructed, the number of scaffolds output, and the minimum, maximum, and average number of bases per scaffold. Lastly, the execution time and computational resources (RAM and CPU usage) needed to execute the reconstruction programs were evaluated using both weighted and unweighted methods. This methodology is publicly available and flexible to the augmentation of search engines to increase the number of programs being considered. Moreover, it is possible to include other datasets so that the performance of each program can be tested under different conditions.

## Availability of Source Code and Requirements

- Project name: HVRS—Human Viral Reconstruction Survey
- Project homepage: https://github.com/viromelab/HVRS/
- Operating system(s): Linux
- Programming language: Shell
- License: GNU GPL3

## Supplementary Material

giaf159_Supplemental_File

giaf159_Authors_Response_To_Reviewer_Comments_original_submission

giaf159_Authors_Response_To_Reviewer_Comments_revision_1

giaf159_GIGA-D-25-00031_original_submission

giaf159_GIGA-D-25-00031_Revision_1

giaf159_GIGA-D-25-00031_Revision_2

giaf159_Reviewer_1_Report_original_submission Levente Laczkó -- 2/23/2025

giaf159_Reviewer_1_Report_revision_1 Levente Laczkó -- 9/12/2025

giaf159_Reviewer_2_Report_original_submission Anton Korobeynikov, Ph.D -- 4/23/2025

giaf159_Reviewer_3_Report_original_submission Serghei Mangul -- 4/23/2025

## Data Availability

All additional supporting data are available in the *GigaScience* repository, GigaDB [154].

## Additional Files

**Supplementary Fig. S1**. Figure comparing the performance of the reconstruction programs in terms of the number of bases reconstructed for datasets without contamination or mitochondrial DNA (DS1 to DS8).

**Supplementary Fig. S2**. Figure comparing the performance of the reconstruction programs in terms of the number of bases reconstructed (excluding “N”) for datasets without contamination or mitochondrial DNA (DS1 to DS8).

**Supplementary Fig. S3**. Figure comparing the performance of the reconstruction programs in terms of the minimum number of reconstructed bases per scaffold for datasets without contamination or mitochondrial DNA (DS1 to DS8).

**Supplementary Fig. S4**. Figure comparing the performance of the reconstruction programs in terms of the minimum number of reconstructed bases per scaffold (excluding “N”) for datasets without contamination or mitochondrial DNA (DS1 to DS8).

**Supplementary Fig. S5**. Figure comparing the performance of the reconstruction programs in terms of the maximum number of reconstructed bases per scaffold for datasets without contamination or mitochondrial DNA (DS1 to DS8).

**Supplementary Fig. S6**. Figure comparing the performance of the reconstruction programs in terms of the maximum number of reconstructed bases per scaffold (excluding “N”) for datasets without contamination or mitochondrial DNA (DS1 to DS8).

**Supplementary Fig. S7**. Figure comparing the performance of the reconstruction programs in terms of the average number of reconstructed bases per scaffold for datasets without contamination or mitochondrial DNA (DS1 to DS8).

**Supplementary Fig. S8**. Figure comparing the performance of the reconstruction programs in terms of the average number of reconstructed bases per scaffold (excluding “N”) for datasets without contamination or mitochondrial DNA (DS1 to DS8).

**Supplementary Fig. S9**. Figure comparing the performance of the reconstruction programs in terms of the ratio between the number of SNPs and the number of bases reconstructed for datasets without contamination or mitochondrial DNA (DS1 to DS8).

**Supplementary Fig. S10**. Figure comparing the performance of the reconstruction programs in terms of the ratio between the number of SNPs and the number of bases reconstructed (excluding “N”) for datasets without contamination or mitochondrial DNA (DS1 to DS8).

**Supplementary Fig. S11**. Figure comparing the performance of the reconstruction programs in terms of the number of bases reconstructed for datasets with contamination and mitochondrial DNA (DS9 to DS16).

**Supplementary Fig. S12**. Figure comparing the performance of the reconstruction programs in terms of the number of bases reconstructed (excluding “N”) for datasets with contamination and mitochondrial DNA (DS9 to DS16).

**Supplementary Fig. S13**. Figure comparing the performance of the reconstruction programs in terms of the minimum number of reconstructed bases per scaffold for datasets with contamination and mitochondrial DNA (DS9 to DS16).

**Supplementary Fig. S14**. Figure comparing the performance of the reconstruction programs in terms of the minimum number of reconstructed bases per scaffold (excluding “N”) for datasets with contamination and mitochondrial DNA (DS9 to DS16).

**Supplementary Fig. S15**. Figure comparing the performance of the reconstruction programs in terms of the maximum number of reconstructed bases per scaffold for datasets with contamination and mitochondrial DNA (DS9 to DS16).

**Supplementary Fig. S16**. Figure comparing the performance of the reconstruction programs in terms of the maximum number of reconstructed bases per scaffold (excluding “N”) for datasets with contamination and mitochondrial DNA (DS9 to DS16).

**Supplementary Fig. S17**. Figure comparing the performance of the reconstruction programs in terms of the average number of reconstructed bases per scaffold for datasets with contamination and mitochondrial DNA (DS9 to DS16).

**Supplementary Fig. S18**. Figure comparing the performance of the reconstruction programs in terms of the average number of reconstructed bases per scaffold (excluding “N”) for datasets with contamination and mitochondrial DNA (DS9 to DS16).

**Supplementary Fig. S19**. Figure comparing the performance of the reconstruction programs in terms of the ratio between the number of SNPs and the number of bases reconstructed for datasets with contamination and mitochondrial DNA (DS9 to DS16).

**Supplementary Fig. S20**. Figure comparing the performance of the reconstruction programs in terms of the ratio between the number of SNPs and the number of bases reconstructed (excluding “N”) for datasets with contamination and mitochondrial DNA (DS9 to DS16).

**Supplementary Fig. S21**. Figure comparing the performance of the reconstruction programs in terms of the number of bases reconstructed for datasets with depth coverage equal to 2× (DS17 to DS24 plus DS9).

**Supplementary Fig. S22**. Figure comparing the performance of the reconstruction programs in terms of the number of bases reconstructed (excluding “N”) for datasets with depth coverage equal to 2× (DS17 to DS24 plus DS9).

**Supplementary Fig. S23**. Figure comparing the performance of the reconstruction programs in terms of the minimum number of reconstructed bases per scaffold for datasets with depth coverage equal to 2× (DS17 to DS24 plus DS9).

**Supplementary Fig. S24**. Figure comparing the performance of the reconstruction programs in terms of the minimum number of reconstructed bases per scaffold (excluding “N”) for datasets with depth coverage equal to 2× (DS17 to DS24 plus DS9).

**Supplementary Fig. S25**. Figure comparing the performance of the reconstruction programs in terms of the maximum number of reconstructed bases per scaffold for datasets with depth coverage equal to 2× (DS17 to DS24 plus DS9).

**Supplementary Fig. S26**. Figure comparing the performance of the reconstruction programs in terms of the maximum number of reconstructed bases per scaffold (excluding “N”) for datasets with depth coverage equal to 2× (DS17 to DS24 plus DS9).

**Supplementary Fig. S27**. Figure comparing the performance of the reconstruction programs in terms of the average number of reconstructed bases per scaffold for datasets with depth coverage equal to 2× (DS17 to DS24 plus DS9).

**Supplementary Fig. S28**. Figure comparing the performance of the reconstruction programs in terms of the average number of reconstructed bases per scaffold (excluding “N”) for datasets with depth coverage equal to 2× (DS17 to DS24 plus DS9).

**Supplementary Fig. S29**. Figure comparing the performance of the reconstruction programs in terms of the ratio between the number of SNPs and the number of bases reconstructed for datasets with depth coverage equal to 2× (DS17 to DS24 plus DS9).

**Supplementary Fig. S30**. Figure comparing the performance of the reconstruction programs in terms of the ratio between the number of SNPs and the number of bases reconstructed (excluding “N”) for datasets with depth coverage equal to 2× (DS17 to DS24 plus DS9).

**Supplementary Fig. S31**. Figure comparing the performance of the reconstruction programs in terms of the identity for datasets with depth coverage equal to 5× (DS25 to DS32 plus DS10).

**Supplementary Fig. S32**. Figure comparing the performance of the reconstruction programs in terms of the NCSD for datasets with depth coverage equal to 5× (DS25 to DS32 plus DS10).

**Supplementary Fig. S33**. Figure comparing the performance of the reconstruction programs in terms of the NRC for datasets with depth coverage equal to 5× (DS25 to DS32 plus DS10).

**Supplementary Fig. S34**. Figure comparing the performance of the reconstruction programs in terms of the number of bases reconstructed for datasets with depth coverage equal to 5× (DS25 to DS32 plus DS10).

**Supplementary Fig. S35**. Figure comparing the performance of the reconstruction programs in terms of the number of bases reconstructed (excluding “N”) for datasets with depth coverage equal to 5× (DS25 to DS32 plus DS10).

**Supplementary Fig. S36**. Figure comparing the performance of the reconstruction programs in terms of the minimum number of reconstructed bases per scaffold for datasets with depth coverage equal to 5× (DS25 to DS32 plus DS10).

**Supplementary Fig. S37**. Figure comparing the performance of the reconstruction programs in terms of the minimum number of reconstructed bases per scaffold (excluding “N”) for datasets with depth coverage equal to 5× (DS25 to DS32 plus DS10).

**Supplementary Fig. S38**. Figure comparing the performance of the reconstruction programs in terms of the maximum number of reconstructed bases per scaffold for datasets with depth coverage equal to 5× (DS25 to DS32 plus DS10).

**Supplementary Fig. S39**. Figure comparing the performance of the reconstruction programs in terms of the maximum number of reconstructed bases per scaffold (excluding “N”) for datasets with depth coverage equal to 5× (DS25 to DS32 plus DS10).

**Supplementary Fig. S40**. Figure comparing the performance of the reconstruction programs in terms of the average number of reconstructed bases per scaffold for datasets with depth coverage equal to 5× (DS25 to DS32 plus DS10).

**Supplementary Fig. S41**. Figure comparing the performance of the reconstruction programs in terms of the average number of reconstructed bases per scaffold (excluding “N”) for datasets with depth coverage equal to 5× (DS25 to DS32 plus DS10).

**Supplementary Fig. S42**. Figure comparing the performance of the reconstruction programs in terms of the ratio between the number of SNPs and the number of bases reconstructed for datasets with depth coverage equal to 5× (DS25 to DS32 plus DS10).

**Supplementary Fig. S43**. Figure comparing the performance of the reconstruction programs in terms of the ratio between the number of SNPs and the number of bases reconstructed (excluding “N”) for datasets with depth coverage equal to 5× (DS25 to DS32 plus DS10).

**Supplementary Fig. S44**. Figure comparing the performance of the reconstruction programs in terms of the identity for datasets with depth coverage equal to 10× (DS33 to DS40 plus DS11).

**Supplementary Fig. S45**. Figure comparing the performance of the reconstruction programs in terms of the NCSD for datasets with depth coverage equal to 10× (DS33 to DS40 plus DS11).

**Supplementary Fig. S46**. Figure comparing the performance of the reconstruction programs in terms of the NRC for datasets with depth coverage equal to 10× (DS33 to DS40 plus DS11).

**Supplementary Fig. S47**. Figure comparing the performance of the reconstruction programs in terms of the number of bases reconstructed for datasets with depth coverage equal to 10× (DS33 to DS40 plus DS11).

**Supplementary Fig. S48**. Figure comparing the performance of the reconstruction programs in terms of the number of bases reconstructed (excluding “N”) for datasets with depth coverage equal to 10× (DS33 to DS40 plus DS11).

**Supplementary Fig. S49**. Figure comparing the performance of the reconstruction programs in terms of the minimum number of reconstructed bases per scaffold for datasets with depth coverage equal to 10× (DS33 to DS40 plus DS11).

**Supplementary Fig. S50**. Figure comparing the performance of the reconstruction programs in terms of the minimum number of reconstructed bases per scaffold (excluding “N”) for datasets with depth coverage equal to 10× (DS33 to DS40 plus DS11).

**Supplementary Fig. S51**. Figure comparing the performance of the reconstruction programs in terms of the maximum number of reconstructed bases per scaffold for datasets with depth coverage equal to 10× (DS33 to DS40 plus DS11).

**Supplementary Fig. S52**. Figure comparing the performance of the reconstruction programs in terms of the maximum number of reconstructed bases per scaffold (excluding “N”) for datasets with depth coverage equal to 10× (DS33 to DS40 plus DS11).

**Supplementary Fig. S53**. Figure comparing the performance of the reconstruction programs in terms of the average number of reconstructed bases per scaffold for datasets with depth coverage equal to 10× (DS33 to DS40 plus DS11).

**Supplementary Fig. S54**. Figure comparing the performance of the reconstruction programs in terms of the average number of reconstructed bases per scaffold (excluding “N”) for datasets with depth coverage equal to 10× (DS33 to DS40 plus DS11).

**Supplementary Fig. S55**. Figure comparing the performance of the reconstruction programs in terms of the ratio between the number of SNPs and the number of bases reconstructed for datasets with depth coverage equal to 10× (DS33 to DS40 plus DS11).

**Supplementary Fig. S56**. Figure comparing the performance of the reconstruction programs in terms of the ratio between the number of SNPs and the number of bases reconstructed (excluding “N”) for datasets with depth coverage equal to 10× (DS33 to DS40 plus DS11).

**Supplementary Fig. S57**. Figure comparing the performance of the reconstruction programs in terms of the identity for datasets with depth coverage equal to 20× (DS41 to DS48 plus DS13).

**Supplementary Fig. S58**. Figure comparing the performance of the reconstruction programs in terms of the NCSD for datasets with depth coverage equal to 20× (DS41 to DS48 plus DS13).

**Supplementary Fig. S59**. Figure comparing the performance of the reconstruction programs in terms of the NRC for datasets with depth coverage equal to 20× (DS41 to DS48 plus DS13).

**Supplementary Fig. S60**. Figure comparing the performance of the reconstruction programs in terms of the number of bases reconstructed for datasets with depth coverage equal to 20× (DS41 to DS48 plus DS13).

**Supplementary Fig. S61**. Figure comparing the performance of the reconstruction programs in terms of the number of bases reconstructed (excluding “N”) for datasets with depth coverage equal to 20× (DS41 to DS48 plus DS13).

**Supplementary Fig. S62**. Figure comparing the performance of the reconstruction programs in terms of the minimum number of reconstructed bases per scaffold for datasets with depth coverage equal to 20× (DS41 to DS48 plus DS13).

**Supplementary Fig. S63**. Figure comparing the performance of the reconstruction programs in terms of the minimum number of reconstructed bases per scaffold (excluding “N”) for datasets with depth coverage equal to 20× (DS41 to DS48 plus DS13).

**Supplementary Fig. S64**. Figure comparing the performance of the reconstruction programs in terms of the maximum number of reconstructed bases per scaffold for datasets with depth coverage equal to 20× (DS41 to DS48 plus DS13).

**Supplementary Fig. S65**. Figure comparing the performance of the reconstruction programs in terms of the maximum number of reconstructed bases per scaffold (excluding “N”) for datasets with depth coverage equal to 20× (DS41 to DS48 plus DS13).

**Supplementary Fig. S66**. Figure comparing the performance of the reconstruction programs in terms of the average number of reconstructed bases per scaffold for datasets with depth coverage equal to 20× (DS41 to DS48 plus DS13).

**Supplementary Fig. S67**. Figure comparing the performance of the reconstruction programs in terms of the average number of reconstructed bases per scaffold (excluding “N”) for datasets with depth coverage equal to 20× (DS41 to DS48 plus DS13).

**Supplementary Fig. S68**. Figure comparing the performance of the reconstruction programs in terms of the ratio between the number of SNPs and the number of bases reconstructed for datasets with depth coverage equal to 20× (DS41 to DS48 plus DS13).

**Supplementary Fig. S69**. Figure comparing the performance of the reconstruction programs in terms of the ratio between the number of SNPs and the number of bases reconstructed (excluding “N”) for datasets with depth coverage equal to 20× (DS41 to DS48 plus DS13).

**Supplementary Fig. S70**. Figure comparing the performance of the reconstruction programs in terms of the number of bases reconstructed for datasets with depth coverage equal to 40× (DS49 to DS56 plus DS16).

**Supplementary Fig. S71**. Figure comparing the performance of the reconstruction programs in terms of the number of bases reconstructed (excluding “N”) for datasets with depth coverage equal to 40× (DS49 to DS56 plus DS16).

**Supplementary Fig. S72**. Figure comparing the performance of the reconstruction programs in terms of the minimum number of reconstructed bases per scaffold for datasets with depth coverage equal to 40× (DS49 to DS56 plus DS16).

**Supplementary Fig. S73**. Figure comparing the performance of the reconstruction programs in terms of the minimum number of reconstructed bases per scaffold (excluding “N”) for datasets with depth coverage equal to 40× (DS49 to DS56 plus DS16).

**Supplementary Fig. S74**. Figure comparing the performance of the reconstruction programs in terms of the maximum number of reconstructed bases per scaffold for datasets with depth coverage equal to 40× (DS49 to DS56 plus DS16).

**Supplementary Fig. S75**. Figure comparing the performance of the reconstruction programs in terms of the maximum number of reconstructed bases per scaffold (excluding “N”) for datasets with depth coverage equal to 40× (DS49 to DS56 plus DS16).

**Supplementary Fig. S76**. Figure comparing the performance of the reconstruction programs in terms of the average number of reconstructed bases per scaffold for datasets with depth coverage equal to 40× (DS49 to DS56 plus DS16).

**Supplementary Fig. S77**. Figure comparing the performance of the reconstruction programs in terms of the average number of reconstructed bases per scaffold (excluding “N”) for datasets with depth coverage equal to 40× (DS49 to DS56 plus DS16).

**Supplementary Fig. S78**. Figure comparing the performance of the reconstruction programs in terms of the ratio between the number of SNPs and the number of bases reconstructed for datasets with depth coverage equal to 40× (DS49 to DS56 plus DS16).

**Supplementary Fig. S79**. Figure comparing the performance of the reconstruction programs in terms of the ratio between the number of SNPs and the number of bases reconstructed (excluding “N”) for datasets with depth coverage equal to 40× (DS49 to DS56 plus DS16).

**Supplementary Fig. S80**. Figure comparing the performance of the reconstruction programs in terms of the number of bases reconstructed for datasets with different viral compositions (datasets 13, 63, 64, and 65).

**Supplementary Fig. S81**. Figure comparing the performance of the reconstruction programs in terms of the number of bases reconstructed (excluding “N”) for datasets with different viral compositions (datasets 13, 63, 64, and 65).

**Supplementary Fig. S82**. Figure comparing the performance of the reconstruction programs in terms of the minimum number of reconstructed bases per scaffold for datasets with different viral compositions (datasets 13, 63, 64, and 65).

**Supplementary Fig. S83**. Figure comparing the performance of the reconstruction programs in terms of the minimum number of reconstructed bases per scaffold (excluding “N”) for datasets with different viral compositions (datasets 13, 63, 64, and 65).

**Supplementary Fig. S84**. Figure comparing the performance of the reconstruction programs in terms of the maximum number of reconstructed bases per scaffold for datasets with different viral compositions (datasets 13, 63, 64, and 65).

**Supplementary Fig. S85**. Figure comparing the performance of the reconstruction programs in terms of the maximum number of reconstructed bases per scaffold (excluding “N”) for datasets with different viral compositions (datasets 13, 63, 64, and 65).

**Supplementary Fig. S86**. Figure comparing the performance of the reconstruction programs in terms of the average number of reconstructed bases per scaffold for datasets with different viral compositions (datasets 13, 63, 64, and 65).

**Supplementary Fig. S87**. Figure comparing the performance of the reconstruction programs in terms of the average number of reconstructed bases per scaffold (excluding “N”) for datasets with different viral compositions (datasets 13, 63, 64, and 65).

**Supplementary Fig. S88**. Figure comparing the performance of the reconstruction programs in terms of the ratio between the number of SNPs and the number of bases reconstructed for datasets with different viral compositions (datasets 13, 63, 64, and 65).

**Supplementary Fig. S89**. Figure comparing the performance of the reconstruction programs in terms of the ratio between the number of SNPs and the number of bases reconstructed (excluding “N”) for datasets with different viral compositions (datasets 13, 63, 64, and 65).

**Supplementary Fig. S90**. Figure comparing the performance of the reconstruction programs in terms of the number of bases reconstructed for datasets 5, 13, 57, and 58.

**Supplementary Fig. S91**. Figure comparing the performance of the reconstruction programs in terms of the number of bases reconstructed (excluding “N”) for datasets 5, 13, 57, and 58.

**Supplementary Fig. S92**. Figure comparing the performance of the reconstruction programs in terms of the minimum number of reconstructed bases per scaffold for datasets 5, 13, 57, and 58.

**Supplementary Fig. S93**. Figure comparing the performance of the reconstruction programs in terms of the minimum number of reconstructed bases per scaffold (excluding “N”) for datasets 5, 13, 57, and 58.

**Supplementary Fig. S94**. Figure comparing the performance of the reconstruction programs in terms of the maximum number of reconstructed bases per scaffold for datasets 5, 13, 57, and 58.

**Supplementary Fig. S95**. Figure comparing the performance of the reconstruction programs in terms of the maximum number of reconstructed bases per scaffold (excluding “N”) for datasets 5, 13, 57, and 58.

**Supplementary Fig. S96**. Figure comparing the performance of the reconstruction programs in terms of the average number of reconstructed bases per scaffold for datasets 5, 13, 57, and 58.

**Supplementary Fig. S97**. Figure comparing the performance of the reconstruction programs in terms of the average number of reconstructed bases per scaffold (excluding “N”) for datasets 5, 13, 57, and 58.

**Supplementary Fig. S98**. Figure comparing the performance of the reconstruction programs in terms of the ratio between the number of SNPs and the number of bases reconstructed for datasets 5, 13, 57, and 58.

**Supplementary Fig. S99**. Figure comparing the performance of the reconstruction programs in terms of the ratio between the number of SNPs and the number of bases reconstructed (excluding “N”) for datasets 5, 13, 57, and 58.

**Supplementary Fig. S100**. Figure comparing the performance of the reconstruction programs in terms of the number of bases reconstructed for datasets with different read lengths (datasets 61, 13, and 62).

**Supplementary Fig. S101**. Figure comparing the performance of the reconstruction programs in terms of the number of bases reconstructed (excluding “N”) for datasets with different read lengths (datasets 61, 13, and 62).

**Supplementary Fig. S102**. Figure comparing the performance of the reconstruction programs in terms of the minimum number of reconstructed bases per scaffold for datasets with different read lengths (datasets 61, 13, and 62).

**Supplementary Fig. S103**. Figure comparing the performance of the reconstruction programs in terms of the minimum number of reconstructed bases per scaffold (excluding “N”) for datasets with different read lengths (datasets 61, 13, and 62).

**Supplementary Fig. S104**. Figure comparing the performance of the reconstruction programs in terms of the maximum number of reconstructed bases per scaffold for datasets with different read lengths (datasets 61, 13, and 62).

**Supplementary Fig. S105**. Figure comparing the performance of the reconstruction programs in terms of the maximum number of reconstructed bases per scaffold (excluding “N”) for datasets with different read lengths (datasets 61, 13, and 62).

**Supplementary Fig. S106**. Figure comparing the performance of the reconstruction programs in terms of the average number of reconstructed bases per scaffold for datasets with different read lengths (datasets 61, 13, and 62).

**Supplementary Fig. S107**. Figure comparing the performance of the reconstruction programs in terms of the average number of reconstructed bases per scaffold (excluding “N”) for datasets with different read lengths (datasets 61, 13, and 62).

**Supplementary Fig. S108**. Figure comparing the performance of the reconstruction programs in terms of the ratio between the number of SNPs and the number of bases reconstructed for datasets with different read lengths (datasets 61, 13, and 62).

**Supplementary Fig. S109**. Figure comparing the performance of the reconstruction programs in terms of the ratio between the number of SNPs and the number of bases reconstructed (excluding “N”) for datasets with different read lengths (datasets 61, 13, and 62).

**Supplementary Fig. S110**. Figure comparing the performance of the reconstruction programs in terms of the number of bases reconstructed for datasets with error rates between 0.0 and 0.05 and depth coverage equal to 5× (DS66 to DS69).

**Supplementary Fig. S111**. Figure comparing the performance of the reconstruction programs in terms of the number of bases reconstructed (excluding “N”) for datasets with error rates between 0.0 and 0.05 and depth coverage equal to 5× (DS66 to DS69).

**Supplementary Fig. S112**. Figure comparing the performance of the reconstruction programs in terms of the minimum number of reconstructed bases per scaffold for datasets with error rates between 0.0 and 0.05 and depth coverage equal to 5× (DS66 to DS69).

**Supplementary Fig. S113**. Figure comparing the performance of the reconstruction programs in terms of the minimum number of reconstructed bases per scaffold (excluding “N”) for datasets with error rates between 0.0 and 0.05 and depth coverage equal to 5× (DS66 to DS69).

**Supplementary Fig. S114**. Figure comparing the performance of the reconstruction programs in terms of the maximum number of reconstructed bases per scaffold for datasets with error rates between 0.0 and 0.05 and depth coverage equal to 5× (DS66 to DS69).

**Supplementary Fig. S115**. Figure comparing the performance of the reconstruction programs in terms of the maximum number of reconstructed bases per scaffold (excluding “N”) for datasets with error rates between 0.0 and 0.05 and depth coverage equal to 5× (DS66 to DS69).

**Supplementary Fig. S116**. Figure comparing the performance of the reconstruction programs in terms of the average number of reconstructed bases per scaffold for datasets with error rates between 0.0 and 0.05 and depth coverage equal to 5× (DS66 to DS69).

**Supplementary Fig. S117**. Figure comparing the performance of the reconstruction programs in terms of the average number of reconstructed bases per scaffold (excluding “N”) for datasets with error rates between 0.0 and 0.05 and depth coverage equal to 5× (DS66 to DS69).

**Supplementary Fig. S118**. Figure comparing the performance of the reconstruction programs in terms of the ratio between the number of SNPs and the number of bases reconstructed for datasets with error rates between 0.0 and 0.05 and depth coverage equal to 5× (DS66 to DS69).

**Supplementary Fig. S119**. Figure comparing the performance of the reconstruction programs in terms of the ratio between the number of SNPs and the number of bases reconstructed (excluding “N”) for datasets with error rates between 0.0 and 0.05 and depth coverage equal to 5× (DS66 to DS69).

**Supplementary Fig. S120**. Figure comparing the performance of the reconstruction programs in terms of the number of bases reconstructed for datasets with error rates between 0.0 and 0.05 and depth coverage equal to 40× (DS70 to DS73).

**Supplementary Fig. S121**. Figure comparing the performance of the reconstruction programs in terms of the number of bases reconstructed (excluding “N”) for datasets with error rates between 0.0 and 0.05 and depth coverage equal to 40× (DS70 to DS73).

**Supplementary Fig. S122**. Figure comparing the performance of the reconstruction programs in terms of the minimum number of reconstructed bases per scaffold for datasets with error rates between 0.0 and 0.05 and depth coverage equal to 40× (DS70 to DS73).

**Supplementary Fig. S123**. Figure comparing the performance of the reconstruction programs in terms of the minimum number of reconstructed bases per scaffold (excluding “N”) for datasets with error rates between 0.0 and 0.05 and depth coverage equal to 40× (DS70 to DS73).

**Supplementary Fig. S124**. Figure comparing the performance of the reconstruction programs in terms of the maximum number of reconstructed bases per scaffold for datasets with error rates between 0.0 and 0.05 and depth coverage equal to 40× (DS70 to DS73).

**Supplementary Fig. S125**. Figure comparing the performance of the reconstruction programs in terms of the maximum number of reconstructed bases per scaffold (excluding “N”) for datasets with error rates between 0.0 and 0.05 and depth coverage equal to 40× (DS70 to DS73).

**Supplementary Fig. S126**. Figure comparing the performance of the reconstruction programs in terms of the average number of reconstructed bases per scaffold for datasets with error rates between 0.0 and 0.05 and depth coverage equal to 40× (DS70 to DS73).

**Supplementary Fig. S127**. Figure comparing the performance of the reconstruction programs in terms of the average number of reconstructed bases per scaffold (excluding “N”) for datasets with error rates between 0.0 and 0.05 and depth coverage equal to 40× (DS70 to DS73).

**Supplementary Fig. S128**. Figure comparing the performance of the reconstruction programs in terms of the ratio between the number of SNPs and the number of bases reconstructed for datasets with error rates between 0.0 and 0.05 and depth coverage equal to 40× (DS70 to DS73).

**Supplementary Fig. S129**. Figure comparing the performance of the reconstruction programs in terms of the ratio between the number of SNPs and the number of bases reconstructed (excluding “N”) for datasets with error rates between 0.0 and 0.05 and depth coverage equal to 40× (DS70 to DS73).

**Supplementary Fig. S130**. Figure comparing the average performance of the reconstruction programs in terms of the execution time across all datasets reconstructed. The y-axis is presented in a logarithmic scale of base 2.

**Supplementary Fig. S131**. Figure comparing the average performance of the reconstruction programs in terms of the weighted execution time across all datasets reconstructed.

**Supplementary Fig. S132**. Figure comparing the average performance of the reconstruction programs in terms of the percentage of CPU used across all datasets reconstructed.

**Supplementary Fig. S133**. Figure comparing the average performance of the reconstruction programs in terms of the weighted percentage of CPU used across all datasets reconstructed.

**Supplementary Fig. S134**. Figure comparing the average performance of the reconstruction programs in terms of the RAM used across all datasets reconstructed.

**Supplementary Fig. S135**. Figure comparing the average performance of the reconstruction programs in terms of the weighted RAM used across all datasets reconstructed.

**Supplementary Fig. S136**. Figure comparing the average performance of the reconstruction programs in terms of the number of scaffolds reconstructed across all datasets reconstructed.

**Supplementary Fig. S137**. Figure comparing the average performance of the reconstruction programs in terms of the number of SNPs across all datasets reconstructed.

**Supplementary Fig. S138**. Figure comparing the average performance of the reconstruction programs in terms of the overall number of bases reconstructed across all datasets reconstructed. The blue bars show the performance using all bases present in the reconstructed file, whereas the orange bars show the results excluding non-reconstructed bases (N).

**Supplementary Fig. S139**. Figure comparing the average performance of the reconstruction programs in terms of the minimum number of bases reconstructed per scaffold across all datasets reconstructed. The blue bars show the performance using all bases present in the reconstructed file, whereas the orange bars show the results excluding non-reconstructed bases (N).

**Supplementary Fig. S140**. Figure comparing the average performance of the reconstruction programs in terms of the maximum number of bases reconstructed by scaffold across all datasets reconstructed. The blue bars show the performance using all bases present in the reconstructed file, whereas the orange bars show the results excluding non-reconstructed bases (N).

**Supplementary Fig. S141**. Figure comparing the average performance of the reconstruction programs in terms of the average number of bases reconstructed by scaffold across all datasets reconstructed. The blue bars show the performance using all bases present in the reconstructed file, whereas the orange bars show the results excluding non-reconstructed bases (N).

**Supplementary Fig. S142**. Figure comparing the average performance of the reconstruction programs in terms of the number of SNPs in relation to the number of bases reconstructed across all datasets reconstructed. The blue bars show the performance using all bases present in the reconstructed file, whereas the orange bars show the results excluding non-reconstructed bases (N).

**Supplementary Fig. S143**. Figure comparing the performance of the reconstruction programs in terms of the number of bases reconstructed for datasets SRR23101281, SRR23101235, SRR23101259, SRR23101276, SRR23101228, and SRR12175231.

**Supplementary Fig. S144**. Figure comparing the performance of the reconstruction programs in terms of the minimum number of reconstructed bases per scaffold for datasets SRR23101281, SRR23101235, SRR23101259, SRR23101276, SRR23101228, and SRR12175231.

**Supplementary Fig. S145**. Figure comparing the performance of the reconstruction programs in terms of the maximum number of reconstructed bases per scaffold for datasets SRR23101281, SRR23101235, SRR23101259, SRR23101276, SRR23101228, and SRR12175231.

**Supplementary Fig. S146**. Figure comparing the performance of the reconstruction programs in terms of the average number of reconstructed bases per scaffold for datasets SRR23101281, SRR23101235, SRR23101259, SRR23101276, SRR23101228, and SRR12175231.

**Supplementary Fig. S147**. Figure comparing the performance of the reconstruction programs in terms of the number of scaffolds reconstructed in dataset SRR23101281.

**Supplementary Fig. S148**. Figure comparing the performance of the reconstruction programs in terms of the number of scaffolds reconstructed in dataset SRR23101235.

**Supplementary Fig. S149**. Figure comparing the performance of the reconstruction programs in terms of the number of scaffolds reconstructed in dataset SRR23101259.

**Supplementary Fig. S150**. Figure comparing the performance of the reconstruction programs in terms of the number of scaffolds reconstructed in dataset SRR23101276.

**Supplementary Fig. S151**. Figure comparing the performance of the reconstruction programs in terms of the number of scaffolds reconstructed in dataset SRR23101228.

**Supplementary Fig. S152**. Figure comparing the performance of the reconstruction programs in terms of the number of scaffolds reconstructed in dataset SRR12175231.

**Supplementary Fig. S153**. Figure comparing the performance of the reconstruction programs in terms of the number of datasets reconstructed across datasets SRR23101281, SRR23101235, SRR23101259, SRR23101276, SRR23101228, and SRR12175231.

**Supplementary Fig. S154**. Figure comparing the average performance of the reconstruction programs in terms of the execution time across datasets SRR23101281, SRR23101235, SRR23101259, SRR23101276, SRR23101228, and SRR12175231. The y-axis is presented in a logarithmic scale of base 2.

**Supplementary Fig. S155**. Figure comparing the average performance of the reconstruction programs in terms of the percentage of CPU used across datasets SRR23101281, SRR23101235, SRR23101259, SRR23101276, SRR23101228, and SRR12175231.

**Supplementary Fig. S156**. Figure comparing the average performance of the reconstruction programs in terms of the RAM used across datasets SRR23101281, SRR23101235, SRR23101259, SRR23101276, SRR23101228, and SRR12175231.

**Supplementary Fig. S157**. Figure comparing the average performance of the reconstruction programs in terms of the overall number of bases reconstructed across datasets SRR23101281, SRR23101235, SRR23101259, SRR23101276, SRR23101228, and SRR12175231. The blue bars show the performance using all bases present in the reconstructed file, whereas the orange bars show the results excluding non-reconstructed bases (N).

**Supplementary Fig. S158**. Figure comparing the average performance of the reconstruction programs in terms of the minimum number of bases reconstructed per scaffold across datasets SRR23101281, SRR23101235, SRR23101259, SRR23101276, SRR23101228, and SRR12175231. The blue bars show the performance using all bases present in the reconstructed file, whereas the orange bars show the results excluding non-reconstructed bases (N).

**Supplementary Fig. S159**. Figure comparing the average performance of the reconstruction programs in terms of the maximum number of bases reconstructed by scaffold across datasets SRR23101281, SRR23101235, SRR23101259, SRR23101276, SRR23101228, and SRR12175231. The blue bars show the performance using all bases present in the reconstructed file, whereas the orange bars show the results excluding non-reconstructed bases (N).

**Supplementary Fig. S160**. Figure comparing the average performance of the reconstruction programs in terms of the average number of bases reconstructed by scaffold across datasets SRR23101281, SRR23101235, SRR23101259, SRR23101276, SRR23101228, and SRR12175231. The blue bars show the performance using all bases present in the reconstructed file, whereas the orange bars show the results excluding non-reconstructed bases (N).

**Supplementary Table S1**. Main characteristics of the synthetic datasets used in the benchmark generated with ART [1]. The fold coverage and read length of the datasets were defined when simulating the sequencing process. The number of SNPs refers to the ratio of mutations added to each of the viral sequences, which were added using GTO [2], and the substitution mutation refers to the ratio of mutations in the contamination genome, defined when using AlcoR [3].

**Supplementary Table S2**. Main characteristics of the synthetic datasets used in the benchmark generated with wgsim [4]. The read length of the datasets was defined when simulating the sequencing process, and the depth coverage was estimated from the number of reads generated. The number of SNPs refers to the ratio of mutations added to each of the viral sequences using GTO [2]; the substitution mutation refers to the ratio of mutations in the contamination genome, defined when using AlcoR [3]; and the error rate was set when simulating the datasets with wgsim.

**Supplementary Table S3**. Viral sequences present in each of the generated datasets used in the benchmark.

**Supplementary Table S4**. Viral sequences contained in the synthetic datasets, along with their GC content and compression ratio. The compression of the sequences was made using GeCo3 [5].

**Supplementary Table S5**. Results obtained for DS1 using the benchmark proposed. The execution time was measured in seconds, the RAM usage was measured in GB, and the CPU usage is presented as a percentage. The executions were, when possible, capped at 6 threads and 48 GB of RAM.

**Supplementary Table S6**. Results obtained for DS2 using the benchmark proposed. The execution time was measured in seconds, the RAM usage was measured in GB, and the CPU usage is presented as a percentage. The executions were, when possible, capped at 6 threads and 48 GB of RAM.

**Supplementary Table S7**. Results obtained for DS3 using the benchmark proposed. The execution time was measured in seconds, the RAM usage was measured in GB, and the CPU usage is presented as a percentage. The executions were, when possible, capped at 6 threads and 48 GB of RAM.

**Supplementary Table S8**. Results obtained for DS4 using the benchmark proposed. The execution time was measured in seconds, the RAM usage was measured in GB, and the CPU usage is presented as a percentage. The executions were, when possible, capped at 6 threads and 48 GB of RAM.

**Supplementary Table S9**. Results obtained for DS5 using the benchmark proposed. The execution time was measured in seconds, the RAM usage was measured in GB, and the CPU usage is presented as a percentage. The executions were, when possible, capped at 6 threads and 48 GB of RAM.

**Supplementary Table S10**. Results obtained for DS6 using the benchmark proposed. The execution time was measured in seconds, the RAM usage was measured in GB, and the CPU usage is presented as a percentage. The executions were, when possible, capped at 6 threads and 48 GB of RAM.

**Supplementary Table S11**. Results obtained for DS7 using the benchmark proposed. The execution time was measured in seconds, the RAM usage was measured in GB, and the CPU usage is presented as a percentage. The executions were, when possible, capped at 6 threads and 48 GB of RAM.

**Supplementary Table S12**. Results obtained for DS8 using the benchmark proposed. The execution time was measured in seconds, the RAM usage was measured in GB, and the CPU usage is presented as a percentage. The executions were, when possible, capped at 6 threads and 48 GB of RAM.

**Supplementary Table S13**. Results obtained for DS9 using the benchmark proposed. The execution time was measured in seconds, the RAM usage was measured in GB, and the CPU usage is presented as a percentage. The executions were, when possible, capped at 6 threads and 48 GB of RAM.

**Supplementary Table S14**. Results obtained for DS10 using the benchmark proposed. The execution time was measured in seconds, the RAM usage was measured in GB, and the CPU usage is presented as a percentage. The executions were, when possible, capped at 6 threads and 48 GB of RAM.

**Supplementary Table S15**. Results obtained for DS11 using the benchmark proposed. The execution time was measured in seconds, the RAM usage was measured in GB, and the CPU usage is presented as a percentage. The executions were, when possible, capped at 6 threads and 48 GB of RAM.

**Supplementary Table S16**. Results obtained for DS12 using the benchmark proposed. The execution time was measured in seconds, the RAM usage was measured in GB, and the CPU usage is presented as a percentage. The executions were, when possible, capped at 6 threads and 48 GB of RAM.

**Supplementary Table S17**. Results obtained for DS13 using the benchmark proposed. The execution time was measured in seconds, the RAM usage was measured in GB, and the CPU usage is presented as a percentage. The executions were, when possible, capped at 6 threads and 48 GB of RAM.

**Supplementary Table S18**. Results obtained for DS14 using the benchmark proposed. The execution time was measured in seconds, the RAM usage was measured in GB, and the CPU usage is presented as a percentage. The executions were, when possible, capped at 6 threads and 48 GB of RAM.

**Supplementary Table S19**. Results obtained for DS15 using the benchmark proposed. The execution time was measured in seconds, the RAM usage was measured in GB, and the CPU usage is presented as a percentage. The executions were, when possible, capped at 6 threads and 48 GB of RAM.

**Supplementary Table S20**. Results obtained for DS16 using the benchmark proposed. The execution time was measured in seconds, the RAM usage was measured in GB, and the CPU usage is presented as a percentage. The executions were, when possible, capped at 6 threads and 48 GB of RAM.

**Supplementary Table S21**. Results obtained for DS17 using the benchmark proposed. The execution time was measured in seconds, the RAM usage was measured in GB, and the CPU usage is presented as a percentage. The executions were, when possible, capped at 6 threads and 48 GB of RAM.

**Supplementary Table S22**. Results obtained for DS18 using the benchmark proposed. The execution time was measured in seconds, the RAM usage was measured in GB, and the CPU usage is presented as a percentage. The executions were, when possible, capped at 6 threads and 48 GB of RAM.

**Supplementary Table S23**. Results obtained for DS19 using the benchmark proposed. The execution time was measured in seconds, the RAM usage was measured in GB, and the CPU usage is presented as a percentage. The executions were, when possible, capped at 6 threads and 48 GB of RAM.

**Supplementary Table S24**. Results obtained for DS20 using the benchmark proposed. The execution time was measured in seconds, the RAM usage was measured in GB, and the CPU usage is presented as a percentage. The executions were, when possible, capped at 6 threads and 48 GB of RAM.

**Supplementary Table S25**. Results obtained for DS21 using the benchmark proposed. The execution time was measured in seconds, the RAM usage was measured in GB, and the CPU usage is presented as a percentage. The executions were, when possible, capped at 6 threads and 48 GB of RAM.

**Supplementary Table S26**. Results obtained for DS22 using the benchmark proposed. The execution time was measured in seconds, the RAM usage was measured in GB and the CPU usage is presented as a percentage. The executions were, when possible, capped at 6 threads and 48 GB of RAM.

**Supplementary Table S27**. Results obtained for DS23 using the benchmark proposed. The execution time was measured in seconds, the RAM usage was measured in GB, and the CPU usage is presented as a percentage. The executions were, when possible, capped at 6 threads and 48 GB of RAM.

**Supplementary Table S28**. Results obtained for DS24 using the benchmark proposed. The execution time was measured in seconds, the RAM usage was measured in GB, and the CPU usage is presented as a percentage. The executions were, when possible, capped at 6 threads and 48 GB of RAM.

**Supplementary Table S29**. Results obtained for DS25 using the benchmark proposed. The execution time was measured in seconds, the RAM usage was measured in GB, and the CPU usage is presented as a percentage. The executions were, when possible, capped at 6 threads and 48 GB of RAM.

**Supplementary Table S30**. Results obtained for DS26 using the benchmark proposed. The execution time was measured in seconds, the RAM usage was measured in GB, and the CPU usage is presented as a percentage. The executions were, when possible, capped at 6 threads and 48 GB of RAM.

**Supplementary Table S31**. Results obtained for DS27 using the benchmark proposed. The execution time was measured in seconds, the RAM usage was measured in GB, and the CPU usage is presented as a percentage. The executions were, when possible, capped at 6 threads and 48 GB of RAM.

**Supplementary Table S32**. Results obtained for DS28 using the benchmark proposed. The execution time was measured in seconds, the RAM usage was measured in GB, and the CPU usage is presented as a percentage. The executions were, when possible, capped at 6 threads and 48 GB of RAM.

**Supplementary Table S33**. Results obtained for DS29 using the benchmark proposed. The execution time was measured in seconds, the RAM usage was measured in GB, and the CPU usage is presented as a percentage. The executions were, when possible, capped at 6 threads and 48 GB of RAM.

**Supplementary Table S34**. Results obtained for DS30 using the benchmark proposed. The execution time was measured in seconds, the RAM usage was measured in GB, and the CPU usage is presented as a percentage. The executions were, when possible, capped at 6 threads and 48 GB of RAM.

**Supplementary Table S35**. Results obtained for DS31 using the benchmark proposed. The execution time was measured in seconds, the RAM usage was measured in GB, and the CPU usage is presented as a percentage. The executions were, when possible, capped at 6 threads and 48 GB of RAM.

**Supplementary Table S36**. Results obtained for DS32 using the benchmark proposed. The execution time was measured in seconds, the RAM usage was measured in GB, and the CPU usage is presented as a percentage. The executions were, when possible, capped at 6 threads and 48 GB of RAM.

**Supplementary Table S37**. Results obtained for DS33 using the benchmark proposed. The execution time was measured in seconds, the RAM usage was measured in GB, and the CPU usage is presented as a percentage. The executions were, when possible, capped at 6 threads and 48 GB of RAM.

**Supplementary Table S38**. Results obtained for DS34 using the benchmark proposed. The execution time was measured in seconds, the RAM usage was measured in GB, and the CPU usage is presented as a percentage. The executions were, when possible, capped at 6 threads and 48 GB of RAM.

**Supplementary Table S39**. Results obtained for DS35 using the benchmark proposed. The execution time was measured in seconds, the RAM usage was measured in GB, and the CPU usage is presented as a percentage. The executions were, when possible, capped at 6 threads and 48 GB of RAM.

**Supplementary Table S40**. Results obtained for DS36 using the benchmark proposed. The execution time was measured in seconds, the RAM usage was measured in GB, and the CPU usage is presented as a percentage. The executions were, when possible, capped at 6 threads and 48 GB of RAM.

**Supplementary Table S41**. Results obtained for DS37 using the benchmark proposed. The execution time was measured in seconds, the RAM usage was measured in GB, and the CPU usage is presented as a percentage. The executions were, when possible, capped at 6 threads and 48 GB of RAM.

**Supplementary Table S42**. Results obtained for DS38 using the benchmark proposed. The execution time was measured in seconds, the RAM usage was measured in GB, and the CPU usage is presented as a percentage. The executions were, when possible, capped at 6 threads and 48 GB of RAM.

**Supplementary Table S43**. Results obtained for DS39 using the benchmark proposed. The execution time was measured in seconds, the RAM usage was measured in GB, and the CPU usage is presented as a percentage. The executions were, when possible, capped at 6 threads and 48 GB of RAM.

**Supplementary Table S44**. Results obtained for DS40 using the benchmark proposed. The execution time was measured in seconds, the RAM usage was measured in GB, and the CPU usage is presented as a percentage. The executions were, when possible, capped at 6 threads and 48 GB of RAM.

**Supplementary Table S45**. Results obtained for DS41 using the benchmark proposed. The execution time was measured in seconds, the RAM usage was measured in GB, and the CPU usage is presented as a percentage. The executions were, when possible, capped at 6 threads and 48 GB of RAM.

**Supplementary Table S46**. Results obtained for DS42 using the benchmark proposed. The execution time was measured in seconds, the RAM usage was measured in GB, and the CPU usage is presented as a percentage. The executions were, when possible, capped at 6 threads and 48 GB of RAM.

**Supplementary Table S47**. Results obtained for DS43 using the benchmark proposed. The execution time was measured in seconds, the RAM usage was measured in GB, and the CPU usage is presented as a percentage. The executions were, when possible, capped at 6 threads and 48 GB of RAM.

**Supplementary Table S48**. Results obtained for DS44 using the benchmark proposed. The execution time was measured in seconds, the RAM usage was measured in GB, and the CPU usage is presented as a percentage. The executions were, when possible, capped at 6 threads and 48 GB of RAM.

**Supplementary Table S49**. Results obtained for DS45 using the benchmark proposed. The execution time was measured in seconds, the RAM usage was measured in GB, and the CPU usage is presented as a percentage. The executions were, when possible, capped at 6 threads and 48 GB of RAM.

**Supplementary Table S50**. Results obtained for DS46 using the benchmark proposed. The execution time was measured in seconds, the RAM usage was measured in GB, and the CPU usage is presented as a percentage. The executions were, when possible, capped at 6 threads and 48 GB of RAM.

**Supplementary Table S51**. Results obtained for DS47 using the benchmark proposed. The execution time was measured in seconds, the RAM usage was measured in GB, and the CPU usage is presented as a percentage. The executions were, when possible, capped at 6 threads and 48 GB of RAM.

**Supplementary Table S52**. Results obtained for DS48 using the benchmark proposed. The execution time was measured in seconds, the RAM usage was measured in GB, and the CPU usage is presented as a percentage. The executions were, when possible, capped at 6 threads and 48 GB of RAM.

**Supplementary Table S53**. Results obtained for DS49 using the benchmark proposed. The execution time was measured in seconds, the RAM usage was measured in GB, and the CPU usage is presented as a percentage. The executions were, when possible, capped at 6 threads and 48 GB of RAM.

**Supplementary Table S54**. Results obtained for DS50 using the benchmark proposed. The execution time was measured in seconds, the RAM usage was measured in GB, and the CPU usage is presented as a percentage. The executions were, when possible, capped at 6 threads and 48 GB of RAM.

**Supplementary Table S55**. Results obtained for DS51 using the benchmark proposed. The execution time was measured in seconds, the RAM usage was measured in GB, and the CPU usage is presented as a percentage. The executions were, when possible, capped at 6 threads and 48 GB of RAM.

**Supplementary Table S56**. Results obtained for DS52 using the benchmark proposed. The execution time was measured in seconds, the RAM usage was measured in GB, and the CPU usage is presented as a percentage. The executions were, when possible, capped at 6 threads and 48 GB of RAM.

**Supplementary Table S57**. Results obtained for DS53 using the benchmark proposed. The execution time was measured in seconds, the RAM usage was measured in GB, and the CPU usage is presented as a percentage. The executions were, when possible, capped at 6 threads and 48 GB of RAM.

**Supplementary Table S58**. Results obtained for DS54 using the benchmark proposed. The execution time was measured in seconds, the RAM usage was measured in GB, and the CPU usage is presented as a percentage. The executions were, when possible, capped at 6 threads and 48 GB of RAM.

**Supplementary Table S59**. Results obtained for DS55 using the benchmark proposed. The execution time was measured in seconds, the RAM usage was measured in GB, and the CPU usage is presented as a percentage. The executions were, when possible, capped at 6 threads and 48 GB of RAM.

**Supplementary Table S60**. Results obtained for DS56 using the benchmark proposed. The execution time was measured in seconds, the RAM usage was measured in GB, and the CPU usage is presented as a percentage. The executions were, when possible, capped at 6 threads and 48 GB of RAM.

**Supplementary Table S61**. Results obtained for DS57 using the benchmark proposed. The execution time was measured in seconds, the RAM usage was measured in GB, and the CPU usage is presented as a percentage. The executions were, when possible, capped at 6 threads and 48 GB of RAM.

**Supplementary Table S62**. Results obtained for DS58 using the benchmark proposed. The execution time was measured in seconds, the RAM usage was measured in GB, and the CPU usage is presented as a percentage. The executions were, when possible, capped at 6 threads and 48 GB of RAM.

**Supplementary Table S63**. Results obtained for DS59 using the benchmark proposed. The execution time was measured in seconds, the RAM usage was measured in GB, and the CPU usage is presented as a percentage. The executions were, when possible, capped at 6 threads and 48 GB of RAM.

**Supplementary Table S64**. Results obtained for DS60 using the benchmark proposed. The execution time was measured in seconds, the RAM usage was measured in GB, and the CPU usage is presented as a percentage. The executions were, when possible, capped at 6 threads and 48 GB of RAM.

**Supplementary Table S65**. Results obtained for DS61 using the benchmark proposed. The execution time was measured in seconds, the RAM usage was measured in GB, and the CPU usage is presented as a percentage. The executions were, when possible, capped at 6 threads and 48 GB of RAM.

**Supplementary Table S66**. Results obtained for DS62 using the benchmark proposed. The execution time was measured in seconds, the RAM usage was measured in GB, and the CPU usage is presented as a percentage. The executions were, when possible, capped at 6 threads and 48 GB of RAM.

**Supplementary Table S67**. Results obtained for DS63 using the benchmark proposed. The execution time was measured in seconds, the RAM usage was measured in GB, and the CPU usage is presented as a percentage. The executions were, when possible, capped at 6 threads and 48 GB of RAM.

**Supplementary Table S68**. Results obtained for DS64 using the benchmark proposed. The execution time was measured in seconds, the RAM usage was measured in GB, and the CPU usage is presented as a percentage. The executions were, when possible, capped at 6 threads and 48 GB of RAM.

**Supplementary Table S69**. Results obtained for DS65 using the benchmark proposed. The execution time was measured in seconds, the RAM usage was measured in GB, and the CPU usage is presented as a percentage. The executions were, when possible, capped at 6 threads and 48 GB of RAM.

**Supplementary Table S70**. Results obtained for DS66 using the benchmark proposed. The execution time was measured in seconds, the RAM usage was measured in GB, and the CPU usage is presented as a percentage. The executions were, when possible, capped at 6 threads and 48 GB of RAM.

**Supplementary Table S71**. Results obtained for DS67 using the benchmark proposed. The execution time was measured in seconds, the RAM usage was measured in GB, and the CPU usage is presented as a percentage. The executions were, when possible, capped at 6 threads and 48 GB of RAM.

**Supplementary Table S72**. Results obtained for DS68 using the benchmark proposed. The execution time was measured in seconds, the RAM usage was measured in GB, and the CPU usage is presented as a percentage. The executions were, when possible, capped at 6 threads and 48 GB of RAM.

**Supplementary Table S73**. Results obtained for DS69 using the benchmark proposed. The execution time was measured in seconds, the RAM usage was measured in GB, and the CPU usage is presented as a percentage. The executions were, when possible, capped at 6 threads and 48 GB of RAM.

**Supplementary Table S74**. Results obtained for DS70 using the benchmark proposed. The execution time was measured in seconds, the RAM usage was measured in GB, and the CPU usage is presented as a percentage. The executions were, when possible, capped at 6 threads and 48 GB of RAM.

**Supplementary Table S75**. Results obtained for DS71 using the benchmark proposed. The execution time was measured in seconds, the RAM usage was measured in GB, and the CPU usage is presented as a percentage. The executions were, when possible, capped at 6 threads and 48 GB of RAM.

**Supplementary Table S76**. Results obtained for DS72 using the benchmark proposed. The execution time was measured in seconds, the RAM usage was measured in GB, and the CPU usage is presented as a percentage. The executions were, when possible, capped at 6 threads and 48 GB of RAM.

**Supplementary Table S77**. Results obtained for DS73 using the benchmark proposed. The execution time was measured in seconds, the RAM usage was measured in GB, and the CPU usage is presented as a percentage. The executions were, when possible, capped at 6 threads and 48 GB of RAM.

**Supplementary Table S78**. Results obtained by dnadiff [6] when analyzing the reconstruction of DS1. Only 1 execution of HVRS is represented.

**Supplementary Table S79**. Results obtained by dnadiff [6] when analyzing the reconstruction of DS2. Only 1 execution of HVRS is represented.

**Supplementary Table S80**. Results obtained by dnadiff [6] when analyzing the reconstruction of DS3. Only 1 execution of HVRS is represented.

**Supplementary Table S81**. Results obtained by dnadiff [6] when analyzing the reconstruction of DS4. Only 1 execution of HVRS is represented.

**Supplementary Table S82**. Results obtained by dnadiff [6] when analyzing the reconstruction of DS5. Only 1 execution of HVRS is represented.

**Supplementary Table S83**. Results obtained by dnadiff [6] when analyzing the reconstruction of DS6. Only 1 execution of HVRS is represented.

**Supplementary Table S84**. Results obtained by dnadiff [6] when analyzing the reconstruction of DS7. Only 1 execution of HVRS is represented.

**Supplementary Table S85**. Results obtained by dnadiff [6] when analyzing the reconstruction of DS8. Only 1 execution of HVRS is represented.

**Supplementary Table S86**. Results obtained by dnadiff [6] when analyzing the reconstruction of DS9. Only 1 execution of HVRS is represented.

**Supplementary Table S87**. Results obtained by dnadiff [6] when analyzing the reconstruction of DS10. Only 1 execution of HVRS is represented.

**Supplementary Table S88**. Results obtained by dnadiff [6] when analyzing the reconstruction of DS11. Only 1 execution of HVRS is represented.

**Supplementary Table S89**. Results obtained by dnadiff [6] when analyzing the reconstruction of DS12. Only 1 execution of HVRS is represented.

**Supplementary Table S90**. Results obtained by dnadiff [6] when analyzing the reconstruction of DS13. Only 1 execution of HVRS is represented.

**Supplementary Table S91**. Results obtained by dnadiff [6] when analyzing the reconstruction of DS14. Only 1 execution of HVRS is represented.

**Supplementary Table S92**. Results obtained by dnadiff [6] when analyzing the reconstruction of DS15. Only 1 execution of HVRS is represented.

**Supplementary Table S93**. Results obtained by dnadiff [6] when analyzing the reconstruction of DS16. Only 1 execution of HVRS is represented.

**Supplementary Table S94**. Results obtained by dnadiff [6] when analyzing the reconstruction of DS17. Only 1 execution of HVRS is represented.

**Supplementary Table S95**. Results obtained by dnadiff [6] when analyzing the reconstruction of DS18. Only 1 execution of HVRS is represented.

**Supplementary Table S96**. Results obtained by dnadiff [6] when analyzing the reconstruction of DS19. Only 1 execution of HVRS is represented.

**Supplementary Table S97**. Results obtained by dnadiff [6] when analyzing the reconstruction of DS20. Only 1 execution of HVRS is represented.

**Supplementary Table S98**. Results obtained by dnadiff [6] when analyzing the reconstruction of DS21. Only 1 execution of HVRS is represented.

**Supplementary Table S99**. Results obtained by dnadiff [6] when analyzing the reconstruction of DS22. Only 1 execution of HVRS is represented.

**Supplementary Table S100**. Results obtained by dnadiff [6] when analyzing the reconstruction of DS23. Only 1 execution of HVRS is represented.

**Supplementary Table S101**. Results obtained by dnadiff [6] when analyzing the reconstruction of DS24. Only 1 execution of HVRS is represented.

**Supplementary Table S102**. Results obtained by dnadiff [6] when analyzing the reconstruction of DS25. Only 1 execution of HVRS is represented.

**Supplementary Table S103**. Results obtained by dnadiff [6] when analyzing the reconstruction of DS26. Only 1 execution of HVRS is represented.

**Supplementary Table S104**. Results obtained by dnadiff [6] when analyzing the reconstruction of DS27. Only 1 execution of HVRS is represented.

**Supplementary Table S105**. Results obtained by dnadiff [6] when analyzing the reconstruction of DS28. Only 1 execution of HVRS is represented.

**Supplementary Table S106**. Results obtained by dnadiff [6] when analyzing the reconstruction of DS29. Only 1 execution of HVRS is represented.

**Supplementary Table S107**. Results obtained by dnadiff [6] when analyzing the reconstruction of DS30. Only 1 execution of HVRS is represented.

**Supplementary Table S108**. Results obtained by dnadiff [6] when analyzing the reconstruction of DS31. Only 1 execution of HVRS is represented.

**Supplementary Table S109**. Results obtained by dnadiff [6] when analyzing the reconstruction of DS32. Only 1 execution of HVRS is represented.

**Supplementary Table S110**. Results obtained by dnadiff [6] when analyzing the reconstruction of DS33. Only 1 execution of HVRS is represented.

**Supplementary Table S111**. Results obtained by dnadiff [6] when analyzing the reconstruction of DS34. Only 1 execution of HVRS is represented.

**Supplementary Table S112**. Results obtained by dnadiff [6] when analyzing the reconstruction of DS35. Only 1 execution of HVRS is represented.

**Supplementary Table S113**. Results obtained by dnadiff [6] when analyzing the reconstruction of DS36. Only 1 execution of HVRS is represented.

**Supplementary Table S114**. Results obtained by dnadiff [6] when analyzing the reconstruction of DS37. Only 1 execution of HVRS is represented.

**Supplementary Table S115**. Results obtained by dnadiff [6] when analyzing the reconstruction of DS38. Only 1 execution of HVRS is represented.

**Supplementary Table S116**. Results obtained by dnadiff [6] when analyzing the reconstruction of DS39. Only 1 execution of HVRS is represented.

**Supplementary Table S117**. Results obtained by dnadiff [6] when analyzing the reconstruction of DS40. Only 1 execution of HVRS is represented.

**Supplementary Table S118**. Results obtained by dnadiff [6] when analyzing the reconstruction of DS41. Only 1 execution of HVRS is represented.

**Supplementary Table S119**. Results obtained by dnadiff [6] when analyzing the reconstruction of DS42. Only 1 execution of HVRS is represented.

**Supplementary Table S120**. Results obtained by dnadiff [6] when analyzing the reconstruction of DS43. Only 1 execution of HVRS is represented.

**Supplementary Table S121**. Results obtained by dnadiff [6] when analyzing the reconstruction of DS44. Only 1 execution of HVRS is represented.

**Supplementary Table S122**. Results obtained by dnadiff [6] when analyzing the reconstruction of DS45. Only 1 execution of HVRS is represented.

**Supplementary Table S123**. Results obtained by dnadiff [6] when analyzing the reconstruction of DS46. Only 1 execution of HVRS is represented.

**Supplementary Table S124**. Results obtained by dnadiff [6] when analyzing the reconstruction of DS47. Only 1 execution of HVRS is represented.

**Supplementary Table S125**. Results obtained by dnadiff [6] when analyzing the reconstruction of DS48. Only 1 execution of HVRS is represented.

**Supplementary Table S126**. Results obtained by dnadiff [6] when analyzing the reconstruction of DS49. Only 1 execution of HVRS is represented.

**Supplementary Table S127**. Results obtained by dnadiff [6] when analyzing the reconstruction of DS50. Only 1 execution of HVRS is represented.

**Supplementary Table S128**. Results obtained by dnadiff [6] when analyzing the reconstruction of DS51. Only 1 execution of HVRS is represented.

**Supplementary Table S129**. Results obtained by dnadiff [6] when analyzing the reconstruction of DS52. Only 1 execution of HVRS is represented.

**Supplementary Table S130**. Results obtained by dnadiff [6] when analyzing the reconstruction of DS53. Only 1 execution of HVRS is represented.

**Supplementary Table S131**. Results obtained by dnadiff [6] when analyzing the reconstruction of DS54. Only 1 execution of HVRS is represented.

**Supplementary Table S132**. Results obtained by dnadiff [6] when analyzing the reconstruction of DS55. Only 1 execution of HVRS is represented.

**Supplementary Table S133**. Results obtained by dnadiff [6] when analyzing the reconstruction of DS56. Only 1 execution of HVRS is represented.

**Supplementary Table S134**. Results obtained by dnadiff [6] when analyzing the reconstruction of DS57. Only 1 execution of HVRS is represented.

**Supplementary Table S135**. Results obtained by dnadiff [6] when analyzing the reconstruction of DS58. Only 1 execution of HVRS is represented.

**Supplementary Table S136**. Results obtained by dnadiff [6] when analyzing the reconstruction of DS59. Only 1 execution of HVRS is represented.

**Supplementary Table S137**. Results obtained by dnadiff [6] when analyzing the reconstruction of DS60. Only 1 execution of HVRS is represented.

**Supplementary Table S138**. Results obtained by dnadiff [6] when analyzing the reconstruction of DS61. Only 1 execution of HVRS is represented.

**Supplementary Table S139**. Results obtained by dnadiff [6] when analyzing the reconstruction of DS62. Only 1 execution of HVRS is represented.

**Supplementary Table S140**. Results obtained by dnadiff [6] when analyzing the reconstruction of DS63. Only 1 execution of HVRS is represented.

**Supplementary Table S141**. Results obtained by dnadiff [6] when analyzing the reconstruction of DS64. Only 1 execution of HVRS is represented.

**Supplementary Table S142**. Results obtained by dnadiff [6] when analyzing the reconstruction of DS65. Only 1 execution of HVRS is represented.

**Supplementary Table S143**. Results obtained by dnadiff [6] when analyzing the reconstruction of DS66. Only 1 execution of HVRS is represented.

**Supplementary Table S144**. Results obtained by dnadiff [6] when analyzing the reconstruction of DS67. Only 1 execution of HVRS is represented.

**Supplementary Table S145**. Results obtained by dnadiff [6] when analyzing the reconstruction of DS68. Only 1 execution of HVRS is represented.

**Supplementary Table S146**. Results obtained by dnadiff [6] when analyzing the reconstruction of DS69. Only 1 execution of HVRS is represented.

**Supplementary Table S147**. Results obtained by dnadiff [6] when analyzing the reconstruction of DS70. Only 1 execution of HVRS is represented.

**Supplementary Table S148**. Results obtained by dnadiff [6] when analyzing the reconstruction of DS71. Only 1 execution of HVRS is represented.

**Supplementary Table S149**. Results obtained by dnadiff [6] when analyzing the reconstruction of DS72. Only 1 execution of HVRS is represented.

**Supplementary Table S150**. Results obtained by dnadiff [6] when analyzing the reconstruction of DS73. Only 1 execution of HVRS is represented.

**Supplementary Table S151**. Viral sequences included in each real dataset according to FALCON-meta [7] (top of similarity value set to 8,000) and the total number of reference genomes found per dataset.

**Supplementary Table S152**. Viral sequences found by FALCON-meta in the dataset SRR23101281, along with their GC content and compression ratio. The compression of the sequences was made using GeCo3 [5].

**Supplementary Table S153**. Viral sequences found by FALCON-meta in the dataset SRR23101235, along with their GC content and compression ratio. The compression of the sequences was made using GeCo3 [5].

**Supplementary Table S154**. Viral sequences found by FALCON-meta in the dataset SRR23101259, along with their GC content and compression ratio. The compression of the sequences was made using GeCo3 [5].

**Supplementary Table S155**. Viral sequences found by FALCON-meta in the dataset SRR23101276, along with their GC content and compression ratio. The compression of the sequences was made using GeCo3 [5].

**Supplementary Table S156**. Viral sequences found by FALCON-meta in the dataset SRR23101228, along with their GC content and compression ratio. The compression of the sequences was made using GeCo3 [5].

**Supplementary Table S157**. Viral sequences found by FALCON-meta in the dataset SRR12175231, along with their GC content and compression ratio. The compression of the sequences was made using GeCo3 [5].

**Supplementary Table S158**. Results obtained for SRR23101281 using the benchmark proposed. The execution time was measured in seconds, the RAM usage was measured in GB, and the CPU usage is presented as a percentage. The executions were, when possible, capped at 6 threads and 48 GB of RAM.

**Supplementary Table S159**. Results obtained for SRR23101235 using the benchmark proposed. The execution time was measured in seconds, the RAM usage was measured in GB, and the CPU usage is presented as a percentage. The executions were, when possible, capped at 6 threads and 48 GB of RAM.

**Supplementary Table S160**. Results obtained for SRR23101259 using the benchmark proposed. The execution time was measured in seconds, the RAM usage was measured in GB, and the CPU usage is presented as a percentage. The executions were, when possible, capped at 6 threads and 48 GB of RAM.

**Supplementary Table S161**. Results obtained for SRR23101276 using the benchmark proposed. The execution time was measured in seconds, the RAM usage was measured in GB, and the CPU usage is presented as a percentage. The executions were, when possible, capped at 6 threads and 48 GB of RAM.

**Supplementary Table S162**. Results obtained for SRR23101228 using the benchmark proposed. The execution time was measured in seconds, the RAM usage was measured in GB, and the CPU usage is presented as a percentage. The executions were, when possible, capped at 6 threads and 48 GB of RAM.

**Supplementary Table S163**. Results obtained for SRR12175231 using the benchmark proposed. The execution time was measured in seconds, the RAM usage was measured in GB, and the CPU usage is presented as a percentage. The executions were, when possible, capped at 6 threads and 48 GB of RAM.

## Abbreviations

B19V: human parvovirus B19; EBV: Epstein–Barr virus; GUI: graphical user interface; HB: hybrid; HCMV: human cytomegalovirus; HiFi reads: highly accurate long reads; HHV6B: human herpesvirus 6B; HMM: hidden Markov model; HPV: human papillomavirus; HPyV7: human polyomavirus 7; MCPyV: Merkel cell polyomavirus; NCD: normalized compression distance; NCSD: normalized compression semi-distance; NGS: next-generation sequencing; NID: normalized information distance; NRC: normalized relative compression; PCR: polymerase chain reaction; RB: reference-based; RF: reference-free; SNP: single-nucleotide polymorphism; VZV: varicella zoster virus.
