# Supplementary material for: An evaluation of computational methods for reconstruction of human viral DNA genomes
Source: Gigascience. 2025 Dec 26;15:giaf159. doi: 10.1093/gigascience/giaf159 (PMC12927430; doi:10.1093/gigascience/giaf159)
Supplement: giaf159_Supplemental_File [file giaf159_supplemental_file.pdf]

Supplementary Material of
"An evaluation of computational methods for reconstruction of human viral DNA genomes"

Maria J. P. Sousa[1,2], Mari Toppinen[3], Lari Pyöriä [4], Klaus Hedman [4], Antti Sajantila [3,5], Maria F. Perdomo [4], Diogo Pratas[1,2,4]

[1]Institute of Electronics and Informatics Engineering of Aveiro and Intelligent Systems Associate Laboratory, University of Aveiro
[2]Department of Electronics, Telecommunications and Informatics, University of Aveiro
[3]Department of Forensic Medicine, University of Helsinki
[4]Department of Virology and Helsinki University Hospital, University of Helsinki
[5]Forensic Medicine Unit, Finnish Institute for Health and Welfare

1 Characteristics of the datasets generated

Table S1: Main characteristics of the synthetic datasets used in the benchmark generated with ART [1]. The fold coverage and read length of the datasets were defined when simulating the sequencing process. The number of SNPs refers to the ratio of mutations added to each one of the viral sequences, which were added using GTO [2] and the substitution mutation refers to the ratio of mutations in the contamination genome, defined when using AlcoR [3].

Identifier	Read length	Fold coverage	SNPs (GTO)	Substitution mutation (AlcoR)	Includes contamination genome	Includes mitochondrial genome
DS1	150	2	0.01	–	No	No
DS2	150	5	0.01	–	No	No
DS3	150	10	0.01	–	No	No
DS4	150	15	0.01	–	No	No
DS5	150	20	0.01	–	No	No
DS6	150	25	0.01	–	No	No
DS7	150	30	0.01	–	No	No
DS8	150	40	0.01	–	No	No
DS9	150	2	0.01	0.3	Yes	Yes
DS10	150	5	0.01	0.3	Yes	Yes
DS11	150	10	0.01	0.3	Yes	Yes
DS12	150	15	0.01	0.3	Yes	Yes
DS13	150	20	0.01	0.3	Yes	Yes
DS14	150	25	0.01	0.3	Yes	Yes
DS15	150	30	0.01	0.3	Yes	Yes
DS16	150	40	0.01	0.3	Yes	Yes
DS17	150	2	–	0.3	Yes	Yes
DS18	150	2	0.03	0.3	Yes	Yes
DS19	150	2	0.05	0.3	Yes	Yes
DS20	150	2	0.07	0.3	Yes	Yes
DS21	150	2	0.09	0.3	Yes	Yes
DS22	150	2	0.11	0.3	Yes	Yes
DS23	150	2	0.13	0.3	Yes	Yes
DS24	150	2	0.15	0.3	Yes	Yes
DS25	150	5	–	0.3	Yes	Yes
DS26	150	5	0.03	0.3	Yes	Yes
DS27	150	5	0.05	0.3	Yes	Yes
DS28	150	5	0.07	0.3	Yes	Yes
DS29	150	5	0.09	0.3	Yes	Yes
DS30	150	5	0.11	0.3	Yes	Yes
DS31	150	5	0.13	0.3	Yes	Yes
DS32	150	5	0.15	0.3	Yes	Yes
DS33	150	10	–	0.3	Yes	Yes
DS34	150	10	0.03	0.3	Yes	Yes
DS35	150	10	0.05	0.3	Yes	Yes
DS36	150	10	0.07	0.3	Yes	Yes
DS37	150	10	0.09	0.3	Yes	Yes
DS38	150	10	0.11	0.3	Yes	Yes
DS39	150	10	0.13	0.3	Yes	Yes
DS40	150	10	0.15	0.3	Yes	Yes
DS41	150	20	–	0.3	Yes	Yes
DS42	150	20	0.03	0.3	Yes	Yes
DS43	150	20	0.05	0.3	Yes	Yes
DS44	150	20	0.07	0.3	Yes	Yes
DS45	150	20	0.09	0.3	Yes	Yes

DS46	150	20	0.11	0.3	Yes	Yes
DS47	150	20	0.13	0.3	Yes	Yes
DS48	150	20	0.15	0.3	Yes	Yes
DS49	150	40	–	0.3	Yes	Yes
DS50	150	40	0.03	0.3	Yes	Yes
DS51	150	40	0.05	0.3	Yes	Yes
DS52	150	40	0.07	0.3	Yes	Yes
DS53	150	40	0.09	0.3	Yes	Yes
DS54	150	40	0.11	0.3	Yes	Yes
DS55	150	40	0.13	0.3	Yes	Yes
DS56	150	40	0.15	0.3	Yes	Yes
DS57	150	20	0.01	0.3	Yes	No
DS58	150	20	0.01	–	No	Yes
DS59	150	30	0.03	0.3	Yes	Yes
DS60	150	40	0.05	0.6	Yes	Yes
DS61	75	20	0.01	0.3	Yes	Yes
DS62	250	20	0.01	0.3	Yes	Yes
DS63	150	20	0.01	0.3	Yes	Yes
DS64	150	20	0.01	0.3	Yes	Yes
DS65	150	20	0.01	0.3	Yes	Yes

Table S2: Main characteristics of the synthetic datasets used in the benchmark generated with wgsim [4]. The read length of the datasets was defined when simulating the sequencing process and the depth coverage was estimated from the number of reads generated. The number of SNPs refers to the ratio of mutations added to each one of the viral sequences using GTO [2], the substitution mutation refers to the ratio of mutations in the contamination genome, defined when using AlcoR [3] and the error rate was set when simulating the datasets with wgsim.

**Identifier**	**Read length**	**Fold coverage**	**SNPs (GTO)**	**Substitution mutation (AlcoR)**	**Includes contamination genome**	**Includes mitochondrial genome**	**Error rate**
DS66	150	5	0.0	0.3	Yes	Yes	0.0
DS67	150	5	0.0	0.3	Yes	Yes	0.01
DS68	150	5	0.0	0.3	Yes	Yes	0.025
DS69	150	5	0.0	0.3	Yes	Yes	0.05
DS70	150	40	0.0	0.3	Yes	Yes	0.0
DS71	150	40	0.0	0.3	Yes	Yes	0.01
DS72	150	40	0.0	0.3	Yes	Yes	0.025
DS73	150	40	0.0	0.3	Yes	Yes	0.05

Table S3: Viral sequences present in each of the generated datasets used in the benchmark.

**Dataset**	**B19V**	**HPV**	**VZV**	**MCPyV**	**HPyV7**	**HHV6B**	**EBV**	**HCMV**
DS1 - DS62	x	x	x	x				
DS63	x	x	x	x		x		
DS64	x	x	x		x		x	
DS65	x	x	x	x				x
DS66 - DS73	x	x	x	x				

Table S4: Viral sequences contained in the synthetic datasets, along with their GC content and compression ratio. The compression of the sequences was made using GeCo3 [5].

**Sequence identification**	**GC content (%)**	**Compression ratio (GeCo3)**
>AY386330.1 B19 virus isolate J35, complete genome	0.43817	0.249419
>KU298932.1 Human papillomavirus 68 isolate 83B.68, complete genome	0.398545	0.253286
>DQ479959.1 Human herpesvirus 3 strain 49, complete genome	0.460913	0.234513
>KX827417.1 Merkel cell polyomavirus isolate MCPyV_124Sweden, complete genome	0.406906	0.256496
>NC_014407.1 Human polyomavirus 7, complete genome	0.428918	0.258429
>NC_000898.1 Human herpesvirus 6B, complete genome	0.427711	0.228603
>NC_009334.1 Human herpesvirus 4, complete genome	0.595425	0.207646
>KP745701.1 Human herpesvirus 5 strain BE/6/2010, complete genome	0.574982	0.241253

2 Supplementary Figures and Tables

2.1 Generated datasets

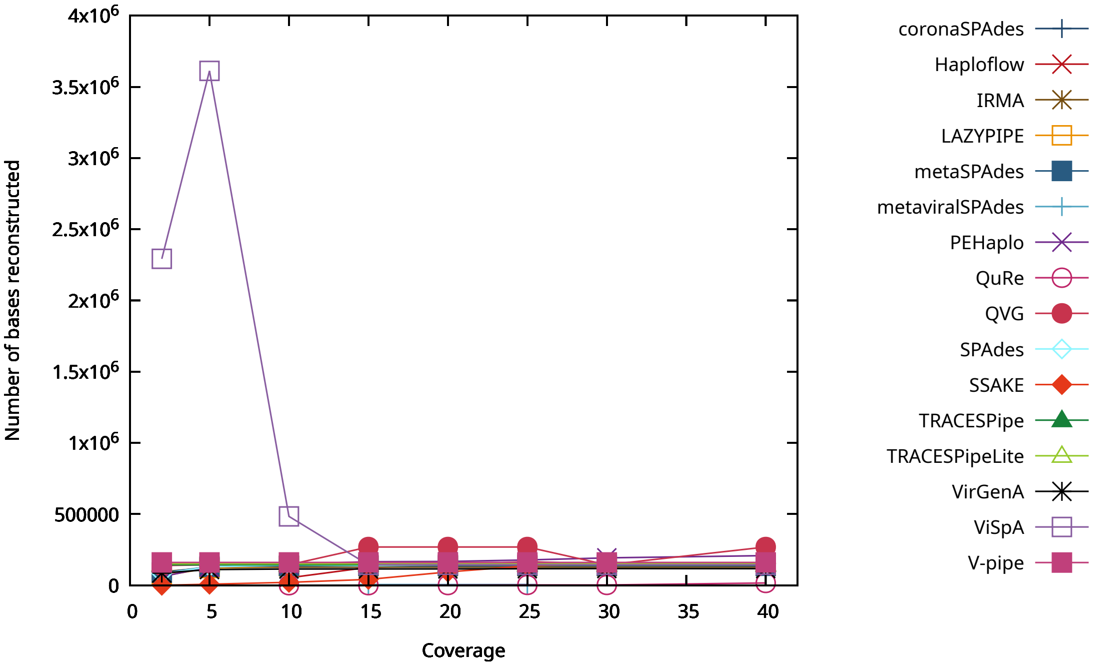

Figure S1: Figure comparing the performance of the reconstruction programs in terms of the number of bases reconstructed for datasets without contamination or mitochondrial DNA (DS1 to DS8).

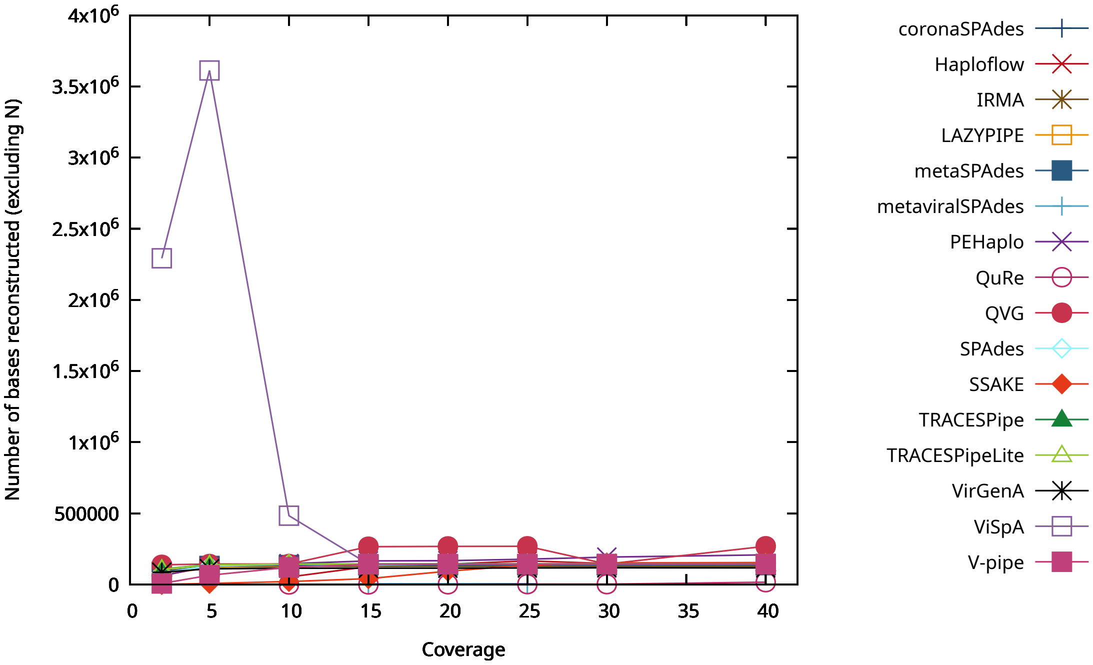

Figure S2: Figure comparing the performance of the reconstruction programs in terms of the number of bases reconstructed (excluding "N") for datasets without contamination or mitochondrial DNA (DS1 to DS8).

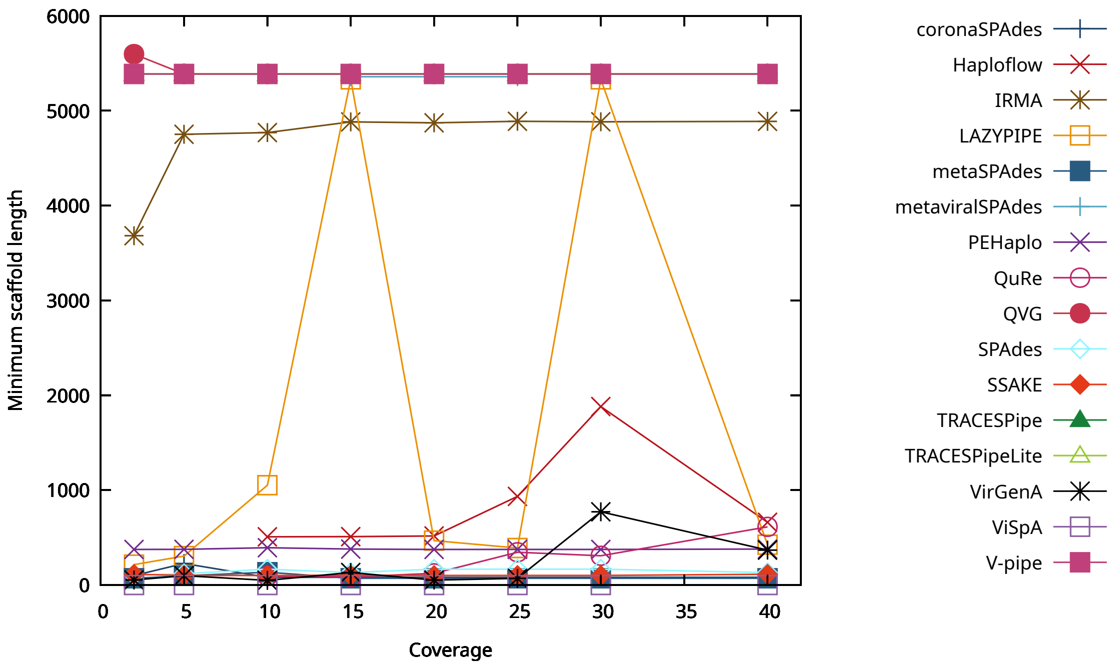

Figure S3: Figure comparing the performance of the reconstruction programs in terms of the minimum number of reconstructed bases per scaffold for datasets without contamination or mitochondrial DNA (DS1 to DS8).

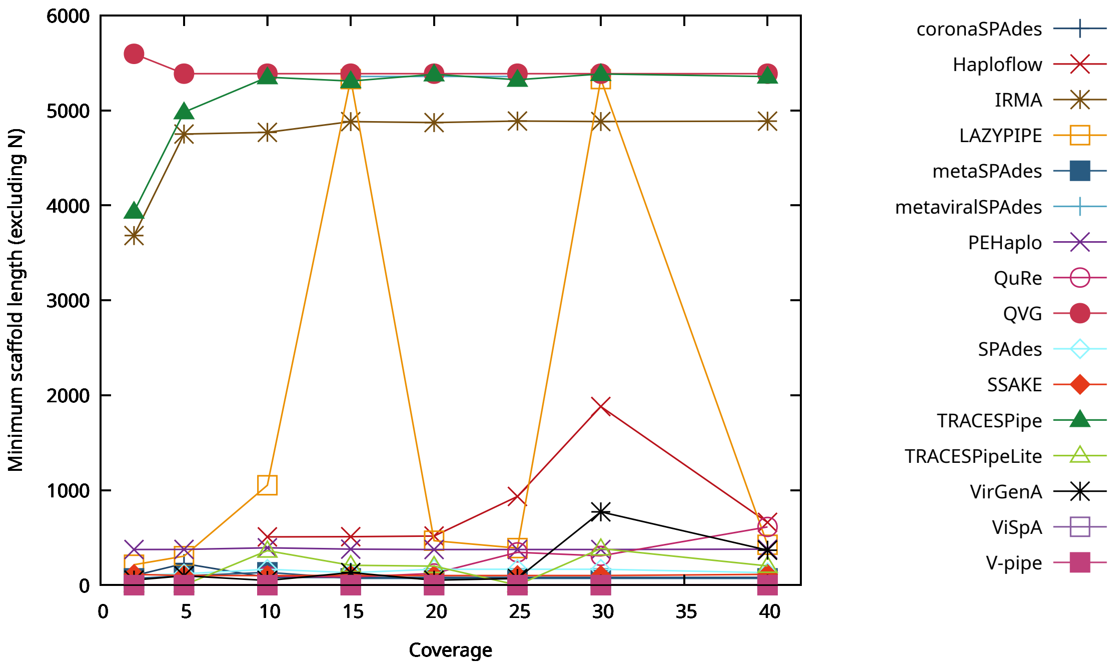

Figure S4: Figure comparing the performance of the reconstruction programs in terms of the minimum number of reconstructed bases per scaffold (excluding "N") for datasets without contamination or mitochondrial DNA (DS1 to DS8).

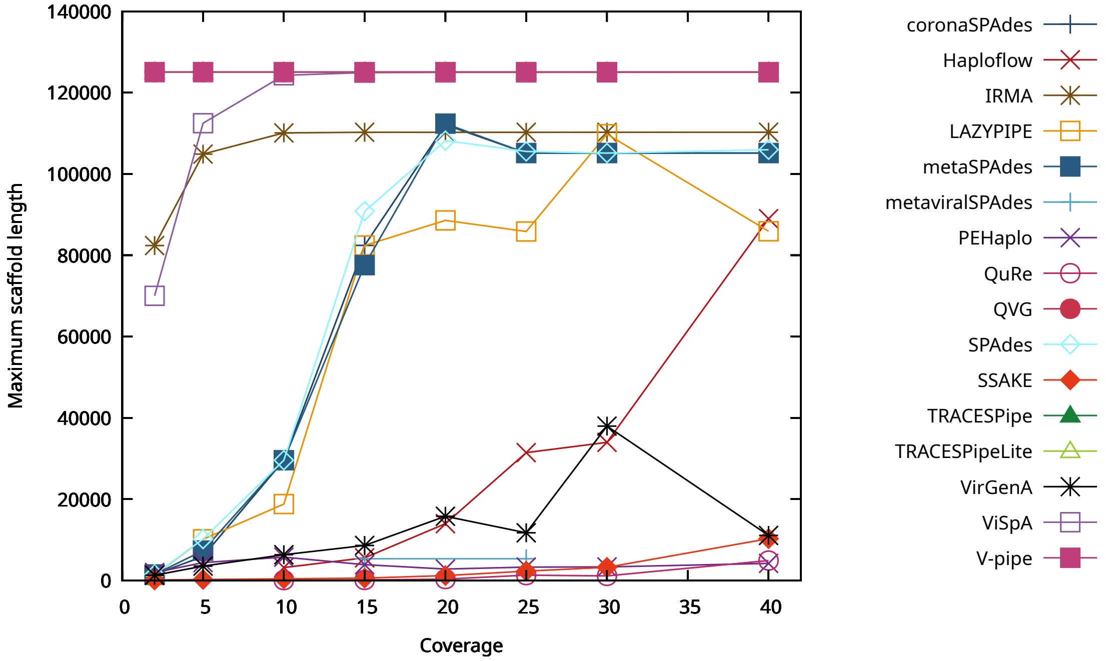

Figure S5: Figure comparing the performance of the reconstruction programs in terms of the maximum number of reconstructed bases per scaffold for datasets without contamination or mitochondrial DNA (DS1 to DS8).

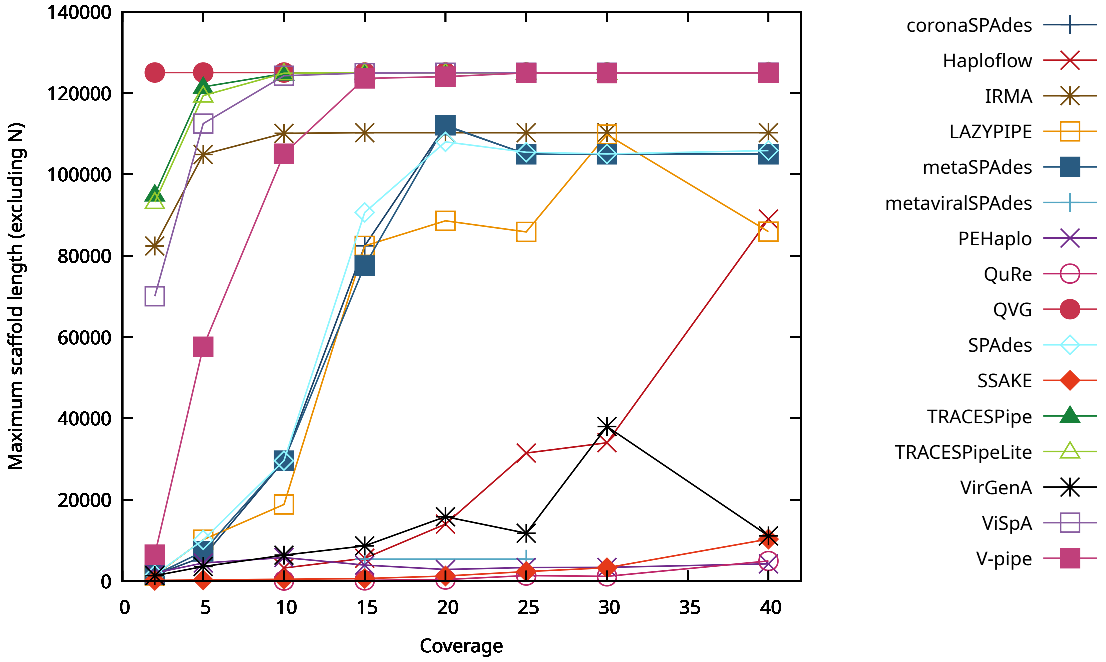

Figure S6: Figure comparing the performance of the reconstruction programs in terms of the maximum number of reconstructed bases per scaffold (excluding "N") for datasets without contamination or mitochondrial DNA (DS1 to DS8).

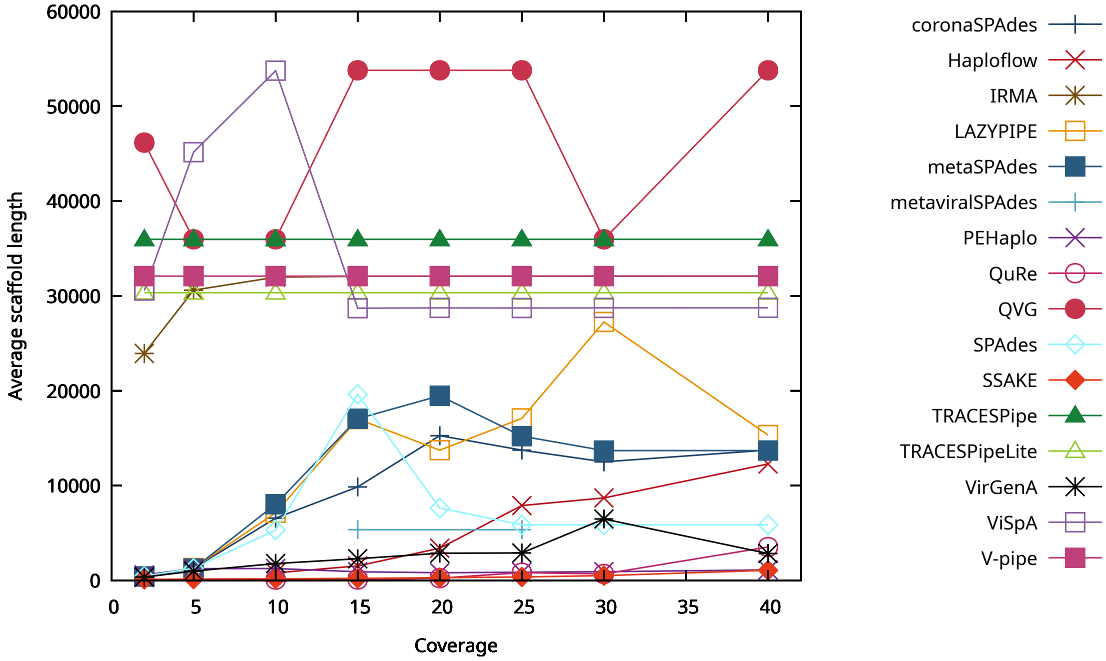

Figure S7: Figure comparing the performance of the reconstruction programs in terms of the average number of reconstructed bases per scaffold for datasets without contamination or mitochondrial DNA (DS1 to DS8).

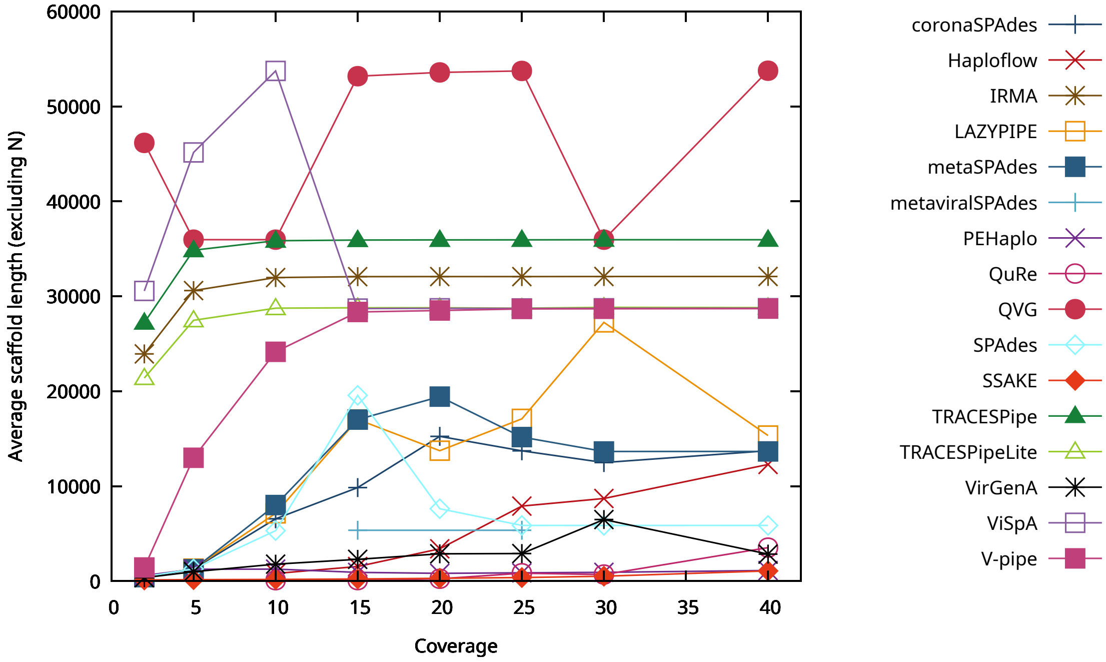

Figure S8: Figure comparing the performance of the reconstruction programs in terms of the average number of reconstructed bases per scaffold (excluding "N") for datasets without contamination or mitochondrial DNA (DS1 to DS8).

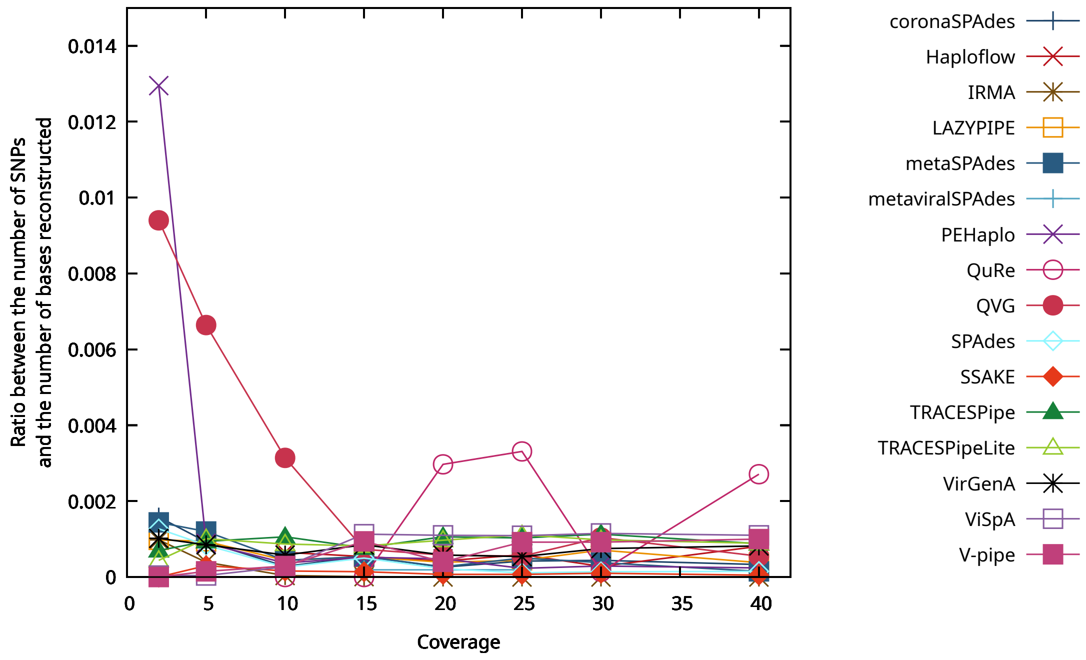

Figure S9: Figure comparing the performance of the reconstruction programs in terms of the ratio between the number of SNPs and the number of bases reconstructed for datasets without contamination or mitochondrial DNA (DS1 to DS8).

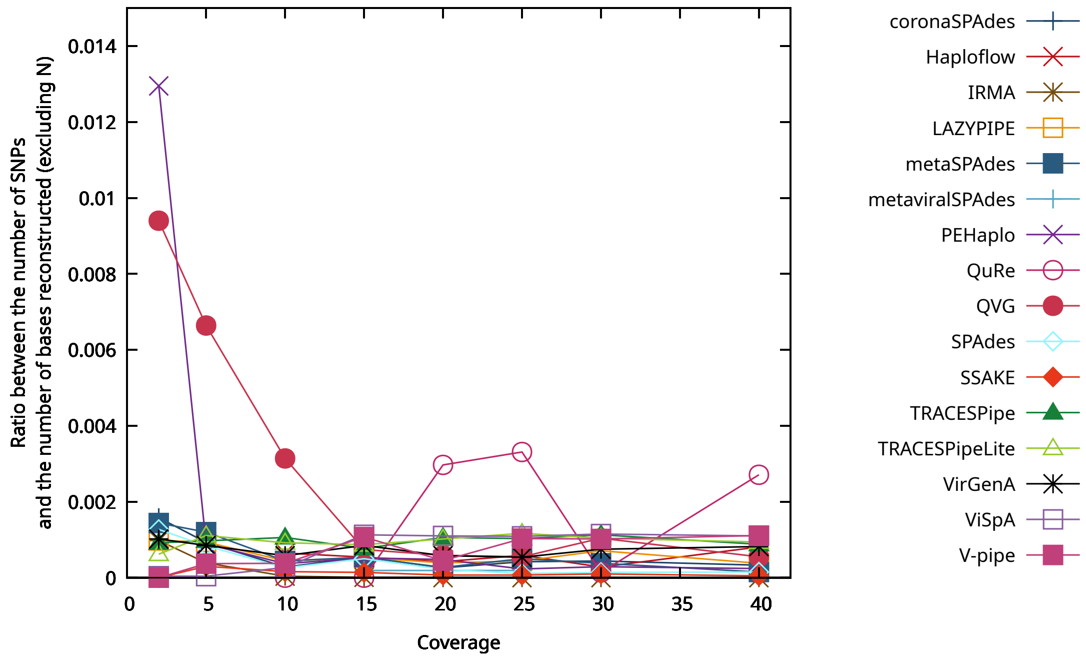

Figure S10: Figure comparing the performance of the reconstruction programs in terms of the ratio between the number of SNPs and the number of bases reconstructed (excluding "N") for datasets without contamination or mitochondrial DNA (DS1 to DS8).

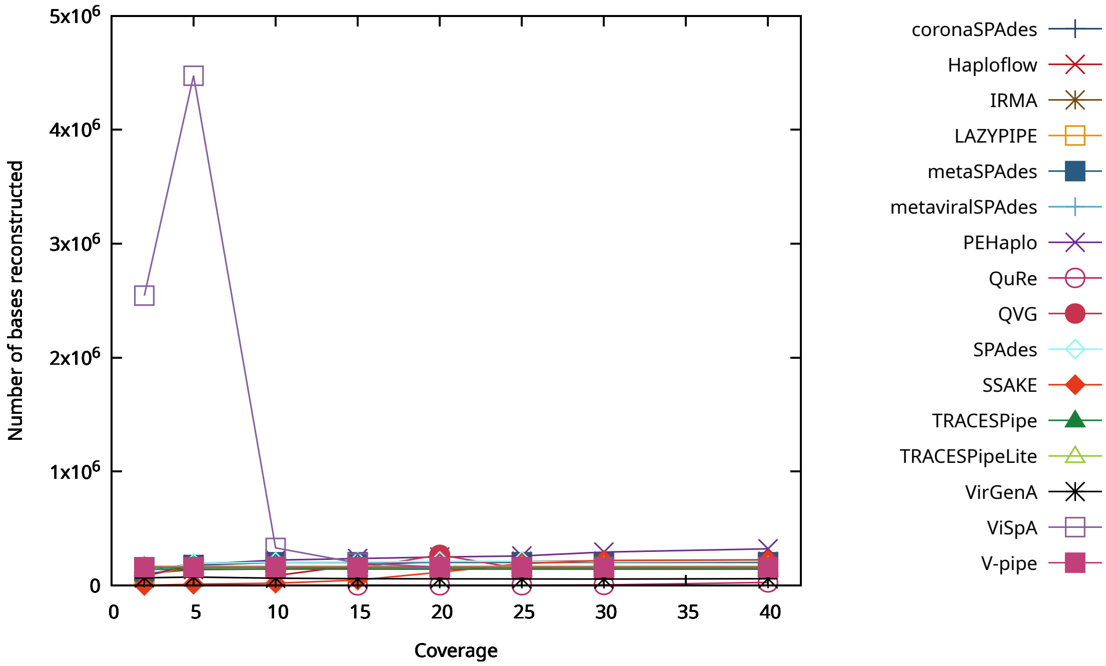

Figure S11: Figure comparing the performance of the reconstruction programs in terms of the number of bases reconstructed for datasets with contamination and mitochondrial DNA (DS9 to DS16).

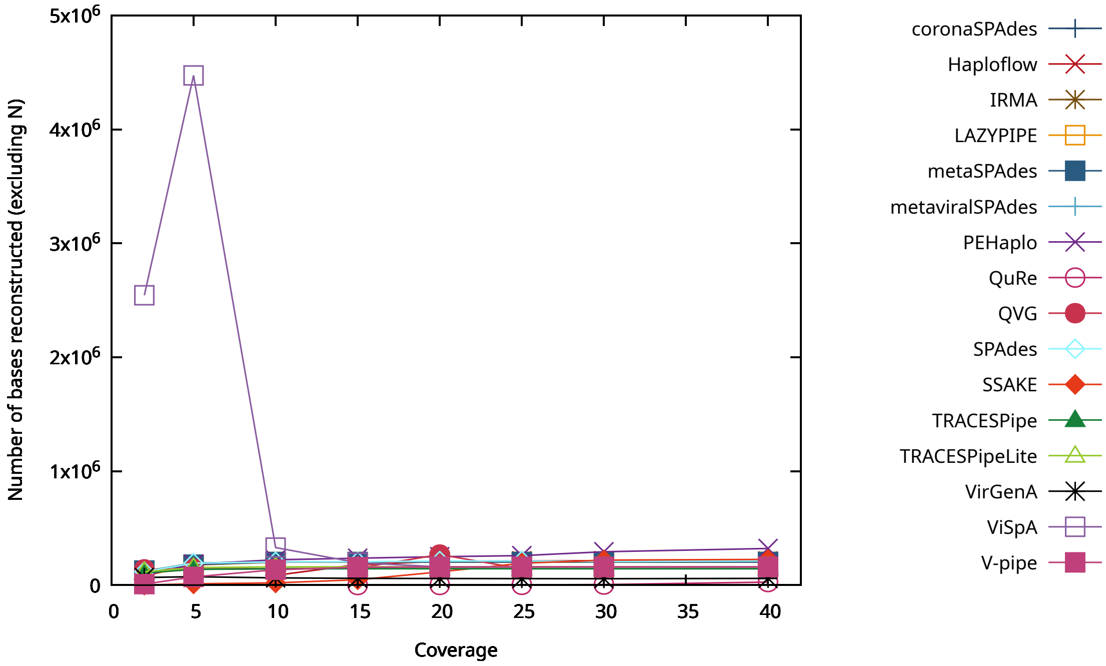

Figure S12: Figure comparing the performance of the reconstruction programs in terms of the number of bases reconstructed (excluding "N") for datasets with contamination and mitochondrial DNA (DS9 to DS16).

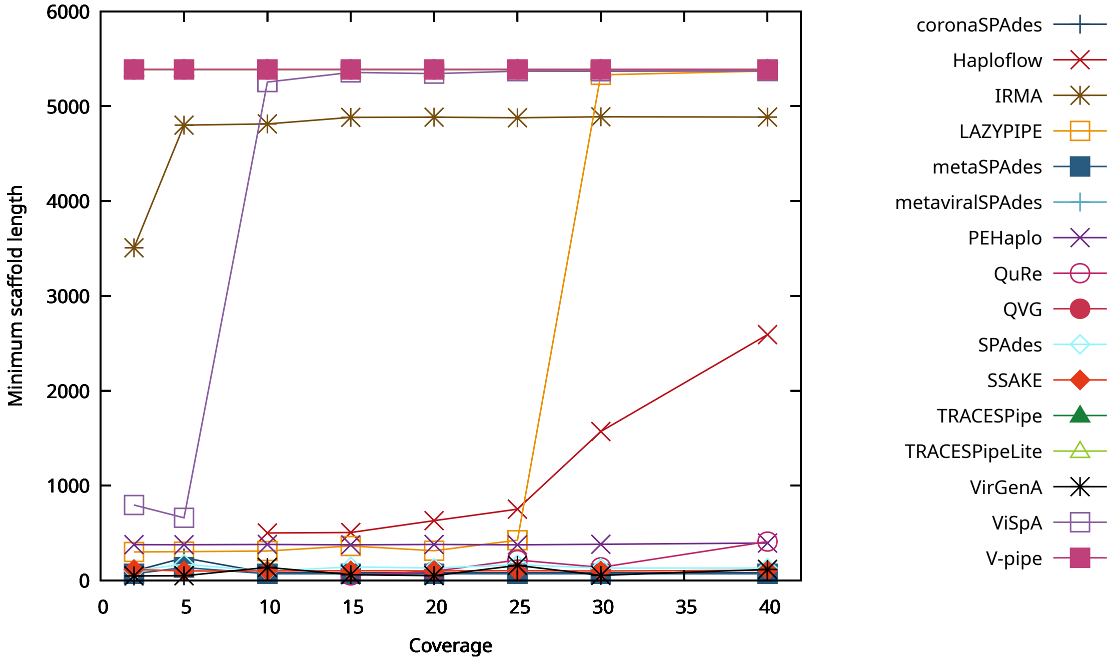

Figure S13: Figure comparing the performance of the reconstruction programs in terms of the minimum number of reconstructed bases per scaffold for datasets with contamination and mitochondrial DNA (DS9 to DS16).

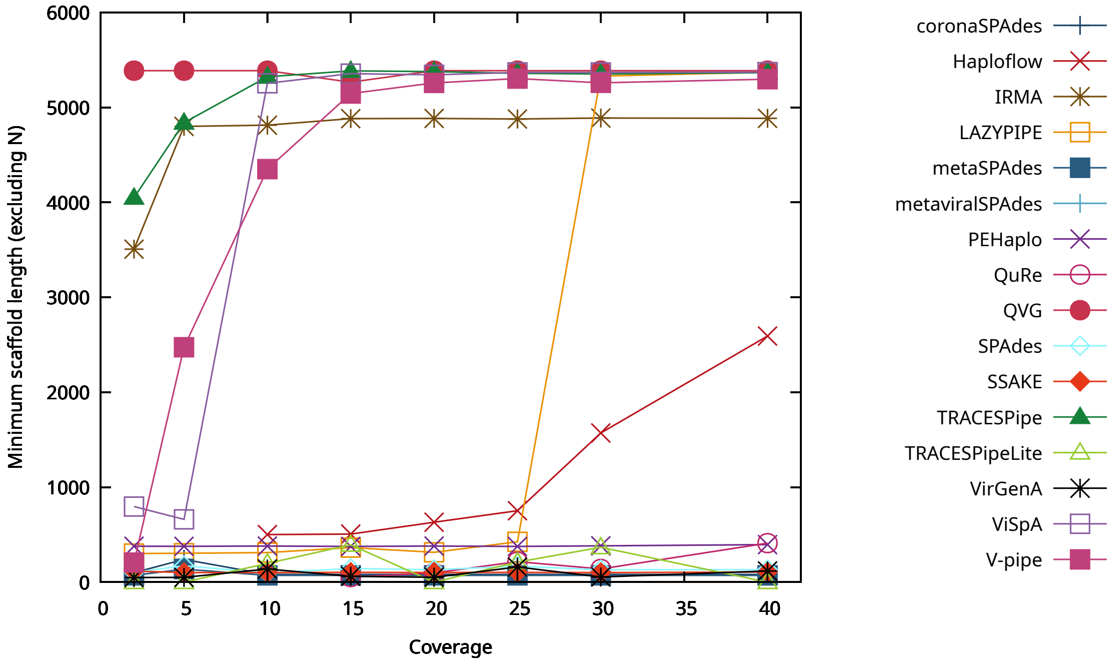

Figure S14: Figure comparing the performance of the reconstruction programs in terms of the minimum number of reconstructed bases per scaffold (excluding "N") for datasets with contamination and mitochondrial DNA (DS9 to DS16).

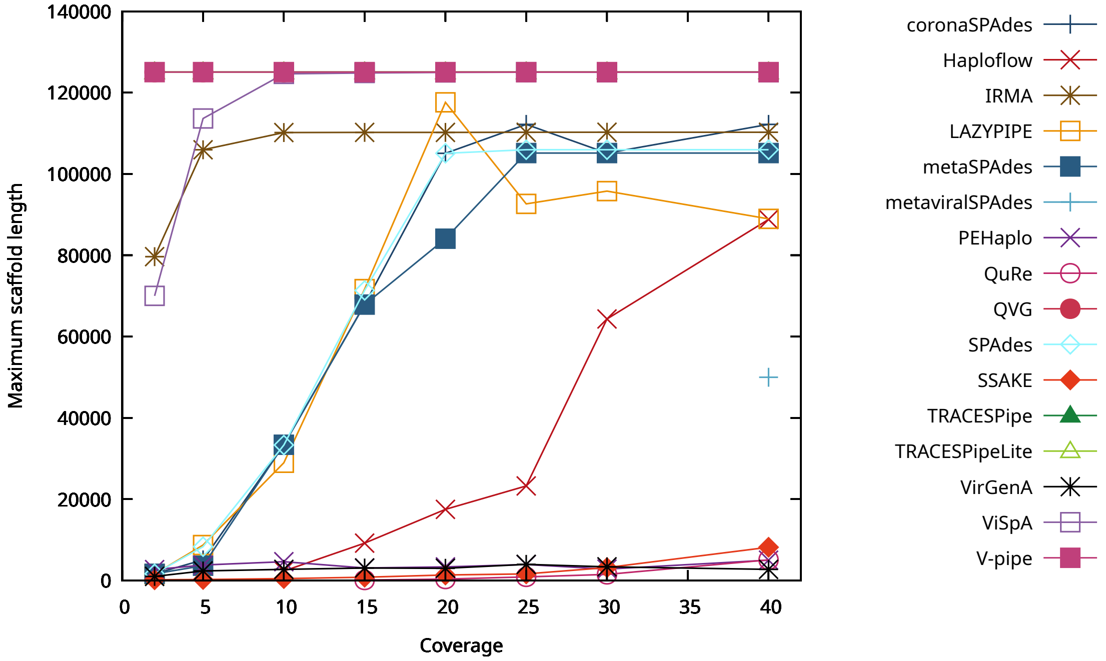

Figure S15: Figure comparing the performance of the reconstruction programs in terms of the maximum number of reconstructed bases per scaffold for datasets with contamination and mitochondrial DNA (DS9 to DS16).

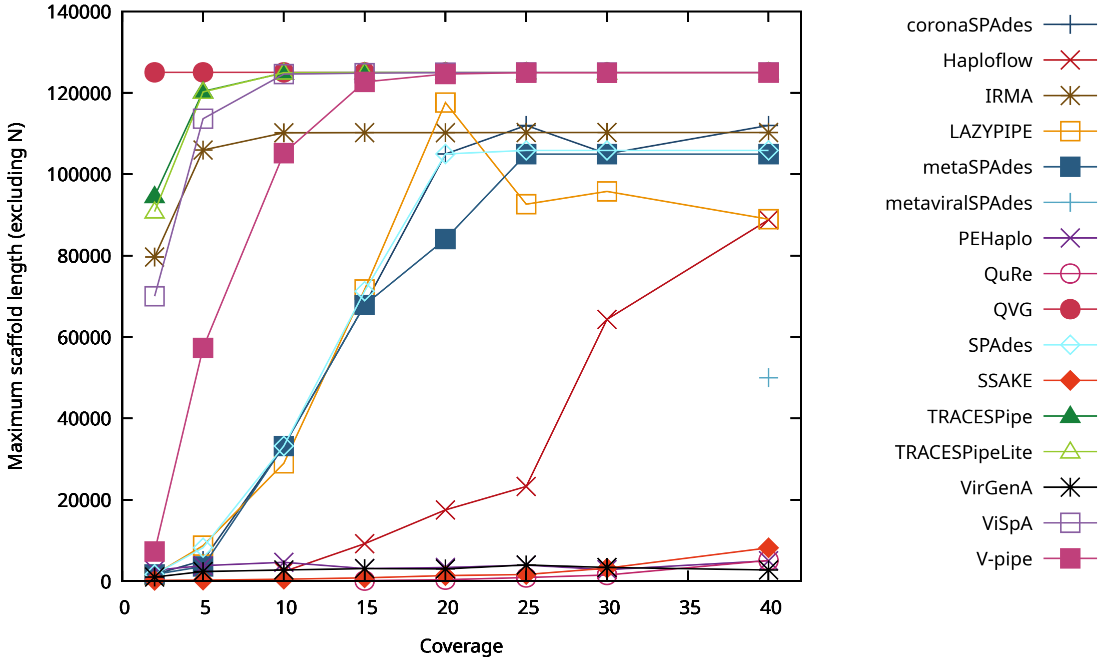

Figure S16: Figure comparing the performance of the reconstruction programs in terms of the maximum number of reconstructed bases per scaffold (excluding "N") for datasets with contamination and mitochondrial DNA (DS9 to DS16).

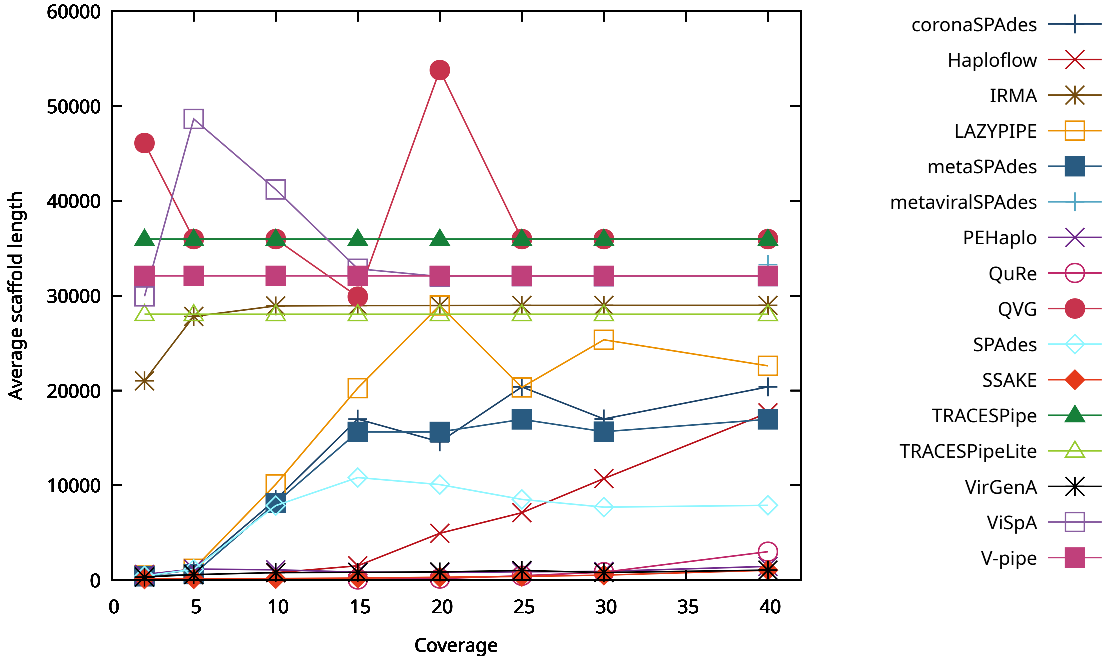

Figure S17: Figure comparing the performance of the reconstruction programs in terms of the average number of reconstructed bases per scaffold for datasets with contamination and mitochondrial DNA (DS9 to DS16).

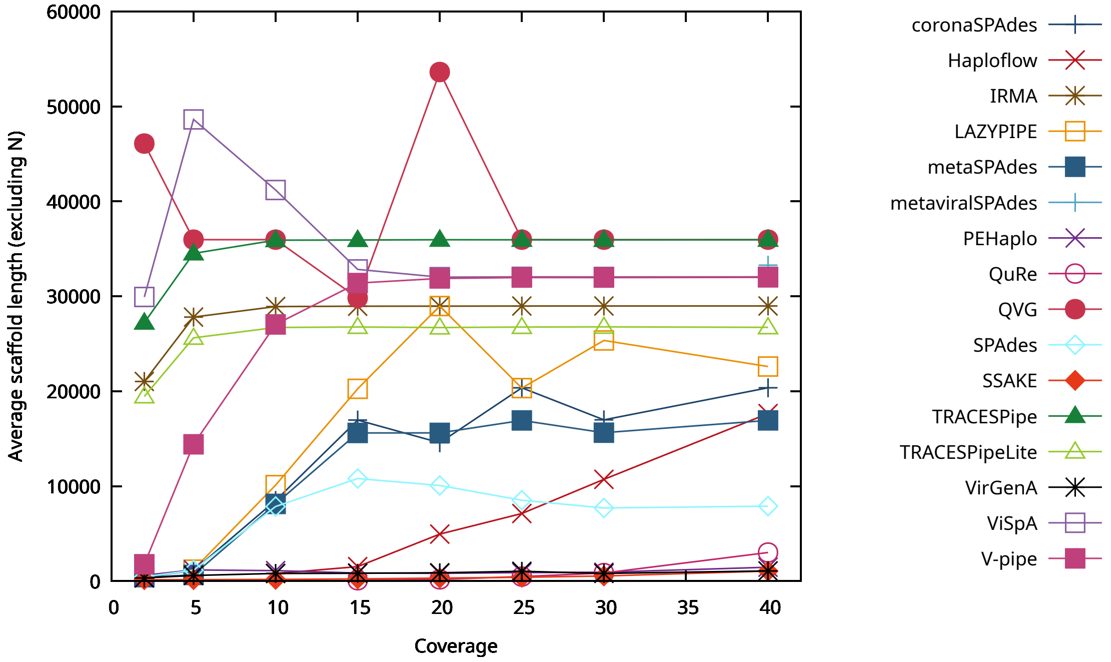

Figure S18: Figure comparing the performance of the reconstruction programs in terms of the average number of reconstructed bases per scaffold (excluding "N") for datasets with contamination and mitochondrial DNA (DS9 to DS16).

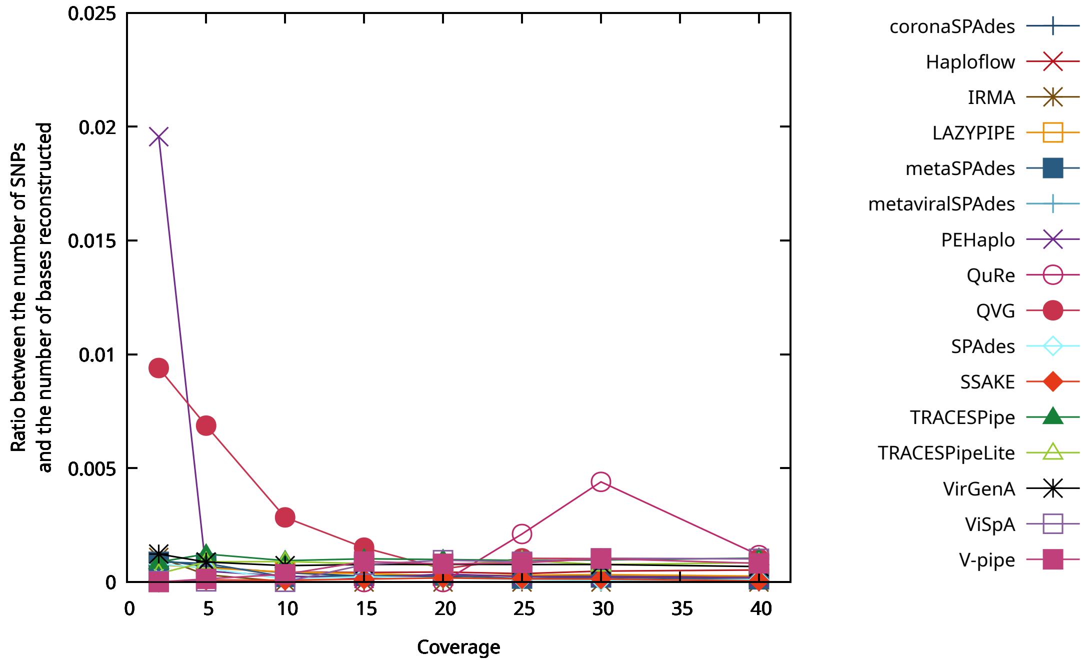

Figure S19: Figure comparing the performance of the reconstruction programs in terms of the ratio between the number of SNPs and the number of bases reconstructed for datasets with contamination and mitochondrial DNA (DS9 to DS16).

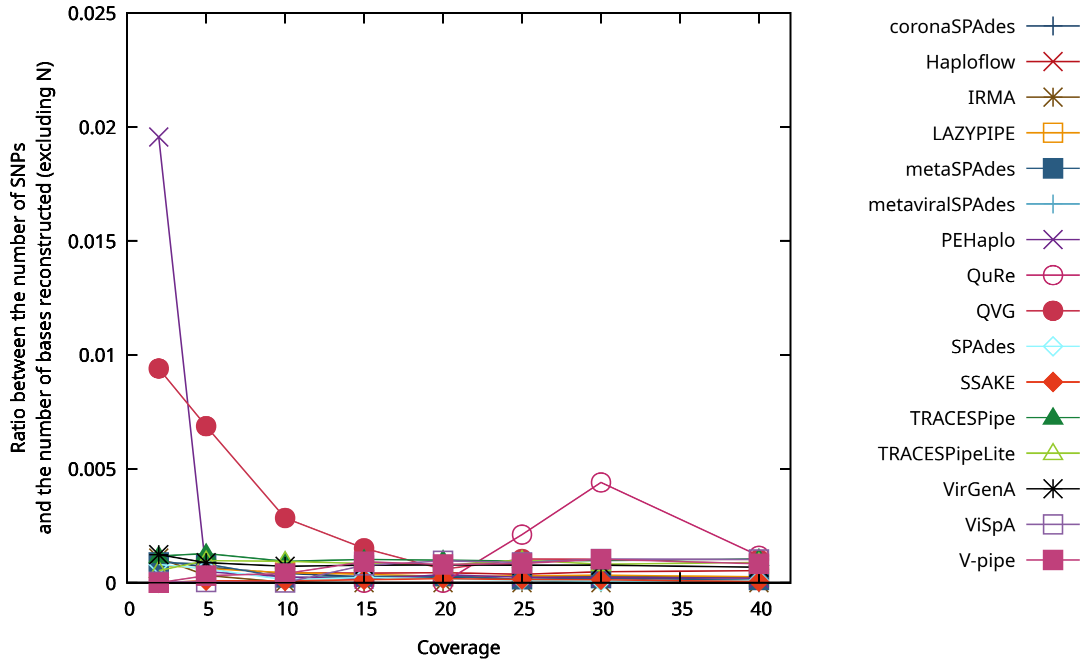

Figure S20: Figure comparing the performance of the reconstruction programs in terms of the ratio between the number of SNPs and the number of bases reconstructed (excluding "N") for datasets with contamination and mitochondrial DNA (DS9 to DS16).

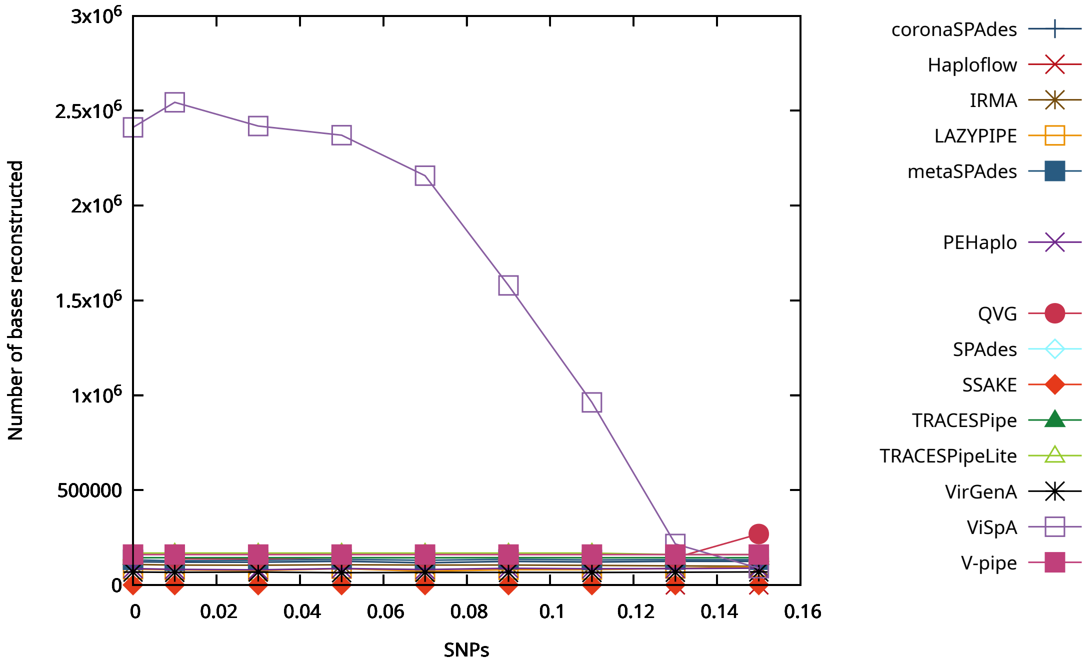

Figure S21: Figure comparing the performance of the reconstruction programs in terms of the number of bases reconstructed for datasets with depth coverage equal to 2x (DS17 to DS24 plus DS9).

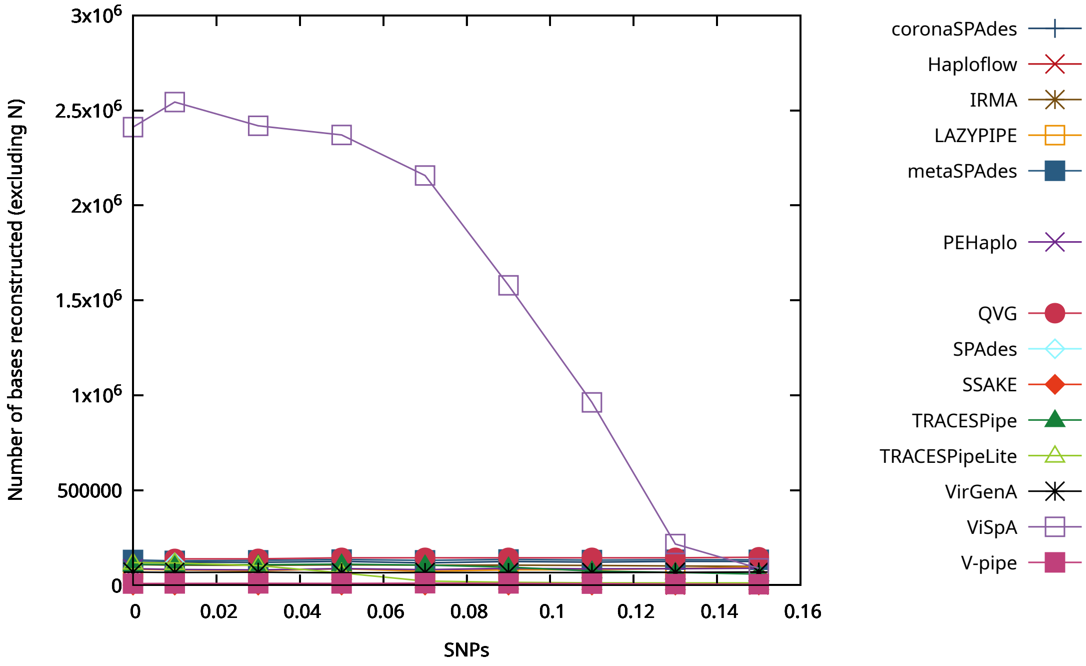

Figure S22: Figure comparing the performance of the reconstruction programs in terms of the number of bases reconstructed (excluding "N") for datasets with depth coverage equal to 2x (DS17 to DS24 plus DS9).

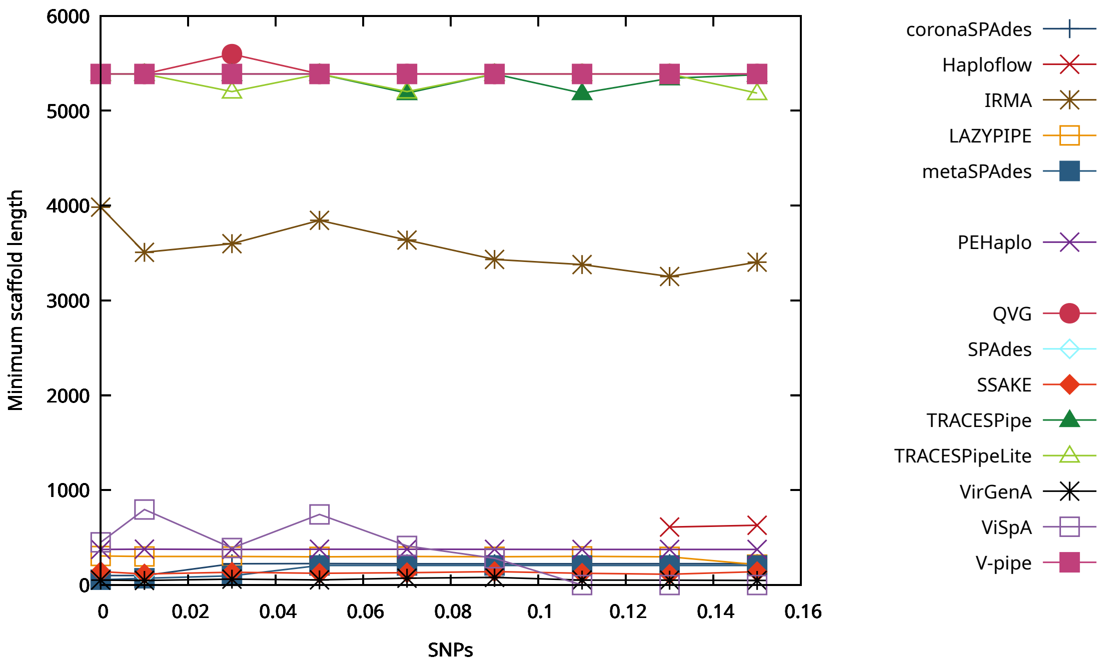

Figure S23: Figure comparing the performance of the reconstruction programs in terms of the minimum number of reconstructed bases per scaffold for datasets with depth coverage equal to 2x (DS17 to DS24 plus DS9).

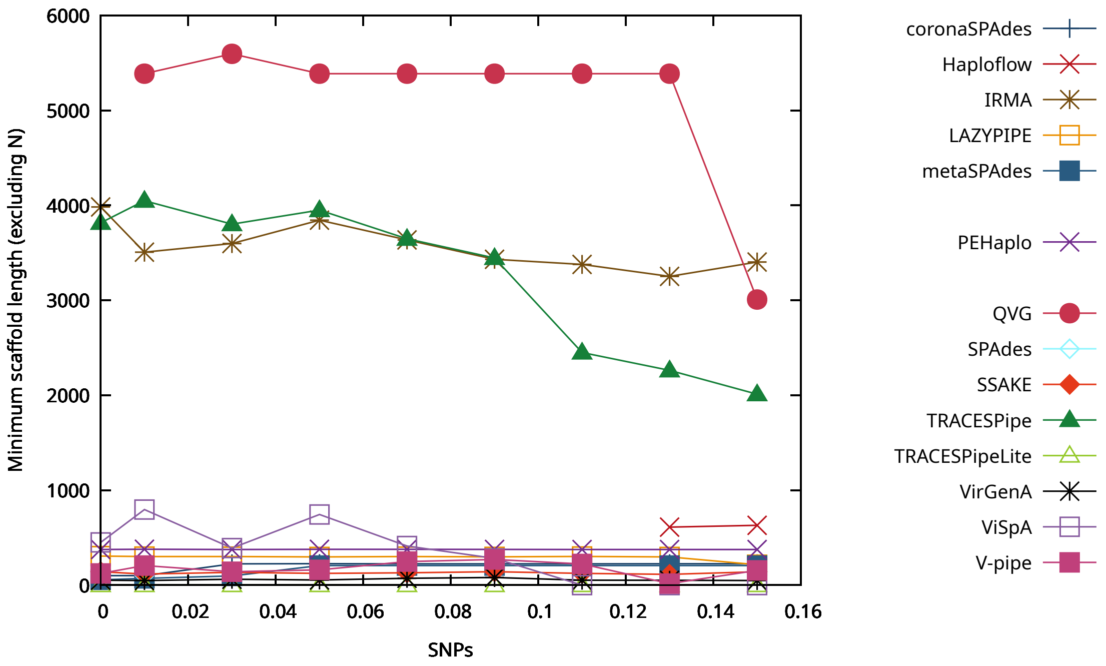

Figure S24: Figure comparing the performance of the reconstruction programs in terms of the minimum number of reconstructed bases per scaffold (excluding "N") for datasets with depth coverage equal to 2x (DS17 to DS24 plus DS9).

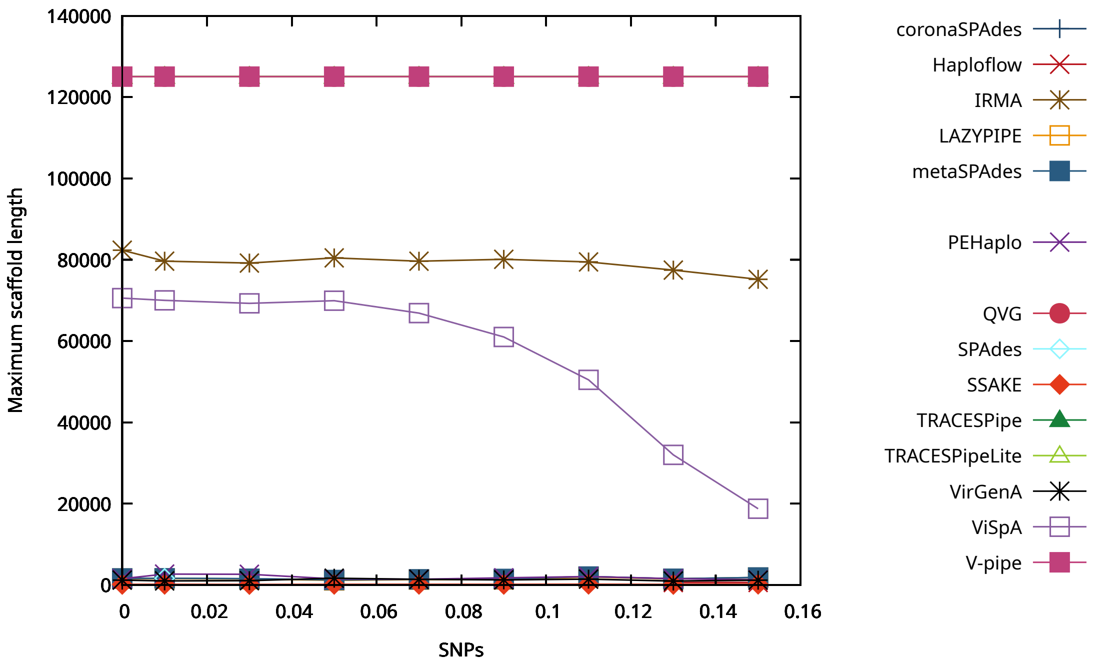

Figure S25: Figure comparing the performance of the reconstruction programs in terms of the maximum number of reconstructed bases per scaffold for datasets with depth coverage equal to 2x (DS17 to DS24 plus DS9).

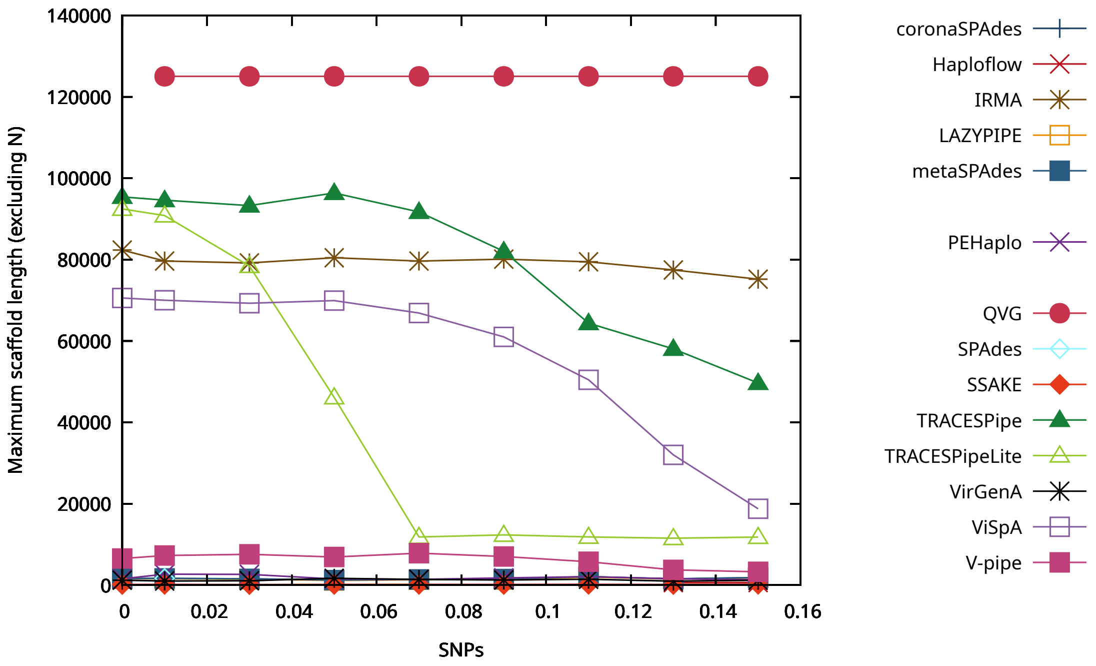

Figure S26: Figure comparing the performance of the reconstruction programs in terms of the maximum number of reconstructed bases per scaffold (excluding "N") for datasets with depth coverage equal to 2x (DS17 to DS24 plus DS9).

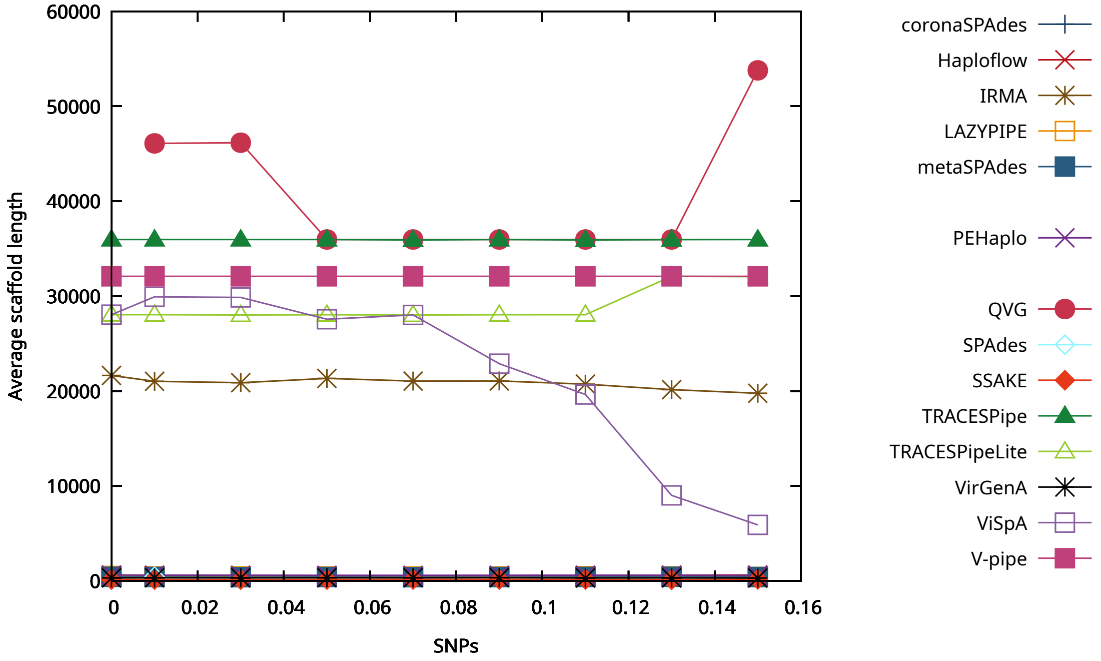

Figure S27: Figure comparing the performance of the reconstruction programs in terms of the average number of reconstructed bases per scaffold for datasets with depth coverage equal to 2x (DS17 to DS24 plus DS9).

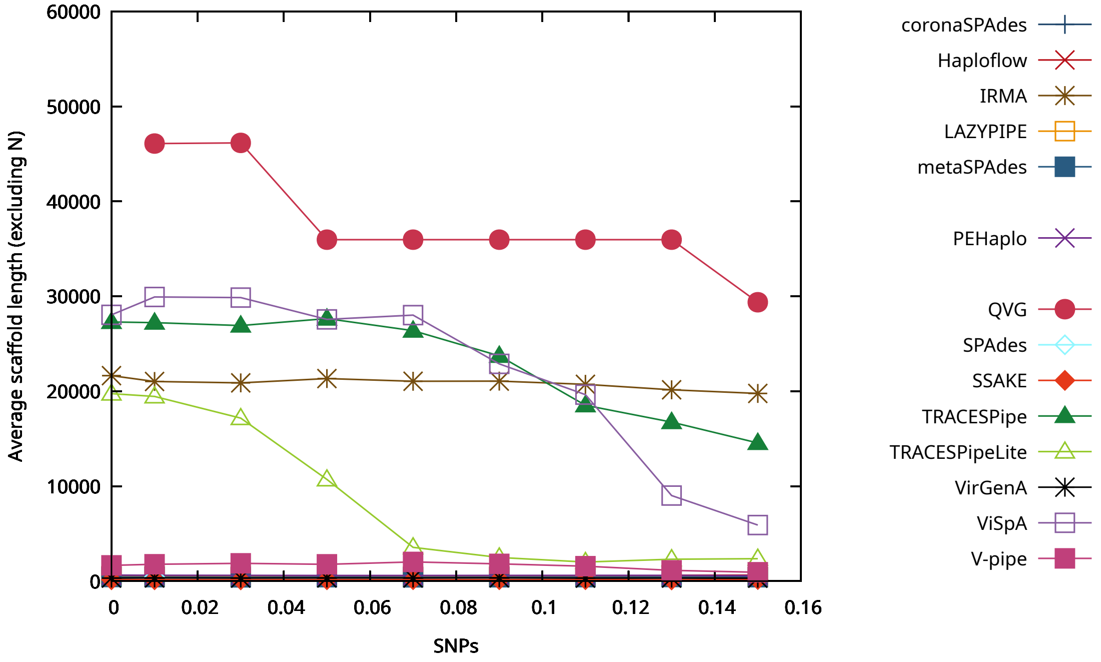

Figure S28: Figure comparing the performance of the reconstruction programs in terms of the average number of reconstructed bases per scaffold (excluding "N") for datasets with depth coverage equal to 2x (DS17 to DS24 plus DS9).

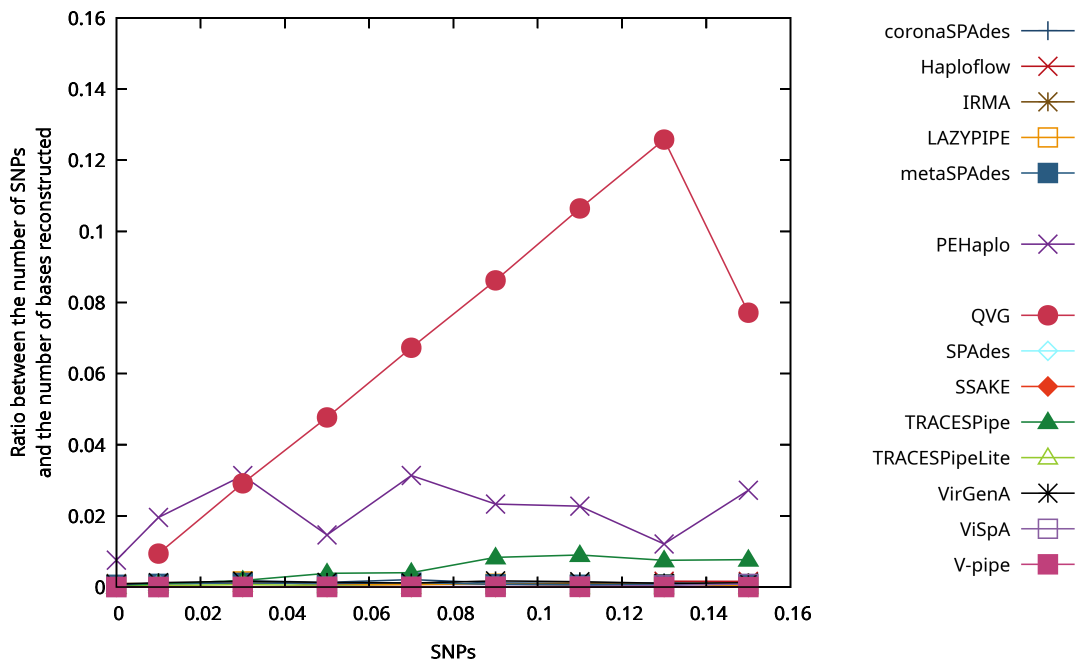

Figure S29: Figure comparing the performance of the reconstruction programs in terms of the ratio between the number of SNPs and the number of bases reconstructed for datasets with depth coverage equal to 2x (DS17 to DS24 plus DS9).

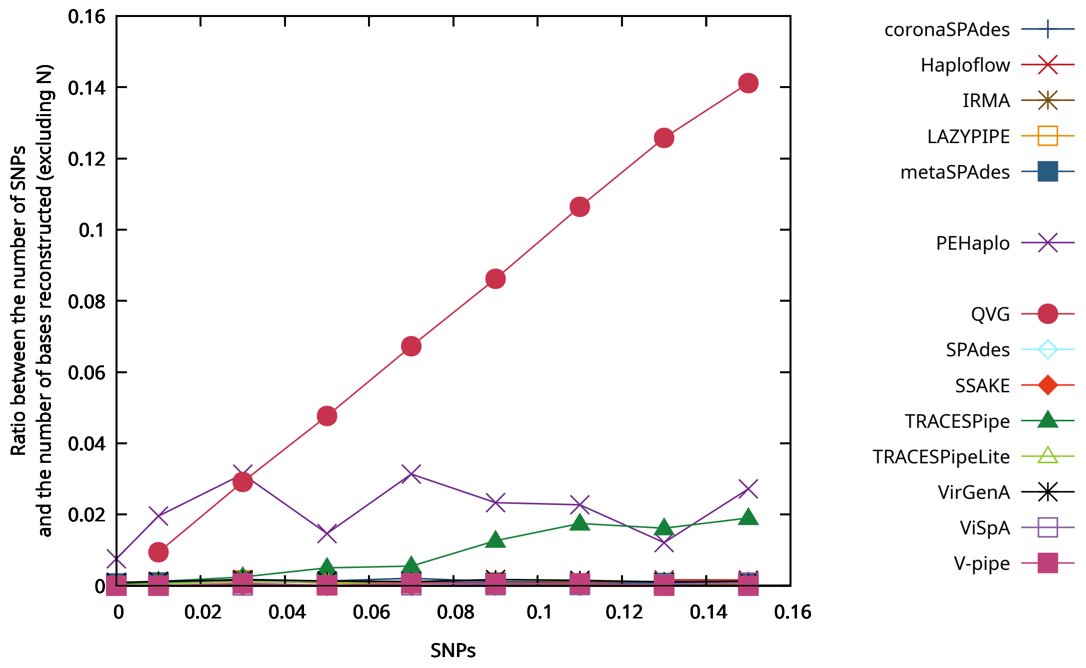

Figure S30: Figure comparing the performance of the reconstruction programs in terms of the ratio between the number of SNPs and the number of bases reconstructed (excluding "N") for datasets with depth coverage equal to 2x (DS17 to DS24 plus DS9).

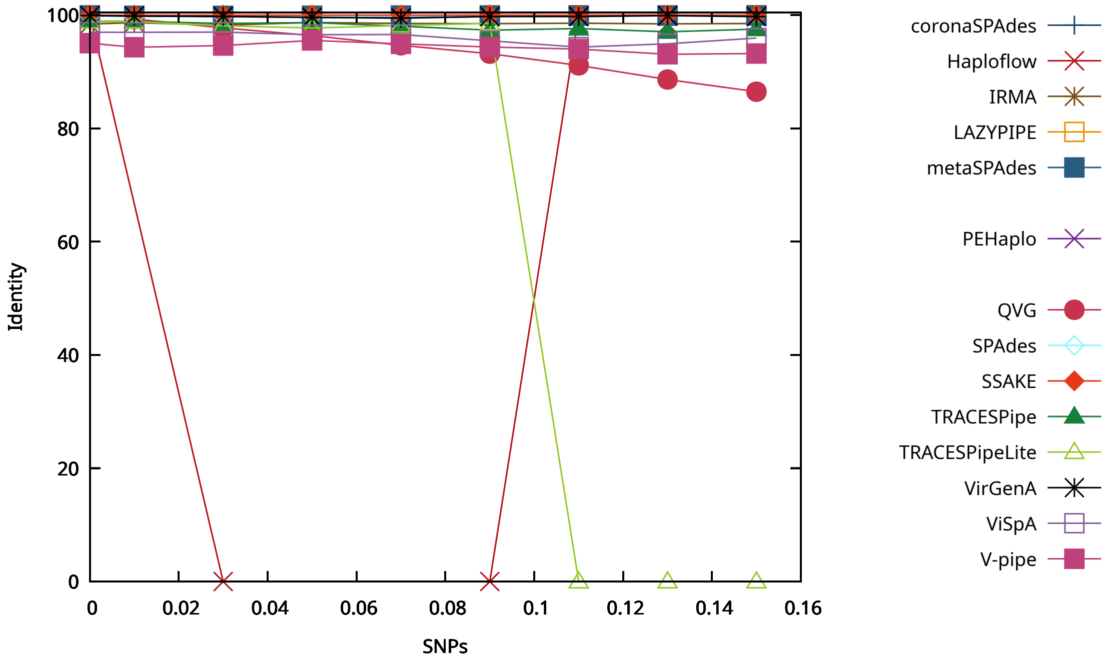

Figure S31: Figure comparing the performance of the reconstruction programs in terms of the identity for datasets with depth coverage equal to 5x (DS25 to DS32 plus DS10).

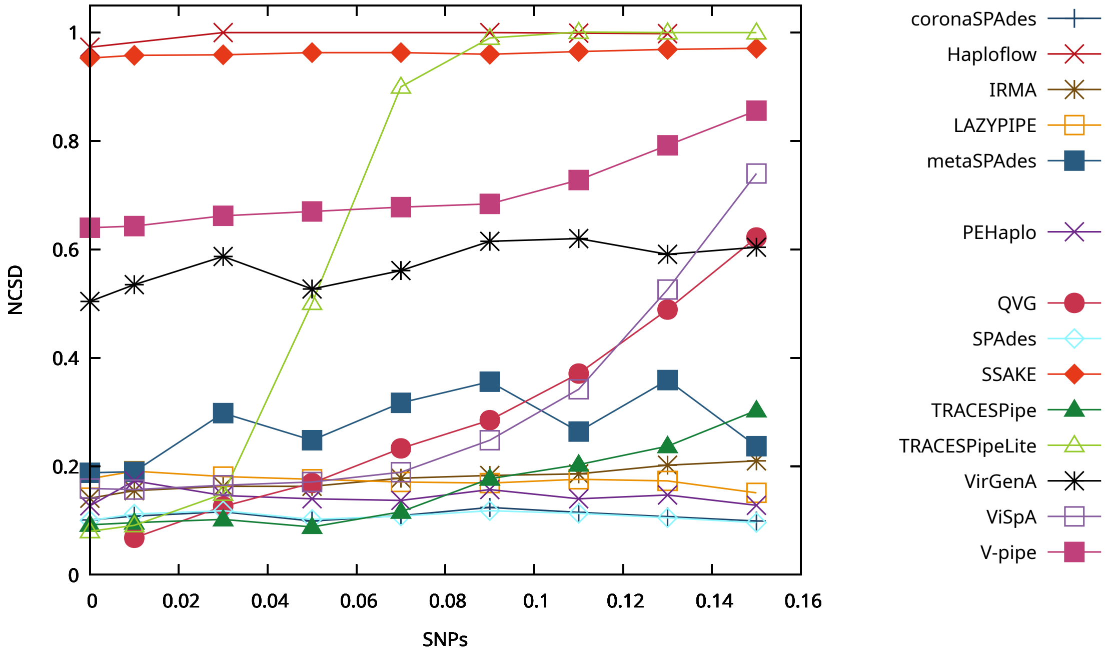

Figure S32: Figure comparing the performance of the reconstruction programs in terms of the NCSD for datasets with depth coverage equal to 5x (DS25 to DS32 plus DS10).

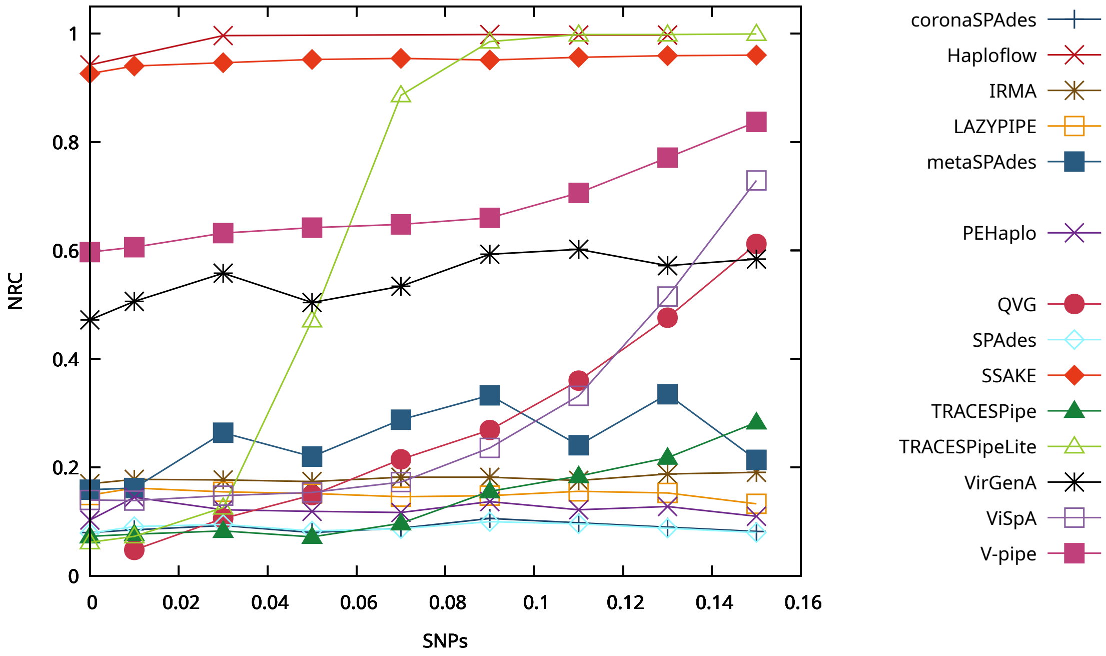

Figure S33: Figure comparing the performance of the reconstruction programs in terms of the NRC for datasets with depth coverage equal to 5x (DS25 to DS32 plus DS10).

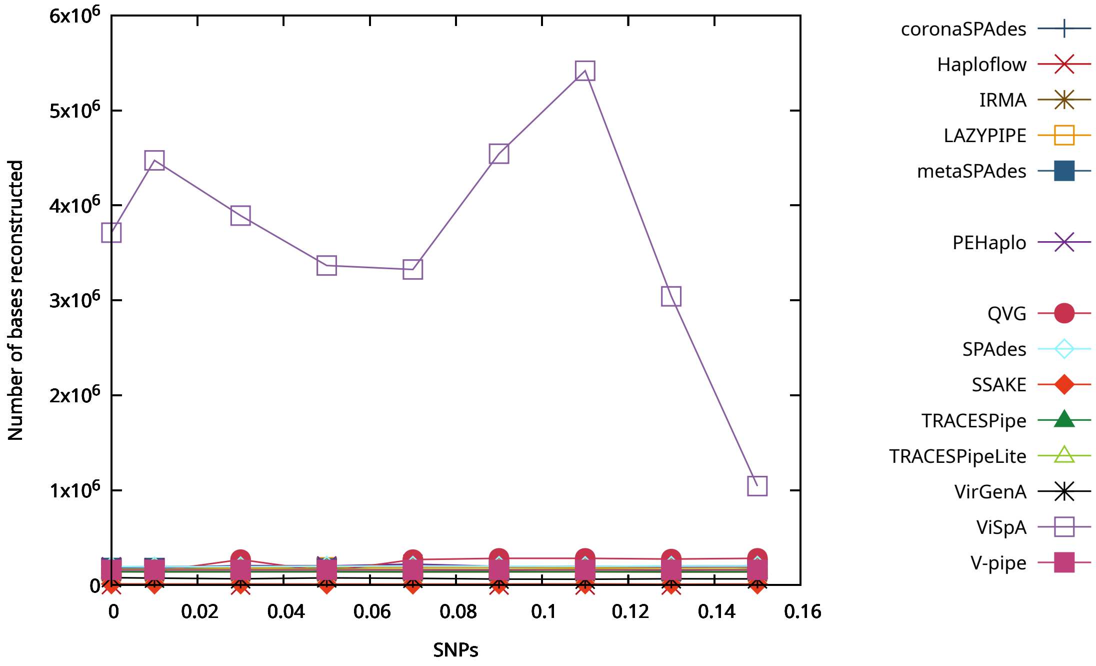

Figure S34: Figure comparing the performance of the reconstruction programs in terms of the number of bases reconstructed for datasets with depth coverage equal to 5x (DS25 to DS32 plus DS10).

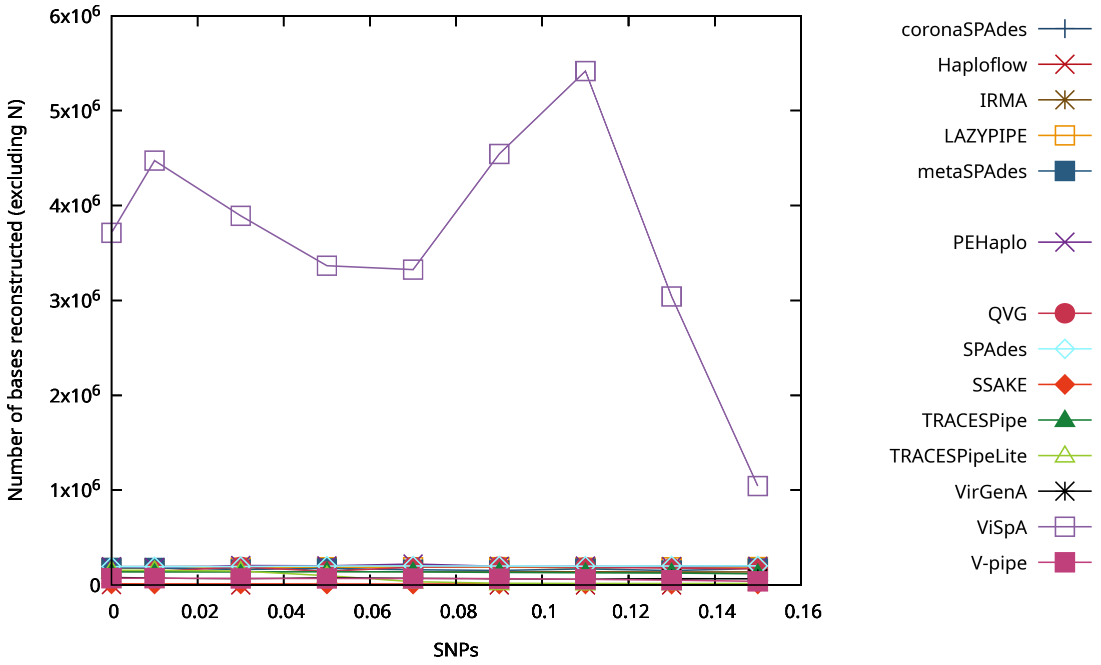

Figure S35: Figure comparing the performance of the reconstruction programs in terms of the number of bases reconstructed (excluding "N") for datasets with depth coverage equal to 5x (DS25 to DS32 plus DS10).

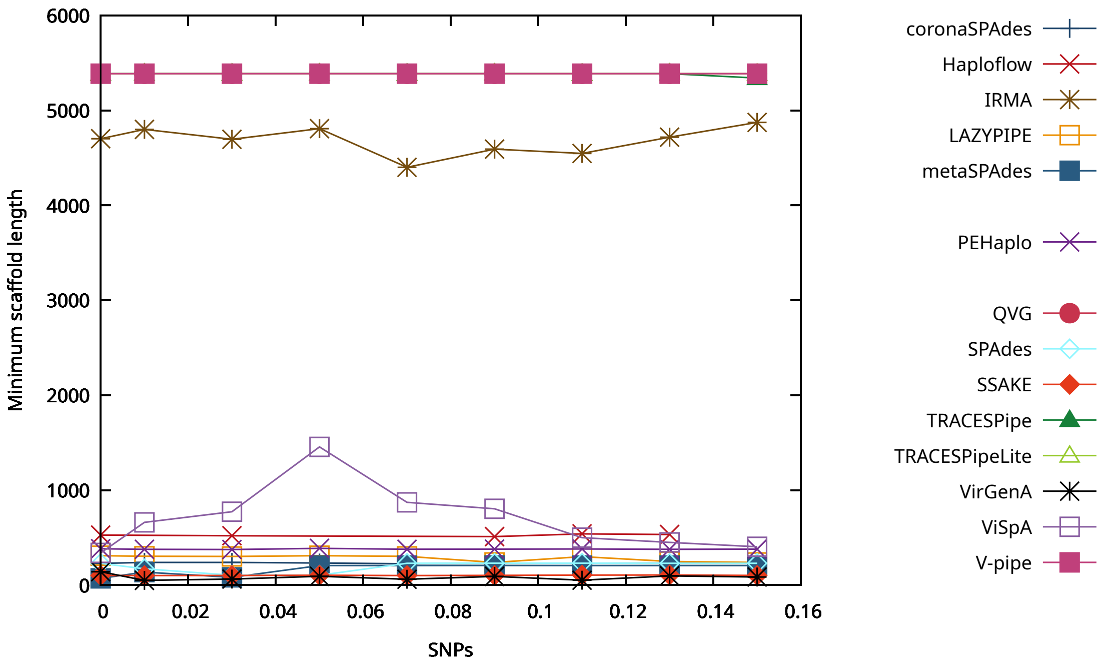

Figure S36: Figure comparing the performance of the reconstruction programs in terms of the minimum number of reconstructed bases per scaffold for datasets with depth coverage equal to 5x (DS25 to DS32 plus DS10).

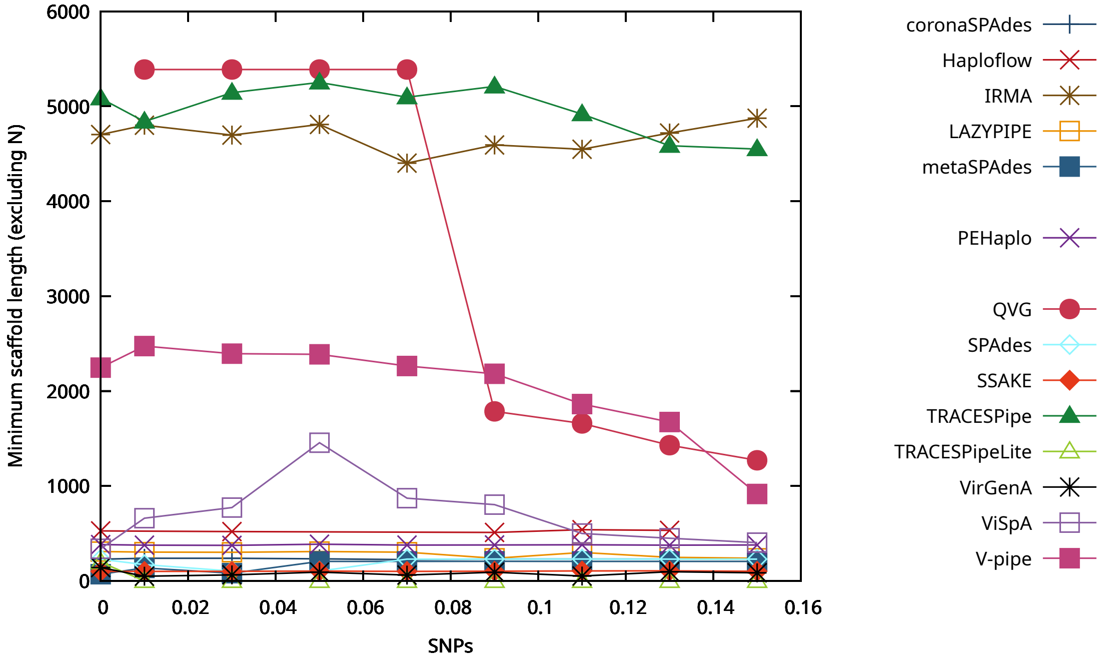

Figure S37: Figure comparing the performance of the reconstruction programs in terms of the minimum number of reconstructed bases per scaffold (excluding "N") for datasets with depth coverage equal to 5x (DS25 to DS32 plus DS10).

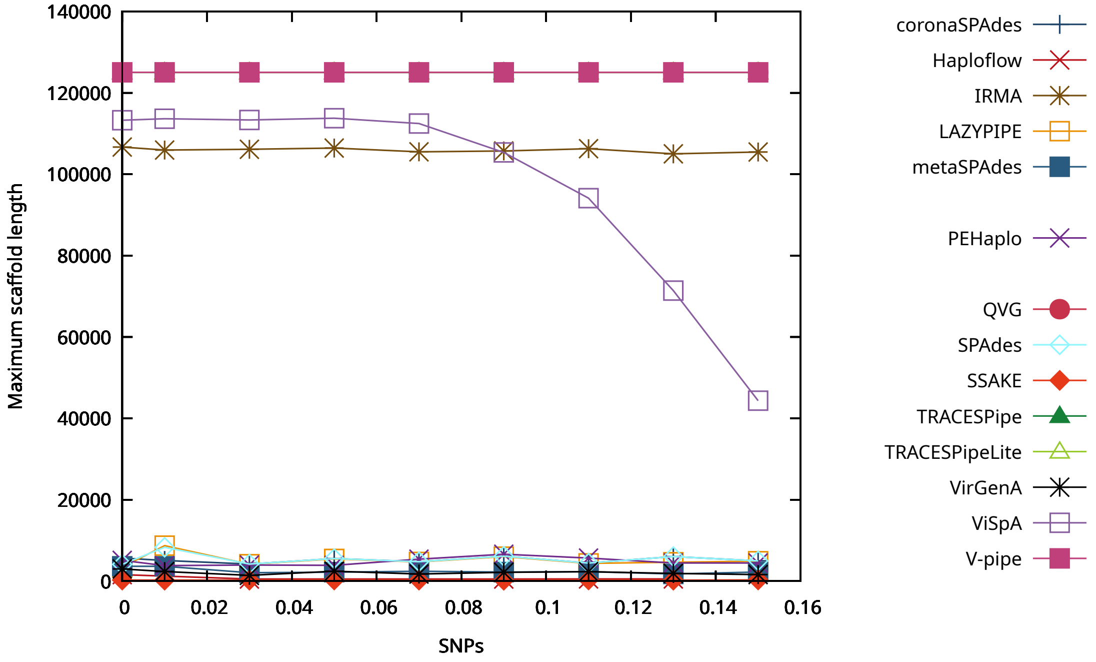

Figure S38: Figure comparing the performance of the reconstruction programs in terms of the maximum number of reconstructed bases per scaffold for datasets with depth coverage equal to 5x (DS25 to DS32 plus DS10).

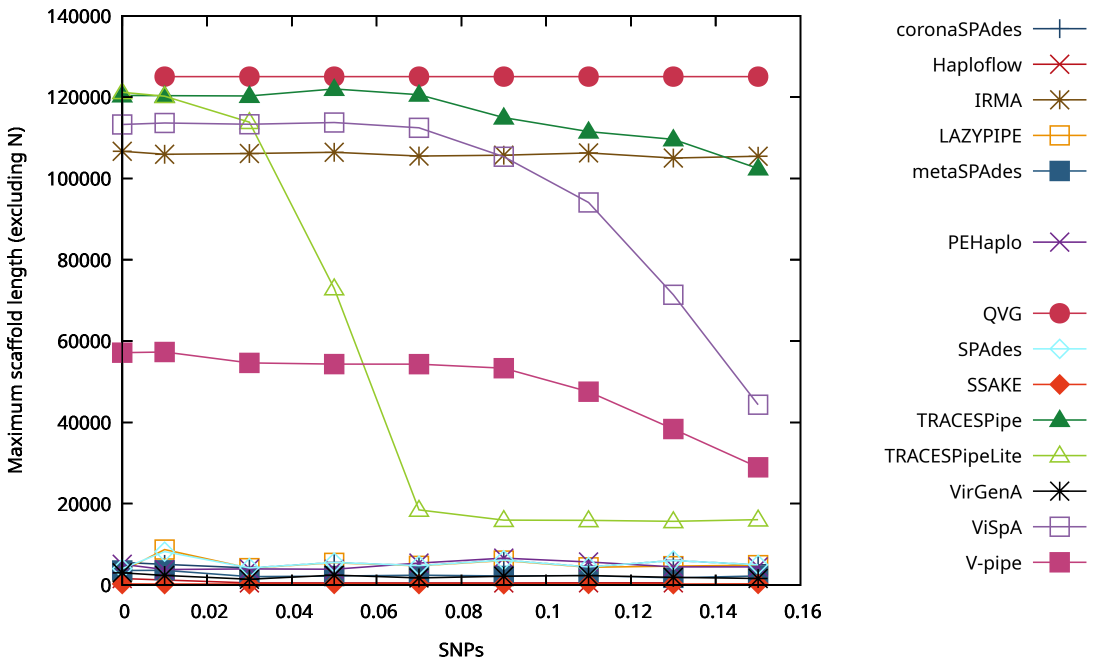

Figure S39: Figure comparing the performance of the reconstruction programs in terms of the maximum number of reconstructed bases per scaffold (excluding "N") for datasets with depth coverage equal to 5x (DS25 to DS32 plus DS10).

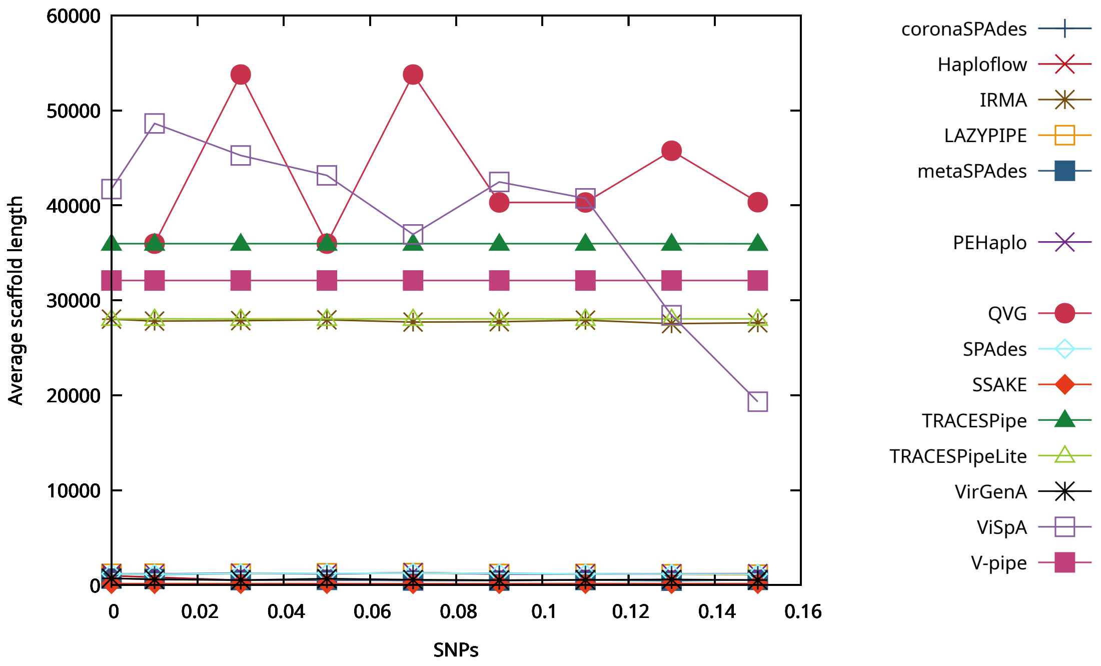

Figure S40: Figure comparing the performance of the reconstruction programs in terms of the average number of reconstructed bases per scaffold for datasets with depth coverage equal to 5x (DS25 to DS32 plus DS10).

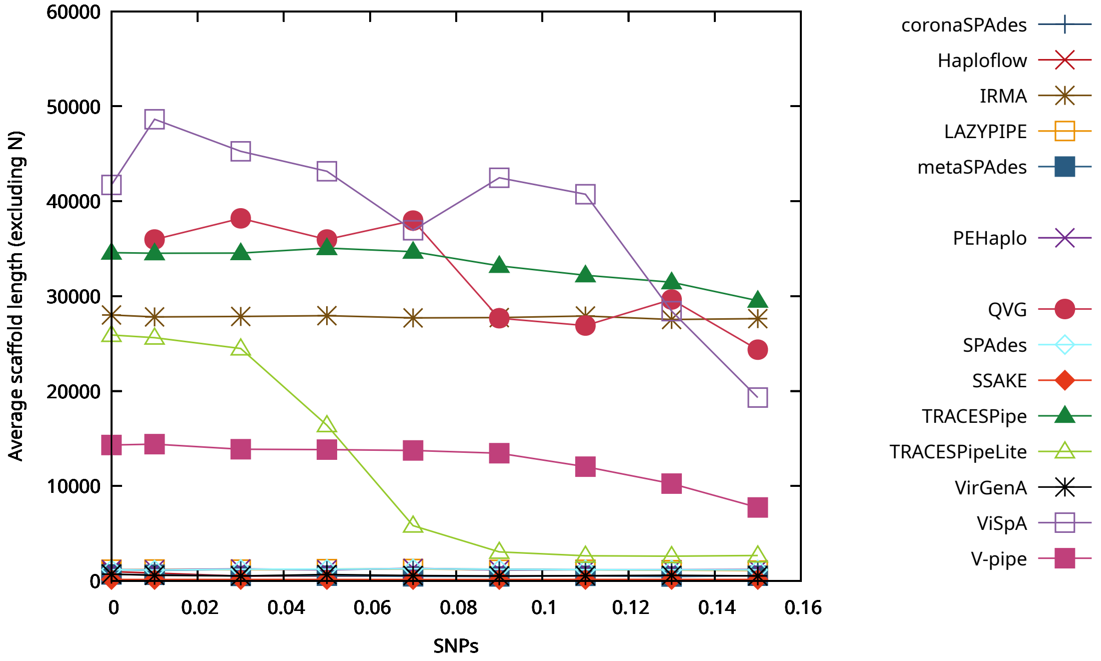

Figure S41: Figure comparing the performance of the reconstruction programs in terms of the average number of reconstructed bases per scaffold (excluding "N") for datasets with depth coverage equal to 5x (DS25 to DS32 plus DS10).

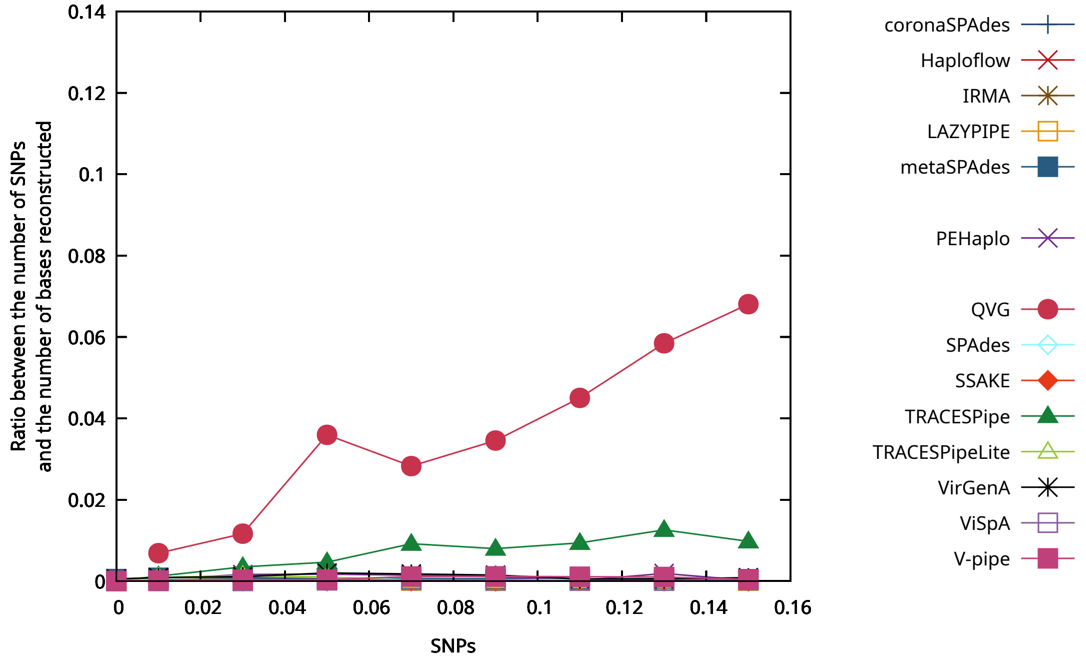

Figure S42: Figure comparing the performance of the reconstruction programs in terms of the ratio between the number of SNPs and the number of bases reconstructed for datasets with depth coverage equal to 5x (DS25 to DS32 plus DS10).

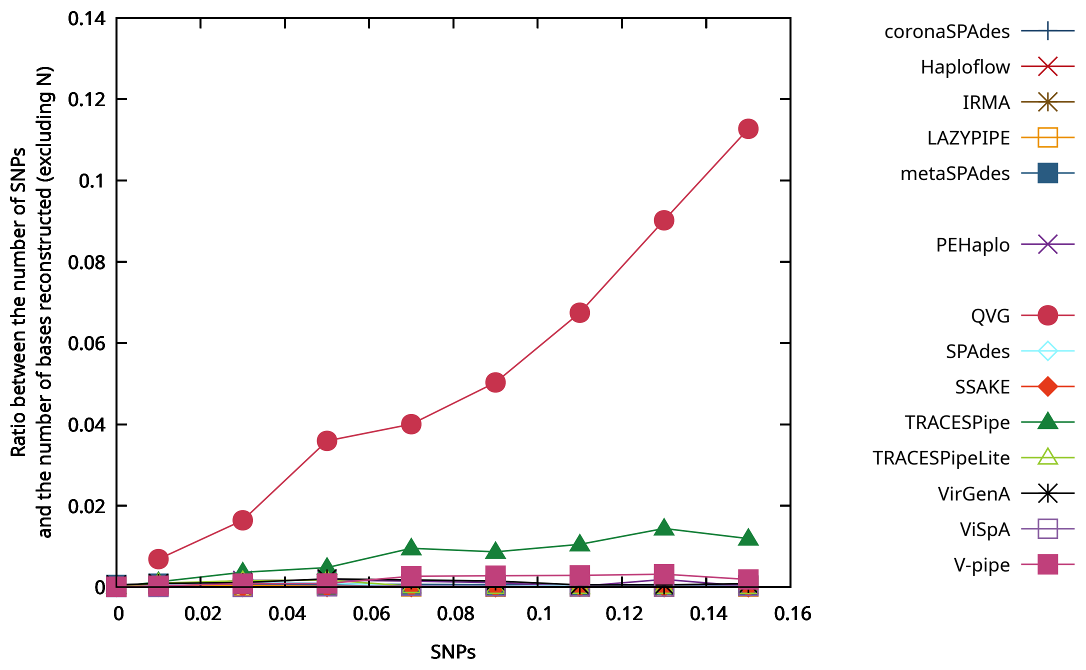

Figure S43: Figure comparing the performance of the reconstruction programs in terms of the ratio between the number of SNPs and the number of bases reconstructed (excluding "N") for datasets with depth coverage equal to 5x (DS25 to DS32 plus DS10).

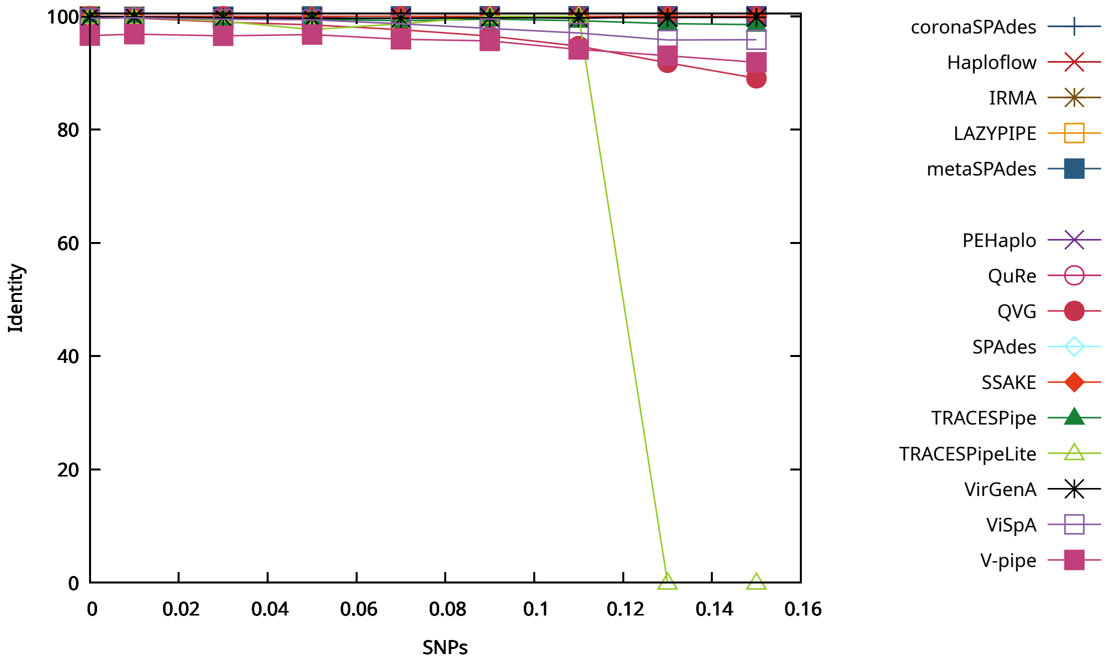

Figure S44: Figure comparing the performance of the reconstruction programs in terms of the identity for datasets with depth coverage equal to 10x (DS33 to DS40 plus DS11).

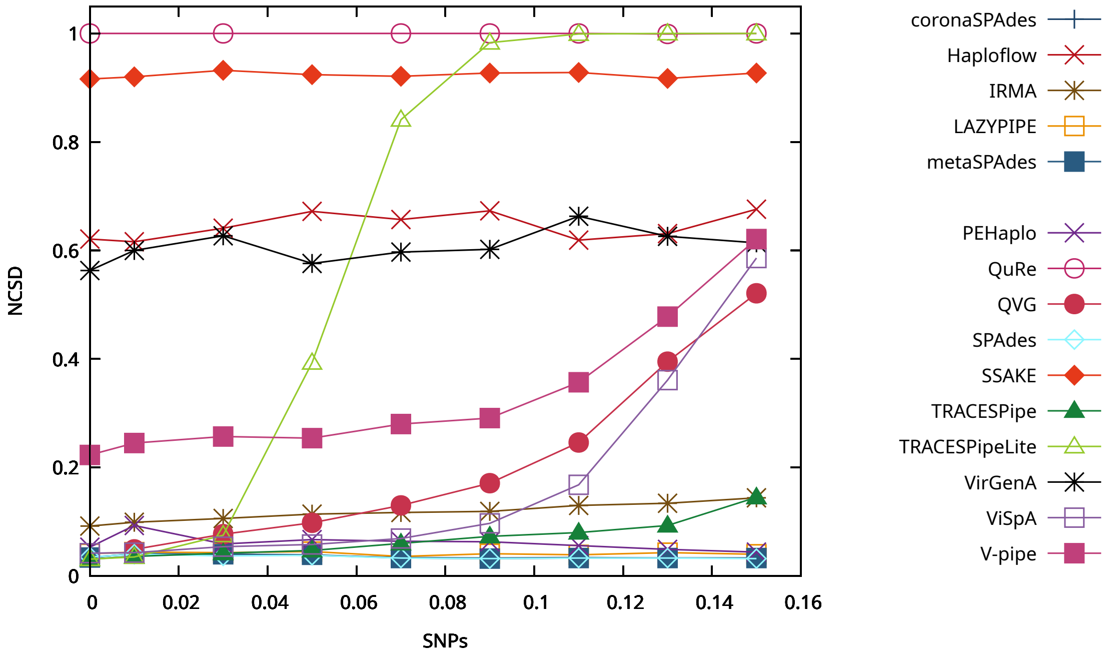

Figure S45: Figure comparing the performance of the reconstruction programs in terms of the NCSD for datasets with depth coverage equal to 10x (DS33 to DS40 plus DS11).

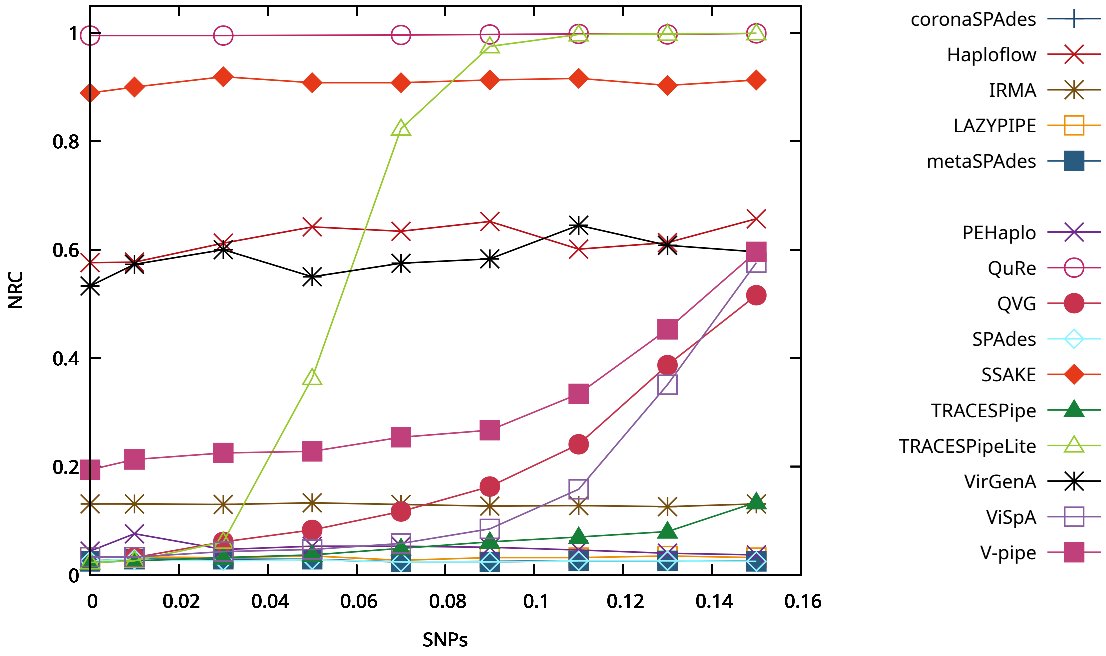

Figure S46: Figure comparing the performance of the reconstruction programs in terms of the NRC for datasets with depth coverage equal to 10x (DS33 to DS40 plus DS11).

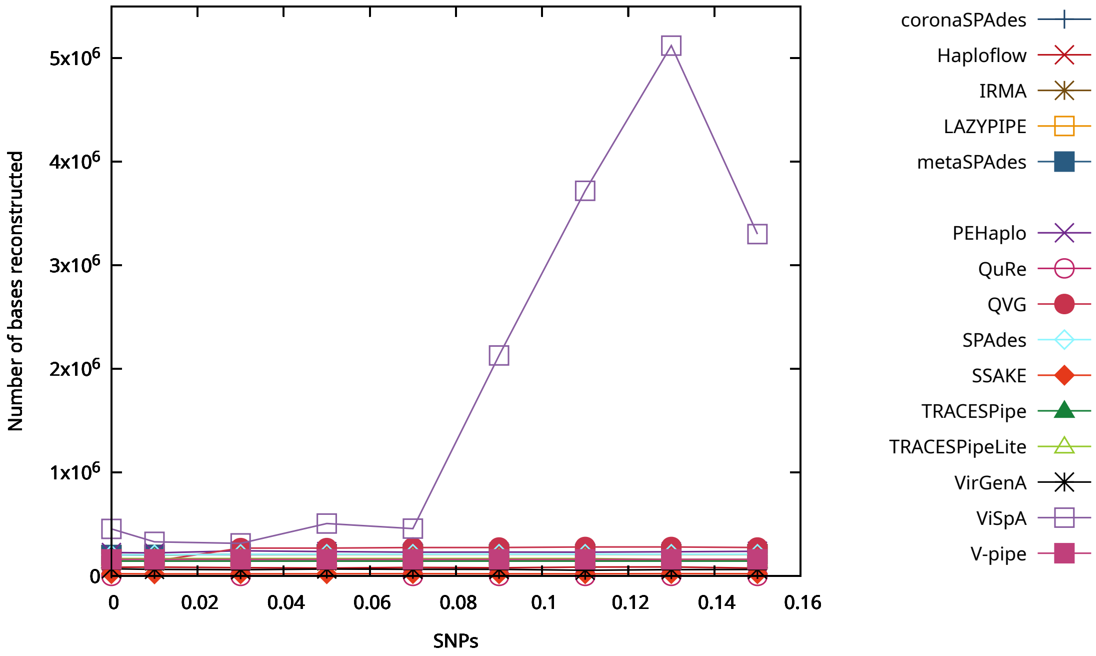

Figure S47: Figure comparing the performance of the reconstruction programs in terms of the number of bases reconstructed for datasets with depth coverage equal to 10x (DS33 to DS40 plus DS11).

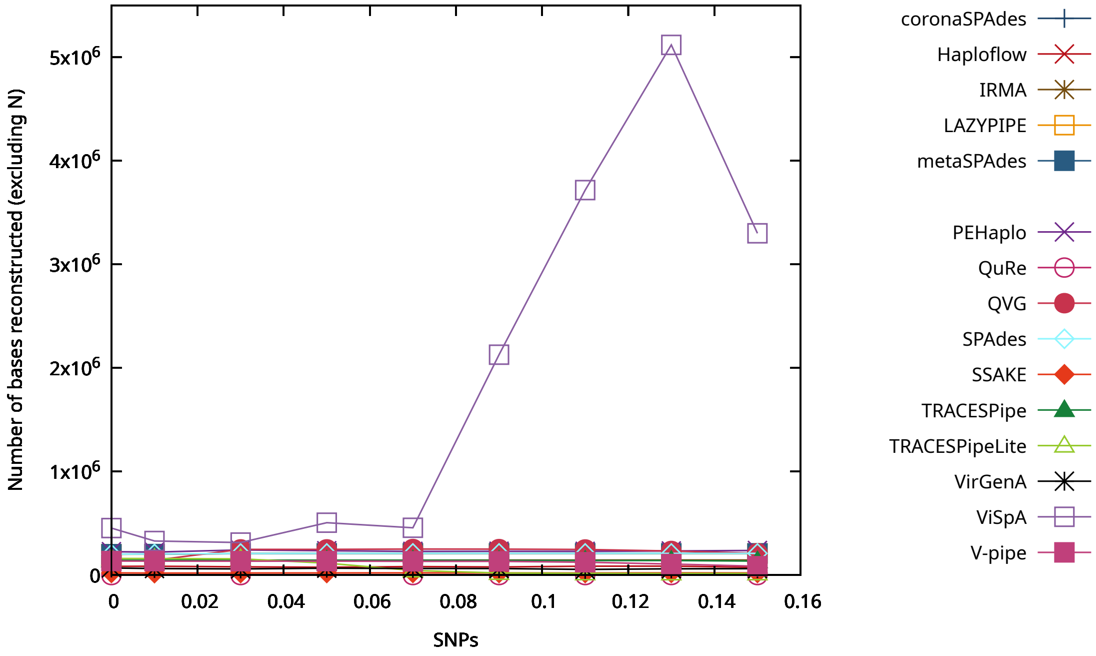

Figure S48: Figure comparing the performance of the reconstruction programs in terms of the number of bases reconstructed (excluding "N") for datasets with depth coverage equal to 10x (DS33 to DS40 plus DS11).

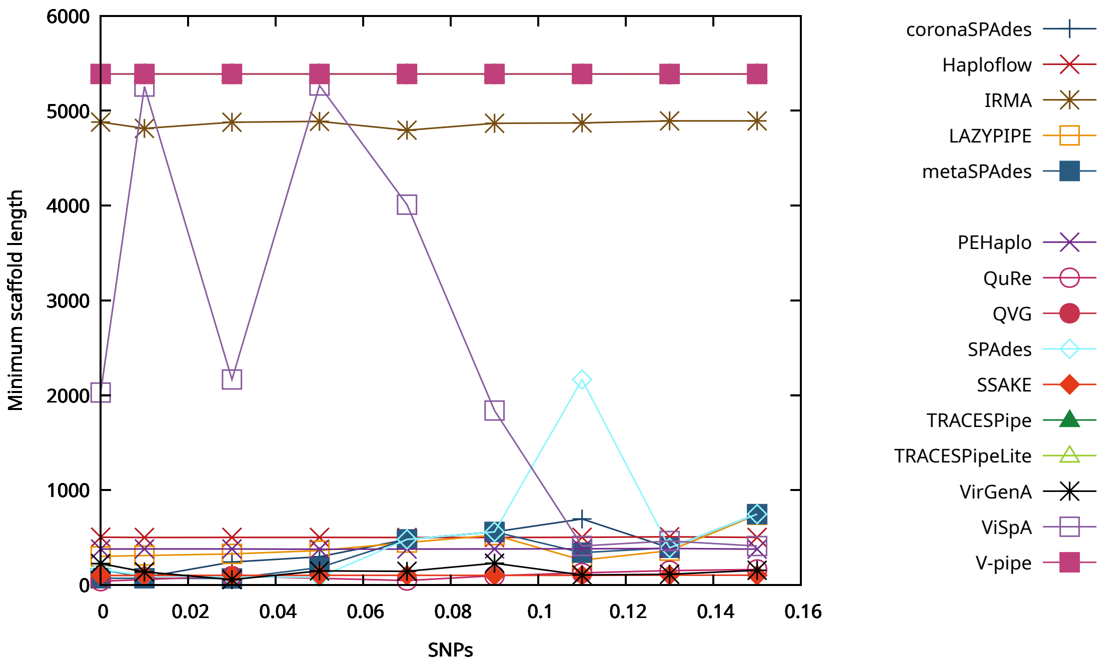

Figure S49: Figure comparing the performance of the reconstruction programs in terms of the minimum number of reconstructed bases per scaffold for datasets with depth coverage equal to 10x (DS33 to DS40 plus DS11).

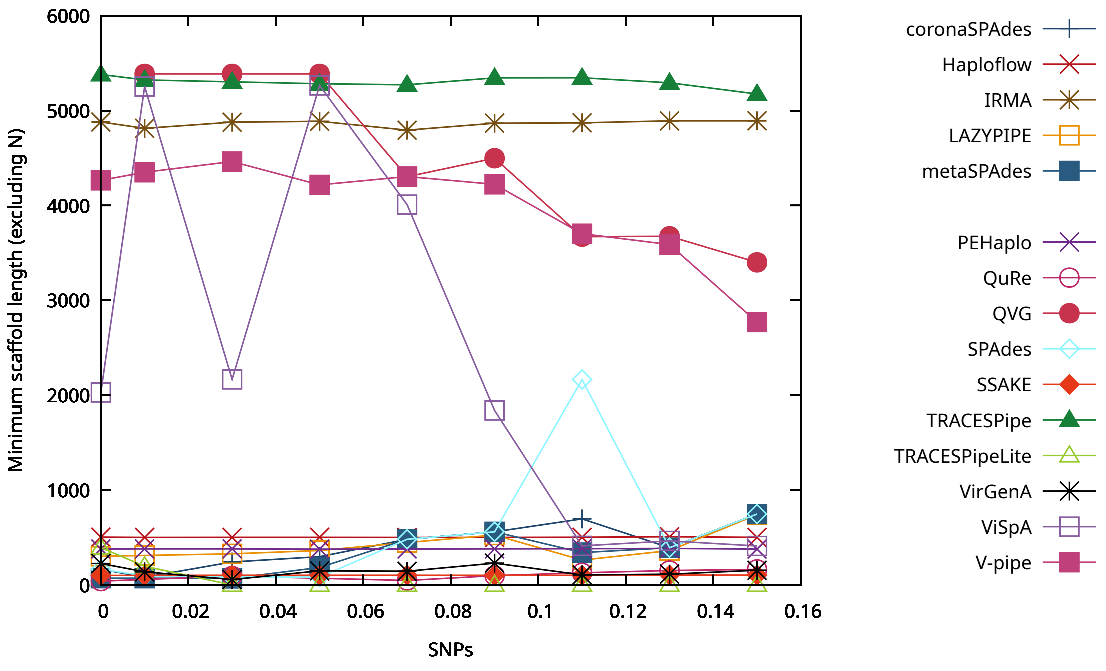

Figure S50: Figure comparing the performance of the reconstruction programs in terms of the minimum number of reconstructed bases per scaffold (excluding "N") for datasets with depth coverage equal to 10x (DS33 to DS40 plus DS11).

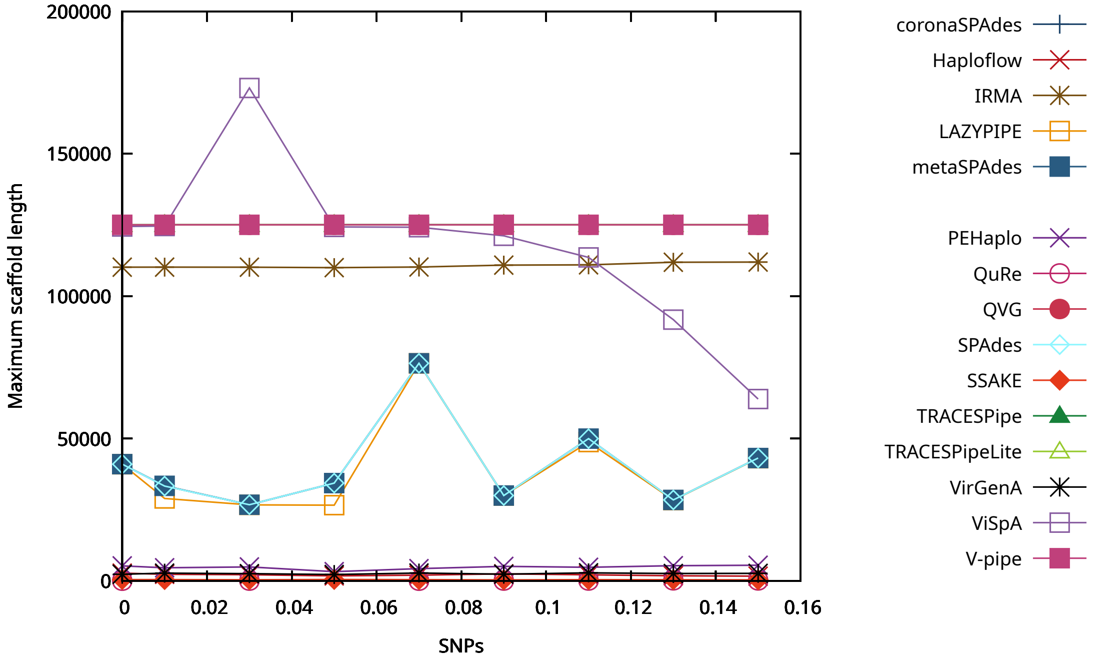

Figure S51: Figure comparing the performance of the reconstruction programs in terms of the maximum number of reconstructed bases per scaffold for datasets with depth coverage equal to 10x (DS33 to DS40 plus DS11).

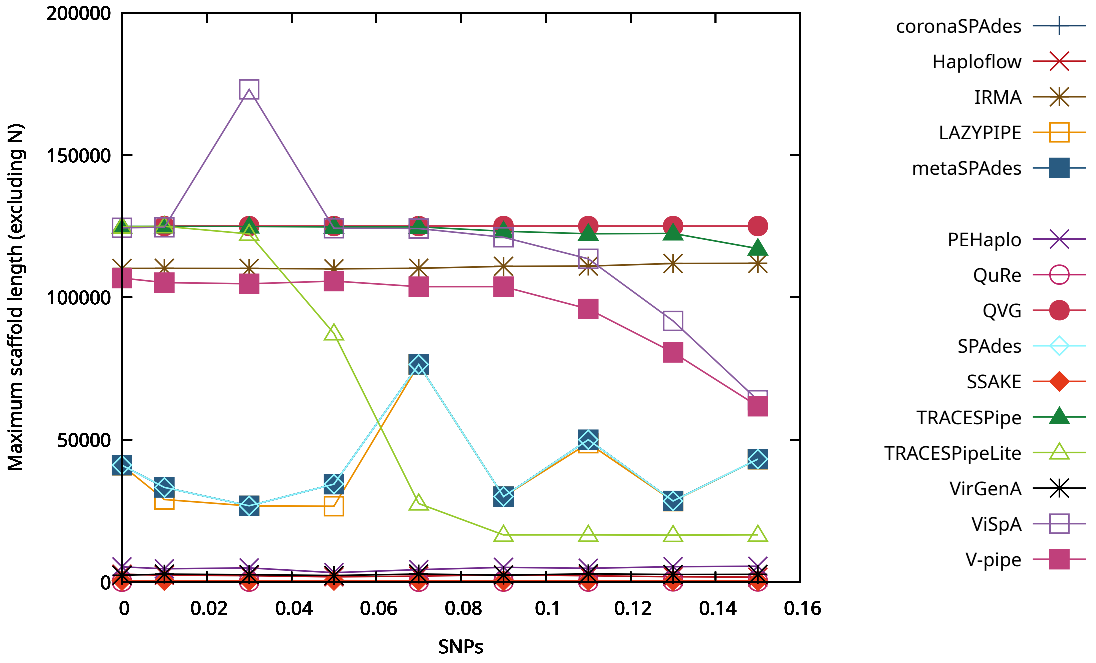

Figure S52: Figure comparing the performance of the reconstruction programs in terms of the maximum number of reconstructed bases per scaffold (excluding "N") for datasets with depth coverage equal to 10x (DS33 to DS40 plus DS11).

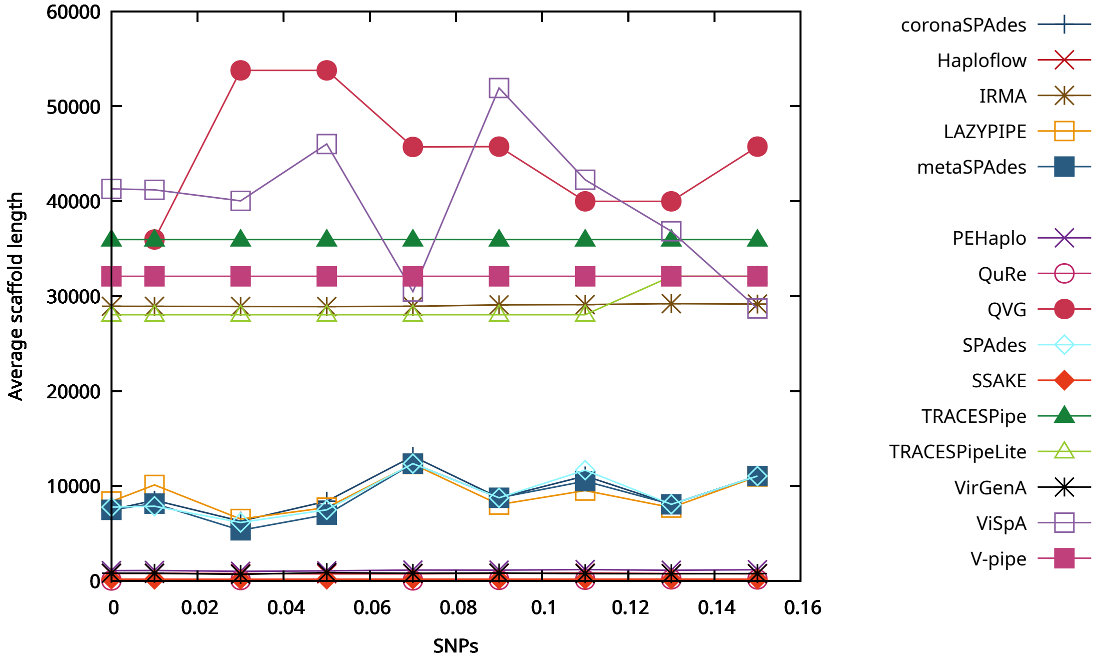

Figure S53: Figure comparing the performance of the reconstruction programs in terms of the average number of reconstructed bases per scaffold for datasets with depth coverage equal to 10x (DS33 to DS40 plus DS11).

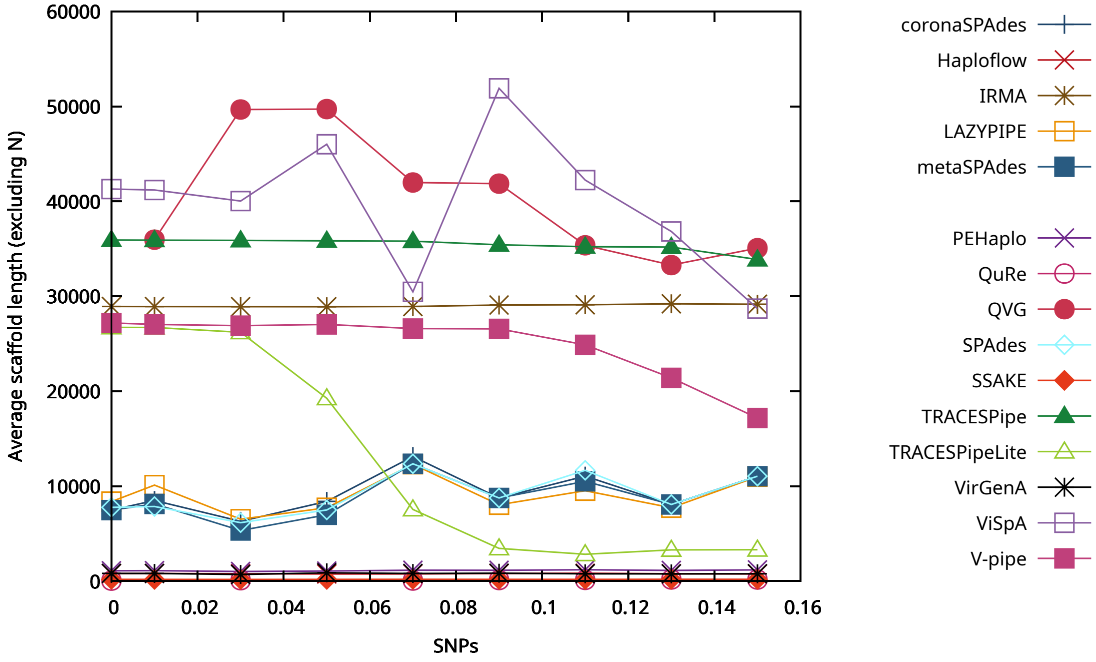

Figure S54: Figure comparing the performance of the reconstruction programs in terms of the average number of reconstructed bases per scaffold (excluding "N") for datasets with depth coverage equal to 10x (DS33 to DS40 plus DS11).

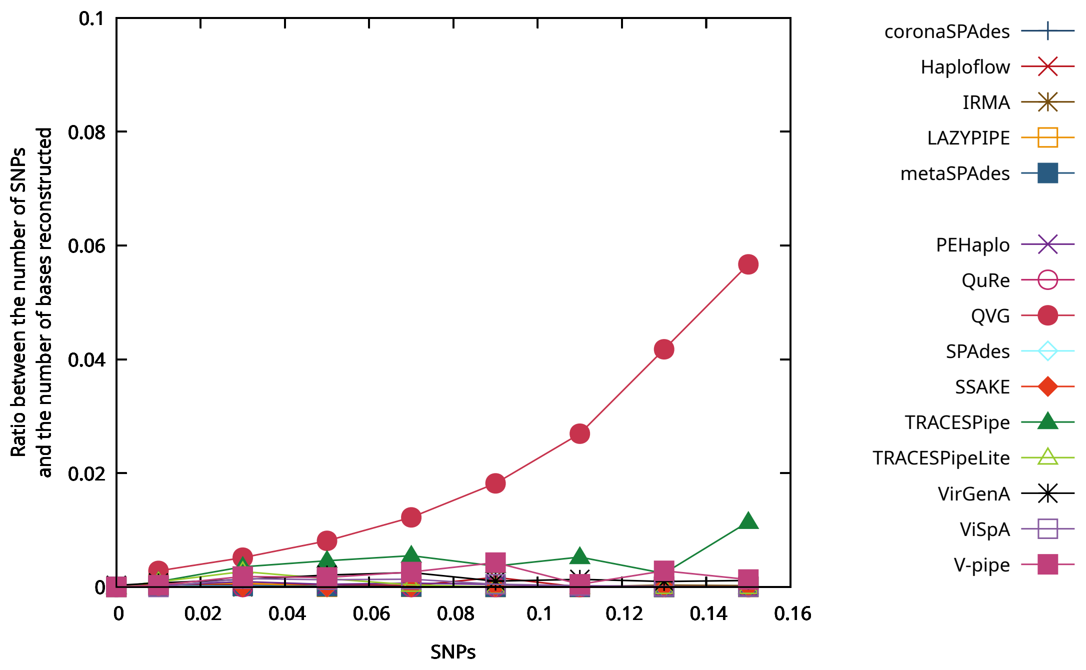

Figure S55: Figure comparing the performance of the reconstruction programs in terms of the ratio between the number of SNPs and the number of bases reconstructed for datasets with depth coverage equal to 10x (DS33 to DS40 plus DS11).

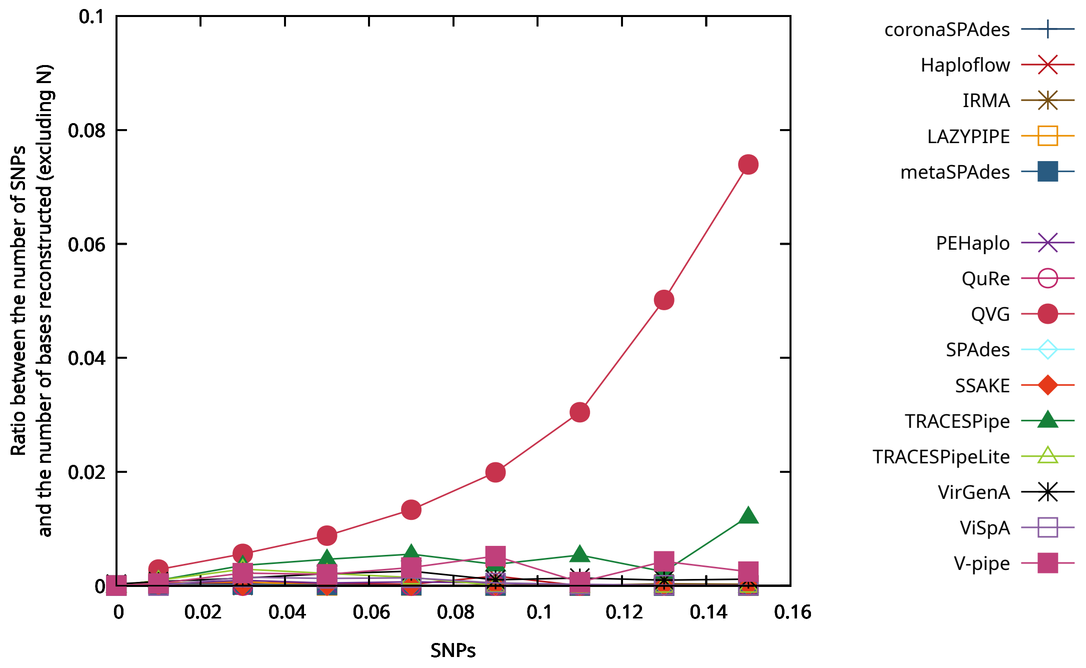

Figure S56: Figure comparing the performance of the reconstruction programs in terms of the ratio between the number of SNPs and the number of bases reconstructed (excluding "N") for datasets with depth coverage equal to 10x (DS33 to DS40 plus DS11).

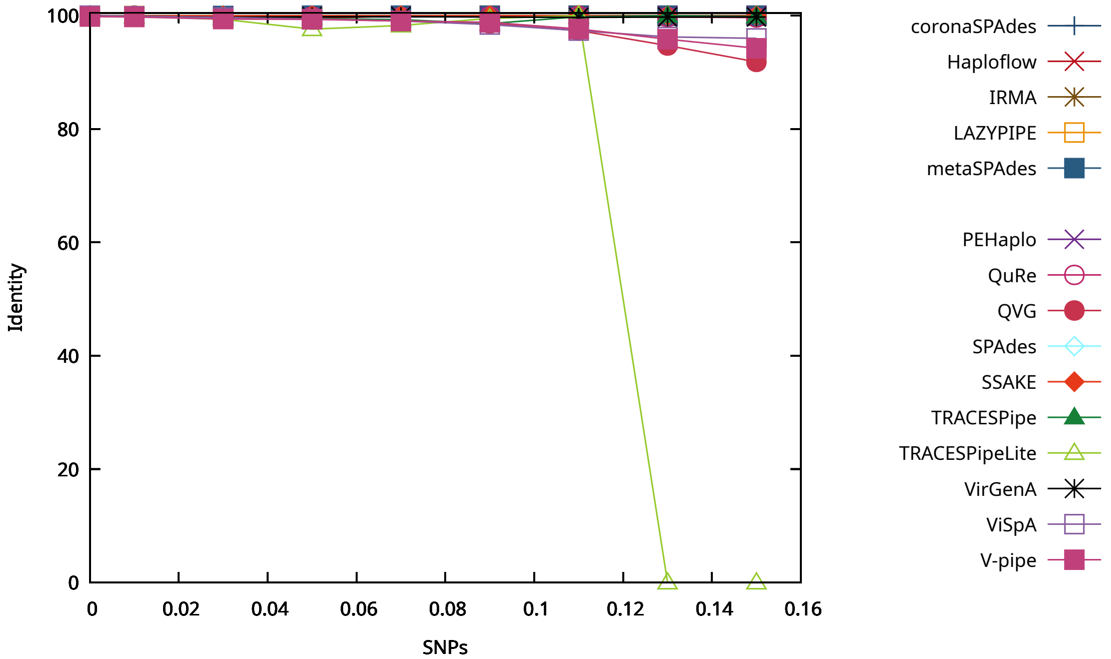

Figure S57: Figure comparing the performance of the reconstruction programs in terms of the identity for datasets with depth coverage equal to 20x (DS41 to DS48 plus DS13).

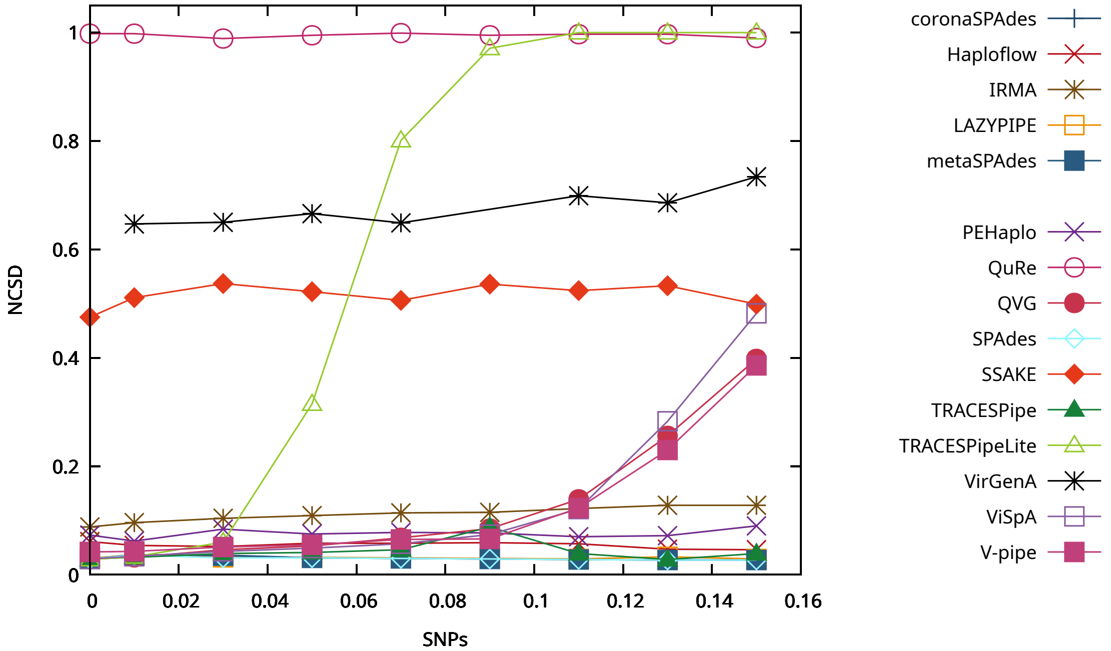

Figure S58: Figure comparing the performance of the reconstruction programs in terms of the NCSD for datasets with depth coverage equal to 20x (DS41 to DS48 plus DS13).

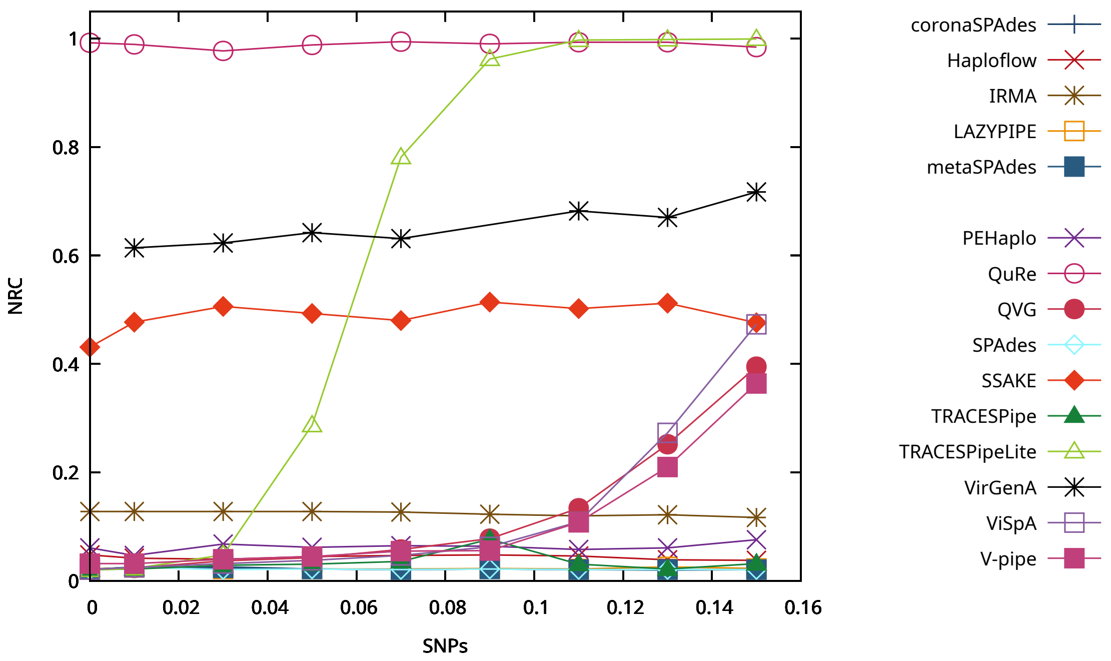

Figure S59: Figure comparing the performance of the reconstruction programs in terms of the NRC for datasets with depth coverage equal to 20x (DS41 to DS48 plus DS13).

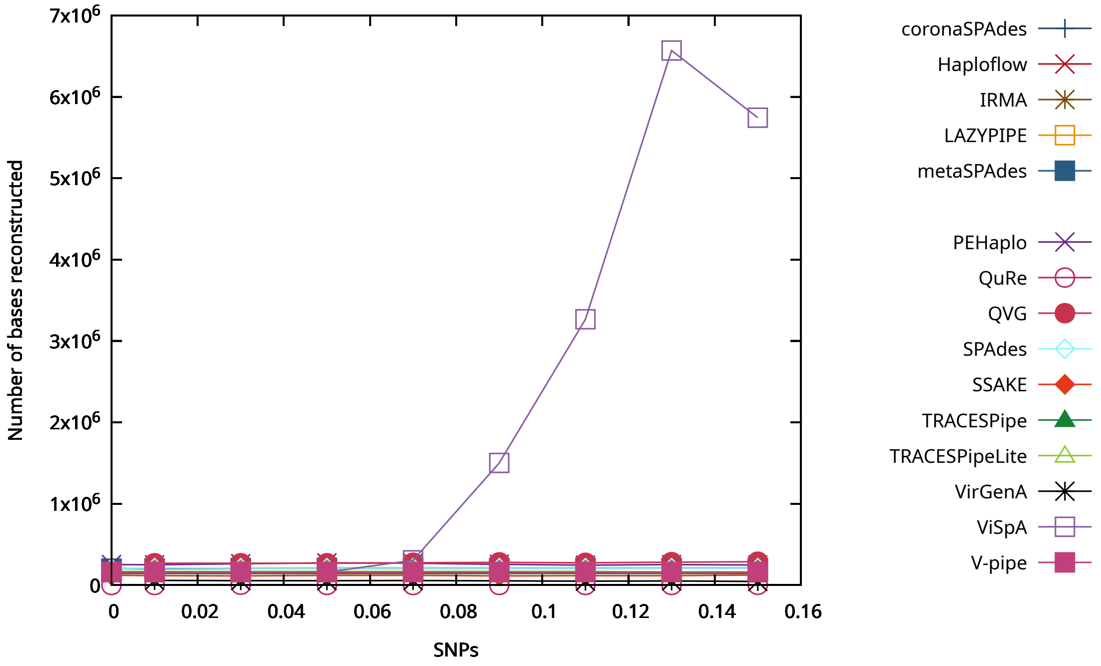

Figure S60: Figure comparing the performance of the reconstruction programs in terms of the number of bases reconstructed for datasets with depth coverage equal to 20x (DS41 to DS48 plus DS13).

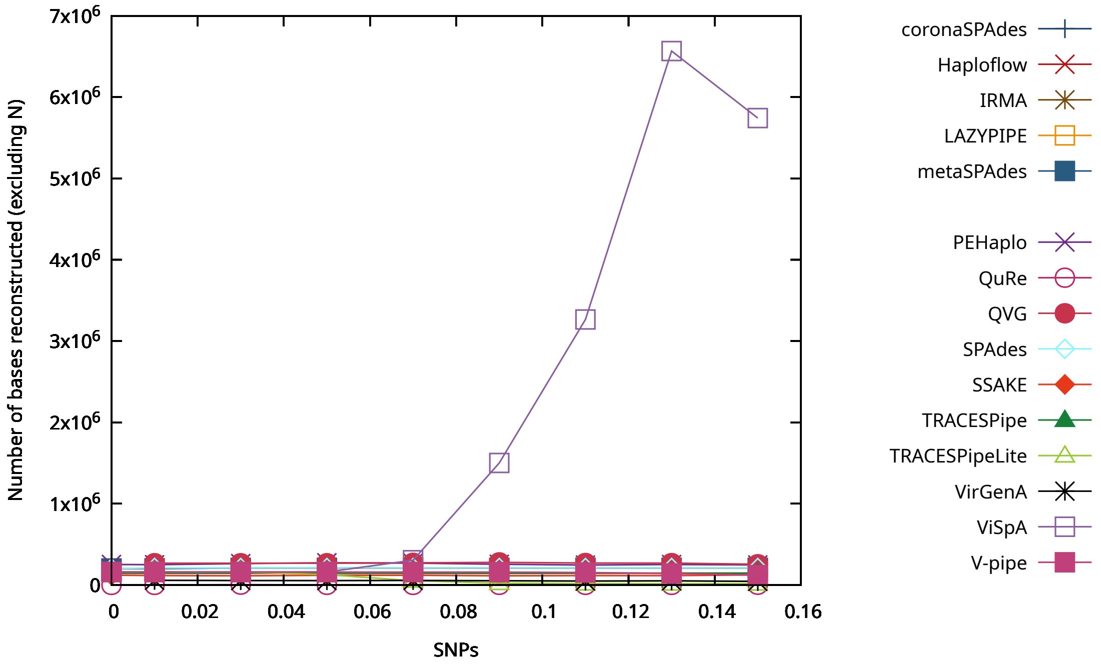

Figure S61: Figure comparing the performance of the reconstruction programs in terms of the number of bases reconstructed (excluding "N") for datasets with depth coverage equal to 20x (DS41 to DS48 plus DS13).

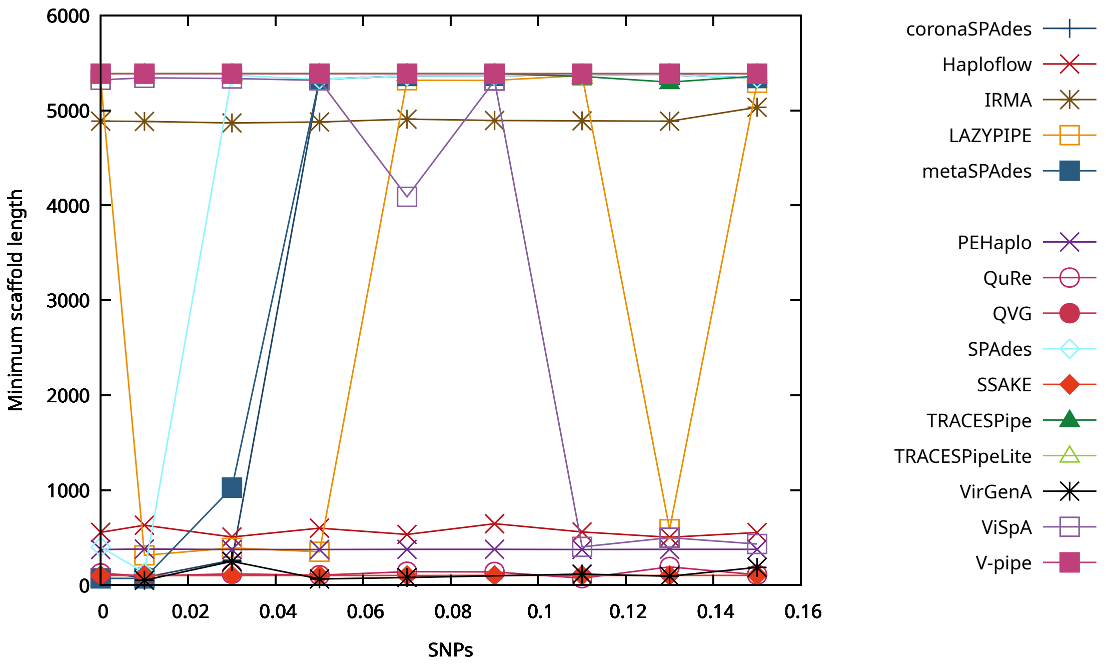

Figure S62: Figure comparing the performance of the reconstruction programs in terms of the minimum number of reconstructed bases per scaffold for datasets with depth coverage equal to 20x (DS41 to DS48 plus DS13).

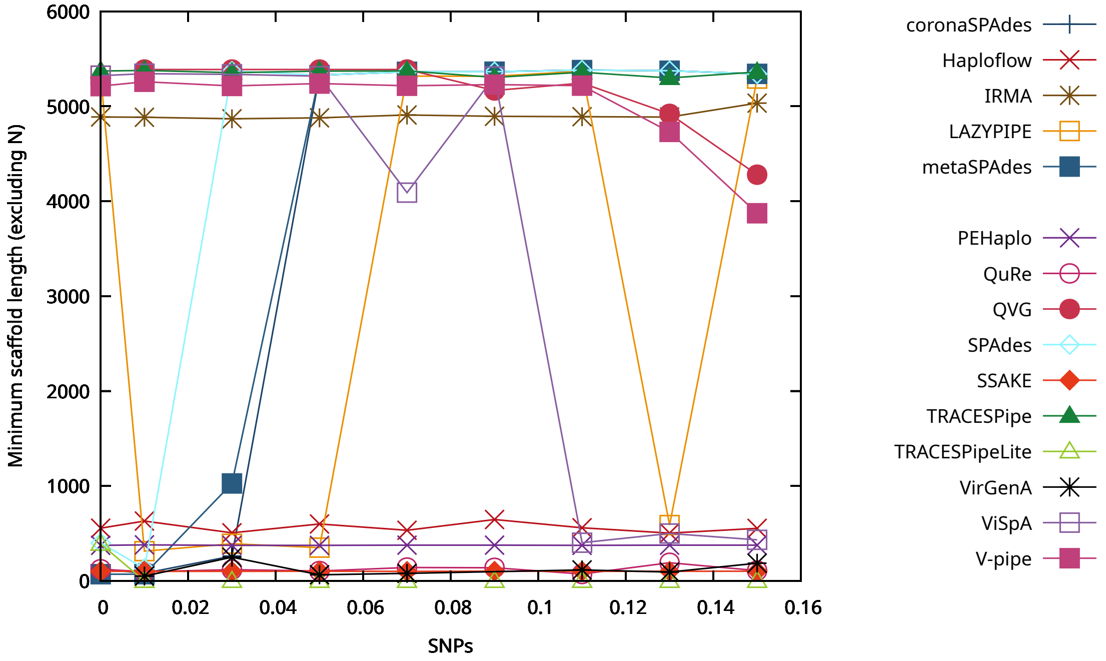

Figure S63: Figure comparing the performance of the reconstruction programs in terms of the minimum number of reconstructed bases per scaffold (excluding "N") for datasets with depth coverage equal to 20x (DS41 to DS48 plus DS13).

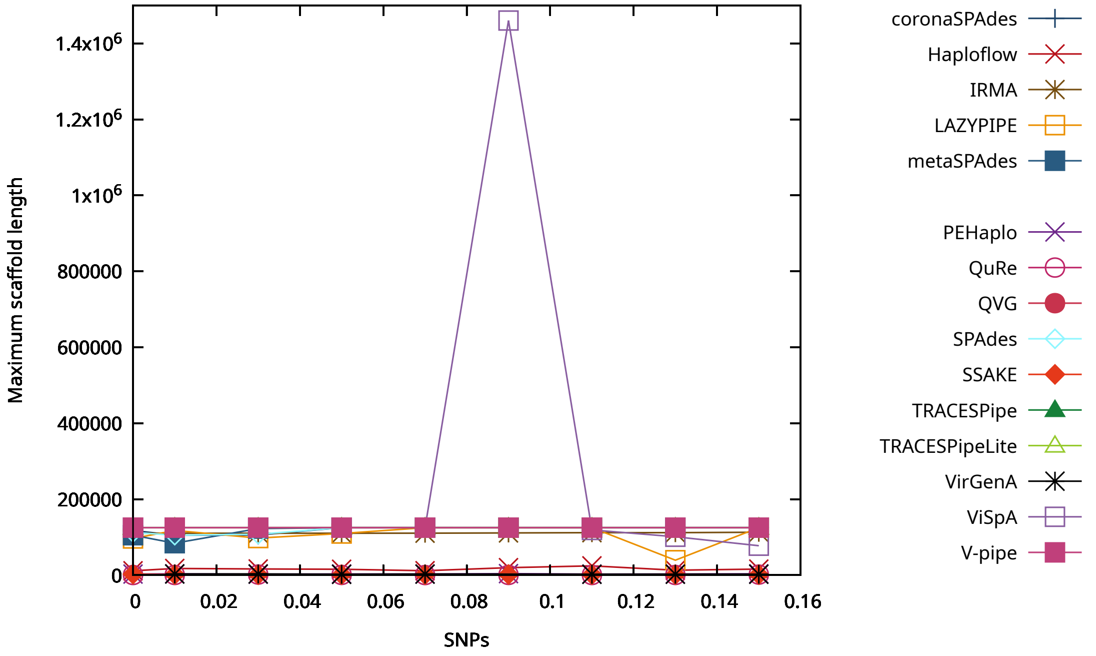

Figure S64: Figure comparing the performance of the reconstruction programs in terms of the maximum number of reconstructed bases per scaffold for datasets with depth coverage equal to 20x (DS41 to DS48 plus DS13).

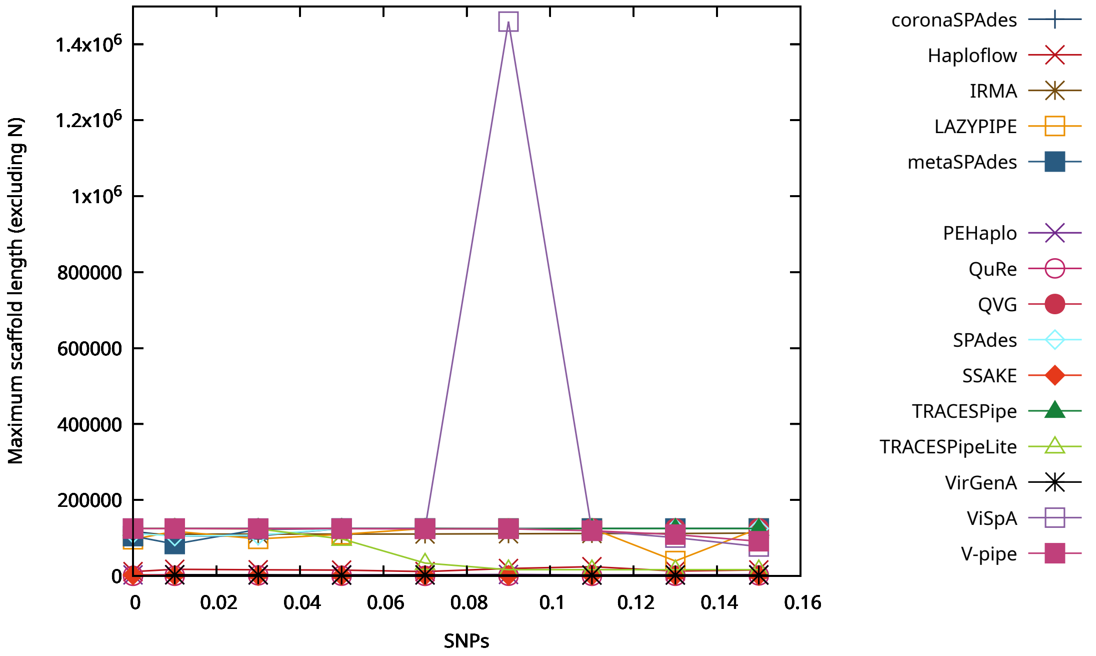

Figure S65: Figure comparing the performance of the reconstruction programs in terms of the maximum number of reconstructed bases per scaffold (excluding "N") for datasets with depth coverage equal to 20x (DS41 to DS48 plus DS13).

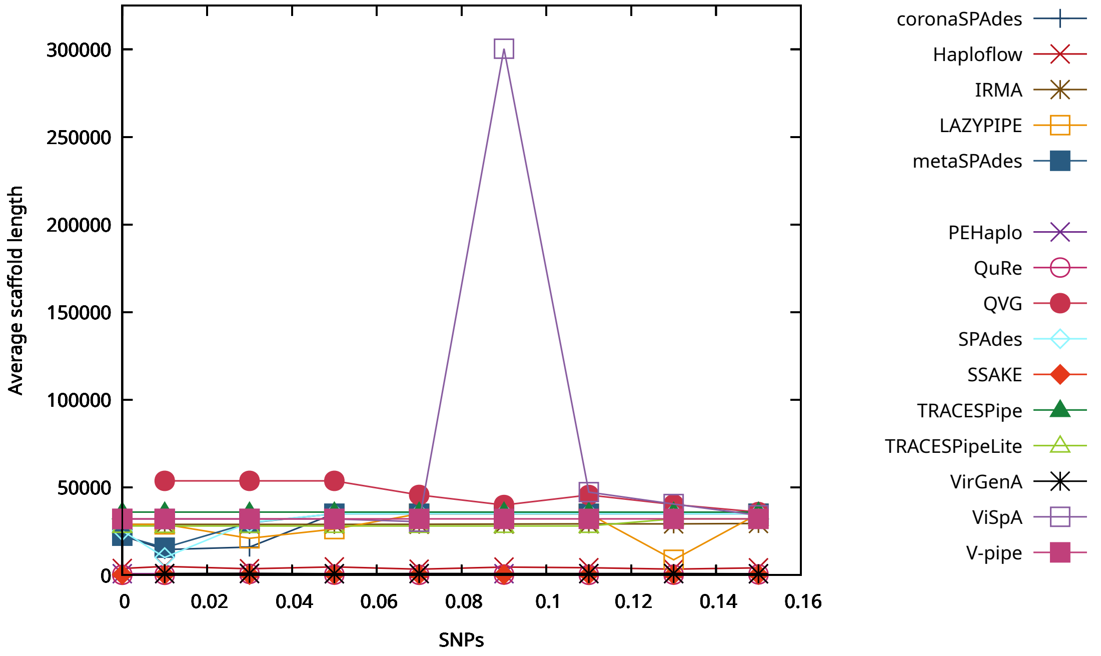

Figure S66: Figure comparing the performance of the reconstruction programs in terms of the average number of reconstructed bases per scaffold for datasets with depth coverage equal to 20x (DS41 to DS48 plus DS13).

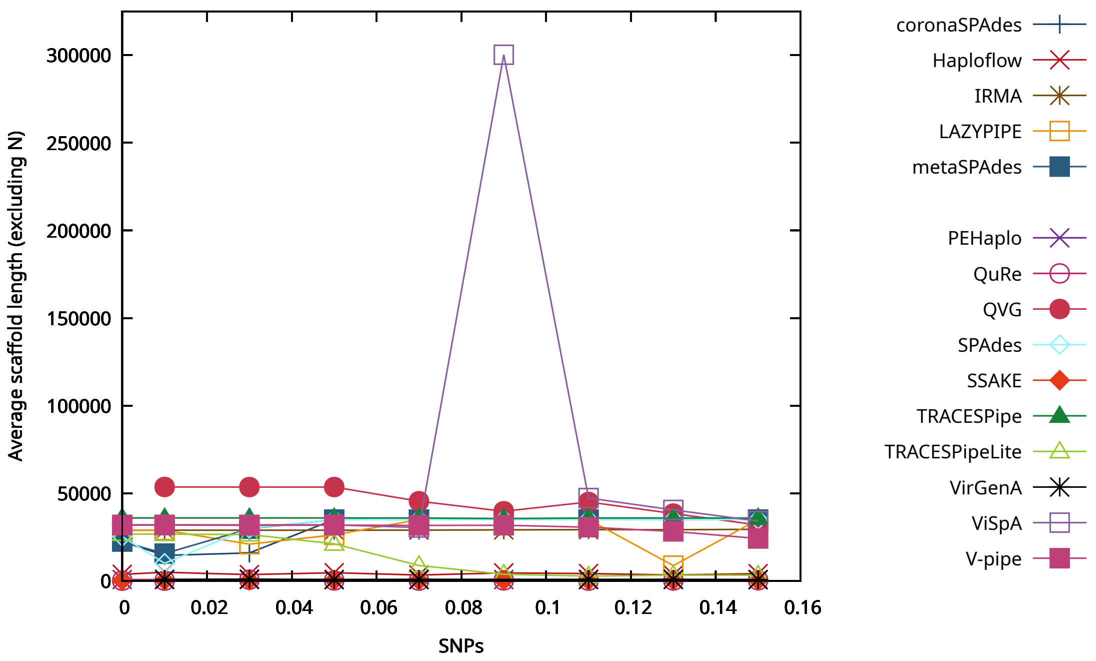

Figure S67: Figure comparing the performance of the reconstruction programs in terms of the average number of reconstructed bases per scaffold (excluding "N") for datasets with depth coverage equal to 20x (DS41 to DS48 plus DS13).

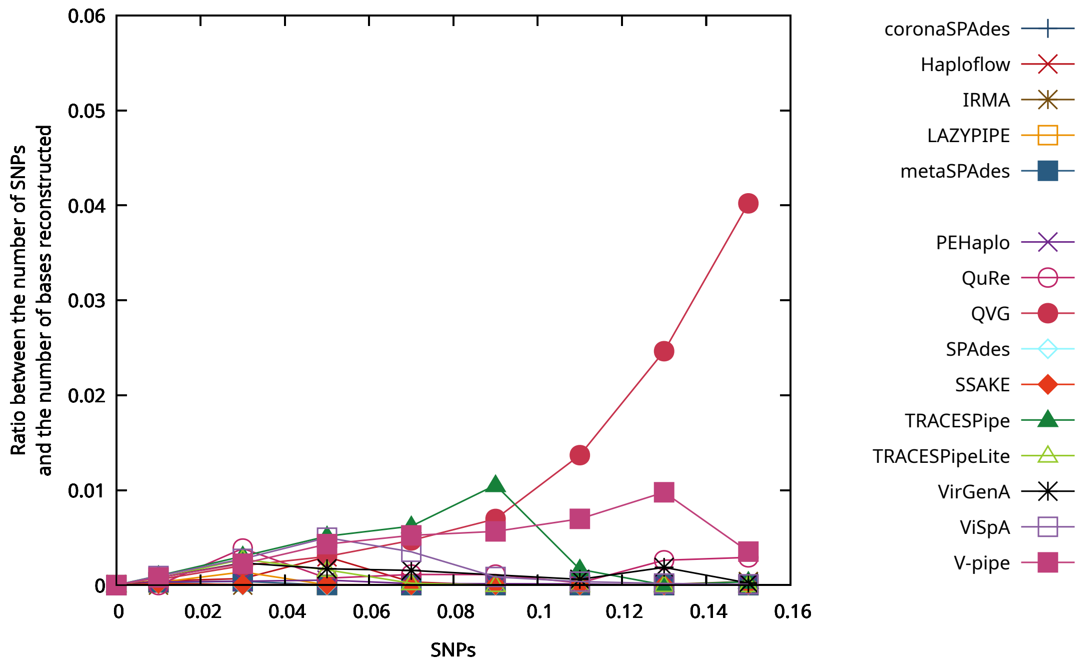

Figure S68: Figure comparing the performance of the reconstruction programs in terms of the ratio between the number of SNPs and the number of bases reconstructed for datasets with depth coverage equal to 20x (DS41 to DS48 plus DS13).

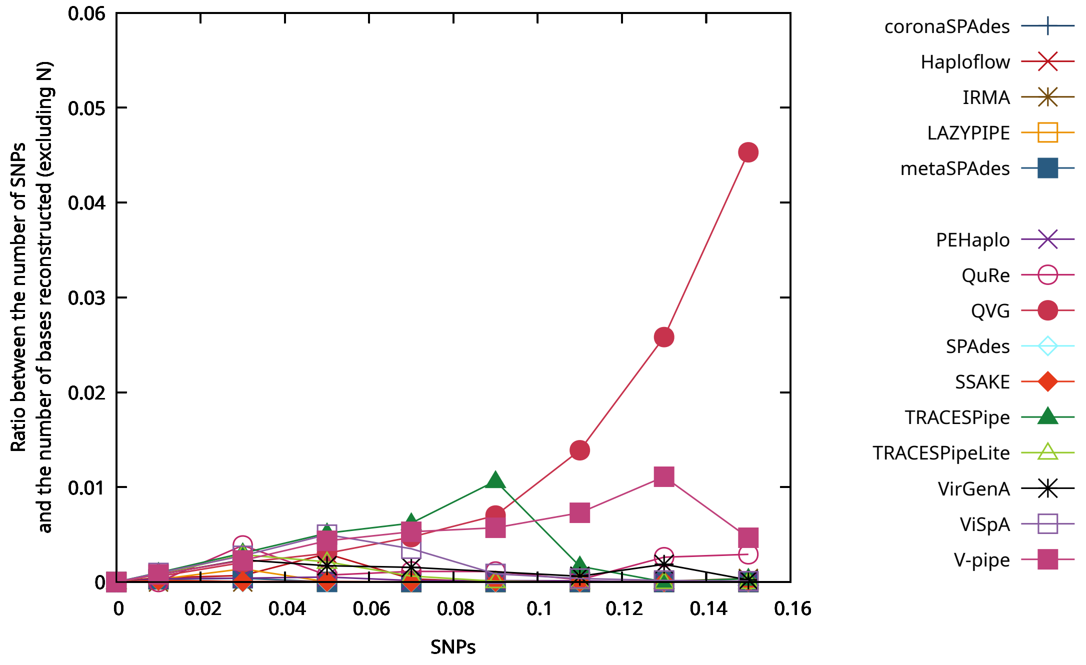

Figure S69: Figure comparing the performance of the reconstruction programs in terms of the ratio between the number of SNPs and the number of bases reconstructed (excluding "N") for datasets with depth coverage equal to 20x (DS41 to DS48 plus DS13).

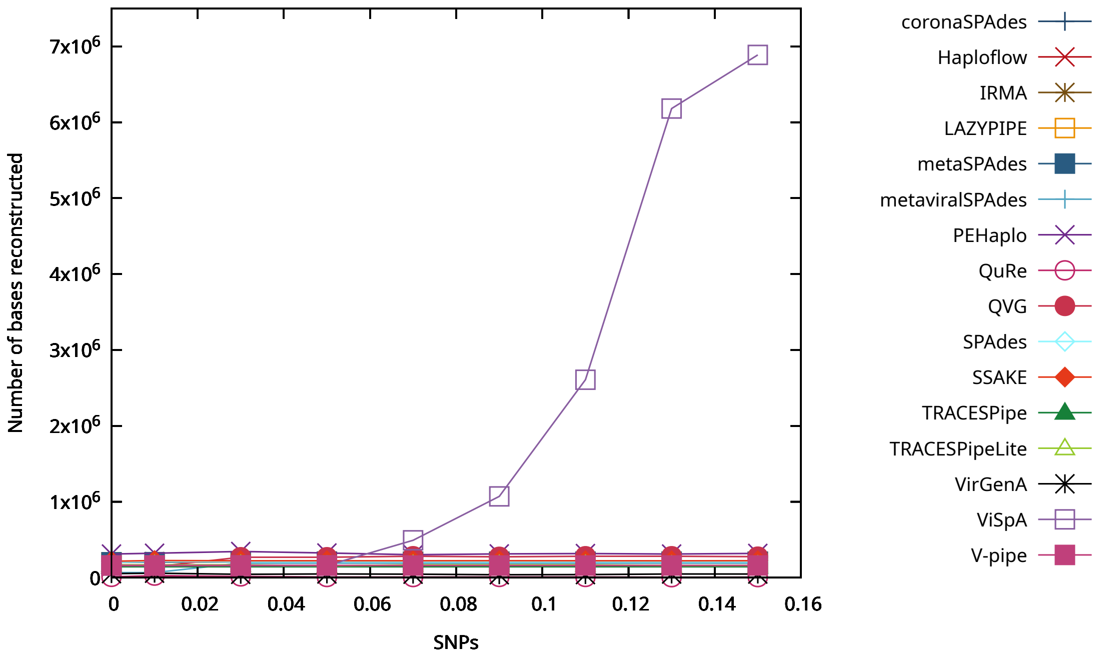

Figure S70: Figure comparing the performance of the reconstruction programs in terms of the number of bases reconstructed for datasets with depth coverage equal to 40x (DS49 to DS56 plus DS16).

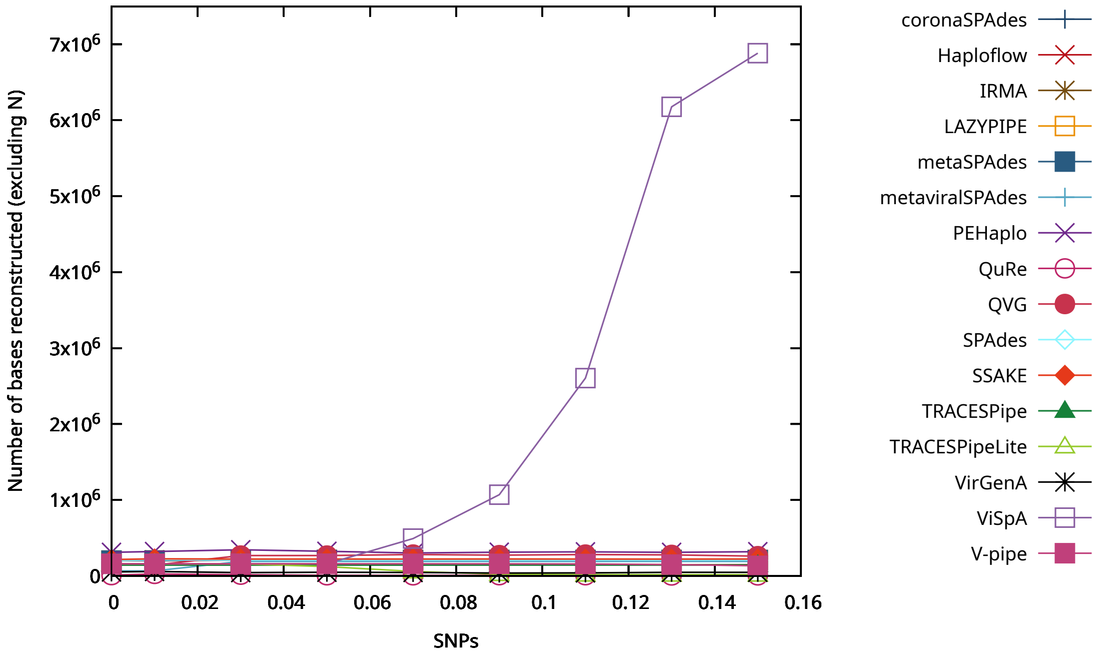

Figure S71: Figure comparing the performance of the reconstruction programs in terms of the number of bases reconstructed (excluding "N") for datasets with depth coverage equal to 40x (DS49 to DS56 plus DS16).

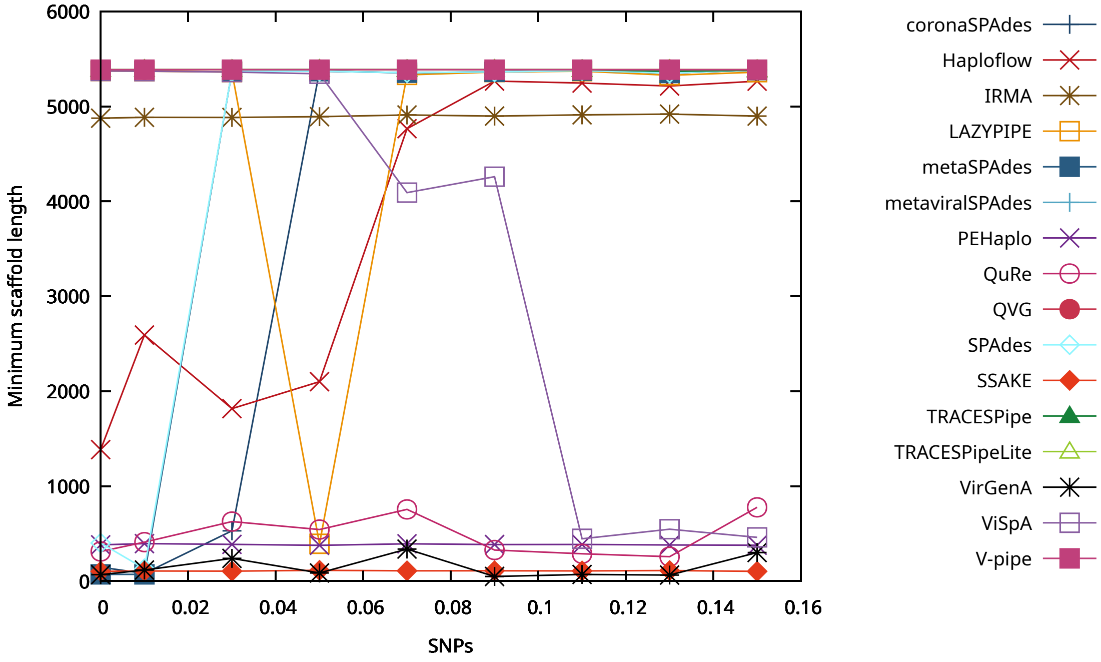

Figure S72: Figure comparing the performance of the reconstruction programs in terms of the minimum number of reconstructed bases per scaffold for datasets with depth coverage equal to 40x (DS49 to DS56 plus DS16).

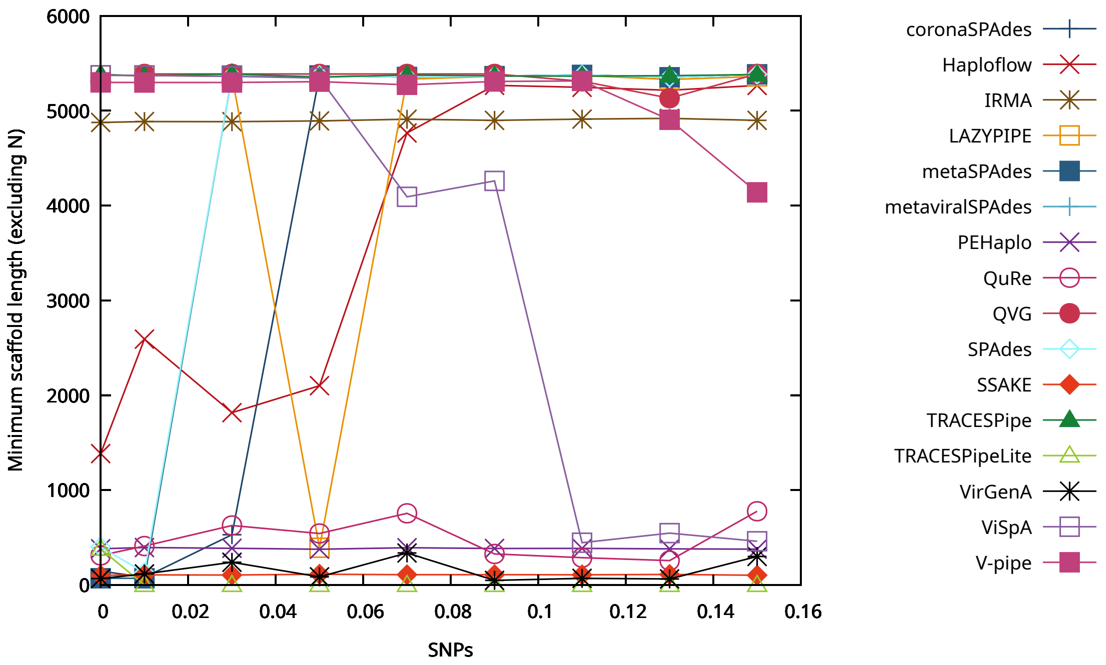

Figure S73: Figure comparing the performance of the reconstruction programs in terms of the minimum number of reconstructed bases per scaffold (excluding "N") for datasets with depth coverage equal to 40x (DS49 to DS56 plus DS16).

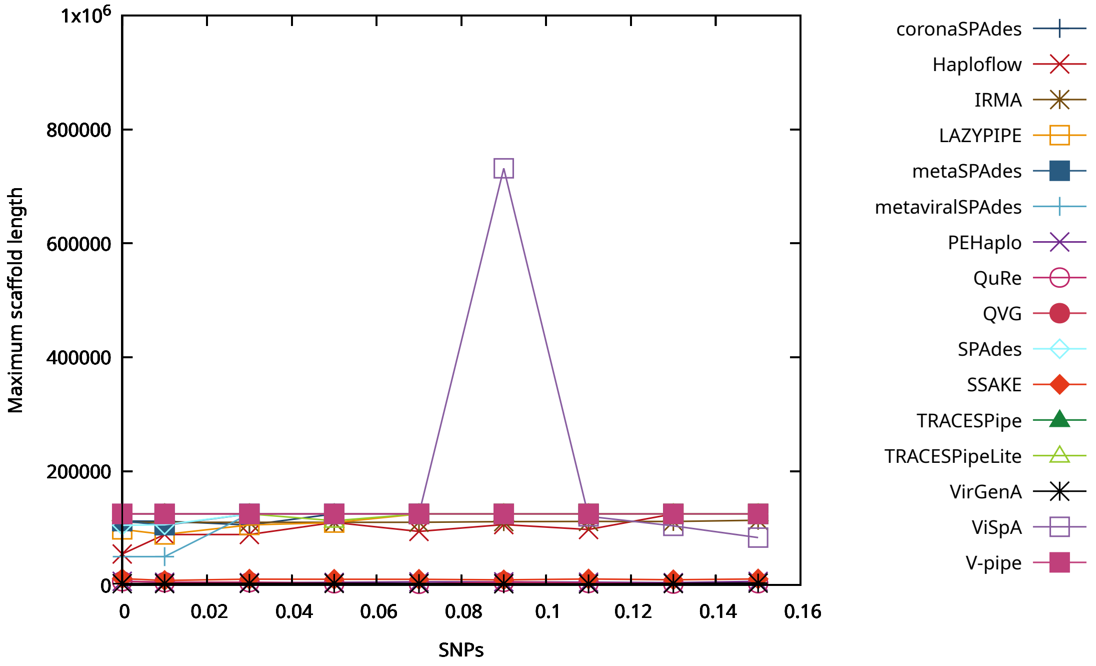

Figure S74: Figure comparing the performance of the reconstruction programs in terms of the maximum number of reconstructed bases per scaffold for datasets with depth coverage equal to 40x (DS49 to DS56 plus DS16).

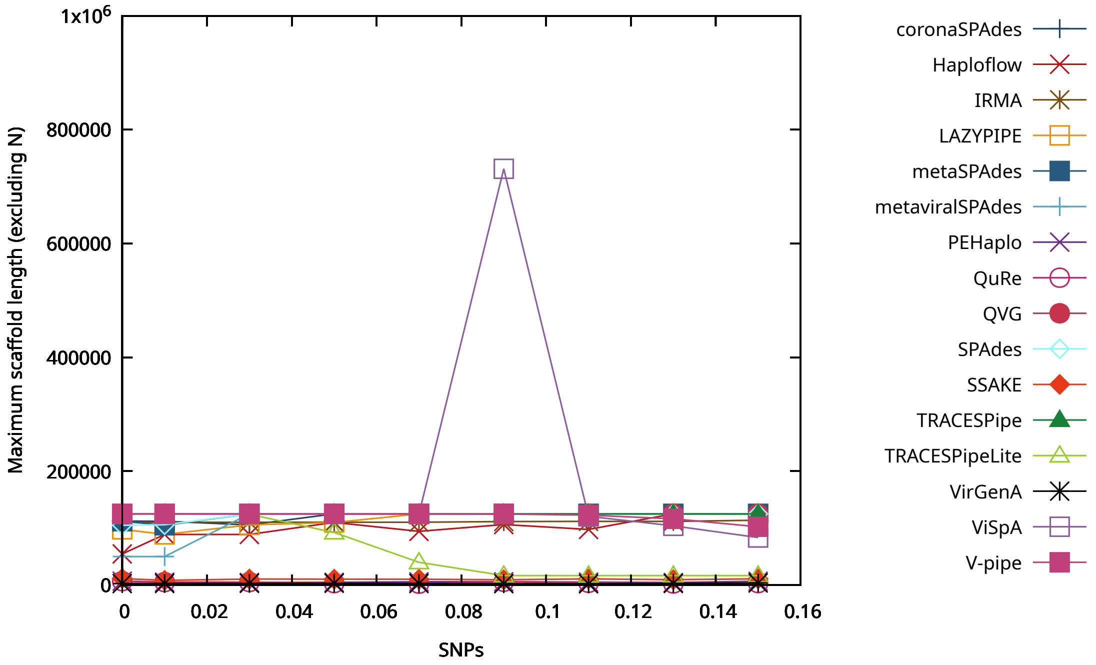

Figure S75: Figure comparing the performance of the reconstruction programs in terms of the maximum number of reconstructed bases per scaffold (excluding "N") for datasets with depth coverage equal to 40x (DS49 to DS56 plus DS16).

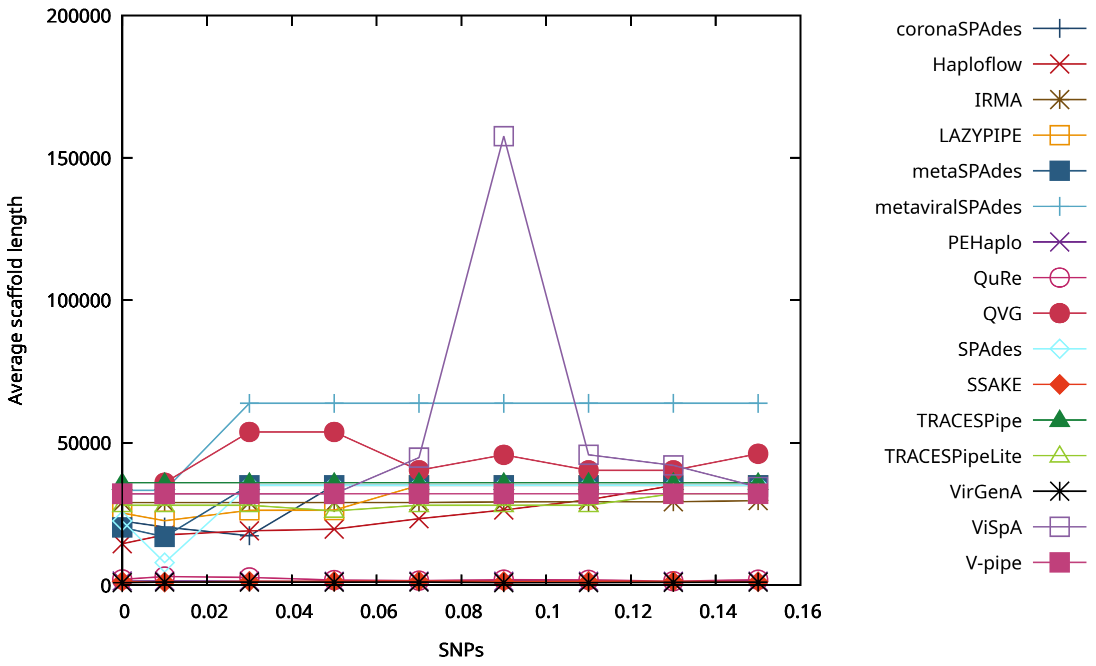

Figure S76: Figure comparing the performance of the reconstruction programs in terms of the average number of reconstructed bases per scaffold for datasets with depth coverage equal to 40x (DS49 to DS56 plus DS16).

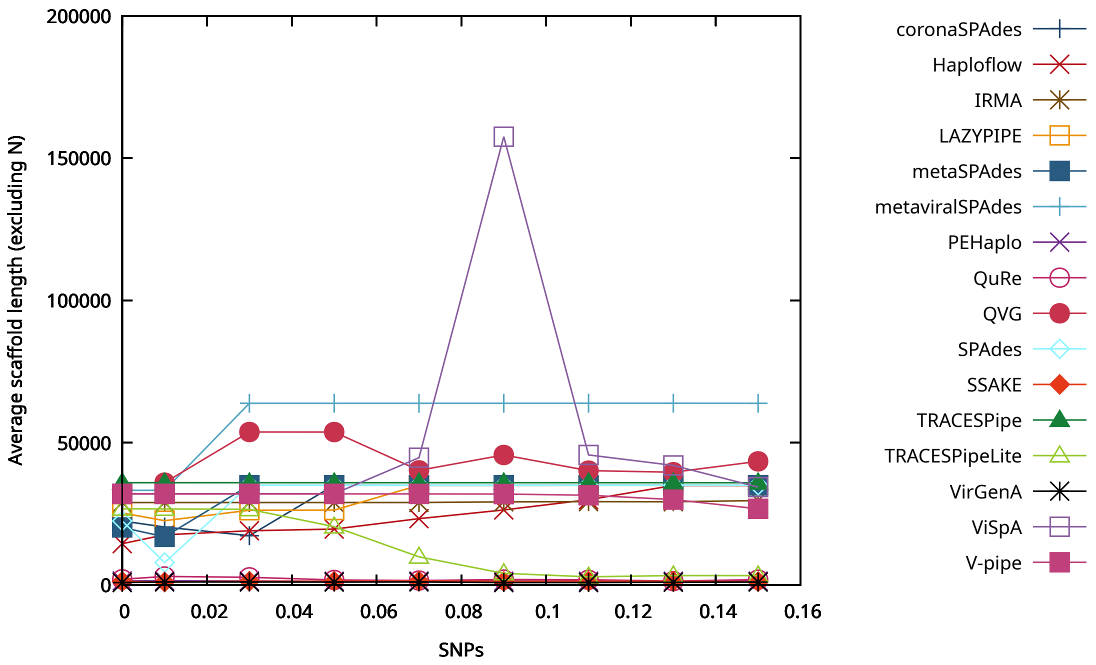

Figure S77: Figure comparing the performance of the reconstruction programs in terms of the average number of reconstructed bases per scaffold (excluding "N") for datasets with depth coverage equal to 40x (DS49 to DS56 plus DS16).

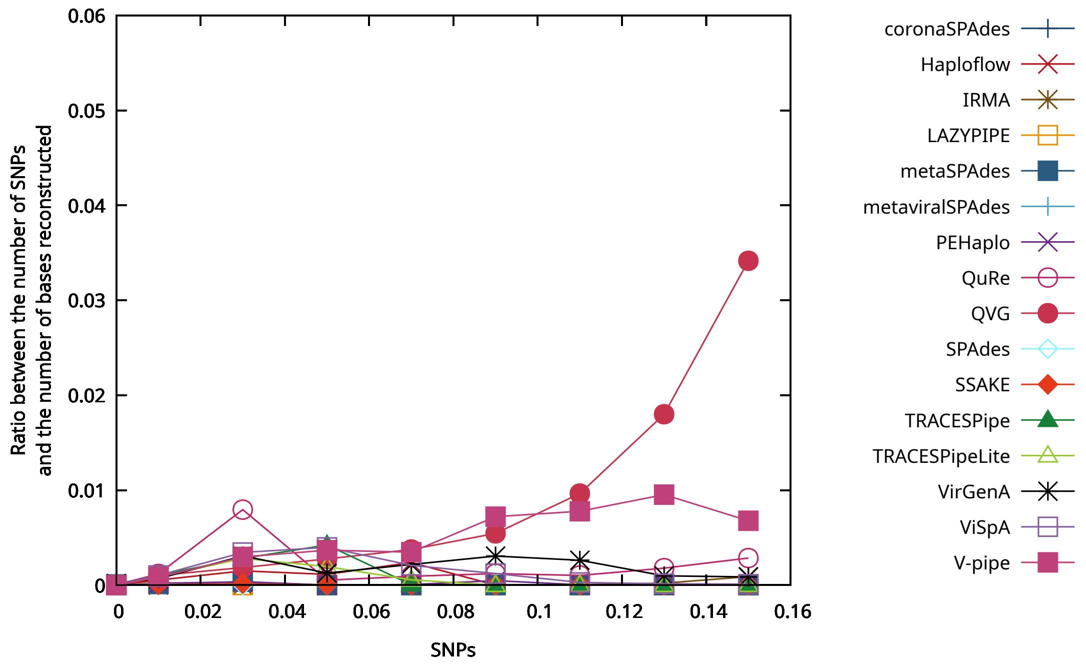

Figure S78: Figure comparing the performance of the reconstruction programs in terms of the ratio between the number of SNPs and the number of bases reconstructed for datasets with depth coverage equal to 40x (DS49 to DS56 plus DS16).

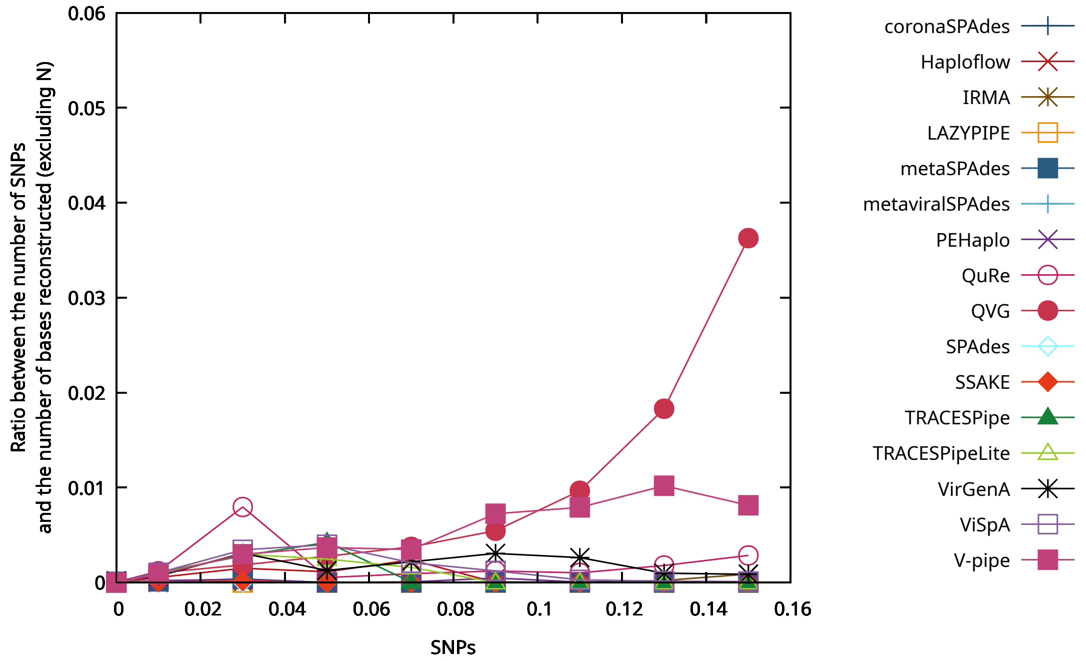

Figure S79: Figure comparing the performance of the reconstruction programs in terms of the ratio between the number of SNPs and the number of bases reconstructed (excluding "N") for datasets with depth coverage equal to 40x (DS49 to DS56 plus DS16).

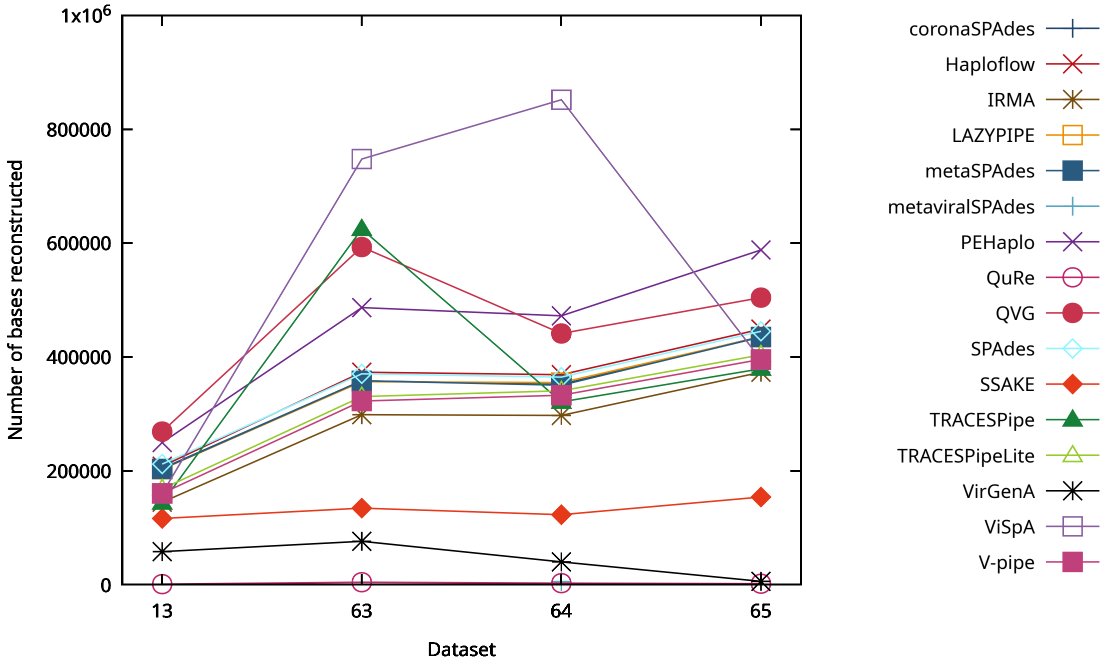

Figure S80: Figure comparing the performance of the reconstruction programs in terms of the number of bases reconstructed for datasets with different viral compositions (datasets 13, 63, 64 and 65).

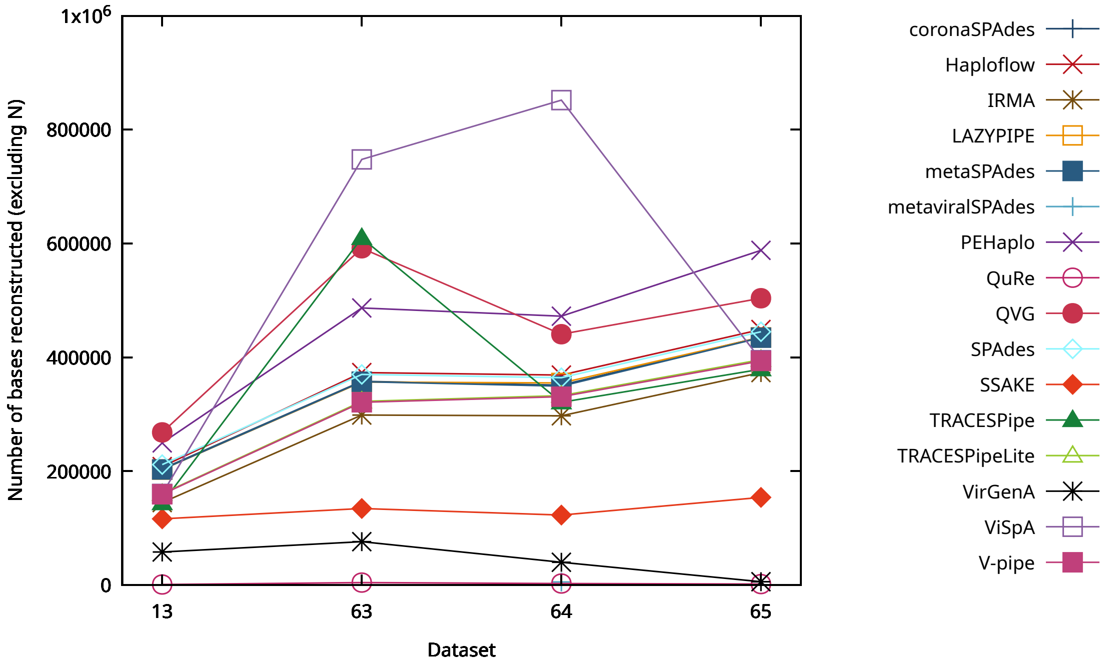

Figure S81: Figure comparing the performance of the reconstruction programs in terms of the number of bases reconstructed (excluding "N") for datasets with different viral compositions (datasets 13, 63, 64 and 65).

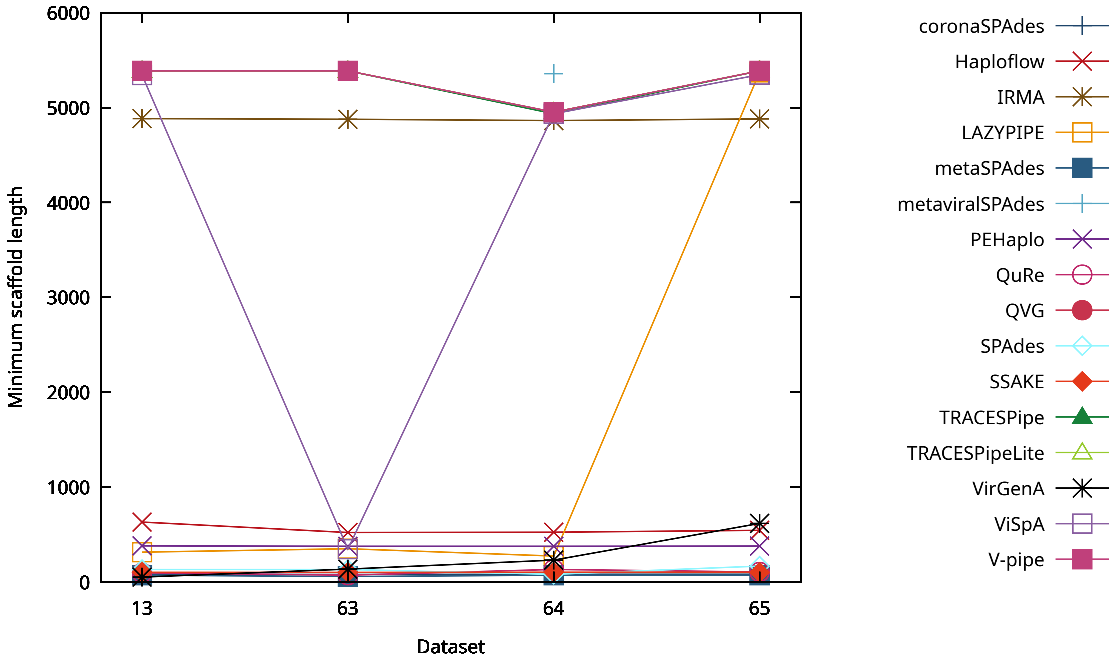

Figure S82: Figure comparing the performance of the reconstruction programs in terms of the minimum number of reconstructed bases per scaffold for datasets with different viral compositions (datasets 13, 63, 64 and 65).

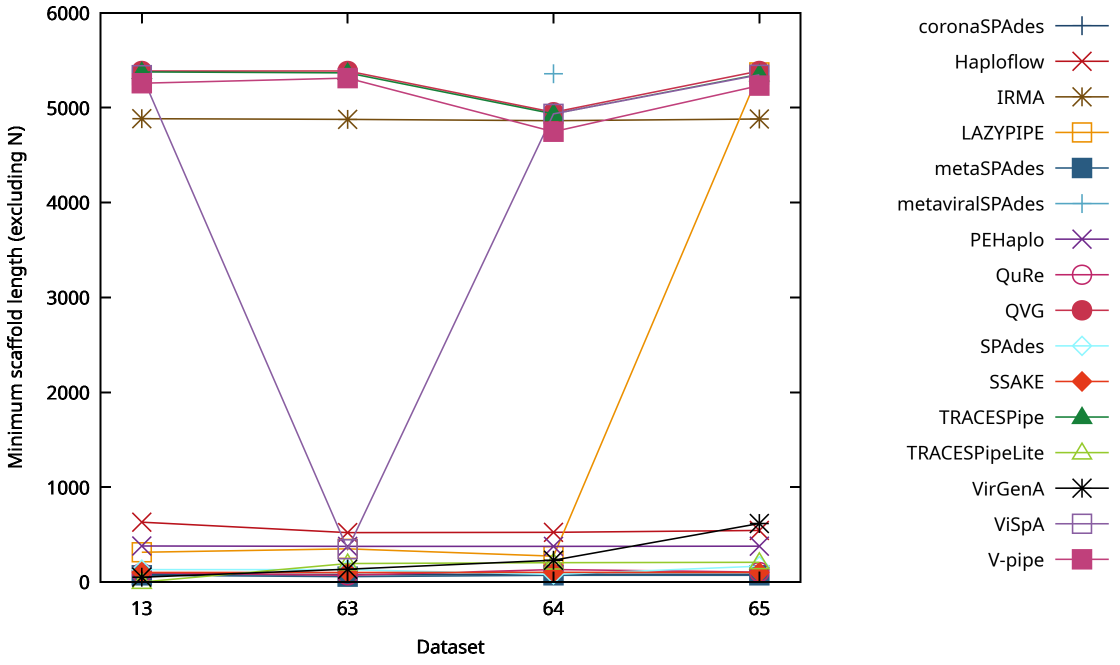

Figure S83: Figure comparing the performance of the reconstruction programs in terms of the minimum number of reconstructed bases per scaffold (excluding "N") for datasets with different viral compositions (datasets 13, 63, 64 and 65).

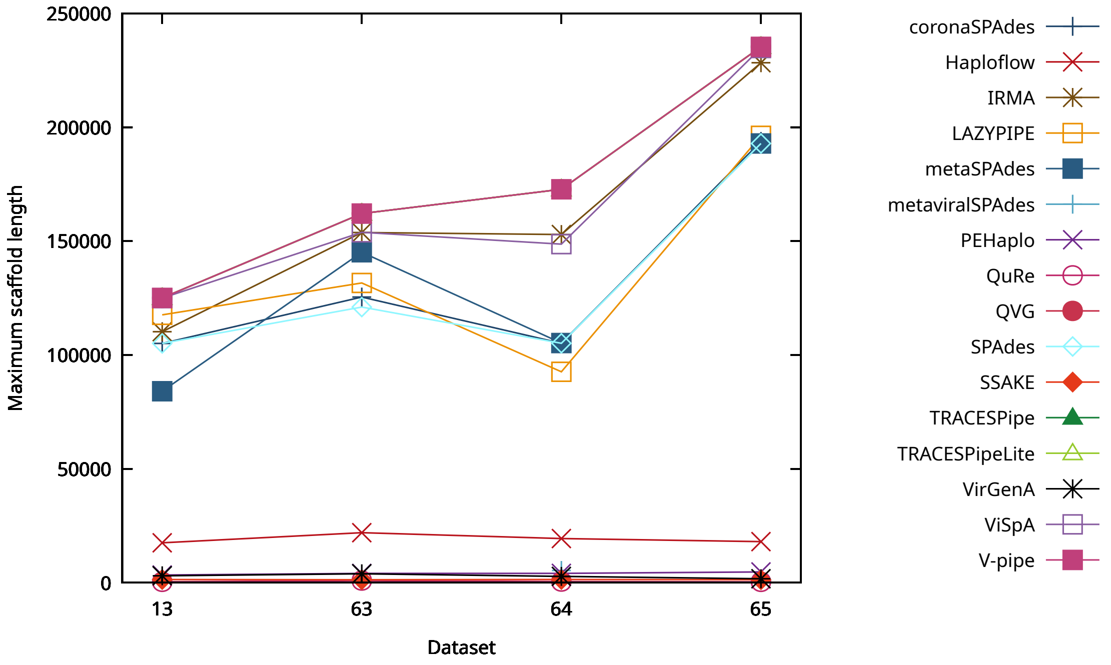

Figure S84: Figure comparing the performance of the reconstruction programs in terms of the maximum number of reconstructed bases per scaffold for datasets with different viral compositions (datasets 13, 63, 64 and 65).

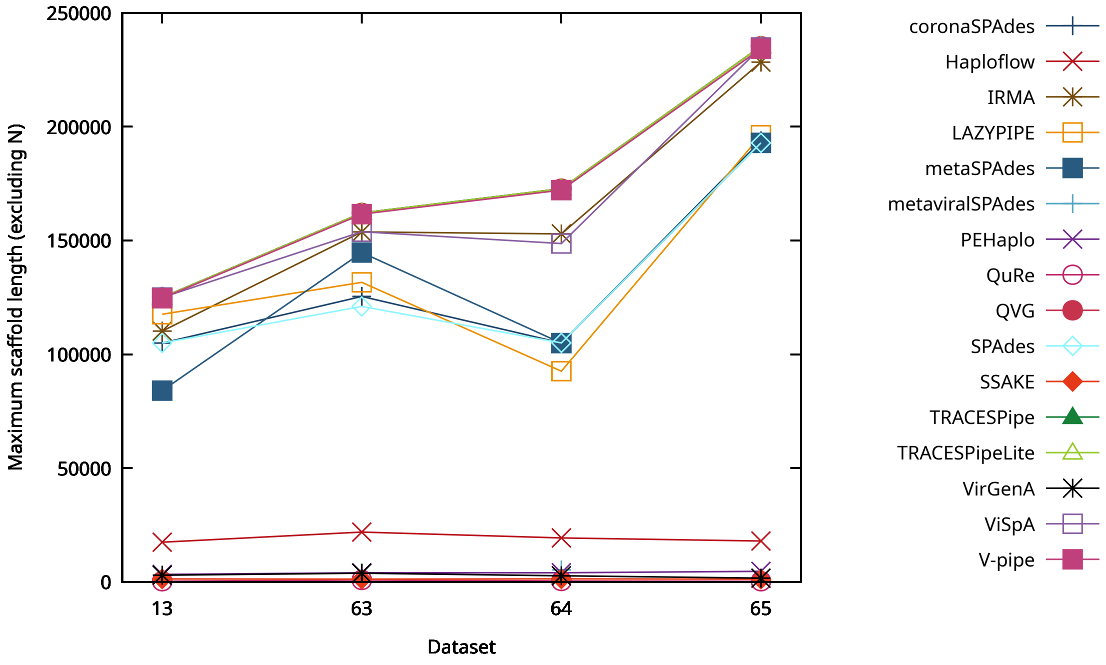

Figure S85: Figure comparing the performance of the reconstruction programs in terms of the maximum number of reconstructed bases per scaffold (excluding "N") for datasets with different viral compositions (datasets 13, 63, 64 and 65).

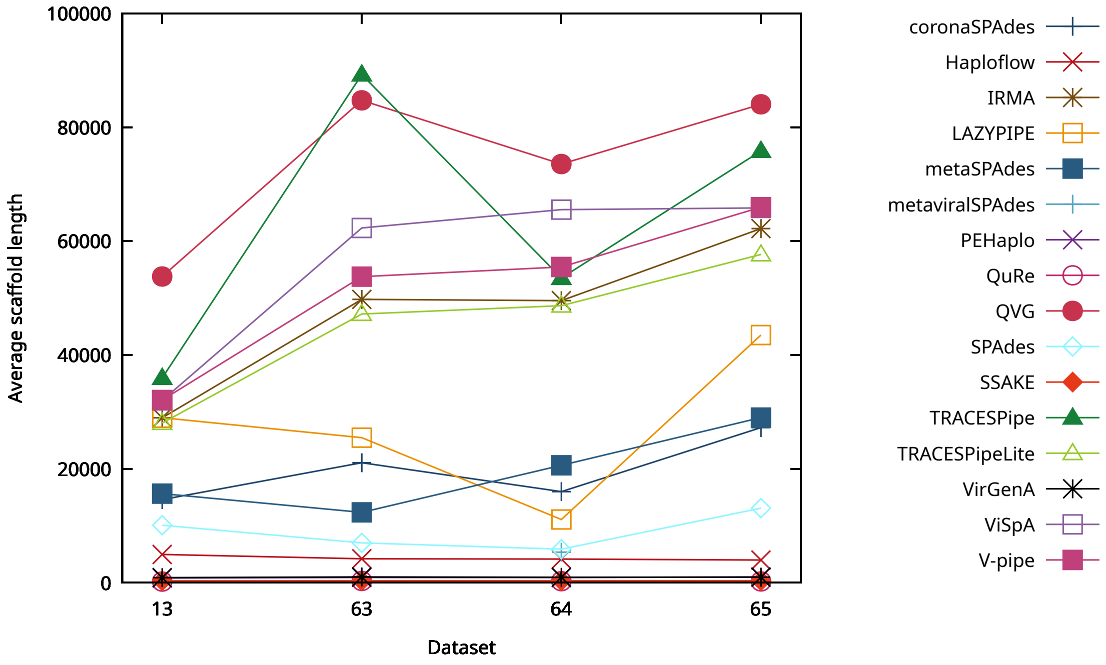

Figure S86: Figure comparing the performance of the reconstruction programs in terms of the average number of reconstructed bases per scaffold for datasets with different viral compositions (datasets 13, 63, 64 and 65).

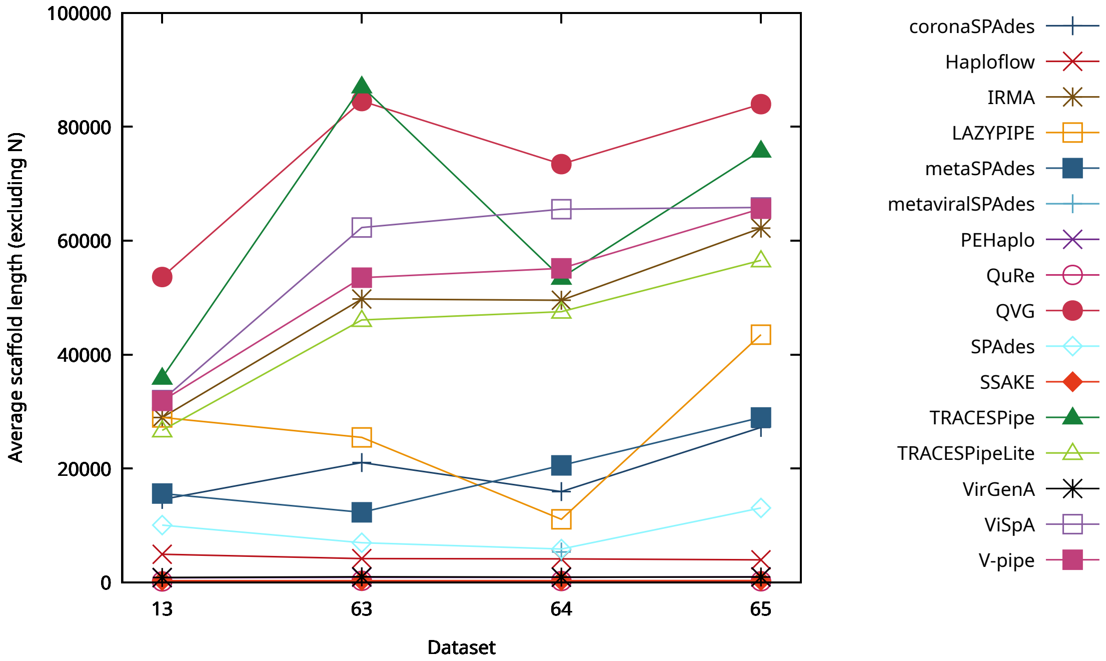

Figure S87: Figure comparing the performance of the reconstruction programs in terms of the average number of reconstructed bases per scaffold (excluding "N") for datasets with different viral compositions (datasets 13, 63, 64 and 65).

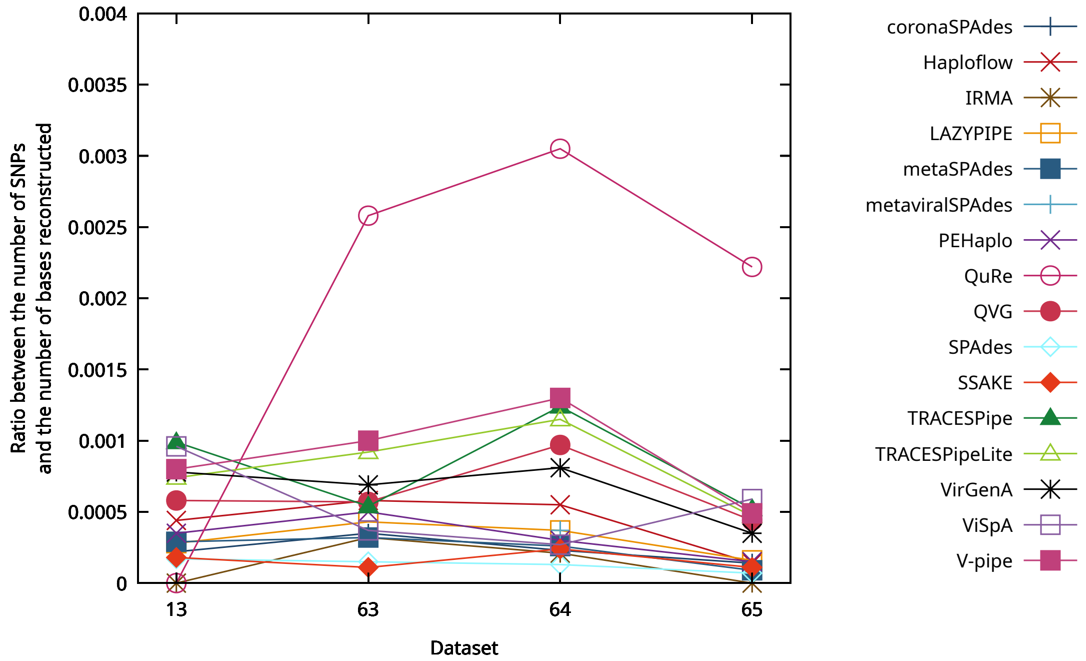

Figure S88: Figure comparing the performance of the reconstruction programs in terms of the ratio between the number of SNPs and the number of bases reconstructed for datasets with different viral compositions (datasets 13, 63, 64 and 65).

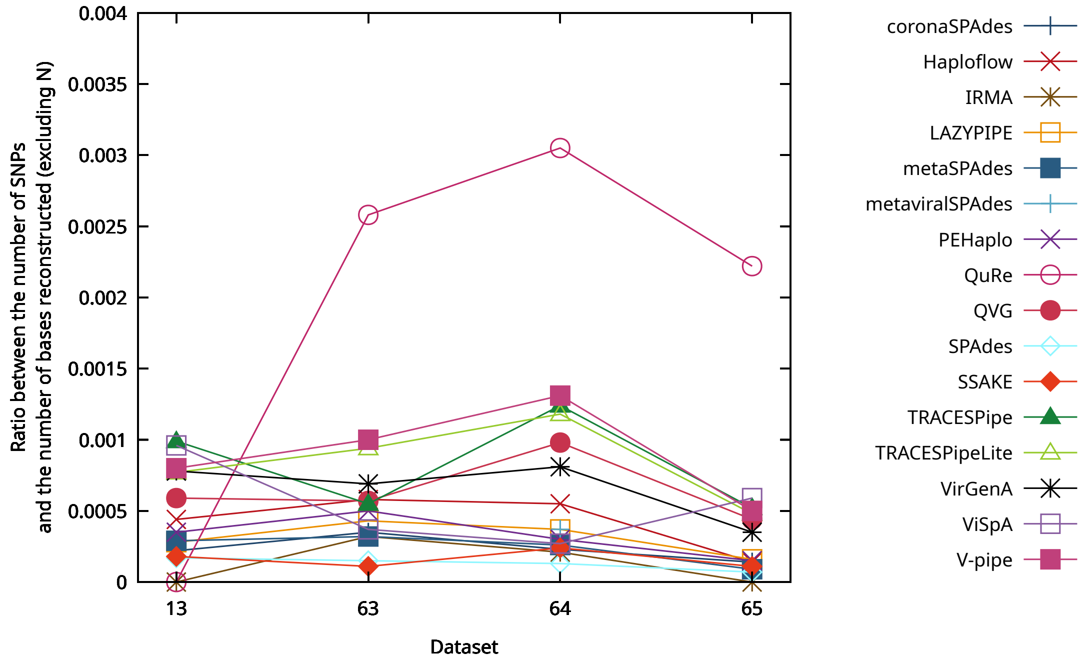

Figure S89: Figure comparing the performance of the reconstruction programs in terms of the ratio between the number of SNPs and the number of bases reconstructed (excluding "N") for datasets with different viral compositions (datasets 13, 63, 64 and 65).

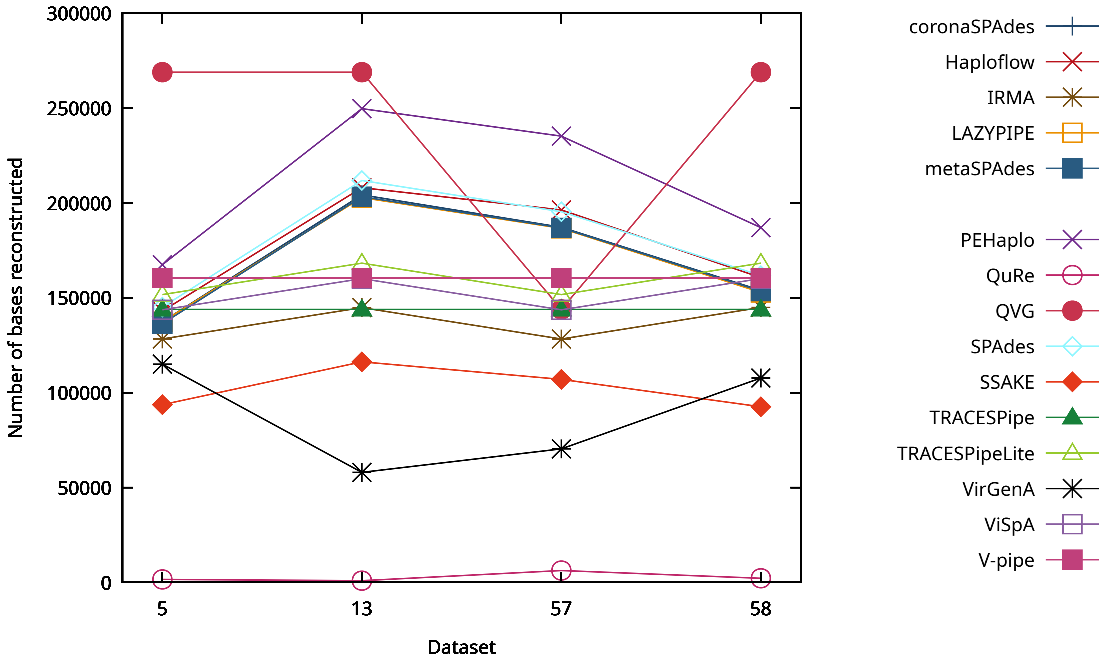

Figure S90: Figure comparing the performance of the reconstruction programs in terms of the number of bases reconstructed for datasets 5, 13, 57 and 58.

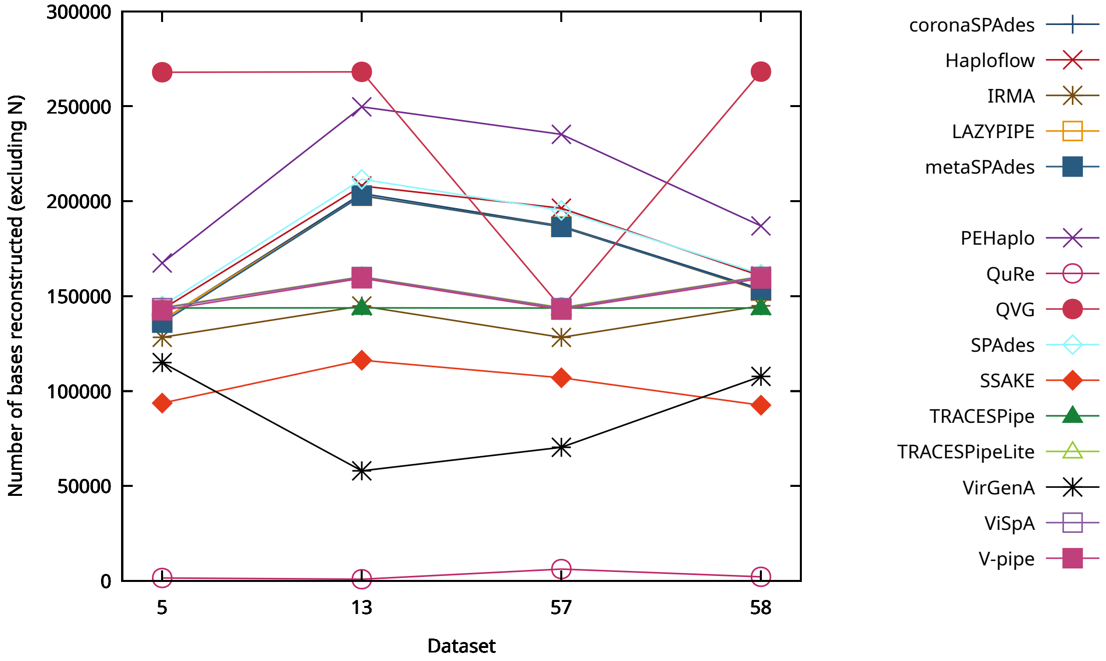

Figure S91: Figure comparing the performance of the reconstruction programs in terms of the number of bases reconstructed (excluding "N") for datasets 5, 13, 57 and 58.

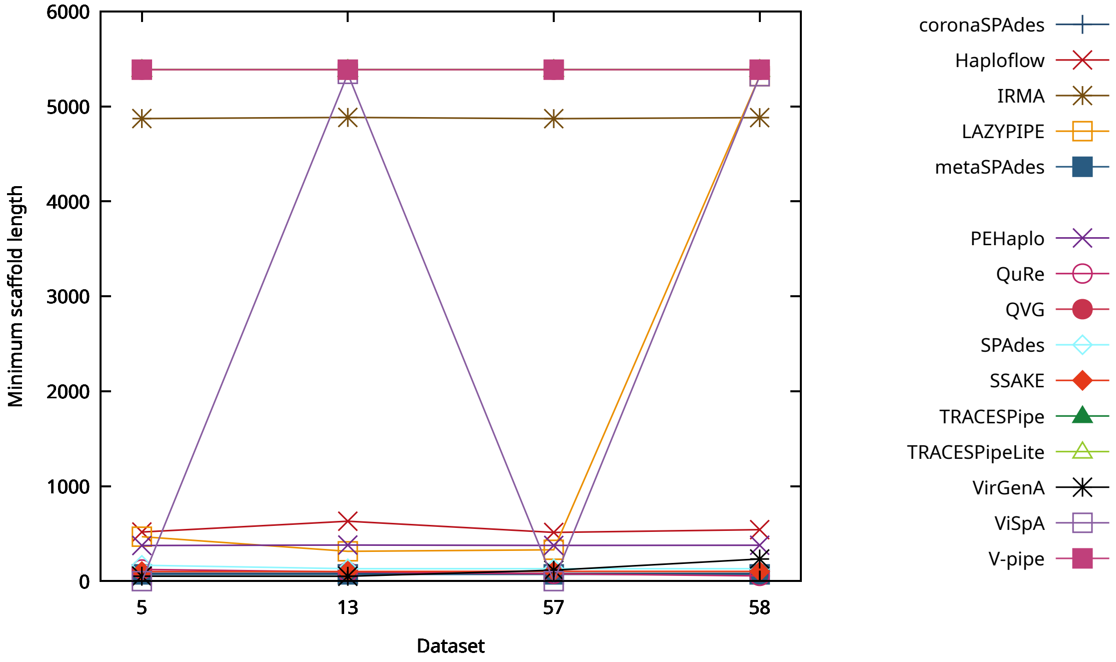

Figure S92: Figure comparing the performance of the reconstruction programs in terms of the minimum number of reconstructed bases per scaffold for datasets 5, 13, 57 and 58.

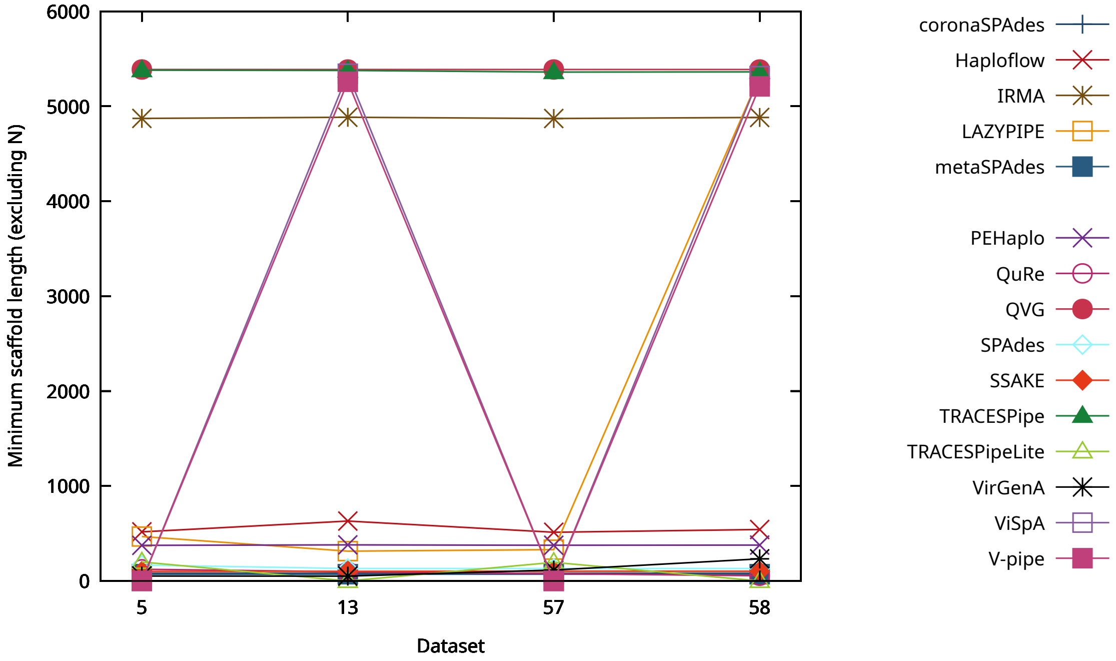

Figure S93: Figure comparing the performance of the reconstruction programs in terms of the minimum number of reconstructed bases per scaffold (excluding "N") for datasets 5, 13, 57 and 58.

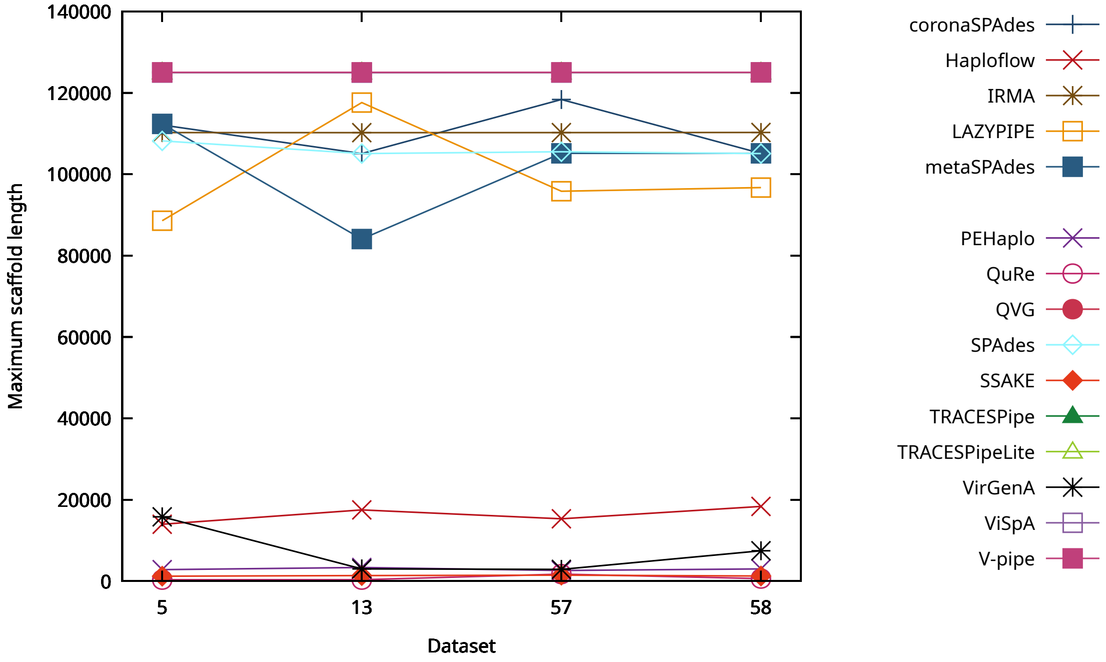

Figure S94: Figure comparing the performance of the reconstruction programs in terms of the maximum number of reconstructed bases per scaffold for datasets 5, 13, 57 and 58.

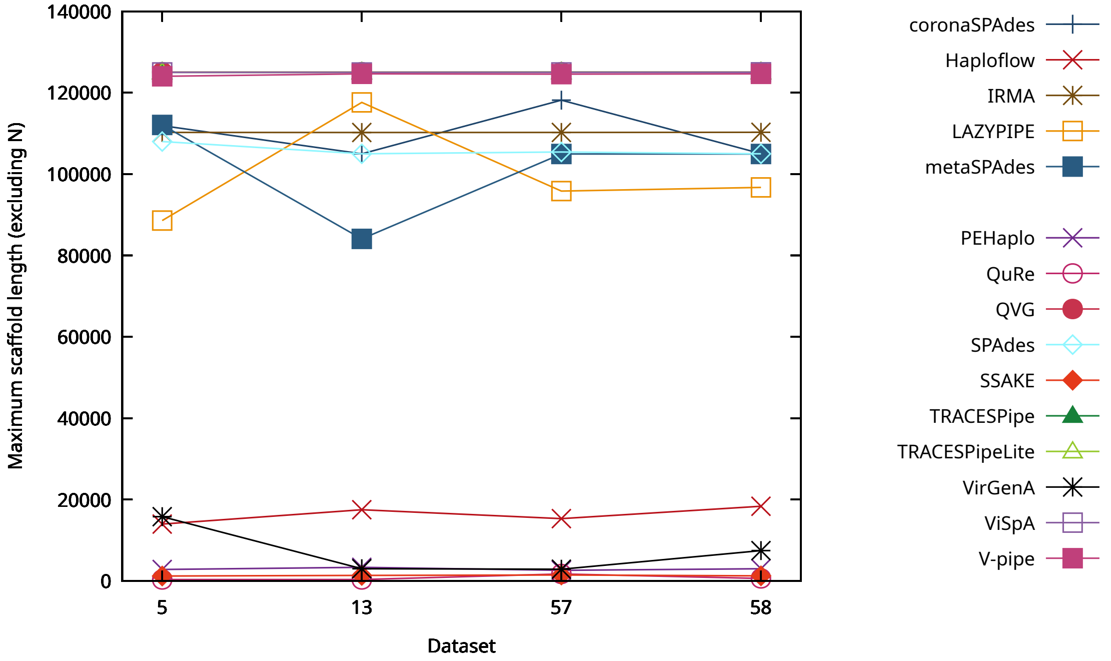

Figure S95: Figure comparing the performance of the reconstruction programs in terms of the maximum number of reconstructed bases per scaffold (excluding "N") for datasets 5, 13, 57 and 58.

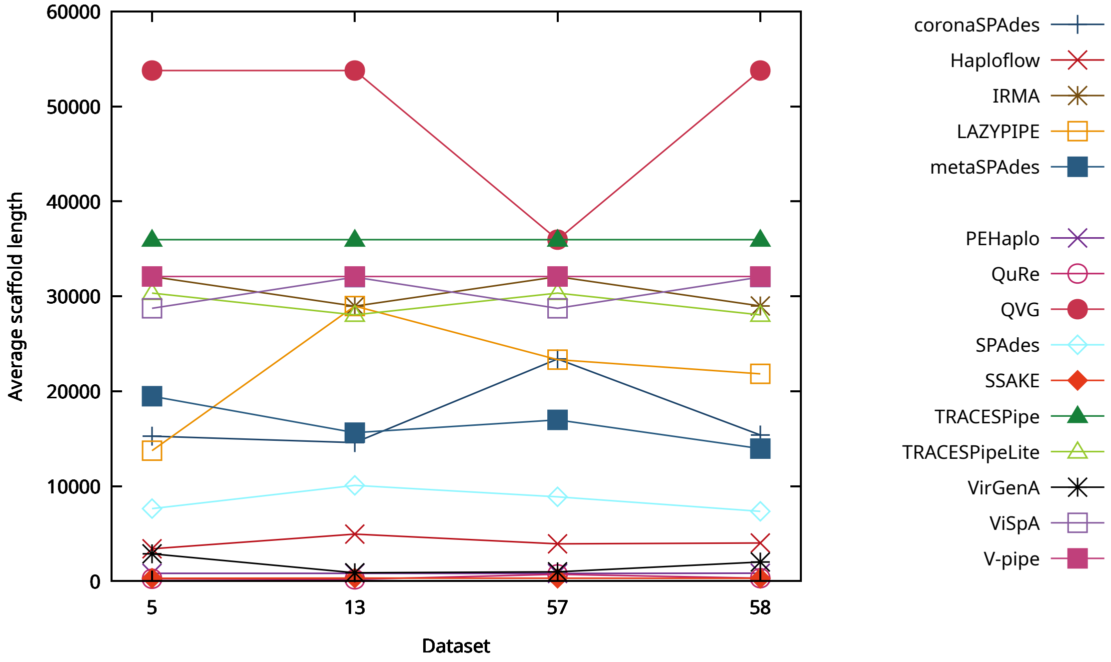

Figure S96: Figure comparing the performance of the reconstruction programs in terms of the average number of reconstructed bases per scaffold for datasets 5, 13, 57 and 58.

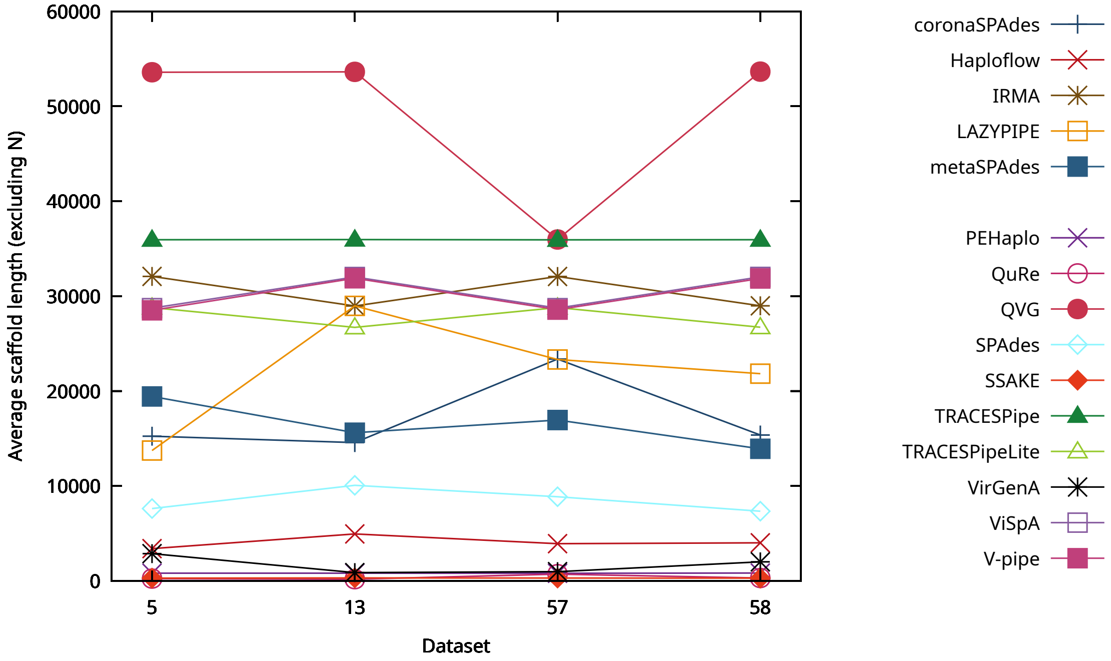

Figure S97: Figure comparing the performance of the reconstruction programs in terms of the average number of reconstructed bases per scaffold (excluding "N") for datasets 5, 13, 57 and 58.

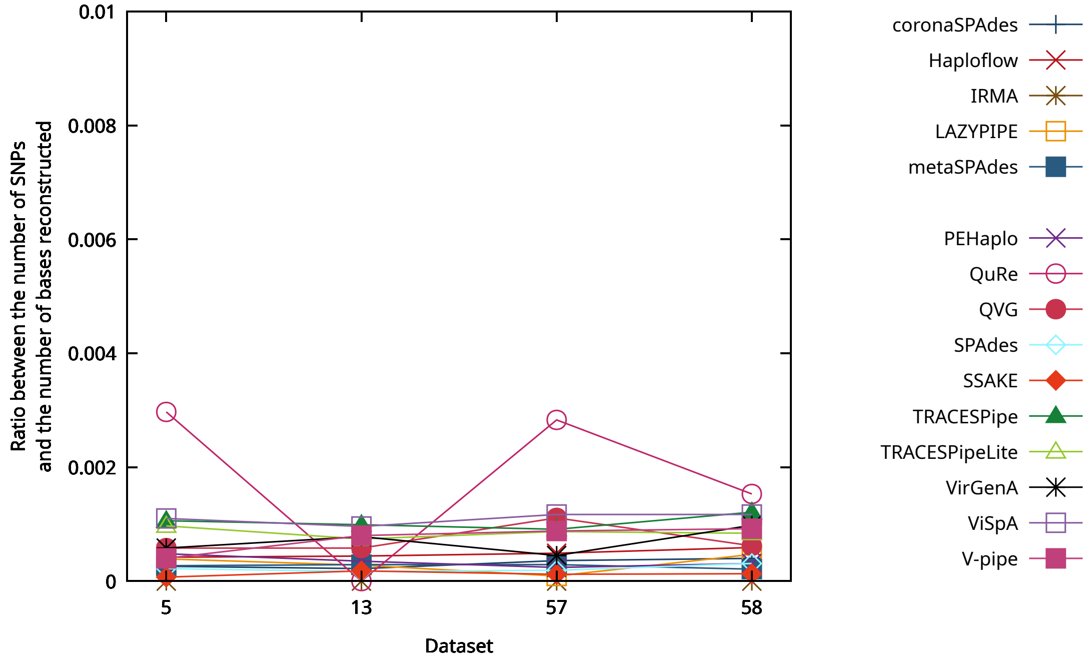

Figure S98: Figure comparing the performance of the reconstruction programs in terms of the ratio between the number of SNPs and the number of bases reconstructed for datasets 5, 13, 57 and 58.

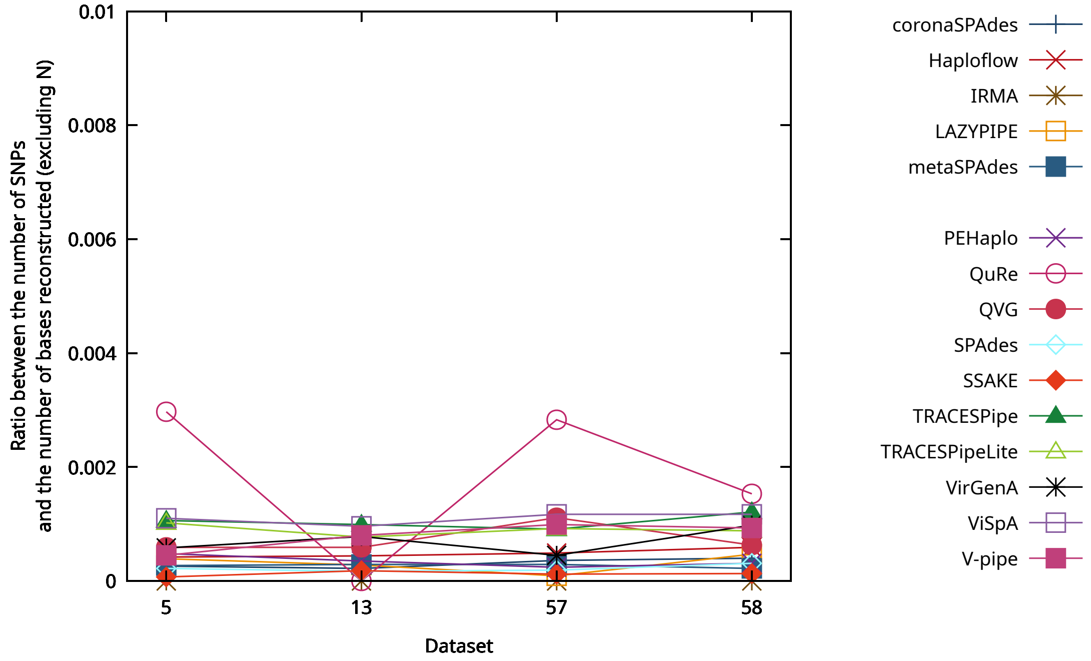

Figure S99: Figure comparing the performance of the reconstruction programs in terms of the ratio between the number of SNPs and the number of bases reconstructed (excluding "N") for datasets 5, 13, 57 and 58.

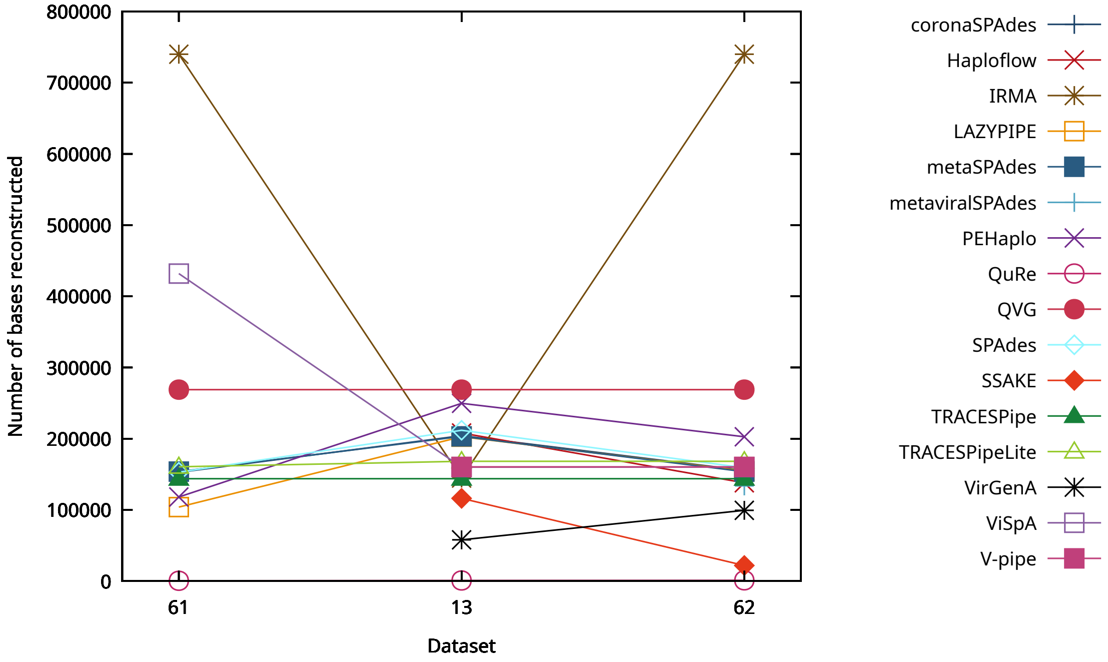

Figure S100: Figure comparing the performance of the reconstruction programs in terms of the number of bases reconstructed for datasets with different read lengths (datasets 61, 13, and 62).

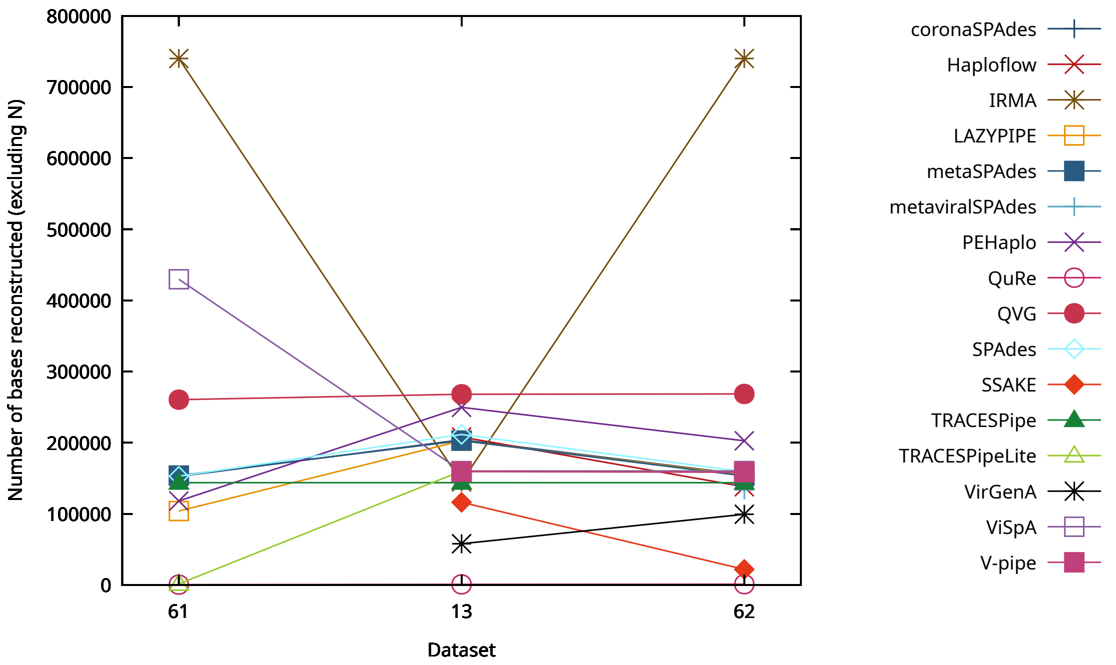

Figure S101: Figure comparing the performance of the reconstruction programs in terms of the number of bases reconstructed (excluding "N") for datasets with different read lengths (datasets 61, 13, and 62).

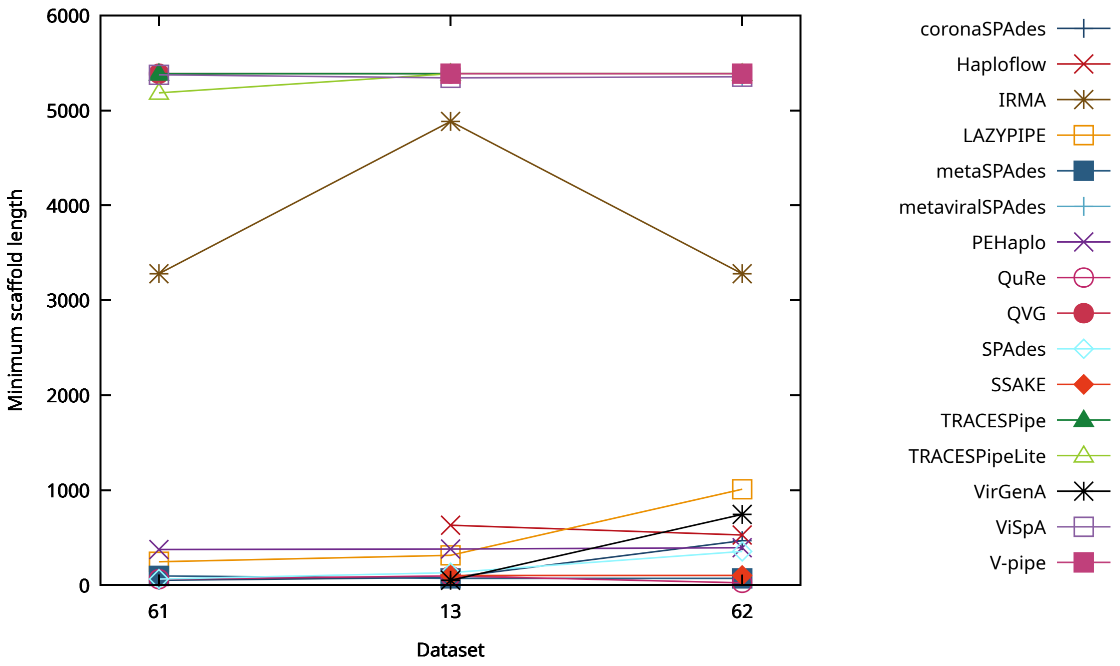

Figure S102: Figure comparing the performance of the reconstruction programs in terms of the minimum number of reconstructed bases per scaffold for datasets with different read lengths (datasets 61, 13, and 62).

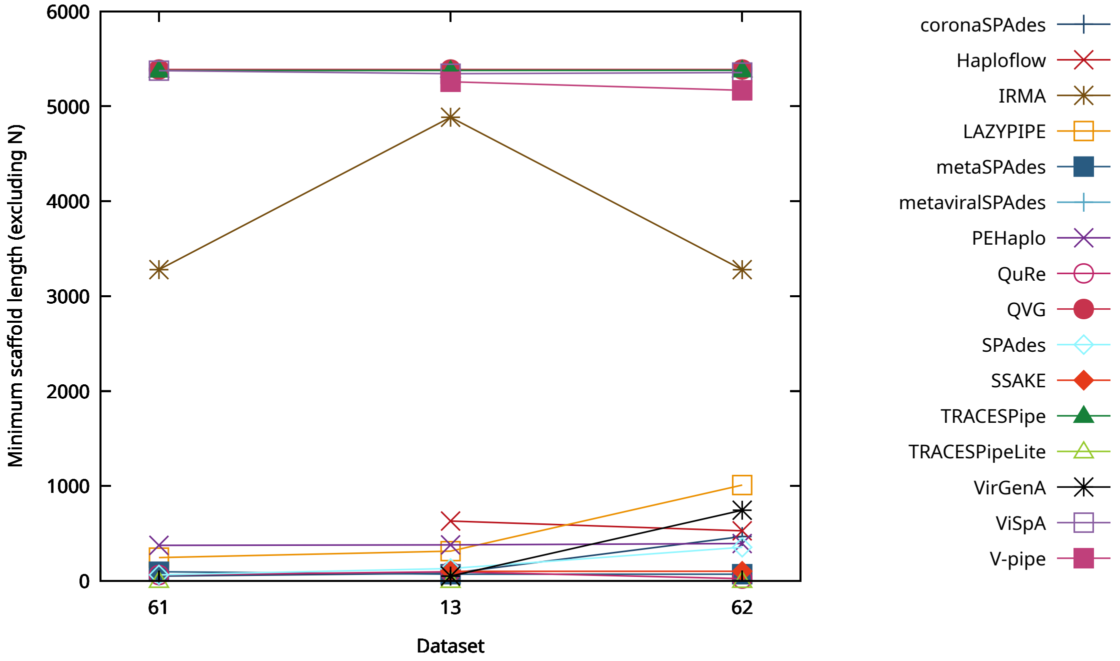

Figure S103: Figure comparing the performance of the reconstruction programs in terms of the minimum number of reconstructed bases per scaffold (excluding "N") for datasets with different read lengths (datasets 61, 13, and 62).

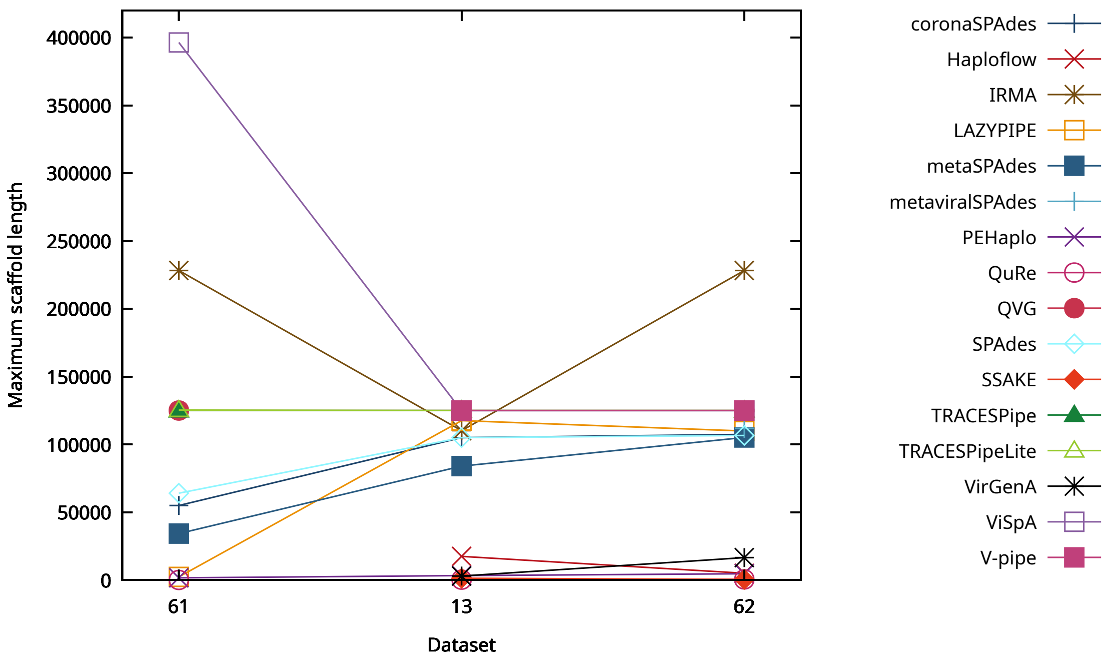

Figure S104: Figure comparing the performance of the reconstruction programs in terms of the maximum number of reconstructed bases per scaffold for datasets with different read lengths (datasets 61, 13, and 62).

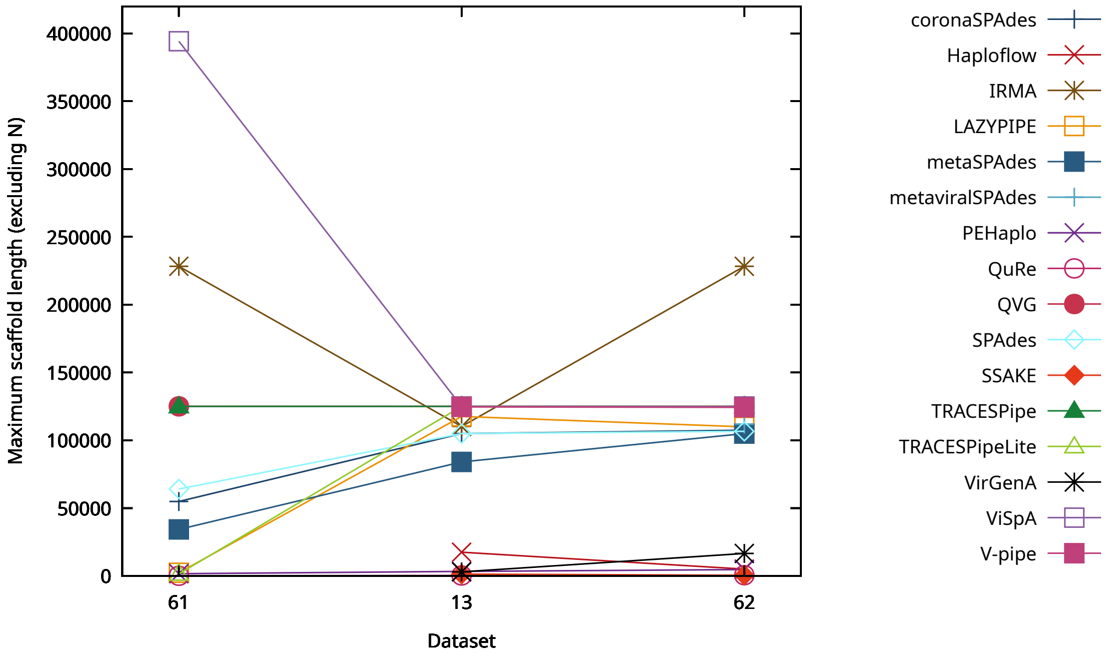

Figure S105: Figure comparing the performance of the reconstruction programs in terms of the maximum number of reconstructed bases per scaffold (excluding "N") for datasets with different read lengths (datasets 61, 13, and 62).

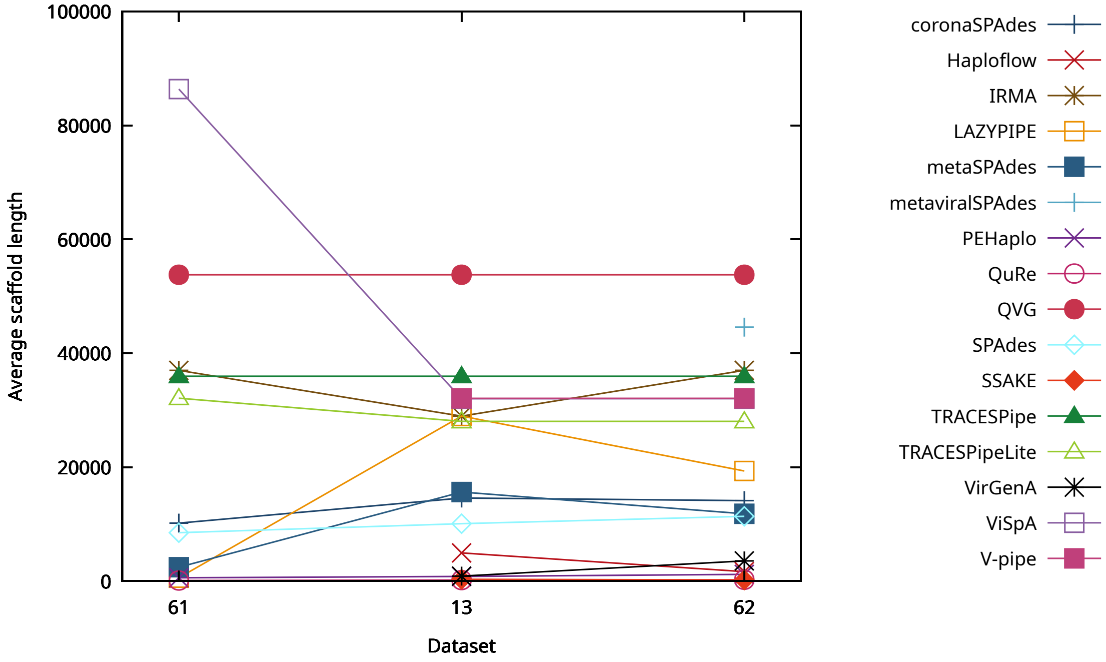

Figure S106: Figure comparing the performance of the reconstruction programs in terms of the average number of reconstructed bases per scaffold for datasets with different read lengths (datasets 61, 13, and 62).

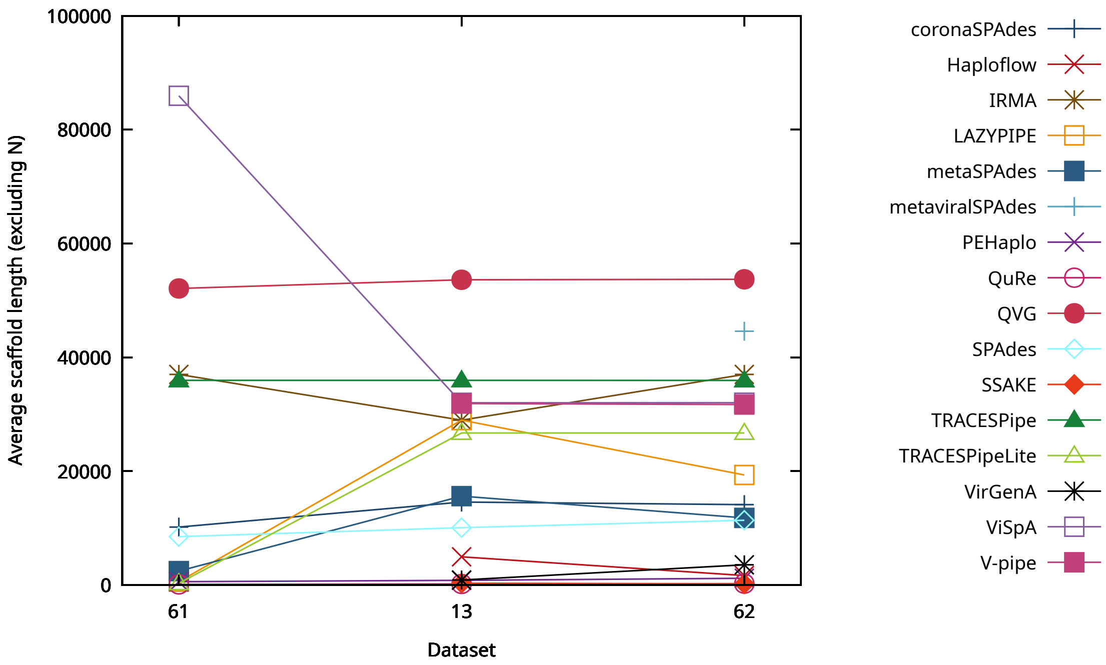

Figure S107: Figure comparing the performance of the reconstruction programs in terms of the average number of reconstructed bases per scaffold (excluding "N") for datasets with different read lengths (datasets 61, 13, and 62).

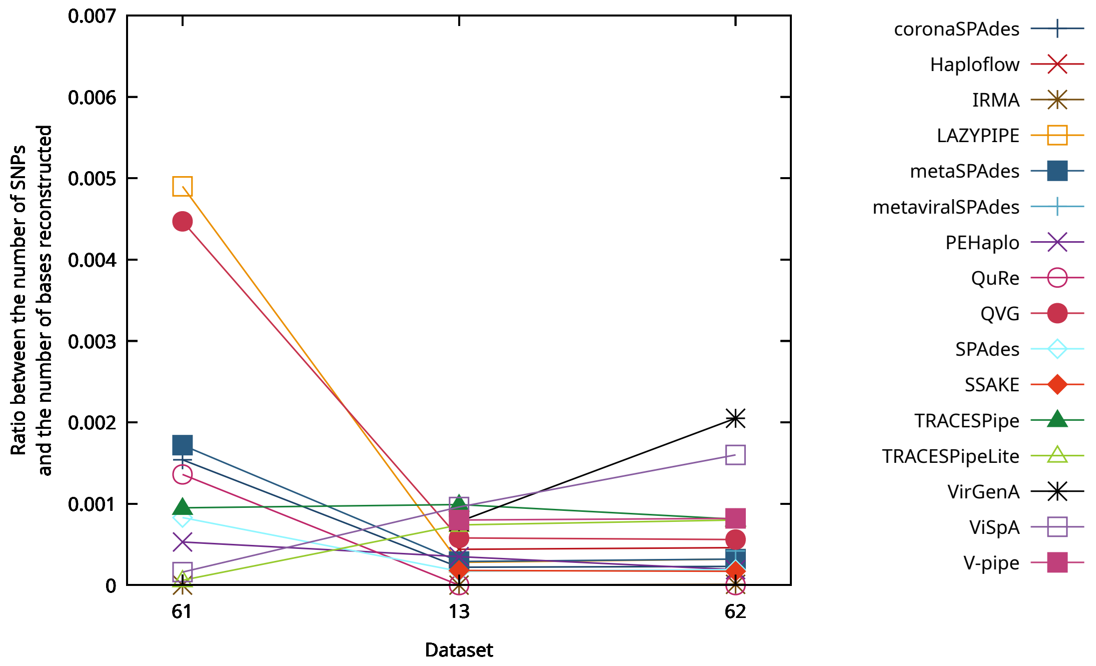

Figure S108: Figure comparing the performance of the reconstruction programs in terms of the ratio between the number of SNPs and the number of bases reconstructed for datasets with different read lengths (datasets 61, 13, and 62).

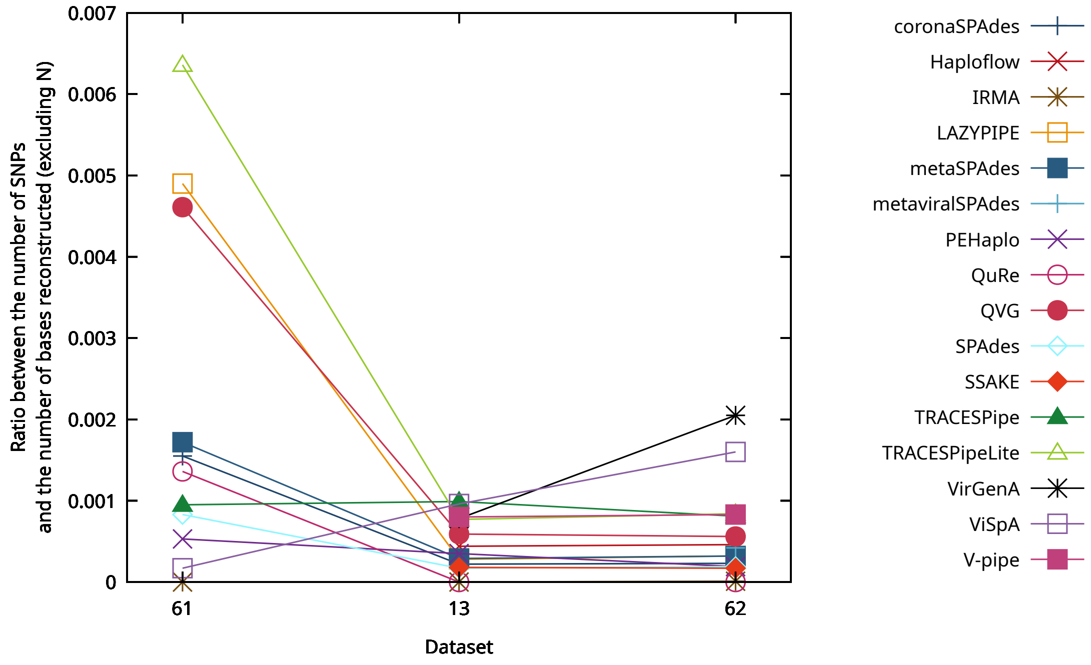

Figure S109: Figure comparing the performance of the reconstruction programs in terms of the ratio between the number of SNPs and the number of bases reconstructed (excluding "N") for datasets with different read lengths (datasets 61, 13, and 62).

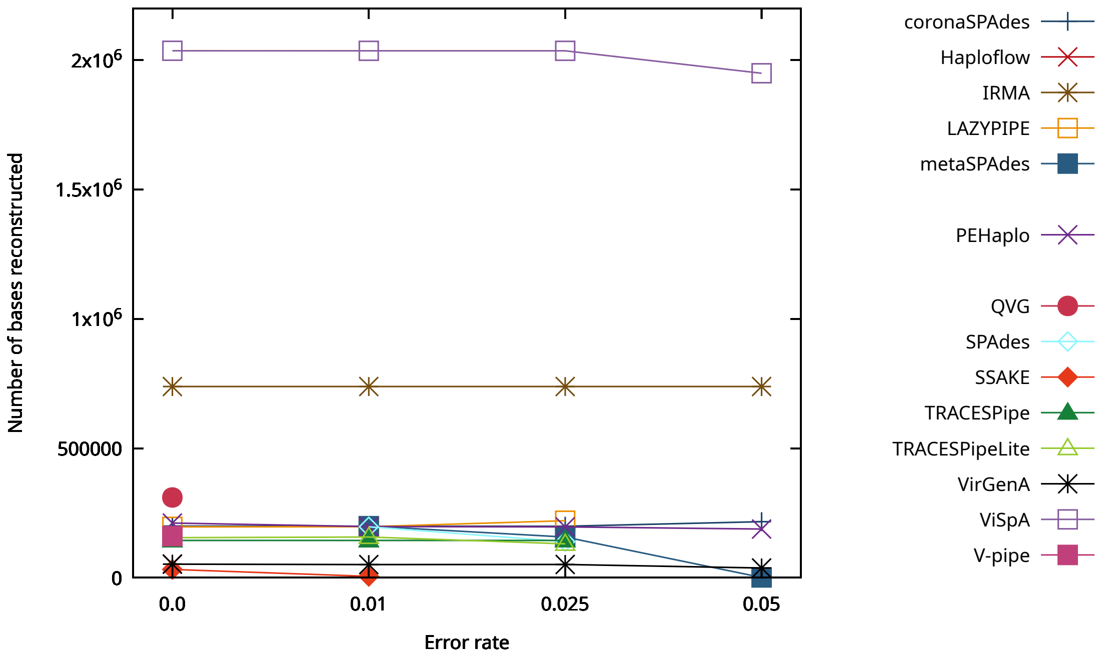

Figure S110: Figure comparing the performance of the reconstruction programs in terms of the number of bases reconstructed for datasets with error rates between 0.0 and 0.05 and depth coverage equal to 5x (DS66 to DS69).

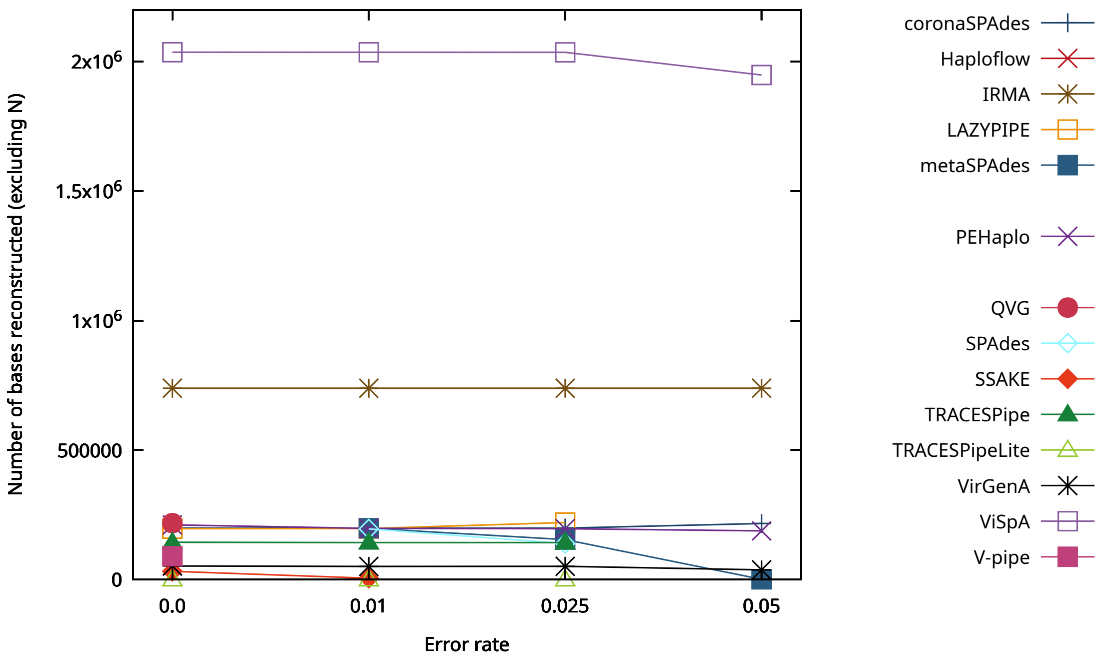

Figure S111: Figure comparing the performance of the reconstruction programs in terms of the number of bases reconstructed (excluding "N") for datasets with error rates between 0.0 and 0.05 and depth coverage equal to 5x (DS66 to DS69).

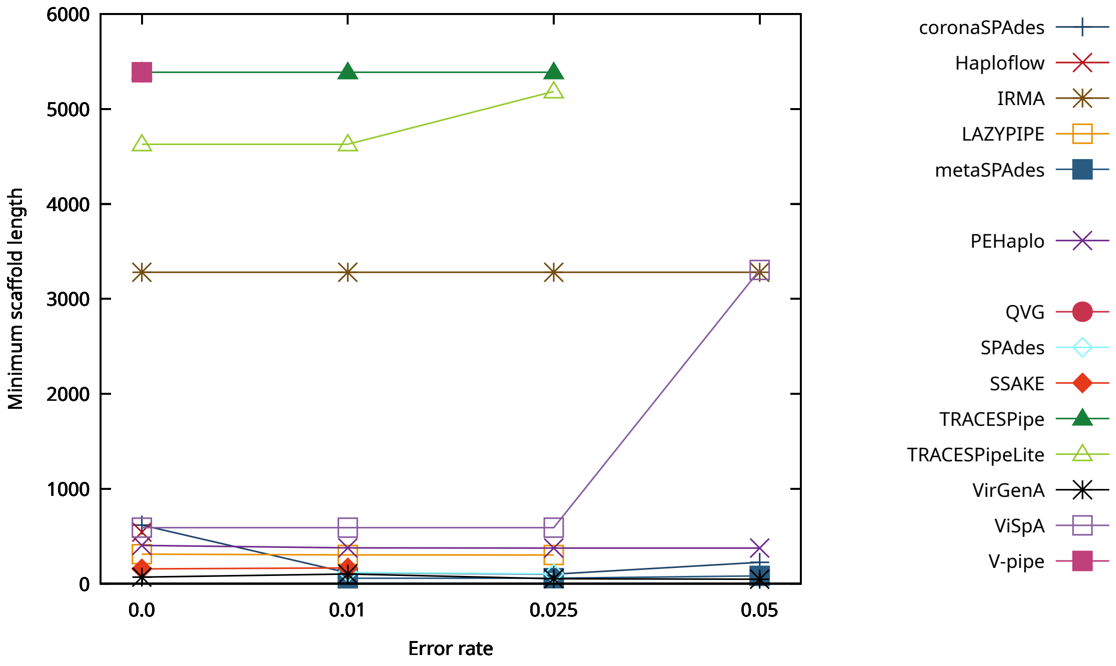

Figure S112: Figure comparing the performance of the reconstruction programs in terms of the minimum number of reconstructed bases per scaffold for datasets with error rates between 0.0 and 0.05 and depth coverage equal to 5x (DS66 to DS69).

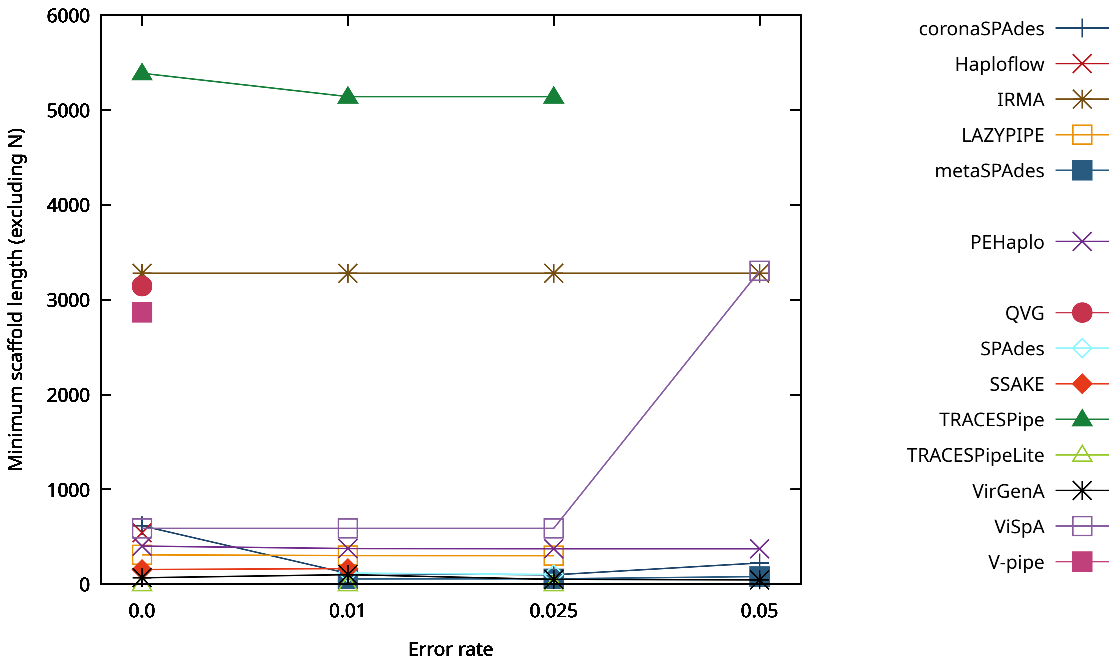

Figure S113: Figure comparing the performance of the reconstruction programs in terms of the minimum number of reconstructed bases per scaffold (excluding "N") for datasets with error rates between 0.0 and 0.05 and depth coverage equal to 5x (DS66 to DS69).

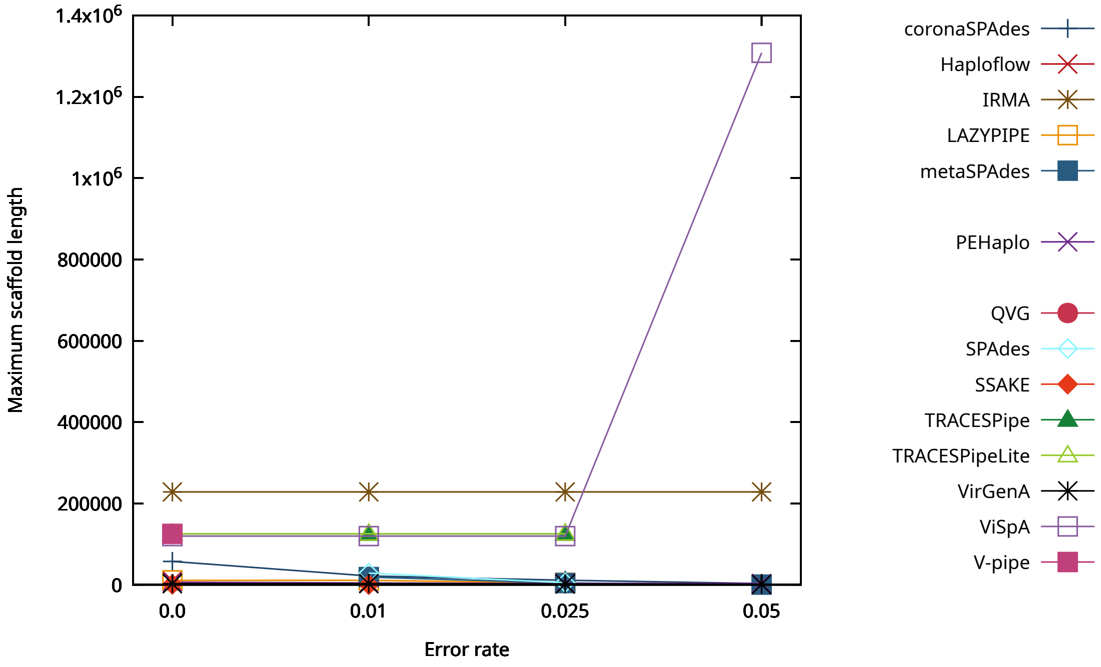

Figure S114: Figure comparing the performance of the reconstruction programs in terms of the maximum number of reconstructed bases per scaffold for datasets with error rates between 0.0 and 0.05 and depth coverage equal to 5x (DS66 to DS69).

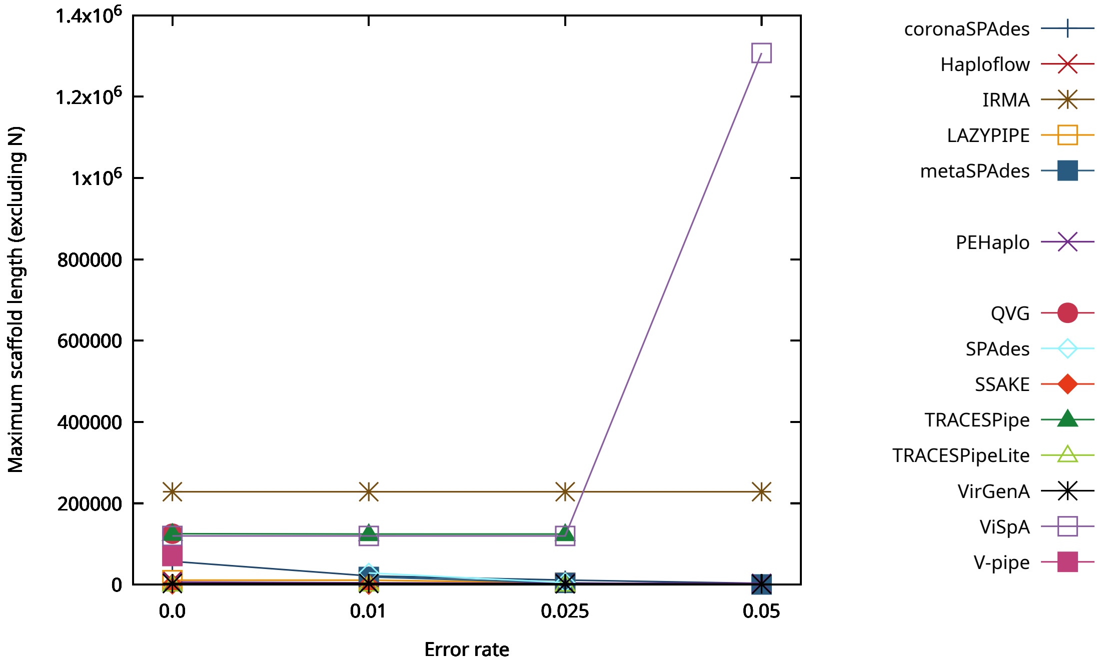

Figure S115: Figure comparing the performance of the reconstruction programs in terms of the maximum number of reconstructed bases per scaffold (excluding "N") for datasets with error rates between 0.0 and 0.05 and depth coverage equal to 5x (DS66 to DS69).

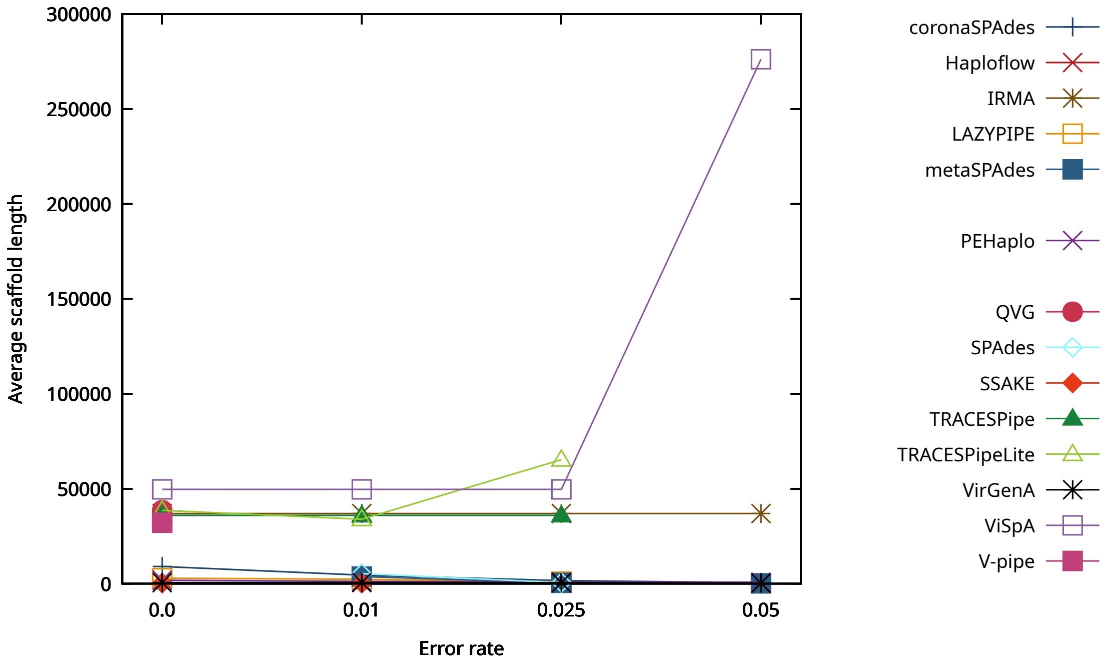

Figure S116: Figure comparing the performance of the reconstruction programs in terms of the average number of reconstructed bases per scaffold for datasets with error rates between 0.0 and 0.05 and depth coverage equal to 5x (DS66 to DS69).

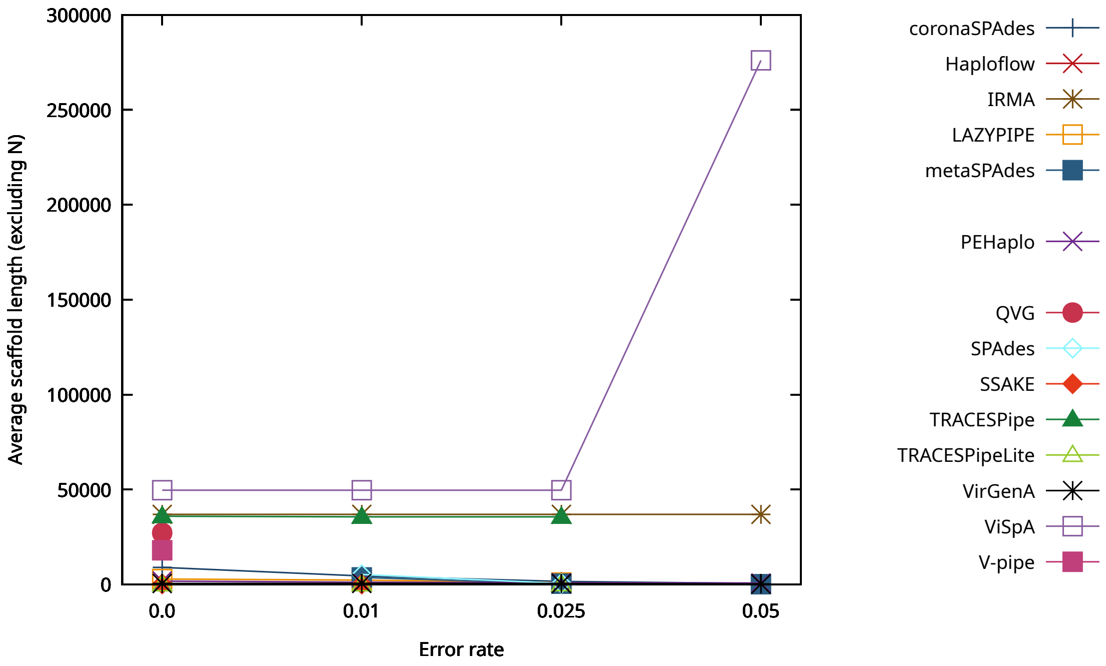

Figure S117: Figure comparing the performance of the reconstruction programs in terms of the average number of reconstructed bases per scaffold (excluding "N") for datasets with error rates between 0.0 and 0.05 and depth coverage equal to 5x (DS66 to DS69).

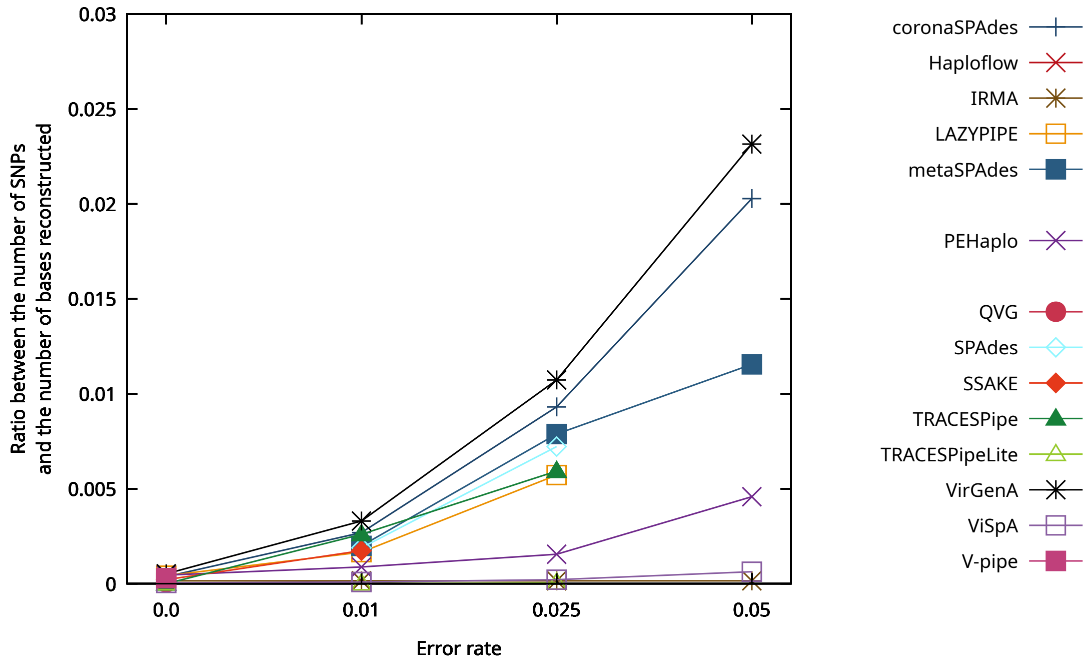

Figure S118: Figure comparing the performance of the reconstruction programs in terms of the ratio between the number of SNPs and the number of bases reconstructed for datasets with error rates between 0.0 and 0.05 and depth coverage equal to 5x (DS66 to DS69).

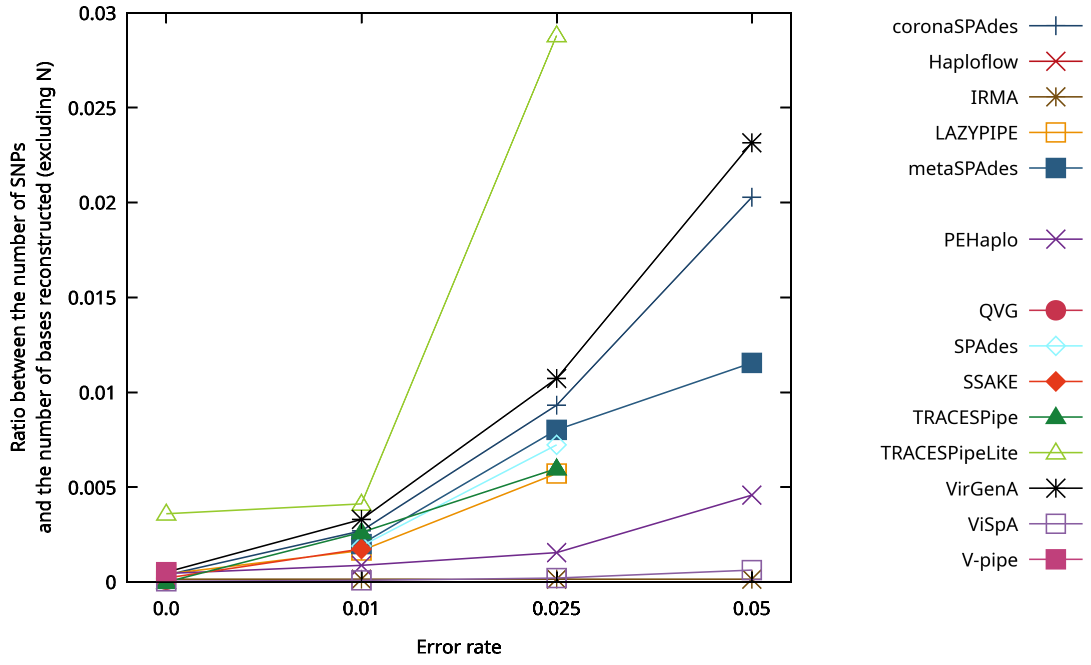

Figure S119: Figure comparing the performance of the reconstruction programs in terms of the ratio between the number of SNPs and the number of bases reconstructed (excluding "N") for datasets with error rates between 0.0 and 0.05 and depth coverage equal to 5x (DS66 to DS69).

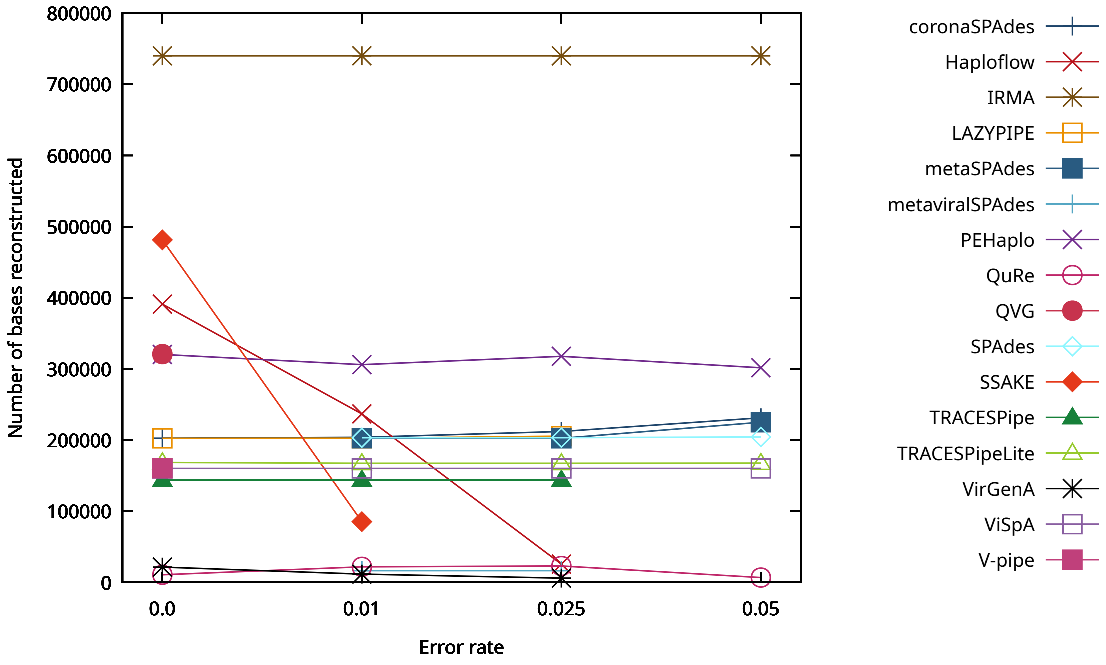

Figure S120: Figure comparing the performance of the reconstruction programs in terms of the number of bases reconstructed for datasets with error rates between 0.0 and 0.05 and depth coverage equal to 40x (DS70 to DS73).

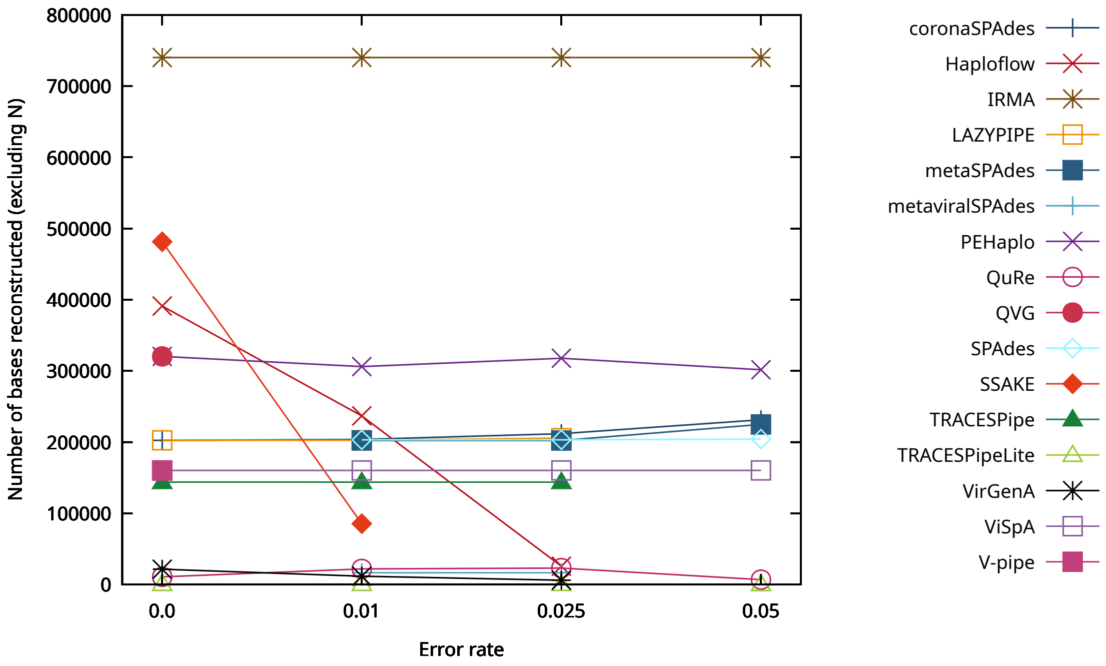

Figure S121: Figure comparing the performance of the reconstruction programs in terms of the number of bases reconstructed (excluding "N") for datasets with error rates between 0.0 and 0.05 and depth coverage equal to 40x (DS70 to DS73).

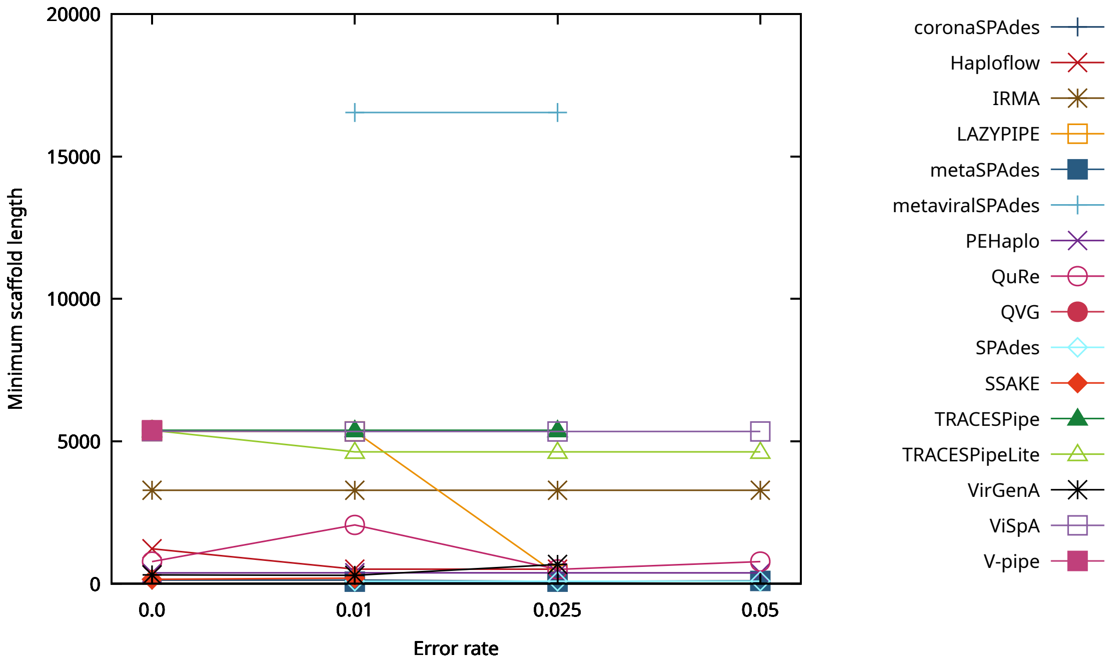

Figure S122: Figure comparing the performance of the reconstruction programs in terms of the minimum number of reconstructed bases per scaffold for datasets with error rates between 0.0 and 0.05 and depth coverage equal to 40x (DS70 to DS73).

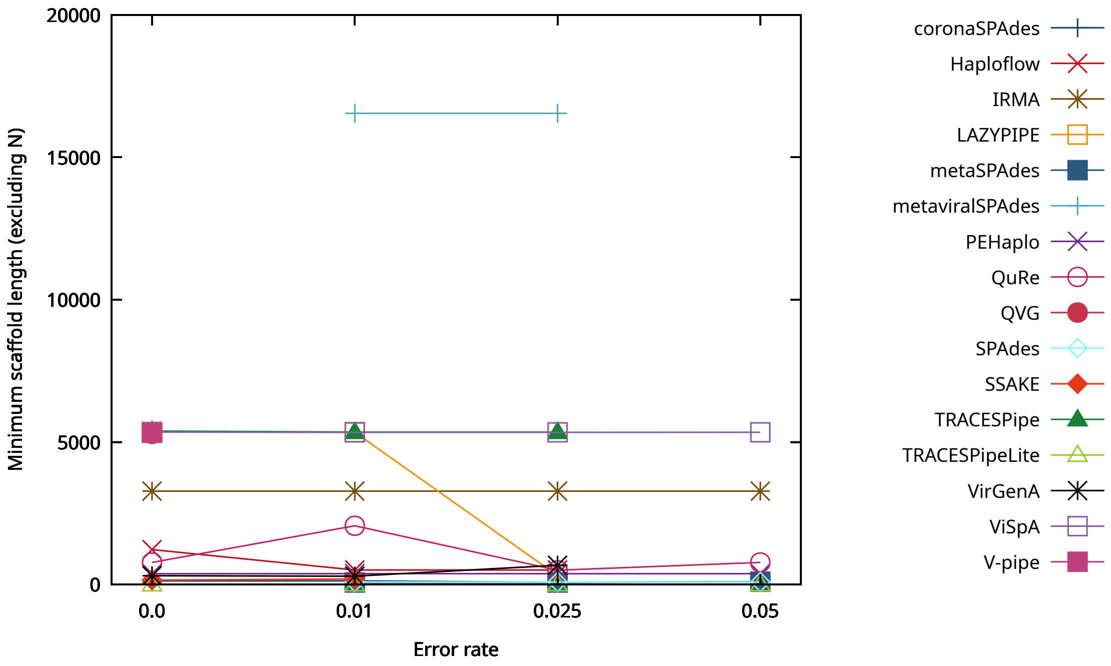

Figure S123: Figure comparing the performance of the reconstruction programs in terms of the minimum number of reconstructed bases per scaffold (excluding "N") for datasets with error rates between 0.0 and 0.05 and depth coverage equal to 40x (DS70 to DS73).

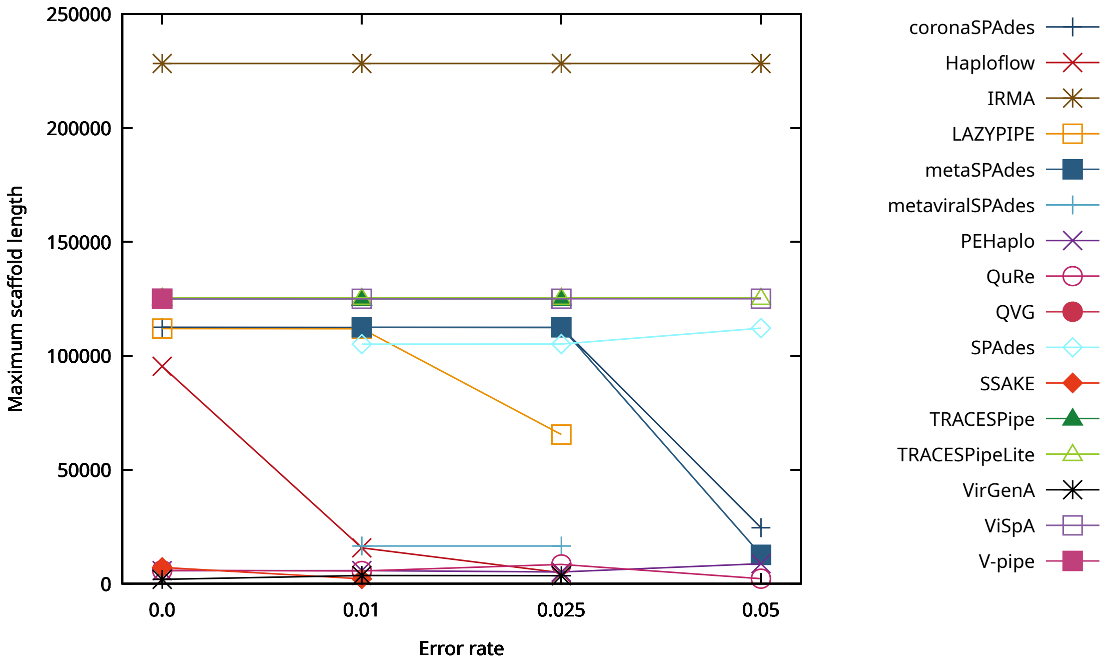

Figure S124: Figure comparing the performance of the reconstruction programs in terms of the maximum number of reconstructed bases per scaffold for datasets with error rates between 0.0 and 0.05 and depth coverage equal to 40x (DS70 to DS73).

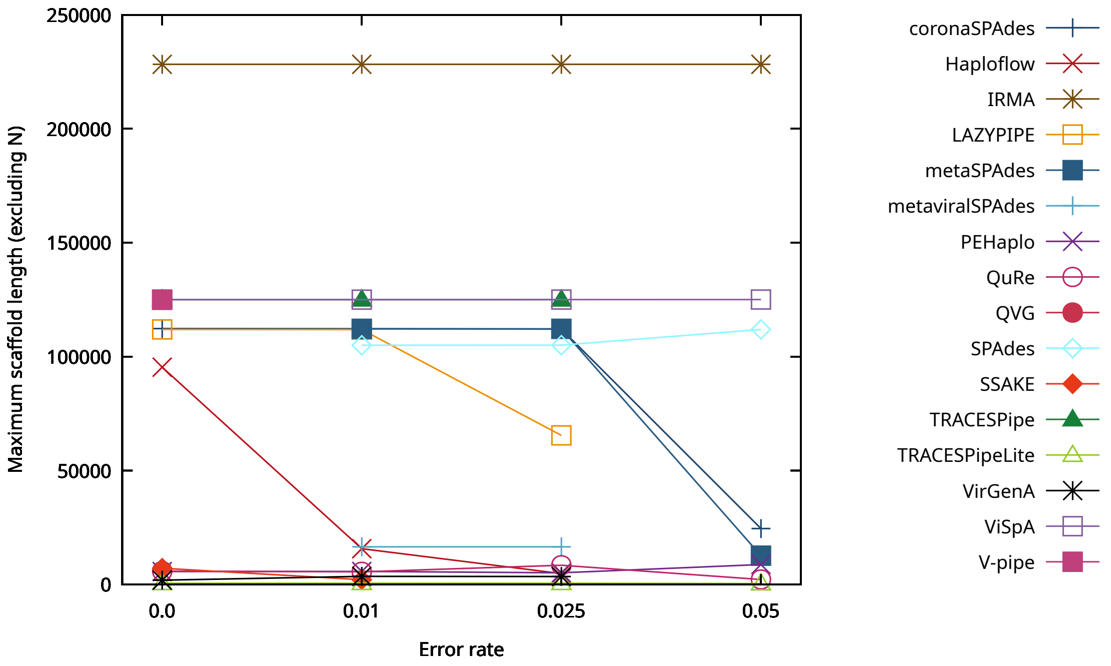

Figure S125: Figure comparing the performance of the reconstruction programs in terms of the maximum number of reconstructed bases per scaffold (excluding "N") for datasets with error rates between 0.0 and 0.05 and depth coverage equal to 40x (DS70 to DS73).

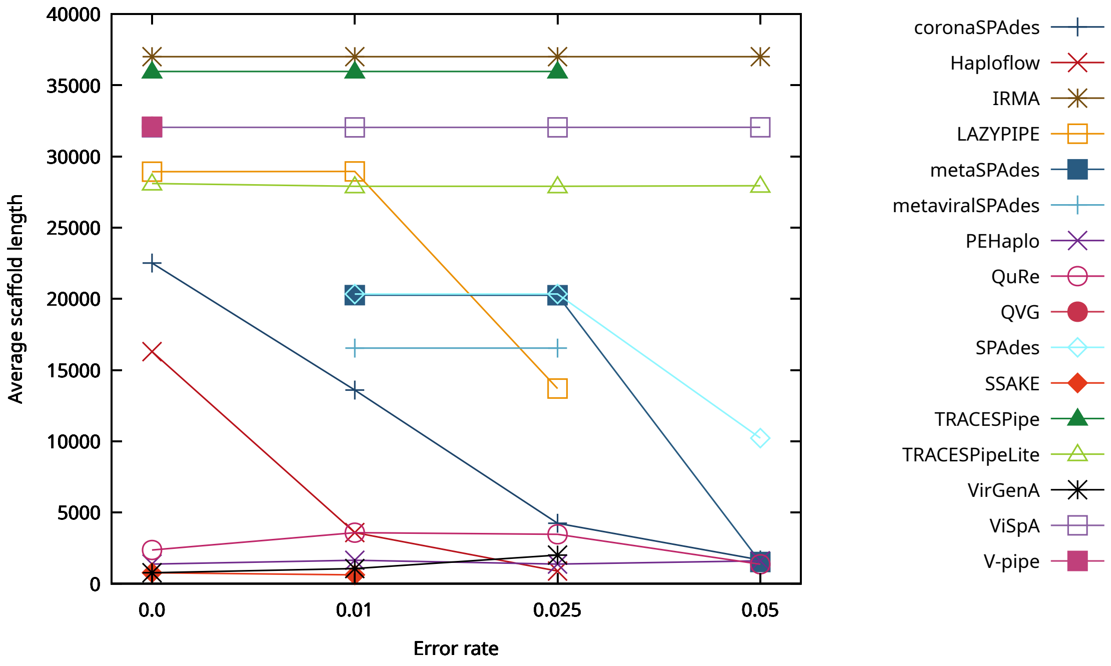

Figure S126: Figure comparing the performance of the reconstruction programs in terms of the average number of reconstructed bases per scaffold for datasets with error rates between 0.0 and 0.05 and depth coverage equal to 40x (DS70 to DS73).

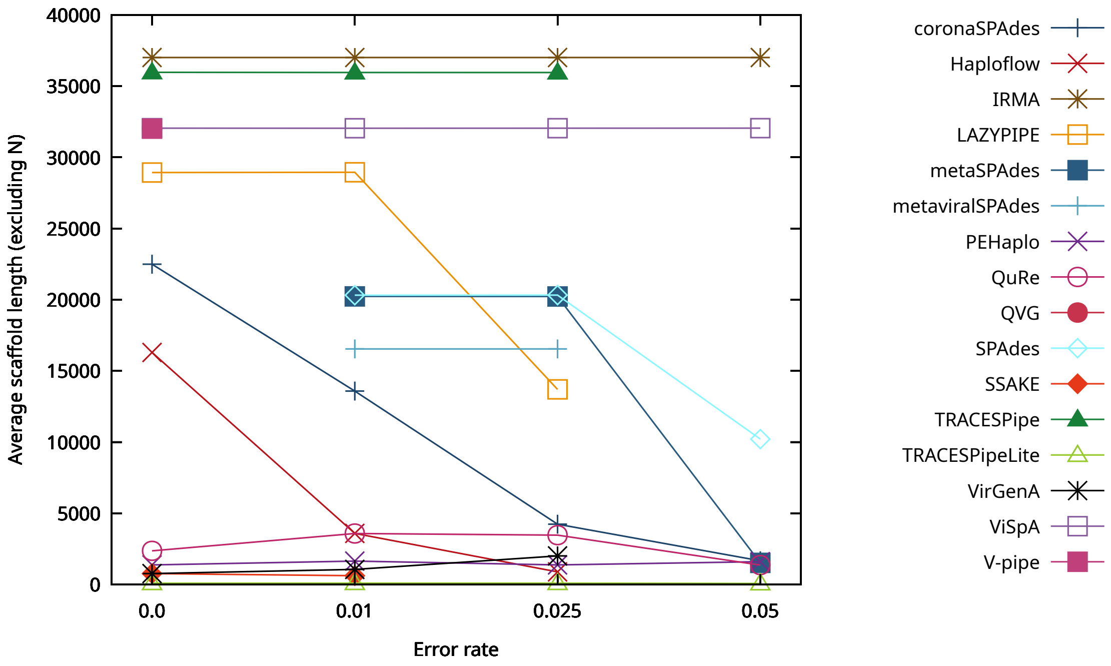

Figure S127: Figure comparing the performance of the reconstruction programs in terms of the average number of reconstructed bases per scaffold (excluding "N") for datasets with error rates between 0.0 and 0.05 and depth coverage equal to 40x (DS70 to DS73).

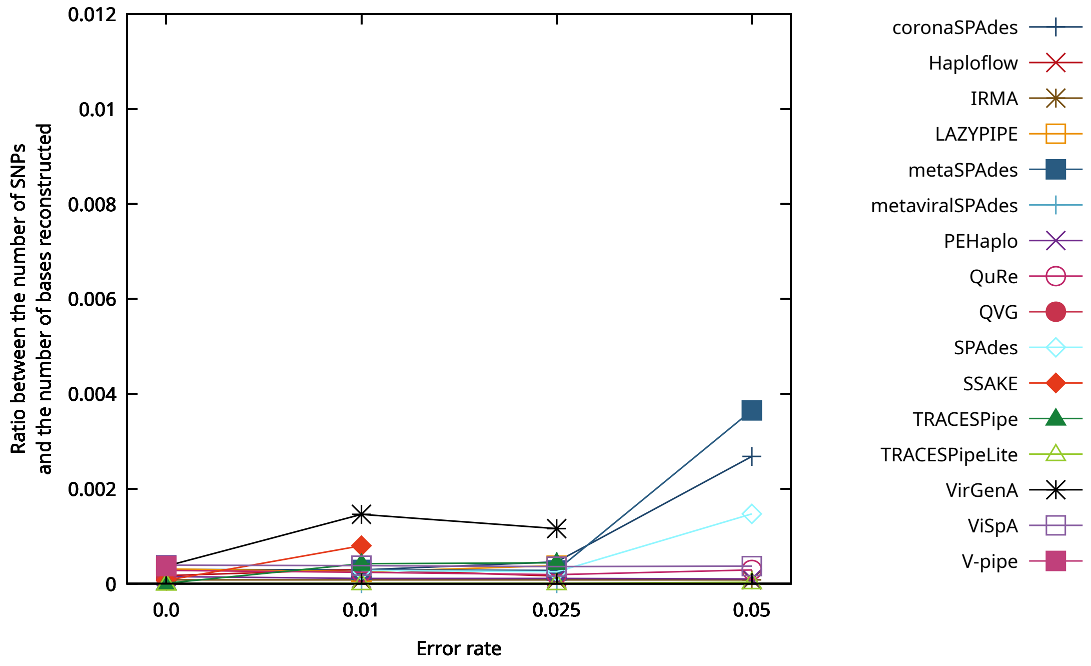

Figure S128: Figure comparing the performance of the reconstruction programs in terms of the ratio between the number of SNPs and the number of bases reconstructed for datasets with error rates between 0.0 and 0.05 and depth coverage equal to 40x (DS70 to DS73).

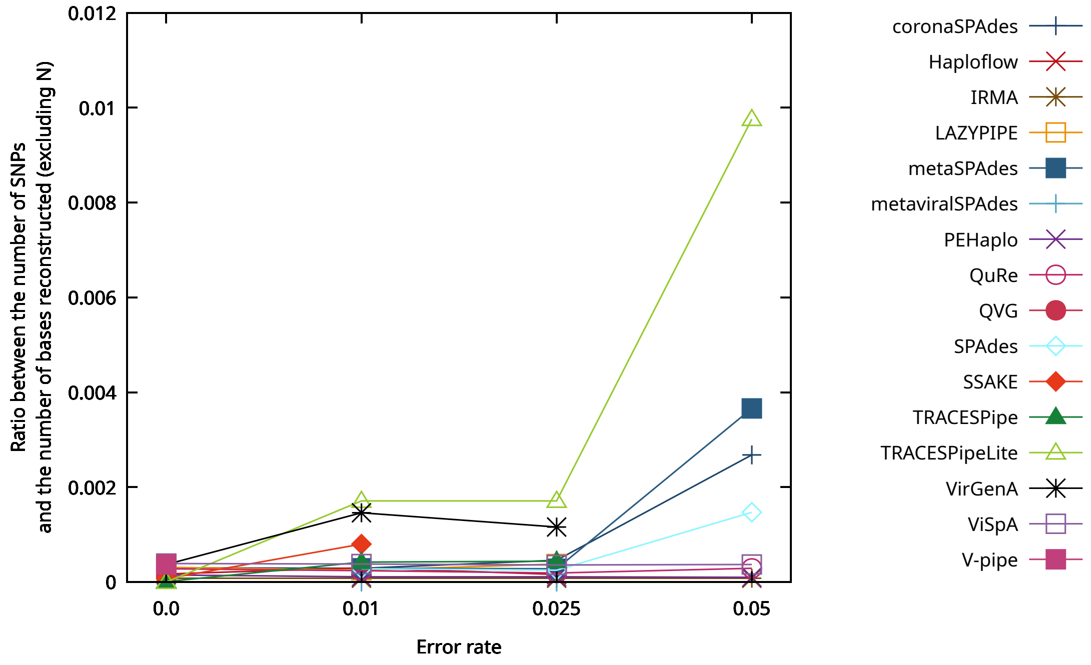

Figure S129: Figure comparing the performance of the reconstruction programs in terms of the ratio between the number of SNPs and the number of bases reconstructed (excluding "N") for datasets with error rates between 0.0 and 0.05 and depth coverage equal to 40x (DS70 to DS73).

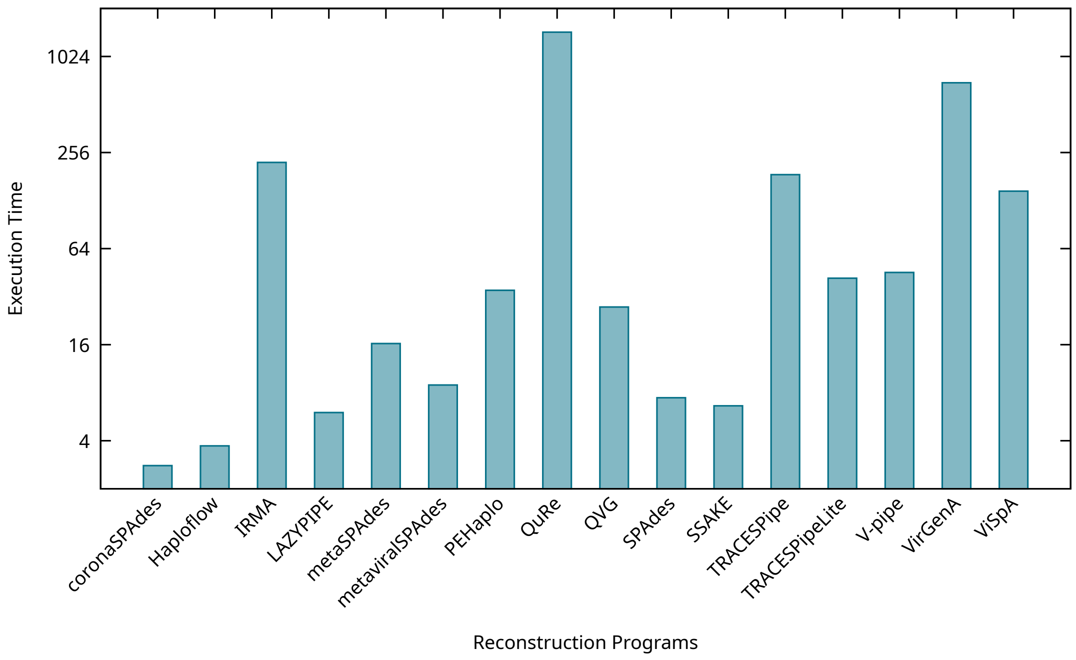

Figure S130: Figure comparing the average performance of the reconstruction programs in terms of the execution time across all datasets reconstructed. The y-axis is presented in a logarithmic scale of base 2.

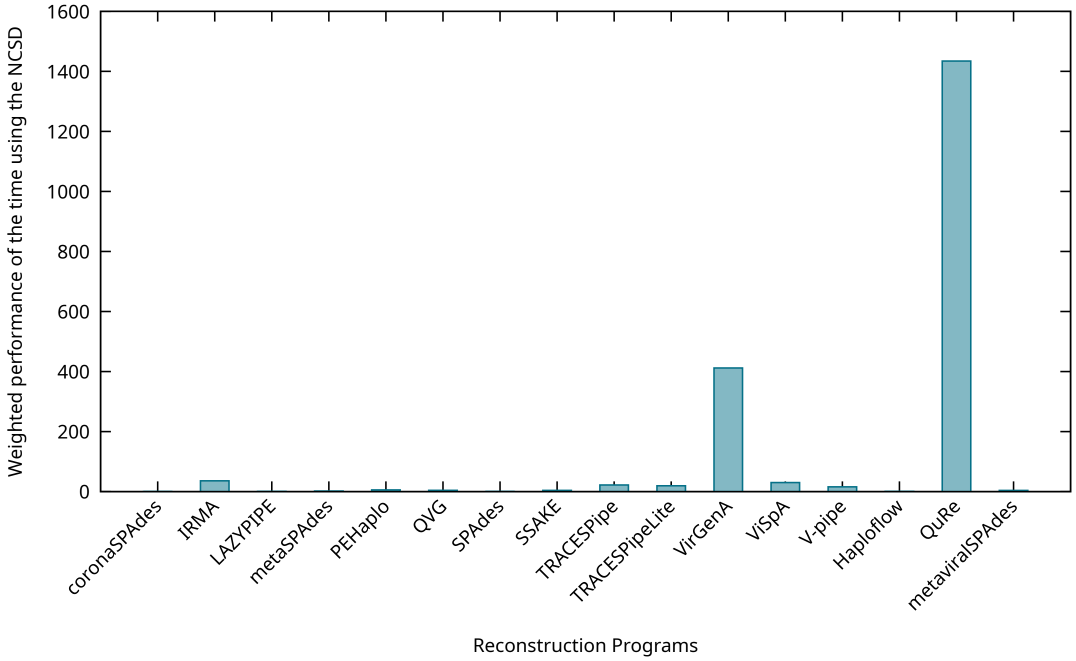

Figure S131: Figure comparing the average performance of the reconstruction programs in terms of the weighted execution time across all datasets reconstructed.

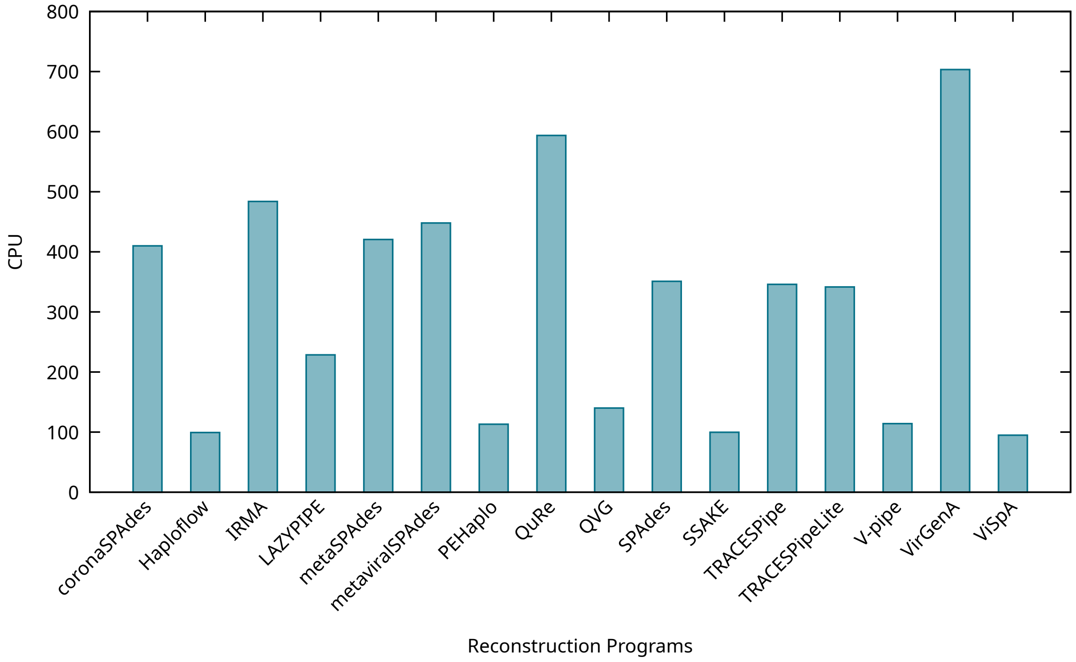

Figure S132: Figure comparing the average performance of the reconstruction programs in terms of the percentage of CPU used across all datasets reconstructed.

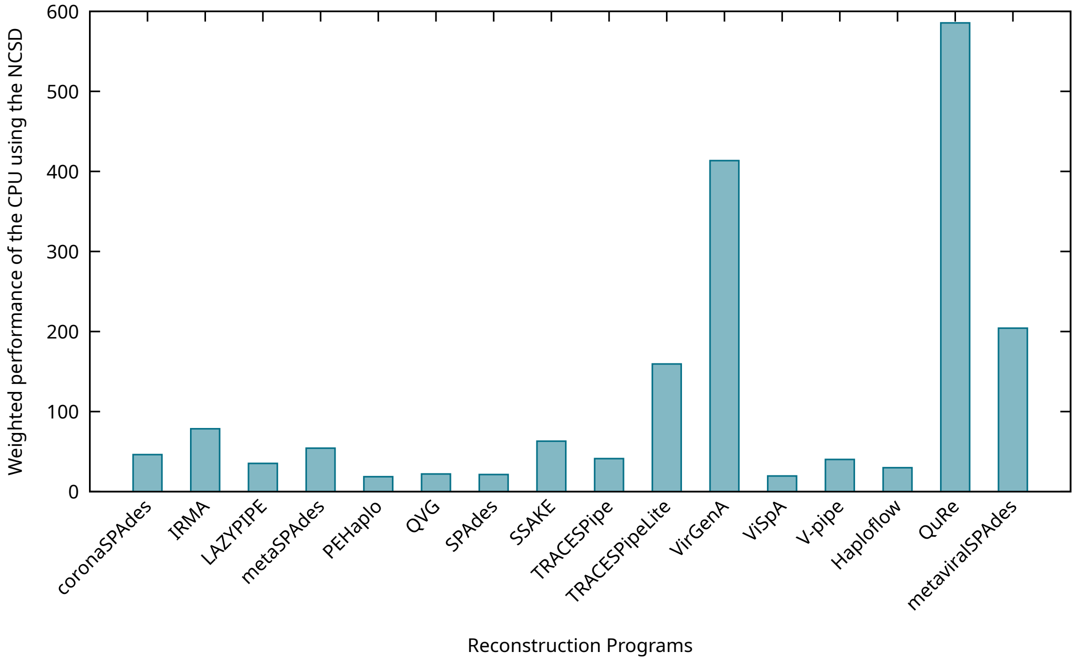

Figure S133: Figure comparing the average performance of the reconstruction programs in terms of the weighted percentage of CPU used across all datasets reconstructed.

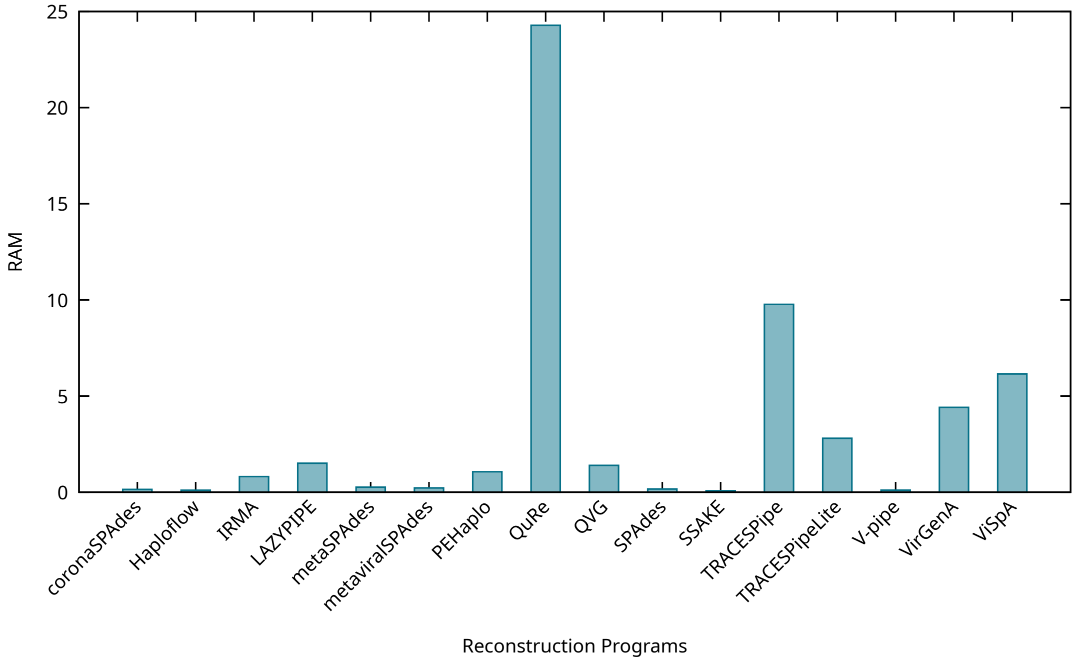

Figure S134: Figure comparing the average performance of the reconstruction programs in terms of the RAM used across all datasets reconstructed.

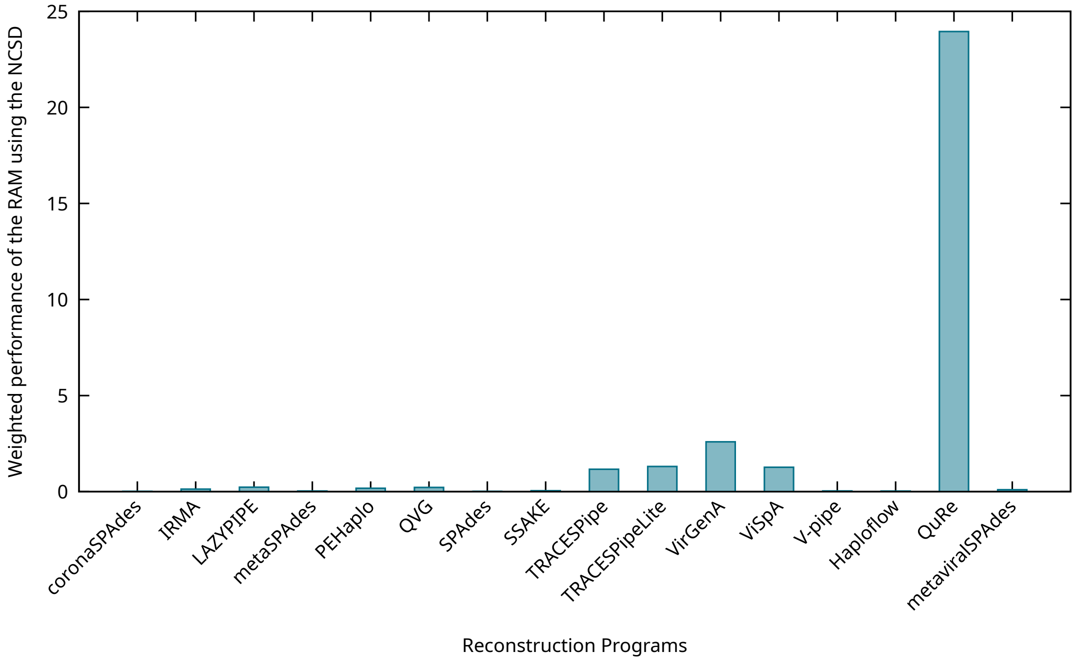

Figure S135: Figure comparing the average performance of the reconstruction programs in terms of the weighted RAM used across all datasets reconstructed.

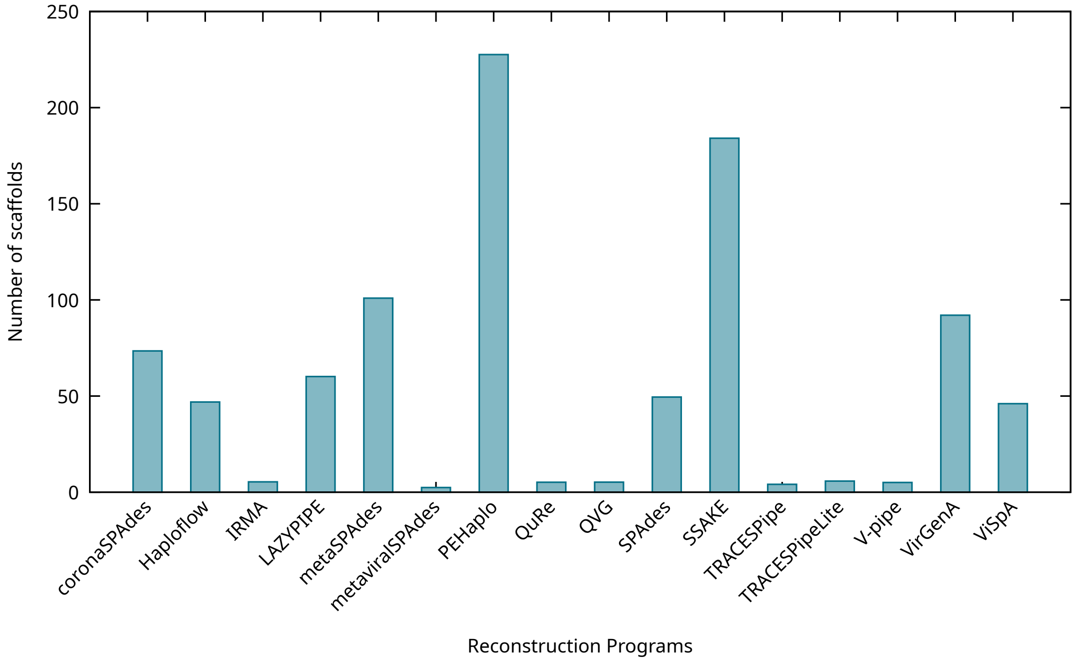

Figure S136: Figure comparing the average performance of the reconstruction programs in terms of the number of scaffolds reconstructed across all datasets reconstructed.

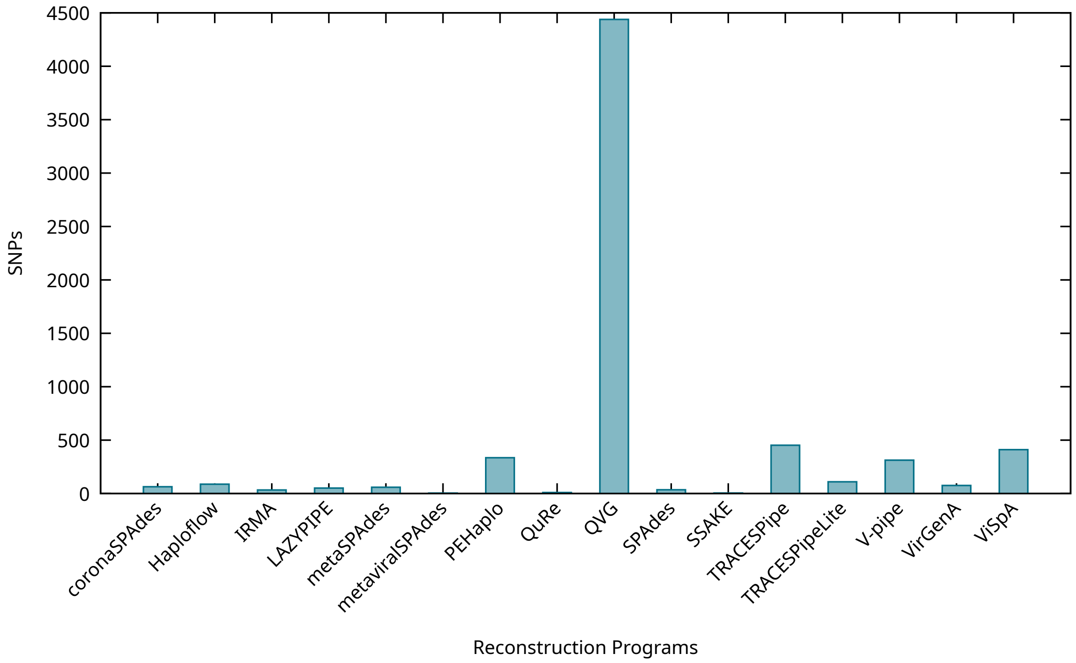

Figure S137: Figure comparing the average performance of the reconstruction programs in terms of the number of SNPs across all datasets reconstructed.

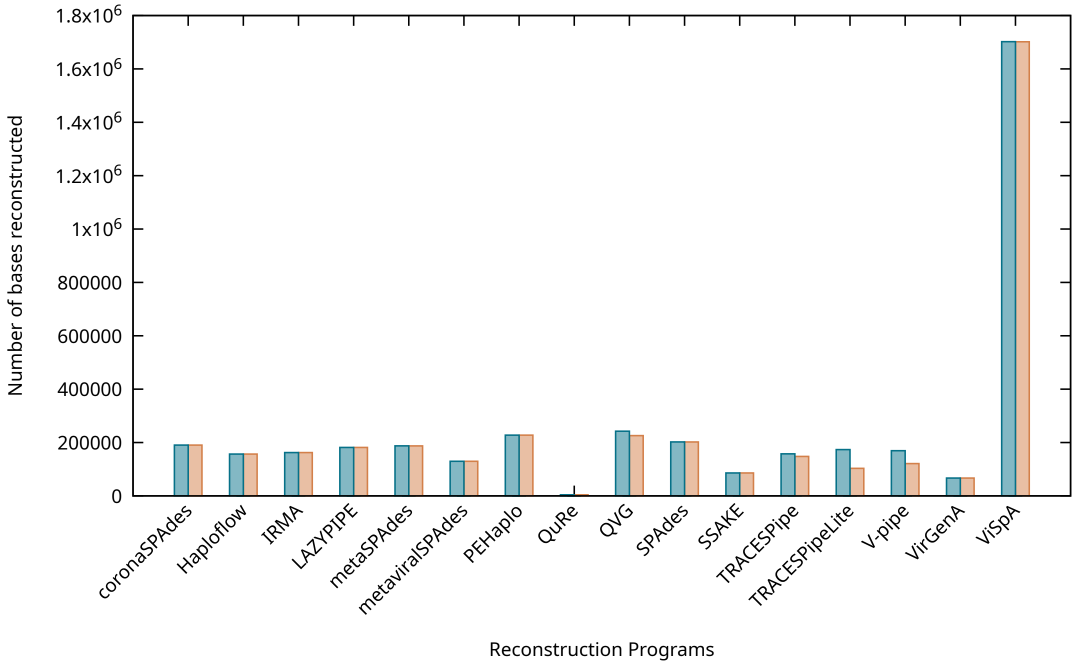

Figure S138: Figure comparing the average performance of the reconstruction programs in terms of the overall number of bases reconstructed across all datasets reconstructed. The blue bars show the performance using all bases present in the reconstructed file, whereas the orange bars show the results excluding non-reconstructed bases (N).

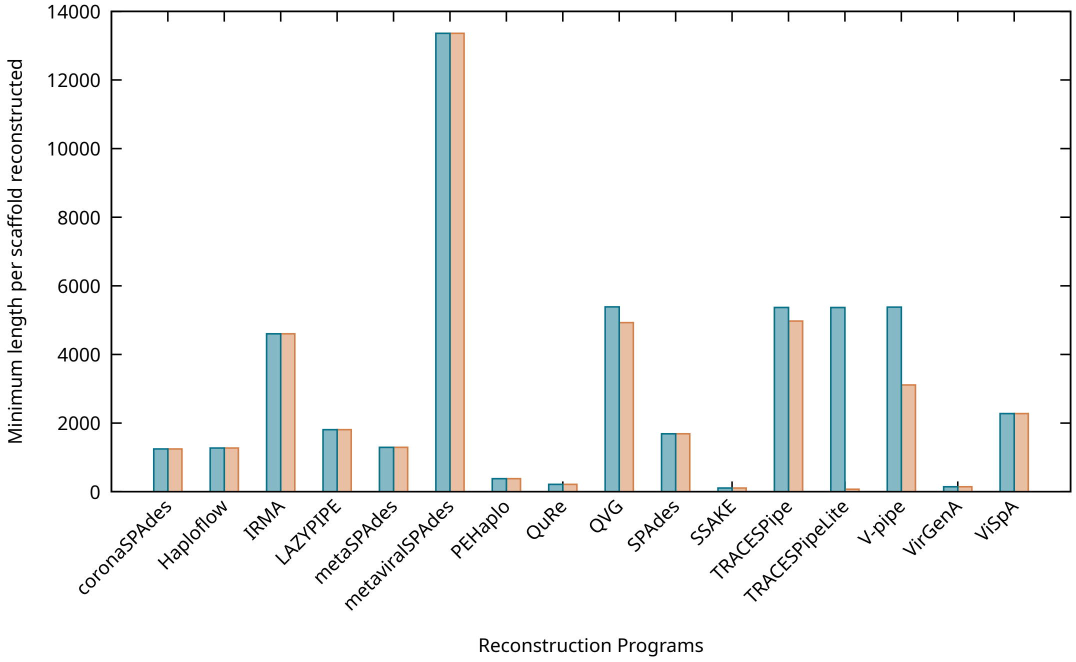

Figure S139: Figure comparing the average performance of the reconstruction programs in terms of the minimum number of bases reconstructed per scaffold across all datasets reconstructed. The blue bars show the performance using all bases present in the reconstructed file, whereas the orange bars show the results excluding non-reconstructed bases (N).

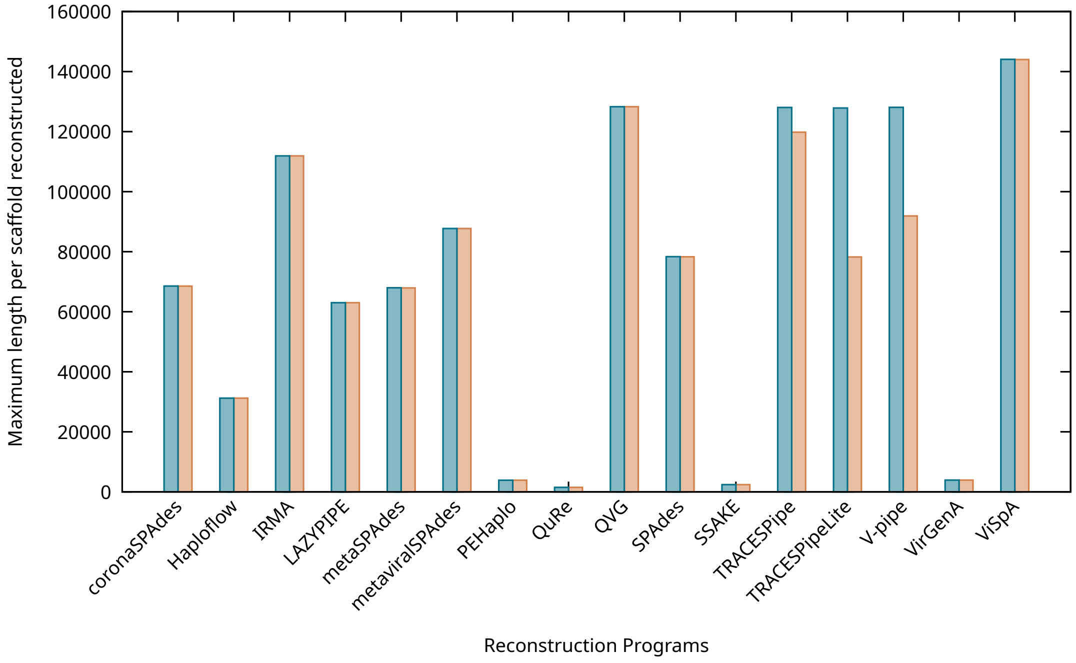

Figure S140: Figure comparing the average performance of the reconstruction programs in terms of the maximum number of bases reconstructed by scaffold across all datasets reconstructed. The blue bars show the performance using all bases present in the reconstructed file, whereas the orange bars show the results excluding non-reconstructed bases (N).

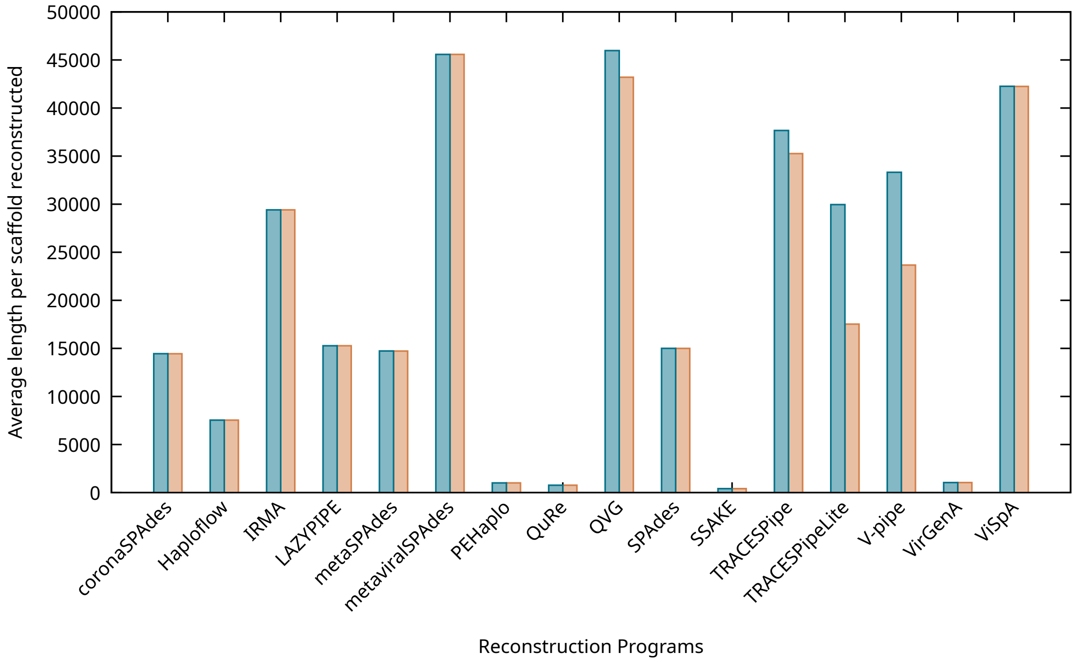

Figure S141: Figure comparing the average performance of the reconstruction programs in terms of the average number of bases reconstructed by scaffold across all datasets reconstructed. The blue bars show the performance using all bases present in the reconstructed file, whereas the orange bars show the results excluding non-reconstructed bases (N).

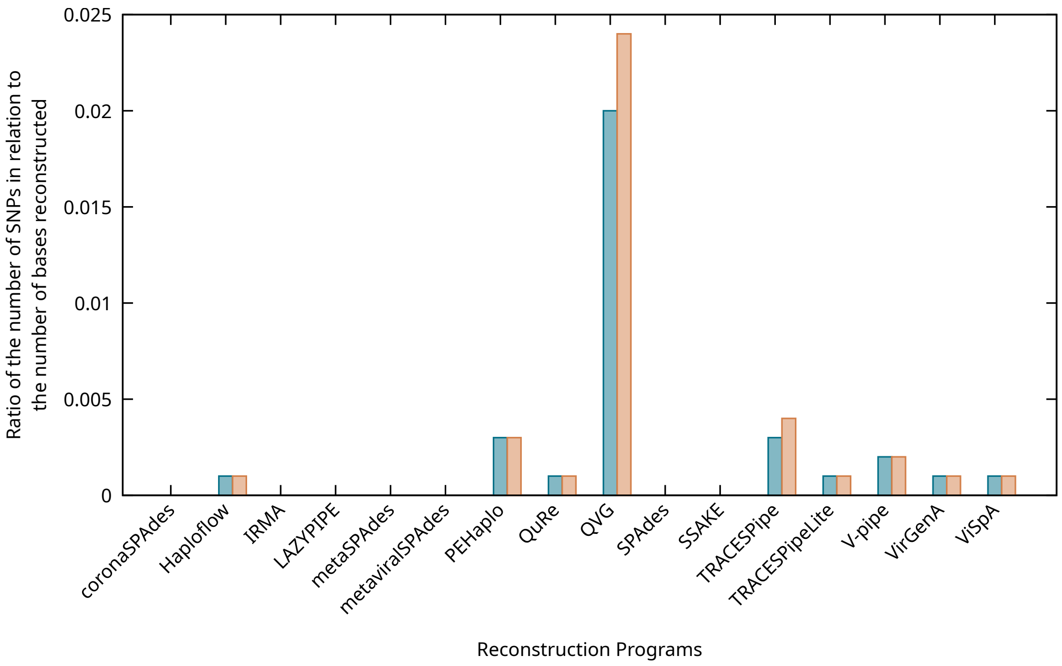

Figure S142: Figure comparing the average performance of the reconstruction programs in terms of the number of SNPs in relation to the number of bases reconstructed across all datasets reconstructed. The blue bars show the performance using all bases present in the reconstructed file, whereas the orange bars show the results excluding non-reconstructed bases (N).

Table S5: Results obtained for DS1 using the benchmark proposed. The execution time was measured in seconds, the RAM usage was measured in GB and the CPU usage is presented as a percentage. The executions were, when possible, capped at 6 threads and 48 GB of RAM.

Reconstruction tool	Execution time	SNPs	Identity	NCSD	NRC	RAM usage	CPU usage	Number of scaffolds	Reconstructed bases	Minimum scaffold length	Maximum scaffold length	Average scaffold length
coronaSPAdes	1.12	131.0	99.824	0.462	0.42	0.133	313.667	182.0	83087.0	100.0	1489.0	456.5
Haploflow	–	–	–	–	–	–	–	–	–	–	–	–
IRMA	35.28	93.667	96.09	0.38	0.383	0.128	317.0	4.0	95730.0	3682.0	82389.0	23932.5
LAZYPIPE	5.455	64.0	99.881	0.579	0.537	1.494	108.667	152.0	65307.0	215.0	1352.0	429.7
metaSPAdes	1.865	128.0	99.854	0.423	0.382	0.225	295.333	210.0	88863.0	71.0	1489.0	423.2
metaviralSPAdes	–	–	–	–	–	–	–	–	–	–	–	–
PEHaplo	11.15	793.0	99.749	0.606	0.568	0.091	46.0	104.0	61220.0	375.0	1961.0	588.7
QuRe	–	–	–	–	–	–	–	–	–	–	–	–
QVG	18.815	1301.0	99.064	0.115	0.092	1.387	82.333	3.0	138473.0	5596.0	125041.0	46157.7
SPAdes	2.315	104.0	99.858	0.466	0.425	0.141	240.667	179.0	82068.0	104.0	1489.0	458.5
SSAKE	2.445	0.0	100.0	1.0	0.994	0.041	84.0	2.0	241.0	113.0	128.0	120.5
TRACESPipe	180.42	100.0	97.349	0.326	0.292	9.768	319.333	4.0	143860.0	5387.0	125041.0	35965.0
TRACESPipeLite	35.58	66.0	97.028	0.323	0.288	2.707	364.0	5.0	151693.0	5387.0	125041.0	30338.6
VirGenA	200.22	88.0	99.884	0.465	0.43	3.857	567.667	238.0	86031.0	53.0	1293.0	361.5
ViSpA	43.835	94.0	95.123	0.523	0.502	4.913	51.0	75.0	2292352.0	0.0	70025.0	30564.7
V-pipe	32.89	0.0	95.791	0.976	0.963	0.104	106.667	5.0	160429.0	5387.0	125041.0	32085.8

Table S6: Results obtained for DS2 using the benchmark proposed. The execution time was measured in seconds, the RAM usage was measured in GB and the CPU usage is presented as a percentage. The executions were, when possible, capped at 6 threads and 48 GB of RAM.

Reconstruction tool	Execution time	SNPs	Identity	NCSD	NRC	RAM usage	CPU usage	Number of scaffolds	Reconstructed bases	Minimum scaffold length	Maximum scaffold length	Average scaffold length
coronaSPAdes	1.37	120.0	99.775	0.115	0.091	0.132	370.667	100.0	129540.0	227.0	5926.0	1295.4
Haploflow	–	–	–	–	–	–	–	–	–	–	–	–
IRMA	76.93	48.333	98.395	0.161	0.182	0.273	371.667	4.0	122405.0	4751.0	104886.0	30601.3
LAZYPIPE	4.255	114.0	99.896	0.17	0.142	1.499	184.667	92.0	121351.0	305.0	10147.0	1319.0
metaSPAdes	2.335	156.0	99.721	0.109	0.085	0.225	341.0	104.0	130243.0	97.0	7357.0	1252.3
metaviralSPAdes	–	–	–	–	–	–	–	–	–	–	–	–
PEHaplo	10.64	114.0	99.877	0.137	0.112	0.114	68.667	102.0	127512.0	376.0	4405.0	1250.1
QuRe	–	–	–	–	–	–	–	–	–	–	–	–
QVG	15.445	955.0	99.333	0.067	0.047	1.393	100.0	4.0	143860.0	5387.0	125041.0	35965.0
SPAdes	3.345	109.0	99.909	0.113	0.089	0.148	317.667	101.0	129841.0	119.0	10193.0	1285.6
SSAKE	4.09	2.333	99.972	0.961	0.945	0.054	100.0	51.0	7772.333	101.333	250.667	152.2
TRACESPipe	177.915	135.0	99.06	0.084	0.066	9.768	341.0	4.0	143860.0	5387.0	125041.0	35965.0
TRACESPipeLite	34.735	153.0	98.787	0.097	0.077	2.749	370.0	5.0	151693.0	5387.0	125041.0	30338.6
VirGenA	309.26	94.0	99.878	0.289	0.263	3.6	643.667	111.333	110418.667	98.333	3544.667	993.667
ViSpA	69.81	137.0	97.596	0.156	0.138	5.02	63.333	80.0	3612120.0	0.0	112477.0	45151.5
V-pipe	32.22	24.0	94.134	0.649	0.612	0.104	115.333	5.0	160429.0	5387.0	125041.0	32085.8

Table S7: Results obtained for DS3 using the benchmark proposed. The execution time was measured in seconds, the RAM usage was measured in GB and the CPU usage is presented as a percentage. The executions were, when possible, capped at 6 threads and 48 GB of RAM.

Reconstruction tool	Execution time	SNPs	Identity	NCSD	NRC	RAM usage	CPU usage	Number of scaffolds	Reconstructed bases	Minimum scaffold length	Maximum scaffold length	Average scaffold length
coronaSPAdes	1.74	39.0	99.868	0.04	0.029	0.138	388.667	21.0	138037.0	81.0	29589.0	6573.2
Haploflow	1.41	34.0	99.882	0.669	0.635	0.05	99.667	65.0	52460.0	508.0	3184.0	807.1
IRMA	87.815	5.0	99.921	0.1	0.132	0.483	426.667	4.0	127894.0	4769.0	110094.0	31973.5
LAZYPIPE	4.905	60.0	99.95	0.048	0.036	1.505	220.0	19.0	135584.0	1052.0	18805.0	7136.0
metaSPAdes	3.02	60.0	99.813	0.042	0.031	0.226	367.333	17.0	136629.0	135.0	29589.0	8037.0
metaviralSPAdes	–	–	–	–	–	–	–	–	–	–	–	–
PEHaplo	14.385	55.0	99.931	0.064	0.049	0.322	96.0	116.0	146720.0	393.0	5751.0	1264.8
QuRe	129.93	0.0	100.0	1.0	0.994	12.4	622.333	3.0	287.0	95.0	96.0	95.7
QVG	17.295	452.0	99.685	0.051	0.033	1.397	114.667	4.0	143860.0	5387.0	125041.0	35965.0
SPAdes	11.17	37.0	99.926	0.04	0.029	0.151	194.333	27.0	143682.0	167.0	29589.0	5321.6
SSAKE	1.46	3.333	99.984	0.885	0.859	0.043	100.0	116.0	20861.333	100.333	418.0	179.767
TRACESPipe	179.45	152.0	99.85	0.038	0.028	9.768	341.333	4.0	143860.0	5387.0	125041.0	35965.0
TRACESPipeLite	36.65	132.333	99.857	0.036	0.026	2.759	357.333	5.0	151693.0	5387.0	125041.0	30338.6
VirGenA	448.105	66.0	99.864	0.252	0.23	3.879	696.0	64.0	114483.0	49.0	6344.0	1788.8
ViSpA	44.995	132.0	99.514	0.046	0.037	5.215	73.333	9.0	483660.0	0.0	124259.0	53740.0
V-pipe	34.765	46.0	97.085	0.251	0.22	0.104	116.667	5.0	160429.0	5387.0	125041.0	32085.8

Table S8: Results obtained for DS4 using the benchmark proposed. The execution time was measured in seconds, the RAM usage was measured in GB and the CPU usage is presented as a percentage. The executions were, when possible, capped at 6 threads and 48 GB of RAM.

**Reconstruction tool**	**Execution time**	**SNPs**	**Identity**	**NCSD**	**NRC**	**RAM usage**	**CPU usage**	**Number of scaffolds**	**Reconstructed bases**	**Minimum scaffold length**	**Maximum scaffold length**	**Average scaffold length**
coronaSPAdes	2.23	74.0	99.837	0.036	0.026	0.139	390.0	14.0	138101.0	81.0	82423.0	9864.4
Haploflow	1.9	68.0	99.908	0.173	0.147	0.059	100.0	82.0	126008.0	510.0	5576.0	1536.7
IRMA	121.98	1.0	99.992	0.096	0.128	0.683	470.333	4.0	128279.0	4882.0	110245.0	32069.8
LAZYPIPE	4.965	72.0	99.889	0.038	0.027	1.508	225.0	8.0	136217.0	5330.0	82405.0	17027.1
metaSPAdes	3.745	73.0	99.899	0.038	0.027	0.226	388.0	8.0	136413.0	71.0	77567.0	17051.6
metaviralSPAdes	4.145	1.0	99.94	0.97	0.961	0.226	422.333	1.0	5358.0	5358.0	5358.0	5358.0
PEHaplo	19.74	86.0	99.935	0.068	0.053	0.558	123.0	180.0	165861.0	379.0	3898.0	921.5
QuRe	225.47	0.0	100.0	0.999	0.992	14.053	561.0	6.333	658.333	86.0	136.0	104.067
QVG	20.035	197.0	99.859	0.035	0.026	1.399	127.667	5.0	268901.0	5387.0	125041.0	53780.2
SPAdes	7.145	68.0	99.791	0.037	0.026	0.154	269.667	7.0	137370.0	131.0	90831.0	19624.3
SSAKE	2.375	5.667	99.986	0.755	0.724	0.051	100.0	183.333	41342.0	102.667	587.0	225.3
TRACESPipe	185.445	111.0	99.92	0.034	0.024	9.768	336.667	4.0	143860.0	5387.0	125041.0	35965.0
TRACESPipeLite	38.555	124.667	99.913	0.033	0.023	2.763	350.333	5.0	151693.0	5387.0	125041.0	30338.6
VirGenA	506.295	98.0	99.831	0.25	0.224	3.962	734.333	50.0	114803.0	132.0	8622.0	2296.1
ViSpA	86.255	162.0	99.782	0.036	0.026	4.907	85.667	5.0	143528.0	0.0	124918.0	28705.6
V-pipe	38.185	151.0	99.49	0.057	0.044	0.104	115.667	5.0	160429.0	5387.0	125041.0	32085.8

Table S9: Results obtained for DS5 using the benchmark proposed. The execution time was measured in seconds, the RAM usage was measured in GB and the CPU usage is presented as a percentage. The executions were, when possible, capped at 6 threads and 48 GB of RAM.

**Reconstruction tool**	**Execution time**	**SNPs**	**Identity**	**NCSD**	**NRC**	**RAM usage**	**CPU usage**	**Number of scaffolds**	**Reconstructed bases**	**Minimum scaffold length**	**Maximum scaffold length**	**Average scaffold length**
coronaSPAdes	2.445	36.0	99.884	0.036	0.025	0.147	402.667	9.0	137420.0	81.0	112095.0	15268.9
Haploflow	2.39	60.0	99.949	0.055	0.046	0.07	99.333	42.0	143037.0	517.0	13976.0	3405.6
IRMA	154.425	0.0	100.0	0.095	0.127	0.876	503.0	4.0	128314.0	4872.0	110237.0	32078.5
LAZYPIPE	5.73	53.0	99.947	0.036	0.026	1.512	245.333	10.0	137370.0	468.0	88573.0	13737.0
metaSPAdes	4.265	37.0	99.824	0.037	0.027	0.226	395.667	7.0	136333.0	71.0	112370.0	19476.1
metaviralSPAdes	–	–	–	–	–	–	–	–	–	–	–	–
PEHaplo	25.725	80.0	99.919	0.08	0.065	0.828	129.0	204.0	167438.0	375.0	2820.0	820.8
QuRe	284.305	4.667	99.652	0.996	0.985	13.86	554.667	6.333	1569.333	123.0	347.0	247.4
QVG	21.065	157.0	99.891	0.032	0.024	1.401	138.333	5.0	268901.0	5387.0	125041.0	53780.2
SPAdes	6.355	32.0	99.931	0.035	0.025	0.153	320.0	19.0	145107.0	167.0	108192.0	7637.2
SSAKE	4.295	6.667	99.987	0.432	0.396	0.065	100.0	313.333	93668.0	100.667	1211.0	299.1
TRACESPipe	188.335	153.0	99.893	0.034	0.024	9.768	338.667	4.0	143860.0	5387.0	125041.0	35965.0
TRACESPipeLite	40.195	147.0	99.894	0.033	0.023	2.766	342.0	5.0	151693.0	5387.0	125041.0	30338.6
VirGenA	611.85	67.0	99.866	0.246	0.224	3.914	709.0	40.0	115017.0	52.0	15798.0	2875.4
ViSpA	149.83	158.0	99.884	0.033	0.024	5.002	90.667	5.0	143678.0	0.0	124984.0	28735.6
V-pipe	40.725	64.0	99.627	0.047	0.037	0.104	115.667	5.0	160429.0	5387.0	125041.0	32085.8

Table S10: Results obtained for DS6 using the benchmark proposed. The execution time was measured in seconds, the RAM usage was measured in GB and the CPU usage is presented as a percentage. The executions were, when possible, capped at 6 threads and 48 GB of RAM.

**Reconstruction tool**	**Execution time**	**SNPs**	**Identity**	**NCSD**	**NRC**	**RAM usage**	**CPU usage**	**Number of scaffolds**	**Reconstructed bases**	**Minimum scaffold length**	**Maximum scaffold length**	**Average scaffold length**
coronaSPAdes	2.735	58.0	99.912	0.036	0.026	0.148	410.333	10.0	137441.0	81.0	105120.0	13744.1
Haploflow	2.985	98.0	99.923	0.035	0.027	0.08	100.0	21.0	165970.0	934.0	31458.0	7903.3
IRMA	188.2	0.0	100.0	0.096	0.128	1.071	533.667	4.0	128304.0	4888.0	110240.0	32076.0
LAZYPIPE	6.575	60.667	99.946	0.037	0.027	1.521	238.333	8.0	136723.0	390.0	85843.0	17090.4
metaSPAdes	4.91	68.0	99.904	0.037	0.026	0.227	403.333	9.0	136939.0	71.0	105136.0	15215.4
metaviralSPAdes	5.37	1.0	99.94	0.97	0.961	0.227	433.333	1.0	5358.0	5358.0	5358.0	5358.0
PEHaplo	31.53	41.0	99.962	0.071	0.058	1.159	136.333	203.0	177772.0	375.0	3301.0	875.7
QuRe	453.73	8.333	99.483	0.991	0.976	14.141	594.333	2.667	2514.333	345.333	1321.0	833.167
QVG	25.5	149.0	99.893	0.031	0.024	1.407	141.667	5.0	268901.0	5387.0	125041.0	53780.2
SPAdes	5.505	16.0	99.944	0.034	0.023	0.162	384.667	25.0	146735.0	167.0	105533.0	5869.4
SSAKE	6.125	9.667	99.988	0.177	0.151	0.075	100.0	363.667	136568.0	101.0	2305.0	375.533
TRACESPipe	199.76	148.0	99.894	0.032	0.022	9.768	333.667	4.0	143860.0	5387.0	125041.0	35965.0
TRACESPipeLite	42.275	167.667	99.882	0.033	0.023	2.768	335.0	5.0	151693.0	5387.0	125041.0	30338.6
VirGenA	685.01	63.0	99.851	0.24	0.218	4.125	730.667	40.0	116189.0	70.0	11738.0	2904.7
ViSpA	219.8	156.0	99.891	0.034	0.025	5.223	97.333	5.0	143613.0	0.0	124985.0	28722.6
V-pipe	44.63	148.0	99.886	0.036	0.026	0.104	114.333	5.0	160429.0	5387.0	125041.0	32085.8

Table S11: Results obtained for DS7 using the benchmark proposed. The execution time was measured in seconds, the RAM usage was measured in GB and the CPU usage is presented as a percentage. The executions were, when possible, capped at 6 threads and 48 GB of RAM.

Reconstruction tool	Execution time	SNPs	Identity	NCSD	NRC	RAM usage	CPU usage	Number of scaffolds	Reconstructed bases	Minimum scaffold length	Maximum scaffold length	Average scaffold length
coronaSPAdes	2.96	60.0	99.864	0.036	0.025	0.149	417.333	11.0	137558.0	81.0	105120.0	12505.3
Haploflow	3.445	42.0	99.862	0.035	0.025	0.089	99.333	17.0	147954.0	1881.0	33983.0	8703.2
IRMA	220.62	0.0	100.0	0.095	0.127	1.268	553.0	4.0	128337.0	4883.0	110250.0	32084.3
LAZYPIPE	6.5	96.333	99.74	0.036	0.025	1.525	235.667	5.0	136271.0	5330.0	109786.0	27254.2
metaSPAdes	5.45	52.0	99.869	0.037	0.026	0.228	411.333	10.0	136939.0	71.0	105136.0	13693.9
metaviralSPAdes	–	–	–	–	–	–	–	–	–	–	–	–
PEHaplo	38.6	55.0	99.957	0.047	0.036	1.461	146.0	208.0	192617.0	375.0	3338.0	926.0
QuRe	6581.505	0.333	99.869	0.991	0.976	14.994	619.333	3.667	2340.333	308.333	1131.333	693.767
QVG	25.245	149.0	99.893	0.034	0.024	1.409	145.667	4.0	143860.0	5387.0	125041.0	35965.0
SPAdes	6.555	20.0	99.941	0.034	0.024	0.162	374.0	25.0	146717.0	167.0	105128.0	5868.7
SSAKE	10.24	15.667	99.983	0.078	0.061	0.087	100.0	294.333	151735.667	101.0	3245.0	515.867
TRACESPipe	198.46	162.0	99.885	0.032	0.022	9.768	330.0	4.0	143860.0	5387.0	125041.0	35965.0
TRACESPipeLite	43.595	149.333	99.893	0.033	0.023	2.771	328.667	5.0	151693.0	5387.0	125041.0	30338.6
VirGenA	735.6	88.0	99.89	0.227	0.206	4.215	742.0	20.333	117296.0	771.667	37979.667	6486.867
ViSpA	282.775	165.0	99.84	0.034	0.025	5.546	106.0	5.0	143678.0	0.0	124993.0	28735.6
V-pipe	46.825	147.0	99.886	0.035	0.025	0.104	114.667	5.0	160429.0	5387.0	125041.0	32085.8

Table S12: Results obtained for DS8 using the benchmark proposed. The execution time was measured in seconds, the RAM usage was measured in GB and the CPU usage is presented as a percentage. The executions were, when possible, capped at 6 threads and 48 GB of RAM.

Reconstruction tool	Execution time	SNPs	Identity	NCSD	NRC	RAM usage	CPU usage	Number of scaffolds	Reconstructed bases	Minimum scaffold length	Maximum scaffold length	Average scaffold length
coronaSPAdes	3.465	46.0	99.876	0.036	0.026	0.15	430.667	10.0	137518.0	81.0	105120.0	13751.8
Haploflow	4.445	119.0	99.87	0.034	0.024	0.106	99.667	12.0	147444.0	661.0	88978.0	12287.0
IRMA	285.545	0.0	100.0	0.095	0.127	1.65	582.667	4.0	128349.0	4887.0	110254.0	32087.3
LAZYPIPE	7.815	52.0	99.879	0.035	0.025	1.531	257.0	9.0	137996.0	425.0	85904.0	15332.9
metaSPAdes	6.63	21.0	99.829	0.037	0.026	0.229	421.0	10.0	136997.0	71.0	105136.0	13699.7
metaviralSPAdes	–	–	–	–	–	–	–	–	–	–	–	–
PEHaplo	61.135	51.0	99.951	0.04	0.03	2.199	143.333	186.0	208292.0	379.0	4211.0	1119.8
QuRe	1778.55	44.0	99.536	0.94	0.902	15.574	615.333	4.333	16232.0	613.0	4914.0	3528.0
QVG	29.54	147.0	99.894	0.031	0.024	1.412	157.667	5.0	268901.0	5387.0	125041.0	53780.2
SPAdes	7.41	22.0	99.94	0.034	0.024	0.165	394.0	25.0	146751.0	131.0	105954.0	5870.0
SSAKE	11.49	7.333	99.99	0.038	0.028	0.098	100.0	144.333	154382.0	113.0	10289.333	1074.2
TRACESPipe	203.685	126.0	99.912	0.032	0.022	9.768	335.333	4.0	143860.0	5387.0	125041.0	35965.0
TRACESPipeLite	46.81	135.667	99.906	0.032	0.022	2.778	316.333	5.0	151693.0	5387.0	125041.0	30338.6
VirGenA	834.305	96.0	99.869	0.234	0.211	4.144	733.0	41.0	116748.0	368.0	11086.0	2847.5
ViSpA	472.815	158.0	99.884	0.034	0.024	5.217	113.333	5.0	143720.0	0.0	124998.0	28744.0
V-pipe	52.95	160.0	99.887	0.035	0.025	0.104	114.333	5.0	160429.0	5387.0	125041.0	32085.8

Table S13: Results obtained for DS9 using the benchmark proposed. The execution time was measured in seconds, the RAM usage was measured in GB and the CPU usage is presented as a percentage. The executions were, when possible, capped at 6 threads and 48 GB of RAM.

Reconstruction tool	Execution time	SNPs	Identity	NCSD	NRC	RAM usage	CPU usage	Number of scaffolds	Reconstructed bases	Minimum scaffold length	Maximum scaffold length	Average scaffold length
coronaSPAdes	1.26	117.0	99.856	0.47	0.428	0.134	357.333	267.0	120540.0	100.0	1650.0	451.5
Haploflow	–	–	–	–	–	–	–	–	–	–	–	–
IRMA	37.405	113.667	96.415	0.399	0.402	0.124	332.0	5.0	105134.0	3507.0	79651.0	21026.8
LAZYPIPE	4.345	61.0	99.89	0.645	0.607	1.496	184.333	162.0	79968.0	301.0	1571.0	493.6
metaSPAdes	2.075	114.0	99.866	0.439	0.401	0.225	326.667	305.0	127880.0	71.0	1650.0	419.3
metaviralSPAdes	–	–	–	–	–	–	–	–	–	–	–	–
PEHaplo	9.67	1616.0	99.819	0.641	0.598	0.091	62.667	135.0	82627.0	378.0	2690.0	612.1
QuRe	–	–	–	–	–	–	–	–	–	–	–	–
QVG	15.65	1300.0	99.059	0.115	0.094	1.389	100.667	3.0	138264.0	5387.0	125041.0	46088.0
SPAdes	2.15	94.0	99.88	0.481	0.443	0.148	368.0	258.0	118081.0	104.0	1650.0	457.7
SSAKE	2.74	0.0	66.667	0.999	0.994	0.049	100.0	3.667	538.333	116.333	177.0	145.767
TRACESPipe	159.42	127.0	96.979	0.334	0.298	9.768	354.333	4.0	143860.0	5387.0	125041.0	35965.0
TRACESPipeLite	35.185	62.0	97.105	0.34	0.303	2.777	367.0	6.0	168289.0	5387.0	125041.0	28048.2
VirGenA	252.57	82.0	99.846	0.589	0.554	3.654	599.667	199.333	66905.0	48.333	1010.0	335.633
ViSpA	43.605	91.667	94.393	0.516	0.492	5.573	58.333	85.0	2544032.0	796.0	70014.0	29929.8
V-pipe	30.595	0.0	98.428	0.971	0.957	0.104	115.0	5.0	160429.0	5387.0	125041.0	32085.8

Table S14: Results obtained for DS10 using the benchmark proposed. The execution time was measured in seconds, the RAM usage was measured in GB and the CPU usage is presented as a percentage. The executions were, when possible, capped at 6 threads and 48 GB of RAM.

**Reconstruction tool**	**Execution time**	**SNPs**	**Identity**	**NCSD**	**NRC**	**RAM usage**	**CPU usage**	**Number of scaffolds**	**Reconstructed bases**	**Minimum scaffold length**	**Maximum scaffold length**	**Average scaffold length**
coronaSPAdes	1.65	132.0	99.856	0.108	0.085	0.139	397.333	174.0	194352.0	239.0	5075.0	1117.0
Haploflow	–	–	–	–	–	–	–	–	–	–	–	–
IRMA	65.315	45.0	98.672	0.155	0.178	0.275	396.0	5.0	139050.333	4800.0	105942.0	27810.067
LAZYPIPE	4.82	109.0	99.867	0.191	0.162	1.502	211.667	145.0	175180.0	304.0	8726.0	1208.1
metaSPAdes	68.465	147.333	99.871	0.19	0.162	0.227	589.0	275.0	179576.0	137.0	3643.333	653.6
metaviralSPAdes	–	–	–	–	–	–	–	–	–	–	–	–
PEHaplo	11.785	88.0	99.917	0.173	0.145	0.171	82.0	156.0	184050.0	377.0	3812.0	1179.8
QuRe	–	–	–	–	–	–	–	–	–	–	–	–
QVG	17.165	989.0	99.317	0.068	0.048	1.394	112.0	4.0	143860.0	5387.0	125041.0	35965.0
SPAdes	4.205	118.0	99.716	0.112	0.091	0.151	320.0	167.0	192461.0	172.0	8313.0	1152.5
SSAKE	6.03	1.0	99.988	0.958	0.94	0.068	100.0	74.667	11568.667	100.667	249.333	154.867
TRACESPipe	174.135	177.0	98.896	0.096	0.077	9.768	350.333	4.0	143860.0	5387.0	125041.0	35965.0
TRACESPipeLite	37.15	149.667	98.858	0.091	0.073	2.81	356.333	6.0	168262.0	5387.0	125041.0	28043.7
VirGenA	378.31	65.0	99.83	0.535	0.506	4.446	681.0	124.0	73908.0	50.0	2356.0	596.0
ViSpA	82.4	125.0	96.964	0.157	0.139	6.633	75.667	92.0	4473795.0	661.0	113638.0	48628.2
V-pipe	34.08	22.0	94.328	0.643	0.606	0.104	115.0	5.0	160429.0	5387.0	125041.0	32085.8

Table S15: Results obtained for DS11 using the benchmark proposed. The execution time was measured in seconds, the RAM usage was measured in GB and the CPU usage is presented as a percentage. The executions were, when possible, capped at 6 threads and 48 GB of RAM.

**Reconstruction tool**	**Execution time**	**SNPs**	**Identity**	**NCSD**	**NRC**	**RAM usage**	**CPU usage**	**Number of scaffolds**	**Reconstructed bases**	**Minimum scaffold length**	**Maximum scaffold length**	**Average scaffold length**
coronaSPAdes	2.205	83.0	99.825	0.04	0.029	0.141	411.667	24.0	203993.0	81.0	33349.0	8499.7
Haploflow	2.06	38.0	99.933	0.616	0.577	0.07	99.333	107.0	86028.0	501.0	2339.0	804.0
IRMA	94.69	3.333	99.975	0.099	0.131	0.482	460.0	5.0	144593.0	4813.0	110179.0	28918.6
LAZYPIPE	5.515	89.0	99.911	0.044	0.033	1.509	248.0	20.0	202280.0	311.0	28959.0	10114.0
metaSPAdes	3.82	47.0	99.853	0.041	0.029	0.227	398.667	25.0	203651.0	71.0	33395.0	8146.0
metaviralSPAdes	–	–	–	–	–	–	–	–	–	–	–	–
PEHaplo	17.03	58.0	99.952	0.093	0.076	0.427	103.667	202.0	222692.0	380.0	4629.0	1102.4
QuRe	–	–	–	–	–	–	–	–	–	–	–	–
QVG	19.385	408.0	99.715	0.049	0.032	1.399	126.667	4.0	143860.0	5387.0	125041.0	35965.0
SPAdes	12.11	27.0	99.862	0.04	0.029	0.158	210.0	26.0	204678.0	97.0	33317.0	7872.2
SSAKE	1.67	1.667	99.956	0.92	0.9	0.048	100.0	109.333	19881.333	100.667	472.0	181.767
TRACESPipe	171.255	135.0	99.861	0.036	0.026	9.768	355.0	4.0	143860.0	5387.0	125041.0	35965.0
TRACESPipeLite	39.35	151.0	99.861	0.035	0.026	2.816	344.667	6.0	168262.0	5387.0	125041.0	28043.7
VirGenA	576.06	46.0	99.872	0.6	0.573	4.565	730.0	82.667	63628.0	138.333	2743.0	808.1
ViSpA	41.54	3.0	99.732	0.043	0.033	5.923	91.0	8.0	329578.0	5254.0	124613.0	41197.3
V-pipe	37.86	54.0	96.844	0.245	0.213	0.104	114.667	5.0	160429.0	5387.0	125041.0	32085.8

Table S16: Results obtained for DS12 using the benchmark proposed. The execution time was measured in seconds, the RAM usage was measured in GB and the CPU usage is presented as a percentage. The executions were, when possible, capped at 6 threads and 48 GB of RAM.

**Reconstruction tool**	**Execution time**	**SNPs**	**Identity**	**NCSD**	**NRC**	**RAM usage**	**CPU usage**	**Number of scaffolds**	**Reconstructed bases**	**Minimum scaffold length**	**Maximum scaffold length**	**Average scaffold length**
coronaSPAdes	2.67	53.0	99.867	0.037	0.026	0.148	415.333	12.0	203872.0	81.0	67883.0	16989.3
Haploflow	2.89	75.0	99.932	0.205	0.179	0.086	99.0	118.0	180100.0	506.0	9199.0	1526.3
IRMA	130.94	0.0	99.984	0.096	0.128	0.678	505.333	5.0	144771.0	4882.0	110219.0	28954.2
LAZYPIPE	6.225	74.0	99.938	0.039	0.028	1.518	261.0	10.0	202548.0	364.0	71673.0	20254.8
metaSPAdes	4.78	55.0	99.823	0.038	0.027	0.229	408.667	13.0	203274.0	71.0	67865.0	15636.5
metaviralSPAdes	–	–	–	–	–	–	–	–	–	–	–	–
PEHaplo	23.755	38.0	99.969	0.078	0.062	0.762	119.667	276.0	236245.0	376.0	3084.0	856.0
QuRe	867.745	0.0	100.0	0.999	0.992	43.794	557.333	6.333	551.667	53.667	117.333	88.933
QVG	22.37	225.0	99.843	0.04	0.027	1.405	143.667	5.0	149456.0	5387.0	125041.0	29891.2
SPAdes	8.97	47.0	99.918	0.037	0.027	0.162	280.0	19.0	205739.0	144.0	71345.0	10828.4
SSAKE	2.64	6.0	99.979	0.81	0.782	0.061	100.0	202.0	47187.0	103.0	809.667	233.567
TRACESPipe	180.16	147.0	99.896	0.034	0.024	9.768	358.333	4.0	143860.0	5387.0	125041.0	35965.0
TRACESPipeLite	41.75	135.667	99.897	0.034	0.024	2.818	332.667	6.0	168262.0	5387.0	125041.0	28043.7
VirGenA	692.265	46.0	99.864	0.627	0.596	4.444	718.333	72.0	59531.0	61.0	3075.0	826.8
ViSpA	68.98	150.0	99.889	0.037	0.027	5.775	103.667	6.0	197005.0	5356.0	124834.0	32834.2
V-pipe	42.695	145.0	99.134	0.071	0.054	0.104	114.667	5.0	160429.0	5387.0	125041.0	32085.8

Table S17: Results obtained for DS13 using the benchmark proposed. The execution time was measured in seconds, the RAM usage was measured in GB and the CPU usage is presented as a percentage. The executions were, when possible, capped at 6 threads and 48 GB of RAM.

Reconstruction tool	Execution time	SNPs	Identity	NCSD	NRC	RAM usage	CPU usage	Number of scaffolds	Reconstructed bases	Minimum scaffold length	Maximum scaffold length	Average scaffold length
coronaSPAdes	3.07	44.0	99.821	0.036	0.025	0.149	422.333	14.0	204248.0	81.0	105072.0	14589.1
Haploflow	3.55	92.0	99.913	0.054	0.042	0.099	99.333	42.0	208025.0	631.0	17503.0	4953.0
IRMA	166.675	0.0	100.0	0.096	0.128	0.877	540.667	5.0	144809.0	4884.0	110215.0	28961.8
LAZYPIPE	6.745	56.0	99.932	0.037	0.025	1.525	265.0	7.0	202811.0	314.0	117612.0	28973.0
metaSPAdes	5.575	59.0	99.861	0.037	0.026	0.229	416.667	13.0	203277.0	71.0	84069.0	15636.7
metaviralSPAdes	–	–	–	–	–	–	–	–	–	–	–	–
PEHaplo	31.845	87.0	99.937	0.062	0.047	1.173	132.333	305.0	249689.0	380.0	3353.0	818.7
QuRe	448.215	0.0	100.0	0.998	0.989	19.991	573.0	5.0	911.333	97.0	354.0	184.133
QVG	24.215	157.0	99.892	0.032	0.024	1.409	156.667	5.0	268901.0	5387.0	125041.0	53780.2
SPAdes	7.09	36.0	99.906	0.036	0.025	0.162	374.0	21.0	211829.0	131.0	105080.0	10087.1
SSAKE	5.59	21.0	99.968	0.511	0.477	0.079	100.0	364.667	116251.0	101.333	1365.0	318.7
TRACESPipe	177.0	143.0	99.902	0.032	0.022	9.768	371.0	4.0	143860.0	5387.0	125041.0	35965.0
TRACESPipeLite	45.255	124.0	99.9	0.033	0.023	2.82	322.333	6.0	168262.0	5387.0	125041.0	28043.7
VirGenA	811.215	45.0	99.882	0.647	0.614	4.22	740.0	66.0	57997.0	51.0	3006.0	878.7
ViSpA	138.105	153.0	99.893	0.034	0.025	6.249	111.0	5.0	160089.0	5343.0	124970.0	32017.8
V-pipe	46.535	128.0	99.765	0.043	0.032	0.104	114.0	5.0	160429.0	5387.0	125041.0	32085.8

Table S18: Results obtained for DS14 using the benchmark proposed. The execution time was measured in seconds, the RAM usage was measured in GB and the CPU usage is presented as a percentage. The executions were, when possible, capped at 6 threads and 48 GB of RAM.

Reconstruction tool	Execution time	SNPs	Identity	NCSD	NRC	RAM usage	CPU usage	Number of scaffolds	Reconstructed bases	Minimum scaffold length	Maximum scaffold length	Average scaffold length
coronaSPAdes	3.45	63.0	99.91	0.036	0.025	0.149	431.0	10.0	203804.0	81.0	112188.0	20380.4
Haploflow	4.24	80.0	99.92	0.044	0.034	0.112	99.0	30.0	213645.0	752.0	23263.0	7121.5
IRMA	201.46	0.0	100.0	0.095	0.128	1.069	562.0	5.0	144874.0	4878.0	110250.0	28974.8
LAZYPIPE	7.56	61.0	99.915	0.036	0.025	1.529	268.0	10.0	203315.0	425.0	92634.0	20331.5
metaSPAdes	6.365	31.0	99.823	0.037	0.026	0.23	420.333	12.0	203510.0	71.0	105136.0	16959.2
metaviralSPAdes	–	–	–	–	–	–	–	–	–	–	–	–
PEHaplo	48.675	61.0	99.954	0.072	0.057	1.594	157.667	289.0	258898.0	376.0	3938.0	895.8
QuRe	520.955	4.333	99.743	0.993	0.979	17.191	580.0	4.333	2053.333	215.667	888.0	478.7
QVG	26.675	150.0	99.893	0.034	0.024	1.412	158.0	4.0	143860.0	5387.0	125041.0	35965.0
SPAdes	7.39	24.0	99.938	0.035	0.024	0.166	392.0	25.0	213118.0	167.0	105954.0	8524.7
SSAKE	8.7	26.333	99.973	0.193	0.167	0.096	100.0	475.0	191768.333	103.0	1626.0	403.833
TRACESPipe	177.085	136.0	99.903	0.033	0.023	9.768	358.0	4.0	143861.0	5387.0	125042.0	35965.3
TRACESPipeLite	47.055	159.333	99.888	0.033	0.023	2.822	316.0	6.0	168262.0	5387.0	125041.0	28043.7
VirGenA	924.645	44.0	99.867	0.635	0.605	4.426	729.0	57.667	57130.333	159.333	3971.0	1034.4
ViSpA	227.875	142.0	99.901	0.034	0.024	6.886	117.667	5.0	160249.0	5369.0	125022.0	32049.8
V-pipe	51.565	134.0	99.89	0.035	0.025	0.104	113.667	5.0	160429.0	5387.0	125041.0	32085.8

Table S19: Results obtained for DS15 using the benchmark proposed. The execution time was measured in seconds, the RAM usage was measured in GB and the CPU usage is presented as a percentage. The executions were, when possible, capped at 6 threads and 48 GB of RAM.

Reconstruction tool	Execution time	SNPs	Identity	NCSD	NRC	RAM usage	CPU usage	Number of scaffolds	Reconstructed bases	Minimum scaffold length	Maximum scaffold length	Average scaffold length
coronaSPAdes	3.95	52.0	99.916	0.036	0.025	0.156	434.333	12.0	204047.0	81.0	105120.0	17003.9
Haploflow	5.015	103.0	99.908	0.035	0.026	0.125	99.333	20.0	214389.0	1571.0	64331.0	10719.5
IRMA	237.415	0.0	100.0	0.095	0.127	1.265	581.667	5.0	144895.0	4888.0	110259.0	28979.0
LAZYPIPE	7.785	66.0	99.929	0.036	0.025	1.533	254.0	8.0	202788.0	5330.0	95777.0	25348.5
metaSPAdes	7.14	40.0	99.836	0.037	0.026	0.229	424.0	13.0	203610.0	71.0	105136.0	15662.3
metaviralSPAdes	–	–	–	–	–	–	–	–	–	–	–	–
PEHaplo	59.915	66.0	99.952	0.057	0.045	2.0	155.0	313.0	292885.0	382.0	2814.0	935.7
QuRe	931.685	21.333	99.609	0.988	0.973	20.736	605.333	5.0	4849.667	139.0	1503.667	851.733
QVG	29.835	148.0	99.893	0.034	0.024	1.413	160.333	4.0	143860.0	5387.0	125041.0	35965.0
SPAdes	8.485	13.0	99.947	0.033	0.023	0.175	397.333	27.0	208155.0	131.0	105954.0	7709.4
SSAKE	10.43	28.333	99.926	0.084	0.067	0.109	100.0	405.667	219691.667	100.333	3179.0	541.6
TRACESPipe	184.94	140.0	99.904	0.033	0.024	9.768	362.0	4.0	143860.0	5387.0	125041.0	35965.0
TRACESPipeLite	48.405	130.333	99.908	0.032	0.023	2.824	307.333	6.0	168262.0	5387.0	125041.0	28043.7
VirGenA	923.85	43.0	99.888	0.647	0.62	4.896	741.0	69.0	56205.0	54.0	3365.0	814.6
ViSpA	292.765	166.0	99.884	0.035	0.024	6.876	123.333	5.0	160211.0	5368.0	125006.0	32042.2
V-pipe	55.025	165.0	99.887	0.035	0.025	0.104	113.667	5.0	160429.0	5387.0	125041.0	32085.8

Table S20: Results obtained for DS16 using the benchmark proposed. The execution time was measured in seconds, the RAM usage was measured in GB and the CPU usage is presented as a percentage. The executions were, when possible, capped at 6 threads and 48 GB of RAM.

**Reconstruction tool**	**Execution time**	**SNPs**	**Identity**	**NCSD**	**NRC**	**RAM usage**	**CPU usage**	**Number of scaffolds**	**Reconstructed bases**	**Minimum scaffold length**	**Maximum scaffold length**	**Average scaffold length**
coronaSPAdes	4.66	47.0	99.923	0.036	0.025	0.153	444.667	10.0	203843.0	81.0	112188.0	20384.3
Haploflow	6.52	113.0	99.876	0.033	0.024	0.151	99.667	12.0	211760.0	2592.0	88805.0	17646.7
IRMA	308.225	0.0	100.0	0.095	0.127	1.651	598.0	5.0	144906.0	4885.0	110253.0	28981.2
LAZYPIPE	9.555	53.0	99.942	0.036	0.025	1.54	270.667	9.0	203432.0	5371.0	88951.0	22603.6
metaSPAdes	8.68	24.0	99.828	0.037	0.026	0.227	432.0	12.0	203549.0	71.0	105136.0	16962.4
metaviralSPAdes	10.065	0.0	0.0	1.0	0.994	0.226	453.0	2.0	66553.0	16561.0	49992.0	33276.5
PEHaplo	81.155	64.0	99.938	0.038	0.029	2.988	149.667	220.0	321707.0	395.0	5006.0	1462.3
QuRe	1980.37	31.333	99.689	0.936	0.898	21.231	638.667	8.667	26562.667	410.667	5081.0	3012.8
QVG	33.705	147.0	99.894	0.034	0.024	1.418	174.0	4.0	143860.0	5387.0	125041.0	35965.0
SPAdes	9.73	22.0	99.94	0.034	0.023	0.185	410.333	27.0	213297.0	131.0	105954.0	7899.9
SSAKE	16.85	11.667	99.989	0.04	0.03	0.132	99.667	214.0	225447.333	107.0	8185.0	1055.367
TRACESPipe	187.29	151.0	99.893	0.032	0.023	9.768	365.667	4.0	143860.0	5387.0	125041.0	35965.0
TRACESPipeLite	53.835	142.667	99.9	0.032	0.023	2.827	293.0	6.0	168262.0	5387.0	125041.0	28043.7
VirGenA	1174.83	40.0	99.844	0.629	0.596	5.112	745.667	55.0	59033.0	117.0	2741.0	1073.3
ViSpA	478.625	164.0	99.884	0.034	0.024	6.95	128.333	5.0	160258.0	5371.0	125015.0	32051.6
V-pipe	63.495	132.0	99.904	0.034	0.024	0.104	113.0	5.0	160429.0	5387.0	125041.0	32085.8

Table S21: Results obtained for DS17 using the benchmark proposed. The execution time was measured in seconds, the RAM usage was measured in GB and the CPU usage is presented as a percentage. The executions were, when possible, capped at 6 threads and 48 GB of RAM.

**Reconstruction tool**	**Execution time**	**SNPs**	**Identity**	**NCSD**	**NRC**	**RAM usage**	**CPU usage**	**Number of scaffolds**	**Reconstructed bases**	**Minimum scaffold length**	**Maximum scaffold length**	**Average scaffold length**
coronaSPAdes	1.25	95.0	99.886	0.448	0.413	0.134	355.333	259.0	122290.0	100.0	1626.0	472.2
Haploflow	–	–	–	–	–	–	–	–	–	–	–	–
IRMA	38.91	108.333	95.83	0.371	0.379	0.129	326.667	5.0	108214.0	3983.0	82349.0	21642.8
LAZYPIPE	4.09	29.0	99.949	0.62	0.583	1.495	178.333	159.0	82856.0	306.0	1572.0	521.1
metaSPAdes	2.075	83.0	99.909	0.393	0.357	0.225	326.667	306.0	132688.0	56.0	1626.0	433.6
metaviralSPAdes	–	–	–	–	–	–	–	–	–	–	–	–
PEHaplo	9.67	654.333	99.878	0.603	0.566	0.091	62.0	137.0	86539.0	375.0	1552.0	631.7
QuRe	–	–	–	–	–	–	–	–	–	–	–	–
QVG	–	–	–	–	–	–	–	–	–	–	–	–
SPAdes	–	–	–	–	–	–	–	–	–	–	–	–
SSAKE	2.62	0.0	100.0	0.999	0.993	0.049	100.0	2.667	410.0	141.333	161.667	150.167
TRACESPipe	164.61	15.0	97.482	0.324	0.287	9.768	361.0	4.0	143860.0	5387.0	125041.0	35965.0
TRACESPipeLite	37.01	14.333	96.444	0.317	0.286	2.776	362.0	6.0	168262.0	5387.0	125041.0	28043.7
VirGenA	263.35	55.0	99.867	0.576	0.543	3.824	608.333	214.0	68240.0	52.0	1205.0	318.9
ViSpA	43.22	54.0	96.054	0.501	0.482	5.525	56.333	86.0	2411952.0	450.0	70566.0	28046.0
V-pipe	30.85	0.0	95.912	0.975	0.961	0.104	115.333	5.0	160429.0	5387.0	125041.0	32085.8

Table S22: Results obtained for DS18 using the benchmark proposed. The execution time was measured in seconds, the RAM usage was measured in GB and the CPU usage is presented as a percentage. The executions were, when possible, capped at 6 threads and 48 GB of RAM.

**Reconstruction tool**	**Execution time**	**SNPs**	**Identity**	**NCSD**	**NRC**	**RAM usage**	**CPU usage**	**Number of scaffolds**	**Reconstructed bases**	**Minimum scaffold length**	**Maximum scaffold length**	**Average scaffold length**
coronaSPAdes	1.27	215.0	99.73	0.492	0.456	0.135	357.0	272.0	120288.0	224.0	1540.0	442.2
Haploflow	–	–	–	–	–	–	–	–	–	–	–	–
IRMA	42.26	115.0	95.586	0.412	0.409	0.122	326.667	5.0	104407.0	3598.0	79183.0	20881.4
LAZYPIPE	4.225	119.0	99.741	0.668	0.631	1.495	188.333	157.0	75760.0	301.0	1464.0	482.5
metaSPAdes	2.095	152.0	99.818	0.447	0.409	0.225	330.333	327.0	130710.0	97.0	1540.0	399.7
metaviralSPAdes	–	–	–	–	–	–	–	–	–	–	–	–
PEHaplo	9.575	2506.667	99.631	0.635	0.6	0.091	62.333	132.0	80018.0	375.0	2641.0	606.2
QuRe	–	–	–	–	–	–	–	–	–	–	–	–
QVG	14.94	4037.0	96.924	0.183	0.16	1.389	103.0	3.0	138473.0	5596.0	125041.0	46157.7
SPAdes	–	–	–	–	–	–	–	–	–	–	–	–
SSAKE	2.695	0.0	100.0	1.0	0.995	0.049	100.0	1.667	224.667	134.667	140.333	137.333
TRACESPipe	159.8	264.0	96.898	0.344	0.311	9.768	362.0	4.0	143859.0	5387.0	125040.0	35964.8
TRACESPipeLite	37.09	116.333	97.189	0.435	0.403	2.779	366.0	6.0	168076.0	5201.0	125041.0	28012.7
VirGenA	239.27	112.0	99.808	0.589	0.556	3.906	593.333	211.0	68194.0	62.0	1072.0	323.2
ViSpA	42.02	158.333	94.834	0.542	0.519	5.458	59.333	81.0	2419083.0	390.0	69277.0	29865.2
V-pipe	30.93	6.0	95.968	0.97	0.955	0.104	115.0	5.0	160429.0	5387.0	125041.0	32085.8

Table S23: Results obtained for DS19 using the benchmark proposed. The execution time was measured in seconds, the RAM usage was measured in GB and the CPU usage is presented as a percentage. The executions were, when possible, capped at 6 threads and 48 GB of RAM.

Reconstruction tool	Execution time	SNPs	Identity	NCSD	NRC	RAM usage	CPU usage	Number of scaffolds	Reconstructed bases	Minimum scaffold length	Maximum scaffold length	Average scaffold length
coronaSPAdes	1.27	164.0	99.656	0.48	0.446	0.134	357.667	274.0	124875.0	226.0	1302.0	455.7
Haploflow	–	–	–	–	–	–	–	–	–	–	–	–
IRMA	45.655	120.667	96.229	0.4	0.395	0.126	328.333	5.0	106677.0	3844.0	80453.0	21335.4
LAZYPIPE	3.935	60.0	99.773	0.633	0.603	1.495	174.667	182.0	86241.0	297.0	1144.0	473.9
metaSPAdes	2.105	126.0	99.747	0.439	0.407	0.224	328.333	323.0	134539.0	206.0	1235.0	416.5
metaviralSPAdes	–	–	–	–	–	–	–	–	–	–	–	–
PEHaplo	9.625	1272.0	99.598	0.632	0.6	0.091	62.333	147.0	86902.0	377.0	1507.0	591.2
QuRe	–	–	–	–	–	–	–	–	–	–	–	–
QVG	15.81	6858.0	95.231	0.207	0.186	1.389	102.333	4.0	143860.0	5387.0	125041.0	35965.0
SPAdes	–	–	–	–	–	–	–	–	–	–	–	–
SSAKE	2.62	0.0	100.0	1.0	0.996	0.049	100.0	4.5	672.5	123.5	173.5	149.7
TRACESPipe	159.595	555.0	96.286	0.324	0.295	9.768	361.667	4.0	143859.0	5387.0	125040.0	35964.8
TRACESPipeLite	36.79	71.0	98.034	0.693	0.667	2.782	365.0	6.0	168246.0	5387.0	125041.0	28041.0
VirGenA	241.31	85.0	99.79	0.608	0.58	3.738	600.667	195.0	64953.0	54.0	1673.0	333.1
ViSpA	41.965	299.0	94.594	0.536	0.518	5.581	57.667	86.0	2370844.0	744.0	69927.0	27568.0
V-pipe	30.795	1.0	95.875	0.972	0.959	0.104	115.333	5.0	160429.0	5387.0	125041.0	32085.8

Table S24: Results obtained for DS20 using the benchmark proposed. The execution time was measured in seconds, the RAM usage was measured in GB and the CPU usage is presented as a percentage. The executions were, when possible, capped at 6 threads and 48 GB of RAM.

Reconstruction tool	Execution time	SNPs	Identity	NCSD	NRC	RAM usage	CPU usage	Number of scaffolds	Reconstructed bases	Minimum scaffold length	Maximum scaffold length	Average scaffold length
coronaSPAdes	1.25	245.0	99.694	0.501	0.473	0.134	355.333	265.0	117128.0	224.0	1400.0	442.0
Haploflow	–	–	–	–	–	–	–	–	–	–	–	–
IRMA	44.69	120.0	96.585	0.41	0.401	0.124	332.333	5.0	105256.0	3636.0	79641.0	21051.2
LAZYPIPE	3.95	48.0	99.831	0.679	0.659	1.495	180.667	154.0	72806.0	301.0	1331.0	472.8
metaSPAdes	2.09	163.0	99.805	0.442	0.415	0.225	328.333	327.0	130602.0	206.0	1400.0	399.4
metaviralSPAdes	–	–	–	–	–	–	–	–	–	–	–	–
PEHaplo	9.63	2558.0	99.72	0.639	0.613	0.091	62.333	138.0	81582.0	377.0	1409.0	591.2
QuRe	–	–	–	–	–	–	–	–	–	–	–	–
QVG	15.93	9676.0	93.217	0.264	0.246	1.389	103.0	4.0	143860.0	5387.0	125041.0	35965.0
SPAdes	–	–	–	–	–	–	–	–	–	–	–	–
SSAKE	2.71	0.0	100.0	0.999	0.994	0.049	99.667	4.333	674.333	129.667	182.0	156.067
TRACESPipe	164.135	580.0	96.547	0.368	0.34	9.768	346.0	4.0	143658.0	5185.0	125041.0	35914.5
TRACESPipeLite	36.01	3.0	98.918	0.951	0.943	2.783	369.333	6.0	168060.0	5201.0	125041.0	28010.0
VirGenA	243.54	75.333	99.82	0.601	0.578	3.817	603.667	201.667	66410.667	72.0	1400.0	329.967
ViSpA	39.75	231.333	95.15	0.553	0.539	5.384	58.0	77.0	2157354.0	412.0	66879.0	28017.6
V-pipe	30.835	7.0	96.532	0.967	0.956	0.104	115.667	5.0	160429.0	5387.0	125041.0	32085.8

Table S25: Results obtained for DS21 using the benchmark proposed. The execution time was measured in seconds, the RAM usage was measured in GB and the CPU usage is presented as a percentage. The executions were, when possible, capped at 6 threads and 48 GB of RAM.

Reconstruction tool	Execution time	SNPs	Identity	NCSD	NRC	RAM usage	CPU usage	Number of scaffolds	Reconstructed bases	Minimum scaffold length	Maximum scaffold length	Average scaffold length
coronaSPAdes	1.25	143.0	99.663	0.473	0.446	0.134	356.0	279.0	124323.0	224.0	1491.0	445.6
Haploflow	–	–	–	–	–	–	–	–	–	–	–	–
IRMA	52.525	79.667	96.301	0.413	0.402	0.126	333.667	5.0	105334.0	3432.0	80110.0	21066.8
LAZYPIPE	3.93	75.0	99.864	0.663	0.64	1.496	172.667	169.0	80739.0	298.0	1388.0	477.7
metaSPAdes	2.105	104.0	99.743	0.43	0.404	0.225	326.667	329.0	135092.0	206.0	1491.0	410.6
metaviralSPAdes	–	–	–	–	–	–	–	–	–	–	–	–
PEHaplo	9.76	2053.0	99.933	0.638	0.618	0.091	63.0	147.0	88046.0	376.0	1768.0	599.0
QuRe	–	–	–	–	–	–	–	–	–	–	–	–
QVG	16.015	12401.0	91.349	0.322	0.304	1.389	102.0	4.0	143860.0	5387.0	125041.0	35965.0
SPAdes	–	–	–	–	–	–	–	–	–	–	–	–
SSAKE	2.755	0.0	100.0	1.0	0.997	0.05	100.0	3.0	456.5	141.5	166.0	152.2
TRACESPipe	154.63	1200.0	95.608	0.468	0.441	9.768	366.667	4.0	143862.0	5387.0	125043.0	35965.5
TRACESPipeLite	36.23	0.0	99.886	0.989	0.984	2.786	367.333	6.0	168251.0	5387.0	125030.0	28041.8
VirGenA	246.785	114.667	99.715	0.602	0.58	3.823	600.0	194.333	66372.667	80.333	1257.0	342.0
ViSpA	32.7	248.0	94.797	0.6	0.59	5.221	58.667	69.0	1578880.0	282.0	61010.0	22882.3
V-pipe	30.975	5.0	94.181	0.974	0.965	0.104	115.0	5.0	160429.0	5387.0	125041.0	32085.8

Table S26: Results obtained for DS22 using the benchmark proposed. The execution time was measured in seconds, the RAM usage was measured in GB and the CPU usage is presented as a percentage. The executions were, when possible, capped at 6 threads and 48 GB of RAM.

Reconstruction tool	Execution time	SNPs	Identity	NCSD	NRC	RAM usage	CPU usage	Number of scaffolds	Reconstructed bases	Minimum scaffold length	Maximum scaffold length	Average scaffold length
coronaSPAdes	1.26	126.0	99.848	0.488	0.467	0.134	355.667	263.0	121202.0	224.0	2079.0	460.8
Haploflow	–	–	–	–	–	–	–	–	–	–	–	–
IRMA	55.205	118.0	96.011	0.429	0.411	0.125	334.667	5.0	103635.667	3378.0	79458.0	20727.133
LAZYPIPE	4.23	36.0	99.934	0.666	0.649	1.496	183.667	163.0	81213.0	302.0	1958.0	498.2
metaSPAdes	2.085	85.0	99.902	0.437	0.414	0.225	326.0	314.0	132017.0	206.0	2079.0	420.4
metaviralSPAdes	–	–	–	–	–	–	–	–	–	–	–	–
PEHaplo	9.715	1950.0	99.939	0.641	0.624	0.091	62.667	142.0	85791.0	375.0	2079.0	604.2
QuRe	–	–	–	–	–	–	–	–	–	–	–	–
QVG	15.49	15309.0	89.322	0.402	0.389	1.389	102.0	4.0	143860.0	5387.0	125041.0	35965.0
SPAdes	–	–	–	–	–	–	–	–	–	–	–	–
SSAKE	2.715	0.0	100.0	1.0	0.997	0.05	100.0	4.0	613.333	123.667	203.333	156.2
TRACESPipe	176.48	1294.0	96.259	0.585	0.559	9.768	336.0	4.0	143658.0	5185.0	125041.0	35914.5
TRACESPipeLite	34.65	0.0	100.0	1.0	0.998	2.787	368.333	6.0	168262.0	5387.0	125041.0	28043.7
VirGenA	239.175	99.0	99.833	0.604	0.584	3.876	598.0	208.0	66126.0	52.0	1445.0	317.9
ViSpA	26.6	175.0	95.432	0.685	0.675	4.889	58.667	49.0	962393.0	0.0	50461.0	19640.7
V-pipe	30.695	5.0	97.153	0.978	0.969	0.104	116.0	5.0	160429.0	5387.0	125041.0	32085.8

Table S27: Results obtained for DS23 using the benchmark proposed. The execution time was measured in seconds, the RAM usage was measured in GB and the CPU usage is presented as a percentage. The executions were, when possible, capped at 6 threads and 48 GB of RAM.

Reconstruction tool	Execution time	SNPs	Identity	NCSD	NRC	RAM usage	CPU usage	Number of scaffolds	Reconstructed bases	Minimum scaffold length	Maximum scaffold length	Average scaffold length
coronaSPAdes	1.275	149.0	99.794	0.47	0.446	0.134	356.0	265.0	124902.0	225.0	1537.0	471.3
Haploflow	0.63	1.0	99.84	0.999	0.996	0.034	99.0	1.0	611.0	611.0	611.0	611.0
IRMA	57.29	101.667	96.919	0.452	0.431	0.122	344.0	5.0	100766.667	3252.0	77451.0	20153.333
LAZYPIPE	3.87	67.0	99.885	0.646	0.626	1.495	176.0	178.0	87278.0	297.0	1496.0	490.3
metaSPAdes	2.095	96.0	99.895	0.43	0.406	0.224	327.667	307.0	133617.0	206.0	1537.0	435.2
metaviralSPAdes	–	–	–	–	–	–	–	–	–	–	–	–
PEHaplo	9.69	1047.0	99.912	0.662	0.643	0.091	62.333	143.0	86513.0	375.0	1566.0	605.0
QuRe	–	–	–	–	–	–	–	–	–	–	–	–
QVG	15.89	18093.0	87.202	0.504	0.489	1.389	102.667	4.0	143860.0	5387.0	125041.0	35965.0
SPAdes	–	–	–	–	–	–	–	–	–	–	–	–
SSAKE	2.635	0.0	100.0	1.0	0.998	0.049	100.0	4.667	621.333	113.333	149.333	132.967
TRACESPipe	173.885	1082.0	96.453	0.632	0.609	9.768	337.0	4.0	143814.0	5341.0	125041.0	35953.5
TRACESPipeLite	33.535	0.0	0.0	1.0	0.998	2.788	380.667	5.0	160429.0	5387.0	125041.0	32085.8
VirGenA	226.8	71.0	99.849	0.597	0.576	3.826	594.333	210.0	67346.0	51.0	971.0	320.7
ViSpA	21.325	100.0	95.819	0.813	0.799	4.838	53.667	24.0	216346.0	0.0	32011.0	9014.4
V-pipe	30.995	0.0	92.188	0.991	0.985	0.103	115.0	5.0	160429.0	5387.0	125041.0	32085.8

Table S28: Results obtained for DS24 using the benchmark proposed. The execution time was measured in seconds, the RAM usage was measured in GB and the CPU usage is presented as a percentage. The executions were, when possible, capped at 6 threads and 48 GB of RAM.

Reconstruction tool	Execution time	SNPs	Identity	NCSD	NRC	RAM usage	CPU usage	Number of scaffolds	Reconstructed bases	Minimum scaffold length	Maximum scaffold length	Average scaffold length
coronaSPAdes	1.275	108.0	99.874	0.467	0.443	0.134	358.0	280.0	124665.0	225.0	1850.0	445.2
Haploflow	0.64	1.0	99.84	0.999	0.997	0.035	99.333	1.0	630.0	630.0	630.0	630.0
IRMA	55.53	124.0	97.017	0.471	0.446	0.118	340.0	5.0	98844.667	3403.0	75189.667	19768.933
LAZYPIPE	3.715	32.0	99.952	0.593	0.571	1.496	153.333	228.0	96038.0	215.0	1807.0	421.2
metaSPAdes	2.115	108.0	99.88	0.435	0.411	0.224	328.333	321.0	133395.0	206.0	1850.0	415.6
metaviralSPAdes	–	–	–	–	–	–	–	–	–	–	–	–
PEHaplo	9.7	2425.0	99.915	0.638	0.617	0.091	62.667	156.0	89145.0	375.0	1281.0	571.4
QuRe	–	–	–	–	–	–	–	–	–	–	–	–
QVG	16.91	20734.0	85.427	0.632	0.62	1.389	102.333	5.0	268901.0	5387.0	125041.0	53780.2
SPAdes	–	–	–	–	–	–	–	–	–	–	–	–
SSAKE	2.7	0.0	100.0	1.0	0.998	0.049	100.0	6.0	977.667	140.667	205.333	170.367
TRACESPipe	170.865	1107.0	96.089	0.703	0.681	9.768	339.0	4.0	143852.0	5379.0	125041.0	35963.0
TRACESPipeLite	33.675	0.0	0.0	1.0	0.999	2.791	378.333	5.0	160227.0	5185.0	125041.0	32045.4
VirGenA	250.375	80.667	99.849	0.59	0.568	4.029	602.0	223.333	69013.667	48.333	1190.667	309.0
ViSpA	18.21	77.0	97.678	0.909	0.9	1.675	48.0	15.0	88580.0	0.0	18761.0	5905.3
V-pipe	30.66	0.0	96.533	0.993	0.986	0.103	115.333	5.0	160429.0	5387.0	125041.0	32085.8

Table S29: Results obtained for DS25 using the benchmark proposed. The execution time was measured in seconds, the RAM usage was measured in GB and the CPU usage is presented as a percentage. The executions were, when possible, capped at 6 threads and 48 GB of RAM.

**Reconstruction tool**	**Execution time**	**SNPs**	**Identity**	**NCSD**	**NRC**	**RAM usage**	**CPU usage**	**Number of scaffolds**	**Reconstructed bases**	**Minimum scaffold length**	**Maximum scaffold length**	**Average scaffold length**
coronaSPAdes	1.65	84.0	99.832	0.101	0.08	0.135	395.667	162.0	194144.0	229.0	5668.0	1198.4
Haploflow	1.15	0.0	100.0	0.973	0.942	0.052	99.333	5.0	4928.0	527.0	1589.0	985.6
IRMA	74.755	48.667	98.435	0.141	0.17	0.276	388.667	5.0	140079.0	4702.0	106678.0	28015.8
LAZYPIPE	4.895	20.0	99.984	0.177	0.149	1.502	217.667	150.0	178673.0	310.0	3436.0	1191.2
metaSPAdes	61.665	89.667	99.912	0.188	0.159	0.227	587.667	260.667	180076.667	71.0	3641.333	691.067
metaviralSPAdes	–	–	–	–	–	–	–	–	–	–	–	–
PEHaplo	11.92	41.0	99.951	0.127	0.103	0.172	83.0	161.0	193494.0	383.0	5108.0	1201.8
QuRe	–	–	–	–	–	–	–	–	–	–	–	–
QVG	–	–	–	–	–	–	–	–	–	–	–	–
SPAdes	6.455	55.0	99.851	0.1	0.079	0.151	239.0	163.0	194317.0	229.0	4121.0	1192.1
SSAKE	5.995	0.667	99.99	0.953	0.926	0.067	100.0	82.0	12700.333	101.333	266.0	155.033
TRACESPipe	171.67	7.0	98.92	0.092	0.073	9.768	357.0	4.0	143860.0	5387.0	125041.0	35965.0
TRACESPipeLite	38.145	2.0	98.858	0.08	0.062	2.81	352.333	6.0	168262.0	5387.0	125041.0	28043.7
VirGenA	419.745	41.333	99.914	0.504	0.472	4.72	686.0	110.667	78565.667	141.0	2993.333	717.867
ViSpA	74.405	34.0	96.982	0.159	0.14	6.814	74.667	89.0	3712310.0	339.0	113271.0	41711.3
V-pipe	34.1	5.0	95.046	0.64	0.597	0.104	115.0	5.0	160429.0	5387.0	125041.0	32085.8

Table S30: Results obtained for DS26 using the benchmark proposed. The execution time was measured in seconds, the RAM usage was measured in GB and the CPU usage is presented as a percentage. The executions were, when possible, capped at 6 threads and 48 GB of RAM.

**Reconstruction tool**	**Execution time**	**SNPs**	**Identity**	**NCSD**	**NRC**	**RAM usage**	**CPU usage**	**Number of scaffolds**	**Reconstructed bases**	**Minimum scaffold length**	**Maximum scaffold length**	**Average scaffold length**
coronaSPAdes	1.67	186.0	99.737	0.116	0.093	0.14	397.667	162.0	197605.0	239.0	4192.0	1219.8
Haploflow	1.15	0.0	0.0	1.0	0.996	0.053	99.333	1.0	520.0	520.0	520.0	520.0
IRMA	67.125	60.333	98.239	0.163	0.177	0.277	389.333	5.0	139295.0	4696.0	106132.0	27859.0
LAZYPIPE	4.97	56.0	99.847	0.181	0.155	1.502	221.333	154.0	184203.0	302.0	4152.0	1196.1
metaSPAdes	83.69	127.333	99.861	0.298	0.264	0.227	590.667	305.667	163554.667	82.0	2060.333	535.067
metaviralSPAdes	–	–	–	–	–	–	–	–	–	–	–	–
PEHaplo	11.835	336.0	99.73	0.146	0.122	0.167	82.667	162.0	205525.0	375.0	3959.0	1268.7
QuRe	–	–	–	–	–	–	–	–	–	–	–	–
QVG	18.86	3136.0	97.748	0.126	0.106	1.395	116.0	5.0	268901.0	5387.0	125041.0	53780.2
SPAdes	3.44	247.0	99.807	0.118	0.095	0.151	361.667	158.0	194868.0	104.0	4192.0	1233.3
SSAKE	5.965	0.333	99.996	0.959	0.946	0.067	100.0	75.667	11887.0	101.667	244.333	157.267
TRACESPipe	171.055	502.0	98.527	0.102	0.083	9.768	354.667	4.0	143861.0	5387.0	125042.0	35965.3
TRACESPipeLite	38.94	226.333	98.125	0.149	0.126	2.813	358.667	6.0	168262.0	5387.0	125041.0	28043.7
VirGenA	377.075	73.333	99.773	0.587	0.558	4.269	686.667	125.667	66339.0	64.0	1416.667	527.867
ViSpA	77.01	128.0	97.005	0.165	0.148	6.568	75.333	86.0	3891818.0	773.0	113335.0	45253.7
V-pipe	34.115	58.0	94.635	0.662	0.632	0.104	115.0	5.0	160429.0	5387.0	125041.0	32085.8

Table S31: Results obtained for DS27 using the benchmark proposed. The execution time was measured in seconds, the RAM usage was measured in GB and the CPU usage is presented as a percentage. The executions were, when possible, capped at 6 threads and 48 GB of RAM.

**Reconstruction tool**	**Execution time**	**SNPs**	**Identity**	**NCSD**	**NRC**	**RAM usage**	**CPU usage**	**Number of scaffolds**	**Reconstructed bases**	**Minimum scaffold length**	**Maximum scaffold length**	**Average scaffold length**
coronaSPAdes	1.66	176.0	99.79	0.099	0.08	0.138	398.333	165.0	202532.0	233.0	5535.0	1227.5
Haploflow	–	–	–	–	–	–	–	–	–	–	–	–
IRMA	83.155	46.667	98.697	0.163	0.174	0.277	391.0	5.0	139708.0	4808.0	106434.0	27941.6
LAZYPIPE	4.97	60.0	99.934	0.176	0.152	1.501	222.667	150.0	186736.0	310.0	5458.0	1244.9
metaSPAdes	94.425	112.0	99.837	0.248	0.22	0.227	591.667	315.0	174803.0	206.0	2200.667	554.933
metaviralSPAdes	–	–	–	–	–	–	–	–	–	–	–	–
PEHaplo	11.64	348.0	99.603	0.14	0.119	0.169	82.333	171.0	201993.0	387.0	3882.0	1181.2
QuRe	–	–	–	–	–	–	–	–	–	–	–	–
QVG	18.35	5172.0	96.4	0.169	0.149	1.394	114.0	4.0	143860.0	5387.0	125041.0	35965.0
SPAdes	4.46	188.0	99.772	0.102	0.083	0.152	306.0	160.0	199571.0	104.0	5535.0	1247.3
SSAKE	6.13	0.333	99.996	0.963	0.952	0.068	100.0	70.0	11021.0	104.0	286.667	157.333
TRACESPipe	171.06	670.0	98.687	0.088	0.072	9.768	360.667	4.0	143861.0	5387.0	125042.0	35965.3
TRACESPipeLite	36.805	171.333	97.728	0.501	0.471	2.815	361.333	6.0	168262.0	5387.0	125041.0	28043.7
VirGenA	401.61	153.0	99.605	0.527	0.504	4.774	684.333	114.0	76231.667	93.0	2432.667	674.5
ViSpA	63.97	620.0	96.542	0.171	0.154	6.704	75.0	78.0	3366127.0	1456.0	113769.0	43155.5
V-pipe	34.535	61.0	95.529	0.67	0.642	0.104	115.0	5.0	160429.0	5387.0	125041.0	32085.8

Table S32: Results obtained for DS28 using the benchmark proposed. The execution time was measured in seconds, the RAM usage was measured in GB and the CPU usage is presented as a percentage. The executions were, when possible, capped at 6 threads and 48 GB of RAM.

**Reconstruction tool**	**Execution time**	**SNPs**	**Identity**	**NCSD**	**NRC**	**RAM usage**	**CPU usage**	**Number of scaffolds**	**Reconstructed bases**	**Minimum scaffold length**	**Maximum scaffold length**	**Average scaffold length**
coronaSPAdes	1.64	97.0	99.84	0.109	0.088	0.138	399.0	156.0	201020.0	225.0	4774.0	1288.6
Haploflow	–	–	–	–	–	–	–	–	–	–	–	–
IRMA	101.42	66.0	98.533	0.178	0.182	0.272	392.333	5.0	138542.0	4401.0	105504.0	27708.4
LAZYPIPE	4.97	24.0	99.94	0.171	0.146	1.502	218.0	149.0	187973.0	303.0	4720.0	1261.6
metaSPAdes	95.205	105.0	99.9	0.317	0.288	0.227	592.333	329.333	160981.0	206.0	2418.333	489.133
metaviralSPAdes	–	–	–	–	–	–	–	–	–	–	–	–
PEHaplo	12.045	323.0	99.525	0.137	0.117	0.17	118.667	166.0	221032.0	378.0	5395.0	1331.5
QuRe	–	–	–	–	–	–	–	–	–	–	–	–
QVG	19.625	7602.0	94.671	0.233	0.215	1.394	117.0	5.0	268901.0	5387.0	125041.0	53780.2
SPAdes	3.205	61.0	99.85	0.108	0.087	0.151	380.0	156.0	201005.0	230.0	4774.0	1288.5
SSAKE	6.0	0.333	99.996	0.963	0.954	0.068	100.0	68.667	10430.667	100.667	254.667	151.9
TRACESPipe	178.92	1322.0	98.045	0.116	0.097	9.768	344.667	4.0	143858.0	5387.0	125039.0	35964.5
TRACESPipeLite	37.02	7.0	98.161	0.9	0.886	2.816	362.333	6.0	168262.0	5387.0	125041.0	28043.7
VirGenA	384.205	126.0	99.474	0.561	0.534	4.382	679.667	126.0	71738.0	62.0	1745.0	569.3
ViSpA	62.585	911.0	96.58	0.189	0.173	7.765	78.667	90.0	3324538.0	872.0	112463.0	36939.3
V-pipe	34.81	182.0	94.928	0.678	0.648	0.104	115.0	5.0	160429.0	5387.0	125041.0	32085.8

Table S33: Results obtained for DS29 using the benchmark proposed. The execution time was measured in seconds, the RAM usage was measured in GB and the CPU usage is presented as a percentage. The executions were, when possible, capped at 6 threads and 48 GB of RAM.

**Reconstruction tool**	**Execution time**	**SNPs**	**Identity**	**NCSD**	**NRC**	**RAM usage**	**CPU usage**	**Number of scaffolds**	**Reconstructed bases**	**Minimum scaffold length**	**Maximum scaffold length**	**Average scaffold length**
coronaSPAdes	1.65	88.0	99.934	0.124	0.106	0.138	393.0	161.0	198559.0	231.0	6121.0	1233.3
Haploflow	1.15	0.0	0.0	1.0	0.998	0.053	99.667	1.0	511.0	511.0	511.0	511.0
IRMA	96.65	47.333	98.492	0.183	0.182	0.276	399.0	5.0	138702.0	4592.0	105718.0	27740.4
LAZYPIPE	4.195	17.0	99.987	0.169	0.148	1.502	194.667	160.0	188434.0	239.0	6018.0	1177.7
metaSPAdes	82.22	88.667	99.9	0.356	0.333	0.227	591.0	320.333	153235.333	207.0	2223.667	478.333
metaviralSPAdes	–	–	–	–	–	–	–	–	–	–	–	–
PEHaplo	11.695	212.0	99.763	0.157	0.137	0.169	82.0	170.0	197190.0	379.0	6615.0	1159.9
QuRe	–	–	–	–	–	–	–	–	–	–	–	–
QVG	21.93	9748.0	93.194	0.285	0.269	1.395	117.333	7.0	282124.0	5387.0	125041.0	40303.4
SPAdes	3.05	45.0	99.965	0.118	0.1	0.151	394.0	163.0	199677.0	231.0	6121.0	1225.0
SSAKE	6.12	0.0	100.0	0.96	0.951	0.068	100.0	75.667	11525.0	102.667	220.667	152.333
TRACESPipe	182.195	1149.0	97.348	0.177	0.156	9.768	345.333	4.0	143860.0	5387.0	125041.0	35965.0
TRACESPipeLite	37.595	0.0	98.534	0.99	0.985	2.818	362.333	6.0	168262.0	5387.0	125041.0	28043.7
VirGenA	379.705	94.0	99.752	0.615	0.593	4.223	685.667	121.0	63587.0	92.0	2157.0	525.5
ViSpA	80.93	807.0	95.473	0.248	0.236	6.841	75.333	107.0	4543976.0	804.0	105356.0	42467.1
V-pipe	35.18	187.0	94.365	0.684	0.66	0.104	114.333	5.0	160429.0	5387.0	125041.0	32085.8

Table S34: Results obtained for DS30 using the benchmark proposed. The execution time was measured in seconds, the RAM usage was measured in GB and the CPU usage is presented as a percentage. The executions were, when possible, capped at 6 threads and 48 GB of RAM.

**Reconstruction tool**	**Execution time**	**SNPs**	**Identity**	**NCSD**	**NRC**	**RAM usage**	**CPU usage**	**Number of scaffolds**	**Reconstructed bases**	**Minimum scaffold length**	**Maximum scaffold length**	**Average scaffold length**
coronaSPAdes	1.64	72.0	99.946	0.115	0.098	0.137	396.0	167.0	198003.0	231.0	4439.0	1185.6
Haploflow	1.14	0.0	100.0	0.999	0.997	0.053	99.0	1.0	540.0	540.0	540.0	540.0
IRMA	92.51	41.333	98.577	0.186	0.176	0.278	406.667	5.0	139538.0	4547.0	106270.0	27907.6
LAZYPIPE	4.425	24.0	99.981	0.176	0.156	1.502	206.0	155.0	183551.0	300.0	4357.0	1184.2
metaSPAdes	85.915	87.5	99.92	0.264	0.241	0.227	591.0	314.5	170446.0	206.0	2317.0	541.95
metaviralSPAdes	–	–	–	–	–	–	–	–	–	–	–	–
PEHaplo	11.7	36.0	99.943	0.14	0.122	0.156	79.0	168.0	200107.0	381.0	5666.0	1191.1
QuRe	–	–	–	–	–	–	–	–	–	–	–	–
QVG	22.13	12704.0	91.129	0.371	0.36	1.395	115.667	7.0	282124.0	5387.0	125041.0	40303.4
SPAdes	3.115	89.0	99.931	0.113	0.096	0.151	385.0	167.0	198125.0	231.0	4439.0	1186.4
SSAKE	6.065	1.333	99.983	0.965	0.956	0.069	100.0	77.0	12252.667	105.667	297.0	159.1
TRACESPipe	165.765	1352.0	97.63	0.203	0.184	9.768	351.0	4.0	143859.0	5386.0	125041.0	35964.8
TRACESPipeLite	36.75	0.0	0.0	1.001	0.998	2.818	365.0	6.0	168262.0	5387.0	125041.0	28043.7
VirGenA	389.84	32.0	99.755	0.62	0.602	4.315	684.667	112.0	62075.0	52.0	2320.0	554.2
ViSpA	84.435	688.0	94.351	0.342	0.332	6.119	70.667	133.0	5417908.0	500.0	94119.0	40736.2
V-pipe	34.785	173.0	94.003	0.728	0.706	0.104	114.0	5.0	160429.0	5387.0	125041.0	32085.8

Table S35: Results obtained for DS31 using the benchmark proposed. The execution time was measured in seconds, the RAM usage was measured in GB and the CPU usage is presented as a percentage. The executions were, when possible, capped at 6 threads and 48 GB of RAM.

Reconstruction tool	Execution time	SNPs	Identity	NCSD	NRC	RAM usage	CPU usage	Number of scaffolds	Reconstructed bases	Minimum scaffold length	Maximum scaffold length	Average scaffold length
coronaSPAdes	1.66	75.0	99.943	0.107	0.09	0.138	399.0	171.0	200252.0	230.0	6031.0	1171.1
Haploflow	1.15	0.0	100.0	0.998	0.997	0.053	99.0	1.0	533.0	533.0	533.0	533.0
IRMA	99.68	43.0	98.459	0.202	0.188	0.274	407.667	5.0	137704.0	4718.0	105011.0	27540.8
LAZYPIPE	4.2	20.0	99.984	0.173	0.153	1.501	195.333	164.0	186700.0	249.0	4643.0	1138.4
metaSPAdes	90.38	90.667	99.906	0.359	0.335	0.227	592.333	325.667	152286.0	206.0	1763.667	467.633
metaviralSPAdes	–	–	–	–	–	–	–	–	–	–	–	–
PEHaplo	11.62	364.0	99.956	0.147	0.128	0.166	82.333	163.0	194113.0	377.0	4461.0	1190.9
QuRe	–	–	–	–	–	–	–	–	–	–	–	–
QVG	20.56	16044.0	88.634	0.489	0.476	1.395	115.667	6.0	274501.0	5387.0	125043.0	45750.2
SPAdes	4.28	44.0	99.966	0.105	0.088	0.151	312.667	171.0	200309.0	230.0	6031.0	1171.4
SSAKE	6.145	0.0	100.0	0.969	0.959	0.068	100.0	62.667	10026.333	106.667	235.333	159.933
TRACESPipe	186.325	1812.0	97.055	0.237	0.218	9.768	333.333	4.0	143860.0	5387.0	125041.0	35965.0
TRACESPipeLite	37.58	0.0	0.0	1.0	0.998	2.819	362.333	6.0	168262.0	5387.0	125041.0	28043.7
VirGenA	390.005	34.0	99.907	0.591	0.572	4.396	678.333	112.333	66470.333	97.333	1887.0	594.333
ViSpA	50.215	292.667	94.943	0.526	0.515	7.026	71.333	107.0	3041834.0	450.0	71401.0	28428.4
V-pipe	34.28	162.0	93.088	0.792	0.771	0.104	114.667	5.0	160429.0	5387.0	125041.0	32085.8

Table S36: Results obtained for DS32 using the benchmark proposed. The execution time was measured in seconds, the RAM usage was measured in GB and the CPU usage is presented as a percentage. The executions were, when possible, capped at 6 threads and 48 GB of RAM.

Reconstruction tool	Execution time	SNPs	Identity	NCSD	NRC	RAM usage	CPU usage	Number of scaffolds	Reconstructed bases	Minimum scaffold length	Maximum scaffold length	Average scaffold length
coronaSPAdes	1.665	66.0	99.951	0.099	0.082	0.137	394.0	173.0	201373.0	230.0	4977.0	1164.0
Haploflow	–	–	–	–	–	–	–	–	–	–	–	–
IRMA	128.915	63.667	98.527	0.21	0.191	0.281	413.333	5.0	138139.333	4874.0	105461.333	27627.867
LAZYPIPE	3.975	19.0	99.986	0.151	0.133	1.502	180.667	172.0	189101.0	239.0	4894.0	1099.4
metaSPAdes	86.175	79.333	99.932	0.237	0.214	0.227	591.333	313.667	173362.0	206.0	2234.0	552.967
metaviralSPAdes	–	–	–	–	–	–	–	–	–	–	–	–
PEHaplo	11.685	34.0	99.963	0.128	0.11	0.156	82.0	163.0	197234.0	379.0	4460.0	1210.0
QuRe	–	–	–	–	–	–	–	–	–	–	–	–
QVG	21.015	19218.0	86.483	0.622	0.612	1.394	117.0	7.0	282329.0	5387.0	125039.0	40332.7
SPAdes	3.12	44.0	99.967	0.096	0.08	0.151	384.0	173.0	201591.0	230.0	4977.0	1165.3
SSAKE	6.015	0.0	100.0	0.971	0.96	0.068	100.0	73.0	11525.333	102.0	296.0	157.667
TRACESPipe	183.67	1406.0	97.524	0.303	0.283	9.768	339.0	4.0	143814.0	5341.0	125041.0	35953.5
TRACESPipeLite	37.17	0.0	0.0	1.0	0.999	2.82	364.0	6.0	168289.0	5387.0	125041.0	28048.2
VirGenA	400.325	56.667	99.747	0.604	0.584	4.473	686.0	124.333	65622.667	85.667	1602.333	533.633
ViSpA	28.805	248.667	95.949	0.74	0.729	6.256	67.333	54.0	1043140.0	404.0	44347.0	19317.4
V-pipe	34.075	72.0	93.219	0.856	0.837	0.104	114.667	5.0	160429.0	5387.0	125041.0	32085.8

Table S37: Results obtained for DS33 using the benchmark proposed. The execution time was measured in seconds, the RAM usage was measured in GB and the CPU usage is presented as a percentage. The executions were, when possible, capped at 6 threads and 48 GB of RAM.

Reconstruction tool	Execution time	SNPs	Identity	NCSD	NRC	RAM usage	CPU usage	Number of scaffolds	Reconstructed bases	Minimum scaffold length	Maximum scaffold length	Average scaffold length
coronaSPAdes	2.125	10.0	99.908	0.035	0.026	0.138	411.333	27.0	202192.0	165.0	41011.0	7488.6
Haploflow	2.04	0.0	100.0	0.621	0.576	0.069	99.333	106.0	84303.0	504.0	2747.0	795.3
IRMA	95.06	7.333	99.952	0.092	0.131	0.488	456.667	5.0	144638.0	4880.0	110164.0	28927.6
LAZYPIPE	5.47	4.0	99.995	0.041	0.033	1.509	248.333	24.0	201014.0	302.0	40981.0	8375.6
metaSPAdes	3.74	8.0	99.858	0.034	0.025	0.227	395.0	27.0	202264.0	71.0	41011.0	7491.3
metaviralSPAdes	–	–	–	–	–	–	–	–	–	–	–	–
PEHaplo	16.93	16.0	99.983	0.053	0.044	0.428	102.0	208.0	226110.0	379.0	5296.0	1087.1
QuRe	720.04	0.0	100.0	1.0	0.995	55.254	611.5	3.0	151.0	40.0	71.0	50.3
QVG	–	–	–	–	–	–	–	–	–	–	–	–
SPAdes	12.25	5.0	99.863	0.035	0.026	0.158	206.667	26.0	202307.0	165.0	41011.0	7781.0
SSAKE	1.665	1.0	99.994	0.916	0.889	0.049	100.0	115.667	21545.333	100.333	473.667	186.267
TRACESPipe	165.305	0.0	99.957	0.031	0.023	9.768	364.667	4.0	143860.0	5387.0	125041.0	35965.0
TRACESPipeLite	40.27	0.0	99.909	0.031	0.023	2.816	339.0	6.0	168262.0	5387.0	125041.0	28043.7
VirGenA	586.465	21.0	99.894	0.563	0.533	4.498	734.333	85.0	69043.0	226.0	2345.0	812.3
ViSpA	40.11	4.0	99.644	0.042	0.033	5.94	91.0	11.0	454253.0	2029.0	124437.0	41295.7
V-pipe	38.28	5.0	96.583	0.223	0.194	0.104	114.0	5.0	160429.0	5387.0	125041.0	32085.8

Table S38: Results obtained for DS34 using the benchmark proposed. The execution time was measured in seconds, the RAM usage was measured in GB and the CPU usage is presented as a percentage. The executions were, when possible, capped at 6 threads and 48 GB of RAM.

Reconstruction tool	Execution time	SNPs	Identity	NCSD	NRC	RAM usage	CPU usage	Number of scaffolds	Reconstructed bases	Minimum scaffold length	Maximum scaffold length	Average scaffold length
coronaSPAdes	2.19	55.0	99.96	0.04	0.028	0.154	409.0	33.0	207952.0	242.0	26757.0	6301.6
Haploflow	2.07	44.0	99.921	0.641	0.612	0.07	99.667	103.0	80047.0	502.0	2199.0	777.2
IRMA	110.605	3.0	99.946	0.106	0.13	0.484	457.333	5.0	144544.0	4878.0	110152.0	28908.8
LAZYPIPE	5.59	148.0	99.856	0.043	0.032	1.51	252.0	32.0	208755.0	328.0	26713.0	6523.6
metaSPAdes	3.815	40.0	99.924	0.04	0.029	0.227	397.667	39.0	208108.0	69.0	26757.0	5336.1
metaviralSPAdes	–	–	–	–	–	–	–	–	–	–	–	–
PEHaplo	17.035	232.0	99.753	0.059	0.047	0.418	107.0	240.0	243109.0	380.0	4924.0	1013.0
QuRe	619.59	0.0	100.0	1.0	0.995	52.658	577.333	1.0	94.0	94.0	94.0	94.0
QVG	21.43	1388.0	98.963	0.077	0.061	1.399	133.667	5.0	268901.0	5387.0	125041.0	53780.2
SPAdes	7.28	20.0	99.918	0.037	0.026	0.159	277.0	34.0	209307.0	85.0	26757.0	6156.1
SSAKE	1.645	0.333	99.998	0.932	0.919	0.049	100.0	118.0	20774.0	102.333	412.0	176.033
TRACESPipe	168.35	511.0	99.591	0.042	0.032	9.768	363.0	4.0	143862.0	5387.0	125043.0	35965.5
TRACESPipeLite	42.105	457.667	99.12	0.075	0.061	2.818	345.333	6.0	168262.0	5387.0	125041.0	28043.7
VirGenA	561.335	80.0	99.75	0.627	0.6	4.497	739.0	86.0	60061.0	57.0	2560.0	698.4
ViSpA	39.015	470.0	99.477	0.054	0.043	6.074	94.667	9.0	314557.0	2166.333	173078.333	40034.533
V-pipe	38.545	297.0	96.547	0.257	0.225	0.104	114.0	5.0	160429.0	5387.0	125041.0	32085.8

Table S39: Results obtained for DS35 using the benchmark proposed. The execution time was measured in seconds, the RAM usage was measured in GB and the CPU usage is presented as a percentage. The executions were, when possible, capped at 6 threads and 48 GB of RAM.

Reconstruction tool	Execution time	SNPs	Identity	NCSD	NRC	RAM usage	CPU usage	Number of scaffolds	Reconstructed bases	Minimum scaffold length	Maximum scaffold length	Average scaffold length
coronaSPAdes	2.1	28.0	99.976	0.038	0.028	0.136	414.667	25.0	209914.0	298.0	34355.0	8396.6
Haploflow	2.08	34.0	99.907	0.672	0.642	0.07	99.333	101.0	76112.0	502.0	1781.0	753.6
IRMA	144.775	6.667	99.851	0.114	0.133	0.478	459.0	5.0	144500.0	4887.0	110002.0	28900.0
LAZYPIPE	4.98	9.0	99.876	0.045	0.035	1.509	238.667	27.0	208600.0	363.0	26610.0	7725.9
metaSPAdes	3.845	29.0	99.974	0.039	0.029	0.227	398.667	30.0	209653.0	184.0	34355.0	6988.4
metaviralSPAdes	–	–	–	–	–	–	–	–	–	–	–	–
PEHaplo	16.905	114.0	99.787	0.067	0.053	0.419	102.0	221.0	235361.0	377.0	3273.0	1065.0
QuRe	–	–	–	–	–	–	–	–	–	–	–	–
QVG	23.075	2184.0	98.482	0.098	0.083	1.399	133.333	5.0	268901.0	5387.0	125041.0	53780.2
SPAdes	7.905	2.0	99.508	0.038	0.028	0.159	267.667	28.0	209843.0	83.0	34355.0	7494.4
SSAKE	1.7	0.333	99.998	0.924	0.908	0.049	100.0	116.667	21313.0	102.0	505.333	182.667
TRACESPipe	171.06	665.0	99.454	0.047	0.037	9.768	349.333	4.0	143860.0	5387.0	125042.0	35965.0
TRACESPipeLite	41.075	249.0	97.658	0.393	0.363	2.82	347.0	6.0	168262.0	5387.0	125041.0	28043.7
VirGenA	523.695	140.667	99.63	0.576	0.55	4.807	733.0	79.333	66812.333	149.0	2257.667	877.0
ViSpA	40.73	647.0	99.377	0.058	0.047	5.922	92.0	11.0	506252.0	5265.0	124298.0	46022.9
V-pipe	38.685	271.0	96.769	0.254	0.228	0.104	114.0	5.0	160429.0	5387.0	125041.0	32085.8

Table S40: Results obtained for DS36 using the benchmark proposed. The execution time was measured in seconds, the RAM usage was measured in GB and the CPU usage is presented as a percentage. The executions were, when possible, capped at 6 threads and 48 GB of RAM.

Reconstruction tool	Execution time	SNPs	Identity	NCSD	NRC	RAM usage	CPU usage	Number of scaffolds	Reconstructed bases	Minimum scaffold length	Maximum scaffold length	Average scaffold length
coronaSPAdes	2.12	19.0	99.986	0.034	0.024	0.135	414.667	16.0	210030.0	481.0	76427.0	13126.9
Haploflow	2.08	21.0	99.886	0.657	0.634	0.07	99.333	109.0	82555.0	501.0	2041.0	757.4
IRMA	148.69	4.667	99.975	0.117	0.13	0.487	468.667	5.0	144632.0	4794.0	110222.0	28926.4
LAZYPIPE	4.81	8.0	99.953	0.036	0.027	1.51	228.333	17.0	209331.0	447.0	76392.0	12313.6
metaSPAdes	3.855	16.0	99.986	0.033	0.024	0.227	394.333	17.0	210105.0	481.0	76427.0	12359.1
metaviralSPAdes	–	–	–	–	–	–	–	–	–	–	–	–
PEHaplo	16.81	157.0	99.806	0.064	0.053	0.419	102.0	200.0	229677.0	379.0	4327.0	1148.4
QuRe	602.145	0.0	100.0	1.0	0.996	52.768	621.667	2.0	117.0	47.0	70.0	58.5
QVG	24.935	3358.0	97.624	0.13	0.117	1.398	134.0	6.0	274288.0	5387.0	125041.0	45714.7
SPAdes	6.585	9.0	99.989	0.033	0.024	0.159	301.0	17.0	210126.0	481.0	76427.0	12360.4
SSAKE	1.71	0.333	99.998	0.921	0.908	0.049	100.0	116.0	22004.333	102.333	464.667	189.6
TRACESPipe	172.975	793.0	99.225	0.06	0.049	9.768	356.333	4.0	143869.0	5387.0	125050.0	35967.3
TRACESPipeLite	39.28	65.667	98.668	0.841	0.823	2.821	352.0	6.0	168262.0	5387.0	125041.0	28043.7
VirGenA	560.25	164.0	99.644	0.597	0.575	4.641	736.333	81.667	64253.667	144.667	2783.333	804.9
ViSpA	37.865	632.0	98.667	0.069	0.058	6.166	92.333	15.0	457100.0	4009.0	124187.0	30473.3
V-pipe	38.605	424.0	95.936	0.28	0.254	0.104	114.333	5.0	160429.0	5387.0	125041.0	32085.8

Table S41: Results obtained for DS37 using the benchmark proposed. The execution time was measured in seconds, the RAM usage was measured in GB and the CPU usage is presented as a percentage. The executions were, when possible, capped at 6 threads and 48 GB of RAM.

Reconstruction tool	Execution time	SNPs	Identity	NCSD	NRC	RAM usage	CPU usage	Number of scaffolds	Reconstructed bases	Minimum scaffold length	Maximum scaffold length	Average scaffold length
coronaSPAdes	2.11	12.0	99.99	0.033	0.025	0.134	415.333	24.0	210176.0	560.0	29993.0	8757.3
Haploflow	2.08	134.0	99.741	0.673	0.652	0.07	99.0	98.0	78659.0	501.0	2497.0	802.6
IRMA	151.255	12.333	99.925	0.119	0.127	0.482	476.333	5.0	145406.333	4867.0	110886.333	29081.267
LAZYPIPE	4.565	4.0	99.995	0.041	0.032	1.509	204.667	26.0	209151.0	526.0	29973.0	8044.3
metaSPAdes	3.765	7.0	99.994	0.032	0.024	0.227	393.667	24.0	210211.0	560.0	29993.0	8758.8
metaviralSPAdes	–	–	–	–	–	–	–	–	–	–	–	–
PEHaplo	16.755	109.0	99.925	0.063	0.051	0.418	101.667	202.0	230677.0	380.0	5122.0	1142.0
QuRe	168.765	0.0	100.0	1.0	0.997	12.763	629.0	3.0	334.0	98.0	118.0	111.3
QVG	26.29	4998.0	96.505	0.171	0.163	1.399	133.0	6.0	274497.0	5387.0	125041.0	45749.5
SPAdes	5.305	9.0	99.991	0.032	0.024	0.159	342.333	24.0	210211.0	560.0	29993.0	8758.8
SSAKE	1.82	1.333	99.991	0.927	0.913	0.05	100.0	114.333	21220.0	101.667	363.667	185.667
TRACESPipe	179.625	530.0	99.5	0.073	0.061	9.768	354.0	4.0	143860.0	5387.0	125041.0	35965.0
TRACESPipeLite	38.6	1.0	99.976	0.983	0.975	2.824	354.667	6.0	168262.0	5387.0	125041.0	28043.7
VirGenA	559.23	66.0	99.727	0.602	0.583	4.454	736.667	77.0	63709.333	229.667	2382.0	834.2
ViSpA	58.445	1051.333	97.847	0.097	0.085	5.949	91.0	41.0	2128909.0	1839.0	121179.0	51924.6
V-pipe	39.24	689.0	95.647	0.291	0.267	0.104	113.667	5.0	160429.0	5387.0	125041.0	32085.8

Table S42: Results obtained for DS38 using the benchmark proposed. The execution time was measured in seconds, the RAM usage was measured in GB and the CPU usage is presented as a percentage. The executions were, when possible, capped at 6 threads and 48 GB of RAM.

Reconstruction tool	Execution time	SNPs	Identity	NCSD	NRC	RAM usage	CPU usage	Number of scaffolds	Reconstructed bases	Minimum scaffold length	Maximum scaffold length	Average scaffold length
coronaSPAdes	2.08	13.0	99.989	0.034	0.026	0.136	413.333	19.0	210132.0	696.0	49969.0	11059.6
Haploflow	2.05	0.0	100.0	0.619	0.601	0.069	99.0	104.0	86123.0	503.0	2164.0	828.1
IRMA	168.96	9.333	99.847	0.13	0.128	0.486	474.0	5.0	145534.0	4871.0	110982.0	29106.8
LAZYPIPE	4.515	4.0	99.995	0.039	0.032	1.51	210.667	22.0	209468.0	262.0	48758.0	9521.3
metaSPAdes	3.8	7.0	99.992	0.033	0.026	0.227	394.333	20.0	210228.0	341.0	49969.0	10511.4
metaviralSPAdes	–	–	–	–	–	–	–	–	–	–	–	–
PEHaplo	16.915	4.0	99.615	0.056	0.046	0.424	101.667	192.0	229893.0	382.0	4797.0	1197.4
QuRe	1250.83	0.0	100.0	1.0	0.998	53.058	508.333	2.0	256.0	128.0	128.0	128.0
QVG	27.45	7540.0	94.73	0.246	0.241	1.4	133.333	7.0	279890.0	5387.0	125044.0	39984.3
SPAdes	5.92	5.0	99.993	0.033	0.026	0.159	316.333	18.0	210085.0	2166.0	49969.0	11671.4
SSAKE	1.7	0.667	99.996	0.928	0.916	0.05	100.0	113.667	20939.333	102.0	451.667	184.233
TRACESPipe	175.74	758.0	99.217	0.08	0.07	9.768	357.0	4.0	143860.0	5387.0	125041.0	35965.0
TRACESPipeLite	39.02	0.0	100.0	0.999	0.997	2.824	351.667	6.0	168262.0	5387.0	125041.0	28043.7
VirGenA	544.415	74.667	99.727	0.663	0.645	4.466	737.0	73.0	55130.0	105.667	2842.667	772.867
ViSpA	76.595	915.333	97.045	0.168	0.158	6.355	85.667	88.0	3718653.0	412.0	113601.0	42257.4
V-pipe	38.6	78.0	94.147	0.357	0.334	0.104	114.667	5.0	160429.0	5387.0	125041.0	32085.8

Table S43: Results obtained for DS39 using the benchmark proposed. The execution time was measured in seconds, the RAM usage was measured in GB and the CPU usage is presented as a percentage. The executions were, when possible, capped at 6 threads and 48 GB of RAM.

Reconstruction tool	Execution time	SNPs	Identity	NCSD	NRC	RAM usage	CPU usage	Number of scaffolds	Reconstructed bases	Minimum scaffold length	Maximum scaffold length	Average scaffold length
coronaSPAdes	2.095	4.0	99.997	0.033	0.026	0.134	413.333	26.0	209877.0	392.0	28454.0	8072.2
Haploflow	2.06	0.0	100.0	0.631	0.613	0.069	99.667	112.0	87072.0	508.0	1820.0	777.4
IRMA	205.48	49.667	99.813	0.134	0.126	0.497	475.0	5.0	146048.0	4892.0	111889.0	29209.6
LAZYPIPE	4.105	3.0	99.998	0.043	0.035	1.509	197.333	27.0	208705.0	362.0	28385.0	7729.8
metaSPAdes	3.71	2.0	99.998	0.033	0.026	0.228	394.333	26.0	209877.0	392.0	28454.0	8072.2
metaviralSPAdes	–	–	–	–	–	–	–	–	–	–	–	–
PEHaplo	16.795	3.0	99.997	0.049	0.04	0.423	101.667	207.0	233449.0	385.0	5356.0	1127.8
QuRe	156.305	0.0	100.0	0.999	0.997	29.787	567.667	3.333	570.667	152.0	200.0	170.667
QVG	26.205	11690.0	91.79	0.395	0.387	1.399	135.667	7.0	279880.0	5385.0	125041.0	39982.9
SPAdes	7.28	2.0	99.998	0.033	0.026	0.158	278.0	26.0	209877.0	392.0	28454.0	8072.2
SSAKE	1.8	1.333	99.992	0.917	0.903	0.05	100.0	118.0	22427.0	104.333	426.333	190.067
TRACESPipe	195.91	345.0	98.7	0.093	0.08	9.768	329.667	4.0	143860.0	5387.0	125041.0	35965.0
TRACESPipeLite	36.545	0.0	0.0	1.0	0.998	2.824	365.333	5.0	160429.0	5387.0	125041.0	32085.8
VirGenA	554.24	59.667	99.8	0.626	0.608	4.352	733.333	80.667	60899.333	113.333	2620.0	762.467
ViSpA	80.615	489.667	95.815	0.361	0.351	6.056	85.333	139.0	5120204.0	464.0	91748.0	36836.0
V-pipe	38.49	457.0	93.037	0.478	0.453	0.104	114.667	5.0	160429.0	5387.0	125041.0	32085.8

Table S44: Results obtained for DS40 using the benchmark proposed. The execution time was measured in seconds, the RAM usage was measured in GB and the CPU usage is presented as a percentage. The executions were, when possible, capped at 6 threads and 48 GB of RAM.

Reconstruction tool	Execution time	SNPs	Identity	NCSD	NRC	RAM usage	CPU usage	Number of scaffolds	Reconstructed bases	Minimum scaffold length	Maximum scaffold length	Average scaffold length
coronaSPAdes	2.09	12.0	99.991	0.033	0.025	0.134	415.667	19.0	209941.0	747.0	43152.0	11049.5
Haploflow	2.08	0.0	100.0	0.676	0.657	0.07	99.667	100.0	75548.0	502.0	1689.0	755.5
IRMA	223.015	36.667	99.801	0.144	0.131	0.506	475.0	5.0	145808.0	4892.0	111955.0	29161.6
LAZYPIPE	4.055	4.0	99.996	0.04	0.032	1.509	194.667	19.0	209013.0	740.0	43095.0	11000.7
metaSPAdes	3.83	7.0	99.993	0.033	0.025	0.227	396.0	19.0	209941.0	747.0	43152.0	11049.5
metaviralSPAdes	–	–	–	–	–	–	–	–	–	–	–	–
PEHaplo	16.88	3.0	99.997	0.044	0.037	0.42	101.333	201.0	238048.0	378.0	5518.0	1184.3
QuRe	169.19	0.0	100.0	1.0	0.999	27.323	588.333	1.0	164.0	164.0	164.0	164.0
QVG	24.015	15561.0	89.044	0.521	0.516	1.398	134.333	6.0	274495.0	5387.0	125041.0	45749.2
SPAdes	6.76	5.0	99.996	0.032	0.025	0.158	290.333	19.0	209941.0	747.0	43152.0	11049.5
SSAKE	1.745	0.667	99.996	0.927	0.913	0.05	100.0	117.667	21592.0	103.333	377.667	183.5
TRACESPipe	194.315	1647.0	98.52	0.145	0.134	9.768	322.0	4.0	143860.0	5387.0	125041.0	35965.0
TRACESPipeLite	36.535	0.0	0.0	1.0	0.999	2.825	366.0	5.0	160429.0	5387.0	125041.0	32085.8
VirGenA	529.36	72.0	99.819	0.614	0.596	4.517	732.0	82.333	62748.333	155.0	2665.667	773.8
ViSpA	53.24	228.0	95.858	0.586	0.576	5.694	79.0	115.0	3300714.0	411.0	63874.0	28701.9
V-pipe	38.25	212.0	91.872	0.621	0.596	0.104	115.0	5.0	160429.0	5387.0	125041.0	32085.8

Table S45: Results obtained for DS41 using the benchmark proposed. The execution time was measured in seconds, the RAM usage was measured in GB and the CPU usage is presented as a percentage. The executions were, when possible, capped at 6 threads and 48 GB of RAM.

Reconstruction tool	Execution time	SNPs	Identity	NCSD	NRC	RAM usage	CPU usage	Number of scaffolds	Reconstructed bases	Minimum scaffold length	Maximum scaffold length	Average scaffold length
coronaSPAdes	3.135	1.0	99.9	0.03	0.022	0.142	410.0	9.0	202464.0	127.0	117531.0	22496.0
Haploflow	3.565	13.0	99.976	0.061	0.048	0.1	99.0	53.0	201565.0	556.0	11370.0	3803.1
IRMA	167.785	0.0	100.0	0.088	0.128	0.879	537.0	5.0	144829.0	4887.0	110250.0	28965.8
LAZYPIPE	7.115	0.0	99.971	0.03	0.022	1.525	254.0	7.0	202223.0	5322.0	96482.0	28889.0
metaSPAdes	5.59	0.0	99.952	0.029	0.021	0.229	410.667	9.0	202467.0	71.0	105065.0	22496.3
metaviralSPAdes	–	–	–	–	–	–	–	–	–	–	–	–
PEHaplo	31.735	0.0	100.0	0.073	0.061	1.162	133.333	312.0	252737.0	375.0	2818.0	810.1
QuRe	438.51	0.0	100.0	0.998	0.992	19.134	566.667	4.333	764.333	124.0	271.0	179.333
QVG	–	–	–	–	–	–	–	–	–	–	–	–
SPAdes	9.97	0.0	99.917	0.029	0.021	0.161	285.667	8.0	202590.0	401.0	112460.0	25323.8
SSAKE	5.595	3.667	99.977	0.475	0.431	0.08	100.0	371.333	121382.0	102.0	2740.0	326.9
TRACESPipe	174.335	0.0	100.0	0.029	0.021	9.768	359.333	4.0	143860.0	5387.0	125041.0	35965.0
TRACESPipeLite	44.705	1.0	99.991	0.028	0.02	2.819	319.333	6.0	168262.0	5387.0	125041.0	28043.7
VirGenA	–	–	–	–	–	–	–	–	–	–	–	–
ViSpA	154.7	0.0	100.0	0.029	0.021	5.972	112.333	5.0	160119.0	5322.0	124995.0	32023.8
V-pipe	45.975	0.0	99.837	0.042	0.032	0.104	114.333	5.0	160429.0	5387.0	125041.0	32085.8

Table S46: Results obtained for DS42 using the benchmark proposed. The execution time was measured in seconds, the RAM usage was measured in GB and the CPU usage is presented as a percentage. The executions were, when possible, capped at 6 threads and 48 GB of RAM.

Reconstruction tool	Execution time	SNPs	Identity	NCSD	NRC	RAM usage	CPU usage	Number of scaffolds	Reconstructed bases	Minimum scaffold length	Maximum scaffold length	Average scaffold length
coronaSPAdes	3.07	7.0	99.995	0.036	0.026	0.165	418.667	13.0	207930.0	262.0	105627.0	15994.6
Haploflow	3.58	142.0	99.844	0.052	0.04	0.1	99.0	57.0	206813.0	507.0	16242.0	3628.3
IRMA	195.99	0.0	100.0	0.104	0.128	0.877	540.0	5.0	144804.0	4868.0	110235.0	28960.8
LAZYPIPE	6.86	291.0	99.786	0.032	0.022	1.525	267.0	10.0	210000.0	392.0	97589.0	21000.0
metaSPAdes	5.62	84.0	99.944	0.035	0.024	0.229	412.0	7.0	208833.0	1027.0	122573.0	29833.3
metaviralSPAdes	–	–	–	–	–	–	–	–	–	–	–	–
PEHaplo	31.74	108.0	99.888	0.084	0.068	1.142	132.333	325.0	263012.0	375.0	2428.0	809.3
QuRe	939.94	16.667	99.476	0.989	0.977	42.47	546.667	6.0	4321.0	118.667	1437.0	707.933
QVG	27.39	551.0	99.537	0.046	0.037	1.409	156.333	5.0	268901.0	5387.0	125041.0	53780.2
SPAdes	6.025	2.0	99.999	0.031	0.021	0.161	399.333	7.0	210263.0	5370.0	107709.0	30037.6
SSAKE	5.59	6.667	99.985	0.537	0.506	0.079	100.0	359.0	113578.333	101.333	1099.0	316.433
TRACESPipe	170.04	438.0	99.663	0.039	0.029	9.768	360.667	4.0	143860.0	5387.0	125041.0	35965.0
TRACESPipeLite	46.425	457.333	99.353	0.06	0.048	2.821	328.333	6.0	168262.0	5387.0	125041.0	28043.7
VirGenA	830.625	127.667	99.713	0.65	0.623	4.427	743.0	55.333	54882.333	250.333	3061.0	1010.967
ViSpA	148.715	444.0	99.676	0.043	0.032	6.12	110.0	5.0	160099.0	5336.0	124985.0	32019.8
V-pipe	47.45	362.0	99.398	0.051	0.04	0.104	112.667	5.0	160429.0	5387.0	125041.0	32085.8

Table S47: Results obtained for DS43 using the benchmark proposed. The execution time was measured in seconds, the RAM usage was measured in GB and the CPU usage is presented as a percentage. The executions were, when possible, capped at 6 threads and 48 GB of RAM.

Reconstruction tool	Execution time	SNPs	Identity	NCSD	NRC	RAM usage	CPU usage	Number of scaffolds	Reconstructed bases	Minimum scaffold length	Maximum scaffold length	Average scaffold length
coronaSPAdes	3.1	2.0	99.991	0.031	0.022	0.142	408.667	6.0	210231.0	5328.0	124992.0	35038.5
Haploflow	3.56	616.0	99.537	0.058	0.045	0.1	99.0	44.0	206813.0	600.0	15451.0	4700.3
IRMA	262.66	0.0	99.999	0.109	0.128	0.88	537.667	5.0	144812.0	4878.0	110278.0	28962.4
LAZYPIPE	6.155	6.0	99.981	0.032	0.022	1.525	254.0	8.0	210138.0	351.0	109533.0	26267.3
metaSPAdes	5.63	2.0	99.998	0.031	0.022	0.229	413.0	6.0	210231.0	5328.0	124992.0	35038.5
metaviralSPAdes	–	–	–	–	–	–	–	–	–	–	–	–
PEHaplo	31.79	145.0	99.839	0.075	0.062	1.144	133.667	358.0	273251.0	375.0	3051.0	763.3
QuRe	410.405	1.333	99.85	0.995	0.988	16.62	562.0	7.0	1825.333	105.0	531.0	262.167
QVG	28.615	812.0	99.432	0.053	0.043	1.409	154.333	5.0	268901.0	5387.0	125041.0	53780.2
SPAdes	6.31	0.0	100.0	0.031	0.022	0.162	389.0	6.0	210231.0	5328.0	124992.0	35038.5
SSAKE	5.385	7.333	99.988	0.522	0.493	0.079	100.0	377.667	119055.667	102.667	1108.333	315.333
TRACESPipe	176.49	741.0	99.481	0.041	0.031	9.768	360.333	4.0	143864.0	5387.0	125045.0	35966.0
TRACESPipeLite	44.145	271.333	97.621	0.315	0.287	2.824	329.0	6.0	168264.0	5387.0	125043.0	28044.0
VirGenA	729.97	93.0	99.788	0.666	0.642	4.277	736.5	68.0	54227.0	65.0	2055.0	797.5
ViSpA	149.68	801.0	99.41	0.049	0.038	6.123	111.667	5.0	160128.0	5317.0	124991.0	32025.6
V-pipe	47.43	693.0	99.348	0.055	0.045	0.104	113.667	5.0	160429.0	5387.0	125041.0	32085.8

Table S48: Results obtained for DS44 using the benchmark proposed. The execution time was measured in seconds, the RAM usage was measured in GB and the CPU usage is presented as a percentage. The executions were, when possible, capped at 6 threads and 48 GB of RAM.

Reconstruction tool	Execution time	SNPs	Identity	NCSD	NRC	RAM usage	CPU usage	Number of scaffolds	Reconstructed bases	Minimum scaffold length	Maximum scaffold length	Average scaffold length
coronaSPAdes	2.89	0.0	100.0	0.03	0.021	0.144	431.0	6.0	210201.0	5363.0	125004.0	35033.5
Haploflow	3.56	69.0	99.933	0.058	0.047	0.1	99.0	61.0	206286.0	534.0	11587.0	3381.7
IRMA	269.055	0.0	100.0	0.114	0.127	0.881	551.0	5.0	145000.0	4908.0	110436.0	29000.0
LAZYPIPE	5.315	0.0	99.983	0.031	0.022	1.525	235.333	6.0	210024.0	5318.0	124924.0	35004.0
metaSPAdes	5.54	0.0	100.0	0.03	0.021	0.23	412.667	6.0	210201.0	5363.0	125004.0	35033.5
metaviralSPAdes	–	–	–	–	–	–	–	–	–	–	–	–
PEHaplo	31.285	44.0	99.949	0.078	0.065	1.147	133.667	328.0	268990.0	377.0	2897.0	820.1
QuRe	436.055	1.0	99.766	0.999	0.994	19.368	579.0	5.0	893.333	141.667	220.333	178.7
QVG	32.375	1293.0	99.056	0.068	0.058	1.409	155.333	6.0	274497.0	5387.0	125041.0	45749.5
SPAdes	6.33	0.0	100.0	0.03	0.021	0.162	389.333	6.0	210201.0	5363.0	125004.0	35033.5
SSAKE	5.815	3.0	99.994	0.506	0.48	0.08	100.0	377.667	119891.0	101.0	1146.0	317.433
TRACESPipe	189.22	893.0	99.294	0.046	0.036	9.768	338.667	4.0	143863.0	5387.0	125043.0	35965.8
TRACESPipeLite	41.835	33.333	98.268	0.801	0.781	2.825	337.0	6.0	168262.0	5387.0	125041.0	28043.7
VirGenA	781.02	87.0	99.827	0.649	0.631	4.476	737.667	63.0	56079.0	80.0	2749.0	890.1
ViSpA	72.62	1069.0	99.064	0.057	0.047	6.0	111.333	10.0	304898.0	4090.0	124891.0	30489.8
V-pipe	47.4	840.0	99.045	0.065	0.055	0.104	113.333	5.0	160429.0	5387.0	125041.0	32085.8

Table S49: Results obtained for DS45 using the benchmark proposed. The execution time was measured in seconds, the RAM usage was measured in GB and the CPU usage is presented as a percentage. The executions were, when possible, capped at 6 threads and 48 GB of RAM.

Reconstruction tool	Execution time	SNPs	Identity	NCSD	NRC	RAM usage	CPU usage	Number of scaffolds	Reconstructed bases	Minimum scaffold length	Maximum scaffold length	Average scaffold length
coronaSPAdes	2.9	1.0	99.991	0.029	0.022	0.142	428.667	6.0	210140.0	5364.0	124966.0	35023.3
Haploflow	3.57	0.0	100.0	0.059	0.048	0.1	99.333	45.0	206927.0	647.0	19632.0	4598.4
IRMA	316.405	8.333	99.994	0.115	0.123	0.888	560.0	5.0	145734.333	4894.0	111130.333	29146.867
LAZYPIPE	5.325	0.0	100.0	0.03	0.023	1.525	236.667	6.0	210022.0	5316.0	124919.0	35003.7
metaSPAdes	5.565	1.0	99.991	0.029	0.022	0.229	412.333	6.0	210147.0	5364.0	124966.0	35024.5
metaviralSPAdes	–	–	–	–	–	–	–	–	–	–	–	–
PEHaplo	31.145	39.0	99.935	0.077	0.064	1.134	132.333	317.0	255235.0	377.0	3917.0	805.2
QuRe	701.145	1.667	99.872	0.995	0.99	17.776	596.333	5.0	1501.333	139.0	487.0	303.367
QVG	35.155	1950.0	98.633	0.085	0.078	1.409	156.667	7.0	279882.0	5387.0	125040.0	39983.1
SPAdes	6.86	1.0	99.991	0.029	0.022	0.161	368.0	6.0	210147.0	5364.0	124966.0	35024.5
SSAKE	5.52	4.0	99.993	0.536	0.514	0.079	100.0	364.333	113342.667	102.667	1526.333	311.067
TRACESPipe	181.755	1516.0	98.594	0.088	0.077	9.768	357.0	4.0	143847.0	5387.0	125052.0	35961.8
TRACESPipeLite	41.105	2.0	99.509	0.971	0.962	2.827	340.667	6.0	168262.0	5387.0	125041.0	28043.7
VirGenA	–	–	–	–	–	–	–	–	–	–	–	–
ViSpA	65.22	1287.667	98.429	0.074	0.063	5.994	113.667	5.0	1501657.0	5314.0	1460372.0	300331.4
V-pipe	47.365	908.0	98.817	0.066	0.056	0.104	114.0	5.0	160429.0	5387.0	125041.0	32085.8

Table S50: Results obtained for DS46 using the benchmark proposed. The execution time was measured in seconds, the RAM usage was measured in GB and the CPU usage is presented as a percentage. The executions were, when possible, capped at 6 threads and 48 GB of RAM.

Reconstruction tool	Execution time	SNPs	Identity	NCSD	NRC	RAM usage	CPU usage	Number of scaffolds	Reconstructed bases	Minimum scaffold length	Maximum scaffold length	Average scaffold length
coronaSPAdes	2.88	3.0	99.99	0.028	0.021	0.142	430.333	6.0	210135.0	5381.0	124977.0	35022.5
Haploflow	3.58	0.0	100.0	0.057	0.046	0.1	99.667	49.0	206675.0	560.0	24401.0	4217.9
IRMA	275.825	3.333	99.989	0.122	0.12	0.892	554.333	5.0	146285.0	4890.0	111769.0	29257.0
LAZYPIPE	4.945	0.0	100.0	0.029	0.022	1.526	224.667	6.0	209964.0	5368.0	124939.0	34994.0
metaSPAdes	5.545	0.0	100.0	0.028	0.021	0.229	411.0	6.0	210135.0	5381.0	124977.0	35022.5
metaviralSPAdes	–	–	–	–	–	–	–	–	–	–	–	–
PEHaplo	31.31	33.0	99.944	0.07	0.058	1.17	132.333	299.0	245592.0	375.0	2712.0	821.4
QuRe	488.97	0.333	99.971	0.997	0.993	17.356	564.667	8.333	1372.333	73.0	259.0	164.733
QVG	34.725	3754.0	97.368	0.139	0.134	1.409	153.333	6.0	274272.0	5387.0	125033.0	45712.0
SPAdes	7.055	0.0	100.0	0.028	0.021	0.161	362.667	6.0	210135.0	5381.0	124977.0	35022.5
SSAKE	5.49	4.0	99.994	0.524	0.502	0.079	100.0	357.0	115824.333	102.0	1312.333	324.5
TRACESPipe	189.475	239.0	99.809	0.039	0.031	9.768	346.0	4.0	143831.0	5358.0	125041.0	35957.8
TRACESPipeLite	40.565	0.0	100.0	1.0	0.997	2.827	340.333	6.0	168262.0	5387.0	125041.0	28043.7
VirGenA	736.52	30.0	99.694	0.699	0.682	4.071	740.667	56.0	48874.0	116.0	2225.0	872.8
ViSpA	91.885	1196.333	97.355	0.121	0.109	5.956	106.0	69.0	3266869.0	403.0	118958.0	47345.9
V-pipe	47.595	1120.0	97.644	0.123	0.108	0.104	113.333	5.0	160429.0	5387.0	125041.0	32085.8

Table S51: Results obtained for DS47 using the benchmark proposed. The execution time was measured in seconds, the RAM usage was measured in GB and the CPU usage is presented as a percentage. The executions were, when possible, capped at 6 threads and 48 GB of RAM.

Reconstruction tool	Execution time	SNPs	Identity	NCSD	NRC	RAM usage	CPU usage	Number of scaffolds	Reconstructed bases	Minimum scaffold length	Maximum scaffold length	Average scaffold length
coronaSPAdes	2.93	0.0	100.0	0.027	0.02	0.145	429.333	6.0	210293.0	5374.0	124987.0	35048.8
Haploflow	3.605	0.0	100.0	0.047	0.039	0.101	99.333	61.0	208117.0	504.0	12951.0	3411.8
IRMA	303.18	11.333	99.99	0.128	0.122	0.898	553.0	5.0	146154.667	4886.0	111820.667	29230.933
LAZYPIPE	6.365	2.0	99.997	0.033	0.026	1.525	269.0	24.0	210020.0	589.0	39288.0	8750.8
metaSPAdes	5.56	1.0	99.991	0.027	0.021	0.229	410.333	6.0	210293.0	5374.0	124987.0	35048.8
metaviralSPAdes	–	–	–	–	–	–	–	–	–	–	–	–
PEHaplo	31.27	0.0	100.0	0.072	0.061	1.155	131.667	310.0	252286.0	377.0	2966.0	813.8
QuRe	372.765	3.0	99.751	0.997	0.993	16.72	559.333	4.333	1143.333	192.0	396.333	262.8
QVG	35.275	6950.0	94.724	0.256	0.252	1.409	155.333	7.0	282112.0	5387.0	125033.0	40301.7
SPAdes	6.135	1.0	99.991	0.027	0.021	0.161	397.333	6.0	210293.0	5374.0	124987.0	35048.8
SSAKE	5.145	3.667	99.992	0.533	0.512	0.08	100.0	360.0	115435.0	102.0	1227.667	320.667
TRACESPipe	202.665	3.0	99.991	0.028	0.022	9.768	326.0	4.0	143701.0	5298.0	125007.0	35925.3
TRACESPipeLite	37.64	0.0	0.0	1.0	0.998	2.828	353.333	5.0	160429.0	5387.0	125041.0	32085.8
VirGenA	764.02	96.0	99.753	0.686	0.67	4.195	737.333	57.0	51406.0	94.0	2356.0	901.9
ViSpA	115.685	1109.0	96.25	0.283	0.273	6.334	99.0	162.0	6569618.0	500.0	101012.0	40553.2
V-pipe	47.425	1569.0	95.842	0.23	0.21	0.104	113.333	5.0	160429.0	5387.0	125041.0	32085.8

Table S52: Results obtained for DS48 using the benchmark proposed. The execution time was measured in seconds, the RAM usage was measured in GB and the CPU usage is presented as a percentage. The executions were, when possible, capped at 6 threads and 48 GB of RAM.

Reconstruction tool	Execution time	SNPs	Identity	NCSD	NRC	RAM usage	CPU usage	Number of scaffolds	Reconstructed bases	Minimum scaffold length	Maximum scaffold length	Average scaffold length
coronaSPAdes	3.005	0.0	100.0	0.027	0.021	0.146	421.333	6.0	210218.0	5341.0	125035.0	35036.3
Haploflow	3.58	0.0	100.0	0.046	0.038	0.1	99.667	50.0	208209.0	554.0	15846.0	4164.2
IRMA	341.925	55.667	99.956	0.128	0.117	0.919	555.333	5.0	147253.333	5033.0	112899.333	29450.667
LAZYPIPE	4.59	0.0	100.0	0.029	0.023	1.525	210.0	6.0	209997.0	5291.0	124999.0	34999.5
metaSPAdes	5.595	0.0	100.0	0.027	0.021	0.229	410.667	6.0	210218.0	5341.0	125035.0	35036.3
metaviralSPAdes	–	–	–	–	–	–	–	–	–	–	–	–
PEHaplo	31.275	9.0	99.986	0.09	0.076	1.157	131.667	305.0	247198.0	377.0	3432.0	810.5
QuRe	725.785	8.667	99.666	0.99	0.984	27.461	599.667	7.333	2964.0	109.333	1368.0	399.9
QVG	34.345	11569.0	91.836	0.398	0.395	1.409	160.0	8.0	287720.0	5387.0	125041.0	35965.0
SPAdes	5.935	0.0	100.0	0.027	0.021	0.161	405.667	6.0	210218.0	5341.0	125035.0	35036.3
SSAKE	5.795	3.333	99.995	0.499	0.476	0.081	100.0	384.667	124712.333	102.667	1256.0	324.133
TRACESPipe	202.775	52.0	99.851	0.039	0.032	9.768	323.333	4.0	143811.0	5361.0	125041.0	35952.8
TRACESPipeLite	37.35	0.0	0.0	1.0	0.999	2.828	354.0	5.0	160429.0	5387.0	125041.0	32085.8
VirGenA	790.66	10.0	99.761	0.734	0.717	3.95	744.0	60.0	44696.0	187.0	2269.0	744.9
ViSpA	89.27	514.667	95.995	0.482	0.473	5.942	97.333	168.0	5743888.0	433.0	77451.0	34189.8
V-pipe	47.02	566.0	94.245	0.386	0.364	0.104	113.333	5.0	160429.0	5387.0	125041.0	32085.8

Table S53: Results obtained for DS49 using the benchmark proposed. The execution time was measured in seconds, the RAM usage was measured in GB and the CPU usage is presented as a percentage. The executions were, when possible, capped at 6 threads and 48 GB of RAM.

Reconstruction tool	Execution time	SNPs	Identity	NCSD	NRC	RAM usage	CPU usage	Number of scaffolds	Reconstructed bases	Minimum scaffold length	Maximum scaffold length	Average scaffold length
coronaSPAdes	4.665	0.0	99.949	0.029	0.022	0.149	438.333	9.0	202617.0	147.0	112509.0	22513.0
Haploflow	6.5	8.0	99.989	0.03	0.022	0.151	99.0	14.0	203336.0	1385.0	54636.0	14524.0
IRMA	307.225	0.0	100.0	0.088	0.127	1.648	597.333	5.0	144862.0	4876.0	110239.0	28972.4
LAZYPIPE	9.515	0.0	99.994	0.028	0.021	1.541	269.0	8.0	202542.0	5373.0	97853.0	25317.8
metaSPAdes	8.75	15.0	99.8	0.029	0.021	0.226	429.667	10.0	202605.0	71.0	112462.0	20260.5
metaviralSPAdes	9.955	0.0	0.0	1.0	0.994	0.227	450.333	2.0	66537.0	16548.0	49989.0	33268.5
PEHaplo	83.755	0.0	99.999	0.031	0.024	3.052	142.0	215.0	311054.0	382.0	6699.0	1446.8
QuRe	3261.255	0.333	99.996	0.946	0.905	21.594	627.333	5.0	10109.0	312.0	6698.333	2021.8
QVG	–	–	–	–	–	–	–	–	–	–	–	–
SPAdes	10.155	0.0	99.9	0.029	0.02	0.185	393.0	9.0	202724.0	401.0	105060.0	22524.9
SSAKE	16.44	5.333	99.992	0.041	0.032	0.132	99.667	170.0	216031.667	103.0	11406.667	1271.9
TRACESPipe	188.11	0.0	100.0	0.028	0.02	9.768	360.667	4.0	143860.0	5387.0	125041.0	35965.0
TRACESPipeLite	52.26	0.0	100.0	0.028	0.02	2.826	286.333	6.0	168262.0	5387.0	125041.0	28043.7
VirGenA	1120.54	9.0	99.932	0.673	0.64	4.812	744.667	62.0	52819.333	68.0	2550.333	852.467
ViSpA	483.085	0.0	100.0	0.028	0.02	7.009	133.0	5.0	160254.0	5373.0	125010.0	32050.8
V-pipe	62.42	0.0	100.0	0.029	0.022	0.104	113.333	5.0	160429.0	5387.0	125041.0	32085.8

Table S54: Results obtained for DS50 using the benchmark proposed. The execution time was measured in seconds, the RAM usage was measured in GB and the CPU usage is presented as a percentage. The executions were, when possible, capped at 6 threads and 48 GB of RAM.

Reconstruction tool	Execution time	SNPs	Identity	NCSD	NRC	RAM usage	CPU usage	Number of scaffolds	Reconstructed bases	Minimum scaffold length	Maximum scaffold length	Average scaffold length
coronaSPAdes	4.65	6.0	99.996	0.036	0.025	0.171	445.667	12.0	207942.0	530.0	105623.0	17328.5
Haploflow	6.57	315.0	99.704	0.034	0.023	0.152	99.333	11.0	209651.0	1817.0	88819.0	19059.2
IRMA	363.795	0.0	100.0	0.103	0.127	1.646	598.667	5.0	144911.0	4884.0	110274.0	28982.2
LAZYPIPE	9.715	1.0	99.963	0.031	0.022	1.542	273.333	8.0	210172.0	5362.0	105627.0	26271.5
metaSPAdes	8.655	78.0	99.947	0.033	0.023	0.226	431.333	6.0	210344.0	5369.0	125025.0	35057.3
metaviralSPAdes	9.85	0.0	100.0	0.163	0.149	0.227	452.333	3.0	191573.0	16560.0	125025.0	63857.7
PEHaplo	79.955	114.0	99.917	0.036	0.027	2.966	145.0	243.0	344843.0	387.0	4514.0	1419.1
QuRe	4168.455	130.333	98.838	0.954	0.922	20.104	636.0	5.333	16396.333	627.333	5111.0	2704.367
QVG	39.435	498.0	99.577	0.041	0.034	1.419	175.333	5.0	268901.0	5387.0	125041.0	53780.2
SPAdes	9.78	1.0	99.999	0.031	0.021	0.186	415.333	6.0	210358.0	5369.0	125025.0	35059.7
SSAKE	16.61	34.667	99.943	0.042	0.031	0.132	100.0	167.667	222211.0	105.333	10487.0	1325.567
TRACESPipe	189.17	404.0	99.719	0.038	0.028	9.768	362.0	4.0	143861.0	5387.0	125042.0	35965.3
TRACESPipeLite	53.82	473.333	99.528	0.053	0.042	2.828	304.0	6.0	168261.0	5387.0	125040.0	28043.5
VirGenA	1118.28	136.0	99.536	0.728	0.696	4.464	748.667	42.333	44162.0	239.667	3002.667	1056.5
ViSpA	454.14	551.0	99.569	0.041	0.031	6.934	130.0	5.0	160260.0	5362.0	125030.0	32052.0
V-pipe	64.33	476.0	99.664	0.041	0.031	0.104	113.0	5.0	160429.0	5387.0	125041.0	32085.8

Table S55: Results obtained for DS51 using the benchmark proposed. The execution time was measured in seconds, the RAM usage was measured in GB and the CPU usage is presented as a percentage. The executions were, when possible, capped at 6 threads and 48 GB of RAM.

Reconstruction tool	Execution time	SNPs	Identity	NCSD	NRC	RAM usage	CPU usage	Number of scaffolds	Reconstructed bases	Minimum scaffold length	Maximum scaffold length	Average scaffold length
coronaSPAdes	4.555	2.0	99.999	0.03	0.021	0.153	436.333	6.0	210359.0	5371.0	125029.0	35059.8
Haploflow	6.505	245.0	99.792	0.033	0.024	0.15	99.667	11.0	216210.0	2101.0	110024.0	19655.5
IRMA	366.775	0.0	100.0	0.108	0.128	1.651	597.333	5.0	144931.0	4892.0	110278.0	28986.2
LAZYPIPE	9.62	1.0	99.478	0.03	0.021	1.541	271.0	8.0	210589.0	393.0	109777.0	26323.6
metaSPAdes	8.5	1.0	99.999	0.03	0.021	0.227	431.0	6.0	210359.0	5371.0	125029.0	35059.8
metaviralSPAdes	9.73	0.0	100.0	0.162	0.149	0.227	452.667	3.0	191575.0	16567.0	125029.0	63858.3
PEHaplo	76.86	0.0	99.944	0.046	0.035	2.917	146.0	242.0	324762.0	377.0	4784.0	1342.0
QuRe	2744.455	4.333	99.567	0.961	0.951	19.926	640.667	4.667	8286.0	543.333	3248.0	1778.933
QVG	42.745	741.0	99.488	0.047	0.04	1.418	174.333	5.0	268901.0	5387.0	125041.0	53780.2
SPAdes	9.16	1.0	99.999	0.03	0.021	0.187	423.667	6.0	210359.0	5371.0	125029.0	35059.8
SSAKE	16.565	0.333	99.999	0.041	0.031	0.133	100.0	170.667	222789.0	113.667	10299.0	1310.0
TRACESPipe	199.645	609.0	99.579	0.042	0.033	9.768	347.0	4.0	143862.0	5388.0	125041.0	35965.5
TRACESPipeLite	49.92	304.0	97.736	0.305	0.275	2.829	307.667	6.0	156261.0	5387.0	113040.0	26043.5
VirGenA	1071.385	62.0	99.659	0.694	0.674	4.739	747.667	47.0	48702.0	86.0	3067.0	1036.2
ViSpA	420.03	638.0	99.522	0.05	0.039	6.923	134.667	5.0	160284.0	5344.0	125026.0	32056.8
V-pipe	65.21	590.0	99.591	0.042	0.033	0.104	112.333	5.0	160429.0	5387.0	125041.0	32085.8

Table S56: Results obtained for DS52 using the benchmark proposed. The execution time was measured in seconds, the RAM usage was measured in GB and the CPU usage is presented as a percentage. The executions were, when possible, capped at 6 threads and 48 GB of RAM.

Reconstruction tool	Execution time	SNPs	Identity	NCSD	NRC	RAM usage	CPU usage	Number of scaffolds	Reconstructed bases	Minimum scaffold length	Maximum scaffold length	Average scaffold length
coronaSPAdes	4.485	0.0	100.0	0.029	0.021	0.156	450.333	6.0	210345.0	5354.0	125021.0	35057.5
Haploflow	6.705	499.0	99.371	0.033	0.024	0.158	99.0	9.0	209870.0	4765.0	94245.0	23318.9
IRMA	502.225	0.333	100.0	0.114	0.127	1.648	609.333	5.0	144956.0	4910.0	110307.0	28991.2
LAZYPIPE	7.88	0.0	99.985	0.029	0.021	1.54	244.0	6.0	210232.0	5332.0	124944.0	35038.7
metaSPAdes	8.51	0.0	100.0	0.029	0.021	0.226	431.333	6.0	210346.0	5355.0	125021.0	35057.7
metaviralSPAdes	9.695	0.0	100.0	0.161	0.149	0.227	452.0	3.0	191576.0	16565.0	125021.0	63858.7
PEHaplo	77.47	18.0	99.983	0.036	0.028	2.949	145.667	217.0	302159.0	393.0	5663.0	1392.4
QuRe	2009.37	7.333	99.882	0.966	0.958	21.751	638.667	5.0	7871.667	755.667	2247.333	1574.333
QVG	50.08	1058.0	99.221	0.056	0.049	1.418	177.667	7.0	282333.0	5387.0	125041.0	40333.3
SPAdes	9.075	0.0	100.0	0.029	0.021	0.186	424.333	6.0	210346.0	5355.0	125021.0	35057.7
SSAKE	16.49	0.0	99.998	0.038	0.028	0.134	99.667	157.333	222399.333	108.667	10223.333	1417.333
TRACESPipe	202.6	6.0	99.986	0.029	0.021	9.768	342.667	4.0	143860.0	5387.0	125041.0	35965.0
TRACESPipeLite	44.915	94.667	98.341	0.76	0.737	2.83	316.333	6.0	168263.0	5387.0	125042.0	28043.8
VirGenA	1129.92	100.0	99.732	0.716	0.697	4.641	749.0	43.333	45948.667	337.667	2685.0	1077.833
ViSpA	212.14	1024.333	99.067	0.059	0.051	7.054	133.333	11.0	492337.0	4092.0	125027.0	44757.9
V-pipe	65.87	556.0	99.201	0.048	0.039	0.104	111.667	5.0	160429.0	5387.0	125041.0	32085.8

Table S57: Results obtained for DS53 using the benchmark proposed. The execution time was measured in seconds, the RAM usage was measured in GB and the CPU usage is presented as a percentage. The executions were, when possible, capped at 6 threads and 48 GB of RAM.

Reconstruction tool	Execution time	SNPs	Identity	NCSD	NRC	RAM usage	CPU usage	Number of scaffolds	Reconstructed bases	Minimum scaffold length	Maximum scaffold length	Average scaffold length
coronaSPAdes	4.51	1.0	99.999	0.028	0.021	0.153	449.333	6.0	210316.0	5363.0	125016.0	35052.7
Haploflow	6.56	0.0	99.862	0.031	0.024	0.152	99.333	8.0	211036.0	5267.0	106254.0	26379.5
IRMA	509.245	7.333	99.976	0.114	0.121	1.67	615.667	5.0	146083.667	4898.0	111451.667	29216.733
LAZYPIPE	7.87	1.0	99.999	0.029	0.021	1.541	243.0	6.0	210236.0	5362.0	124985.0	35039.3
metaSPAdes	8.775	1.0	99.999	0.028	0.021	0.227	432.667	6.0	210317.0	5364.0	125016.0	35052.8
metaviralSPAdes	9.735	0.0	100.0	0.159	0.149	0.228	450.667	3.0	191566.0	16562.0	125016.0	63855.3
PEHaplo	77.42	148.0	99.874	0.039	0.032	2.949	145.667	230.0	312923.0	385.0	5712.0	1360.5
QuRe	4281.52	11.333	97.628	0.955	0.94	21.561	661.667	4.667	9349.667	327.667	5612.0	1921.567
QVG	52.19	1495.0	98.959	0.067	0.061	1.418	176.333	6.0	274497.0	5387.0	125041.0	45749.5
SPAdes	8.805	1.0	99.999	0.028	0.021	0.186	434.0	6.0	210317.0	5364.0	125016.0	35052.8
SSAKE	16.84	0.333	99.998	0.04	0.032	0.134	100.0	177.333	223227.0	109.0	9255.0	1258.9
TRACESPipe	191.53	1.0	99.999	0.028	0.021	9.768	347.333	4.0	143826.0	5387.0	125015.0	35956.5
TRACESPipeLite	44.375	0.0	99.651	0.963	0.953	2.832	316.667	6.0	168262.0	5387.0	125041.0	28043.7
VirGenA	1048.67	117.0	99.608	0.772	0.755	4.34	751.0	45.0	38219.0	49.0	2393.0	849.3
ViSpA	117.795	1309.667	98.537	0.081	0.07	6.876	132.667	11.333	1070682.0	4259.667	731683.333	157628.2
V-pipe	65.76	1157.0	99.096	0.054	0.046	0.104	112.333	5.0	160429.0	5387.0	125041.0	32085.8

Table S58: Results obtained for DS54 using the benchmark proposed. The execution time was measured in seconds, the RAM usage was measured in GB and the CPU usage is presented as a percentage. The executions were, when possible, capped at 6 threads and 48 GB of RAM.

Reconstruction tool	Execution time	SNPs	Identity	NCSD	NRC	RAM usage	CPU usage	Number of scaffolds	Reconstructed bases	Minimum scaffold length	Maximum scaffold length	Average scaffold length
coronaSPAdes	4.285	0.0	100.0	0.027	0.02	0.147	450.0	6.0	210356.0	5377.0	125033.0	35059.3
Haploflow	6.52	0.0	100.0	0.031	0.024	0.15	99.0	7.0	209821.0	5246.0	97636.0	29974.4
IRMA	511.585	6.0	99.993	0.121	0.119	1.681	615.0	5.0	146449.0	4911.0	111796.0	29289.8
LAZYPIPE	6.73	0.0	100.0	0.027	0.021	1.54	247.667	6.0	210294.0	5371.0	125028.0	35049.0
metaSPAdes	8.68	0.0	100.0	0.027	0.02	0.227	430.333	6.0	210360.0	5377.0	125037.0	35060.0
metaviralSPAdes	9.805	0.0	100.0	0.157	0.149	0.227	451.667	3.0	191590.0	16563.0	125037.0	63863.3
PEHaplo	77.29	14.0	99.99	0.033	0.027	2.934	146.667	236.0	317531.0	386.0	5125.0	1345.5
QuRe	2940.205	9.333	98.734	0.963	0.956	21.97	653.333	4.667	9153.667	288.0	3775.667	1847.5
QVG	51.87	2719.0	98.103	0.102	0.099	1.418	180.0	7.0	282128.0	5387.0	125043.0	40304.0
SPAdes	9.225	0.0	100.0	0.027	0.02	0.184	431.333	6.0	210360.0	5377.0	125037.0	35060.0
SSAKE	16.785	0.0	99.999	0.04	0.032	0.134	100.0	168.667	222414.0	107.667	10779.333	1320.9
TRACESPipe	206.685	0.0	100.0	0.027	0.02	9.768	332.0	4.0	143844.0	5387.0	125034.0	35961.0
TRACESPipeLite	43.725	0.0	100.0	0.998	0.995	2.832	318.0	6.0	168262.0	5387.0	125041.0	28043.7
VirGenA	1104.195	106.0	99.366	0.755	0.737	4.453	748.333	47.0	40533.0	70.0	2131.0	862.4
ViSpA	123.16	723.333	98.415	0.113	0.101	7.22	131.0	57.0	2607820.0	446.0	120733.0	45751.2
V-pipe	65.31	1248.0	98.469	0.088	0.077	0.104	112.333	5.0	160429.0	5387.0	125041.0	32085.8

Table S59: Results obtained for DS55 using the benchmark proposed. The execution time was measured in seconds, the RAM usage was measured in GB and the CPU usage is presented as a percentage. The executions were, when possible, capped at 6 threads and 48 GB of RAM.

Reconstruction tool	Execution time	SNPs	Identity	NCSD	NRC	RAM usage	CPU usage	Number of scaffolds	Reconstructed bases	Minimum scaffold length	Maximum scaffold length	Average scaffold length
coronaSPAdes	4.41	1.0	99.991	0.027	0.02	0.152	452.0	6.0	210365.0	5352.0	125038.0	35060.8
Haploflow	6.73	0.0	100.0	0.03	0.023	0.158	99.0	6.0	209884.0	5215.0	125010.0	34980.7
IRMA	625.51	30.333	99.969	0.126	0.121	1.686	614.667	5.0	146254.0	4919.0	111752.0	29250.8
LAZYPIPE	6.975	0.0	100.0	0.028	0.021	1.54	239.667	6.0	210254.0	5328.0	125032.0	35042.3
metaSPAdes	8.71	0.0	100.0	0.027	0.02	0.228	433.333	6.0	210365.0	5352.0	125038.0	35060.8
metaviralSPAdes	9.81	0.0	100.0	0.157	0.149	0.227	452.333	3.0	191599.0	16563.0	125038.0	63866.3
PEHaplo	77.335	1.0	99.999	0.03	0.023	2.952	146.667	231.0	311157.0	381.0	4353.0	1347.0
QuRe	4987.555	10.333	99.756	0.981	0.973	20.421	644.333	4.667	5779.667	257.333	2589.333	1370.1
QVG	51.48	5078.0	96.251	0.186	0.183	1.418	175.333	7.0	282124.0	5387.0	125039.0	40303.4
SPAdes	8.875	0.0	100.0	0.027	0.02	0.186	432.333	6.0	210365.0	5352.0	125038.0	35060.8
SSAKE	16.715	1.0	99.998	0.037	0.03	0.133	100.0	172.333	221907.0	111.333	9392.667	1287.967
TRACESPipe	214.09	1.0	99.999	0.027	0.021	9.768	328.0	4.0	143780.0	5369.0	125030.0	35945.0
TRACESPipeLite	41.18	0.0	0.0	1.0	0.998	2.832	329.333	5.0	160429.0	5387.0	125041.0	32085.8
VirGenA	1131.14	47.0	99.869	0.705	0.689	5.079	746.0	50.0	48048.0	64.0	2789.0	961.0
ViSpA	143.375	885.667	96.128	0.258	0.247	6.886	128.667	147.0	6180917.0	547.0	104055.0	42047.1
V-pipe	64.945	1529.0	97.021	0.162	0.146	0.104	112.333	5.0	160429.0	5387.0	125041.0	32085.8

Table S60: Results obtained for DS56 using the benchmark proposed. The execution time was measured in seconds, the RAM usage was measured in GB and the CPU usage is presented as a percentage. The executions were, when possible, capped at 6 threads and 48 GB of RAM.

Reconstruction tool	Execution time	SNPs	Identity	NCSD	NRC	RAM usage	CPU usage	Number of scaffolds	Reconstructed bases	Minimum scaffold length	Maximum scaffold length	Average scaffold length
coronaSPAdes	4.425	0.0	100.0	0.027	0.02	0.145	446.333	6.0	210347.0	5383.0	125013.0	35057.8
Haploflow	6.58	0.0	100.0	0.029	0.023	0.151	99.0	6.0	209839.0	5266.0	125013.0	34973.2
IRMA	760.465	131.667	99.832	0.118	0.109	1.747	614.333	5.0	148365.667	4898.0	113863.667	29673.133
LAZYPIPE	6.265	0.0	100.0	0.027	0.021	1.541	233.0	6.0	210280.0	5361.0	125011.0	35046.7
metaSPAdes	8.685	0.0	100.0	0.027	0.02	0.227	431.667	6.0	210347.0	5383.0	125013.0	35057.8
metaviralSPAdes	9.62	0.0	100.0	0.157	0.149	0.227	448.667	3.0	191553.0	16553.0	125013.0	63851.0
PEHaplo	76.98	0.0	99.982	0.03	0.023	2.929	145.667	232.0	319486.0	378.0	6558.0	1377.1
QuRe	3719.365	19.333	99.452	0.969	0.961	21.975	644.333	3.667	6776.0	777.333	3287.0	1907.833
QVG	45.865	9454.0	93.317	0.322	0.319	1.419	181.333	6.0	276745.0	5387.0	125045.0	46124.2
SPAdes	8.895	0.0	100.0	0.027	0.02	0.185	430.0	6.0	210347.0	5383.0	125013.0	35057.8
SSAKE	16.835	0.0	99.999	0.043	0.035	0.134	100.0	188.0	223020.0	103.0	10877.333	1186.367
TRACESPipe	214.57	0.0	100.0	0.027	0.021	9.768	327.333	4.0	143754.0	5381.0	124971.0	35938.5
TRACESPipeLite	41.935	0.0	0.0	1.0	0.999	2.833	328.0	5.0	160429.0	5387.0	125041.0	32085.8
VirGenA	1090.135	41.0	99.859	0.712	0.694	4.887	747.333	46.0	47781.0	301.0	3604.0	1038.7
ViSpA	121.98	669.667	96.097	0.434	0.426	6.946	119.0	200.0	6887728.0	461.0	83474.0	34438.6
V-pipe	64.435	1088.0	94.938	0.301	0.28	0.104	112.0	5.0	160429.0	5387.0	125041.0	32085.8

Table S61: Results obtained for DS57 using the benchmark proposed. The execution time was measured in seconds, the RAM usage was measured in GB and the CPU usage is presented as a percentage. The executions were, when possible, capped at 6 threads and 48 GB of RAM.

Reconstruction tool	Execution time	SNPs	Identity	NCSD	NRC	RAM usage	CPU usage	Number of scaffolds	Reconstructed bases	Minimum scaffold length	Maximum scaffold length	Average scaffold length
coronaSPAdes	2.935	67.0	99.901	0.036	0.026	0.148	414.667	8.0	187175.0	81.0	118363.0	23396.9
Haploflow	3.305	97.0	99.926	0.052	0.039	0.092	99.333	50.0	196302.0	513.0	15318.0	3926.0
IRMA	157.865	0.0	100.0	0.096	0.128	0.874	540.667	4.0	128277.0	4871.0	110232.0	32069.3
LAZYPIPE	6.71	16.0	99.874	0.036	0.025	1.523	260.333	8.0	186605.0	330.0	95821.0	23325.6
metaSPAdes	5.36	54.0	99.867	0.037	0.026	0.229	409.667	11.0	186838.0	71.0	105136.0	16985.3
metaviralSPAdes	–	–	–	–	–	–	–	–	–	–	–	–
PEHaplo	31.375	56.0	99.94	0.075	0.06	1.091	140.333	291.0	235200.0	376.0	2611.0	808.2
QuRe	571.335	17.667	99.514	0.986	0.969	14.26	592.333	8.333	6253.0	77.0	1723.0	716.933
QVG	21.51	160.0	99.893	0.035	0.025	1.408	153.333	4.0	143860.0	5387.0	125041.0	35965.0
SPAdes	6.93	38.0	99.928	0.035	0.024	0.162	355.0	22.0	195346.0	131.0	105523.0	8879.4
SSAKE	4.97	12.333	99.981	0.524	0.489	0.075	100.0	347.667	106998.0	103.333	1467.0	307.7
TRACESPipe	197.17	131.0	99.91	0.034	0.025	9.768	332.333	4.0	143861.0	5387.0	125042.0	35965.3
TRACESPipeLite	41.355	132.0	99.911	0.033	0.023	2.813	336.333	5.0	151693.0	5387.0	125041.0	30338.6
VirGenA	764.6	32.0	99.901	0.549	0.518	4.891	727.667	72.0	70380.0	115.0	2888.0	977.5
ViSpA	138.655	168.0	99.883	0.034	0.024	5.0	99.0	5.0	143673.0	0.0	124983.0	28734.6
V-pipe	45.1	141.0	99.684	0.043	0.032	0.104	114.0	5.0	160429.0	5387.0	125041.0	32085.8

Table S62: Results obtained for DS58 using the benchmark proposed. The execution time was measured in seconds, the RAM usage was measured in GB and the CPU usage is presented as a percentage. The executions were, when possible, capped at 6 threads and 48 GB of RAM.

Reconstruction tool	Execution time	SNPs	Identity	NCSD	NRC	RAM usage	CPU usage	Number of scaffolds	Reconstructed bases	Minimum scaffold length	Maximum scaffold length	Average scaffold length
coronaSPAdes	2.63	62.0	99.908	0.036	0.025	0.147	407.333	10.0	153918.0	81.0	105120.0	15391.8
Haploflow	2.67	94.0	99.914	0.069	0.053	0.076	99.333	40.0	160635.0	542.0	18358.0	4015.9
IRMA	162.62	0.667	99.999	0.095	0.127	0.874	508.333	5.0	144910.0	4882.0	110266.0	28982.0
LAZYPIPE	6.105	71.333	99.939	0.037	0.027	1.519	247.333	7.0	152818.0	5318.0	96717.0	21831.1
metaSPAdes	4.695	33.0	99.84	0.037	0.026	0.226	401.333	11.0	153524.0	71.0	105136.0	13956.7
metaviralSPAdes	–	–	–	–	–	–	–	–	–	–	–	–
PEHaplo	26.01	58.0	99.958	0.094	0.077	0.882	128.333	224.0	186980.0	378.0	3000.0	834.7
QuRe	427.43	3.333	99.789	0.995	0.983	21.496	540.333	7.0	2176.667	55.0	617.667	310.933
QVG	23.075	168.0	99.881	0.032	0.024	1.406	143.667	5.0	268901.0	5387.0	125041.0	53780.2
SPAdes	6.175	50.0	99.896	0.036	0.025	0.161	343.0	22.0	161709.0	131.0	105080.0	7350.4
SSAKE	4.31	12.0	99.98	0.507	0.468	0.067	100.0	309.0	92565.0	102.333	1256.667	299.6
TRACESPipe	168.845	174.0	99.877	0.033	0.023	9.768	366.0	4.0	143860.0	5387.0	125041.0	35965.0
TRACESPipeLite	42.845	141.333	99.903	0.033	0.023	2.782	327.0	6.0	168262.0	5387.0	125041.0	28043.7
VirGenA	674.175	105.333	99.88	0.301	0.277	4.426	718.667	59.0	107721.667	233.0	7460.667	2014.267
ViSpA	155.465	188.0	99.867	0.035	0.025	6.154	107.333	5.0	160133.0	5318.0	125016.0	32026.6
V-pipe	42.7	148.0	99.74	0.044	0.033	0.104	114.667	5.0	160429.0	5387.0	125041.0	32085.8

Table S63: Results obtained for DS59 using the benchmark proposed. The execution time was measured in seconds, the RAM usage was measured in GB and the CPU usage is presented as a percentage. The executions were, when possible, capped at 6 threads and 48 GB of RAM.

Reconstruction tool	Execution time	SNPs	Identity	NCSD	NRC	RAM usage	CPU usage	Number of scaffolds	Reconstructed bases	Minimum scaffold length	Maximum scaffold length	Average scaffold length
coronaSPAdes	3.875	46.0	99.967	0.037	0.026	0.164	435.0	12.0	207916.0	531.0	105629.0	17326.3
Haploflow	4.99	457.0	99.66	0.052	0.04	0.126	99.333	16.0	211700.0	1680.0	45394.0	13231.3
IRMA	279.805	1.0	99.999	0.103	0.128	1.261	579.0	5.0	144874.0	4888.0	110252.0	28974.8
LAZYPIPE	8.545	1.0	99.963	0.032	0.022	1.533	265.667	8.0	210190.0	4291.0	105465.0	26273.8
metaSPAdes	7.23	76.0	99.946	0.033	0.023	0.229	429.333	7.0	210082.0	328.0	124464.0	30011.7
metaviralSPAdes	–	–	–	–	–	–	–	–	–	–	–	–
PEHaplo	53.755	132.0	99.886	0.055	0.043	1.981	145.333	301.0	314300.0	381.0	3694.0	1044.2
QuRe	1377.655	21.0	99.51	0.976	0.954	21.302	610.667	5.0	6075.0	212.667	3038.667	1163.5
QVG	32.245	503.0	99.573	0.045	0.035	1.413	162.0	4.0	143860.0	5387.0	125041.0	35965.0
SPAdes	7.705	2.0	99.998	0.031	0.021	0.174	411.667	6.0	210332.0	5371.0	125032.0	35055.3
SSAKE	14.73	1.333	99.985	0.082	0.064	0.116	100.0	409.667	220053.333	101.333	3104.333	537.167
TRACESPipe	180.345	498.0	99.653	0.039	0.028	9.768	363.0	4.0	143859.0	5387.0	125040.0	35964.8
TRACESPipeLite	50.66	473.0	99.503	0.055	0.044	2.825	315.0	6.0	168262.0	5387.0	125041.0	28043.7
VirGenA	976.155	96.0	99.713	0.706	0.688	4.682	744.0	53.0	46445.0	170.0	2247.0	876.3
ViSpA	276.105	610.0	99.564	0.042	0.032	6.875	123.0	5.0	160186.0	5342.0	125007.0	32037.2
V-pipe	55.975	454.0	99.683	0.042	0.031	0.104	113.0	5.0	160429.0	5387.0	125041.0	32085.8

Table S64: Results obtained for DS60 using the benchmark proposed. The execution time was measured in seconds, the RAM usage was measured in GB and the CPU usage is presented as a percentage. The executions were, when possible, capped at 6 threads and 48 GB of RAM.

Reconstruction tool	Execution time	SNPs	Identity	NCSD	NRC	RAM usage	CPU usage	Number of scaffolds	Reconstructed bases	Minimum scaffold length	Maximum scaffold length	Average scaffold length
coronaSPAdes	4.54	0.0	100.0	0.03	0.021	0.153	437.333	6.0	210367.0	5374.0	125037.0	35061.2
Haploflow	6.56	206.0	99.844	0.032	0.023	0.152	99.0	8.0	211720.0	5095.0	115022.0	26465.0
IRMA	364.3	0.0	100.0	0.108	0.128	1.639	594.667	5.0	144923.0	4892.0	110283.0	28984.6
LAZYPIPE	8.9	0.0	99.976	0.031	0.022	1.54	256.0	7.0	210287.0	351.0	124743.0	30041.0
metaSPAdes	8.62	0.0	100.0	0.03	0.021	0.228	433.0	6.0	210367.0	5374.0	125037.0	35061.2
metaviralSPAdes	9.695	0.0	100.0	0.162	0.149	0.226	450.667	3.0	191597.0	16565.0	125037.0	63865.7
PEHaplo	78.54	146.0	99.894	0.032	0.024	2.94	149.667	218.0	334264.0	411.0	4869.0	1533.3
QuRe	2492.145	6.333	99.863	0.978	0.97	22.494	632.0	4.333	5290.667	365.0	2446.0	1215.2
QVG	42.885	736.0	99.488	0.047	0.04	1.419	173.333	5.0	268901.0	5387.0	125041.0	53780.2
SPAdes	9.33	0.0	100.0	0.03	0.021	0.185	427.333	6.0	210367.0	5374.0	125037.0	35061.2
SSAKE	17.125	0.333	99.998	0.042	0.032	0.134	100.0	175.0	224017.667	115.333	8673.0	1280.467
TRACESPipe	191.88	689.0	99.51	0.044	0.035	9.768	361.333	4.0	143867.0	5387.0	125046.0	35966.8
TRACESPipeLite	50.12	218.667	97.556	0.273	0.246	2.829	309.0	6.0	168263.0	5387.0	125041.0	28043.8
VirGenA	1022.68	49.0	99.685	0.711	0.69	5.317	746.0	52.0	46643.0	61.0	2554.0	897.0
ViSpA	438.595	784.0	99.413	0.051	0.04	6.925	128.0	5.0	160360.0	5360.0	125072.0	32072.0
V-pipe	65.25	586.0	99.59	0.042	0.033	0.104	112.667	5.0	160429.0	5387.0	125041.0	32085.8

Table S65: Results obtained for DS61 using the benchmark proposed. The execution time was measured in seconds, the RAM usage was measured in GB and the CPU usage is presented as a percentage. The executions were, when possible, capped at 6 threads and 48 GB of RAM.

**Reconstruction tool**	**Execution time**	**SNPs**	**Identity**	**NCSD**	**NRC**	**RAM usage**	**CPU usage**	**Number of scaffolds**	**Reconstructed bases**	**Minimum scaffold length**	**Maximum scaffold length**	**Average scaffold length**
coronaSPAdes	4.925	236.0	99.739	0.048	0.031	0.152	454.667	15.0	152771.0	51.0	55005.0	10184.7
Haploflow	–	–	–	–	–	–	–	–	–	–	–	–
IRMA	1.315	3.0	99.482	0.096	0.129	0.018	350.333	20.0	739977.667	3280.0	228301.0	36998.933
LAZYPIPE	5.2	509.0	99.373	0.401	0.362	1.135	200.333	176.0	103969.0	246.0	2358.0	590.7
metaSPAdes	11.16	264.0	99.775	0.066	0.049	1.28	324.0	63.0	153819.0	97.0	34311.0	2441.6
metaviralSPAdes	–	–	–	–	–	–	–	–	–	–	–	–
PEHaplo	62.695	63.0	99.924	0.393	0.357	1.381	145.667	201.0	118152.0	375.0	1679.0	587.8
QuRe	543.77	0.667	99.859	0.999	0.992	15.148	504.0	4.667	488.667	62.667	124.0	104.633
QVG	23.33	1201.0	99.166	0.076	0.06	1.113	147.0	5.0	268901.0	5387.0	125041.0	53780.2
SPAdes	12.685	127.0	99.849	0.042	0.028	0.215	375.667	18.0	152999.0	64.0	64084.0	8499.9
SSAKE	–	–	–	–	–	–	–	–	–	–	–	–
TRACESPipe	198.34	136.0	99.902	0.033	0.023	9.768	351.667	4.0	143861.0	5387.0	125042.0	35965.3
TRACESPipeLite	29.91	9.667	98.204	0.998	0.987	2.495	386.667	5.0	160560.0	5185.0	125374.0	32112.0
VirGenA	–	–	–	–	–	–	–	–	–	–	–	–
ViSpA	281.355	71.0	99.561	0.049	0.035	10.972	100.0	5.0	431884.0	5375.0	396401.0	86376.8
V-pipe	–	–	–	–	–	–	–	–	–	–	–	–

Table S66: Results obtained for DS62 using the benchmark proposed. The execution time was measured in seconds, the RAM usage was measured in GB and the CPU usage is presented as a percentage. The executions were, when possible, capped at 6 threads and 48 GB of RAM.

**Reconstruction tool**	**Execution time**	**SNPs**	**Identity**	**NCSD**	**NRC**	**RAM usage**	**CPU usage**	**Number of scaffolds**	**Reconstructed bases**	**Minimum scaffold length**	**Maximum scaffold length**	**Average scaffold length**
coronaSPAdes	5.605	35.0	99.969	0.036	0.025	0.131	446.0	11.0	155427.0	468.0	107424.0	14129.7
Haploflow	4.945	64.0	99.901	0.19	0.167	0.195	99.667	83.0	138074.0	528.0	5061.0	1663.5
IRMA	148.875	4.0	99.225	0.096	0.129	0.594	456.667	20.0	739993.0	3280.0	228301.0	36999.7
LAZYPIPE	6.485	49.0	99.911	0.036	0.025	1.14	276.333	8.0	154657.0	1010.0	109941.0	19332.1
metaSPAdes	10.69	49.0	99.856	0.037	0.027	1.268	317.333	13.0	153697.0	71.0	105176.0	11822.8
metaviralSPAdes	6.42	56.0	99.953	0.179	0.159	0.16	444.667	3.0	133759.0	5358.0	111885.0	44586.3
PEHaplo	22.625	39.0	99.958	0.057	0.043	0.641	142.333	171.0	202724.0	394.0	4707.0	1185.5
QuRe	1181.29	0.0	100.0	0.996	0.985	59.396	413.667	4.667	1090.667	24.0	551.667	239.233
QVG	22.615	151.0	99.891	0.032	0.024	1.116	175.667	5.0	268901.0	5387.0	125041.0	53780.2
SPAdes	11.695	31.0	99.981	0.032	0.023	0.21	366.0	14.0	159217.0	355.0	106624.0	11372.6
SSAKE	1.955	3.667	99.98	0.891	0.866	0.043	100.0	110.667	21937.0	102.0	500.333	198.367
TRACESPipe	201.68	116.0	99.922	0.033	0.023	9.768	355.333	4.0	143860.0	5387.0	125041.0	35965.0
TRACESPipeLite	50.57	135.0	99.904	0.033	0.024	2.82	396.333	6.0	168262.0	5387.0	125041.0	28043.7
VirGenA	924.485	204.0	99.778	0.361	0.332	4.556	649.667	28.0	99425.0	745.0	16600.0	3550.9
ViSpA	87.575	256.0	99.815	0.04	0.027	10.756	99.0	5.0	160146.0	5355.0	124967.0	32029.2
V-pipe	47.835	132.0	99.715	0.052	0.038	0.095	118.667	5.0	160429.0	5387.0	125041.0	32085.8

Table S67: Results obtained for DS63 using the benchmark proposed. The execution time was measured in seconds, the RAM usage was measured in GB and the CPU usage is presented as a percentage. The executions were, when possible, capped at 6 threads and 48 GB of RAM.

**Reconstruction tool**	**Execution time**	**SNPs**	**Identity**	**NCSD**	**NRC**	**RAM usage**	**CPU usage**	**Number of scaffolds**	**Reconstructed bases**	**Minimum scaffold length**	**Maximum scaffold length**	**Average scaffold length**
coronaSPAdes	5.115	126.0	99.957	0.027	0.018	0.177	437.333	17.0	358134.0	79.0	125432.0	21066.7
Haploflow	6.62	217.0	99.888	0.04	0.031	0.174	99.0	89.0	373224.0	521.0	21945.0	4193.5
IRMA	824.055	97.0	99.921	0.056	0.068	1.715	610.0	6.0	298596.333	4877.0	153774.333	49766.067
LAZYPIPE	10.14	152.0	99.854	0.027	0.018	1.539	281.0	14.0	356563.0	350.0	131634.0	25468.8
metaSPAdes	9.305	113.0	99.868	0.027	0.019	0.233	437.667	29.0	358115.0	57.0	145112.0	12348.8
metaviralSPAdes	–	–	–	–	–	–	–	–	–	–	–	–
PEHaplo	192.015	242.0	99.895	0.045	0.037	1.867	120.0	535.0	486718.0	376.0	4058.0	909.8
QuRe	2122.53	11.0	99.367	0.996	0.975	20.128	623.667	14.333	4255.333	71.0	907.0	296.467
QVG	45.55	335.667	99.881	0.024	0.019	1.419	168.0	7.0	593129.0	5387.0	162114.0	84732.7
SPAdes	13.14	54.0	99.939	0.025	0.017	0.188	321.667	53.0	370365.0	131.0	121039.0	6988.0
SSAKE	7.265	14.667	99.968	0.671	0.641	0.109	99.333	416.0	134437.0	100.667	1270.667	322.9
TRACESPipe	255.81	336.0	99.89	0.022	0.016	9.768	300.333	7.0	624802.0	5387.0	162114.0	89257.4
TRACESPipeLite	55.815	303.333	99.894	0.024	0.016	2.844	289.0	7.0	330376.0	5387.0	162114.0	47196.6
VirGenA	2002.26	52.333	99.877	0.763	0.742	5.45	739.0	76.667	76151.0	135.333	3894.0	996.9
ViSpA	321.31	275.0	99.873	0.023	0.017	6.139	124.0	12.0	747730.0	346.0	153863.0	62310.8
V-pipe	74.32	321.0	99.756	0.033	0.025	0.111	113.667	6.0	322543.0	5387.0	162114.0	53757.2

Table S68: Results obtained for DS64 using the benchmark proposed. The execution time was measured in seconds, the RAM usage was measured in GB and the CPU usage is presented as a percentage. The executions were, when possible, capped at 6 threads and 48 GB of RAM.

Reconstruction tool	Execution time	SNPs	Identity	NCSD	NRC	RAM usage	CPU usage	Number of scaffolds	Reconstructed bases	Minimum scaffold length	Maximum scaffold length	Average scaffold length
coronaSPAdes	5.185	81.0	99.821	0.03	0.02	0.17	446.0	22.0	351746.0	81.0	105120.0	15988.5
Haploflow	6.725	203.0	99.897	0.048	0.037	0.177	99.0	89.0	368848.0	524.0	19368.0	4144.4
IRMA	924.655	61.0	99.786	0.063	0.073	1.79	616.667	6.0	297266.333	4863.0	152903.333	49544.4
LAZYPIPE	10.56	131.0	99.929	0.029	0.023	1.539	293.667	32.0	354981.0	273.0	92612.0	11093.2
metaSPAdes	9.575	92.0	99.893	0.031	0.021	0.233	444.0	17.0	350428.0	71.0	105234.0	20613.4
metaviralSPAdes	11.27	2.0	99.96	0.985	0.968	0.232	457.333	1.0	5358.0	5358.0	5358.0	5358.0
PEHaplo	70.365	142.0	99.952	0.053	0.041	2.036	138.333	524.0	472293.0	376.0	4078.0	901.3
QuRe	1708.795	8.0	99.632	0.997	0.971	16.215	632.0	10.0	2621.667	131.667	460.333	260.267
QVG	43.555	429.667	99.857	0.029	0.02	1.417	167.667	6.0	441230.0	4952.0	172764.0	73538.3
SPAdes	15.89	48.0	99.846	0.029	0.02	0.187	312.667	62.0	364614.0	80.0	105128.0	5880.9
SSAKE	6.665	29.667	99.967	0.692	0.619	0.109	100.0	373.333	122899.0	102.333	1390.0	328.833
TRACESPipe	217.615	398.0	99.866	0.027	0.018	9.768	331.667	6.0	321125.0	4935.0	172764.0	53520.8
TRACESPipeLite	59.8	391.667	99.868	0.027	0.019	2.843	283.0	7.0	340591.0	4952.0	172764.0	48655.9
VirGenA	2028.57	32.333	99.85	0.875	0.845	5.204	719.667	39.0	39978.333	230.0	2755.0	944.7
ViSpA	241.025	231.667	99.901	0.025	0.019	5.383	121.667	13.0	851902.0	4935.0	148680.0	65530.9
V-pipe	77.81	434.0	99.686	0.038	0.027	0.113	112.667	6.0	332758.0	4952.0	172764.0	55459.7

Table S69: Results obtained for DS65 using the benchmark proposed. The execution time was measured in seconds, the RAM usage was measured in GB and the CPU usage is presented as a percentage. The executions were, when possible, capped at 6 threads and 48 GB of RAM.

Reconstruction tool	Execution time	SNPs	Identity	NCSD	NRC	RAM usage	CPU usage	Number of scaffolds	Reconstructed bases	Minimum scaffold length	Maximum scaffold length	Average scaffold length
coronaSPAdes	5.685	61.0	99.963	0.02	0.014	0.173	452.667	16.0	435772.0	81.0	193790.0	27235.8
Haploflow	7.96	63.0	99.958	0.039	0.031	0.208	99.333	113.0	448894.0	544.0	18022.0	3972.5
IRMA	690.105	0.667	100.0	0.051	0.071	3.483	613.667	6.0	373222.0	4881.0	228347.0	62203.7
LAZYPIPE	11.34	70.0	99.968	0.02	0.014	1.543	285.333	10.0	435025.0	5372.0	196252.0	43502.5
metaSPAdes	10.91	40.0	99.919	0.02	0.014	0.234	442.333	15.0	434957.0	71.0	192881.0	28997.1
metaviralSPAdes	–	–	–	–	–	–	–	–	–	–	–	–
PEHaplo	60.06	86.0	99.974	0.039	0.033	2.059	154.333	592.0	587990.0	378.0	4705.0	993.2
QuRe	1598.62	3.667	99.636	0.999	0.984	15.896	634.667	8.0	1648.667	105.333	341.0	217.2
QVG	48.065	221.667	99.941	0.02	0.014	1.42	172.0	6.0	504230.0	5387.0	235329.0	84038.3
SPAdes	11.665	33.0	99.97	0.019	0.014	0.202	421.667	34.0	445057.0	167.0	192883.0	13089.9
SSAKE	8.3	17.667	99.981	0.688	0.665	0.124	100.0	454.0	153953.667	102.0	1281.0	338.967
TRACESPipe	307.15	196.0	99.951	0.018	0.013	9.768	262.0	5.0	379189.0	5387.0	235329.0	75837.8
TRACESPipeLite	63.295	189.667	99.951	0.018	0.013	2.847	280.333	7.0	403590.0	5387.0	235329.0	57655.7
VirGenA	2023.705	2.0	99.964	0.989	0.975	6.078	747.0	6.0	5797.0	617.0	1716.0	966.2
ViSpA	670.29	232.0	99.94	0.019	0.013	4.608	120.0	6.0	395003.0	5347.0	234813.0	65833.8
V-pipe	82.375	195.0	99.805	0.029	0.022	0.128	113.333	6.0	395758.0	5387.0	235329.0	65959.7

Table S70: Results obtained for DS66 using the benchmark proposed. The execution time was measured in seconds, the RAM usage was measured in GB and the CPU usage is presented as a percentage. The executions were, when possible, capped at 6 threads and 48 GB of RAM.

Reconstruction tool	Execution time	SNPs	Identity	NCSD	NRC	RAM usage	CPU usage	Number of scaffolds	Reconstructed bases	Minimum scaffold length	Maximum scaffold length	Average scaffold length
coronaSPAdes	1.655	74.0	99.529	0.067	0.051	0.126	382.333	22.0	199154.0	617.0	57322.0	9052.5
Haploflow	0.98	12.0	99.958	0.782	0.729	0.043	99.333	29.0	52448.0	543.0	9432.0	1808.6
IRMA	77.875	113.333	99.004	0.112	0.147	0.306	348.0	20.0	738741.0	3280.0	228301.0	36937.1
LAZYPIPE	5.15	83.0	99.881	0.08	0.062	1.123	272.667	68.0	196275.0	311.0	10764.0	2886.4
metaSPAdes	–	–	–	–	–	–	–	–	–	–	–	–
metaviralSPAdes	–	–	–	–	–	–	–	–	–	–	–	–
PEHaplo	12.71	96.0	99.898	0.085	0.065	0.169	82.0	118.0	211011.0	403.0	6209.0	1788.2
QuRe	–	–	–	–	–	–	–	–	–	–	–	–
QVG	19.4	26.0	99.97	0.03	0.022	1.098	147.0	8.0	309875.0	5387.0	125041.0	38734.4
SPAdes	–	–	–	–	–	–	–	–	–	–	–	–
SSAKE	1.725	6.667	99.963	0.878	0.845	0.053	94.333	106.667	31727.0	155.667	988.0	297.333
TRACESPipe	140.65	0.0	100.0	0.027	0.019	9.768	320.333	4.0	143860.0	5387.0	125041.0	35965.0
TRACESPipeLite	28.03	1.0	99.65	1.0	0.992	2.415	408.333	4.0	154492.0	4628.0	125459.0	38623.0
VirGenA	475.715	27.0	99.879	0.674	0.647	4.516	614.333	99.0	52168.0	69.0	2488.0	526.9
ViSpA	83.735	76.0	97.49	0.106	0.087	10.377	70.333	41.0	2036329.0	590.0	119636.0	49666.6
V-pipe	37.265	47.0	93.199	0.539	0.501	0.096	121.667	5.0	160429.0	5387.0	125041.0	32085.8

Table S71: Results obtained for DS67 using the benchmark proposed. The execution time was measured in seconds, the RAM usage was measured in GB and the CPU usage is presented as a percentage. The executions were, when possible, capped at 6 threads and 48 GB of RAM.

Reconstruction tool	Execution time	SNPs	Identity	NCSD	NRC	RAM usage	CPU usage	Number of scaffolds	Reconstructed bases	Minimum scaffold length	Maximum scaffold length	Average scaffold length
coronaSPAdes	2.31	534.0	99.23	0.09	0.068	0.128	440.333	44.0	198727.0	115.0	21526.0	4516.5
Haploflow	–	–	–	–	–	–	–	–	–	–	–	–
IRMA	2.01	113.333	99.004	0.112	0.147	0.018	258.667	20.0	738741.0	3280.0	228301.0	36937.1
LAZYPIPE	5.98	326.333	99.717	0.104	0.082	1.123	324.0	85.0	196904.0	303.0	10764.0	2316.5
metaSPAdes	8.765	396.0	99.257	0.093	0.071	1.144	282.667	53.0	199083.0	57.0	18809.0	3756.3
metaviralSPAdes	–	–	–	–	–	–	–	–	–	–	–	–
PEHaplo	13.335	174.0	99.81	0.15	0.123	0.167	82.667	164.0	197500.0	377.0	4251.0	1204.3
QuRe	–	–	–	–	–	–	–	–	–	–	–	–
QVG	–	–	–	–	–	–	–	–	–	–	–	–
SPAdes	7.44	372.0	99.231	0.097	0.076	0.212	324.0	37.0	196493.0	119.0	28671.0	5310.6
SSAKE	23.175	8.667	99.772	0.982	0.967	0.112	99.333	21.667	4972.333	166.0	290.667	229.333
TRACESPipe	196.665	374.0	99.443	0.064	0.046	9.768	353.333	4.0	143862.0	5387.0	125041.0	35965.5
TRACESPipeLite	28.64	1.0	82.942	1.0	0.992	2.419	392.333	4.667	157048.0	4628.0	125451.0	33940.167
VirGenA	478.48	167.0	99.547	0.692	0.659	4.371	613.333	87.0	50548.0	102.0	2663.0	581.0
ViSpA	83.905	176.0	97.402	0.111	0.092	10.385	71.333	41.0	2036252.0	590.0	119633.0	49664.7
V-pipe	–	–	–	–	–	–	–	–	–	–	–	–

Table S72: Results obtained for DS68 using the benchmark proposed. The execution time was measured in seconds, the RAM usage was measured in GB and the CPU usage is presented as a percentage. The executions were, when possible, capped at 6 threads and 48 GB of RAM.

Reconstruction tool	Execution time	SNPs	Identity	NCSD	NRC	RAM usage	CPU usage	Number of scaffolds	Reconstructed bases	Minimum scaffold length	Maximum scaffold length	Average scaffold length
coronaSPAdes	2.645	1849.0	98.38	0.144	0.119	0.128	450.667	122.0	198666.0	100.0	11091.0	1628.4
Haploflow	–	–	–	–	–	–	–	–	–	–	–	–
IRMA	2.06	113.333	99.004	0.112	0.147	0.018	255.0	20.0	738741.0	3280.0	228301.0	36937.1
LAZYPIPE	8.955	1259.333	98.683	0.171	0.145	1.123	408.333	214.0	220088.0	302.0	4541.0	1028.4
metaSPAdes	9.21	1236.0	97.389	0.449	0.402	1.234	291.667	348.0	156951.0	58.0	4204.0	451.0
metaviralSPAdes	–	–	–	–	–	–	–	–	–	–	–	–
PEHaplo	17.855	305.0	99.634	0.237	0.203	0.161	84.667	205.0	196232.0	375.0	3007.0	957.2
QuRe	–	–	–	–	–	–	–	–	–	–	–	–
QVG	–	–	–	–	–	–	–	–	–	–	–	–
SPAdes	8.15	1005.0	98.627	0.416	0.369	0.214	359.0	238.0	139204.0	104.0	4871.0	584.9
SSAKE	–	–	–	–	–	–	–	–	–	–	–	–
TRACESPipe	198.29	853.0	99.11	0.076	0.057	9.768	352.0	4.0	143860.0	5387.0	125041.0	35965.0
TRACESPipeLite	24.76	7.0	97.13	1.0	0.993	2.422	431.0	2.0	130636.0	5185.0	125451.0	65318.0
VirGenA	562.43	547.0	98.758	0.703	0.663	4.491	626.0	99.0	50967.0	52.0	1807.0	514.8
ViSpA	81.63	429.0	97.245	0.119	0.099	10.389	71.333	41.0	2036253.0	590.0	119629.0	49664.7
V-pipe	–	–	–	–	–	–	–	–	–	–	–	–

Table S73: Results obtained for DS69 using the benchmark proposed. The execution time was measured in seconds, the RAM usage was measured in GB and the CPU usage is presented as a percentage. The executions were, when possible, capped at 6 threads and 48 GB of RAM.

Reconstruction tool	Execution time	SNPs	Identity	NCSD	NRC	RAM usage	CPU usage	Number of scaffolds	Reconstructed bases	Minimum scaffold length	Maximum scaffold length	Average scaffold length
coronaSPAdes	2.83	4386.0	95.882	0.289	0.261	0.13	461.667	412.0	216325.0	225.0	2919.0	525.1
Haploflow	–	–	–	–	–	–	–	–	–	–	–	–
IRMA	2.06	113.333	99.004	0.112	0.147	0.018	257.333	20.0	738741.0	3280.0	228301.0	36937.1
LAZYPIPE	–	–	–	–	–	–	–	–	–	–	–	–
metaSPAdes	6.675	9.0	98.845	0.998	0.986	1.261	230.0	5.0	779.0	82.0	250.0	155.8
metaviralSPAdes	–	–	–	–	–	–	–	–	–	–	–	–
PEHaplo	32.96	860.0	99.135	0.383	0.344	0.134	95.0	241.0	187855.0	375.0	3105.0	779.5
QuRe	–	–	–	–	–	–	–	–	–	–	–	–
QVG	–	–	–	–	–	–	–	–	–	–	–	–
SPAdes	–	–	–	–	–	–	–	–	–	–	–	–
SSAKE	–	–	–	–	–	–	–	–	–	–	–	–
TRACESPipe	–	–	–	–	–	–	–	–	–	–	–	–
TRACESPipeLite	–	–	–	–	–	–	–	–	–	–	–	–
VirGenA	626.61	864.0	97.171	0.817	0.78	4.225	642.0	138.0	37318.0	47.0	1047.0	270.4
ViSpA	77.325	1233.333	96.587	0.148	0.123	10.387	71.0	16.667	1949510.0	3304.667	1308182.333	276180.6
V-pipe	–	–	–	–	–	–	–	–	–	–	–	–

Table S74: Results obtained for DS70 using the benchmark proposed. The execution time was measured in seconds, the RAM usage was measured in GB and the CPU usage is presented as a percentage. The executions were, when possible, capped at 6 threads and 48 GB of RAM.

Reconstruction tool	Execution time	SNPs	Identity	NCSD	NRC	RAM usage	CPU usage	Number of scaffolds	Reconstructed bases	Minimum scaffold length	Maximum scaffold length	Average scaffold length
coronaSPAdes	3.54	60.0	99.906	0.037	0.025	0.134	411.333	9.0	202625.0	127.0	112520.0	22513.9
Haploflow	4.27	66.0	99.92	0.032	0.024	0.046	99.333	24.0	391070.0	1226.0	95377.0	16294.6
IRMA	410.355	61.667	99.94	0.095	0.132	1.69	530.667	20.0	740099.0	3280.0	228301.0	37005.0
LAZYPIPE	9.285	62.0	99.941	0.037	0.025	1.177	274.0	7.0	202499.0	5353.0	111952.0	28928.4
metaSPAdes	–	–	–	–	–	–	–	–	–	–	–	–
metaviralSPAdes	–	–	–	–	–	–	–	–	–	–	–	–
PEHaplo	86.75	49.0	99.933	0.039	0.028	3.011	144.667	233.0	320369.0	379.0	5691.0	1375.0
QuRe	3951.595	3.0	99.968	0.945	0.906	18.99	548.667	4.667	10751.0	774.667	5865.0	2364.167
QVG	42.615	35.0	99.97	0.031	0.022	1.148	179.333	10.0	320860.0	5387.0	125041.0	32086.0
SPAdes	–	–	–	–	–	–	–	–	–	–	–	–
SSAKE	17.94	33.333	99.968	0.035	0.025	0.212	99.0	628.667	481386.0	154.0	7145.0	766.3
TRACESPipe	150.87	2.0	99.998	0.027	0.019	9.768	322.667	4.0	143860.0	5387.0	125041.0	35965.0
TRACESPipeLite	40.235	0.0	95.14	0.999	0.992	2.649	404.333	6.0	168593.333	5385.333	125374.0	28098.867
VirGenA	1246.765	8.0	99.905	0.868	0.844	4.121	670.667	28.0	21501.0	309.0	1898.0	767.9
ViSpA	481.925	62.0	99.941	0.037	0.024	11.228	117.0	5.0	160219.0	5353.0	125027.0	32043.8
V-pipe	64.205	60.0	99.96	0.035	0.023	0.096	112.667	5.0	160429.0	5387.0	125041.0	32085.8

Table S75: Results obtained for DS71 using the benchmark proposed. The execution time was measured in seconds, the RAM usage was measured in GB and the CPU usage is presented as a percentage. The executions were, when possible, capped at 6 threads and 48 GB of RAM.

Reconstruction tool	Execution time	SNPs	Identity	NCSD	NRC	RAM usage	CPU usage	Number of scaffolds	Reconstructed bases	Minimum scaffold length	Maximum scaffold length	Average scaffold length
coronaSPAdes	8.935	59.0	99.837	0.038	0.026	0.191	456.333	15.0	203934.0	127.0	112453.0	13595.6
Haploflow	12.225	72.0	99.927	0.049	0.035	0.466	99.0	66.0	236820.0	510.0	15720.0	3588.2
IRMA	6.62	61.667	99.94	0.095	0.132	0.018	579.333	20.0	740099.0	3280.0	228301.0	37005.0
LAZYPIPE	11.62	51.0	99.856	0.036	0.025	1.178	291.0	7.0	202633.0	5346.0	111890.0	28947.6
metaSPAdes	16.59	59.0	99.843	0.037	0.025	1.32	378.667	10.0	202606.0	71.0	112509.0	20260.6
metaviralSPAdes	12.26	0.0	0.0	1.0	0.994	0.177	459.333	1.0	16543.0	16543.0	16543.0	16543.0
PEHaplo	79.97	34.0	99.943	0.039	0.028	2.978	152.667	186.0	306040.0	382.0	5740.0	1645.4
QuRe	2322.935	5.333	99.956	0.924	0.886	16.685	521.0	6.0	21886.333	2062.667	5567.333	3581.767
QVG	–	–	–	–	–	–	–	–	–	–	–	–
SPAdes	18.805	56.0	99.775	0.037	0.025	0.215	408.333	10.0	203372.0	87.0	105110.0	20337.2
SSAKE	7.075	68.667	99.825	0.658	0.614	0.191	99.0	138.0	85429.0	195.333	2125.0	618.133
TRACESPipe	212.975	60.0	99.949	0.035	0.024	9.768	357.333	4.0	143867.0	5387.0	125046.0	35966.8
TRACESPipeLite	37.72	1.0	97.806	0.999	0.991	2.665	400.333	6.0	167425.0	4628.0	125374.0	27904.2
VirGenA	1644.225	17.0	99.671	0.941	0.925	3.796	671.0	11.0	11665.0	293.0	3574.0	1060.5
ViSpA	560.355	61.0	99.931	0.036	0.025	11.279	120.0	5.0	160195.0	5343.0	125027.0	32039.0
V-pipe	–	–	–	–	–	–	–	–	–	–	–	–

Table S76: Results obtained for DS72 using the benchmark proposed. The execution time was measured in seconds, the RAM usage was measured in GB and the CPU usage is presented as a percentage. The executions were, when possible, capped at 6 threads and 48 GB of RAM.

Reconstruction tool	Execution time	SNPs	Identity	NCSD	NRC	RAM usage	CPU usage	Number of scaffolds	Reconstructed bases	Minimum scaffold length	Maximum scaffold length	Average scaffold length
coronaSPAdes	12.98	97.0	99.77	0.041	0.028	0.292	462.667	50.0	212108.0	79.0	112394.0	4242.2
Haploflow	18.43	4.0	99.98	0.874	0.82	0.831	99.0	29.0	25690.0	509.0	4784.0	885.9
IRMA	6.64	61.667	99.94	0.095	0.132	0.018	586.0	20.0	740099.0	3280.0	228301.0	37005.0
LAZYPIPE	18.175	79.0	99.817	0.039	0.026	1.178	421.667	15.0	205542.0	312.0	65453.0	13702.8
metaSPAdes	17.345	57.0	99.84	0.037	0.025	1.311	387.333	10.0	202541.0	71.0	112509.0	20254.1
metaviralSPAdes	12.375	0.0	0.0	1.0	0.994	0.189	463.0	1.0	16543.0	16543.0	16543.0	16543.0
PEHaplo	79.745	35.0	99.939	0.042	0.031	2.989	156.667	231.0	317751.0	380.0	5139.0	1375.5
QuRe	2940.78	4.333	99.973	0.906	0.854	17.277	524.333	6.667	23099.333	501.667	8455.0	3466.633
QVG	–	–	–	–	–	–	–	–	–	–	–	–
SPAdes	23.365	51.0	99.789	0.037	0.025	0.255	432.333	10.0	203414.0	87.0	105152.0	20341.4
SSAKE	–	–	–	–	–	–	–	–	–	–	–	–
TRACESPipe	216.18	63.0	99.948	0.036	0.024	9.768	352.667	4.0	143865.0	5387.0	125044.0	35966.3
TRACESPipeLite	34.42	1.0	96.49	0.999	0.991	2.678	373.333	6.0	167425.0	4628.0	125374.0	27904.2
VirGenA	2439.03	7.0	99.791	0.972	0.958	5.137	684.0	3.0	6025.0	676.0	3498.0	2008.3
ViSpA	580.625	57.0	99.931	0.037	0.025	11.305	120.667	5.0	160213.0	5343.0	125043.0	32042.6
V-pipe	–	–	–	–	–	–	–	–	–	–	–	–

Table S77: Results obtained for DS73 using the benchmark proposed. The execution time was measured in seconds, the RAM usage was measured in GB and the CPU usage is presented as a percentage. The executions were, when possible, capped at 6 threads and 48 GB of RAM.

Reconstruction tool	Execution time	SNPs	Identity	NCSD	NRC	RAM usage	CPU usage	Number of scaffolds	Reconstructed bases	Minimum scaffold length	Maximum scaffold length	Average scaffold length
coronaSPAdes	16.535	621.0	99.483	0.058	0.041	0.334	452.667	139.0	231351.0	100.0	24551.0	1664.4
Haploflow	–	–	–	–	–	–	–	–	–	–	–	–
IRMA	6.67	61.667	99.94	0.095	0.132	0.018	590.0	20.0	740099.0	3280.0	228301.0	37005.0
LAZYPIPE	–	–	–	–	–	–	–	–	–	–	–	–
metaSPAdes	27.795	822.0	98.968	0.081	0.061	1.298	398.667	148.0	225094.0	97.0	12654.0	1520.9
metaviralSPAdes	–	–	–	–	–	–	–	–	–	–	–	–
PEHaplo	81.845	30.0	99.944	0.04	0.03	2.983	159.333	188.0	301636.0	379.0	8762.0	1604.4
QuRe	2625.565	2.0	99.939	0.985	0.975	17.85	484.333	5.0	6822.667	775.333	2190.333	1364.533
QVG	–	–	–	–	–	–	–	–	–	–	–	–
SPAdes	48.21	300.0	99.633	0.054	0.037	0.357	397.0	20.0	204437.0	87.0	112135.0	10221.9
SSAKE	–	–	–	–	–	–	–	–	–	–	–	–
TRACESPipe	–	–	–	–	–	–	–	–	–	–	–	–
TRACESPipeLite	33.17	4.333	92.915	1.0	0.992	2.671	369.0	6.0	167654.0	4628.0	125374.0	27942.3
VirGenA	–	–	–	–	–	–	–	–	–	–	–	–
ViSpA	549.38	60.0	99.921	0.037	0.026	11.258	119.667	5.0	160230.0	5344.0	125057.0	32046.0
V-pipe	–	–	–	–	–	–	–	–	–	–	–	–

Table S78: Results obtained by dnadiff [6] when analysing the reconstruction of DS1. Only one execution of HVRS is represented.

Reconstruction tool	Total seqs	Aligned seqs	Aligned bases	Aligned bases (%)	Unaligned bases	Unaligned bases (%)	Average length	SNPs	Identity
coronaSPAdes	182	182	83085	99.9976	2	0.0024	455.1530	131	99.824
Haploflow	–	–	–	–	–	–	–	–	–
IRMA	4	4	95726	99.9958	4	0.0042	712.6889	99	96.085
LAZYPIPE	152	152	65304	99.9954	3	0.0046	425.7403	64	99.881
metaSPAdes	210	210	88861	99.9977	2	0.0023	423.1476	128	99.854
metaviralSPAdes	–	–	–	–	–	–	–	–	–
PEHaplo	98	98	58459	99.9949	3	0.0051	590.3500	672	99.749
QuRe	–	–	–	–	–	–	–	–	–
QVG	3	3	138473	100.0000	0	0.0000	46157.6667	1301	99.064
SPAdes	179	179	82066	99.9976	2	0.0024	457.0778	104	99.858
SSAKE	2	2	248	100.0000	0	0.0000	124.0000	0	100.000
TRACESPipe	4	4	113776	79.0880	30084	20.9120	904.0250	100	97.349
TRACESPipeLite	5	4	112549	74.1952	39144	25.8048	848.6031	66	97.028
V-pipe	5	3	5704	3.5555	154725	96.4445	139.1220	0	95.791
VirGenA	238	237	85972	99.9314	59	0.0686	367.6026	88	99.884
ViSpA	75	74	2289898	99.8929	2454	0.1071	326.3165	94	95.123

Table S79: Results obtained by dnadiff [6] when analysing the reconstruction of DS2. Only one execution of HVRS is represented.

Reconstruction tool	Total seqs	Aligned seqs	Aligned bases	Aligned bases (%)	Unaligned bases	Unaligned bases (%)	Average length	SNPs	Identity
coronaSPAdes	100	100	129534	99.9954	6	0.0046	1295.2647	120	99.775
Haploflow	–	–	–	–	–	–	–	–	–
IRMA	4	4	122405	100.0000	0	0.0000	4372.4643	49	98.394
LAZYPIPE	92	92	121346	99.9959	5	0.0041	1291.8723	114	99.896
metaSPAdes	104	104	130237	99.9954	6	0.0046	1244.3883	156	99.721
metaviralSPAdes	–	–	–	–	–	–	–	–	–
PEHaplo	102	102	127508	99.9969	4	0.0031	1250.0784	114	99.877
QuRe	–	–	–	–	–	–	–	–	–
QVG	4	4	143858	99.9986	2	0.0014	35964.5000	955	99.333
SPAdes	101	101	129739	99.9214	102	0.0786	1292.6600	109	99.909
SSAKE	55	55	8469	100.0000	0	0.0000	153.9818	3	99.965
TRACESPipe	4	4	141490	98.3526	2370	1.6474	7860.5556	135	99.060
TRACESPipeLite	5	4	140020	92.3049	11673	7.6951	7000.8500	153	98.787
V-pipe	5	4	68492	42.6930	91937	57.3070	317.7523	24	94.134
VirGenA	114	113	108795	99.9458	59	0.0542	928.0348	97	99.881
ViSpA	80	79	3611375	99.9794	745	0.0206	1894.4265	137	97.596

Table S80: Results obtained by dnadiff [6] when analysing the reconstruction of DS3. Only one execution of HVRS is represented.

Reconstruction tool	Total seqs	Aligned seqs	Aligned bases	Aligned bases (%)	Unaligned bases	Unaligned bases (%)	Average length	SNPs	Identity
coronaSPAdes	21	21	138037	100.0000	0	0.0000	7628.9444	39	99.868
Haploflow	65	65	52460	100.0000	0	0.0000	802.8254	34	99.882
IRMA	4	4	127894	100.0000	0	0.0000	25579.6000	4	99.921
LAZYPIPE	19	19	135584	100.0000	0	0.0000	6793.4000	60	99.950
metaSPAdes	17	17	136529	99.9268	100	0.0732	8005.9412	60	99.813
metaviralSPAdes	–	–	–	–	–	–	–	–	–
PEHaplo	116	116	146720	100.0000	0	0.0000	1260.0345	55	99.931
QuRe	3	2	192	66.8990	95	33.1010	96.0000	0	100.000
QVG	4	4	143858	99.9986	2	0.0014	35964.5000	452	99.685
SPAdes	27	27	143582	99.9304	100	0.0696	5126.0000	37	99.926
SSAKE	114	114	19911	100.0000	0	0.0000	174.6579	4	99.980
TRACESPipe	4	4	143518	99.7623	342	0.2377	35879.5000	152	99.850
TRACESPipeLite	5	5	143881	94.8501	7812	5.1499	35879.5000	126	99.869
V-pipe	5	4	126205	78.6672	34224	21.3328	1225.0101	46	97.085
VirGenA	64	63	114363	99.8952	120	0.1048	1549.3243	66	99.864
ViSpA	9	8	483660	100.0000	0	0.0000	25607.0000	132	99.514

Table S81: Results obtained by dnadiff [6] when analysing the reconstruction of DS4. Only one execution of HVRS is represented.

Reconstruction tool	Total seqs	Aligned seqs	Aligned bases	Aligned bases (%)	Unaligned bases	Unaligned bases (%)	Average length	SNPs	Identity
coronaSPAdes	14	14	138001	99.9276	100	0.0724	9851.4286	74	99.837
Haploflow	82	82	126008	100.0000	0	0.0000	1503.9500	68	99.908
IRMA	4	4	128279	100.0000	0	0.0000	25655.8000	1	99.992
LAZYPIPE	8	8	136217	100.0000	0	0.0000	17011.5000	72	99.889
metaSPAdes	8	8	136315	99.9282	98	0.0718	12383.2727	73	99.899
metaviralSPAdes	1	1	5358	100.0000	0	0.0000	5358.0000	1	99.940
PEHaplo	180	180	165859	99.9988	2	0.0012	931.3077	86	99.935
QuRe	7	6	620	87.2011	91	12.7989	108.2000	0	100.000
QVG	4	4	143858	99.9986	2	0.0014	35964.5000	197	99.859
SPAdes	7	7	137172	99.8559	198	0.1441	15257.6667	68	99.791
SSAKE	174	174	37612	99.9973	1	0.0027	216.1609	6	99.984
TRACESPipe	4	4	143701	99.8895	159	0.1105	35925.2500	111	99.920
TRACESPipeLite	5	5	143953	94.8976	7740	5.1024	35936.2500	128	99.910
V-pipe	5	4	142613	88.8948	17816	11.1052	15845.8889	151	99.490
VirGenA	50	50	114754	99.9573	49	0.0427	2028.7544	98	99.831
ViSpA	5	4	143528	100.0000	0	0.0000	30096.6000	162	99.782

Table S82: Results obtained by dnadiff [6] when analysing the reconstruction of DS5. Only one execution of HVRS is represented.

Reconstruction tool	Total seqs	Aligned seqs	Aligned bases	Aligned bases (%)	Unaligned bases	Unaligned bases (%)	Average length	SNPs	Identity
coronaSPAdes	9	9	137420	100.0000	0	0.0000	17150.6250	36	99.884
Haploflow	42	42	143037	100.0000	0	0.0000	3533.0526	60	99.949
IRMA	4	4	128314	100.0000	0	0.0000	25663.2000	0	100.000
LAZYPIPE	10	10	137370	100.0000	0	0.0000	13677.4000	53	99.947
metaSPAdes	7	7	136233	99.9267	100	0.0733	15670.8889	37	99.824
metaviralSPAdes	–	–	–	–	–	–	–	–	–
PEHaplo	204	204	167437	99.9994	1	0.0006	810.4350	80	99.919
QuRe	6	5	1328	90.7724	135	9.2276	265.6000	6	99.471
QVG	4	4	143858	99.9986	2	0.0014	35964.5000	157	99.891
SPAdes	19	19	145008	99.9318	99	0.0682	8170.3529	32	99.931
SSAKE	313	313	91113	100.0000	0	0.0000	294.6271	7	99.982
TRACESPipe	4	4	143762	99.9319	98	0.0681	35940.5000	153	99.893
TRACESPipeLite	5	5	143991	94.9226	7702	5.0774	35948.0000	148	99.885
V-pipe	5	4	143412	89.3928	17017	10.6072	34271.2500	64	99.627
VirGenA	40	40	114988	99.9748	29	0.0252	2570.5349	67	99.866
ViSpA	5	4	143678	100.0000	0	0.0000	35919.5000	158	99.884

Table S83: Results obtained by dnadiff [6] when analysing the reconstruction of DS6. Only one execution of HVRS is represented.

Reconstruction tool	Total seqs	Aligned seqs	Aligned bases	Aligned bases (%)	Unaligned bases	Unaligned bases (%)	Average length	SNPs	Identity
coronaSPAdes	10	10	137343	99.9287	98	0.0713	17109.7500	58	99.912
Haploflow	21	21	165922	99.9711	48	0.0289	6905.3500	98	99.923
IRMA	4	4	128304	100.0000	0	0.0000	25660.8000	0	100.000
LAZYPIPE	8	8	136723	100.0000	0	0.0000	17025.1250	62	99.954
metaSPAdes	9	9	136739	99.8539	200	0.1461	12391.5455	68	99.904
metaviralSPAdes	1	1	5358	100.0000	0	0.0000	5358.0000	1	99.940
PEHaplo	203	203	177772	100.0000	0	0.0000	851.4518	41	99.962
QuRe	2	2	1429	100.0000	0	0.0000	714.5000	9	99.367
QVG	4	4	143858	99.9986	2	0.0014	35964.5000	149	99.893
SPAdes	25	25	146732	99.9980	3	0.0020	5662.7200	16	99.944
SSAKE	366	366	138013	99.9978	3	0.0022	387.2277	10	99.988
TRACESPipe	4	4	143780	99.9444	80	0.0556	35945.0000	148	99.894
TRACESPipeLite	5	4	143724	94.7466	7969	5.2534	35931.0000	163	99.885
V-pipe	5	4	143379	89.3722	17050	10.6278	35844.7500	148	99.886
VirGenA	40	40	116127	99.9466	62	0.0534	2425.4783	63	99.851
ViSpA	5	4	143613	100.0000	0	0.0000	35903.2500	156	99.891

Table S84: Results obtained by dnadiff [6] when analysing the reconstruction of DS7. Only one execution of HVRS is represented.

Reconstruction tool	Total seqs	Aligned seqs	Aligned bases	Aligned bases (%)	Unaligned bases	Unaligned bases (%)	Average length	SNPs	Identity
coronaSPAdes	11	11	137556	99.9985	2	0.0015	17125.1250	60	99.864
Haploflow	17	17	147874	99.9459	80	0.0541	8398.6471	42	99.862
IRMA	4	4	128337	100.0000	0	0.0000	25667.4000	0	100.000
LAZYPIPE	5	5	136271	100.0000	0	0.0000	20978.2857	95	99.733
metaSPAdes	10	10	136839	99.9270	100	0.0730	11394.9167	52	99.869
metaviralSPAdes	–	–	–	–	–	–	–	–	–
PEHaplo	208	208	192610	99.9964	7	0.0036	944.5989	55	99.957
QuRe	5	5	3694	99.9729	1	0.0271	586.7500	0	99.706
QVG	4	4	143858	99.9986	2	0.0014	35964.5000	149	99.893
SPAdes	25	25	146714	99.9980	3	0.0020	6129.3478	20	99.941
SSAKE	304	304	152481	100.0000	0	0.0000	534.7591	24	99.976
TRACESPipe	4	4	143834	99.9819	26	0.0181	35958.5000	162	99.885
TRACESPipeLite	5	5	144200	95.0604	7493	4.9396	35952.5000	144	99.896
V-pipe	5	4	143447	89.4146	16982	10.5854	35861.7500	147	99.886
VirGenA	15	15	117822	99.9847	18	0.0153	5139.9565	93	99.881
ViSpA	5	4	143678	100.0000	0	0.0000	30127.2000	165	99.840

Table S85: Results obtained by dnadiff [6] when analysing the reconstruction of DS8. Only one execution of HVRS is represented.

Reconstruction tool	Total seqs	Aligned seqs	Aligned bases	Aligned bases (%)	Unaligned bases	Unaligned bases (%)	Average length	SNPs	Identity
coronaSPAdes	10	10	137518	100.0000	0	0.0000	19572.5714	46	99.876
Haploflow	12	12	147403	99.9722	41	0.0278	11911.0000	119	99.870
IRMA	4	4	128349	100.0000	0	0.0000	25669.8000	0	100.000
LAZYPIPE	9	9	137996	100.0000	0	0.0000	16365.4444	52	99.879
metaSPAdes	10	10	136896	99.9263	101	0.0737	9973.8571	21	99.829
metaviralSPAdes	–	–	–	–	–	–	–	–	–
PEHaplo	186	186	208290	99.9990	2	0.0010	1138.3012	51	99.951
QuRe	3	3	9628	100.0000	0	0.0000	3209.3333	33	99.378
QVG	4	4	143858	99.9986	2	0.0014	35964.5000	147	99.894
SPAdes	25	25	146751	100.0000	0	0.0000	5894.0000	22	99.940
SSAKE	132	132	153157	100.0000	0	0.0000	1433.9216	5	99.994
TRACESPipe	4	4	143827	99.9771	33	0.0229	35956.7500	126	99.912
TRACESPipeLite	5	5	144026	94.9457	7667	5.0543	35956.0000	129	99.911
V-pipe	5	4	143517	89.4583	16912	10.5417	35879.2500	160	99.887
VirGenA	41	41	116653	99.9186	95	0.0814	2401.8723	96	99.869
ViSpA	5	4	143719	99.9993	1	0.0007	35929.7500	158	99.884

Table S86: Results obtained by dnadiff [6] when analysing the reconstruction of DS9. Only one execution of HVRS is represented.

Reconstruction tool	Total seqs	Aligned seqs	Aligned bases	Aligned bases (%)	Unaligned bases	Unaligned bases (%)	Average length	SNPs	Identity
coronaSPAdes	267	183	81775	67.8406	38765	32.1594	446.8579	117	99.856
Haploflow	–	–	–	–	–	–	–	–	–
IRMA	5	4	92865	88.3301	12269	11.6699	701.2406	106	96.424
LAZYPIPE	162	116	55407	69.2865	24561	30.7135	477.6466	61	99.890
metaSPAdes	305	209	86582	67.7057	41298	32.2943	414.2679	114	99.866
metaviralSPAdes	–	–	–	–	–	–	–	–	–
PEHaplo	125	87	53115	68.0665	24919	31.9335	582.2529	1616	99.819
QuRe	–	–	–	–	–	–	–	–	–
QVG	3	3	138262	99.9986	2	0.0014	46087.3333	1300	99.059
SPAdes	258	176	79767	67.5528	38314	32.4472	457.1494	94	99.880
SSAKE	4	0	0	0.0000	603	100.0000	0.0000	0	0.000
TRACESPipe	4	4	114586	79.6510	29274	20.3490	848.9380	127	96.979
TRACESPipeLite	6	4	109703	65.1873	58586	34.8127	893.3967	62	97.105
V-pipe	5	4	6397	3.9874	154032	96.0126	116.3091	0	98.428
VirGenA	200	196	66371	98.9254	721	1.0746	341.8842	79	99.849
ViSpA	85	77	2498349	98.2043	45683	1.7957	340.7778	96	94.399

Table S87: Results obtained by dnadiff [6] when analysing the reconstruction of DS10. Only one execution of HVRS is represented.

Reconstruction tool	Total seqs	Aligned seqs	Aligned bases	Aligned bases (%)	Unaligned bases	Unaligned bases (%)	Average length	SNPs	Identity
coronaSPAdes	174	121	131458	67.6391	62894	32.3609	1094.1000	132	99.856
Haploflow	–	–	–	–	–	–	–	–	–
IRMA	5	4	123283	88.6596	15769	11.3404	4252.1034	39	98.685
LAZYPIPE	145	102	118542	67.6687	56638	32.3313	1130.3905	109	99.867
metaSPAdes	273	182	121872	67.5060	58663	32.4940	665.9672	154	99.867
metaviralSPAdes	–	–	–	–	–	–	–	–	–
PEHaplo	155	107	123302	67.1550	60306	32.8450	1134.5741	88	99.917
QuRe	–	–	–	–	–	–	–	–	–
QVG	4	4	143858	99.9986	2	0.0014	35964.5000	989	99.317
SPAdes	167	114	129567	67.3212	62894	32.6788	1147.7130	118	99.716
SSAKE	76	56	8350	71.9952	3248	28.0048	149.1071	1	99.988
TRACESPipe	4	4	140451	97.6303	3409	2.3697	6384.1364	177	98.896
TRACESPipeLite	6	4	140336	83.4033	27926	16.5967	6973.5000	149	98.883
V-pipe	5	4	68824	42.9000	91605	57.1000	332.6683	22	94.328
VirGenA	124	123	73477	99.4168	431	0.5832	570.2913	65	99.830
ViSpA	92	82	4391811	98.1675	81984	1.8325	1988.2969	125	96.964

Table S88: Results obtained by dnadiff [6] when analysing the reconstruction of DS11. Only one execution of HVRS is represented.

Reconstruction tool	Total seqs	Aligned seqs	Aligned bases	Aligned bases (%)	Unaligned bases	Unaligned bases (%)	Average length	SNPs	Identity
coronaSPAdes	24	18	137432	67.3709	66561	32.6291	10514.2308	83	99.825
Haploflow	107	72	60173	69.9458	25855	30.0542	823.7361	38	99.933
IRMA	5	4	128055	88.5624	16538	11.4376	21342.8333	4	99.974
LAZYPIPE	20	14	135935	67.2014	66345	32.7986	9703.9286	89	99.911
metaSPAdes	25	19	136999	67.2715	66652	32.7285	6834.5000	47	99.853
metaviralSPAdes	–	–	–	–	–	–	–	–	–
PEHaplo	202	139	150703	67.6733	71989	32.3267	1061.7338	58	99.952
QuRe	–	–	–	–	–	–	–	–	–
QVG	4	4	143858	99.9986	2	0.0014	35964.5000	408	99.715
SPAdes	26	20	137920	67.3839	66758	32.6161	7022.8000	27	99.862
SSAKE	114	80	15281	72.0428	5930	27.9572	191.0125	4	99.974
TRACESPipe	4	4	143756	99.9277	104	0.0723	35939.0000	135	99.861
TRACESPipeLite	6	5	143842	85.4869	24420	14.5131	35911.2500	148	99.861
V-pipe	5	4	126788	79.0306	33641	20.9694	1333.6458	54	96.844
VirGenA	93	92	60436	99.3001	426	0.6999	583.4216	42	99.886
ViSpA	8	7	313106	95.0021	16472	4.9979	29090.5000	3	99.732

Table S89: Results obtained by dnadiff [6] when analysing the reconstruction of DS12. Only one execution of HVRS is represented.

Reconstruction tool	Total seqs	Aligned seqs	Aligned bases	Aligned bases (%)	Unaligned bases	Unaligned bases (%)	Average length	SNPs	Identity
coronaSPAdes	12	10	137312	67.3521	66560	32.6479	13723.1000	53	99.867
Haploflow	118	80	123050	68.3232	57050	31.6768	1523.6579	75	99.932
IRMA	5	4	128255	88.5916	16516	11.4084	25651.0000	0	99.984
LAZYPIPE	10	8	136253	67.2695	66295	32.7305	15137.3333	74	99.938
metaSPAdes	13	11	136713	67.2555	66561	32.7445	10526.3846	55	99.823
metaviralSPAdes	–	–	–	–	–	–	–	–	–
PEHaplo	276	189	159825	67.6522	76420	32.3478	833.5574	38	99.969
QuRe	4	2	224	56.2814	174	43.7186	112.0000	0	100.000
QVG	4	4	143858	99.9986	2	0.0014	35964.5000	225	99.843
SPAdes	19	17	138979	67.5511	66760	32.4489	7294.8947	47	99.918
SSAKE	214	148	34855	69.3190	15427	30.6810	235.5068	8	99.968
TRACESPipe	4	4	143717	99.9006	143	0.0994	35929.2500	147	99.896
TRACESPipeLite	6	5	144132	85.6593	24130	14.3407	35933.2500	128	99.903
V-pipe	5	4	142389	88.7552	18040	11.2448	15821.0000	145	99.134
VirGenA	72	71	59108	99.2894	423	0.7106	741.7722	46	99.864
ViSpA	6	5	180530	91.6373	16475	8.3627	28693.4000	150	99.889

Table S90: Results obtained by dnadiff [6] when analysing the reconstruction of DS13. Only one execution of HVRS is represented.

Reconstruction tool	Total seqs	Aligned seqs	Aligned bases	Aligned bases (%)	Unaligned bases	Unaligned bases (%)	Average length	SNPs	Identity
coronaSPAdes	14	12	137731	67.4332	66517	32.5668	12662.9091	44	99.821
Haploflow	42	32	142611	68.5547	65414	31.4453	4715.4828	92	99.913
IRMA	5	4	128300	88.5995	16509	11.4005	25660.2000	0	100.000
LAZYPIPE	7	5	136414	67.2616	66397	32.7384	17129.5000	56	99.932
metaSPAdes	13	11	136759	67.2772	66518	32.7228	11396.5833	59	99.861
metaviralSPAdes	–	–	–	–	–	–	–	–	–
PEHaplo	305	211	173528	69.4977	76161	30.5023	834.8408	87	99.937
QuRe	4	3	695	87.8635	96	12.1365	231.6667	0	100.000
QVG	4	4	143858	99.9986	2	0.0014	35964.5000	157	99.892
SPAdes	21	19	145210	68.5506	66619	31.4494	6673.6190	36	99.906
SSAKE	373	266	84777	69.9457	36427	30.0543	320.4580	28	99.964
TRACESPipe	4	4	143825	99.9757	35	0.0243	35956.2500	143	99.902
TRACESPipeLite	6	4	143716	85.4120	24546	14.5880	28808.8000	123	99.900
V-pipe	5	4	143294	89.3193	17135	10.6807	35823.5000	128	99.765
VirGenA	66	65	57306	98.8086	691	1.1914	717.4805	45	99.882
ViSpA	5	4	143592	89.6951	16497	10.3049	35898.0000	153	99.893

Table S91: Results obtained by dnadiff [6] when analysing the reconstruction of DS14. Only one execution of HVRS is represented.

Reconstruction tool	Total seqs	Aligned seqs	Aligned bases	Aligned bases (%)	Unaligned bases	Unaligned bases (%)	Average length	SNPs	Identity
coronaSPAdes	10	8	137186	67.3127	66618	32.6873	19568.8571	63	99.910
Haploflow	30	23	147275	68.9344	66370	31.0656	6243.7273	80	99.920
IRMA	5	4	128330	88.5804	16544	11.4196	25666.2000	0	100.000
LAZYPIPE	10	8	136874	67.3212	66441	32.6788	15232.7778	61	99.915
metaSPAdes	12	10	136892	67.2655	66618	32.7345	10740.7692	31	99.823
metaviralSPAdes	–	–	–	–	–	–	–	–	–
PEHaplo	289	184	173544	67.0318	85354	32.9682	942.4171	61	99.954
QuRe	4	3	1460	82.7195	305	17.2805	486.6667	4	99.722
QVG	4	4	143858	99.9986	2	0.0014	35964.5000	150	99.893
SPAdes	25	23	146599	68.7877	66519	31.2123	6707.6190	24	99.938
SSAKE	489	345	134050	69.4322	59016	30.5678	396.7190	25	99.969
TRACESPipe	4	4	143798	99.9562	63	0.0438	35949.5000	136	99.903
TRACESPipeLite	6	5	144010	85.5868	24252	14.4132	35949.0000	161	99.885
V-pipe	5	4	143451	89.4171	16978	10.5829	35862.7500	134	99.890
VirGenA	68	67	57858	99.7036	172	0.2964	716.9630	33	99.899
ViSpA	5	4	143728	89.6904	16521	10.3096	35932.0000	142	99.901

Table S92: Results obtained by dnadiff [6] when analysing the reconstruction of DS15. Only one execution of HVRS is represented.

Reconstruction tool	Total seqs	Aligned seqs	Aligned bases	Aligned bases (%)	Unaligned bases	Unaligned bases (%)	Average length	SNPs	Identity
coronaSPAdes	12	10	137407	67.3409	66640	32.6591	17112.1250	52	99.916
Haploflow	20	16	147883	68.9788	66506	31.0212	9187.7333	103	99.908
IRMA	5	4	128341	88.5752	16554	11.4248	25668.4000	0	100.000
LAZYPIPE	8	6	136283	67.2047	66505	32.7953	17121.3750	66	99.929
metaSPAdes	13	11	136969	67.2703	66641	32.7297	10737.5385	40	99.836
metaviralSPAdes	–	–	–	–	–	–	–	–	–
PEHaplo	313	207	197805	67.5367	95080	32.4633	972.9206	66	99.952
QuRe	5	4	1017	82.4818	216	17.5182	254.2500	2	99.804
QVG	4	4	143858	99.9986	2	0.0014	35964.5000	148	99.893
SPAdes	27	25	141613	68.0325	66542	31.9675	5893.5833	13	99.947
SSAKE	404	288	149793	68.6158	68514	31.3842	557.5290	39	99.915
TRACESPipe	4	4	143764	99.9333	96	0.0667	35941.0000	140	99.904
TRACESPipeLite	6	5	144130	85.6581	24132	14.3419	35941.7500	111	99.920
V-pipe	5	4	143381	89.3735	17048	10.6265	35845.2500	165	99.887
VirGenA	69	68	55640	98.9948	565	1.0052	713.9333	43	99.888
ViSpA	5	4	143681	89.6824	16530	10.3176	35920.2500	166	99.884

Table S93: Results obtained by dnadiff [6] when analysing the reconstruction of DS16. Only one execution of HVRS is represented.

Reconstruction tool	Total seqs	Aligned seqs	Aligned bases	Aligned bases (%)	Unaligned bases	Unaligned bases (%)	Average length	SNPs	Identity
coronaSPAdes	10	8	137191	67.3023	66652	32.6977	19569.5714	47	99.923
Haploflow	12	10	145468	68.6947	66292	31.3053	13811.4000	113	99.876
IRMA	5	4	128345	88.5712	16561	11.4288	25669.0000	0	100.000
LAZYPIPE	9	7	136903	67.2967	66529	32.7033	19557.5714	53	99.942
metaSPAdes	12	10	136897	67.2551	66652	32.7449	10741.1538	24	99.828
metaviralSPAdes	2	0	0	0.0000	66553	100.0000	0.0000	0	0.000
PEHaplo	220	149	222518	69.1679	99189	30.8321	1489.3525	64	99.938
QuRe	8	7	25124	97.6941	593	2.3059	2470.2500	32	99.676
QVG	4	4	143858	99.9986	2	0.0014	35964.5000	147	99.894
SPAdes	27	25	146744	68.7980	66553	31.2020	5893.7083	22	99.940
SSAKE	223	164	155802	68.7576	70794	31.2424	1224.4667	10	99.993
TRACESPipe	4	4	143813	99.9673	47	0.0327	35953.2500	151	99.893
TRACESPipeLite	6	4	143814	85.4703	24448	14.5297	35953.5000	138	99.903
V-pipe	5	4	143515	89.4570	16914	10.5430	35878.7500	132	99.904
VirGenA	55	55	58585	99.2411	448	0.7589	804.4429	40	99.844
ViSpA	5	4	143700	89.6679	16558	10.3321	35925.0000	164	99.884

Table S94: Results obtained by dnadiff [6] when analysing the reconstruction of DS17. Only one execution of HVRS is represented.

Reconstruction tool	Total seqs	Aligned seqs	Aligned bases	Aligned bases (%)	Unaligned bases	Unaligned bases (%)	Average length	SNPs	Identity
coronaSPAdes	259	174	84390	69.0081	37900	30.9919	485.0000	95	99.886
Haploflow	–	–	–	–	–	–	–	–	–
IRMA	5	4	96234	88.9293	11980	11.0707	730.7576	112	95.775
LAZYPIPE	159	109	58380	70.4596	24476	29.5404	526.9550	29	99.949
metaSPAdes	306	212	92930	70.0365	39758	29.9635	436.3726	83	99.909
metaviralSPAdes	–	–	–	–	–	–	–	–	–
PEHaplo	131	94	60152	71.9005	23508	28.0995	627.6632	522	99.878
QuRe	–	–	–	–	–	–	–	–	–
QVG	–	–	–	–	–	–	–	–	–
SPAdes	–	–	–	–	–	–	–	–	–
SSAKE	2	2	251	100.0000	0	0.0000	125.5000	0	100.000
TRACESPipe	4	4	114023	79.2597	29837	20.7403	857.2960	15	97.482
TRACESPipeLite	6	4	113527	67.4704	54735	32.5296	930.9835	15	96.411
V-pipe	5	3	5686	3.5442	154743	96.4558	132.2326	0	95.912
VirGenA	214	210	67931	99.5472	309	0.4528	321.8883	55	99.867
ViSpA	86	75	2357091	97.7255	54861	2.2745	338.8186	54	96.054

Table S95: Results obtained by dnadiff [6] when analysing the reconstruction of DS18. Only one execution of HVRS is represented.

Reconstruction tool	Total seqs	Aligned seqs	Aligned bases	Aligned bases (%)	Unaligned bases	Unaligned bases (%)	Average length	SNPs	Identity
coronaSPAdes	272	182	80543	66.9585	39745	33.0415	444.3128	215	99.730
Haploflow	–	–	–	–	–	–	–	–	–
IRMA	5	4	91913	88.0334	12494	11.9666	720.5312	109	95.614
LAZYPIPE	157	113	52867	69.7822	22893	30.2178	467.8496	119	99.741
metaSPAdes	327	219	87274	66.7692	43436	33.2308	399.8945	152	99.818
metaviralSPAdes	–	–	–	–	–	–	–	–	–
PEHaplo	118	92	53767	73.0381	19848	26.9619	591.6413	2702	99.631
QuRe	–	–	–	–	–	–	–	–	–
QVG	3	3	138473	100.0000	0	0.0000	34647.7500	4037	96.924
SPAdes	–	–	–	–	–	–	–	–	–
SSAKE	1	1	167	100.0000	0	0.0000	167.0000	0	100.000
TRACESPipe	4	4	112911	78.4873	30948	21.5127	899.5920	264	96.898
TRACESPipeLite	6	4	95121	56.5940	72955	43.4060	617.0987	120	97.176
V-pipe	5	3	6746	4.2050	153683	95.7950	140.5417	6	95.968
VirGenA	211	210	67948	99.6393	246	0.3607	323.3269	112	99.808
ViSpA	81	72	2367542	97.8694	51541	2.1306	328.8703	174	94.834

Table S96: Results obtained by dnadiff [6] when analysing the reconstruction of DS19. Only one execution of HVRS is represented.

Reconstruction tool	Total seqs	Aligned seqs	Aligned bases	Aligned bases (%)	Unaligned bases	Unaligned bases (%)	Average length	SNPs	Identity
coronaSPAdes	274	184	83666	66.9998	41209	33.0002	460.4348	164	99.656
Haploflow	–	–	–	–	–	–	–	–	–
IRMA	5	4	94356	88.4502	12321	11.5498	674.8500	126	96.223
LAZYPIPE	182	128	59305	68.7666	26936	31.2334	464.5349	60	99.773
metaSPAdes	323	219	90359	67.1619	44180	32.8381	415.5000	126	99.747
metaviralSPAdes	–	–	–	–	–	–	–	–	–
PEHaplo	138	97	56919	69.0530	25509	30.9470	594.6146	1272	99.598
QuRe	–	–	–	–	–	–	–	–	–
QVG	4	4	143858	99.9986	2	0.0014	35964.5000	6858	95.231
SPAdes	–	–	–	–	–	–	–	–	–
SSAKE	–	–	–	–	–	–	–	–	–
TRACESPipe	4	4	117131	81.4207	26728	18.5793	974.2667	555	96.286
TRACESPipeLite	6	4	53684	31.9080	114562	68.0920	331.5000	73	98.109
V-pipe	5	4	6608	4.1190	153821	95.8810	132.1600	1	95.875
VirGenA	195	195	64904	99.9246	49	0.0754	321.3941	85	99.790
ViSpA	86	79	2326634	98.1353	44210	1.8647	331.6303	299	94.594

Table S97: Results obtained by dnadiff [6] when analysing the reconstruction of DS20. Only one execution of HVRS is represented.

Reconstruction tool	Total seqs	Aligned seqs	Aligned bases	Aligned bases (%)	Unaligned bases	Unaligned bases (%)	Average length	SNPs	Identity
coronaSPAdes	265	176	80299	68.5566	36829	31.4434	457.4971	245	99.694
Haploflow	–	–	–	–	–	–	–	–	–
IRMA	5	4	93433	88.7598	11832	11.2402	628.2013	120	96.533
LAZYPIPE	154	111	52118	71.5848	20688	28.4152	468.6667	48	99.831
metaSPAdes	327	221	90222	69.0816	40380	30.9184	409.6575	163	99.805
metaviralSPAdes	–	–	–	–	–	–	–	–	–
PEHaplo	125	93	54594	72.7532	20446	27.2468	583.7684	2658	99.720
QuRe	–	–	–	–	–	–	–	–	–
QVG	4	4	143860	100.0000	0	0.0000	35965.0000	9676	93.217
SPAdes	–	–	–	–	–	–	–	–	–
SSAKE	5	4	609	78.5806	166	21.4194	152.2500	0	100.000
TRACESPipe	4	4	110655	77.0267	33003	22.9733	791.9023	580	96.547
TRACESPipeLite	6	2	9730	5.7896	158330	94.2104	237.3171	3	98.918
V-pipe	5	4	7649	4.7678	152780	95.2322	141.6481	7	96.532
VirGenA	206	201	64285	99.3755	404	0.6245	319.2150	70	99.812
ViSpA	77	69	2115952	98.0809	41402	1.9191	305.1036	224	95.150

Table S98: Results obtained by dnadiff [6] when analysing the reconstruction of DS21. Only one execution of HVRS is represented.

Reconstruction tool	Total seqs	Aligned seqs	Aligned bases	Aligned bases (%)	Unaligned bases	Unaligned bases (%)	Average length	SNPs	Identity
coronaSPAdes	279	190	85483	68.7588	38840	31.2412	450.5550	143	99.663
Haploflow	–	–	–	–	–	–	–	–	–
IRMA	5	4	92996	88.2868	12338	11.7132	656.0352	80	96.255
LAZYPIPE	169	111	54996	68.1158	25743	31.8842	495.4595	75	99.864
metaSPAdes	329	221	92276	68.3060	42816	31.6940	418.2387	104	99.743
metaviralSPAdes	–	–	–	–	–	–	–	–	–
PEHaplo	138	94	57468	68.6620	26229	31.3380	613.8602	2053	99.934
QuRe	–	–	–	–	–	–	–	–	–
QVG	4	4	143816	99.9694	44	0.0306	35953.2500	12401	91.349
SPAdes	–	–	–	–	–	–	–	–	–
SSAKE	–	–	–	–	–	–	–	–	–
TRACESPipe	4	4	98066	68.1667	45796	31.8333	694.0821	1200	95.608
TRACESPipeLite	6	2	2614	1.5536	165637	98.4464	201.0769	0	99.886
V-pipe	5	4	6352	3.9594	154077	96.0406	129.6327	5	94.181
VirGenA	198	193	64608	99.0814	599	0.9186	330.9643	95	99.732
ViSpA	69	58	1514235	95.9056	64645	4.0944	278.6948	248	94.797

Table S99: Results obtained by dnadiff [6] when analysing the reconstruction of DS22. Only one execution of HVRS is represented.

Reconstruction tool	Total seqs	Aligned seqs	Aligned bases	Aligned bases (%)	Unaligned bases	Unaligned bases (%)	Average length	SNPs	Identity
coronaSPAdes	263	184	82843	68.3512	38359	31.6488	450.2337	126	99.848
Haploflow	–	–	–	–	–	–	–	–	–
IRMA	5	4	91779	88.5675	11847	11.4325	651.7376	114	96.015
LAZYPIPE	163	111	54566	67.1888	26647	32.8112	491.5856	36	99.934
metaSPAdes	314	224	91343	69.1903	40674	30.8097	407.7812	85	99.902
metaviralSPAdes	–	–	–	–	–	–	–	–	–
PEHaplo	133	93	55541	68.0008	26136	31.9992	578.8958	1950	99.939
QuRe	–	–	–	–	–	–	–	–	–
QVG	4	4	143860	100.0000	0	0.0000	35965.0000	15309	89.322
SPAdes	–	–	–	–	–	–	–	–	–
SSAKE	3	2	369	70.8253	152	29.1747	184.5000	0	100.000
TRACESPipe	4	4	74753	52.0354	68905	47.9646	528.3147	1294	96.259
TRACESPipeLite	6	1	221	0.1313	168041	99.8687	221.0000	0	100.000
V-pipe	5	4	5301	3.3043	155128	96.6957	117.8000	5	97.153
VirGenA	208	203	65807	99.5176	319	0.4824	318.0628	99	99.833
ViSpA	49	39	906889	94.2327	55504	5.7673	249.4409	175	95.431

Table S100: Results obtained by dnadiff [6] when analysing the reconstruction of DS23. Only one execution of HVRS is represented.

Reconstruction tool	Total seqs	Aligned seqs	Aligned bases	Aligned bases (%)	Unaligned bases	Unaligned bases (%)	Average length	SNPs	Identity
coronaSPAdes	265	188	85953	68.8164	38949	31.1836	455.6402	149	99.794
Haploflow	1	1	611	100.0000	0	0.0000	611.0000	1	99.840
IRMA	5	4	89102	88.4492	11636	11.5508	507.0511	97	96.971
LAZYPIPE	178	121	58166	66.6445	29112	33.3555	480.7107	67	99.885
metaSPAdes	307	219	92416	69.1649	41201	30.8351	421.9909	96	99.895
metaviralSPAdes	–	–	–	–	–	–	–	–	–
PEHaplo	134	89	53334	64.8903	28857	35.1097	599.2584	1047	99.912
QuRe	–	–	–	–	–	–	–	–	–
QVG	4	4	143818	99.9708	42	0.0292	35954.5000	18093	87.202
SPAdes	–	–	–	–	–	–	–	–	–
SSAKE	3	1	124	32.7177	255	67.2823	124.0000	0	100.000
TRACESPipe	4	4	66560	46.2820	77254	53.7180	469.6993	1082	96.453
TRACESPipeLite	5	0	0	0.0000	160429	100.0000	0.0000	0	0.000
V-pipe	5	3	2539	1.5826	157890	98.4174	133.6316	0	92.188
VirGenA	210	205	66928	99.3793	418	0.6207	320.5311	71	99.849
ViSpA	24	15	173066	79.9950	43280	20.0050	205.2892	100	95.819

Table S101: Results obtained by dnadiff [6] when analysing the reconstruction of DS24. Only one execution of HVRS is represented.

Reconstruction tool	Total seqs	Aligned seqs	Aligned bases	Aligned bases (%)	Unaligned bases	Unaligned bases (%)	Average length	SNPs	Identity
coronaSPAdes	280	190	86772	69.6041	37893	30.3959	456.6947	108	99.874
Haploflow	1	1	630	100.0000	0	0.0000	630.0000	1	99.840
IRMA	5	4	86789	87.7667	12097	12.2333	489.2697	130	97.010
LAZYPIPE	228	154	67093	69.8609	28945	30.1391	435.6688	32	99.952
metaSPAdes	321	214	91855	68.8594	41540	31.1406	429.2290	108	99.880
metaviralSPAdes	–	–	–	–	–	–	–	–	–
PEHaplo	139	98	57283	71.1661	23209	28.8339	567.5248	2425	99.915
QuRe	–	–	–	–	–	–	–	–	–
QVG	4	4	143851	99.9937	9	0.0063	35962.7500	20734	85.427
SPAdes	–	–	–	–	–	–	–	–	–
SSAKE	7	3	507	42.3559	690	57.6441	169.0000	0	100.000
TRACESPipe	4	4	56696	39.4127	87156	60.5873	443.2171	1107	96.089
TRACESPipeLite	5	0	0	0.0000	160227	100.0000	0.0000	0	0.000
V-pipe	5	4	2595	1.6175	157834	98.3825	108.1250	0	96.533
VirGenA	222	214	68076	99.2376	523	0.7624	311.0548	79	99.853
ViSpA	15	4	47037	53.1012	41543	46.8988	157.9459	77	97.678

Table S102: Results obtained by dnadiff [6] when analysing the reconstruction of DS25. Only one execution of HVRS is represented.

Reconstruction tool	Total seqs	Aligned seqs	Aligned bases	Aligned bases (%)	Unaligned bases	Unaligned bases (%)	Average length	SNPs	Identity
coronaSPAdes	162	105	130031	66.9766	64113	33.0234	1227.5377	84	99.832
Haploflow	5	4	4374	88.7581	554	11.2419	1093.5000	0	100.000
IRMA	5	4	124223	88.6807	15856	11.3193	5916.0952	54	98.431
LAZYPIPE	150	97	119840	67.0722	58833	32.9278	1235.4639	20	99.984
metaSPAdes	265	171	121006	66.7513	60273	33.2487	703.5233	96	99.888
metaviralSPAdes	–	–	–	–	–	–	–	–	–
PEHaplo	159	106	129864	67.4051	62798	32.5949	1201.2991	41	99.951
QuRe	–	–	–	–	–	–	–	–	–
QVG	–	–	–	–	–	–	–	–	–
SPAdes	163	106	130203	67.0055	64114	32.9945	1217.6729	55	99.851
SSAKE	93	71	10866	75.8322	3463	24.1678	152.8028	1	99.982
TRACESPipe	4	4	140919	97.9557	2941	2.0443	7045.9500	7	98.920
TRACESPipeLite	6	5	142456	84.6632	25806	15.3368	12932.5455	2	98.858
V-pipe	5	4	68320	42.5858	92109	57.4142	321.9183	5	95.046
VirGenA	104	104	81489	99.9375	51	0.0625	741.0472	44	99.916
ViSpA	89	76	3628951	97.7545	83359	2.2455	1843.3582	34	96.987

Table S103: Results obtained by dnadiff [6] when analysing the reconstruction of DS26. Only one execution of HVRS is represented.

Reconstruction tool	Total seqs	Aligned seqs	Aligned bases	Aligned bases (%)	Unaligned bases	Unaligned bases (%)	Average length	SNPs	Identity
coronaSPAdes	162	114	133959	67.7913	63646	32.2087	1219.8532	186	99.737
Haploflow	1	0	0	0.0000	520	100.0000	0.0000	0	0.000
IRMA	5	4	123337	88.5437	15958	11.4563	5377.6087	48	98.297
LAZYPIPE	154	107	124737	67.7171	59466	32.2829	1202.6505	56	99.847
metaSPAdes	308	217	110049	67.5524	52860	32.4476	515.9275	110	99.891
metaviralSPAdes	–	–	–	–	–	–	–	–	–
PEHaplo	162	112	140459	68.3416	65066	31.6584	1214.0741	336	99.730
QuRe	–	–	–	–	–	–	–	–	–
QVG	4	4	143860	100.0000	0	0.0000	35965.0000	3136	97.748
SPAdes	158	110	131221	67.3384	63647	32.6616	1215.7477	247	99.807
SSAKE	78	54	8741	70.7544	3613	29.2456	161.8704	0	100.000
TRACESPipe	4	4	140829	97.8924	3032	2.1076	7041.1500	502	98.527
TRACESPipeLite	6	4	135071	80.2742	33191	19.7258	2811.5532	215	98.103
V-pipe	5	4	65656	40.9253	94773	59.0747	319.8768	58	94.635
VirGenA	126	124	66483	99.2891	476	0.7109	503.2308	62	99.783
ViSpA	86	78	3828546	98.3742	63272	1.6258	1925.1970	128	97.005

Table S104: Results obtained by dnadiff [6] when analysing the reconstruction of DS27. Only one execution of HVRS is represented.

Reconstruction tool	Total seqs	Aligned seqs	Aligned bases	Aligned bases (%)	Unaligned bases	Unaligned bases (%)	Average length	SNPs	Identity
coronaSPAdes	165	121	138722	68.4939	63810	31.5061	1157.6218	176	99.790
Haploflow	–	–	–	–	–	–	–	–	–
IRMA	5	4	123765	88.5883	15943	11.4117	4760.9615	50	98.694
LAZYPIPE	150	107	126613	67.8032	60123	32.1968	1175.1321	60	99.934
metaSPAdes	315	220	120372	68.1122	56354	31.8878	547.9816	142	99.854
metaviralSPAdes	–	–	–	–	–	–	–	–	–
PEHaplo	170	120	137977	68.6006	63154	31.3994	1145.8017	348	99.603
QuRe	–	–	–	–	–	–	–	–	–
QVG	4	4	143858	99.9986	2	0.0014	35964.5000	5172	96.400
SPAdes	160	116	135761	68.0264	63810	31.9736	1182.3304	188	99.772
SSAKE	75	51	8352	69.7104	3629	30.2896	163.7647	1	99.988
TRACESPipe	4	4	142304	98.9177	1557	1.0823	10164.5714	670	98.687
TRACESPipeLite	6	4	85311	50.7013	82951	49.2987	543.8258	111	97.683
V-pipe	5	4	63188	39.3869	97241	60.6131	284.2133	61	95.529
VirGenA	120	120	74055	99.6622	251	0.3378	563.0385	158	99.568
ViSpA	78	70	3285350	97.6003	80777	2.3997	2079.4355	620	96.542

Table S105: Results obtained by dnadiff [6] when analysing the reconstruction of DS28. Only one execution of HVRS is represented.

Reconstruction tool	Total seqs	Aligned seqs	Aligned bases	Aligned bases (%)	Unaligned bases	Unaligned bases (%)	Average length	SNPs	Identity
coronaSPAdes	156	103	136938	68.1216	64082	31.8784	1340.0294	97	99.840
Haploflow	–	–	–	–	–	–	–	–	–
IRMA	5	4	122469	88.3985	16073	11.6015	4224.1724	78	98.519
LAZYPIPE	149	97	127856	68.0183	60117	31.9817	1318.1031	24	99.940
metaSPAdes	338	230	107948	67.5093	51953	32.4907	469.6594	105	99.901
metaviralSPAdes	–	–	–	–	–	–	–	–	–
PEHaplo	166	115	158080	71.5191	62952	28.4809	1281.1509	323	99.525
QuRe	–	–	–	–	–	–	–	–	–
QVG	4	4	143860	100.0000	0	0.0000	35965.0000	7602	94.671
SPAdes	156	103	136919	68.1172	64086	31.8828	1337.9903	61	99.850
SSAKE	65	45	6832	69.8569	2948	30.1431	151.8222	0	100.000
TRACESPipe	4	4	141043	98.0432	2815	1.9568	7835.5556	1322	98.045
TRACESPipeLite	6	3	19599	11.6479	148663	88.3521	283.7391	9	98.176
V-pipe	5	4	63775	39.7528	96654	60.2472	302.8990	182	94.928
VirGenA	126	125	71184	99.2277	554	0.7723	544.1439	126	99.474
ViSpA	90	76	3190744	95.9756	133794	4.0244	1778.5270	911	96.581

Table S106: Results obtained by dnadiff [6] when analysing the reconstruction of DS29. Only one execution of HVRS is represented.

Reconstruction tool	Total seqs	Aligned seqs	Aligned bases	Aligned bases (%)	Unaligned bases	Unaligned bases (%)	Average length	SNPs	Identity
coronaSPAdes	161	111	134153	67.5633	64406	32.4367	1208.5856	88	99.934
Haploflow	1	0	0	0.0000	511	100.0000	0.0000	0	0.000
IRMA	5	4	122742	88.4933	15960	11.5067	3610.6765	47	98.493
LAZYPIPE	160	111	127975	67.9150	60459	32.0850	1152.9279	17	99.987
metaSPAdes	317	211	100064	66.7129	49928	33.2871	473.7048	90	99.908
metaviralSPAdes	–	–	–	–	–	–	–	–	–
PEHaplo	170	120	133304	67.6018	63886	32.3982	1115.9496	212	99.763
QuRe	–	–	–	–	–	–	–	–	–
QVG	4	4	143813	99.9673	47	0.0327	35953.2500	9748	93.194
SPAdes	163	113	135271	67.7449	64406	32.2551	1197.0885	45	99.965
SSAKE	68	50	7572	72.9550	2807	27.0450	151.4400	0	100.000
TRACESPipe	4	4	136260	94.7171	7600	5.2829	3696.7222	1149	97.348
TRACESPipeLite	6	2	2485	1.4769	165777	98.5231	225.9091	0	98.534
V-pipe	5	4	63104	39.3345	97325	60.6655	299.6019	187	94.365
VirGenA	121	121	63149	99.3112	438	0.6888	490.2266	94	99.752
ViSpA	107	97	4450371	97.9400	93605	2.0600	1215.4554	807	95.473

Table S107: Results obtained by dnadiff [6] when analysing the reconstruction of DS30. Only one execution of HVRS is represented.

Reconstruction tool	Total seqs	Aligned seqs	Aligned bases	Aligned bases (%)	Unaligned bases	Unaligned bases (%)	Average length	SNPs	Identity
coronaSPAdes	167	114	135762	68.5656	62241	31.4344	1190.8947	72	99.946
Haploflow	1	1	540	100.0000	0	0.0000	540.0000	0	100.000
IRMA	5	4	123633	88.6017	15905	11.3983	3254.7895	41	98.577
LAZYPIPE	155	110	126982	69.1808	56569	30.8192	1154.3818	24	99.981
metaSPAdes	313	210	116656	68.2822	54188	31.7178	555.5048	90	99.918
metaviralSPAdes	–	–	–	–	–	–	–	–	–
PEHaplo	168	112	135846	67.8867	64261	32.1133	1205.4414	36	99.943
QuRe	–	–	–	–	–	–	–	–	–
QVG	4	4	143860	100.0000	0	0.0000	35965.0000	12704	91.129
SPAdes	167	114	135875	68.5804	62250	31.4196	1198.9027	89	99.931
SSAKE	81	52	8211	62.9581	4831	37.0419	157.9038	1	99.988
TRACESPipe	4	4	131760	91.5897	12099	8.4103	2787.9348	1352	97.630
TRACESPipeLite	6	0	0	0.0000	168262	100.0000	0.0000	0	0.000
V-pipe	5	4	55920	34.8565	104509	65.1435	273.0493	173	94.003
VirGenA	112	109	61705	99.4039	370	0.5961	524.6864	32	99.755
ViSpA	133	128	5342677	98.6114	75231	1.3886	724.9864	688	94.350

Table S108: Results obtained by dnadiff [6] when analysing the reconstruction of DS31. Only one execution of HVRS is represented.

Reconstruction tool	Total seqs	Aligned seqs	Aligned bases	Aligned bases (%)	Unaligned bases	Unaligned bases (%)	Average length	SNPs	Identity
coronaSPAdes	171	117	136962	68.3948	63290	31.6052	1170.6154	75	99.943
Haploflow	1	1	533	100.0000	0	0.0000	533.0000	0	100.000
IRMA	5	4	122039	88.6242	15665	11.3758	2491.7143	40	98.481
LAZYPIPE	164	112	127384	68.2292	59316	31.7708	1137.3571	20	99.984
metaSPAdes	322	227	104046	68.4747	47902	31.5253	458.3524	84	99.913
metaviralSPAdes	–	–	–	–	–	–	–	–	–
PEHaplo	162	112	133603	68.9795	60082	31.0205	1192.8839	364	99.956
QuRe	–	–	–	–	–	–	–	–	–
QVG	4	4	143820	99.9708	42	0.0292	35955.0000	16044	88.634
SPAdes	171	117	137018	68.4033	63291	31.5967	1171.0940	44	99.966
SSAKE	61	43	6817	70.8628	2803	29.1372	158.5349	0	100.000
TRACESPipe	4	4	128565	89.3681	15295	10.6319	2272.0714	1812	97.055
TRACESPipeLite	6	0	0	0.0000	168262	100.0000	0.0000	0	0.000
V-pipe	5	4	45781	28.5366	114648	71.4634	235.2092	162	93.088
VirGenA	105	105	70354	99.2929	501	0.7071	602.2564	34	99.883
ViSpA	107	96	2925222	96.1664	116612	3.8336	419.7128	292	94.943

Table S109: Results obtained by dnadiff [6] when analysing the reconstruction of DS32. Only one execution of HVRS is represented.

Reconstruction tool	Total seqs	Aligned seqs	Aligned bases	Aligned bases (%)	Unaligned bases	Unaligned bases (%)	Average length	SNPs	Identity
coronaSPAdes	173	116	137962	68.5107	63411	31.4893	1189.3276	66	99.951
Haploflow	–	–	–	–	–	–	–	–	–
IRMA	5	4	121836	88.1662	16353	11.8338	1792.6618	58	98.531
LAZYPIPE	172	118	130397	68.9563	58704	31.0437	1105.0593	19	99.986
metaSPAdes	319	218	121490	69.5110	53288	30.4890	557.2936	75	99.936
metaviralSPAdes	–	–	–	–	–	–	–	–	–
PEHaplo	162	112	134948	68.6758	61552	31.3242	1204.8929	34	99.963
QuRe	–	–	–	–	–	–	–	–	–
QVG	4	4	143849	99.9937	9	0.0063	35962.2500	19218	86.483
SPAdes	173	116	138178	68.5437	63413	31.4563	1191.1897	44	99.967
SSAKE	74	46	6815	60.2191	4502	39.7809	148.1522	0	100.000
TRACESPipe	4	4	116877	81.2696	26937	18.7304	1523.8816	1406	97.524
TRACESPipeLite	6	0	0	0.0000	168289	100.0000	0.0000	0	0.000
V-pipe	5	4	32649	20.3511	127780	79.6489	195.5030	72	93.219
VirGenA	129	127	62578	98.8204	747	1.1796	453.0647	63	99.640
ViSpA	54	46	952339	91.2954	90801	8.7046	263.7931	249	95.949

Table S110: Results obtained by dnadiff [6] when analysing the reconstruction of DS33. Only one execution of HVRS is represented.

Reconstruction tool	Total seqs	Aligned seqs	Aligned bases	Aligned bases (%)	Unaligned bases	Unaligned bases (%)	Average length	SNPs	Identity
coronaSPAdes	27	20	135645	67.0872	66547	32.9128	6464.4762	10	99.908
Haploflow	106	72	59512	70.5930	24791	29.4070	826.5556	0	100.000
IRMA	5	4	128071	88.5459	16567	11.4541	30076.5000	8	99.947
LAZYPIPE	24	17	134839	67.0794	66175	32.9206	6749.3000	4	99.995
metaSPAdes	27	20	135717	67.0989	66547	32.9011	6467.3333	8	99.858
metaviralSPAdes	–	–	–	–	–	–	–	–	–
PEHaplo	208	144	152091	67.2642	74019	32.7358	1041.5972	16	99.983
QuRe	3	1	71	47.0199	80	52.9801	71.0000	0	100.000
QVG	–	–	–	–	–	–	–	–	–
SPAdes	26	19	135760	67.1059	66547	32.8941	6473.8571	5	99.863
SSAKE	120	83	15779	69.9052	6793	30.0948	190.1084	1	99.994
TRACESPipe	4	4	143815	99.9687	45	0.0313	35953.7500	0	99.957
TRACESPipeLite	6	5	143992	85.5761	24270	14.4239	35899.5000	0	99.909
V-pipe	5	4	129232	80.5540	31197	19.4460	1621.7564	5	96.583
VirGenA	85	85	68280	98.8949	763	1.1051	727.4362	21	99.894
ViSpA	11	10	437940	96.4088	16313	3.5912	27190.6667	4	99.644

Table S111: Results obtained by dnadiff [6] when analysing the reconstruction of DS34. Only one execution of HVRS is represented.

Reconstruction tool	Total seqs	Aligned seqs	Aligned bases	Aligned bases (%)	Unaligned bases	Unaligned bases (%)	Average length	SNPs	Identity
coronaSPAdes	33	23	141458	68.0244	66494	31.9756	6417.0909	55	99.960
Haploflow	103	69	56298	70.3312	23749	29.6688	819.5735	44	99.921
IRMA	5	4	128125	88.6408	16419	11.3592	25625.2000	1	99.951
LAZYPIPE	32	22	142705	68.3600	66050	31.6400	8057.4706	148	99.856
metaSPAdes	39	29	141614	68.0483	66494	31.9517	5048.4643	40	99.924
metaviralSPAdes	–	–	–	–	–	–	–	–	–
PEHaplo	240	170	170017	69.9345	73092	30.0655	984.4410	232	99.753
QuRe	1	1	94	100.0000	0	0.0000	94.0000	0	100.000
QVG	4	4	143860	100.0000	0	0.0000	35965.0000	1388	98.963
SPAdes	34	24	142811	68.2304	66496	31.7696	6772.8571	20	99.918
SSAKE	118	76	13291	62.4548	7990	37.5452	174.8816	1	99.993
TRACESPipe	4	4	143608	99.8234	254	0.1766	28793.8000	511	99.591
TRACESPipeLite	6	4	142274	84.5550	25988	15.4450	11856.1667	426	99.119
V-pipe	5	4	126584	78.9034	33845	21.0966	1301.9574	297	96.547
VirGenA	86	85	59524	99.1059	537	0.8941	643.8000	80	99.750
ViSpA	11	9	289066	91.8962	25491	8.1038	17846.3750	470	99.476

Table S112: Results obtained by dnadiff [6] when analysing the reconstruction of DS35. Only one execution of HVRS is represented.

Reconstruction tool	Total seqs	Aligned seqs	Aligned bases	Aligned bases (%)	Unaligned bases	Unaligned bases (%)	Average length	SNPs	Identity
coronaSPAdes	25	17	143450	68.3375	66464	31.6625	8438.2353	28	99.976
Haploflow	101	69	52541	69.0312	23571	30.9688	752.6714	34	99.907
IRMA	5	4	127959	88.5529	16541	11.4471	25591.8000	7	99.851
LAZYPIPE	27	18	142502	68.3135	66098	31.6865	7518.6842	9	99.876
metaSPAdes	30	21	143105	68.2580	66548	31.7420	6814.5238	29	99.974
metaviralSPAdes	–	–	–	–	–	–	–	–	–
PEHaplo	221	160	162054	68.8534	73307	31.1466	1020.8312	114	99.787
QuRe	–	–	–	–	–	–	–	–	–
QVG	4	4	143858	99.9986	2	0.0014	35964.5000	2184	98.482
SPAdes	28	20	143379	68.3268	66464	31.6732	9595.7333	2	99.508
SSAKE	116	82	14497	69.6971	6303	30.3029	176.7927	1	99.993
TRACESPipe	4	4	143550	99.7845	310	0.2155	35887.5000	665	99.454
TRACESPipeLite	6	4	102550	60.9466	65712	39.0534	779.6562	308	97.694
V-pipe	5	4	126469	78.8318	33960	21.1682	1356.4556	271	96.769
VirGenA	60	60	69319	99.3579	448	0.6421	875.3462	164	99.505
ViSpA	11	10	489793	96.7489	16459	3.2511	15837.8889	647	99.377

Table S113: Results obtained by dnadiff [6] when analysing the reconstruction of DS36. Only one execution of HVRS is represented.

Reconstruction tool	Total seqs	Aligned seqs	Aligned bases	Aligned bases (%)	Unaligned bases	Unaligned bases (%)	Average length	SNPs	Identity
coronaSPAdes	16	7	143569	68.3564	66461	31.6436	20509.8571	19	99.986
Haploflow	109	74	54771	66.3449	27784	33.6551	740.1486	21	99.886
IRMA	5	4	128093	88.5648	16539	11.4352	25618.6000	4	99.975
LAZYPIPE	17	8	143217	68.4165	66114	31.5835	17902.1250	8	99.953
metaSPAdes	17	8	143644	68.3677	66461	31.6323	17955.5000	16	99.986
metaviralSPAdes	–	–	–	–	–	–	–	–	–
PEHaplo	200	143	157637	68.6342	72040	31.3658	1096.0634	157	99.806
QuRe	2	1	70	59.8291	47	40.1709	70.0000	0	100.000
QVG	4	4	143860	100.0000	0	0.0000	35965.0000	3358	97.624
SPAdes	17	8	143665	68.3709	66461	31.6291	17958.1250	9	99.989
SSAKE	112	74	13967	67.5485	6710	32.4515	188.7432	0	100.000
TRACESPipe	4	4	143539	99.7706	330	0.2294	28707.8000	793	99.225
TRACESPipeLite	6	4	29276	17.3991	138986	82.6009	312.7368	63	98.598
V-pipe	5	4	125828	78.4322	34601	21.5678	1312.6809	424	95.936
VirGenA	89	89	62324	99.4939	317	0.5061	645.8211	135	99.656
ViSpA	15	13	429646	93.9939	27454	6.0061	19072.7500	632	98.667

Table S114: Results obtained by dnadiff [6] when analysing the reconstruction of DS37. Only one execution of HVRS is represented.

Reconstruction tool	Total seqs	Aligned seqs	Aligned bases	Aligned bases (%)	Unaligned bases	Unaligned bases (%)	Average length	SNPs	Identity
coronaSPAdes	24	16	143707	68.3746	66469	31.6254	8981.6875	12	99.990
Haploflow	98	66	52403	66.6205	26256	33.3795	796.9692	134	99.741
IRMA	5	4	128872	88.6291	16534	11.3709	14321.2222	13	99.905
LAZYPIPE	26	18	143054	68.3975	66097	31.6025	7947.4444	4	99.995
metaSPAdes	24	16	143742	68.3799	66469	31.6201	8983.8750	7	99.994
metaviralSPAdes	–	–	–	–	–	–	–	–	–
PEHaplo	202	137	158122	68.5469	72555	31.4531	1151.0224	109	99.925
QuRe	3	2	236	70.6587	98	29.3413	118.0000	0	100.000
QVG	4	4	143813	99.9673	47	0.0327	35953.2500	4998	96.505
SPAdes	24	16	143742	68.3799	66469	31.6201	8983.8750	9	99.991
SSAKE	113	78	14434	68.9995	6485	31.0005	185.0513	2	99.986
TRACESPipe	4	4	141961	98.6800	1899	1.3200	19776.8571	530	99.500
TRACESPipeLite	6	2	4163	2.4741	164099	97.5259	219.1053	1	99.976
V-pipe	5	4	125968	78.5195	34461	21.4805	1258.7938	689	95.647
VirGenA	81	81	62280	99.9904	6	0.0096	655.4105	61	99.705
ViSpA	41	40	2112414	99.2252	16495	0.7748	6898.8500	1051	97.848

Table S115: Results obtained by dnadiff [6] when analysing the reconstruction of DS38. Only one execution of HVRS is represented.

Reconstruction tool	Total seqs	Aligned seqs	Aligned bases	Aligned bases (%)	Unaligned bases	Unaligned bases (%)	Average length	SNPs	Identity
coronaSPAdes	19	16	143610	68.3428	66522	31.6572	8975.6250	13	99.989
Haploflow	104	73	60641	70.4121	25482	29.5879	830.6986	0	100.000
IRMA	5	4	128992	88.6336	16542	11.3664	9214.3571	8	99.852
LAZYPIPE	22	18	143056	68.2949	66412	31.7051	7947.5556	4	99.995
metaSPAdes	20	17	143706	68.3572	66522	31.6428	8453.2941	7	99.992
metaviralSPAdes	–	–	–	–	–	–	–	–	–
PEHaplo	192	134	157837	68.6567	72056	31.3433	1193.7727	4	99.615
QuRe	2	2	256	100.0000	0	0.0000	128.0000	0	100.000
QVG	4	4	143863	100.0000	0	0.0000	35965.7500	7540	94.730
SPAdes	18	15	143563	68.3357	66522	31.6643	9570.8667	5	99.993
SSAKE	112	79	14076	68.8077	6381	31.1923	178.1772	0	100.000
TRACESPipe	4	4	141458	98.3303	2402	1.6697	23576.3333	758	99.217
TRACESPipeLite	6	1	434	0.2579	167828	99.7421	217.0000	0	100.000
V-pipe	5	4	118868	74.0938	41561	25.9062	996.4262	78	94.147
VirGenA	79	78	52175	99.2788	379	0.7212	544.9688	77	99.786
ViSpA	88	87	3699971	99.4976	18682	0.5024	2102.8525	915	97.046

Table S116: Results obtained by dnadiff [6] when analysing the reconstruction of DS39. Only one execution of HVRS is represented.

Reconstruction tool	Total seqs	Aligned seqs	Aligned bases	Aligned bases (%)	Unaligned bases	Unaligned bases (%)	Average length	SNPs	Identity
coronaSPAdes	26	22	143612	68.4267	66265	31.5733	6527.8182	4	99.997
Haploflow	112	76	59297	68.1011	27775	31.8989	780.2237	0	100.000
IRMA	5	4	129301	88.6127	16616	11.3873	5622.6522	58	99.818
LAZYPIPE	27	23	142691	68.3697	66014	31.6303	6203.9565	3	99.998
metaSPAdes	26	22	143612	68.4267	66265	31.5733	6527.8182	2	99.998
metaviralSPAdes	–	–	–	–	–	–	–	–	–
PEHaplo	207	137	158836	68.0388	74613	31.9612	1177.6692	3	99.997
QuRe	3	3	504	100.0000	0	0.0000	168.0000	0	100.000
QVG	4	4	143816	99.9708	42	0.0292	35954.0000	11690	91.790
SPAdes	26	22	143612	68.4267	66265	31.5733	6527.8182	2	99.998
SSAKE	119	81	16045	69.1386	7162	30.8614	198.0864	0	100.000
TRACESPipe	4	4	141230	98.1718	2630	1.8282	12042.0833	345	98.700
TRACESPipeLite	5	0	0	0.0000	160429	100.0000	0.0000	0	0.000
V-pipe	5	4	103260	64.3649	57169	35.6351	603.4588	457	93.037
VirGenA	85	85	58507	99.3699	371	0.6301	637.5714	66	99.810
ViSpA	139	138	5096959	99.5460	23245	0.4540	687.4257	490	95.815

Table S117: Results obtained by dnadiff [6] when analysing the reconstruction of DS40. Only one execution of HVRS is represented.

Reconstruction tool	Total seqs	Aligned seqs	Aligned bases	Aligned bases (%)	Unaligned bases	Unaligned bases (%)	Average length	SNPs	Identity
coronaSPAdes	19	13	143535	68.3692	66406	31.6308	11041.1538	12	99.991
Haploflow	100	70	52778	69.8602	22770	30.1398	753.9714	0	100.000
IRMA	5	4	129116	88.4671	16832	11.5329	4310.2000	8	99.770
LAZYPIPE	19	13	142865	68.3522	66148	31.6478	10989.6154	4	99.996
metaSPAdes	19	13	143535	68.3692	66406	31.6308	11041.1538	7	99.993
metaviralSPAdes	–	–	–	–	–	–	–	–	–
PEHaplo	201	148	163127	68.5269	74921	31.4731	1080.3878	3	99.997
QuRe	1	1	164	100.0000	0	0.0000	164.0000	0	100.000
QVG	4	4	143849	99.9937	9	0.0063	35962.2500	15561	89.044
SPAdes	19	13	143535	68.3692	66406	31.6308	11041.1538	5	99.996
SSAKE	118	78	14256	65.7201	7436	34.2799	182.7692	0	100.000
TRACESPipe	4	4	136021	94.5510	7839	5.4490	7159.0000	1647	98.520
TRACESPipeLite	5	0	0	0.0000	160429	100.0000	0.0000	0	0.000
V-pipe	5	4	80756	50.3375	79673	49.6625	412.8918	212	91.872
VirGenA	71	71	66652	99.5534	299	0.4466	806.4268	142	99.743
ViSpA	115	114	3266451	98.9620	34263	1.0380	360.3264	228	95.858

Table S118: Results obtained by dnadiff [6] when analysing the reconstruction of DS41. Only one execution of HVRS is represented.

Reconstruction tool	Total seqs	Aligned seqs	Aligned bases	Aligned bases (%)	Unaligned bases	Unaligned bases (%)	Average length	SNPs	Identity
coronaSPAdes	9	7	135952	67.1487	66512	32.8513	22627.6667	1	99.900
Haploflow	53	37	136232	67.5871	65333	32.4129	3699.4167	13	99.976
IRMA	5	4	128291	88.5810	16538	11.4190	25658.2000	0	100.000
LAZYPIPE	7	5	135740	67.1239	66483	32.8761	17046.7500	0	99.971
metaSPAdes	9	7	135754	67.0499	66713	32.9501	15083.7778	0	99.952
metaviralSPAdes	–	–	–	–	–	–	–	–	–
PEHaplo	312	211	172528	68.2638	80209	31.7362	811.3268	0	100.000
QuRe	6	5	786	79.0744	208	20.9256	157.2000	0	100.000
QVG	–	–	–	–	–	–	–	–	–
SPAdes	8	6	136077	67.1687	66513	32.8313	22676.5000	0	99.917
SSAKE	375	254	83659	69.1791	37272	30.8209	328.5882	6	99.940
TRACESPipe	4	4	143743	99.9187	117	0.0813	35935.7500	0	100.000
TRACESPipeLite	6	5	144112	85.6474	24150	14.3526	35932.2500	1	99.991
V-pipe	5	4	143178	89.2470	17251	10.7530	28635.6000	0	99.837
VirGenA	–	–	–	–	–	–	–	–	–
ViSpA	5	4	143596	89.6808	16523	10.3192	35899.0000	0	100.000

Table S119: Results obtained by dnadiff [6] when analysing the reconstruction of DS42. Only one execution of HVRS is represented.

Reconstruction tool	Total seqs	Aligned seqs	Aligned bases	Aligned bases (%)	Unaligned bases	Unaligned bases (%)	Average length	SNPs	Identity
coronaSPAdes	13	11	141427	68.0166	66503	31.9834	12857.0000	7	99.995
Haploflow	57	40	141863	68.5948	64950	31.4052	3563.6410	142	99.844
IRMA	5	4	128272	88.5832	16532	11.4168	25654.4000	0	100.000
LAZYPIPE	10	8	143546	68.3552	66454	31.6448	27438.6000	291	99.786
metaSPAdes	7	5	142230	68.1071	66603	31.8929	28240.6000	84	99.944
metaviralSPAdes	–	–	–	–	–	–	–	–	–
PEHaplo	325	227	185352	70.4728	77660	29.5272	800.9231	108	99.888
QuRe	5	5	3332	99.9100	3	0.0900	666.4000	17	99.457
QVG	4	4	143860	100.0000	0	0.0000	35965.0000	551	99.537
SPAdes	7	5	143760	68.3715	66503	31.6285	28752.0000	2	99.999
SSAKE	341	230	73715	68.6519	33660	31.3481	323.0177	9	99.979
TRACESPipe	4	4	143774	99.9402	86	0.0598	28827.0000	438	99.663
TRACESPipeLite	6	4	143111	85.0525	25151	14.9475	17888.6250	479	99.338
V-pipe	5	4	142834	89.0325	17595	10.9675	25481.8333	362	99.398
VirGenA	46	46	56111	98.5527	824	1.4473	971.4386	155	99.655
ViSpA	5	4	143596	89.6920	16503	10.3080	35899.0000	444	99.676

Table S120: Results obtained by dnadiff [6] when analysing the reconstruction of DS43. Only one execution of HVRS is represented.

Reconstruction tool	Total seqs	Aligned seqs	Aligned bases	Aligned bases (%)	Unaligned bases	Unaligned bases (%)	Average length	SNPs	Identity
coronaSPAdes	6	4	143694	68.3505	66537	31.6495	35923.5000	2	99.991
Haploflow	44	30	141462	68.4009	65351	31.5991	4426.0312	616	99.537
IRMA	5	4	128264	88.5728	16548	11.4272	25654.4000	0	99.999
LAZYPIPE	8	6	143685	68.3765	66453	31.6235	20538.1429	6	99.981
metaSPAdes	6	4	143694	68.3505	66537	31.6495	35923.5000	2	99.998
metaviralSPAdes	–	–	–	–	–	–	–	–	–
PEHaplo	358	243	190191	69.6030	83060	30.3970	774.5275	145	99.839
QuRe	6	5	1596	91.4613	149	8.5387	319.2000	1	99.874
QVG	4	4	143858	99.9986	2	0.0014	35964.5000	812	99.432
SPAdes	6	4	143694	68.3505	66537	31.6495	35923.5000	0	100.000
SSAKE	374	244	77929	65.7773	40545	34.2227	321.4149	6	99.991
TRACESPipe	4	4	143811	99.9632	53	0.0368	35952.7500	741	99.481
TRACESPipeLite	6	4	114628	68.1239	53636	31.8761	1097.5192	359	97.533
V-pipe	5	4	143283	89.3124	17146	10.6876	35820.7500	693	99.348
VirGenA	68	68	53807	99.2255	420	0.7745	662.0506	93	99.788
ViSpA	5	4	143614	89.6870	16514	10.3130	35903.5000	801	99.410

Table S121: Results obtained by dnadiff [6] when analysing the reconstruction of DS44. Only one execution of HVRS is represented.

Reconstruction tool	Total seqs	Aligned seqs	Aligned bases	Aligned bases (%)	Unaligned bases	Unaligned bases (%)	Average length	SNPs	Identity
coronaSPAdes	6	4	143731	68.3779	66470	31.6221	35932.7500	0	100.000
Haploflow	61	42	141410	68.5505	64876	31.4495	3356.0476	69	99.933
IRMA	5	4	128494	88.6166	16506	11.3834	21416.0000	0	100.000
LAZYPIPE	6	4	143600	68.3731	66424	31.6269	35900.0000	0	99.983
metaSPAdes	6	4	143731	68.3779	66470	31.6221	35932.7500	0	100.000
metaviralSPAdes	–	–	–	–	–	–	–	–	–
PEHaplo	328	237	191181	71.0736	77809	28.9264	775.8610	44	99.949
QuRe	5	4	650	77.1971	192	22.8029	171.3333	0	99.806
QVG	4	4	143860	100.0000	0	0.0000	35965.0000	1293	99.056
SPAdes	6	4	143731	68.3779	66470	31.6221	35932.7500	0	100.000
SSAKE	348	236	76303	68.7198	34732	31.2802	323.3178	2	99.997
TRACESPipe	4	4	143771	99.9361	92	0.0639	35941.2500	893	99.294
TRACESPipeLite	6	4	37385	22.2183	130877	77.7817	330.1982	38	98.367
V-pipe	5	4	142897	89.0718	17532	10.9282	28579.4000	840	99.045
VirGenA	63	63	55574	99.0995	505	0.9005	742.2933	87	99.827
ViSpA	10	9	288411	94.5926	16487	5.4074	24618.3333	1069	99.064

Table S122: Results obtained by dnadiff [6] when analysing the reconstruction of DS45. Only one execution of HVRS is represented.

Reconstruction tool	Total seqs	Aligned seqs	Aligned bases	Aligned bases (%)	Unaligned bases	Unaligned bases (%)	Average length	SNPs	Identity
coronaSPAdes	6	4	143666	68.3668	66474	31.6332	35916.5000	1	99.991
Haploflow	45	35	140928	68.1052	65999	31.8948	4026.5143	0	100.000
IRMA	5	4	129184	88.6413	16554	11.3587	11744.2727	8	99.994
LAZYPIPE	6	4	143567	68.3581	66455	31.6419	35891.7500	0	100.000
metaSPAdes	6	4	143671	68.3669	66476	31.6331	35917.7500	1	99.991
metaviralSPAdes	–	–	–	–	–	–	–	–	–
PEHaplo	317	218	177593	69.5802	77642	30.4198	803.7321	39	99.935
QuRe	5	4	1545	91.7458	139	8.2542	386.2500	1	99.936
QVG	4	4	143812	99.9673	47	0.0327	35953.0000	1950	98.633
SPAdes	6	4	143671	68.3669	66476	31.6331	35917.7500	1	99.991
SSAKE	387	276	84168	69.6876	36611	30.3124	305.6667	2	99.994
TRACESPipe	4	4	143175	99.5328	672	0.4672	17896.8750	1516	98.594
TRACESPipeLite	6	3	6474	3.8476	161788	96.1524	239.7778	2	99.509
V-pipe	5	4	143231	89.2800	17198	10.7200	29026.6000	908	98.817
VirGenA	–	–	–	–	–	–	–	–	–
ViSpA	5	4	1485102	98.8976	16555	1.1024	12345.7500	1289	98.403

Table S123: Results obtained by dnadiff [6] when analysing the reconstruction of DS46. Only one execution of HVRS is represented.

Reconstruction tool	Total seqs	Aligned seqs	Aligned bases	Aligned bases (%)	Unaligned bases	Unaligned bases (%)	Average length	SNPs	Identity
coronaSPAdes	6	4	143695	68.3822	66440	31.6178	35923.7500	3	99.990
Haploflow	49	30	141422	68.4272	65253	31.5728	4714.0667	0	100.000
IRMA	5	4	129691	88.6564	16594	11.3436	9264.9286	3	99.989
LAZYPIPE	6	4	143602	68.3936	66362	31.6064	35900.5000	0	100.000
metaSPAdes	6	4	143695	68.3822	66440	31.6178	35923.7500	0	100.000
metaviralSPAdes	–	–	–	–	–	–	–	–	–
PEHaplo	299	201	167945	68.3837	77647	31.6163	836.8693	33	99.944
QuRe	8	7	1074	85.3058	185	14.6942	153.4286	0	100.000
QVG	4	4	143852	100.0000	0	0.0000	35963.0000	3754	97.368
SPAdes	6	4	143695	68.3822	66440	31.6178	35923.7500	0	100.000
SSAKE	377	260	83650	68.4013	38643	31.5987	322.9535	6	99.992
TRACESPipe	4	4	143660	99.8811	171	0.1189	35914.2500	239	99.809
TRACESPipeLite	6	1	227	0.1349	168035	99.8651	227.0000	0	100.000
V-pipe	5	4	140715	87.7117	19714	12.2883	7035.7500	1120	97.644
VirGenA	56	56	48588	99.4148	286	0.5852	675.3973	30	99.694
ViSpA	69	68	3250188	99.4894	16681	0.5106	3986.9714	1195	97.355

Table S124: Results obtained by dnadiff [6] when analysing the reconstruction of DS47. Only one execution of HVRS is represented.

Reconstruction tool	Total seqs	Aligned seqs	Aligned bases	Aligned bases (%)	Unaligned bases	Unaligned bases (%)	Average length	SNPs	Identity
coronaSPAdes	6	4	143784	68.3732	66509	31.6268	35946.0000	0	100.000
Haploflow	61	40	142664	68.5499	65453	31.4501	3566.6000	0	100.000
IRMA	5	4	129557	88.6618	16568	11.3382	7621.2941	13	99.988
LAZYPIPE	24	19	143592	68.3706	66428	31.6294	7557.4737	2	99.997
metaSPAdes	6	4	143784	68.3732	66509	31.6268	35946.0000	1	99.991
metaviralSPAdes	–	–	–	–	–	–	–	–	–
PEHaplo	310	207	170956	67.7628	81330	32.2372	824.4406	0	100.000
QuRe	4	4	1128	99.9114	1	0.0886	282.0000	4	99.648
QVG	4	4	143812	99.9708	42	0.0292	30786.6000	6950	94.724
SPAdes	6	4	143784	68.3732	66509	31.6268	35946.0000	1	99.991
SSAKE	357	236	78994	68.3830	36523	31.6170	335.6213	3	99.996
TRACESPipe	4	4	143701	100.0000	0	0.0000	35925.2500	3	99.991
TRACESPipeLite	5	0	0	0.0000	160429	100.0000	0.0000	0	0.000
V-pipe	5	4	131713	82.1005	28716	17.8995	1995.6515	1569	95.842
VirGenA	57	57	50620	98.4710	786	1.5290	708.3662	96	99.753
ViSpA	162	161	6551066	99.7176	18552	0.2824	1033.9009	1108	96.251

Table S125: Results obtained by dnadiff [6] when analysing the reconstruction of DS48. Only one execution of HVRS is represented.

Reconstruction tool	Total seqs	Aligned seqs	Aligned bases	Aligned bases (%)	Unaligned bases	Unaligned bases (%)	Average length	SNPs	Identity
coronaSPAdes	6	4	143723	68.3686	66495	31.6314	35930.7500	0	100.000
Haploflow	50	29	142509	68.4452	65700	31.5548	4914.1034	0	100.000
IRMA	5	4	130625	88.7302	16591	11.2698	5939.1818	55	99.958
LAZYPIPE	6	4	143565	68.3653	66432	31.6347	35891.2500	0	100.000
metaSPAdes	6	4	143723	68.3686	66495	31.6314	35930.7500	0	100.000
metaviralSPAdes	–	–	–	–	–	–	–	–	–
PEHaplo	305	207	166087	67.1878	81111	32.8122	806.0739	9	99.986
QuRe	7	6	2236	90.5630	233	9.4370	372.6667	7	99.639
QVG	4	4	143851	99.9937	9	0.0063	35962.7500	11569	91.836
SPAdes	6	4	143723	68.3686	66495	31.6314	35930.7500	0	100.000
SSAKE	360	249	78812	68.0047	37080	31.9953	318.0850	3	99.994
TRACESPipe	4	4	143770	99.9715	41	0.0285	35942.5000	52	99.851
TRACESPipeLite	5	0	0	0.0000	160429	100.0000	0.0000	0	0.000
V-pipe	5	4	114314	71.2552	46115	28.7448	905.5476	566	94.245
VirGenA	60	60	44245	98.9910	451	1.0090	638.4143	10	99.761
ViSpA	168	167	5712543	99.4543	31345	0.5457	500.4251	515	95.995

Table S126: Results obtained by dnadiff [6] when analysing the reconstruction of DS49. Only one execution of HVRS is represented.

Reconstruction tool	Total seqs	Aligned seqs	Aligned bases	Aligned bases (%)	Unaligned bases	Unaligned bases (%)	Average length	SNPs	Identity
coronaSPAdes	9	7	135981	67.1123	66636	32.8877	15117.0000	0	99.949
Haploflow	14	11	136947	67.3501	66389	32.6499	13556.2000	8	99.989
IRMA	5	4	128314	88.5767	16548	11.4233	25662.8000	0	100.000
LAZYPIPE	8	6	136021	67.1569	66521	32.8431	17016.6250	0	99.994
metaSPAdes	10	8	136068	67.1593	66537	32.8407	16994.7500	15	99.800
metaviralSPAdes	2	0	0	0.0000	66537	100.0000	0.0000	0	0.000
PEHaplo	215	140	210019	67.5185	101035	32.4815	1488.6016	0	99.999
QuRe	5	4	8273	85.4649	1407	14.5351	2068.2500	0	100.000
QVG	–	–	–	–	–	–	–	–	–
SPAdes	9	7	136187	67.1785	66537	32.8215	17030.7500	0	99.900
SSAKE	169	110	145530	67.1713	71125	32.8287	1871.2162	8	99.988
TRACESPipe	4	4	143829	99.9785	31	0.0215	35957.2500	0	100.000
TRACESPipeLite	6	5	144185	85.6908	24077	14.3092	35951.0000	0	100.000
V-pipe	5	4	143497	89.4458	16932	10.5542	35874.2500	0	100.000
VirGenA	61	61	52214	98.5226	783	1.4774	693.5205	8	99.925
ViSpA	5	4	143715	89.6795	16539	10.3205	35928.7500	0	100.000

Table S127: Results obtained by dnadiff [6] when analysing the reconstruction of DS50. Only one execution of HVRS is represented.

Reconstruction tool	Total seqs	Aligned seqs	Aligned bases	Aligned bases (%)	Unaligned bases	Unaligned bases (%)	Average length	SNPs	Identity
coronaSPAdes	12	10	141394	67.9968	66548	32.0032	14139.4000	6	99.996
Haploflow	11	9	143215	68.3111	66436	31.6889	14321.6000	315	99.704
IRMA	5	4	128351	88.5723	16560	11.4277	25670.4000	0	100.000
LAZYPIPE	8	6	143661	68.3540	66511	31.6460	19257.1250	1	99.963
metaSPAdes	6	4	143796	68.3623	66548	31.6377	35949.0000	78	99.947
metaviralSPAdes	3	1	125025	65.2623	66548	34.7377	125025.0000	0	100.000
PEHaplo	243	176	249458	72.3396	95385	27.6604	1397.3841	114	99.917
QuRe	4	3	4905	84.0617	930	15.9383	1635.0000	4	99.918
QVG	4	4	143860	100.0000	0	0.0000	35965.0000	498	99.577
SPAdes	6	4	143810	68.3644	66548	31.6356	35952.5000	1	99.999
SSAKE	164	116	151144	68.2720	70241	31.7280	1594.0769	104	99.924
TRACESPipe	4	4	143833	99.9805	28	0.0195	35958.2500	404	99.719
TRACESPipeLite	6	4	143280	85.1534	24981	14.8466	20468.5714	475	99.527
V-pipe	5	4	143544	89.4751	16885	10.5249	35886.0000	476	99.664
VirGenA	37	37	46433	98.9958	471	1.0042	842.7500	96	99.523
ViSpA	5	4	143725	89.6824	16535	10.3176	35931.2500	551	99.569

Table S128: Results obtained by dnadiff [6] when analysing the reconstruction of DS51. Only one execution of HVRS is represented.

Reconstruction tool	Total seqs	Aligned seqs	Aligned bases	Aligned bases (%)	Unaligned bases	Unaligned bases (%)	Average length	SNPs	Identity
coronaSPAdes	6	4	143813	68.3655	66546	31.6345	35953.2500	2	99.999
Haploflow	11	9	149790	69.2799	66420	30.7201	20506.2857	245	99.792
IRMA	5	4	128364	88.5690	16567	11.4310	25673.0000	0	100.000
LAZYPIPE	8	6	144054	68.4053	66535	31.5947	18936.7500	1	99.478
metaSPAdes	6	4	143813	68.3655	66546	31.6345	35953.2500	1	99.999
metaviralSPAdes	3	1	125029	65.2637	66546	34.7363	125029.0000	0	100.000
PEHaplo	242	159	223138	68.7082	101624	31.2918	1384.3066	0	99.944
QuRe	5	4	6906	77.9458	1954	22.0542	1561.6000	3	98.835
QVG	4	4	143858	99.9986	2	0.0014	35964.5000	741	99.488
SPAdes	6	4	143813	68.3655	66546	31.6345	35953.2500	1	99.999
SSAKE	172	108	151571	67.7982	71991	32.2018	1791.6707	1	99.998
TRACESPipe	4	4	143805	99.9604	57	0.0396	35951.2500	609	99.579
TRACESPipeLite	6	4	110125	70.4750	46136	29.5250	1280.5233	315	97.753
V-pipe	5	4	143518	89.4589	16911	10.5411	35879.5000	590	99.591
VirGenA	47	47	48429	99.4394	273	0.5606	792.5410	62	99.659
ViSpA	5	4	143735	89.6752	16549	10.3248	35933.7500	638	99.522

Table S129: Results obtained by dnadiff [6] when analysing the reconstruction of DS52. Only one execution of HVRS is represented.

Reconstruction tool	Total seqs	Aligned seqs	Aligned bases	Aligned bases (%)	Unaligned bases	Unaligned bases (%)	Average length	SNPs	Identity
coronaSPAdes	6	4	143790	68.3591	66555	31.6409	35947.5000	0	100.000
Haploflow	9	7	143379	68.3180	66491	31.6820	20162.7143	499	99.371
IRMA	5	4	128391	88.5724	16565	11.4276	25678.2000	0	100.000
LAZYPIPE	6	4	143689	68.3478	66543	31.6522	28766.4000	0	99.985
metaSPAdes	6	4	143791	68.3593	66555	31.6407	35947.7500	0	100.000
metaviralSPAdes	3	1	125021	65.2592	66555	34.7408	125021.0000	0	100.000
PEHaplo	217	155	211608	70.0320	90551	29.9680	1426.8676	18	99.983
QuRe	5	4	6961	81.6444	1565	18.3556	1740.2500	4	99.942
QVG	4	4	143860	100.0000	0	0.0000	35965.0000	1058	99.221
SPAdes	6	4	143791	68.3593	66555	31.6407	35947.7500	0	100.000
SSAKE	147	101	150862	68.2769	70094	31.7231	1793.2073	0	99.999
TRACESPipe	4	4	143804	99.9611	56	0.0389	35951.0000	6	99.986
TRACESPipeLite	6	4	43893	26.0859	124370	73.9141	359.3025	103	98.240
V-pipe	5	4	143508	89.4527	16921	10.5473	30718.4000	556	99.201
VirGenA	46	46	42874	99.5704	185	0.4296	661.7833	55	99.801
ViSpA	11	10	475775	96.6360	16562	3.3640	46120.6667	1024	99.061

Table S130: Results obtained by dnadiff [6] when analysing the reconstruction of DS53. Only one execution of HVRS is represented.

Reconstruction tool	Total seqs	Aligned seqs	Aligned bases	Aligned bases (%)	Unaligned bases	Unaligned bases (%)	Average length	SNPs	Identity
coronaSPAdes	6	4	143766	68.3571	66550	31.6429	35941.5000	1	99.999
Haploflow	8	6	144613	68.5253	66423	31.4747	24101.1667	0	99.862
IRMA	5	4	129518	88.6606	16565	11.3394	9974.5385	7	99.976
LAZYPIPE	6	4	143712	68.3575	66524	31.6425	35928.0000	1	99.999
metaSPAdes	6	4	143767	68.3573	66550	31.6427	35941.7500	1	99.999
metaviralSPAdes	3	1	125016	65.2600	66550	34.7400	125016.0000	0	100.000
PEHaplo	230	155	215544	68.8808	97379	31.1192	1403.2101	148	99.874
QuRe	4	3	1652	53.3247	1446	46.6753	550.6667	2	99.878
QVG	4	4	143813	99.9673	47	0.0327	35953.2500	1495	98.959
SPAdes	6	4	143767	68.3573	66550	31.6427	35941.7500	1	99.999
SSAKE	179	112	151367	67.8481	71730	32.1519	1724.0588	0	99.997
TRACESPipe	4	4	143806	99.9861	20	0.0139	35951.5000	1	99.999
TRACESPipeLite	6	3	8049	4.7836	160213	95.2164	236.5758	0	99.658
V-pipe	5	4	143508	89.4527	16921	10.5473	35875.2500	1157	99.096
VirGenA	–	–	–	–	–	–	–	–	–
ViSpA	5	4	1054129	98.4540	16553	1.5460	19976.6667	1293	98.564

Table S131: Results obtained by dnadiff [6] when analysing the reconstruction of DS54. Only one execution of HVRS is represented.

Reconstruction tool	Total seqs	Aligned seqs	Aligned bases	Aligned bases (%)	Unaligned bases	Unaligned bases (%)	Average length	SNPs	Identity
coronaSPAdes	6	4	143803	68.3617	66553	31.6383	35950.7500	0	100.000
Haploflow	7	5	143470	68.3773	66351	31.6227	28694.0000	0	100.000
IRMA	5	4	129886	88.6903	16563	11.3097	8660.5333	6	99.993
LAZYPIPE	6	4	143771	68.3667	66523	31.6333	35942.7500	0	100.000
metaSPAdes	6	4	143807	68.3623	66553	31.6377	35951.7500	0	100.000
metaviralSPAdes	3	1	125037	65.2628	66553	34.7372	125037.0000	0	100.000
PEHaplo	236	174	227715	71.7143	89816	28.2857	1330.0724	14	99.990
QuRe	6	5	14299	90.0838	1574	9.9162	2850.4000	9	96.671
QVG	4	4	143862	100.0000	0	0.0000	35965.5000	2719	98.103
SPAdes	6	4	143807	68.3623	66553	31.6377	35951.7500	0	100.000
SSAKE	171	113	151255	68.0070	71156	31.9930	1585.4516	0	99.998
TRACESPipe	4	4	143818	99.9819	26	0.0181	35954.5000	0	100.000
TRACESPipeLite	6	2	746	0.4434	167516	99.5566	248.6667	0	100.000
V-pipe	5	4	142742	88.9752	17687	11.0248	15859.4444	1248	98.469
VirGenA	47	47	40467	99.8372	66	0.1628	638.5938	106	99.366
ViSpA	57	56	2591210	99.3631	16610	0.6369	4487.0000	723	98.415

Table S132: Results obtained by dnadiff [6] when analysing the reconstruction of DS55. Only one execution of HVRS is represented.

Reconstruction tool	Total seqs	Aligned seqs	Aligned bases	Aligned bases (%)	Unaligned bases	Unaligned bases (%)	Average length	SNPs	Identity
coronaSPAdes	6	4	143804	68.3593	66561	31.6407	35951.0000	1	99.991
Haploflow	6	4	143453	68.3487	66431	31.6513	35863.2500	0	100.000
IRMA	5	4	129570	88.6658	16563	11.3342	8638.4667	32	99.973
LAZYPIPE	6	4	143728	68.3592	66526	31.6408	35932.0000	0	100.000
metaSPAdes	6	4	143804	68.3593	66561	31.6407	35951.0000	0	100.000
metaviralSPAdes	3	1	125038	65.2603	66561	34.7397	125038.0000	0	100.000
PEHaplo	231	155	211923	68.1081	99234	31.8919	1394.5214	1	99.999
QuRe	5	4	3165	76.8204	955	23.1796	791.2500	7	99.780
QVG	4	4	143818	99.9708	42	0.0292	30787.8000	5078	96.251
SPAdes	6	4	143804	68.3593	66561	31.6407	35951.0000	0	100.000
SSAKE	169	111	151654	68.4257	69979	31.5743	1768.2410	1	99.998
TRACESPipe	4	4	143780	100.0000	0	0.0000	35945.0000	1	99.999
TRACESPipeLite	5	0	0	0.0000	160429	100.0000	0.0000	0	0.000
V-pipe	5	4	137879	85.9439	22550	14.0561	4055.2647	1529	97.021
VirGenA	50	49	47872	99.6337	176	0.3663	749.9844	47	99.869
ViSpA	147	146	6162466	99.7015	18451	0.2985	1315.8614	886	96.130

Table S133: Results obtained by dnadiff [6] when analysing the reconstruction of DS56. Only one execution of HVRS is represented.

Reconstruction tool	Total seqs	Aligned seqs	Aligned bases	Aligned bases (%)	Unaligned bases	Unaligned bases (%)	Average length	SNPs	Identity
coronaSPAdes	6	4	143807	68.3666	66540	31.6334	35951.7500	0	100.000
Haploflow	6	4	143521	68.3958	66318	31.6042	35880.2500	0	100.000
IRMA	5	4	131193	88.6997	16714	11.3003	8200.5625	111	99.845
LAZYPIPE	6	4	143754	68.3631	66526	31.6369	35938.5000	0	100.000
metaSPAdes	6	4	143807	68.3666	66540	31.6334	35951.7500	0	100.000
metaviralSPAdes	3	1	125013	65.2629	66540	34.7371	125013.0000	0	100.000
PEHaplo	232	160	214186	67.0408	105300	32.9592	1313.4211	0	99.982
QuRe	4	3	4482	86.2255	716	13.7745	1494.0000	13	99.710
QVG	4	4	143855	99.9937	9	0.0063	35963.7500	9454	93.317
SPAdes	6	4	143807	68.3666	66540	31.6334	35951.7500	0	100.000
SSAKE	189	130	152803	68.5086	70239	31.4914	1499.6429	0	99.999
TRACESPipe	4	4	143754	100.0000	0	0.0000	35938.5000	0	100.000
TRACESPipeLite	5	0	0	0.0000	160429	100.0000	0.0000	0	0.000
V-pipe	5	4	126236	78.6865	34193	21.3135	1491.8675	1088	94.938
VirGenA	46	46	47210	98.8050	571	1.1950	728.9692	41	99.859
ViSpA	200	199	6867933	99.7126	19795	0.2874	597.7722	670	96.098

Table S134: Results obtained by dnadiff [6] when analysing the reconstruction of DS57. Only one execution of HVRS is represented.

Reconstruction tool	Total seqs	Aligned seqs	Aligned bases	Aligned bases (%)	Unaligned bases	Unaligned bases (%)	Average length	SNPs	Identity
coronaSPAdes	8	7	137147	73.2721	50028	26.7279	22823.8333	67	99.901
Haploflow	50	35	146800	74.7827	49502	25.2173	4281.9688	97	99.926
IRMA	4	4	128277	100.0000	0	0.0000	25655.4000	0	100.000
LAZYPIPE	8	7	136744	73.2799	49861	26.7201	14206.8000	16	99.874
metaSPAdes	11	10	136810	73.2239	50028	26.7761	11392.5000	54	99.867
metaviralSPAdes	–	–	–	–	–	–	–	–	–
PEHaplo	291	220	176357	74.9817	58843	25.0183	783.8852	56	99.940
QuRe	7	7	4065	99.9754	1	0.0246	459.6000	9	99.609
QVG	4	4	143858	99.9986	2	0.0014	35964.5000	160	99.893
SPAdes	22	21	145317	74.3895	50029	25.6105	6667.7143	38	99.928
SSAKE	371	269	82929	72.1969	31936	27.8031	308.9476	10	99.983
TRACESPipe	4	4	143717	99.8999	144	0.1001	35929.2500	131	99.910
TRACESPipeLite	5	5	143976	94.9128	7717	5.0872	35945.0000	178	99.876
V-pipe	5	4	143243	89.2875	17186	10.7125	28648.6000	141	99.684
VirGenA	72	72	69940	99.3748	440	0.6252	802.0714	32	99.901
ViSpA	5	4	143673	100.0000	0	0.0000	35918.2500	168	99.883

Table S135: Results obtained by dnadiff [6] when analysing the reconstruction of DS58. Only one execution of HVRS is represented.

Reconstruction tool	Total seqs	Aligned seqs	Aligned bases	Aligned bases (%)	Unaligned bases	Unaligned bases (%)	Average length	SNPs	Identity
coronaSPAdes	10	9	137255	89.1741	16663	10.8259	15237.3333	62	99.908
Haploflow	40	37	144560	89.9928	16075	10.0072	3653.6486	94	99.914
IRMA	5	4	128346	88.5695	16564	11.4305	25669.2000	0	100.000
LAZYPIPE	7	6	136300	89.1911	16518	10.8089	19452.5714	72	99.943
metaSPAdes	11	10	136859	89.1450	16665	10.8550	9971.2143	33	99.840
metaviralSPAdes	–	–	–	–	–	–	–	–	–
PEHaplo	224	197	167559	89.6133	19421	10.3867	835.2775	58	99.958
QuRe	7	5	1620	90.3010	174	9.6990	307.7500	3	99.755
QVG	4	4	143858	99.9986	2	0.0014	35964.5000	168	99.881
SPAdes	22	21	145142	89.7551	16567	10.2449	6670.3810	50	99.896
SSAKE	296	256	77442	86.9012	11673	13.0988	301.9881	16	99.970
TRACESPipe	4	4	143776	99.9416	84	0.0584	35944.0000	174	99.877
TRACESPipeLite	6	4	143790	85.4560	24472	14.5440	35947.5000	139	99.903
V-pipe	5	4	143297	89.3211	17132	10.6789	35824.2500	148	99.740
VirGenA	70	70	105575	99.9650	37	0.0350	1389.0400	105	99.876
ViSpA	5	4	143614	89.6842	16519	10.3158	35903.5000	188	99.867

Table S136: Results obtained by dnadiff [6] when analysing the reconstruction of DS59. Only one execution of HVRS is represented.

Reconstruction tool	Total seqs	Aligned seqs	Aligned bases	Aligned bases (%)	Unaligned bases	Unaligned bases (%)	Average length	SNPs	Identity
coronaSPAdes	12	10	141384	68.0005	66532	31.9995	14138.4000	46	99.967
Haploflow	16	13	145293	68.6316	66407	31.3684	11753.8333	457	99.660
IRMA	5	4	128326	88.5777	16548	11.4223	25665.2000	1	99.999
LAZYPIPE	8	6	143705	68.3691	66485	31.6309	19259.2500	1	99.963
metaSPAdes	7	5	143550	68.3305	66532	31.6695	35805.5000	76	99.946
metaviralSPAdes	–	–	–	–	–	–	–	–	–
PEHaplo	301	215	227014	72.2284	87286	27.7716	1012.9946	132	99.886
QuRe	4	3	4244	95.1143	218	4.8857	1414.6667	6	99.860
QVG	4	4	143860	100.0000	0	0.0000	35965.0000	503	99.573
SPAdes	6	4	143800	68.3681	66532	31.6319	35950.0000	2	99.998
SSAKE	408	275	150044	68.2881	69678	31.7119	558.7424	0	99.995
TRACESPipe	4	4	143806	99.9632	53	0.0368	35951.5000	498	99.653
TRACESPipeLite	6	4	143272	85.1482	24990	14.8518	20467.4286	452	99.519
V-pipe	5	4	143417	89.3959	17012	10.6041	35854.2500	454	99.683
VirGenA	53	53	46333	99.7589	112	0.2411	722.7500	96	99.713
ViSpA	5	4	143668	89.6882	16518	10.3118	35917.0000	610	99.564

Table S137: Results obtained by dnadiff [6] when analysing the reconstruction of DS60. Only one execution of HVRS is represented.

Reconstruction tool	Total seqs	Aligned seqs	Aligned bases	Aligned bases (%)	Unaligned bases	Unaligned bases (%)	Average length	SNPs	Identity
coronaSPAdes	6	4	143807	68.3601	66560	31.6399	35951.7500	0	100.000
Haploflow	8	6	145254	68.6067	66466	31.3933	20520.2857	206	99.844
IRMA	5	4	128358	88.5698	16565	11.4302	25672.4000	0	100.000
LAZYPIPE	7	5	143750	68.3590	66537	31.6410	20574.4286	0	99.976
metaSPAdes	6	4	143807	68.3601	66560	31.6399	35951.7500	0	100.000
metaviralSPAdes	3	1	125037	65.2604	66560	34.7396	125037.0000	0	100.000
PEHaplo	218	150	238466	71.3406	95798	28.6594	1562.7647	146	99.894
QuRe	4	4	4362	100.0000	0	0.0000	1090.5000	5	99.888
QVG	4	4	143858	99.9986	2	0.0014	35964.5000	736	99.488
SPAdes	6	4	143807	68.3601	66560	31.6399	35951.7500	0	100.000
SSAKE	171	116	152862	68.4041	70607	31.5959	1784.5000	0	99.999
TRACESPipe	4	4	143816	99.9646	51	0.0354	35954.0000	689	99.510
TRACESPipeLite	6	4	121031	71.9297	47232	28.0703	1384.5955	52	97.436
V-pipe	5	4	143557	89.4832	16872	10.5168	35889.2500	586	99.590
VirGenA	–	–	–	–	–	–	–	–	–
ViSpA	5	4	143799	89.6726	16561	10.3274	35949.7500	784	99.413

Table S138: Results obtained by dnadiff [6] when analysing the reconstruction of DS61. Only one execution of HVRS is represented.

Reconstruction tool	Total seqs	Aligned seqs	Aligned bases	Aligned bases (%)	Unaligned bases	Unaligned bases (%)	Average length	SNPs	Identity
coronaSPAdes	15	13	136145	89.1170	16626	10.8830	9732.0000	236	99.739
Haploflow	–	–	–	–	–	–	–	–	–
IRMA	20	15	172489	23.3110	567458	76.6890	22187.8333	1	99.997
LAZYPIPE	176	160	93649	90.0740	10320	9.9260	585.3062	509	99.373
metaSPAdes	63	58	137397	89.3238	16422	10.6762	3086.0227	264	99.775
metaviralSPAdes	–	–	–	–	–	–	–	–	–
PEHaplo	201	177	105160	89.0040	12992	10.9960	578.5402	63	99.924
QuRe	6	3	368	63.5579	211	36.4421	122.6667	0	100.000
QVG	4	4	143858	99.9986	2	0.0014	35964.5000	1201	99.166
SPAdes	18	17	136418	89.1627	16581	10.8373	9078.1333	127	99.849
SSAKE	–	–	–	–	–	–	–	–	–
TRACESPipe	4	4	143838	99.9840	23	0.0160	35959.5000	136	99.902
TRACESPipeLite	5	1	1605	0.9996	158955	99.0004	224.4286	12	98.155
V-pipe	5	0	0	0.0000	160429	100.0000	0.0000	0	0.000
VirGenA	–	–	–	–	–	–	–	–	–
ViSpA	5	4	415335	96.1682	16549	3.8318	22691.0000	71	99.561

Table S139: Results obtained by dnadiff [6] when analysing the reconstruction of DS62. Only one execution of HVRS is represented.

Reconstruction tool	Total seqs	Aligned seqs	Aligned bases	Aligned bases (%)	Unaligned bases	Unaligned bases (%)	Average length	SNPs	Identity
coronaSPAdes	11	10	138811	89.3095	16616	10.6905	15284.5556	35	99.969
Haploflow	83	77	123549	89.4803	14525	10.5197	1586.3333	64	99.901
IRMA	20	15	172537	23.3160	567456	76.6840	20139.0000	4	99.225
LAZYPIPE	8	7	138145	89.3235	16512	10.6765	17207.1250	49	99.911
metaSPAdes	13	12	136983	89.1254	16714	10.8746	11397.7500	49	99.856
metaviralSPAdes	3	2	117242	87.6517	16517	12.3483	58621.0000	56	99.953
PEHaplo	171	151	181218	89.3915	21506	10.6085	1232.6176	39	99.958
QuRe	6	4	1039	86.2957	165	13.7043	259.7500	0	100.000
QVG	4	4	143858	99.9986	2	0.0014	35964.5000	151	99.891
SPAdes	14	13	142701	89.6267	16516	10.3733	11792.6667	31	99.981
SSAKE	104	91	18016	86.8743	2722	13.1257	197.9780	1	99.994
TRACESPipe	4	4	143775	99.9409	85	0.0591	35943.7500	116	99.922
TRACESPipeLite	6	4	143749	85.4316	24513	14.5684	35937.2500	135	99.904
V-pipe	5	4	142704	88.9515	17725	11.0485	35676.0000	132	99.715
VirGenA	28	28	99108	99.6812	317	0.3188	2921.3235	204	99.778
ViSpA	5	4	143636	89.6907	16510	10.3093	35909.0000	256	99.815

Table S140: Results obtained by dnadiff [6] when analysing the reconstruction of DS63. Only one execution of HVRS is represented.

Reconstruction tool	Total seqs	Aligned seqs	Aligned bases	Aligned bases (%)	Unaligned bases	Unaligned bases (%)	Average length	SNPs	Identity
coronaSPAdes	17	15	291456	81.3818	66678	18.6182	22351.3846	126	99.957
Haploflow	89	71	307366	82.3543	65858	17.6457	4463.4030	217	99.888
IRMA	6	5	282079	94.4722	16505	5.5278	35306.0000	96	99.944
LAZYPIPE	14	12	290101	81.3604	66462	18.6396	20026.4667	152	99.854
metaSPAdes	29	27	291139	81.2976	66976	18.7024	12105.9167	113	99.868
metaviralSPAdes	–	–	–	–	–	–	–	–	–
PEHaplo	535	433	399761	82.1340	86957	17.8660	928.0124	242	99.895
QuRe	14	11	3434	88.5965	442	11.4035	238.2222	7	99.446
QVG	5	5	305972	99.9993	2	0.0007	61194.4000	341	99.881
SPAdes	53	51	303689	81.9972	66676	18.0028	6193.8542	54	99.939
SSAKE	417	346	111014	82.5727	23430	17.4273	322.5982	18	99.980
TRACESPipe	7	7	608310	97.3604	16492	2.6396	61077.2000	336	99.890
TRACESPipeLite	7	6	306116	92.6569	24260	7.3431	61183.8000	303	99.893
V-pipe	6	5	305271	94.6451	17272	5.3549	61050.2000	321	99.756
VirGenA	83	82	78143	98.7739	970	1.2261	703.3364	56	99.861
ViSpA	12	11	731237	97.7943	16493	2.2057	49566.8333	275	99.871

Table S141: Results obtained by dnadiff [6] when analysing the reconstruction of DS64. Only one execution of HVRS is represented.

Reconstruction tool	Total seqs	Aligned seqs	Aligned bases	Aligned bases (%)	Unaligned bases	Unaligned bases (%)	Average length	SNPs	Identity
coronaSPAdes	22	20	285059	81.0411	66687	18.9589	15512.4211	81	99.821
Haploflow	89	72	302847	82.1062	66001	17.8938	3948.0694	203	99.897
IRMA	6	5	281319	94.4423	16555	5.5577	11491.0800	44	99.726
LAZYPIPE	32	30	288596	81.2990	66385	18.7010	9284.5161	131	99.929
metaSPAdes	17	15	283200	80.8155	67228	19.1845	11828.8750	92	99.893
metaviralSPAdes	1	1	5358	100.0000	0	0.0000	5358.0000	2	99.960
PEHaplo	524	407	383606	81.2220	88687	18.7780	945.7447	142	99.952
QuRe	11	10	2906	93.5608	200	6.4392	284.0000	10	99.559
QVG	5	5	316189	100.0000	0	0.0000	54330.5000	432	99.857
SPAdes	62	60	298126	81.7648	66488	18.2352	5423.3148	48	99.846
SSAKE	376	313	108791	85.6096	18287	14.3904	351.1732	28	99.971
TRACESPipe	6	6	321012	99.9648	113	0.0352	52947.0000	398	99.866
TRACESPipeLite	7	6	316226	92.8463	24365	7.1537	52938.1667	395	99.867
V-pipe	6	5	315365	94.7731	17393	5.2269	52560.8333	434	99.686
VirGenA	73	73	81237	98.5611	1186	1.4389	709.0783	61	99.875
ViSpA	13	12	835387	98.0614	16515	1.9386	29216.8000	235	99.894

Table S142: Results obtained by dnadiff [6] when analysing the reconstruction of DS65. Only one execution of HVRS is represented.

Reconstruction tool	Total seqs	Aligned seqs	Aligned bases	Aligned bases (%)	Unaligned bases	Unaligned bases (%)	Average length	SNPs	Identity
coronaSPAdes	16	14	369164	84.7149	66608	15.2851	33496.1818	61	99.963
Haploflow	113	96	382763	85.2680	66131	14.7320	3963.2553	63	99.958
IRMA	6	5	356682	95.5683	16540	4.4317	50954.7143	0	100.000
LAZYPIPE	10	8	368598	84.7303	66427	15.2697	33603.4545	70	99.968
metaSPAdes	15	13	368348	84.6861	66609	15.3139	21879.9412	40	99.919
metaviralSPAdes	–	–	–	–	–	–	–	–	–
PEHaplo	592	510	502042	85.3827	85948	14.6173	997.5705	86	99.974
QuRe	11	7	1333	75.9544	422	24.0456	196.3333	2	99.746
QVG	5	5	379187	99.9995	2	0.0005	75837.4000	224	99.941
SPAdes	34	32	378544	85.0552	66513	14.9448	12046.7097	33	99.970
SSAKE	417	349	117901	84.2156	22098	15.7844	339.9246	12	99.990
TRACESPipe	5	5	379100	99.9765	89	0.0235	75820.0000	196	99.951
TRACESPipeLite	7	6	379329	93.9887	24261	6.0113	75824.2000	190	99.951
V-pipe	6	5	378196	95.5624	17562	4.4376	54028.0000	195	99.805
VirGenA	6	6	5407	93.2724	390	6.7276	262.0000	2	99.964
ViSpA	6	5	378485	95.8183	16518	4.1817	75697.0000	232	99.940

Table S143: Results obtained by dnadiff [6] when analysing the reconstruction of DS66. Only one execution of HVRS is represented.

Reconstruction tool	Total seqs	Aligned seqs	Aligned bases	Aligned bases (%)	Unaligned bases	Unaligned bases (%)	Average length	SNPs	Identity
coronaSPAdes	22	15	133751	67.1596	65403	32.8404	8715.5333	74	99.529
Haploflow	29	19	38642	73.6768	13806	26.3232	1752.6842	12	99.958
IRMA	20	15	173187	23.4435	565554	76.5565	11664.8333	114	99.004
LAZYPIPE	68	48	131975	67.2398	64300	32.7602	2641.7400	83	99.881
metaSPAdes	–	–	–	–	–	–	–	–	–
metaviralSPAdes	–	–	–	–	–	–	–	–	–
PEHaplo	118	81	141330	66.9776	69681	33.0224	1720.4304	96	99.898
QuRe	–	–	–	–	–	–	–	–	–
QVG	5	4	143860	89.6721	16569	10.3279	35965.0000	26	99.970
SPAdes	–	–	–	–	–	–	–	–	–
SSAKE	109	73	23883	71.9259	9322	28.0741	328.9861	5	99.979
TRACESPipe	4	4	143860	100.0000	0	0.0000	35965.0000	0	100.000
TRACESPipeLite	4	1	282	0.1825	154210	99.8175	282.0000	1	99.650
V-pipe	5	4	88634	55.2481	71795	44.7519	457.2842	47	93.199
VirGenA	99	99	51644	98.9956	524	1.0044	499.7864	27	99.879
ViSpA	41	37	2009655	98.6901	26674	1.3099	6337.9524	76	97.490

Table S144: Results obtained by dnadiff [6] when analysing the reconstruction of DS67. Only one execution of HVRS is represented.

Reconstruction tool	Total seqs	Aligned seqs	Aligned bases	Aligned bases (%)	Unaligned bases	Unaligned bases (%)	Average length	SNPs	Identity
coronaSPAdes	44	31	133140	66.9964	65587	33.0036	3915.9412	534	99.230
Haploflow	–	–	–	–	–	–	–	–	–
IRMA	20	15	173187	23.4435	565554	76.5565	11664.8333	114	99.004
LAZYPIPE	85	65	132741	67.4141	64163	32.5859	2181.8500	325	99.717
metaSPAdes	53	44	133654	67.1348	65429	32.8652	2960.3556	396	99.257
metaviralSPAdes	–	–	–	–	–	–	–	–	–
PEHaplo	164	118	131837	66.7529	65663	33.2471	1122.2174	174	99.810
QuRe	–	–	–	–	–	–	–	–	–
QVG	–	–	–	–	–	–	–	–	–
SPAdes	37	30	131602	66.9754	64891	33.0246	3872.8824	372	99.231
SSAKE	20	18	4078	89.3319	487	10.6681	226.5556	9	99.779
TRACESPipe	4	4	143281	99.5961	581	0.4039	28656.2000	374	99.443
TRACESPipeLite	5	1	245	0.1535	159397	99.8465	199.0000	1	82.950
V-pipe	5	0	0	0.0000	160429	100.0000	0.0000	0	0.000
VirGenA	87	87	50232	99.3749	316	0.6251	501.6735	167	99.547
ViSpA	41	37	2009531	98.6877	26721	1.3123	6865.0000	176	97.402

Table S145: Results obtained by dnadiff [6] when analysing the reconstruction of DS68. Only one execution of HVRS is represented.

Reconstruction tool	Total seqs	Aligned seqs	Aligned bases	Aligned bases (%)	Unaligned bases	Unaligned bases (%)	Average length	SNPs	Identity
coronaSPAdes	122	89	133242	67.0683	65424	32.9317	1506.9659	1849	98.380
Haploflow	–	–	–	–	–	–	–	–	–
IRMA	20	15	173187	23.4435	565554	76.5565	11664.8333	114	99.004
LAZYPIPE	214	149	148946	67.6757	71142	32.3243	1124.8934	1266	98.683
metaSPAdes	348	219	102673	65.4172	54278	34.5828	472.6587	1236	97.389
metaviralSPAdes	–	–	–	–	–	–	–	–	–
PEHaplo	204	137	125825	64.2705	69949	35.7295	915.0692	305	99.634
QuRe	–	–	–	–	–	–	–	–	–
QVG	–	–	–	–	–	–	–	–	–
SPAdes	238	151	93509	67.1741	45695	32.8259	619.0199	1005	98.627
SSAKE	–	–	–	–	–	–	–	–	–
TRACESPipe	4	4	143281	99.5975	579	0.4025	28656.2000	853	99.110
TRACESPipeLite	2	0	0	0.0000	130636	100.0000	0.0000	0	0.000
V-pipe	5	0	0	0.0000	160429	100.0000	0.0000	0	0.000
VirGenA	99	98	50627	99.3329	340	0.6671	476.6250	547	98.758
ViSpA	41	37	2009507	98.6865	26746	1.3135	6885.6842	429	97.245

Table S146: Results obtained by dnadiff [6] when analysing the reconstruction of DS69. Only one execution of HVRS is represented.

Reconstruction tool	Total seqs	Aligned seqs	Aligned bases	Aligned bases (%)	Unaligned bases	Unaligned bases (%)	Average length	SNPs	Identity
coronaSPAdes	412	281	144252	66.6830	72073	33.3170	543.6135	4386	95.882
Haploflow	–	–	–	–	–	–	–	–	–
IRMA	20	15	173187	23.4435	565554	76.5565	11664.8333	114	99.004
LAZYPIPE	–	–	–	–	–	–	–	–	–
metaSPAdes	5	5	779	100.0000	0	0.0000	155.8000	9	98.845
metaviralSPAdes	–	–	–	–	–	–	–	–	–
PEHaplo	234	156	121381	65.6075	63630	34.3925	807.7541	860	99.135
QuRe	–	–	–	–	–	–	–	–	–
QVG	–	–	–	–	–	–	–	–	–
SPAdes	–	–	–	–	–	–	–	–	–
SSAKE	–	–	–	–	–	–	–	–	–
TRACESPipe	–	–	–	–	–	–	–	–	–
TRACESPipeLite	1	0	0	0.0000	5185	100.0000	0.0000	0	0.000
V-pipe	5	0	0	0.0000	160429	100.0000	0.0000	0	0.000
VirGenA	–	–	–	–	–	–	–	–	–
ViSpA	5	4	1920559	98.5150	28951	1.4850	6477.5714	1216	96.587

Table S147: Results obtained by dnadiff [6] when analysing the reconstruction of DS70. Only one execution of HVRS is represented.

Reconstruction tool	Total seqs	Aligned seqs	Aligned bases	Aligned bases (%)	Unaligned bases	Unaligned bases (%)	Average length	SNPs	Identity
coronaSPAdes	9	7	135980	67.1092	66645	32.8908	19407.4286	60	99.906
Haploflow	24	18	259437	66.3403	131633	33.6597	13548.3000	66	99.920
IRMA	20	15	174546	23.5841	565553	76.4159	25668.6000	61	99.941
LAZYPIPE	7	5	136015	67.1682	66484	32.8318	17061.5000	62	99.938
metaSPAdes	–	–	–	–	–	–	–	–	–
metaviralSPAdes	–	–	–	–	–	–	–	–	–
PEHaplo	233	161	221725	69.2093	98644	30.7907	1380.3209	49	99.933
QuRe	5	4	7545	93.7966	499	6.2034	1886.2500	1	99.986
QVG	5	4	143861	89.6721	16569	10.3279	35965.2500	35	99.970
SPAdes	–	–	–	–	–	–	–	–	–
SSAKE	616	442	325331	67.8722	153998	32.1278	1028.4013	31	99.968
TRACESPipe	4	4	143860	100.0000	0	0.0000	35965.0000	2	99.998
TRACESPipeLite	6	1	561	0.3328	168032	99.6672	443.5000	0	95.140
V-pipe	5	4	143596	89.5075	16833	10.4925	35899.0000	60	99.960
VirGenA	28	28	21001	97.6745	500	2.3255	569.8108	8	99.905
ViSpA	5	4	143689	89.6829	16530	10.3171	35922.2500	62	99.941

Table S148: Results obtained by dnadiff [6] when analysing the reconstruction of DS71. Only one execution of HVRS is represented.

Reconstruction tool	Total seqs	Aligned seqs	Aligned bases	Aligned bases (%)	Unaligned bases	Unaligned bases (%)	Average length	SNPs	Identity
coronaSPAdes	15	12	137169	67.2615	66765	32.7385	17008.8750	59	99.837
Haploflow	66	43	151738	64.0731	85082	35.9269	3804.9167	72	99.927
IRMA	20	15	174546	23.5841	565553	76.4159	25668.6000	61	99.941
LAZYPIPE	7	5	136124	67.1776	66509	32.8224	16312.8889	51	99.856
metaSPAdes	10	8	135971	67.1110	66635	32.8890	13604.7000	59	99.843
metaviralSPAdes	1	0	0	0.0000	16543	100.0000	0.0000	0	0.000
PEHaplo	186	127	212720	69.5073	93320	30.4927	1681.6429	34	99.943
QuRe	7	6	28024	90.7631	2852	9.2369	3774.3333	4	99.964
QVG	–	–	–	–	–	–	–	–	–
SPAdes	10	8	136818	67.2747	66554	32.7253	20483.0000	56	99.775
SSAKE	142	97	67758	71.1295	27502	28.8705	746.2561	59	99.837
TRACESPipe	4	4	143813	99.9625	54	0.0375	35953.2500	60	99.949
TRACESPipeLite	6	1	587	0.3506	166838	99.6494	456.5000	1	99.122
V-pipe	5	0	0	0.0000	160429	100.0000	0.0000	0	0.000
VirGenA	11	11	10180	87.2696	1485	12.7304	381.2963	17	99.671
ViSpA	5	4	143664	89.6807	16531	10.3193	35916.0000	61	99.931

Table S149: Results obtained by dnadiff [6] when analysing the reconstruction of DS72. Only one execution of HVRS is represented.

Reconstruction tool	Total seqs	Aligned seqs	Aligned bases	Aligned bases (%)	Unaligned bases	Unaligned bases (%)	Average length	SNPs	Identity
coronaSPAdes	50	37	142847	67.3464	69261	32.6536	17584.3750	97	99.770
Haploflow	29	21	19866	77.3297	5824	22.6703	946.0000	4	99.980
IRMA	20	15	174546	23.5841	565553	76.4159	25668.6000	61	99.941
LAZYPIPE	15	12	138697	67.4787	66845	32.5213	12293.8333	79	99.817
metaSPAdes	10	8	135965	67.1296	66576	32.8704	13604.1000	57	99.840
metaviralSPAdes	1	0	0	0.0000	16543	100.0000	0.0000	0	0.000
PEHaplo	231	148	217945	68.5899	99806	31.4101	1496.5645	35	99.939
QuRe	7	6	30536	97.5186	777	2.4814	4603.0000	7	99.963
QVG	–	–	–	–	–	–	–	–	–
SPAdes	10	8	136857	67.2800	66557	32.7200	18386.2500	51	99.789
SSAKE	–	–	–	–	–	–	–	–	–
TRACESPipe	4	4	143811	99.9625	54	0.0375	35952.7500	63	99.948
TRACESPipeLite	6	1	587	0.3506	166838	99.6494	456.5000	1	95.174
V-pipe	5	0	0	0.0000	160429	100.0000	0.0000	0	0.000
VirGenA	–	–	–	–	–	–	–	–	–
ViSpA	5	4	143681	89.6812	16532	10.3188	35920.2500	57	99.931

Table S150: Results obtained by dnadiff [6] when analysing the reconstruction of DS73. Only one execution of HVRS is represented.

Reconstruction tool	Total seqs	Aligned seqs	Aligned bases	Aligned bases (%)	Unaligned bases	Unaligned bases (%)	Average length	SNPs	Identity
coronaSPAdes	139	94	154786	66.9053	76565	33.0947	7209.9474	621	99.483
Haploflow	–	–	–	–	–	–	–	–	–
IRMA	20	15	174546	23.5841	565553	76.4159	25668.6000	61	99.941
LAZYPIPE	–	–	–	–	–	–	–	–	–
metaSPAdes	148	100	151127	67.1395	73967	32.8605	2322.4918	822	98.968
metaviralSPAdes	–	–	–	–	–	–	–	–	–
PEHaplo	188	111	204055	67.6494	97581	32.3506	1898.2292	30	99.944
QuRe	5	4	1636	66.1812	836	33.8188	398.3333	1	99.916
QVG	–	–	–	–	–	–	–	–	–
SPAdes	20	15	137820	67.4144	66617	32.5856	19449.5714	300	99.633
SSAKE	–	–	–	–	–	–	–	–	–
TRACESPipe	–	–	–	–	–	–	–	–	–
TRACESPipeLite	6	1	523	0.3120	167131	99.6880	523.0000	8	84.840
V-pipe	5	0	0	0.0000	160429	100.0000	0.0000	0	0.000
VirGenA	–	–	–	–	–	–	–	–	–
ViSpA	5	4	143697	89.6817	16533	10.3183	35924.2500	60	99.921

2.2 Real datasets

Table S151: Viral sequences included in each real dataset according to FALCON-meta [7] (top of similarity value set to 8,000) and the total number of reference genomes found per dataset.

Virus	SRR23101281	SRR23101235	SRR23101259	SRR23101276	SRR23101228	SRR12175231
B19V	x	x	x	x	x	
BKPyV	x	x	x	x	x	x
BuV	x	x	x	x	x	x
CuV	x	x	x	x	x	x
EBV	x	x	x	x		
EV	x	x	x	x	x	x
HAV		x	x	x	x	x
HBoV	x					
HCMV					x	
HDV	x	x	x	x		x
HERV	x	x	x	x	x	x
HHV6	x	x		x	x	
HHV7	x	x	x	x	x	x
HPV16	x	x	x	x	x	x
HPV2	x					
HPV31	x				x	
HPV51	x					
HPV58	x					
HPV6	x	x		x	x	x
HPyV12	x	x	x	x	x	x
HPyV6	x	x	x	x		
HPyV7	x	x	x	x	x	x
HPyV9		x	x	x	x	x
HSV-1	x	x		x		
HSV-2	x	x	x	x		
JCPyV	x	x	x	x	x	x
KIPyV	x	x	x		x	x
LIPyV	x	x	x	x	x	
MCPyV	x	x	x	x	x	x
MT	x	x	x	x	x	x
MWPyV	x	x	x	x	x	x
NJPyV	x	x	x	x	x	x
ReDoV						x
SENV	x					
STLPyV		x	x	x	x	x
SV40	x	x	x	x	x	x
TSPyV	x	x	x	x	x	x
TTV	x	x	x	x	x	x
TTVmid	x	x	x	x	x	x
TTVmin	x	x	x	x	x	x
WUPyV	x		x		x	
Total number of references	36	32	30	31	30	26

Table S152: Viral sequences found by FALCON-meta in the dataset SRR23101281, along with their GC content and compression ratio. The compression of the sequences was made using GeCo3 [5].

**Sequence identification**	**GC content (%)**	**Compression ratio (GeCo3)**
>FJ591158.1 Human parvovirus B19 isolate KU1, complete genome	0.433296	0.253094
>AB211390.1 BK polyomavirus DNA, complete genome, isolate: THK-8	0.391296	0.256329
>AB847989.1 Bufavirus-3 genes for NS1, putative VP1, hypothetical protein, VP2, partial and complete cds, strain: BTN-310	0.3953	0.251311
>KT868810.1 Cutavirus strain BR-283 NS1 gene, partial cds; and putative VP1, hypothetical protein, VP2, and hypothetical protein genes, complete cds	0.390703	0.253098
>MG021310.1 Human gammaherpesvirus 4 isolate Mutu-GC3, complete genome	0.594484	0.208357
>MH484165.1 Enterovirus C96 isolate 3499BRA-PA10, complete genome	0.436925	0.252009
>HM132056.1 Human bocavirus 3 isolate 46-BJ07, complete genome	0.420832	0.260729
>MH791030.1 Hepatitis delta virus isolate 63, complete genome	0.591423	0.282143
>AY037929.1 Human endogenous retrovirus K115 complete genome	0.418049	0.240279
>NC_000898.1 Human herpesvirus 6B, complete genome	0.427711	0.228603
>NC_001716.2 Human herpesvirus 7, complete genome	0.36219	0.218126
>JQ004092.1 Human papillomavirus type 16 strain CU8, complete genome	0.365693	0.250632
>EF117891.1 Human papillomavirus type 2 isolate CN-HB1, complete genome	0.482886	0.257379
>HQ537675.1 Human papillomavirus type 31 isolate IN221709, complete genome	0.369541	0.250818
>LR861992.1 Human papillomavirus type 51 isolate LNS6584190_HPV51, complete genome	0.390453	0.251951
>KY225957.1 Human papillomavirus 58 isolate ZWE043998, complete genome	0.376997	0.250958
>MK463906.1 Human papillomavirus type 6 isolate HPV6-gw-0605, complete genome	0.408036	0.253275
>NC_020890.1 Human polyomavirus 12 strain hu1403, complete genome	0.395986	0.259039
>KM655817.1 Human polyomavirus 6 isolate QLD-61Br, complete genome	0.4257	0.260199
>HM011564.1 Polyomavirus HPyV7 isolate 707a, complete genome	0.426349	0.258384
>MH999850.1 Human alphaherpesvirus 1 strain HSV-H15119, complete genome	0.680496	0.21049
>NC_001798.1 Human herpesvirus 2, complete genome	0.703857	0.210873
>AF281599.1 JC virus strain 125B agnoprotein, VP2, VP3, VP1, large T antigen, and small t antigen genes, complete cds	0.39967	0.258496
>NC_009238.1 KI polyomavirus Stockholm 60, complete genome	0.393651	0.257687
>KY404016.1 LI polyomavirus isolate LIPyV, complete genome	0.398558	0.258254
>JF813001.1 Merkel cell polyomavirus isolate 352, complete genome	0.407522	0.257231
>NC_012920.1 Homo sapiens mitochondrion, complete genome	0.443599	0.245866
>KC571703.1 MW polyomavirus isolate HB087C, complete genome	0.368975	0.25734
>NC_024118.1 New Jersey polyomavirus-2013 isolate NJ-PyV-2013, complete genome	0.392522	0.25778
>KM593803.2 SEN virus strain HDMU-97, complete genome	0.534115	0.264514
>AF345345.1 Simian virus 40 strain GM00637H defective variant 12, complete genome	0.407582	0.25803
>KM007161.1 Trichodysplasia spinulosa-associated polyomavirus isolate TSV_Br, complete genome	0.402292	0.25783
>MN768089.1 MAG: Torque teno virus isolate SAfiA-608-30 ORF1 gene, complete cds	0.494667	0.26891
>MN776672.1 MAG: Torque teno midi virus isolate SAfiA-587-291 ORF1 gene, complete cds	0.378368	0.267369
>MN771265.1 MAG: TTV-like mini virus isolate SAfiA-578-13, complete genome	0.38739	0.261207
>EU296475.1 WU Polyomavirus strain CLFF, complete genome	0.392044	0.256023

Table S153: Viral sequences found by FALCON-meta in the dataset SRR23101235, along with their GC content and compression ratio. The compression of the sequences was made using GeCo3 [5].

**Sequence identification**	**GC content (%)**	**Compression ratio (GeCo3)**
>MT410184.1 Human parvovirus B19 isolate AA-01, complete genome	0.436292	0.247987
>AB263916.1 BK polyomavirus DNA, complete genome, isolate: ETH-3	0.390619	0.256081
>AB847989.1 Bufavirus-3 genes for NS1, putative VP1, hypothetical protein, VP2, partial and complete cds, strain: BTN-310	0.3953	0.251311
>KX685945.1 Cutavirus strain CGG5-268 NS1, VP1, hypothetical protein, and VP2 genes, complete cds	0.391959	0.252414
>MG021306.1 Human gammaherpesvirus 4 isolate YCCEL1-GC2, complete genome	0.599659	0.202577
>MH484165.1 Enterovirus C96 isolate 3499BRA-PA10, complete genome	0.436925	0.252009
>LC373510.1 Human hepatitis A virus HA16-1496 genomic RNA, complete genome	0.379568	0.2511
>KM110801.1 Hepatitis delta virus isolate SOG, complete genome	0.599762	0.278932
>AY037929.1 Human endogenous retrovirus K115 complete genome	0.418049	0.240279
>NC_000898.1 Human herpesvirus 6B, complete genome	0.427711	0.228603
>NC_001716.2 Human herpesvirus 7, complete genome	0.36219	0.218126
>JQ004099.1 Human papillomavirus type 16 strain CU15, complete genome	0.364294	0.250698
>MK463907.1 Human papillomavirus type 6 isolate HPV6-gw-0607, complete genome	0.40933	0.253595
>NC_020890.1 Human polyomavirus 12 strain hu1403, complete genome	0.395986	0.259039
>KR090570.1 Human polyomavirus 6 isolate QLD-49Br, complete genome	0.428542	0.260402
>HM011566.1 Polyomavirus HPyV7 isolate 713a, complete genome	0.428918	0.258429
>FR823284.2 Human polyomavirus IPPyV complete genome, isolate 915 F 06 001 PJ	0.391408	0.257904
>JN555585.1 Human herpesvirus 1 strain 17, complete genome	0.683009	0.213575
>NC_001798.1 Human herpesvirus 2, complete genome	0.703857	0.210873
>MF662187.1 JC polyomavirus strain 8, complete genome	0.404725	0.257466
>NC_009238.1 KI polyomavirus Stockholm 60, complete genome	0.393651	0.257687
>KY404016.1 LI polyomavirus isolate LIPyV, complete genome	0.398558	0.258254
>HM011544.1 Merkel cell polyomavirus isolate 14a, complete genome	0.406343	0.256629
>NC_012920.1 Homo sapiens mitochondrion, complete genome	0.443599	0.245866
>KC571700.1 MW polyomavirus isolate HB017C, complete genome	0.365202	0.257189
>NC_024118.1 New Jersey polyomavirus-2013 isolate NJ-PyV-2013, complete genome	0.392522	0.25778
>NC_020106.1 STL polyomavirus strain MA138, complete genome	0.366206	0.257693
>AY271816.1 Simian virus 40 strain PML-1EK, complete genome	0.40631	0.257165
>KM007161.1 Trichodysplasia spinulosa-associated polyomavirus isolate TSV_Br, complete genome	0.402292	0.25783
>MZ286238.1 Anelloviridae sp. isolate SP13_C2, complete genome	0.440636	0.261548
>MN778812.1 MAG: Torque teno midi virus isolate SAfiA-611-265 ORF1 gene, complete cds	0.375769	0.265283
>MN771265.1 MAG: TTV-like mini virus isolate SAfiA-578-13, complete genome	0.38739	0.261207

Table S154: Viral sequences found by FALCON-meta in the dataset SRR23101259, along with their GC content and compression ratio. The compression of the sequences was made using GeCo3 [5].

**Sequence identification**	**GC content (%)**	**Compression ratio (GeCo3)**
>MT410186.1 Human parvovirus B19 isolate AA-03 nonstructural protein (NS1) and capsid protein 1 (VP1) genes, complete cds	0.441171	0.257271
>AB269865.1 BK polyomavirus DNA, complete genome, strain: SWC-4	0.394481	0.256266
>AB847988.1 Bufavirus-3 genes for NS1, putative VP1, hypothetical protein, VP2, partial and complete cds, strain: BTN-109	0.396071	0.25132
>KX685945.1 Cutavirus strain CGG5-268 NS1, VP1, hypothetical protein, and VP2 genes, complete cds	0.391959	0.252414
>MG021306.1 Human gammaherpesvirus 4 isolate YCCEL1-GC2, complete genome	0.599659	0.202577
>MH484165.1 Enterovirus C96 isolate 3499BRA-PA10, complete genome	0.436925	0.252009
>LC373510.1 Human hepatitis A virus HA16-1496 genomic RNA, complete genome	0.379568	0.2511
>KJ744238.1 Hepatitis delta virus isolate D28, complete genome	0.588551	0.282479
>NC_022518.1 Human endogenous retrovirus K113 complete genome	0.418602	0.237623
>NC_001716.2 Human herpesvirus 7, complete genome	0.36219	0.218126
>JQ004092.1 Human papillomavirus type 16 strain CU8, complete genome	0.365693	0.250632
>NC_020890.1 Human polyomavirus 12 strain hu1403, complete genome	0.395986	0.259039
>KX379631.1 Human polyomavirus 6 isolate Bile-81, complete genome	0.430166	0.260199
>HM011564.1 Polyomavirus HPyV7 isolate 707a, complete genome	0.426349	0.258384
>FR823284.2 Human polyomavirus IPPyV complete genome, isolate 915 F 06 001 PJ	0.391408	0.257904
>NC_001798.1 Human herpesvirus 2, complete genome	0.703857	0.210873
>AB092585.1 JC virus DNA, complete genome, isolate:AN-11	0.402772	0.257466
>NC_009238.1 KI polyomavirus Stockholm 60, complete genome	0.393651	0.257687
>KY404016.1 LI polyomavirus isolate LIPyV, complete genome	0.398558	0.258254
>HM011548.1 Merkel cell polyomavirus isolate 16b, complete genome	0.404652	0.256183
>NC_012920.1 Homo sapiens mitochondrion, complete genome	0.443599	0.245866
>KC571701.1 MW polyomavirus isolate HB039C, complete genome	0.369988	0.257542
>NC_024118.1 New Jersey polyomavirus-2013 isolate NJ-PyV-2013, complete genome	0.392522	0.25778
>KR090571.1 STL polyomavirus isolate QLD-13M6r, complete genome	0.366492	0.257538
>EF579659.1 Simian virus 40 isolate SV40(Ad)201, complete genome	0.405985	0.257286
>KM007161.1 Trichodysplasia spinulosa-associated polyomavirus isolate TSV_Br, complete genome	0.402292	0.25783
>MZ286238.1 Anelloviridae sp. isolate SP13_C2, complete genome	0.440636	0.261548
>MN778535.1 MAG: Torque teno midi virus isolate SAfiA-429-25 ORF1 gene, complete cds	0.377463	0.267428
>MN771265.1 MAG: TTV-like mini virus isolate SAfiA-578-13, complete genome	0.38739	0.261207
>EF444549.1 WU Polyomavirus strain B0, complete genome	0.391662	0.256023

Table S155: Viral sequences found by FALCON-meta in the dataset SRR23101276, along with their GC content and compression ratio. The compression of the sequences was made using GeCo3 [5].

Sequence identification	GC content (%)	Compression ratio (GeCo3)
>MT410186.1 Human parvovirus B19 isolate AA-03 nonstructural protein (NS1) and capsid protein 1 (VP1) genes, complete cds	0.441171	0.257271
>AB365171.1 BK polyomavirus DNA, complete genome, isolate: SHA-43	0.394675	0.256072
>AB982222.1 Bufavirus-3 genes for NS1, VP1, VP2, complete cds, strain: AHP-740	0.397682	0.252002
>NC_039050.1 Cutavirus strain BR-337 NS1, putative VP1, hypothetical protein, VP2, and hypothetical protein genes, complete cds	0.389138	0.251963
>MG021308.1 Human gammaherpesvirus 4 isolate Mutu-GC1, complete genome	0.594794	0.208138
>MH484165.1 Enterovirus C96 isolate 3499BRA-PA10, complete genome	0.436925	0.252009
>LC373510.1 Human hepatitis A virus HA16-1496 genomic RNA, complete genome	0.379568	0.2511
>MN984457.1 Hepatitis delta virus isolate HDV_Kyr28, complete genome	0.586453	0.280879
>NC_022518.1 Human endogenous retrovirus K113 complete genome	0.418602	0.237623
>NC_000898.1 Human herpesvirus 6B, complete genome	0.427711	0.228603
>NC_001716.2 Human herpesvirus 7, complete genome	0.36219	0.218126
>JN565303.1 Human papillomavirus type 16 strain ZG01-258, complete genome	0.367352	0.250345
>MK463908.1 Human papillomavirus type 6 isolate HPV6-gw-0609, complete genome	0.408954	0.253595
>NC_020890.1 Human polyomavirus 12 strain hu1403, complete genome	0.395986	0.259039
>KR090570.1 Human polyomavirus 6 isolate QLD-49Br, complete genome	0.428542	0.260402
>MG674199.1 Human polyomavirus 7 isolate CRC01, complete genome	0.428946	0.258527
>FR823284.2 Human polyomavirus IPPyV complete genome, isolate 915 F 06 001 PJ	0.391408	0.257904
>JN555585.1 Human herpesvirus 1 strain 17, complete genome	0.683009	0.213575
>NC_001798.1 Human herpesvirus 2, complete genome	0.703857	0.210873
>AB038252.1 JC polyomavirus DNA, complete genome, isolate: GH-1	0.403286	0.257137
>KY404016.1 LI polyomavirus isolate LIPyV, complete genome	0.398558	0.258254
>KJ128376.1 Merkel cell polyomavirus isolate AlDo capsid protein VP1 (VP1) gene, partial cds; and capsid protein VP2 (VP2), truncated large T antigen (LT), and small T antigen (ST) genes, complete cds	0.397408	0.257362
>NC_012920.1 Homo sapiens mitochondrion, complete genome	0.443599	0.245866
>KC571701.1 MW polyomavirus isolate HB039C, complete genome	0.369988	0.257542
>NC_024118.1 New Jersey polyomavirus-2013 isolate NJ-PyV-2013, complete genome	0.392522	0.25778
>NC_020106.1 STL polyomavirus strain MA138, complete genome	0.366206	0.257693
>AF168994.2 Simian virus 40 strain T302, complete genome	0.406753	0.25747
>KM007161.1 Trichodysplasia spinulosa-associated polyomavirus isolate TSV_Br, complete genome	0.402292	0.25783
>MZ286238.1 Anelloviridae sp. isolate SP13_C2, complete genome	0.440636	0.261548
>MN777551.1 MAG: Torque teno midi virus isolate SAfiA-35-91 ORF1 gene, complete cds	0.365789	0.261556
>MN771265.1 MAG: TTV-like mini virus isolate SAfiA-578-13, complete genome	0.38739	0.261207

Table S156: Viral sequences found by FALCON-meta in the dataset SRR23101228, along with their GC content and compression ratio. The compression of the sequences was made using GeCo3 [5].

Sequence identification	GC content (%)	Compression ratio (GeCo3)
>AB030673.1 Human parvovirus B19 NS1, VP1, VP2 genes for non-structural protein NS1, capsid protein VP1, capsid protein VP2, complete cds, isolate: N8	0.420052	0.261396
>AB211388.1 BK polyomavirus DNA, complete genome, isolate: KOM-7	0.395131	0.256425
>AB982221.1 Bufavirus-3 genes for NS1, VP1, VP2, complete cds, strain: AHP-692	0.397393	0.250788
>KX685945.1 Cutavirus strain CGG5-268 NS1, VP1, hypothetical protein, and VP2 genes, complete cds	0.391959	0.252414
>MH484165.1 Enterovirus C96 isolate 3499BRA-PA10, complete genome	0.436925	0.252009
>LC373510.1 Human hepatitis A virus HA16-1496 genomic RNA, complete genome	0.379568	0.2511
>GQ221974.1 Human herpesvirus 5 strain 3157, complete genome	0.574836	0.241428
>NC_022518.1 Human endogenous retrovirus K113 complete genome	0.418602	0.237623
>NC_000898.1 Human herpesvirus 6B, complete genome	0.427711	0.228603
>NC_001716.2 Human herpesvirus 7, complete genome	0.36219	0.218126
>JQ004092.1 Human papillomavirus type 16 strain CU8, complete genome	0.365693	0.250632
>HQ537667.1 Human papillomavirus type 31 isolate QV18335, complete genome	0.371212	0.251105
>JN252314.1 Human papillomavirus type 6 isolate PV2345, complete genome	0.404133	0.253206
>NC_020890.1 Human polyomavirus 12 strain hu1403, complete genome	0.395986	0.259039
>MG674199.1 Human polyomavirus 7 isolate CRC01, complete genome	0.428946	0.258527
>FR823284.2 Human polyomavirus IPPyV complete genome, isolate 915 F 06 001 PJ	0.391408	0.257904
>MF662195.1 JC polyomavirus strain 14, complete genome	0.40578	0.257321
>EU358766.1 KI polyomavirus isolate CU-255, complete genome	0.395446	0.257969
>KY404016.1 LI polyomavirus isolate LIPyV, complete genome	0.398558	0.258254
>KJ128376.1 Merkel cell polyomavirus isolate AlDo capsid protein VP1 (VP1) gene, partial cds; and capsid protein VP2 (VP2), truncated large T antigen (LT), and small T antigen (ST) genes, complete cds	0.397408	0.257362
>NC_012920.1 Homo sapiens mitochondrion, complete genome	0.443599	0.245866
>KC571701.1 MW polyomavirus isolate HB039C, complete genome	0.369988	0.257542
>NC_024118.1 New Jersey polyomavirus-2013 isolate NJ-PyV-2013, complete genome	0.392522	0.25778
>KR090571.1 STL polyomavirus isolate QLD-13M6r, complete genome	0.366492	0.257538
>EF579659.1 Simian virus 40 isolate SV40(Ad)201, complete genome	0.405985	0.257286
>KM007161.1 Trichodysplasia spinulosa-associated polyomavirus isolate TSV_Br, complete genome	0.402292	0.25783
>MZ286238.1 Anelloviridae sp. isolate SP13_C2, complete genome	0.440636	0.261548
>MN778535.1 MAG: Torque teno midi virus isolate SAfiA-429-25 ORF1 gene, complete cds	0.377463	0.267428
>MN771265.1 MAG: TTV-like mini virus isolate SAfiA-578-13, complete genome	0.38739	0.261207
>GU296362.1 WU Polyomavirus isolate O342, complete genome	0.39162	0.256121

Table S157: Viral sequences found by FALCON-meta in the dataset SRR12175231, along with their GC content and compression ratio. The compression of the sequences was made using GeCo3 [5].

Sequence identification	GC content (%)	Compression ratio (GeCo3)
>AB365178.1 BK polyomavirus DNA, complete genome, isolate: SHA-78	0.394481	0.25646
>AB982217.1 Bufavirus-3 genes for NS1, VP1, VP2, complete cds, strain: AHP-178	0.39657	0.251359
>KT868810.1 Cutavirus strain BR-283 NS1 gene, partial cds; and putative VP1, hypothetical protein, VP2, and hypothetical protein genes, complete cds	0.390703	0.253098
>MH484165.1 Enterovirus C96 isolate 3499BRA-PA10, complete genome	0.436925	0.252009
>LC373510.1 Human hepatitis A virus HA16-1496 genomic RNA, complete genome	0.379568	0.2511
>KM110801.1 Hepatitis delta virus isolate SOG, complete genome	0.599762	0.278932
>NC_022518.1 Human endogenous retrovirus K113 complete genome	0.418602	0.237623
>NC_001716.2 Human herpesvirus 7, complete genome	0.36219	0.218126
>JQ004092.1 Human papillomavirus type 16 strain CU8, complete genome	0.365693	0.250632
>MK463906.1 Human papillomavirus type 6 isolate HPV6-gw-0605, complete genome	0.408036	0.253275
>NC_020890.1 Human polyomavirus 12 strain hu1403, complete genome	0.395986	0.259039
>MG674199.1 Human polyomavirus 7 isolate CRC01, complete genome	0.428946	0.258527
>FR823284.2 Human polyomavirus IPPyV complete genome, isolate 915 F 06 001 PJ	0.391408	0.257904
>AB038252.1 JC polyomavirus DNA, complete genome, isolate: GH-1	0.403286	0.257137
>KC571691.1 KI polyomavirus isolate HN057, complete genome	0.394444	0.257885
>KJ128376.1 Merkel cell polyomavirus isolate AlDo capsid protein VP1 (VP1) gene, partial cds; and capsid protein VP2 (VP2), truncated large T antigen (LT), and small T antigen (ST) genes, complete cds	0.397408	0.257362
>NC_012920.1 Homo sapiens mitochondrion, complete genome	0.443599	0.245866
>KC571704.1 MW polyomavirus isolate HN037, complete genome	0.369627	0.25745
>NC_024118.1 New Jersey polyomavirus-2013 isolate NJ-PyV-2013, complete genome	0.392522	0.25778
>MK059772.1 Human gut-associated vientovirus MW, complete genome	0.346028	0.263866
>KM893862.1 STL polyomavirus isolate HB201, complete genome	0.361675	0.257119
>AF168994.2 Simian virus 40 strain T302, complete genome	0.406753	0.25747
>KM007161.1 Trichodysplasia spinulosa-associated polyomavirus isolate TSV_Br, complete genome	0.402292	0.25783
>MZ286238.1 Anelloviridae sp. isolate SP13_C2, complete genome	0.440636	0.261548
>MN778482.1 MAG: Torque teno midi virus isolate SAfiA-131-19 ORF1 gene, complete cds	0.377468	0.266254
>MN771265.1 MAG: TTV-like mini virus isolate SAfiA-578-13, complete genome	0.38739	0.261207

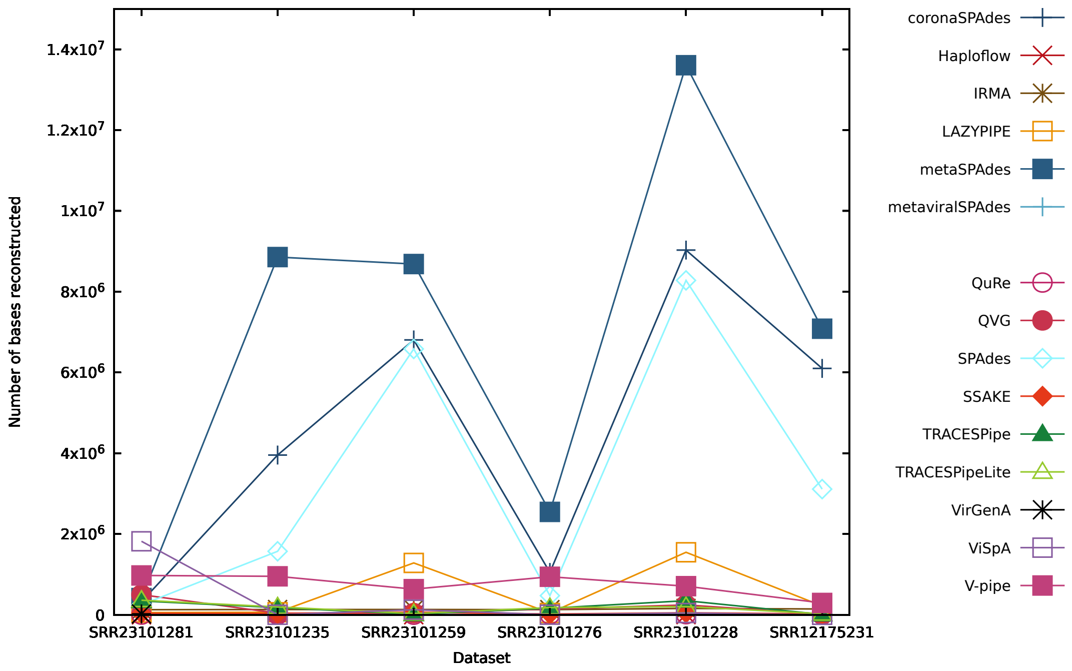

Figure S143: Figure comparing the performance of the reconstruction programs in terms of the number of bases reconstructed for datasets SRR23101281, SRR23101235, SRR23101259, SRR23101276, SRR23101228 and SRR12175231.

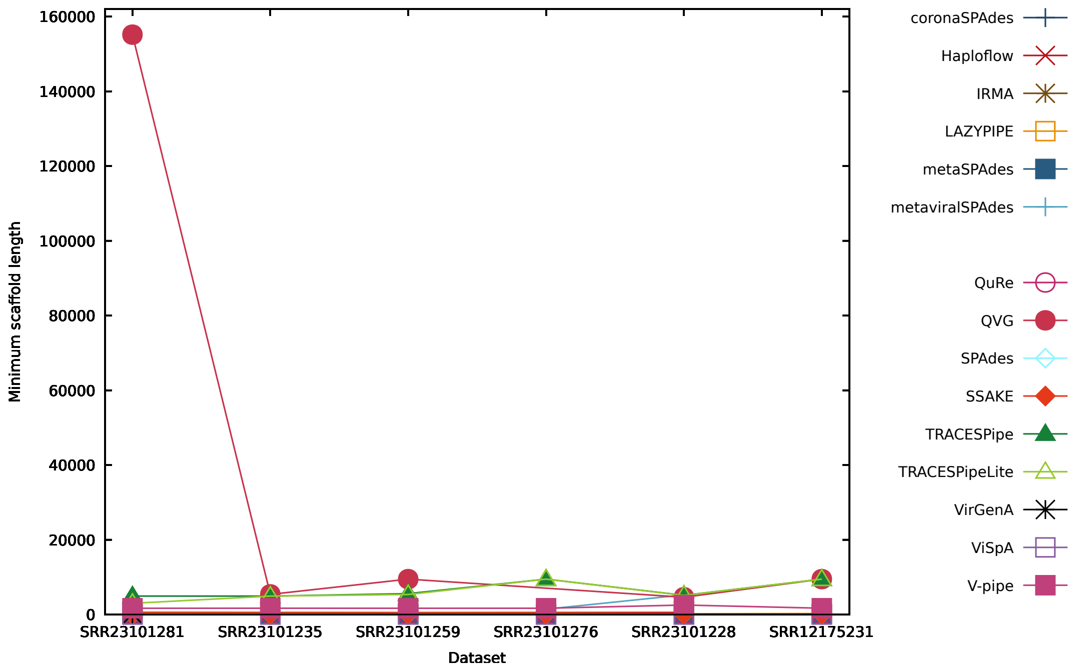

Figure S144: Figure comparing the performance of the reconstruction programs in terms of the minimum number of reconstructed bases per scaffold for datasets SRR23101281, SRR23101235, SRR23101259, SRR23101276, SRR23101228 and SRR12175231.

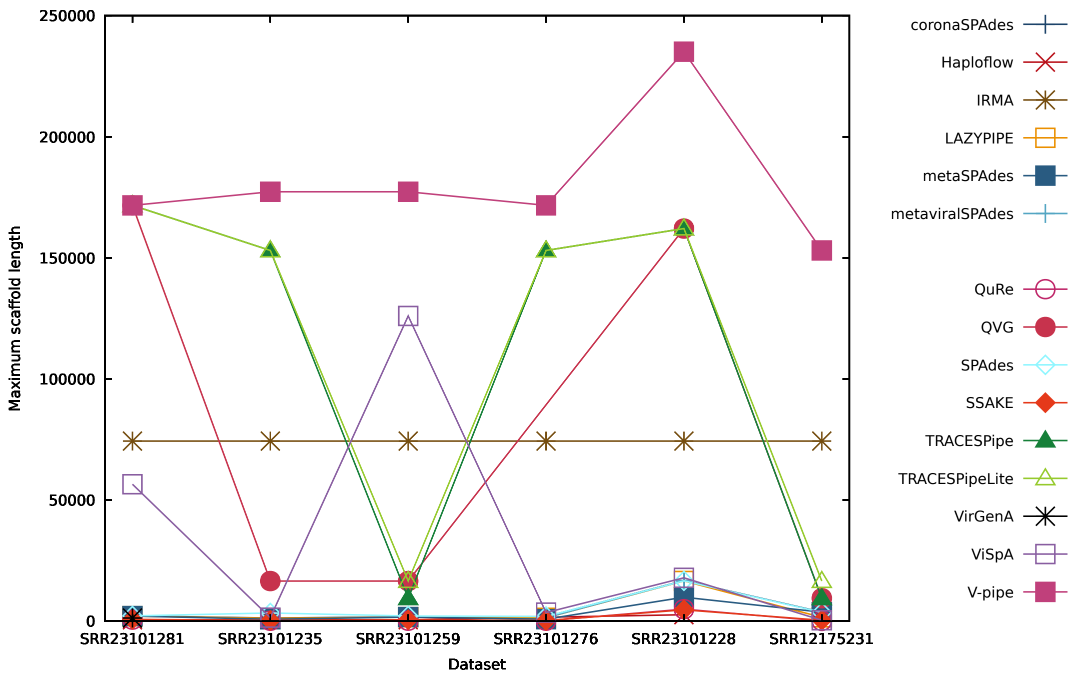

Figure S145: Figure comparing the performance of the reconstruction programs in terms of the maximum number of reconstructed bases per scaffold for datasets SRR23101281, SRR23101235, SRR23101259, SRR23101276, SRR23101228 and SRR12175231.

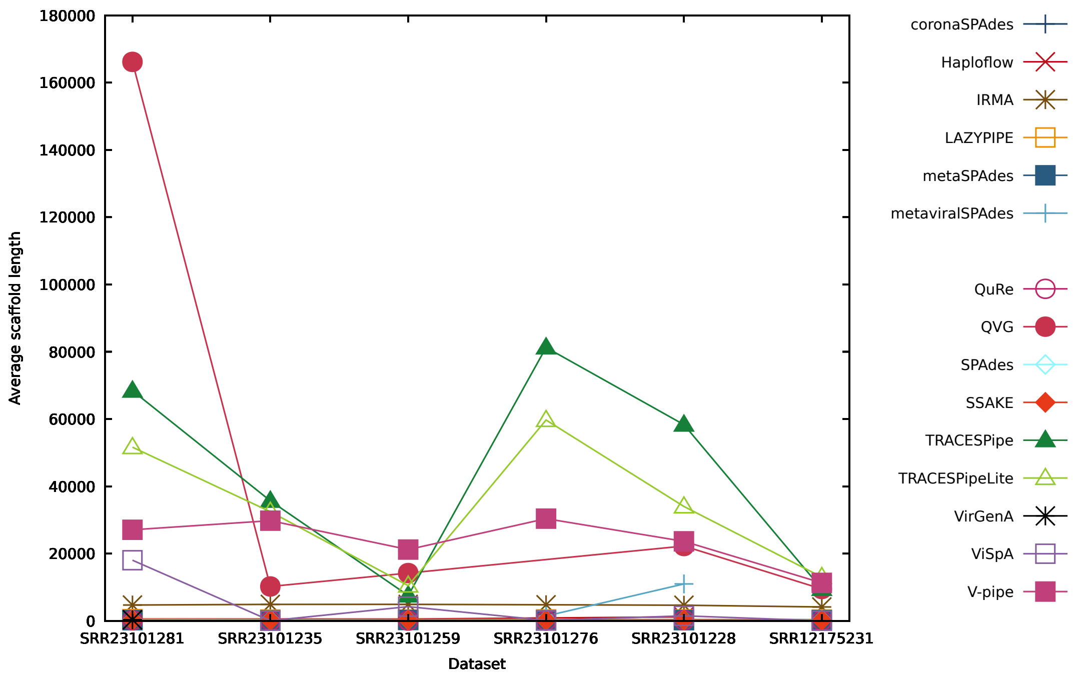

Figure S146: Figure comparing the performance of the reconstruction programs in terms of the average number of reconstructed bases per scaffold for datasets SRR23101281, SRR23101235, SRR23101259, SRR23101276, SRR23101228 and SRR12175231.

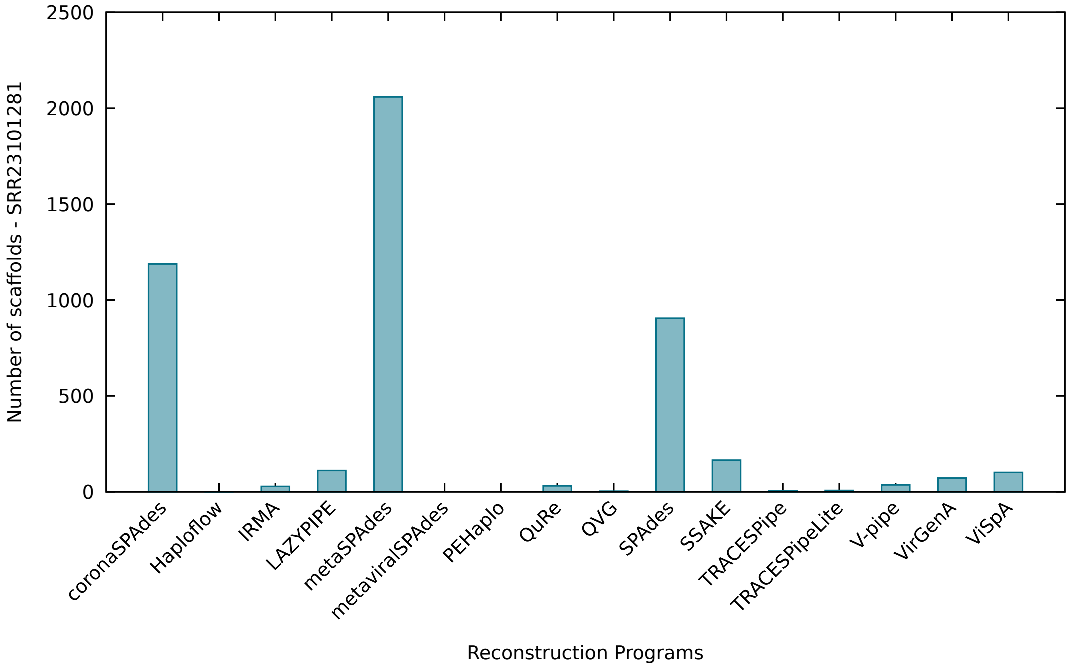

Figure S147: Figure comparing the performance of the reconstruction programs in terms of the number of scaffolds reconstructed in dataset SRR23101281.

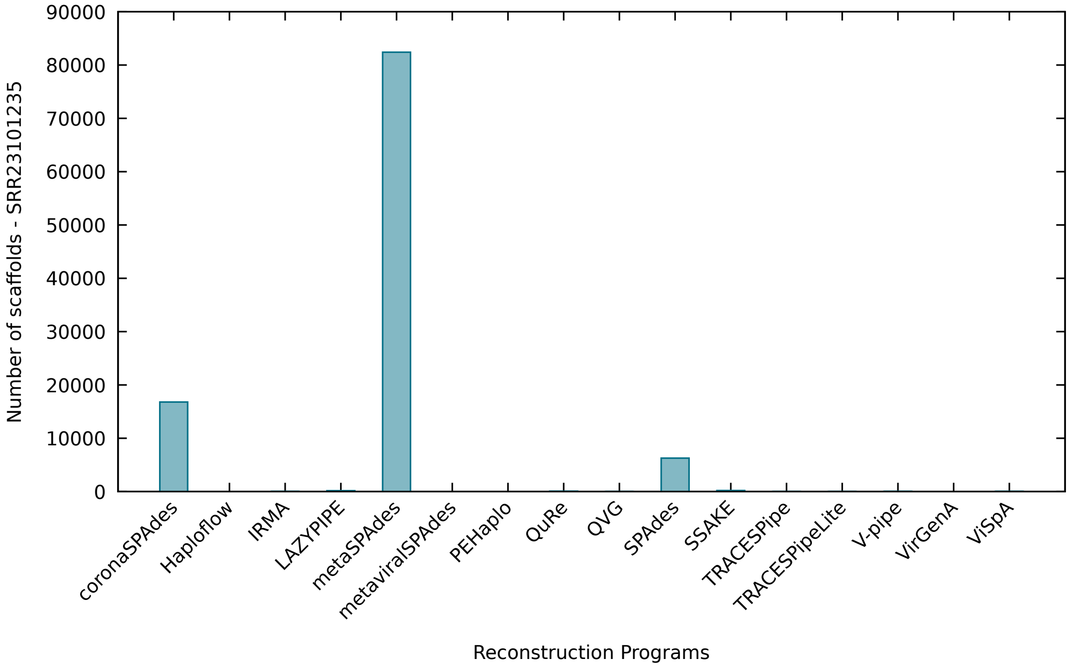

Figure S148: Figure comparing the performance of the reconstruction programs in terms of the number of scaffolds reconstructed in dataset SRR23101235.

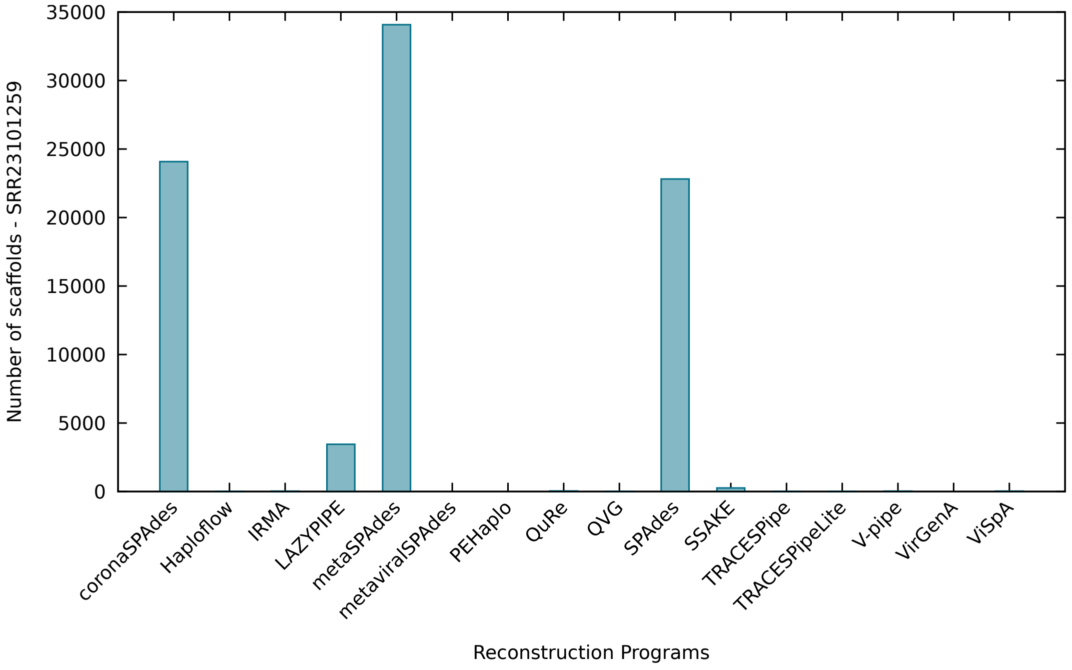

Figure S149: Figure comparing the performance of the reconstruction programs in terms of the number of scaffolds reconstructed in dataset SRR23101259.

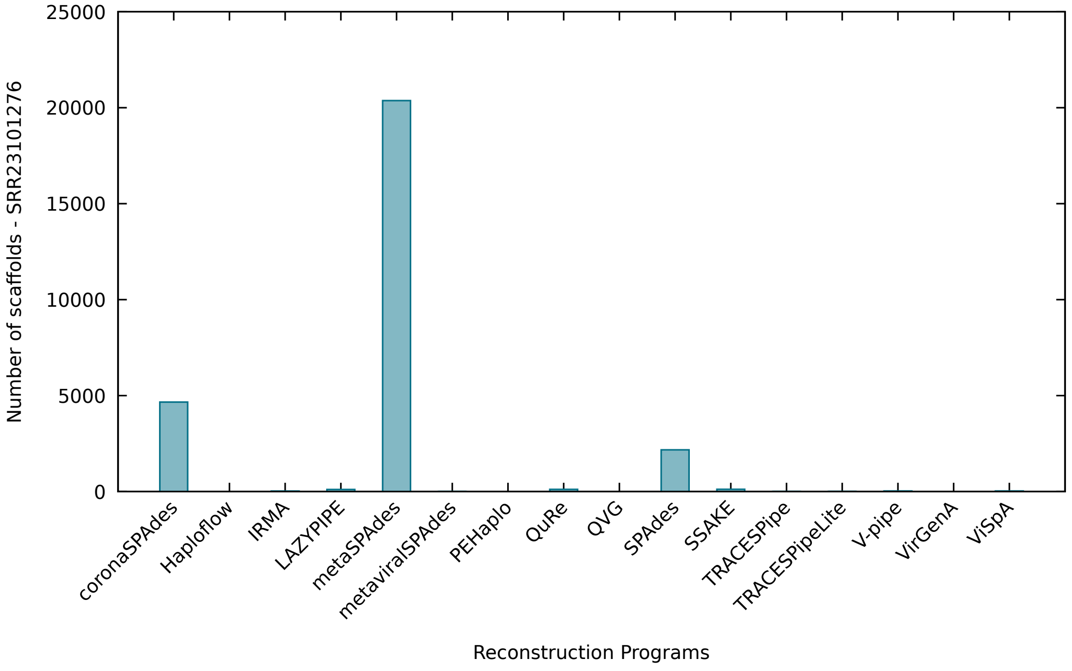

Figure S150: Figure comparing the performance of the reconstruction programs in terms of the number of scaffolds reconstructed in dataset SRR23101276.

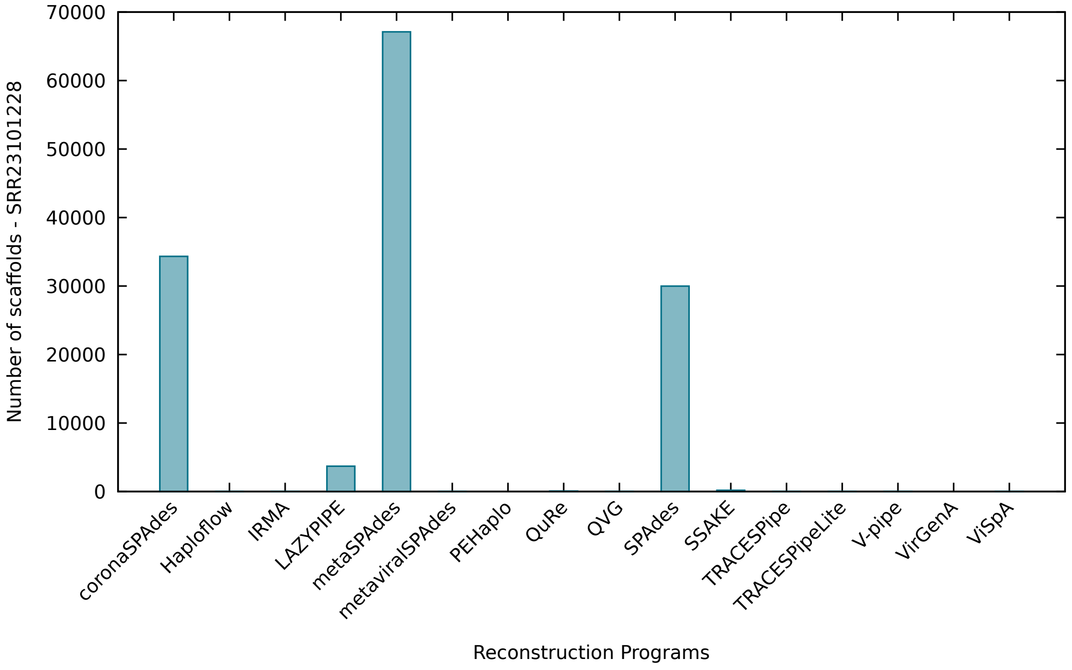

Figure S151: Figure comparing the performance of the reconstruction programs in terms of the number of scaffolds reconstructed in dataset SRR23101228.

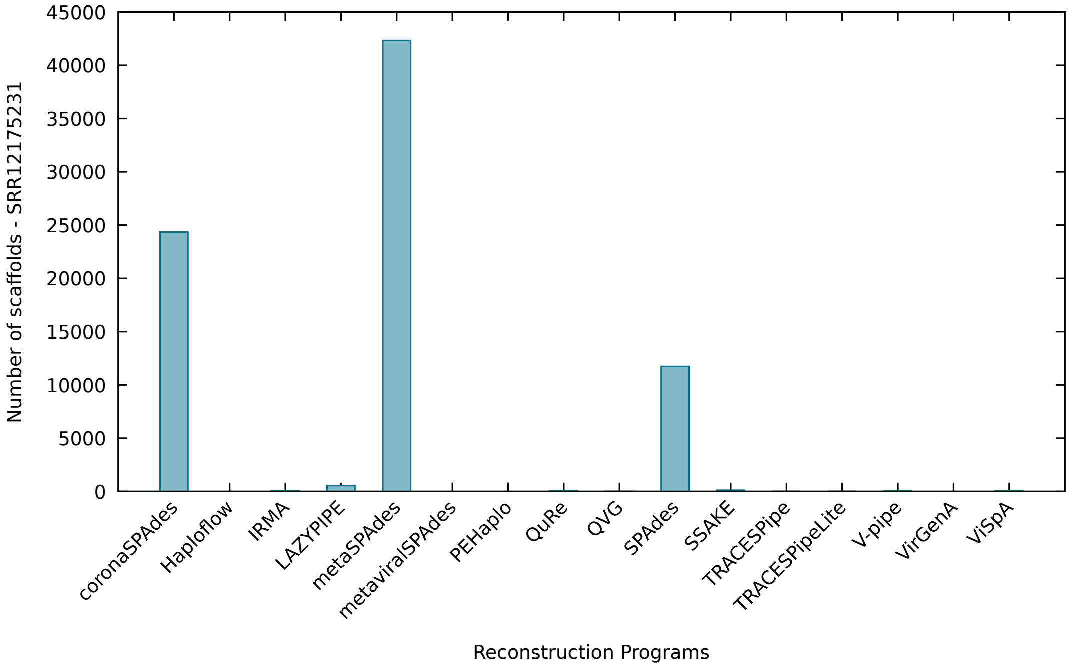

Figure S152: Figure comparing the performance of the reconstruction programs in terms of the number of scaffolds reconstructed in dataset SRR12175231.

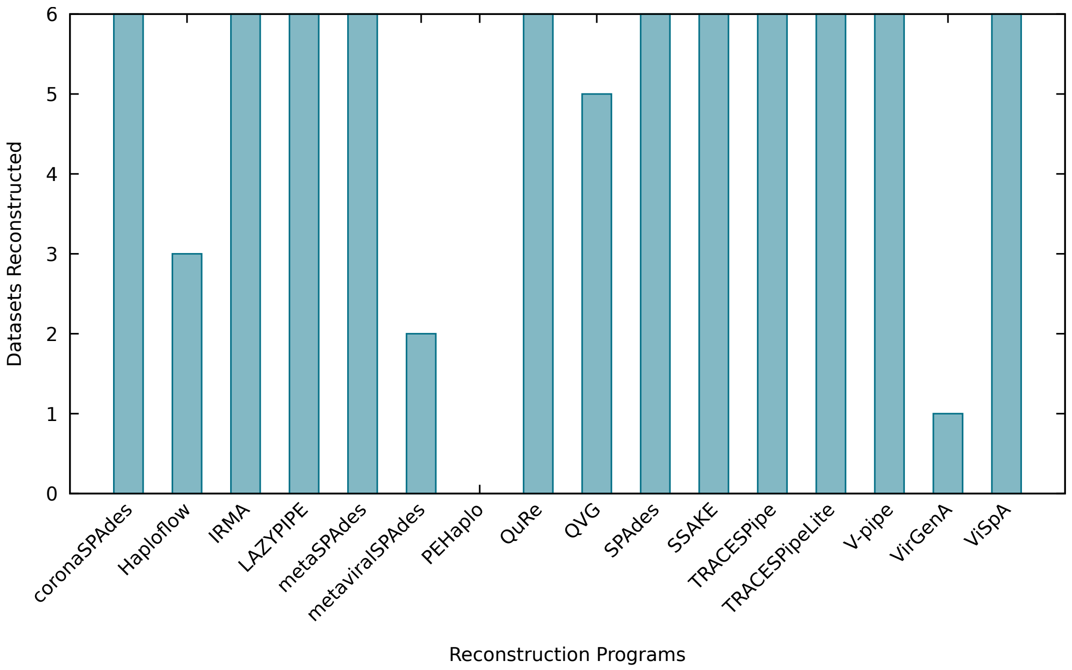

Figure S153: Figure comparing the performance of the reconstruction programs in terms of the number of datasets reconstructed across datasets SRR23101281, SRR23101235, SRR23101259, SRR23101276, SRR23101228 and SRR12175231.

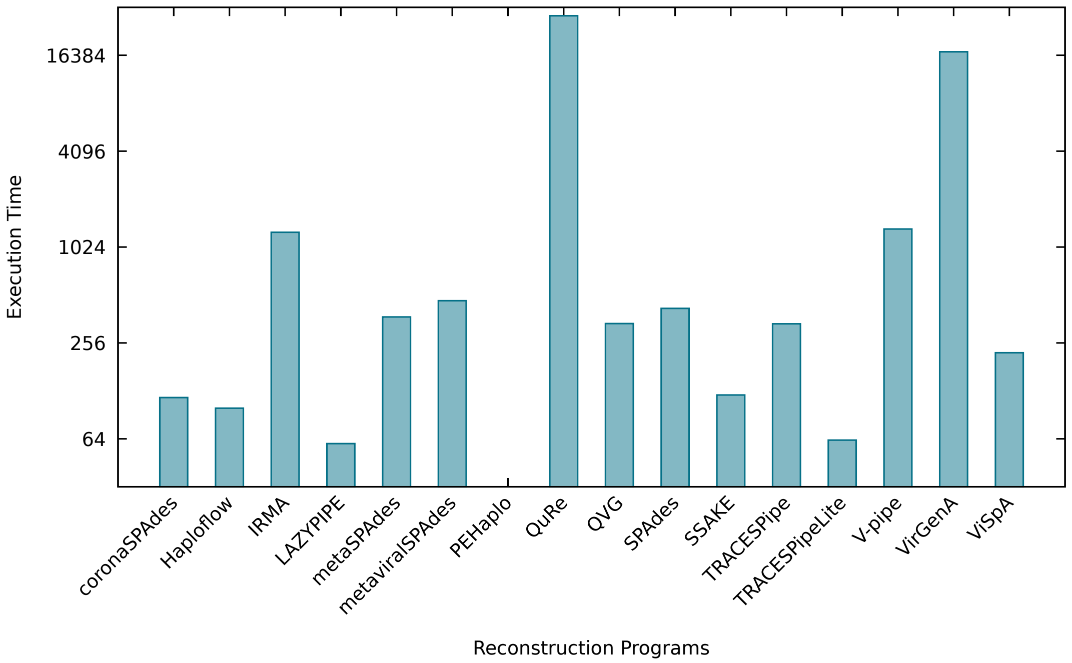

Figure S154: Figure comparing the average performance of the reconstruction programs in terms of the execution time across datasets SRR23101281, SRR23101235, SRR23101259, SRR23101276, SRR23101228 and SRR12175231. The y-axis is presented in a logarithmic scale of base 2.

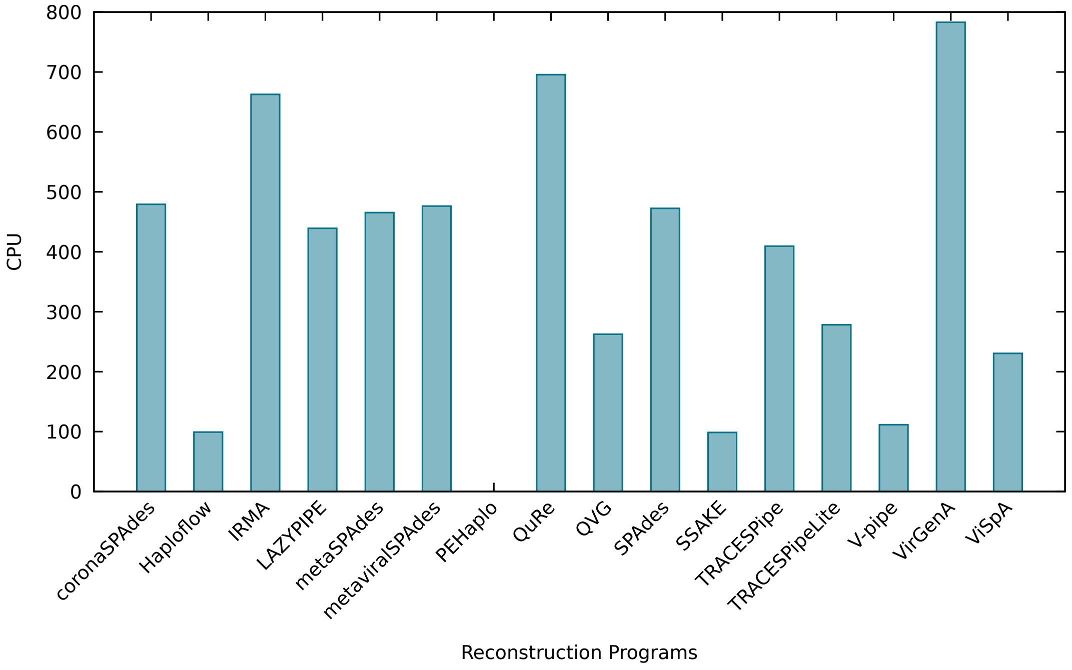

Figure S155: Figure comparing the average performance of the reconstruction programs in terms of the percentage of CPU used across datasets SRR23101281, SRR23101235, SRR23101259, SRR23101276, SRR23101228 and SRR12175231.

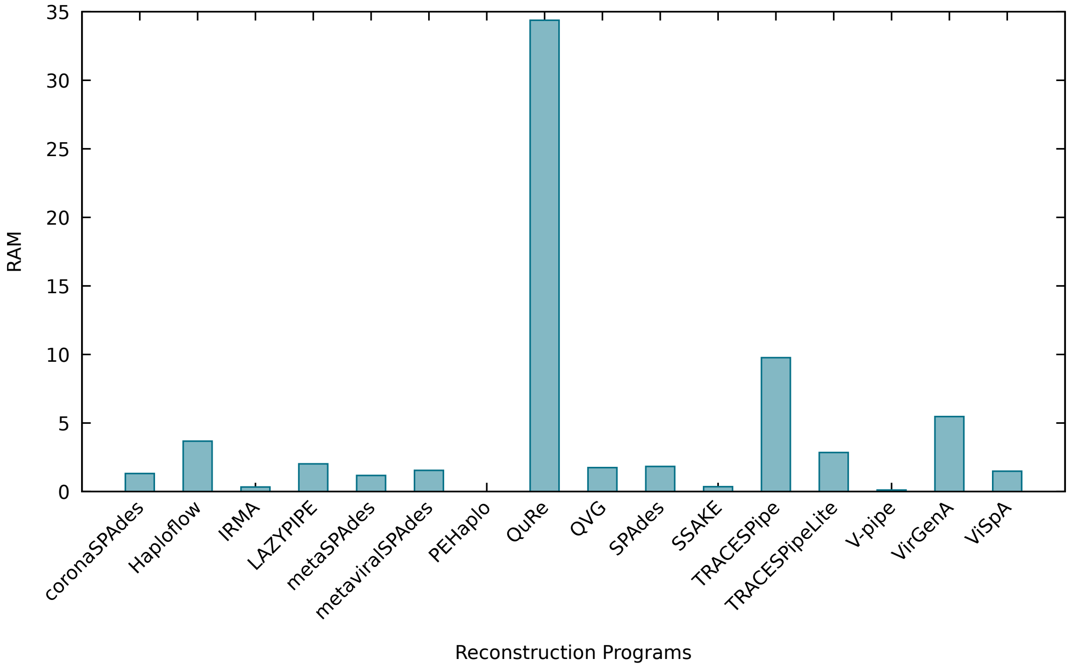

Figure S156: Figure comparing the average performance of the reconstruction programs in terms of the RAM used across datasets SRR23101281, SRR23101235, SRR23101259, SRR23101276, SRR23101228 and SRR12175231.

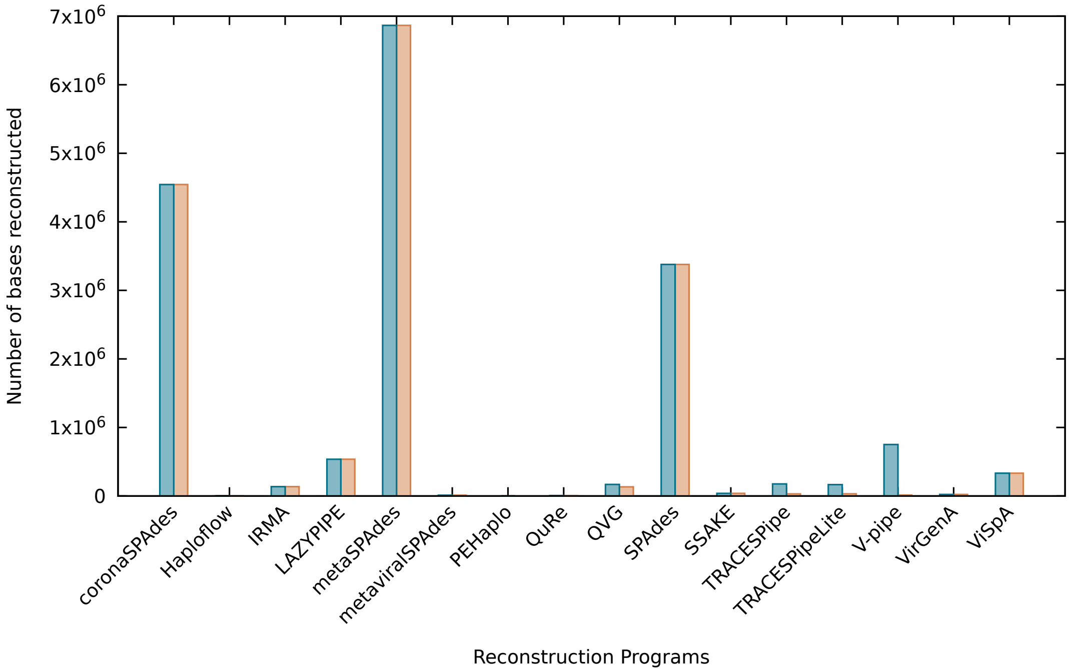

Figure S157: Figure comparing the average performance of the reconstruction programs in terms of the overall number of bases reconstructed across datasets SRR23101281, SRR23101235, SRR23101259, SRR23101276, SRR23101228 and SRR12175231. The blue bars show the performance using all bases present in the reconstructed file, whereas the orange bars show the results excluding non-reconstructed bases (N).

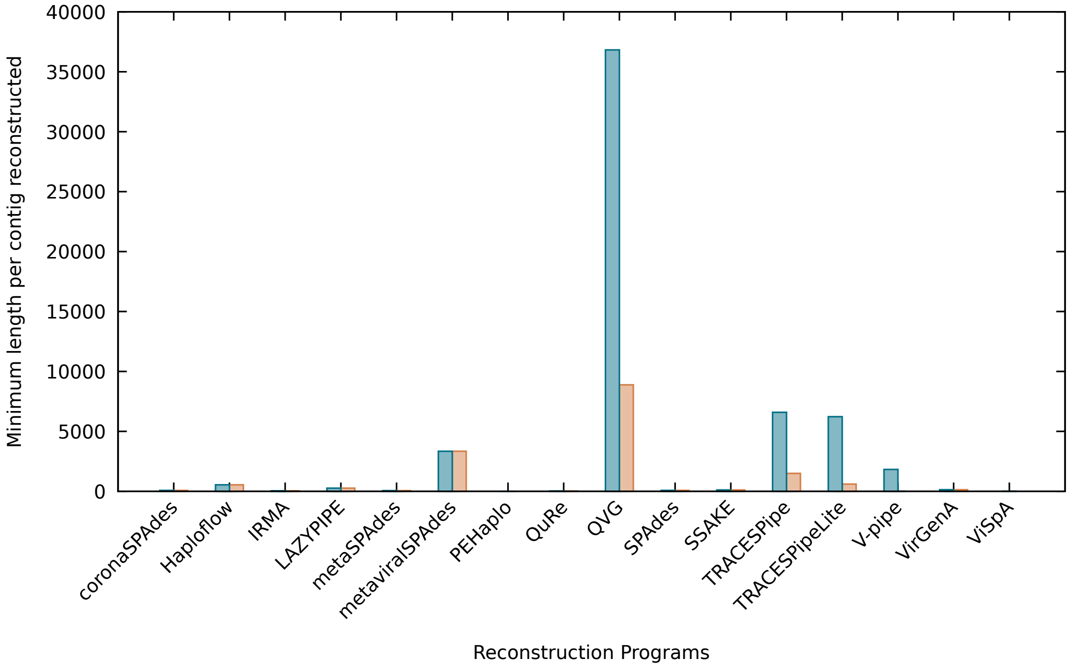

Figure S158: Figure comparing the average performance of the reconstruction programs in terms of the minimum number of bases reconstructed per scaffold across datasets SRR23101281, SRR23101235, SRR23101259, SRR23101276, SRR23101228 and SRR12175231. The blue bars show the performance using all bases present in the reconstructed file, whereas the orange bars show the results excluding non-reconstructed bases (N).

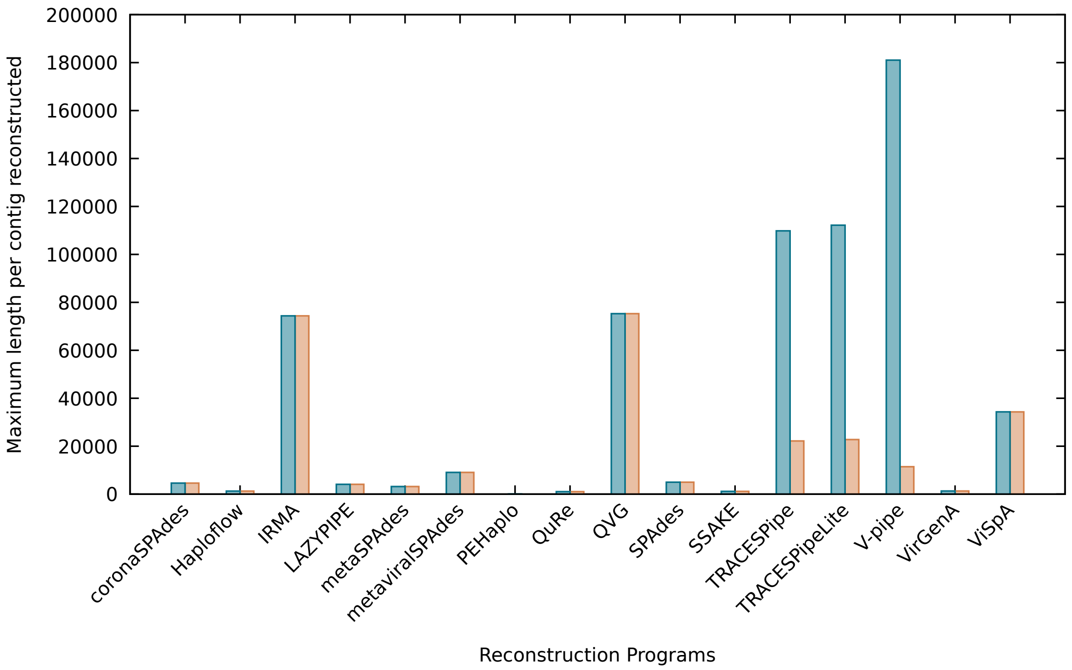

Figure S159: Figure comparing the average performance of the reconstruction programs in terms of the maximum number of bases reconstructed by scaffold across datasets SRR23101281, SRR23101235, SRR23101259, SRR23101276, SRR23101228 and SRR12175231. The blue bars show the performance using all bases present in the reconstructed file, whereas the orange bars show the results excluding non-reconstructed bases (N).

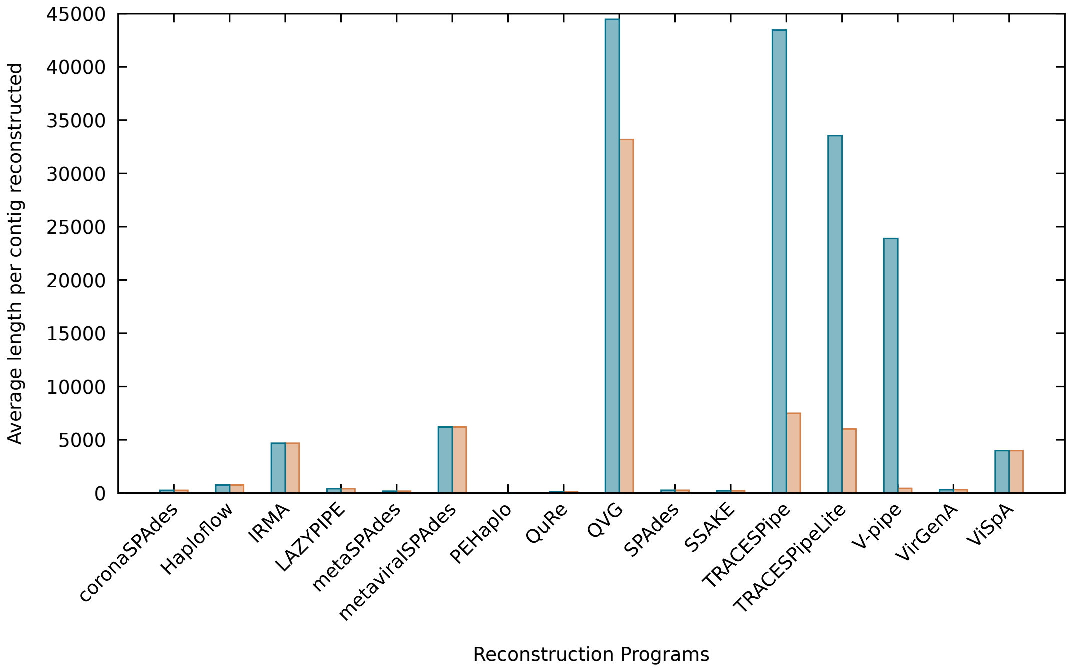

Figure S160: Figure comparing the average performance of the reconstruction programs in terms of the average number of bases reconstructed by scaffold across datasets SRR23101281, SRR23101235, SRR23101259, SRR23101276, SRR23101228 and SRR12175231. The blue bars show the performance using all bases present in the reconstructed file, whereas the orange bars show the results excluding non-reconstructed bases (N).

Table S158: Results obtained for SRR23101281 using the benchmark proposed. The execution time was measured in seconds, the RAM usage was measured in GB and the CPU usage is presented as a percentage. The executions were, when possible, capped at 6 threads and 48 GB of RAM.

Reconstruction tool	Execution time	RAM usage	CPU usage	Number of scaffolds	Reconstructed bases	Minimum scaffold length	Maximum scaffold length	Average scaffold length	Reconstructed bases (excluding N)	Minimum scaffold length (excluding N)	Maximum scaffold length (excluding N)	Average scaffold length (excluding N)
coronaSPAdes	8.74	0.191	471.5	1188.0	323936.0	74.0	2171.0	272.7	323936.0	74.0	2171.0	272.7
Haploflow	9.705	0.456	99.0	1.0	564.0	564.0	564.0	564.0	564.0	564.0	564.0	564.0
IRMA	379.445	0.318	552.5	28.0	126512.0	44.5	74366.0	4707.2	126510.5	44.5	74365.0	4707.15
LAZYPIPE	8.175	1.792	316.5	111.0	55765.0	303.0	2065.0	502.4	55765.0	303.0	2065.0	502.4
metaSPAdes	22.625	0.281	444.0	2059.0	415696.0	56.0	2111.0	201.9	415696.0	56.0	2111.0	201.9
metaviralSPAdes	–	–	–	–	–	–	–	–	–	–	–	–
PEHaplo	–	–	–	–	–	–	–	–	–	–	–	–
QuRe	8145.92	19.898	693.0	31.0	4528.0	54.5	641.0	164.5	4528.0	54.5	641.0	164.5
QVG	123.54	1.489	151.0	3.0	498530.0	155156.0	171687.0	166176.7	363660.0	36817.0	171687.0	121220.0
SPAdes	27.73	0.327	429.0	905.0	248396.0	78.0	2171.0	274.5	248396.0	78.0	2171.0	274.5
SSAKE	22.655	0.102	100.0	165.0	37364.5	113.0	748.0	226.55	37364.5	113.0	748.0	226.55
TRACESPipe	170.025	9.768	369.5	5.0	342114.0	4937.0	171704.0	68422.8	115285.0	337.0	94675.0	23057.0
TRACESPipeLite	44.55	2.847	325.5	7.0	361404.0	3000.0	171694.0	51629.1	104462.0	242.0	82985.0	14923.15
VirGenA	17223.62	5.47	783.0	71.5	22776.5	136.0	1308.0	318.5	22776.5	136.0	1308.0	318.5
ViSpA	146.355	4.966	92.5	101.0	1820439.0	0.0	56546.0	18024.1	1818763.0	0.0	56485.0	18007.6
V-pipe	368.615	0.112	117.0	36.0	974599.0	1679.0	171685.0	27072.2	46033.0	0.0	40509.0	1278.7

Table S159: Results obtained for SRR23101235 using the benchmark proposed. The execution time was measured in seconds, the RAM usage was measured in GB and the CPU usage is presented as a percentage. The executions were, when possible, capped at 6 threads and 48 GB of RAM.

Reconstruction tool	Execution time	RAM usage	CPU usage	Number of scaffolds	Reconstructed bases	Minimum scaffold length	Maximum scaffold length	Average scaffold length	Reconstructed bases (excluding N)	Minimum scaffold length (excluding N)	Maximum scaffold length (excluding N)	Average scaffold length (excluding N)
coronaSPAdes	204.76	1.959	491.5	16794.0	3955512.0	74.0	1302.0	235.5	3955512.0	74.0	1302.0	235.5
Haploflow	–	–	–	–	–	–	–	–	–	–	–	–
IRMA	1802.825	0.389	690.5	28.0	131421.5	44.5	74364.5	4892.5	131420.0	44.5	74363.5	4892.45
LAZYPIPE	47.925	2.434	432.0	167.0	63442.0	272.0	1219.0	379.9	63442.0	272.0	1219.0	379.9
metaSPAdes	559.805	1.353	484.5	82410.0	8856513.0	56.0	799.0	107.5	8856513.0	56.0	799.0	107.5
metaviralSPAdes	–	–	–	–	–	–	–	–	–	–	–	–
PEHaplo	–	–	–	–	–	–	–	–	–	–	–	–
QuRe	49325.69	49.125	695.0	80.5	4810.5	13.0	177.0	62.95	4810.5	13.0	177.0	62.95
QVG	448.425	2.406	250.0	4.0	40887.0	5392.0	16569.0	10221.8	31538.0	114.0	16569.0	7884.5
SPAdes	675.875	1.845	496.0	6270.0	1572536.0	78.0	3386.0	250.8	1572436.0	78.0	3386.0	250.8
SSAKE	352.905	0.444	99.0	199.5	34077.0	104.5	314.5	170.8	34077.0	104.5	314.5	170.8
TRACESPipe	258.065	9.768	392.5	5.0	178452.0	4926.0	153083.0	35690.4	11897.0	377.0	7241.0	2379.4
TRACESPipeLite	68.295	2.85	251.5	6.0	194720.0	4926.0	153080.0	32453.3	16974.0	377.0	10064.0	2829.0
VirGenA	–	–	–	–	–	–	–	–	–	–	–	–
ViSpA	254.74	0.17	272.0	32.0	1459.0	0.0	1459.0	45.6	1459.0	0.0	1459.0	45.6
V-pipe	1805.84	0.11	109.5	32.0	953074.0	1684.0	177314.0	29783.6	3301.0	0.0	2343.0	103.2

Table S160: Results obtained for SRR23101259 using the benchmark proposed. The execution time was measured in seconds, the RAM usage was measured in GB and the CPU usage is presented as a percentage. The executions were, when possible, capped at 6 threads and 48 GB of RAM.

Reconstruction tool	Execution time	RAM usage	CPU usage	Number of scaffolds	Reconstructed bases	Minimum scaffold length	Maximum scaffold length	Average scaffold length	Reconstructed bases (excluding N)	Minimum scaffold length (excluding N)	Maximum scaffold length (excluding N)	Average scaffold length (excluding N)
coronaSPAdes	78.37	1.101	483.5	24083.0	6805865.0	74.0	1797.0	282.6	6805865.0	74.0	1797.0	282.6
Haploflow	93.365	3.31	99.5	5.0	2663.0	508.0	588.0	532.6	2663.0	508.0	588.0	532.6
IRMA	1074.31	0.292	681.0	28.0	131803.0	44.5	74366.0	4906.25	131802.0	44.5	74365.0	4906.2
LAZYPIPE	58.99	1.891	470.5	3451.0	1284821.5	272.0	1655.0	372.3	1284821.5	272.0	1655.0	372.3
metaSPAdes	189.725	1.16	469.0	34078.0	8684069.0	56.0	1706.0	254.8	8683669.0	56.0	1706.0	254.8
metaviralSPAdes	–	–	–	–	–	–	–	–	–	–	–	–
PEHaplo	–	–	–	–	–	–	–	–	–	–	–	–
QuRe	19822.305	38.659	694.5	40.0	2739.0	20.0	192.5	74.5	2739.0	20.0	192.5	74.5
QVG	326.36	1.527	308.0	3.0	42610.0	9472.0	16569.0	14203.3	32806.0	6765.0	16569.0	10935.3
SPAdes	260.475	2.138	445.5	22812.0	6582169.0	78.0	2126.0	288.5	6582169.0	78.0	2126.0	288.5
SSAKE	21.545	0.353	96.5	260.0	67590.5	122.0	547.5	260.0	67590.5	122.0	547.5	260.0
TRACESPipe	326.75	9.768	419.0	2.0	15060.0	5588.0	9472.0	7530.0	3369.0	1673.0	1696.0	1684.5
TRACESPipeLite	64.295	2.85	257.0	3.0	31413.0	5363.0	16578.0	10471.0	16343.0	200.0	14983.0	5447.65
VirGenA	–	–	–	–	–	–	–	–	–	–	–	–
ViSpA	226.925	2.518	244.0	30.0	126910.0	0.0	126033.0	4230.3	126895.0	0.0	126018.0	4229.8
V-pipe	1373.24	0.11	111.0	30.0	635696.0	1677.0	177314.0	21189.9	7308.0	0.0	7308.0	243.6

Table S161: Results obtained for SRR23101276 using the benchmark proposed. The execution time was measured in seconds, the RAM usage was measured in GB and the CPU usage is presented as a percentage. The executions were, when possible, capped at 6 threads and 48 GB of RAM.

Reconstruction tool	Execution time	RAM usage	CPU usage	Number of scaffolds	Reconstructed bases	Minimum scaffold length	Maximum scaffold length	Average scaffold length	Reconstructed bases (excluding N)	Minimum scaffold length (excluding N)	Maximum scaffold length (excluding N)	Average scaffold length (excluding N)
coronaSPAdes	74.27	0.92	469.0	4662.0	1049277.0	74.0	1849.0	225.1	1049277.0	74.0	1849.0	225.1
Haploflow	–	–	–	–	–	–	–	–	–	–	–	–
IRMA	1061.895	0.202	661.5	28.0	128546.0	44.5	74366.0	4780.85	128545.0	44.5	74365.0	4780.85
LAZYPIPE	35.055	1.948	436.0	109.0	45900.0	269.0	1407.0	421.1	45900.0	269.0	1407.0	421.1
metaSPAdes	269.395	0.818	472.5	20370.0	2548210.0	56.0	823.0	125.1	2548210.0	56.0	823.0	125.1
metaviralSPAdes	292.695	0.866	477.0	1.0	1435.0	1435.0	1435.0	1435.0	1435.0	1435.0	1435.0	1435.0
PEHaplo	–	–	–	–	–	–	–	–	–	–	–	–
QuRe	26099.33	29.646	705.5	114.5	10380.5	32.0	198.0	90.7	10380.5	32.0	198.0	90.7
QVG	–	–	–	–	–	–	–	–	–	–	–	–
SPAdes	365.18	1.259	478.5	2174.0	474636.0	78.0	1946.0	218.3	474636.0	78.0	1946.0	218.3
SSAKE	90.685	0.262	99.0	118.5	19769.0	108.0	266.0	166.8	19769.0	108.0	266.0	166.8
TRACESPipe	209.23	9.768	430.5	2.0	162552.0	9472.0	153080.0	81276.0	16854.0	2185.0	14669.0	8427.0
TRACESPipeLite	44.895	2.849	317.5	3.0	179122.0	9472.0	153081.0	59707.3	12420.0	1049.0	7985.0	4140.0
VirGenA	–	–	–	–	–	–	–	–	–	–	–	–
ViSpA	177.1	0.175	233.5	31.0	3901.0	0.0	3553.0	125.8	3898.0	0.0	3550.0	125.7
V-pipe	1036.955	0.109	111.0	31.0	941992.0	1683.0	171693.0	30386.8	3824.0	0.0	1990.0	123.4

Table S162: Results obtained for SRR23101228 using the benchmark proposed. The execution time was measured in seconds, the RAM usage was measured in GB and the CPU usage is presented as a percentage. The executions were, when possible, capped at 6 threads and 48 GB of RAM.

Reconstruction tool	Execution time	RAM usage	CPU usage	Number of scaffolds	Reconstructed bases	Minimum scaffold length	Maximum scaffold length	Average scaffold length	Reconstructed bases (excluding N)	Minimum scaffold length (excluding N)	Maximum scaffold length (excluding N)	Average scaffold length (excluding N)
coronaSPAdes	212.26	2.317	477.5	34330.0	9029188.0	74.0	16749.0	263.0	9029168.0	74.0	16749.0	263.0
Haploflow	195.975	7.266	99.0	5.0	5910.0	554.0	2669.0	1182.0	5910.0	554.0	2669.0	1182.0
IRMA	2532.53	0.526	704.0	34.0	157990.5	36.0	74366.0	4650.95	157989.5	36.0	74365.0	4650.9
LAZYPIPE	161.8	2.041	521.5	3700.0	1548550.0	200.0	16621.0	418.55	1548550.0	200.0	16621.0	418.55
metaSPAdes	705.85	2.226	485.0	67109.0	13606179.0	56.0	9896.0	202.7	13605629.0	56.0	9896.0	202.7
metaviralSPAdes	650.015	2.226	475.5	2.0	21945.0	5248.0	16697.0	10972.5	21945.0	5248.0	16697.0	10972.5
PEHaplo	–	–	–	–	–	–	–	–	–	–	–	–
QuRe	56292.89	41.919	686.5	71.5	15601.5	26.0	4964.0	218.25	15601.5	26.0	4964.0	218.25
QVG	542.285	1.63	340.5	11.0	244664.0	4628.0	162114.0	22242.2	230785.0	397.0	162114.0	20980.45
SPAdes	725.575	3.188	487.0	29991.0	8279425.0	78.0	16647.0	276.1	8279325.0	78.0	16647.0	276.1
SSAKE	50.395	0.591	98.0	184.0	50034.5	110.0	4693.5	271.15	50034.5	110.0	4693.5	271.15
TRACESPipe	825.77	9.768	398.5	6.0	349944.0	5142.0	162115.0	58324.0	36105.0	1038.0	11452.0	6017.5
TRACESPipeLite	109.39	2.85	202.5	6.0	204008.5	5121.0	162115.0	34001.45	35548.5	62.0	16565.0	5924.75
VirGenA	–	–	–	–	–	–	–	–	–	–	–	–
ViSpA	356.615	0.905	294.5	30.0	44751.0	0.0	17921.0	1491.7	44738.0	0.0	17920.0	1491.3
V-pipe	2351.975	0.124	109.5	30.0	709217.0	2538.0	235154.0	23640.6	26192.0	0.0	16158.0	873.1

Table S163: Results obtained for SRR12175231 using the benchmark proposed. The execution time was measured in seconds, the RAM usage was measured in GB and the CPU usage is presented as a percentage. The executions were, when possible, capped at 6 threads and 48 GB of RAM.

Reconstruction tool	Execution time	RAM usage	CPU usage	Number of scaffolds	Reconstructed bases	Minimum scaffold length	Maximum scaffold length	Average scaffold length	Reconstructed bases (excluding N)	Minimum scaffold length (excluding N)	Maximum scaffold length (excluding N)	Average scaffold length (excluding N)
coronaSPAdes	118.965	1.407	482.5	24345.0	6100374.0	76.0	3747.0	250.6	6100374.0	76.0	3747.0	250.6
Haploflow	–	–	–	–	–	–	–	–	–	–	–	–
IRMA	754.165	0.263	686.5	35.0	144368.0	36.0	74366.0	4124.8	144366.0	36.0	74365.0	4124.7
LAZYPIPE	46.445	2.021	459.0	556.0	219356.0	271.0	1579.0	394.5	219356.0	271.0	1579.0	394.5
metaSPAdes	489.01	1.208	437.5	42325.0	7083013.0	56.0	3724.0	167.3	7082803.0	56.0	3724.0	167.3
metaviralSPAdes	–	–	–	–	–	–	–	–	–	–	–	–
PEHaplo	–	–	–	–	–	–	–	–	–	–	–	–
QuRe	14239.35	27.03	699.0	29.0	1702.5	29.5	104.5	63.25	1702.5	29.5	104.5	63.25
QVG	253.02	1.685	263.0	2.0	18944.0	9472.0	9472.0	9472.0	9785.0	313.0	9472.0	4892.5
SPAdes	476.85	2.237	499.0	11734.0	3112349.0	78.0	3646.0	265.2	3112349.0	78.0	3646.0	265.2
SSAKE	185.6	0.376	99.0	113.5	22480.0	124.5	372.0	198.05	22480.0	124.5	372.0	198.05
TRACESPipe	235.165	9.768	446.5	1.0	9472.0	9472.0	9472.0	9472.0	3363.0	3363.0	3363.0	3363.0
TRACESPipeLite	45.58	2.85	315.5	2.0	26042.0	9473.0	16569.0	13021.0	5703.0	1686.0	4017.0	2851.5
VirGenA	–	–	–	–	–	–	–	–	–	–	–	–
ViSpA	171.445	0.176	246.5	26.0	584.0	0.0	379.0	22.5	584.0	0.0	379.0	22.5
V-pipe	1036.14	0.106	111.0	26.0	293390.0	1684.0	153080.0	11284.2	486.0	0.0	325.0	18.7

3 Software and Hardware recommendations

The benchmark was executed on a computer running Linux Ubuntu 22.04.4 LTS with an Intel® Core™ i7-6700 CPU @ 3.40GHz × 8 processor and with 64 GB of RAM and the computational resources were limited, when possible, to 48 GB of RAM and 6 CPU threads. For each program, the execution time was capped at six hours, either overall or per reference genome provided. If the computer running the benchmark has different specifications, it is recommended that the options --threads and --memory are set accordingly when executing "Reconstruction.sh".

4 Reproducibility

4.1 Installation

4.1.1 Downloading the benchmark

To download the benchmark files, Git must be installed. Git can be installed using:

```
sudo apt install git -y
```

To retrieve and give permissions to all the files necessary to run the benchmark, the following commands should be used:

```
git clone https://github.com/viromelab/HVRS.git
cd HVRS/src/
chmod +x *.sh
```

4.1.2 Miniconda installation

To install and execute the benchmark, Miniconda [8] should be installed. If Miniconda is not installed, please execute the following command:

```
./Installation.sh --miniconda
```

Accept the terms and conditions and finish the installation of Miniconda. After the installation, the execution of the program pauses and the message "Please close and reopen this terminal" will appear. When that happens, press any key, close and reopen the terminal so the changes are applied.
If Miniconda was not initialized, please use the following command:

```
conda init
```

4.1.3 Changing paths

The options "PYTHON2_PATH" and "CONDA_PREFIX" present in the file "Installation.sh" should be in accordance with the paths where they are installed on the computer. If they are not in accordance, they should be altered to ensure the proper execution of the benchmark.

After making the changes, if necessary, return to the path to which the benchmark was downloaded using the command:

```
cd HVRS/src/
```

4.1.4 Reconstruction tools installation

To install all of the reconstruction tools used in the benchmark, as well as the tools used in the simulation, classification and evaluation processes, the following step will need to be performed:

```
./Installation.sh --all
```

There are options available to install only some of the programs included in the benchmark, however, it is recommended that all programs be installed. It is also recommended that after the installation process, the terminal is closed and opened again.

Along the installation process, the execution may pause and a prompt may appear on the screen. Please follow the instructions given by the prompt and press any key to resume the installation process.

4.1.5 Installation options

> **-h, --help**
> Displays the help menu and exits the program.

> **--miniconda**
> Installs Miniconda.

> **--all**
> Installs all reconstruction programs, as well as the dataset-generating, classification and evaluation tools.

> **--coronaspades**
> Installs coronaSPAdes [9].

> **--haploflow**
> Installs Haploflow [10].

> **--irma**
> Installs IRMA [11].

> **--lazypipe**
> Installs LAZYPIPE (version 2) [12].

> **--metaspades**
> Installs metaSPAdes [13].

> **--metaviralspades**
> Installs metaviralSPAdes [14].

> **--pehaplo**
> Installs PEHaplo [15].

> **--qure**
> Installs QuRe [16].

> **--qvg**
> Installs QVG [17].

> **--spades**
> Installs SPAdes [18].

> **--ssake**
> Installs SSAKE [19].

> **--tracespipe**
> Installs TRACESPipe [20].

> **--tracespipelite**
> Installs TRACESPipeLite [20].

> **--virgena**
> Installs VirGenA [21].

> **--vispa**
> Installs ViSpA [22].

> **--vpipe**
> Installs V-pipe [23].

> **--tools**
> Installs other tools used in the benchmark, including the dataset-generating, classification and evaluation tools.

4.2 Simulation of the datasets

To generate and retrieve the default datasets, the following command should be used:

```
./Simulation.sh
```

In this file, each of the datasets can be changed. If datasets are added or removed, those changes should be reflected in the files "Reconstruction.sh" and "Evaluation.sh", to ensure the results are correct.

4.3 Verification

To verify whether or not the tools were correctly installed, the datasets were successfully generated and the reference viruses were extracted, the following command can be executed.

```
./Verification.sh --all -c
```

4.3.1 Verification options

> **-h, --help**
> Displays the help menu and exits the program.

> **-t, --tools**
> Check if all of the reconstruction programs are installed.

> **-d, --datasets**
> Check if all of the datasets and reference genomes were successfully generated.

> **--all**
> Check if the tools were successfully installed and if the datasets and reference genomes were generated.

> **--coronaspades**
> Checks if coronaSPAdes [9] is installed.

> **--haploflow**
> Checks if Haploflow [10] is installed.

> **--irma**
> Checks if IRMA [11] is installed.

> **--lazypipe**
> Checks if LAZYPIPE (version 2) [12] is installed.

> **--metaspades**
> Checks if metaSPAdes [13] is installed.

> **--metaviralspades**
> Checks if metaviralSPAdes [14] is installed.

> **--pehaplo**
> Checks if PEHaplo [15] is installed.

> **--qure**
> Checks if QuRe [16] is installed.

> **--qvg**
> Checks if QVG [17] is installed.

> **--spades**
> Checks if SPAdes [18] is installed.

> **--ssake**
> Checks if SSAKE [19] is installed.

> **--tracespipe**
> Checks if TRACESPipe [20] is installed.

> **--tracespipelite**
> Checks if TRACESPipeLite [20] is installed.

> **--virgena**
> Checks if VirGenA [21] is installed.

> **--vispa**
> Checks if ViSpA [22]is installed.

> **--vpipe**
> Checks if V-pipe [23] is installed.

> **--other**
Checks if the programs necessary to generate and classify the datasets and evaluate the results are installed.

> **-c, --careful**
Checks if the reconstruction programs execute correctly.

4.4 Reconstruction

To reconstruct the datasets generated in the simulation step, the following command should be used:

```
./Reconstruction.sh --all --threads THREADS --memory MEMORY --virgena-timeout VIRGENA_TIMEOUT
```

If you wish to modify the datasets and/or reference genomes considered by default, the file "Reconstruction.sh" should be modified accordingly. There are also options available if only some of the programs available on the benchmark should be executed.

The values of "THREADS" and "MEMORY" should be altered to suit the computer's specifications. Additionally, there is a timeout implemented for the execution of the tool VirGenA [21], where the processing time for each reference considered is capped to the value of "VIRGENA_TIMEOUT", which is set by default to 15 minutes. Depending on the computer running the benchmark and on the dataset considered, this value may have to be altered to ensure that the timeout does not occur too soon, preventing the tool from reconstructing the datasets. Additionally, there is a timeout for all tools, set by default at six (6) hours per dataset (for tools that do not require a reference to be given) and six (6) hours per reference (for tools that require a reference to be given). This parameter can be altered as well to fit specifications.

Note: The installation of the reconstruction programs is verified at the beginning of the execution process and tools that are considered to not be installed will be skipped.

If the reference genomes are unknown, the following command can be executed:

```
./Reconstruction.sh --all --threads THREADS --memory MEMORY --virgena-timeout VIRGENA_TIMEOUT --
    reads READS_NAME --top_falcon TOP_FALCON
```

The "READS_NAME" must be substituted by the name of the files to be reconstructed before the _1.fq and _2.fq. For example, "READS_NAME" should be substituted by "dataset" if the files containing the reads are named "dataset_1.fq" and "dataset_2.fq".

The "TOP_FALCON" parameter should be changed accordingly depending on the number of references that must be retrieved by FALCON-meta [7]. It should be noted that this number does not correspond to the actual number of references to be used but to the value of top of similarity considered by FALCON-meta which should be overestimated.

4.4.1 Reconstruction options

> **-h, --help**
Displays the help menu and exits the program.

> **--all**
Reconstructs the genomes using all of the reconstruction programs available.

> **--coronaspades**
Reconstructs the genomes using coronaSPAdes [9].

> **--haploflow**
Reconstructs the genomes using Haploflow [10].

> **--irma**
Reconstructs the genomes using IRMA [11].

> **--lazypipe**
Reconstructs the genomes using LAZYPIPE (version 2) [12].

> **--metaspades**
Reconstructs the genomes using metaSPAdes [13].

> **--metaviralspades**
Reconstructs the genomes using metaviralSPAdes [14].

> **--pehaplo**
Reconstructs the genomes using PEHaplo [15].

> **--qure**
Reconstructs the genomes using QuRe [16].

> **--qvg**
Reconstructs the genomes using QVG [17].

> **--spades**
Reconstructs the genomes using SPAdes [18].

> **--ssake**
Reconstructs the genomes using SSAKE [19].

> **--tracespipe**
Reconstructs the genomes using TRACESPipe [20].

> **--tracespipelite**
Reconstructs the genomes using TRACESPipeLite [20].

> **--virgena**
Reconstructs the genomes using VirGenA [21].

> **--vispa**
Reconstructs the genomes using ViSpA [22].

> **--vpipe**
Reconstructs the genomes using V-pipe [23].

> **-t, --threads**
Sets the maximum amount of threads used (when possible).

> **-m, --memory**
Sets the maximum amount of RAM used (when possible).

> **--virgena-timeout**
Sets the maximum time used by VirGenA [21] to reconstruct a genome for each reference.

> **--timeout**
Sets the maximum time used by all reconstruction programs. If the reconstruction program requires a reference, the maximum time corresponds to the maximum time to reconstruct a genome for each reference. If this value is larger than the virgena-timeout, the virgena-timeout is overridden.

> **-r, --reads**
Name of the files containing the FASTQ reads. The string must be the name before _1 and _2.fq. The references are retrieved using FALCON-meta [7].

> **-y, --yes**
Assumes the answer to all prompts is yes. This may lead to loss of data, specifically regarding the reconstructed directory and the files containing viral references.

> **--top_falcon**
Sets the top of similarity value for FALCON-meta [7].

4.5 Evaluation

To evaluate the reconstruction made by each of the tools, the following command should be used.

```
./Evaluation.sh
```

The evaluation process is made using dnadiff from the MUMmer4 package [6], GeCo3 [5] and SeqKit [24]. The results of the evaluation process are available as 2 files: total_stats.tsv and total_stats.tex. Additionally, results regarding the execution of dnadiff are stored in 2 files: dnadiff_stats.tsv and dnadiff_stats.tsv. These 4 files contain information about the performance of each of the tools tested and are present in the directory "reconstructed". If the default datasets were modified, the "Evaluation.sh" file should be changed accordingly.

4.6 Plots

To generate plots, the following command should be used:

```
./Plots.sh
```

All of the plots generated are available in the directory "Graphs". If tools or datasets were added to the benchmark, the files "Plots.sh" and "Plots2.sh" should be altered accordingly.

4.7 Cleaning

To erase all of the files generated for the datasets, the following command should be used:

```
./Cleaning.sh
```

4.8 Run all steps

To run all of the steps of the benchmark, after downloading the benchmark following the instructions described in Section 4.1.1 of the supplement, please change the variables "PYTHON2_PATH" and "CONDA_PREFIX" to the paths where Python 2 and Miniconda are installed, on the file "Installation.sh", if necessary. Additionally, the values of the options --threads and --memory should be changed to fit the specifications of the computer running the benchmark. Afterwards, the following command should be used:

```
./Run_benchmark.sh
```

References

[1] Weichun Huang, Leping Li, Jason R. Myers, and Gabor T. Marth. ART: a next-generation sequencing read simulator. *Bioinformatics*, 28(4):593–594, 2012.

[2] João R. Almeida, Armando J. Pinho, José L. Oliveira, Olga Fajarda, and Diogo Pratas. GTO: a toolkit to unify pipelines in genomic and proteomic research. *SoftwareX*, 12:100535, 2020.

[3] Jorge M. Silva, Weihong Qi, Armando J. Pinho, and Diogo Pratas. AlcoR: alignment-free simulation, mapping, and visualization of low-complexity regions in biological data. *bioRxiv*, pages 2023–04, 2023.

[4] Heng Li. wgsim, 2011. Accessed: 2025-10-10.

[5] Milton Silva, Diogo Pratas, and Armando J. Pinho. Efficient DNA sequence compression with neural networks. *GigaScience*, 9(11):giaa119, 2020.

[6] Stefan Kurtz, Adam Phillippy, Arthur L. Delcher, Michael Smoot, Martin Shumway, Corina Antonescu, and Steven L. Salzberg. Versatile and open software for comparing large genomes. *Genome biology*, 5(2):1–9, 2004.

[7] Diogo Pratas, Morteza Hosseini, Gonçalo Grilo, Armando J. Pinho, Raquel M. Silva, Tânia Caetano, João Carneiro, and Filipe Pereira. Metagenomic composition analysis of an ancient sequenced polar bear jawbone from Svalbard. *Genes*, 9(9):445, 2018.

[8] Anaconda, Inc. Miniconda, 2013.

[9] Dmitry Meleshko, Iman Hajirasouliha, and Anton Korobeynikov. coronaSPAdes: from biosynthetic gene clusters to RNA viral assemblies. *Bioinformatics*, 38(1):1–8, 2021.

[10] Adrian Fritz, Andreas Bremges, Zhi-Luo Deng, Till R. Lesker, Jasper Götting, Tina Ganzenmueller, Alexander Sczyrba, Alexander Dilthey, Frank Klawonn, and Alice C. McHardy. Haploflow: strain-resolved de novo assembly of viral genomes. *Genome Biology*, 22:1–19, 2021.

[11] Samuel S Shepard, Sarah Meno, Justin Bahl, Malania M Wilson, John Barnes, and Elizabeth Neuhaus. Viral deep sequencing needs an adaptive approach: IRMA, the iterative refinement meta-assembler. *BMC genomics*, 17:1–18, 2016.

[12] Ilya Plyusnin, Olli Vapalahti, Tarja Sironen, Ravi Kant, and Teemu Smura. Enhanced Viral Metagenomics with Lazypipe 2. *Viruses*, 15(2):431, 2023.

[13] Sergey Nurk, Dmitry Meleshko, Anton Korobeynikov, and Pavel A. Pevzner. metaSPAdes: a new versatile metagenomic assembler. *Genome research*, 27(5):824–834, 2017.

[14] Dmitry Antipov, Mikhail Raiko, Alla Lapidus, and Pavel A. Pevzner. Metaviral SPAdes: assembly of viruses from metagenomic data. *Bioinformatics*, 36(14):4126–4129, 2020.

[15] Jiao Chen, Yingchao Zhao, and Yanni Sun. De novo haplotype reconstruction in viral quasispecies using paired-end read guided path finding. *Bioinformatics*, 34(17):2927–2935, 2018.

[16] Mattia C. F. Prosperi and Marco Salemi. QuRe: software for viral quasispecies reconstruction from next-generation sequencing data. *Bioinformatics*, 28(1):132–133, 2012.

[17] Alex Váradi, Eszter Kaszab, Gábor Kardos, Eszter Prépost, Krisztina Szarka, and Levente Laczkó. Rapid genotyping of targeted viral samples using Illumina short-read sequencing data. *Plos one*, 17(9):e0274414, 2022.

[18] Andrey Prjibelski, Dmitry Antipov, Dmitry Meleshko, Alla Lapidus, and Anton Korobeynikov. Using SPAdes de novo assembler. *Current protocols in bioinformatics*, 70(1):e102, 2020.

[19] René L. Warren, Granger G. Sutton, Steven J. M. Jones, and Robert A. Holt. Assembling millions of short DNA sequences using SSAKE. *Bioinformatics*, 23(4):500–501, 2007.

[20] Diogo Pratas, Mari Toppinen, Lari Pyöriä, Klaus Hedman, Antti Sajantila, and Maria F. Perdomo. A hybrid pipeline for reconstruction and analysis of viral genomes at multi-organ level. *GigaScience*, 9(8):giaa086, 2020.

[21] Gennady G. Fedonin, Yury S. Fantin, Alexnader V. Favorov, German A. Shipulin, and Alexey D. Neverov. VirGenA: a reference-based assembler for variable viral genomes. *Briefings in bioinformatics*, 20(1):15–25, 2019.

[22] Irina Astrovskaya, Bassam Tork, Serghei Mangul, Kelly Westbrooks, Ion Măndoiu, Peter Balfe, and Alex Zelikovsky. Inferring viral quasispecies spectra from 454 pyrosequencing reads. In *BMC bioinformatics*, volume 12, pages 1–10. BioMed Central, 2011.

[23] Susana Posada-Céspedes, David Seifert, Ivan Topolsky, Kim P. Jablonski, Karin J. Metzner, and Niko Beerenwinkel. V-pipe: a computational pipeline for assessing viral genetic diversity from high-throughput data. *Bioinformatics*, 37(12):1673–1680, 2021.

[24] Wei Shen, Shuai Le, Yan Li, and Fuquan Hu. SeqKit: a cross-platform and ultrafast toolkit for FASTA/Q file manipulation. *PloS one*, 11(10):e0163962, 2016.
