# Supplementary material for: An evaluation of computational methods for reconstruction of human viral DNA genomes
Source: Gigascience. 2025 Dec 26;15:giaf159. doi: 10.1093/gigascience/giaf159 (PMC12927430; doi:10.1093/gigascience/giaf159)
Supplement: giaf159_Authors_Response_To_Reviewer_Comments_original_submission [file giaf159_authors_response_to_reviewer_comments_original_submission.pdf]

Author's Response To Reviewer Comments

Dear Editor,
We greatly appreciate the opportunity given to revise our manuscript. We would like to thank the Reviewers, whose suggestions allowed us to improve our manuscript in many ways. We have now addressed the points raised by them, as outlined in blue in this revision letter. Following their suggestions, appropriate changes have been introduced to the manuscript, as shown also in blue, including the addition of further information regarding the viral genomes considered/found when testing the benchmark. We hope we have been able to address their concerns and that our manuscript is now suitable for publication at GigaScience.

Reviewer 1
Reviewer: The authors describe that multiple strains may be present within a single infection. Indeed, the variability of strains within a single infection may be particularly important for some viruses. QuRe, ViSpA, SAVAGE and ViQUF are explicitly designed to find quasispecies. Are there any other tools in the benchmark that can predict whether samples are heterogeneous (or whose results can be used to infer this)?

Authors: In the article, we have pointed out that a tool reconstructed the quasispecies only when this was explicitly indicated by the authors in the original articles describing the tool. From the tools considered in this study, only QuRe, SAVAGE, viaDBG, VIQUF and ViSpA are reported to able to reconstruct the quasispecies. As for the ability to assess sample heterogeneity, while some tools may produce results that could indirectly suggest heterogeneity (e.g., through assembly fragmentation or coverage variation), only the aforementioned tools are explicitly designed for quasispecies reconstruction. Additionally, we have previously investigated intra-host variability of viral genomes across different human organs, which can provide further context on sample heterogeneity and strain diversity [2].

Reviewer: The authors have used the human mitochondrion as a source of contamination to test whether the tools are sensitive to it. Is there a reason why only the mitochondrion was used for this test and other, perhaps random, human DNA fragments were not?

Authors: In the article, random DNA fragments, referred to as contamination, were added to most synthetic samples in addition to the human mitochondrial DNA. We used mtDNA because it can usually be found in these samples, as reported in our previous work [2, 3]. The non-viral inclusions of each dataset and the characteristics of the simulated sequencing process can be found in the Supplementary table 1.

Reviewer: The error rate can strongly influence the accuracy of reference-based genome reconstructions. Has the effect of error rate been tested or could it affect the results, e.g. are there any tools in the benchmark that are less sensitive to higher error rates?

Authors: We appreciate the reviewer's comment. It is not entirely clear whether the term "error rates" refers to sequencing errors, mutation rates, or alignment inaccuracies. However, in our benchmark, we assessed the performance of the reconstruction tools using synthetic datasets in which the viral genomes were systematically mutated to include between 0% and 15% single nucleotide polymorphisms (SNPs). This setup allowed us to evaluate how the tools handle increasing levels of sequence divergence from the reference. While this does not directly simulate sequencing errors, it does provide insight into each tool's robustness to genetic variability, which can resemble the impact of elevated error rates in practical scenarios. A more detailed study focused specifically on sequencing error sensitivity could be an interesting direction for future work.

Reviewer: In the synthetic dataset, the coverage ranged from 2-40×. This range represents scenarios where the viral copy number is low, but especially if the viral DNA was enriched before sequencing, the coverage could be much higher. Is there a reason to specifically choose 40x coverage as the highest coverage value? I agree that low coverage is a difficult challenge, but checking the performance of different tools at high read depth can help readers to choose the right tool for these use cases if there is a difference in the performance of the tools at e.g. >100x coverage.

Authors: We thank the reviewer for this valuable observation. In our study, we selected coverage values ranging from 2x to 40x to represent low to moderate viral load scenarios, which are particularly challenging for reference-based reconstruction. Higher coverage datasets were not included due to practical constraints—reconstructing genomes at higher depths required significantly more computational time and memory. However, the benchmark framework we developed is fully extensible and customizable. Users interested in evaluating tool performance at higher coverages (e.g., >100x), or under different conditions, can easily generate and include their own datasets to suit specific research questions or practical scenarios. We believe this flexibility is a strength of the benchmark and encourages broader community use and adaptation.

Reviewer: The authors correctly describe that the complexity of genomes can be a challenge for accurate genome reconstruction. Assessing the complexity (e.g. repetitive content ratio, GC ratio) of the genomes used in the synthetic dataset can add additional value to the results by showing how different tools perform on genomes of different complexity.

Authors: We thank the reviewer for the suggestion and we have added the Supplementary Tables 3 and 135 to 140 containing the GC content and compression ratio of each virus in (or estimated to be in) the datasets.

Reviewer: Some reference-based tools (QVG, TRACESPipe, TRACESPipeLite and V-pipe) produced results with many gaps. Could the different approach be a reason for how they deal with low coverage regions? QVG, for example, masks positions with low sequencing depth to increase the specificity of the search for polymorphisms. Can the gaps be explained by the variation in sequencing depth, i.e. could the gaps be linked to genomic regions with very low or very high sequencing depth?

Authors: We believe the presence of gaps in the reconstructed genomes can be attributed to several factors, in particular the reconstruction methodology and the specific methodologies used by the tools evaluated. Reference-free tools tend to produce highly fragmented scaffolds with few gaps, while the tools mentioned by the Reviewer often generate fewer, but longer scaffolds. Although there are some variations, the tendency of these tools to reconstruct viral genomes into a single scaffold, combined with the fact that they rely on reference sequences which provide an estimate of expected genome length, likely contributes to the higher number of gaps observed. In addition to this factor, the specific methodology of the tools may also be a contributing factor to the existence of gaps in the reconstructed files.

Reviewer: I agree that benchmarking real datasets without the correct original sequence is a difficult task. I believe that showing the coverage and completeness (e.g. the ratio of the reconstructed length of the reference genome) can be an additional and useful information for the reader to choose the right tool for different tasks. The expected length of the viral genomes could be determined by the length of the reference genomes used, based on the classification of FALCON-meta, and in the case of de novo pipelines, the scaffolds that do not match the references could be classified using e.g. kraken2. This could show how complete the reconstructed genomes are and whether there are other viral genomes in the samples that FALCON-meta missed but still represent valuable information. Supplementary Figures S143-S146 show the number of reconstructed bases with and without gaps, but I think that this experiment should be emphasised more in the main text and that the ratio of reconstructed bases to the expected genome sizes might be more informative than just the total number of reconstructed base pairs.

Authors: We appreciate the reviewer's suggestion. However, the main manuscript and Supplementary Material are quite extensive, and we have already made selections regarding the data shown and described. Additionally, any findings using this metric would still be only indicative, as the true composition of the dataset would continue to be unknown. In the future, we plan to test the performance of a tool developed following more laboratorial methods in which the samples are synthesized and sequenced, making their characteristics and composition known.

Reviewer 2
Reviewer: To our surprise, the authors (who are also authors of two tools under evaluation) somehow do

not distinguish different kinds of input data that is pretty important as it effectively determines the choice of the tool. Clearly, there is no silver bullet here and there is no single push-button solution that could handle universally all kinds of input data. It is very strange that e.g. authors do not distinguish between DNA and RNA viruses. The sequencing approaches for these kinds of data is very different, they have entirely different internal structure and organization and the challenges associated with assembly process are different. This is summarized well in e.g. (Grabherr et al, 2011), (Bushmanova et al, 2019) and (Meleshko et al, 2022) among the others. To add second dimension here: we can have more or less "pure" viral culture, or metagenome / metavirome, or some highly divergent metavirome (e.g. in case of HIV or other viruses undergo reverse transcription). The host contamination is more sound for DNA viruses, etc. So, to summarize - all (very complex!) variations of input data were somehow folded into a single "human viruses" title, which is really misleading. It is the properties of input data that should guide the choice of the appropriate tool.

Authors: In this article we have specifically focused on assessing the performance of genome reconstruction tools when reconstructing sequences belonging to DNA viruses. To clarify this focus, we have changed the title, revised the abstract and made corresponding changes in the manuscript.
We agree that the characteristics of the input data should be considered when selecting the appropriate reconstruction tool. For this reason, we evaluated the performance of various tools across different scenarios and provided recommendations based on our findings. Note that the methodology and benchmark are publicly available and designed to be flexible for testing with different input data. We selected these data characteristics because they reflect scenarios we have previously worked on, and our goal was to maintain a configuration comparable to what we found in [2, 3].

Reviewer: Next, the choice of tools is also somewhat questionable. Some well-known tools like PRICE or VICUNA were omitted. Ok, IVA is here and this might be enough for "classical" viral assemblies. But then generic-purpose metagenome assembler metaSPAdes is considered without other choices. How about MEGAHIT? for RNA viral data - what's about Trinity or rnaSPAdes? It was strange to see coronaSPAdes mentioned, while it is essentially rnaviralSPAdes + set of SARS-Cov 2 HMMs. Why not just rnaviralSPAdes if we already know we're not going to reconstruct coronaviral data? Another thing is that the majority of tools are tuned for a particular tasks: there are tools for quasispecies assembly, so they would aim to preserve all the variation present. Metagenomic assemblers aim to provide a backbone consensus of a metagenome. Usually assemblers for RNA data aim for the reconstruction as many transcripts as possible (so their "duplication rate" might be misleading). metaviralSPAdes aims to reconstruct full-length circular and linear viruses from complex contaminated metagenomes, so it could be very conservative, etc. It feels like the benchmarking compares something warm with something soft giving misleading guidance to the reader.

Authors: In the article, we focused on benchmarking tools that were specialised to reconstruct viral genomes, and therefore tended to exclude general-purpose tools.
PRICE was included in this study as the authors report the reconstruction of viral genomes. However it was only partially reproducible, meaning that it was only able to be partially installed or it was only able to perform some of the tasks desired, but did not output results. This may be because of several factors, namely due to version conflicts between dependencies required in the execution process, conflicts with other programs installed, conflicts with the version of the operating system used, or the dependencies being no longer available. VICUNA was not included in the benchmark as it is requires a registration to be downloaded, placing it outside the scope of our research. However, we appreciate you bringing this to our attention and have now added VICUNA to the list of tools that fall outside our study's parameters.
The inclusion of some variations of SPAdes was made so we could compare the performance differences between them and assess which ones would be more suitable in different scenarios. We have tested coronaSPAdes chosen instead of rnaviralSPAdes, as it includes the HMMs which could potentially be adapted to the samples provided.

Reviewer: Finally, it is the year 2025, but the pipeline is just a huge pile of shell scripts that install tools (sometimes outdated as far as I can see, e.g. it uses SPAdes 3.13.0 that was released more than 5 years ago) often globally, sometimes only via conda. It could hardly be named as "reproducible" pipeline: error handling is quite non-existing, if something happens in between the user might end with some partially resolved state. There are lots of frameworks and approaches developed recently that provide all necessary

details like job isolation, installation, restart & checkpointing, data acquisition, etc. To put things simple: why everything is done manually via hand-written shell scripts and not based on say, Nextflow? There are lots of ready modules from ‘nf-core‘ that one could just reuse. Likely some ideas could be taken from https://github.com/nf-core/viralrecon/ and other pipelines available there.

Authors: We thank the Reviewer for the feedback and suggestions and we feel the same hurdle in this complex and timely task of comparing the pipelines. There are two main reasons for using older versions of certain tools. First, the HVRS was under development for over two years, and the versions of the tools considered often reflect what was available at that time. Second, installing the latest versions of all tools often presented challenges due to compatibility issues and version conflicts.
While we made every effort to ensure the benchmark is reproducible, we cannot guarantee that it is compatible with every individual system configuration. To address this, we have provided a virtual machine with the complete benchmark environment pre-installed, available via GigaDB. This way we ensure the reproducibility and allow the users to test the benchmark using different host operating systems.

Reviewer 3
Reviewer: What are the types of data used in the manuscript? Is it solely metagenomics data? If so, it would be beneficial to clarify this in the abstract and potentially in the title

Authors: In this article, we have tested only datasets containing metagenomic data. We have made this focus more explicit in the manuscript.

Reviewer: Was the real data comprised of metagenomics? It would be advantageous to include some text explaining the nature of the data.

Authors: We appreciate the Reviewer’s suggestion and have included more information regarding the real datasets in the manuscript.

Reviewer: In the section titled “ Performance in real datasets, ” It is unclear why the results of FALSCONmeta are regarded as the gold standard.

Authors: The FALCON-meta was regarded as the gold standard in the context of this manuscript as other methods used to infer metagenomic composition of sequenced reads (e.g. Kraken2, Centrifuge) do not provide the direct accession code for the most suitable reference, indicating only which entities are contained within a sample. Having the accession codes of the viruses contained in the datasets is particularly important in this study, as we use them to retrieve the most suitable references from the viral database included in the benchmark. Moreover, FALCON-meta sorts the output by similarity and minimises the underestimation of similarity (based on data compression, an approximation of the Kolmogorov Complexity [1]).

Reviewer: The phrase “availability of viral sequences”Seemingly suggests that the author intends to reference viral sequencing data or metagenomics data. Currently, it reads as though it refers to viral reference genomes.

Authors: We thank the Reviewer for the suggestion and we have made the corresponding change in the manuscript.

References
[1] Ming Li and Paul Vitanyi. An introduction to Kolmogorov complexity and its applications. Vol. 3. Springer, 2008.
[2] Lari Pyöriä et al. “Intra-host genomic diversity and integration landscape of human tissue-resident DNA virome”. In: Nucleic Acids Research 52.21 (2024), pp. 13073–13093.
[3] Lari Pyöriä et al. “Unmasking the tissue-resident eukaryotic DNA virome in humans”. In: Nucleic Acids Research 51.7 (2023), pp. 3223–3239.

Cookie Preference Center

We use cookies which are necessary to make our site work. We may also use additional cookies to analyze, improve and personalize our content and your digital experience. For more information, see our Cookie Policy.

You may choose not to allow some types of cookies. However, blocking some types may impact your experience of our site and the services we are able to offer. See the different category headings below to find out more or change your settings.

Allow all

Manage Consent Preferences

Strictly Necessary Cookies

Always active

These cookies are necessary for the website to function and cannot be switched off in our systems. They are usually only set in response to actions made by you which amount to a request for services, such as setting your privacy preferences, logging in or filling in forms. You can set your browser to block or alert you about these cookies, but some parts of the site will not then work.

Functional Cookies

These cookies enable the website to provide enhanced functionality and personalisation. They may be set by us or by third party providers whose services we have added to our pages. If you do not allow these cookies then some or all of these services may not function properly.

Performance Cookies

These cookies allow us to count visits and traffic sources so we can measure and improve the performance of our site. They help us to know which pages are the most and least popular and see how visitors move around the site.

Targeting Cookies

These cookies may be set through our site by our advertising partners. They may be used by those companies to build a profile of your interests and show you relevant adverts on other sites. If you do not allow these cookies, you will experience less targeted advertising.

Cookie List

Clear

Apply Cancel
Consent Leg.Interest

Confirm my choices

Powered by onetrust
