# Supplementary material for: An evaluation of computational methods for reconstruction of human viral DNA genomes
Source: Gigascience. 2025 Dec 26;15:giaf159. doi: 10.1093/gigascience/giaf159 (PMC12927430; doi:10.1093/gigascience/giaf159)
Supplement: giaf159_Authors_Response_To_Reviewer_Comments_revision_1 [file giaf159_authors_response_to_reviewer_comments_revision_1.pdf]

Author's Response To Reviewer Comments

Dear Editor,
We greatly appreciate the opportunity given to revise our manuscript. We would like to thank the reviewer, whose suggestions allowed us to improve our manuscript. We have now addressed the point raised by the reviewer, as outlined in blue in this revision letter. Following the reviewer's suggestions, appropriate changes have been introduced to the manuscript, shown also in blue, including the addition of information regarding the datasets used and the addition of a new section describing the effects that different error rates can have on the performance of the reconstruction tools considered. Additionally, some changes were made to the section "Read length" of the manuscript. We hope we have been able to address the reviewer's concerns and that our manuscript is now suitable for publication at GigaScience.

Reviewer 1
Reviewer: I have reviewed the updated manuscript by Sousa et al., titled "An evaluation of computational methods for reconstruction of human viral DNA genomes". The authors have addressed the revisions suggested by three reviewers and these have been carefully considered to improve the manuscript. In my opinion, the authors have succeeded in improving the quality of the paper and refining the title to better reflect the scope of the study. They have provided sufficient and well-reasoned responses to the reviewers' comments, which have either been incorporated into the manuscript or convincingly refuted.
In my opinion, the only exception to the above is the following comment: Reviewer: The error rate can strongly influence the accuracy of reference-based genome reconstructions. Has the effect of error rate been tested or could it affect the results, e.g. are there any tools in the benchmark that are less sensitive to higher error rates? Authors: We appreciate the reviewer's comment. It is not entirely clear whether the term "error rates" refers to sequencing errors, mutation rates, or alignment inaccuracies. However, in our benchmark, we assessed the performance of the reconstruction tools using synthetic datasets in which the viral genomes were systematically mutated to include between 0% and 15% single nucleotide polymorphisms (SNPs). This setup allowed us to evaluate how the tools handle increasing levels of sequence divergence from the reference. While this does not directly simulate sequencing errors, it does provide insight into each tool's robustness to genetic variability, which can resemble the impact of elevated error rates in practical scenarios. A more detailed study focused specifically on sequencing error sensitivity could be an interesting direction for future work.
I realise that I have not clearly reflected to sequencing error rates in my previous comment. While the use of simulated data with varying degrees of polymorphism has adequately assessed the robustness of variant calling under conditions of higher genetic distances, sequencing error rates present a different challenge. Polymorphisms occur as consistent changes at the same genomic positions, whereas sequencing errors are largely random and can also increase the false-positive rate of variant calls. Another reviewer also mentioned that there are different protocols to carry out the sequencing experiments. Some of them have very different error profiles. Error rate is an important factor for NGS libraries (sometimes linked to the exact library preparation protocol), and the resilience of different pipelines to sequencing "noise" can be a critical feature. Evaluating the impact of different levels of sequencing errors would therefore provide further insight into the performance of pipelines, particularly in terms of the reliability of variant calling and filtering approaches given different levels of sequencing errors. Personally, I would expect differences between pipelines that rely on different variant callers. I understand that the study focused on DNA viruses, which are generally sequenced using similar protocols and therefore subject to comparable error rates. Nevertheless, I believe that the sequencing error rate should be explicitly described in the Methods section. Authors should aim to clarify the underlying assumptions of the read simulations so that readers can better assess the applicability of the results to datasets generated with different library preparation protocols.
After addressing this point, I support the publication of this manuscript in GigaScience as it would be a valuable contribution to the journal.

Authors: We appreciate the reviewer's clarification regarding this comment, which allowed us to improve the manuscript.
To simulate sequencing errors, we had previously only used the ART default quality profiles. As such, the quality profile HiSeq 2500 for all datasets except for DS61 and DS62, with DS61 being simulated with the

Genome Analyzer II quality profile, and DS62 being simulated with the MiSeq v1 quality profile. To further test the effect that error rates can have on the performance of the reconstruction tools we added a new section, “Error rates”, where we compare the performance of the reconstruction tools in eight datasets with error rates between 0.0 and 0.05 and a depth coverage of 5x and 40x. We have added a clarification about the quality profiles and error rates used in the section “Reconstruction benchmark methodology” of the manuscript and the full characteristics and results obtained using these datasets were included either in the manuscript or in the Supplementary Material.

---

em Editorial Manager®

Cookie Preference Center

We use cookies which are necessary to make our site work. We may also use additional cookies to analyze, improve and personalize our content and your digital experience. For more information, see our Cookie Policy.

You may choose not to allow some types of cookies. However, blocking some types may impact your experience of our site and the services we are able to offer. See the different category headings below to find out more or change your settings.

Allow all

Manage Consent Preferences

Strictly Necessary Cookies

Always active

These cookies are necessary for the website to function and cannot be switched off in our systems. They are usually only set in response to actions made by you which amount to a request for services, such as setting your privacy preferences, logging in or filling in forms. You can set your browser to block or alert you about these cookies, but some parts of the site will not then work.

Functional Cookies

These cookies enable the website to provide enhanced functionality and personalisation. They may be set by us or by third party providers whose services we

have added to our pages. If you do not allow these cookies then some or all of these services may not function properly.

Performance Cookies

These cookies allow us to count visits and traffic sources so we can measure and improve the performance of our site. They help us to know which pages are the most and least popular and see how visitors move around the site.

Targeting Cookies

These cookies may be set through our site by our advertising partners. They may be used by those companies to build a profile of your interests and show you relevant adverts on other sites. If you do not allow these cookies, you will experience less targeted advertising.

Cookie List

Clear

Apply Cancel
Consent Leg.Interest

Confirm my choices

Powered by onetrust
