# Supplementary material for: An evaluation of computational methods for reconstruction of human viral DNA genomes
Source: Gigascience. 2025 Dec 26;15:giaf159. doi: 10.1093/gigascience/giaf159 (PMC12927430; doi:10.1093/gigascience/giaf159)
Supplement: giaf159_Reviewer_1_Report_original_submission — Levente Laczkó -- 2/23/2025 [file giaf159_reviewer_1_report_original_submission.pdf]

Reviewer Report

Title: An evaluation of computational methods for reconstruction of human viral DNA genomes

Version: Original Submission **Date:** 2/23/2025

Reviewer name: Levente LaczkÃ³

Reviewer Comments to Author:

I reviewed the manuscript titled "An evaluation of computational methods for reconstruction of human viral genomes" by Sousa et al. The authors reviewed different tools for the reconstruction of viral genomes and developed a benchmarking framework to measure the performance of the different tools. The benchmarking was performed with both synthetic and real sequencing data, and the authors provide recommendations for different scenarios. The benchmarking framework developed with Bash is also made available on GitHub, providing the scientific community a good example to increase reproducibility. The analysis steps are also clearly described in the manuscript. Independent benchmarks, such as presented in the manuscript, are valuable contributions to the scientific literature and help to select the right tool for different tasks. The manuscript is clearly structured and well written, and the results are appropriately presented with rich supplementary material. I definitely recommend the publication of the manuscript in GigaScience. However, I have some questions that I think should be addressed before publishing the final version to further improve the manuscript.

The authors describe that multiple strains may be present within a single infection. Indeed, the variability of strains within a single infection may be particularly important for some viruses. QuRe, ViSpA, SAVAGE and ViQUF are explicitly designed to find quasispecies. Are there any other tools in the benchmark that can predict whether samples are heterogeneous (or whose results can be used to infer this)?

The authors have used the human mitochondrion as a source of contamination to test whether the tools are sensitive to it. Is there a reason why only the mitochondrion was used for this test and other, perhaps random, human DNA fragments were not?

The error rate can strongly influence the accuracy of reference-based genome reconstructions. Has the effect of error rate been tested or could it affect the results, e.g. are there any tools in the benchmark that are less sensitive to higher error rates?

In the synthetic dataset, the coverage ranged from 2-40Ã—. This range represents scenarios where the viral copy number is low, but especially if the viral DNA was enriched before sequencing, the coverage could be much higher. Is there a reason to specifically choose 40x coverage as the highest coverage value? I agree that low coverage is a difficult challenge, but checking the performance of different tools at high read depth can help readers to choose the right tool for these use cases if there is a difference in the performance of the tools at e.g. >100x coverage.

The authors correctly describe that the complexity of genomes can be a challenge for accurate genome reconstruction. Assessing the complexity (e.g. repetitive content ratio, GC ratio) of the genomes used in the synthetic dataset can add additional value to the results by showing how different tools perform on genomes of different complexity.

Some reference-based tools (QVG, TRACESPipe, TRACESPipeLite and V-pipe) produced results with many gaps. Could the different approach be a reason for how they deal with low coverage regions? QVG, for example, masks positions with low sequencing depth to increase the specificity of the search for polymorphisms. Can the gaps be explained by the variation in sequencing depth, i.e. could the gaps be linked to genomic regions with very low or very high sequencing depth?
I agree that benchmarking real datasets without the correct original sequence is a difficult task. I believe that showing the coverage and completeness (e.g. the ratio of the reconstructed length of the reference genome) can be an additional and useful information for the reader to choose the right tool for different tasks. The expected length of the viral genomes could be determined by the length of the reference genomes used, based on the classification of FALCON-meta, and in the case of de novo pipelines, the scaffolds that do not match the references could be classified using e.g. kraken2. This could show how complete the reconstructed genomes are and whether there are other viral genomes in the samples that FALCON-meta missed but still represent valuable information. Supplementary Figures S143-S146 show the number of reconstructed bases with and without gaps, but I think that this experiment should be emphasised more in the main text and that the ratio of reconstructed bases to the expected genome sizes might be more informative than just the total number of reconstructed base pairs.
1) Are the methods appropriate to the aims of the study, are they well described, and are necessary controls included?
Yes
2) Are the conclusions adequately supported by the data shown?
Yes
3) Please indicate the quality of language in the manuscript. Does it require a heavy editing for language and clarity?
The language is well understandable
4) Are you able to assess all statistics in the manuscript, including the appropriateness of statistical tests used?
Yes

Methods

Are the methods appropriate to the aims of the study, are they well described, and are necessary controls included? Choose an item.

Conclusions

Are the conclusions adequately supported by the data shown? Choose an item.

Reporting Standards

Does the manuscript adhere to the journal's guidelines on minimum standards of reporting? Choose an item.

Choose an item.

Statistics

Are you able to assess all statistics in the manuscript, including the appropriateness of statistical tests used? Choose an item.

Quality of Written English

Please indicate the quality of language in the manuscript: Choose an item.

Declaration of Competing Interests

Please complete a declaration of competing interests, considering the following questions:

- Have you in the past five years received reimbursements, fees, funding, or salary from an organisation that may in any way gain or lose financially from the publication of this manuscript, either now or in the future?
- Do you hold any stocks or shares in an organisation that may in any way gain or lose financially from the publication of this manuscript, either now or in the future?
- Do you hold or are you currently applying for any patents relating to the content of the manuscript?
- Have you received reimbursements, fees, funding, or salary from an organization that holds or has applied for patents relating to the content of the manuscript?
- Do you have any other financial competing interests?
- Do you have any non-financial competing interests in relation to this paper?

If you can answer no to all of the above, write 'I declare that I have no competing interests' below. If your reply is yes to any, please give details below.

I declare that I have no competing interests

I agree to the open peer review policy of the journal. I understand that my name will be included on my report to the authors and, if the manuscript is accepted for publication, my named report including any attachments I upload will be posted on the website along with the authors' responses. I agree for my report to be made available under an Open Access Creative Commons CC-BY license (http://creativecommons.org/licenses/by/4.0/). I understand that any comments which I do not wish to be included in my named report can be included as confidential comments to the editors, which will not be published.

Choose an item.

To further support our reviewers, we have joined with Publons, where you can gain additional credit to further highlight your hard work (see: https://publons.com/journal/530/gigascience). On publication of this paper, your review will be automatically added to Publons, you can then choose whether or not to claim your Publons credit. I understand this statement.

Yes Choose an item.
