# Supplementary material for: An evaluation of computational methods for reconstruction of human viral DNA genomes
Source: Gigascience. 2025 Dec 26;15:giaf159. doi: 10.1093/gigascience/giaf159 (PMC12927430; doi:10.1093/gigascience/giaf159)
Supplement: giaf159_Reviewer_1_Report_revision_1 — Levente Laczkó -- 9/12/2025 [file giaf159_reviewer_1_report_revision_1.pdf]

Reviewer Report

Title: An evaluation of computational methods for reconstruction of human viral DNA genomes

Version: Revision 1 **Date:** 9/12/2025

Reviewer name: Levente LaczkÃ³

Reviewer Comments to Author:

I have reviewed the updated manuscript by Sousa et al., titled "An evaluation of computational methods for reconstruction of human viral DNA genomes". The authors have addressed the revisions suggested by three reviewers and these have been carefully considered to improve the manuscript. In my opinion, the authors have succeeded in improving the quality of the paper and refining the title to better reflect the scope of the study. They have provided sufficient and well-reasoned responses to the reviewers' comments, which have either been incorporated into the manuscript or convincingly refuted.
In my opinion, the only exception to the above is the following comment:
Reviewer: The error rate can strongly influence the accuracy of reference-based genome reconstructions.
Has the effect of error rate been tested or could it affect the results, e.g. are there any tools in the benchmark
that are less sensitive to higher error rates?
Authors: We appreciate the reviewer's comment. It is not entirely clear whether the term "error rates" refers to sequencing errors, mutation rates, or alignment inaccuracies. However, in our benchmark, we assessed the performance of the reconstruction tools using synthetic datasets in which the viral genomes
were systematically mutated to include between 0% and 15% single nucleotide polymorphisms (SNPs).
This setup allowed us to evaluate how the tools handle increasing levels of sequence divergence from the
reference. While this does not directly simulate sequencing errors, it does provide insight into each tool's
robustness to genetic variability, which can resemble the impact of elevated error rates in practical scenarios.
A more detailed study focused specifically on sequencing error sensitivity could be an interesting direction
for future work.
I realise that I have not clearly reflected to sequencing error rates in my previous comment. While the use of simulated data with varying degrees of polymorphism has adequately assessed the robustness of variant calling under conditions of higher genetic distances, sequencing error rates present a different challenge. Polymorphisms occur as consistent changes at the same genomic positions, whereas sequencing errors are largely random and can also increase the false-positive rate of variant calls.
Another reviewer also mentioned that there are different protocols to carry out the sequencing experiments. Some of them have very different error profiles. Error rate is an important factor for NGS

libraries (sometimes linked to the exact library preparation protocol), and the resilience of different pipelines to sequencing "noise" can be a critical feature. Evaluating the impact of different levels of sequencing errors would therefore provide further insight into the performance of pipelines, particularly in terms of the reliability of variant calling and filtering approaches given different levels of sequencing errors. Personally, I would expect differences between pipelines that rely on different variant callers.
I understand that the study focused on DNA viruses, which are generally sequenced using similar protocols and therefore subject to comparable error rates. Nevertheless, I believe that the sequencing error rate should be explicitly described in the Methods section. Authors should aim to clarify the underlying assumptions of the read simulations so that readers can better assess the applicability of the results to datasets generated with different library preparation protocols.
After addressing this point, I support the publication of this manuscript in GigaScience as it would be a valuable contribution to the journal.

Methods

Are the methods appropriate to the aims of the study, are they well described, and are necessary controls included? Choose an item.

Conclusions

Are the conclusions adequately supported by the data shown? Choose an item.

Reporting Standards

Does the manuscript adhere to the journal's guidelines on [minimum standards of reporting?]() Choose an item.

Choose an item.

Statistics

Are you able to assess all statistics in the manuscript, including the appropriateness of statistical tests used? Choose an item.

Quality of Written English

Please indicate the quality of language in the manuscript: Choose an item.

Declaration of Competing Interests

Please complete a declaration of competing interests, considering the following questions:

- Have you in the past five years received reimbursements, fees, funding, or salary from an organisation that may in any way gain or lose financially from the publication of this manuscript, either now or in the future?

- Do you hold any stocks or shares in an organisation that may in any way gain or lose financially from the publication of this manuscript, either now or in the future?
- Do you hold or are you currently applying for any patents relating to the content of the manuscript?
- Have you received reimbursements, fees, funding, or salary from an organization that holds or has applied for patents relating to the content of the manuscript?
- Do you have any other financial competing interests?
- Do you have any non-financial competing interests in relation to this paper?

If you can answer no to all of the above, write 'I declare that I have no competing interests' below. If your reply is yes to any, please give details below.

I declare that I have no competing interests

I agree to the open peer review policy of the journal. I understand that my name will be included on my report to the authors and, if the manuscript is accepted for publication, my named report including any attachments I upload will be posted on the website along with the authors' responses. I agree for my report to be made available under an Open Access Creative Commons CC-BY license (http://creativecommons.org/licenses/by/4.0/). I understand that any comments which I do not wish to be included in my named report can be included as confidential comments to the editors, which will not be published.

Choose an item.

To further support our reviewers, we have joined with Publons, where you can gain additional credit to further highlight your hard work (see: https://publons.com/journal/530/gigascience). On publication of this paper, your review will be automatically added to Publons, you can then choose whether or not to claim your Publons credit. I understand this statement.

Yes Choose an item.
