# Supplementary material for: An evaluation of computational methods for reconstruction of human viral DNA genomes
Source: Gigascience. 2025 Dec 26;15:giaf159. doi: 10.1093/gigascience/giaf159 (PMC12927430; doi:10.1093/gigascience/giaf159)
Supplement: giaf159_Reviewer_2_Report_original_submission — Anton Korobeynikov, Ph.D -- 4/23/2025 [file giaf159_reviewer_2_report_original_submission.pdf]

Reviewer Report

Title: An evaluation of computational methods for reconstruction of human viral DNA genomes

Version: Original Submission **Date:** 4/23/2025

Reviewer name: Anton Korobeynikov, Ph.D

Reviewer Comments to Author:

Sousa et all in their article provided an attempt to review available computational methods to assemble human viruses from real and simulated data. While the review itself seems to be valuable, we believe that the exposition contains several methodological issues that renders it somehow useful. We will try to summarize these high-level issues instead of going down through small details here and there.

To our surprise, the authors (who are also authors of two tools under evaluation) somehow do not distinguish different kinds of input data that is pretty important as it effectively determines the choice of the tool. Clearly, there is no silver bullet here and there is no single push-button solution that could handle universally all kinds of input data. It is very strange that e.g. authors do not distinguish between DNA and RNA viruses. The sequencing approaches for these kinds of data is very different, they have entirely different internal structure and organization and the challenges associated with assembly process are different. This is summarized well in e.g. (Grabherr et al, 2011), (Bushmanova et al, 2019) and (Meleshko et al, 2022) among the others. To add second dimension here: we can have more or less "pure" viral culture, or metagenome / metavirome, or some highly divergent metavirome (e.g. in case of HIV or other viruses undergo reverse transcription). The host contamination is more sound for DNA viruses, etc. So, to summarize - all (very complex!) variations of input data were somehow folded into a single "human viruses" title, which is really misleading. It is the properties of input data that should guide the choice of the appropriate tool.

Next, the choice of tools is also somewhat questionable. Some well-known tools like PRICE or VICUNA were omitted. Ok, IVA is here and this might be enough for "classical" viral assemblies. But then generic-purpose metagenome assembler metaSPAdes is considered without other choices. How about MEGAHIT? for RNA viral data - what's about Trinity or rnaSPAdes? It was strange to see coronaSPAdes mentioned, while it is essentially rnaviralSPAdes + set of SARS-Cov 2 HMMs. Why not just rnaviralSPAdes if we already know we're not going to reconstruct coronaviral data? Another thing is that the majority of tools are tuned for a particular tasks: there are tools for quasispecies assembly, so they would aim to preserve all the variation present. Metagenomic assemblers aim to provide a backbone consensus of a metagenome. Usually assemblers for RNA data aim for the reconstruction as many transcripts as possible (so their "duplication rate" might be misleading). metaviralSPAdes aims to reconstruct full-length circular and linear viruses from complex contaminated metagenomes, so it could be very conservative, etc. It feels like the benchmarking compares something warm with something soft giving misleading guidance to the reader.

Finally, it is the year 2025, but the pipeline is just a huge pile of shell scripts that install tools (sometimes outdated as far as I can see, e.g. it uses SPAdes 3.13.0 that was released more than 5 years ago) often globally, sometimes only via conda. It could hardly be named as "reproducible" pipeline: error handling

is quite non-existing, if something happens in between the user might end with some partially resolved state. There are lots of frameworks and approaches developed recently that provide all necessary details like job isolation, installation, restart & checkpointing, data acquisition, etc. To put things simple: why everything is done manually via hand-written shell scripts and not based on say, Nextflow? There are lots of ready modules from `nf-core` that one could just reuse. Likely some ideas could be taken from https://github.com/nf-core/viralrecon/ and other pipelines available there.

Methods

Are the methods appropriate to the aims of the study, are they well described, and are necessary controls included? Choose an item.

Conclusions

Are the conclusions adequately supported by the data shown? Choose an item.

Reporting Standards

Does the manuscript adhere to the journal's guidelines on [minimum standards of reporting?] Choose an item.

Choose an item.

Statistics

Are you able to assess all statistics in the manuscript, including the appropriateness of statistical tests used? Choose an item.

Quality of Written English

Please indicate the quality of language in the manuscript: Choose an item.

Declaration of Competing Interests

Please complete a declaration of competing interests, considering the following questions:

- Have you in the past five years received reimbursements, fees, funding, or salary from an organisation that may in any way gain or lose financially from the publication of this manuscript, either now or in the future?
- Do you hold any stocks or shares in an organisation that may in any way gain or lose financially from the publication of this manuscript, either now or in the future?
- Do you hold or are you currently applying for any patents relating to the content of the manuscript?
- Have you received reimbursements, fees, funding, or salary from an organization that holds or has applied for patents relating to the content of the manuscript?

- Do you have any other financial competing interests?
- Do you have any non-financial competing interests in relation to this paper?

If you can answer no to all of the above, write 'I declare that I have no competing interests' below. If your reply is yes to any, please give details below.

I am the author of some assemblers the author compare their own with. Though, I believe this does not imply a competing interest.

I agree to the open peer review policy of the journal. I understand that my name will be included on my report to the authors and, if the manuscript is accepted for publication, my named report including any attachments I upload will be posted on the website along with the authors' responses. I agree for my report to be made available under an Open Access Creative Commons CC-BY license (http://creativecommons.org/licenses/by/4.0/). I understand that any comments which I do not wish to be included in my named report can be included as confidential comments to the editors, which will not be published.

Choose an item.

To further support our reviewers, we have joined with Publons, where you can gain additional credit to further highlight your hard work (see: https://publons.com/journal/530/gigascience). On publication of this paper, your review will be automatically added to Publons, you can then choose whether or not to claim your Publons credit. I understand this statement.

Yes Choose an item.
