# Supplementary material for: An evaluation of computational methods for reconstruction of human viral DNA genomes
Source: Gigascience. 2025 Dec 26;15:giaf159. doi: 10.1093/gigascience/giaf159 (PMC12927430; doi:10.1093/gigascience/giaf159)
Supplement: giaf159_Reviewer_3_Report_original_submission — Serghei Mangul -- 4/23/2025 [file giaf159_reviewer_3_report_original_submission.pdf]

Reviewer Report

Title: An evaluation of computational methods for reconstruction of human viral DNA genomes

Version: Original Submission **Date:** 4/23/2025

Reviewer name: Serghei Mangul

Reviewer Comments to Author:

The paper is well written and provides a valuable contribution to the field. My only concern pertains to the real data utilized, which lacks a gold standard. Consequently, I question whether the real data adds significant value to the analysis, given the absence of a gold standard.
Major comments:1. What are the types of data used in the manuscript? Is it solely metagenomics data? If so, it would be beneficial to clarify this in the abstract and potentially in the title
2. . 2. Was the real data comprised of metagenomics? It would be advantageous to include some text explaining the nature of the data
3. . 3. In the section titled " Performance in real datasets, " It is unclear why the results of FALSCON-meta are regarded as the gold standard
. Minor comments:1. The phrase "availability of viral sequences"Seemingly suggests that the author intends to reference viral sequencing data or metagenomics data. Currently, it reads as though it refers to viral reference genomes.

Methods

Are the methods appropriate to the aims of the study, are they well described, and are necessary controls included? Choose an item.

Conclusions

Are the conclusions adequately supported by the data shown? Choose an item.

Reporting Standards

Does the manuscript adhere to the journal's guidelines on [minimum standards of reporting?]() Choose an item.

Choose an item.

Statistics

Are you able to assess all statistics in the manuscript, including the appropriateness of statistical tests used? Choose an item.

Quality of Written English

Please indicate the quality of language in the manuscript: Choose an item.

Declaration of Competing Interests

Please complete a declaration of competing interests, considering the following questions:

- Have you in the past five years received reimbursements, fees, funding, or salary from an organisation that may in any way gain or lose financially from the publication of this manuscript, either now or in the future?
- Do you hold any stocks or shares in an organisation that may in any way gain or lose financially from the publication of this manuscript, either now or in the future?
- Do you hold or are you currently applying for any patents relating to the content of the manuscript?
- Have you received reimbursements, fees, funding, or salary from an organization that holds or has applied for patents relating to the content of the manuscript?
- Do you have any other financial competing interests?
- Do you have any non-financial competing interests in relation to this paper?

If you can answer no to all of the above, write 'I declare that I have no competing interests' below. If your reply is yes to any, please give details below.

I declare that I have no competing interests

I agree to the open peer review policy of the journal. I understand that my name will be included on my report to the authors and, if the manuscript is accepted for publication, my named report including any attachments I upload will be posted on the website along with the authors' responses. I agree for my report to be made available under an Open Access Creative Commons CC-BY license (http://creativecommons.org/licenses/by/4.0/). I understand that any comments which I do not wish to be included in my named report can be included as confidential comments to the editors, which will not be published.

Choose an item.

To further support our reviewers, we have joined with Publons, where you can gain additional credit to further highlight your hard work (see: https://publons.com/journal/530/gigascience). On publication of this paper, your review will be automatically added to Publons, you can then choose whether or not to claim your Publons credit. I understand this statement.

Yes Choose an item.
